# Hepatitis B virus X induces inflammation and cancer in mice liver through dysregulation of cytoskeletal remodeling and lipid metabolism

**DOI:** 10.18632/oncotarget.12372

**Published:** 2016-09-30

**Authors:** Zhongwei Xu, Linghui Zhai, Tailong Yi, Huiying Gao, Fengxu Fan, Yanchang Li, Youliang Wang, Ning Li, Xiaohua Xing, Na Su, Feilin Wu, Lei Chang, Xiuli Chen, Erhei Dai, Chao Zhao, Xiao Yang, Chunping Cui, Ping Xu

**Affiliations:** ^1^ State Key Laboratory of Proteomics, Beijing Proteome Research Center, National Engineering Research Center for Protein Drugs, National Center for Protein Sciences Beijing, Institute of Radiation Medicine, Beijing, 102206, P.R. China; ^2^ Beijing Institute of Bioengineering, Beijing, 100071, P. R. China; ^3^ Key Laboratory of Combinatorial Biosynthesis and Drug Discovery, Ministry of Education and Wuhan University School of Pharmaceutical Sciences, Wuhan, 430072, P. R. China; ^4^ Anhui Medical University, Hefei, 230032, China; ^5^ Key Laboratory of Medical Molecular Virology, School of Basic Medical Sciences, and Research Center on Aging and Medicine, Fudan University, Shanghai, 200032, China; ^6^ The Fifth Hospital of Shijiazhuang City, Shijiazhuang, 050021, China; ^7^ Central Laboratory, Logistics University of Chinese People's Armed Police Force, Tianjin, 300309, China

**Keywords:** HBx, SILAM, CDC42, CFL1, ADFP

## Abstract

Hepatitis B virus X protein (HBx) participates in the occurrence and development processes of hepatocellular carcinoma (HCC) as a multifunctional regulation factor. However, the underlying molecular mechanism remains obscure. Here, we describe the use of *p21*^HBx/+^ mouse and SILAM (Stable Isotope Labeling in Mammals) strategy to define the pathological mechanisms for the occurrence and development of HBx induced liver cancer. We systematically compared a series of proteome samples from regular mice, 12- and 24-month old *p21*^HBx/+^ mice representing the inflammation and HCC stages of liver disease respectively and their nontransgenic wild-type (WT) littermates. Totally we identified 22 and 97 differentially expressed proteins out of a total of 2473 quantified proteins. Bioinformatics analysis suggested that the lipid metabolism and CDC42-induced cytoskeleton remodeling pathways were strongly activated by the HBx transgene. Interestingly, the protein-protein interaction MS study revealed that HBx directly interacted with multiple proteins in these two pathways. The same effect of up-regulation of cytoskeleton and lipid metabolism related proteins, including CDC42, CFL1, PPARγ and ADFP, was also observed in the Huh-7 cells transfected with HBx. More importantly, CFL1 and ADFP were specifically accumulated in HBV-associated HCC (HBV-HCC) patient samples, and their expression levels were positively correlated with the severity of HBV-related liver disease. These results provide evidence that HBx induces the dysregulation of cytoskeleton remodeling and lipid metabolism and leads to the occurrence and development of liver cancer. The CFL1 and ADFP might be served as potential biomarkers for prognosis and diagnosis of HBV-HCC.

## INTRODUCTION

Hepatocellular carcinoma (HCC) is one of the major malignancies worldwide [1]. The main cause of HCC is persistent hepatitis B virus (HBV) infection. The HBV genome is composed of four open reading frame coding four viral proteins of HBsAg, HBcAg, HBp, and HBx. Among them, HBx is considered to be one of the distinctly important function of hepatocarcinogenesis as a multi-functional regulation factor [2]. HBx does not directly bind the target motifs of downstream regulated gene, however, it exhibits co-transcription factor activity through interaction with nuclear transcriptional factors, such as NF-κB and activating protein 1(AP-1), and then activates the signal transduction pathways that function primarily in the cytoplasm, including PI3K/AKT, JAK/STAT, and MAPK [3, 4]. Though etiological study focused on the HBx as a co-transcriptional factor using ChIP-seq [5–7], it still obscure for HBx-induced expression level change of regulated proteins, post-translational modification and signaling network in protein level. Many studies have shown that HBx induces fatty acid oxidation and increase the intracellular ATP and NADOH level to induce the resistance to glucose deprivation by activation of AMPK and FAO pathways in live cancer cells [8]. C-terminal region of HBx led to mitochondrial DNA damage by increasing the reactive oxygen species (ROS) production and 8-oxoguanine (8-oxoG) formation [9]. However, the detailed mechanisms of HBx-induced the liver cells malignant transformation was still not clear.

Comparative MS provide a global and comprehensive approach to quantitatively elucidate biological processes, signaling pathways and transduct networks in both physiological and pathological states in the protein level. However, the understanding of the molecular pathogenesis of HBV-HCC has been limited in the field because the employing of patients with various genetic backgrounds and lifestyles as well as *in vitro* studies using HCC cell lines. Hence, the appropriate animal models of HCC that permit longitudinal studies from mild inflammation to HCC stages in a homogenous genetic background under controlled environmental conditions are extremely useful in the proteomics (MS)-based research for biomarkers or critical proteins participated in hepatocarcinogenesis. The HBx transgenic models containing the different integrated locus had been applied in previous studies, including the major urinary protein, β-globin, Albumin, antithrombin III and p21 locus [8–11]. Among them, the *p21*^HBx/+^ mice provided the ideal model used widely for HBx functional study and the mechanism of HCC [12]. This is not only because the transferred HBx could lead HCC in mice, but also the classical study confirmed that p21 deficiency did not directly elevated the susceptibility to HCC [13]. So far, stable isotope labeling in mammals (SILAM)-based methods have been classically applied to proteomic analysis because of their well-controlled experimental process and highly accurate quantitation feature compared with the other methods [14]. Here we performed comparative MS analysis of liver tissues from the control of C57BL/6, a series of HBx-induced disease tissues from mild inflammation to HCC and their nontransgenic WT littermates using SILAM as internal standard. The results were further confirmed by the HBx-interactome that dysregulation of CDC42-induced cytoskeleton remodeling and lipid metabolism-induced by mitochondrial dysfunction might be pivotal factors in HBx-induced progression from inflammation to HCC.

## RESULTS

### Sample preparation for large-scale MS study

**Figure 1:**
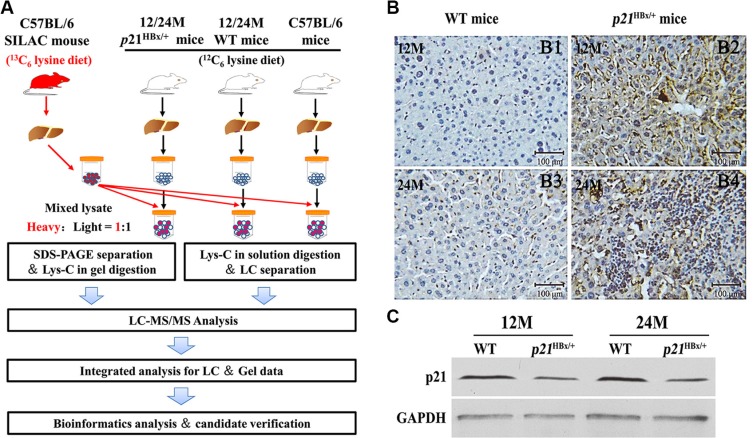
Large-Scale protein profiling of the *p21*^HBx/+^ transgenic mice and their WT littermates with SILAM approach (**A**) Workflow of quantitative MS study for *p21*^HBx/+^ mice and SILAM. Protein extracts from livers of 12M and 24M *p21*^HBx/+^ mice (*n* = 5/group) and their WT littermates (*n* = 5/group) were mixed at 1:1 ratio with liver proteins prepared from SILAM and separated by SDS-PAGE. (**B**) HBx protein level in 12M and 24M *p21*^HBx/+^ mice and WT littermates were analyzed by IHC. (B1) 12M WT; (B2) 12M *p21*^HBx/+^ mice; (B3) 24M WT; (B4) 24M *p21*^HBx/+^ mice; (**C**) The p21 protein level of 12M and 24M *p21*^HBx/+^ mice and their WT littermates was analyzed by WB.

Yang. *et al*. demonstrated that *p21*^HBx/+^ mice exhibited the high rate of occurrence and development process from inflammation to HCC, which had been served as a successful and widely applied animal model to mimic HBV-related HCC with no relationship between p21-null and the development of liver cancer [12, 13]. To decipher the molecular mechanism underlying HBx-induced liver disease from mild inflammation to HCC, we designed a quantitative MS strategy to compare liver tissue samples from the control of C57BL/6, inflammation-stage, and HCC-stage animals (Figure 1A). Equal amounts of liver protein from five mice in each group were pooled to reduce the effects of individual variation [15]. The SILAM with 97.0% labeling efficiency was served as internal standard (Supplementary Figure S1). Expression of HBx protein in the transgenic livers at 12M and 24M ages was confirmed by IHC using an HBx polyclonal antibody from Prof. Akihide Ryo. Most of the *p21*^HBx/+^ mice samples continuously express HBx protein. However, a lower proportion of cells within the 12M *p21*^HBx/+^ mice expressing HBx protein were observed compared with liver tissue of WT littermate mice, and HBx protein level of 24M *p21*^HBx/+^ mice significantly increased compared with the samples of 12M *p21*^HBx/+^ mice (Figure 1B). The p21 expression in hepatocytes of *p21*^HBx/+^ transgenic livers was decreased just above 50% compared with those of WT mice, which avoided the disruption of p21-null affecting HBx function in liver (Figure 1C).

### Quantitative comparison of samples from control of C57BL/6, 12M and 24M p21^HBx/+^ mice

**Figure 2:**
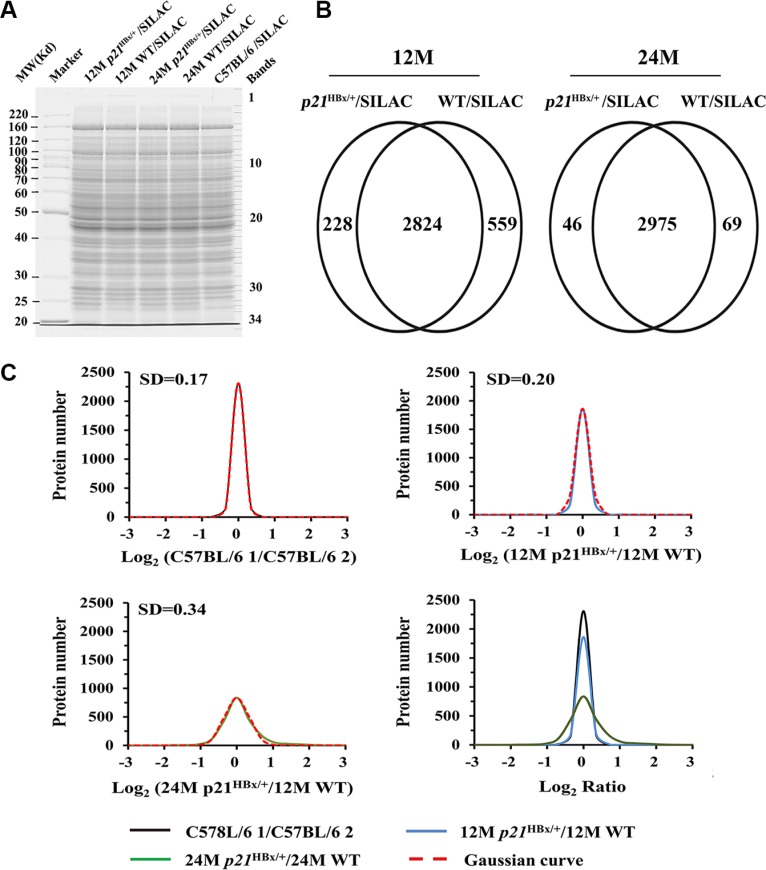
Data analysis for quantified proteins in quantitative MS study (**A**) SDS-PAGE analysis of MS samples. Equal amounts of total liver lysate prepared from *p21*^HBx/+^ mice of different ages and their WT littermates were mixed with liver lysate prepared from SILAM and separated by 10% SDS-PAGE. Each gel lane was sliced into 34 bands for in-gel digestion and LC-MS analysis. (**B**) Overlap comparison of identified proteins between 12M and 24M *p21*^HBx/+^ mice and their corresponding WT littermates. (**C**) (C1–C4) Log2 ratio distributions of quantified proteins between two groups of control mice (*n* = 3128) (C1), 12M *p21*^HBx/+^ mice and their WT littermates (12M WT) (*n* = 2660) (C2), and 24M *p21*^HBx/+^ mice and their WT littermates (24M WT) (*n* = 2466) (C3), respectively. (C4) The comparison of Log2 ratio distributions of quantified proteins in A to C.

Figure 2A shows representative samples from liver samples for each group, which showed similar pattern and intensity (Figure 2A) and highly reproducible LC-MS analysis using gel and off line LC separation strategies (Supplementary Figure S2A and S2B). The numbers of common identified proteins between Gel and LC separation strategy were 3052 and 3383 in 12M *p21*^HBx/+^ mice/SILAM and their WT littermates/SILAM, and the number of pair compared proteins was 2824 between between12M *p21*^HBx/+^ mice/SILAM and their WT littermates/SILAM samples using gel and 2D LC strategies. Meanwhile, the numbers of common identified proteins between Gel and LC separation strategy were 3021 and 3044 proteins in the 24M *p21*^HBx/+^ mice/SILAM and their WT littermates/SILAM using gel and LC strategies, and the number of pair compared proteins proteins between 24M *p21*^HBx/+^ mice/SILAM and their WT littermates/SILAM was 2975 proteins using gel and 2D LC strategies (Figure 2B). A total of 3893 proteins were identified in MS analysis for combining results of 12M and 24M *p21*^HBx/+^ mice and their WT littermates (protein level FDR < 1%). Among them, 2473 proteins were identified across all these groups (Supplementary Figure S2C).

The average correlation coefficient (R^2^) of quantitative data in a series of repeated experiments including of C57BL/6 mice/SILAM, 12M *p21*^HBx/+^ mice/SILAM, 12M WT/SILAM, 24M *p21*^HBx/+^ mice/SILAM, 24M WT/SILAM were 0.92, 0.90, 0.88, 0.91 and 0.90, respectively (Supplementary Figure S3). These results presented that MS data was stable and reliable. The protein numbers applied to the spike-in analysis were 2669, 2225 and 2360 in C57BL/6 1 mice *vs.* C57BL/6 1 mice, 12M *p21*^HBx/+^, *vs.* WT littermate mice and 24M *p21*^HBx/+^ *vs.* WT littermate WT mice, respectively (Supplementary Tables S1, S2 and S3).

Details for quantitative data confirmation and analysis are shown in Supplementary Figure S4. The log2 ratio distributions of quantified proteins in each individual group quantified by SILAM approach were fit to Gaussian curves (Supplementary Figure S5). Because of the same heavy labeled internal standard used in this study, the distribution of log_2_^(C57BL/6 1 *vs.* 57BL/6 2)^, log_2_^(12M^ *p21*^HBx/+ *vs.*12M WT)^ and log_2_^(24M^ *p21*^HBx/+ *vs.* 24M WT)^ were calculated and fitted to Gaussian curves with standard deviations (SDs) of 0.17, 0.20 and 0.34, respectively (1–3 of Figure 2C). As shown in Figure 2C, SD value of distribution curve of 24M *p21*^HBx/+^ mice vs. their WT littermates was increased compared with those of 12M *p21*^HBx/+^ HBx or C57BL/6 control mice (*p* < 0.01). However, SD value of the distribution curve for 12M *p21*^HBx/+^ mice vs. their WT littermates was not significant difference from that of C57BL/6 control group (*p* > 0.05). These results suggest that expression levels of a significant portion of proteins are altered in HBx-induced HCC than those in HBx-induced inflammation.

By setting the quantitative values larger than 0.68 (4 times the SD of the C57BL/6 1 mice *vs.* C57BL/6 2 mice experiments) as threshold for significant up- or down-regulation of proteins in this study, we identified 10 up-regulated and 11 down-regulated proteins in 12M *p21*^HBx/+^ samples compared with their WT littermates. We also identified 244 up-regulated and 32 down-regulated proteins in 24M *p21*^HBx/+^ samples compared with their WT littermates, which was strongly related to HCC stage of HBx-induced liver cancer. The differentially expressed proteins and quantities are shown for 12M and 24M *p21*^HBx/+^ mice in Supplementary Tables S4 and S5, respectively.

### Bioinformatics analysis of the differentially expressed proteins in p21^HBx/+^ mice

Gene Ontology (GO) classification indicates that HBx perturbs the liver proteome in a profound manner, suggesting that HBx is involved in a broad range of cellular processes (Supplementary Figure S6). The biological process analysis showed that actin cytoskeleton organization, oxidation reduction and Rho protein signal transduction processes was heavily activated in 12M *p21*^HBx/+^ samples (Supplementary Figure S6A). Among them, CDC42, ACTN2 and TTN were heavily involved in these biological processes (Supplementary Table S6). However, in 24M *p21*^HBx/+^ samples, with the exception of muscle organ development with even severe effect, we found that proteins involved in actin cytoskeletal organization (e.g.,TLN1 and EZR) and small GTPase-induced signal transduction (e.g., ARFGAP1, SMAP2) were mainly activated (Supplementary Figure S6A, Supplementary Table S7). Importantly, CDC42, a member of small RhoGTPase family, was differentially expressed in both 12M and 24M *p21*^HBx/+^ mice. Dysregulation of CDC42 might promote the cytoskeletal dynamics and cell-extracellular matrix interaction [16]. Cellular component analysis indicated dysregulation of Z-disc formation in 12M *p21*^HBx/+^ samples, and of mitochondrial and actin cytoskeleton in 24M *p21*^HBx/+^ samples (Supplementary Figure S6B, Supplementary Tables S6 and S7). The molecular function analysis of 12M *p21*^HBx/+^ samples mainly focused on Rho/Ras pathway related factor activity (Supplementary Figure S6C, Supplementary Tables S6 and S7). KEGG pathway enrichment analysis was mainly related to focal adhesion and steroid hormone biosynthesis (Supplementary Figure S6D). However, previous research suggests that lipid metabolism disorder was likely due to mitochondrial dysfunction. We reviewed the identified differentially expressed proteins, and found that multiple proteins related to lipid metabolism, such as CYP17A1, ADFP, DHTKD1, and AKR1C18, were increased in 12M *p21*^HBx/+^ samples (Supplementary Table S6). Furthermore, more proteins (43 of 276) in this process were differentially expressed in 24M *p21*^HBx/+^ samples (Supplementary Table S7), including oxidation reduction (e.g., NDUFA5 and HMOX1), fatty acid metabolic process (e.g., PLIN2 and ANXA1), and glucose metabolism (e.g., G6PDX and DHTKD1), possibly resulting in mitochondrial dysfunction. Hence, mitochondrial dysfunction might be another critical factor in HBx-induced progression from inflammation to HCC.

### Profiling of HBx-interacting proteins

**Figure 3:**
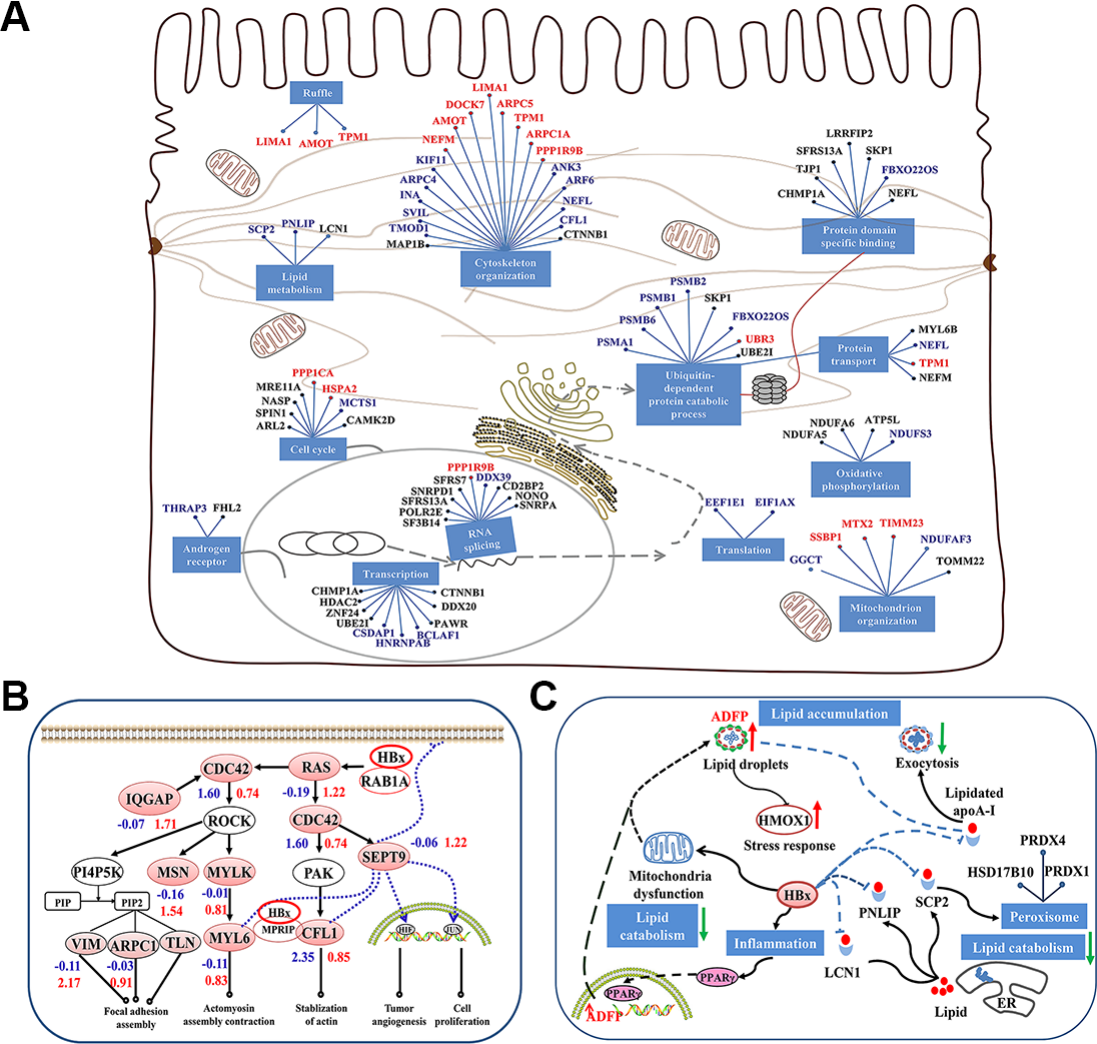
Model of HBx-induced liver carcinogenesis via dysregulation of cytoskeletal remodeling and lipid metabolism (**A**) The functional network of HBx interactors. Solid lines between genes indicate a protein interaction. Cellular mechanisms are indicated in blue boxes. The red indicates the spectra counts (SC) of peptides using MS was more or equal to 5 (SC ≥ 5), the blue indicates that SC was more than 3 and less than 5 (3 < SC < 5), and the black indicates that SC was less than 3 (SC ≤ 3). (**B**) HBx-induced CDC42 signaling promotes cytoskeletal remodeling and carcinogenesis. HBx triggers CDC42 signaling by binding the RAS oncogene, resulting in the activation of ROCK and promoting CFL1 expression. CDC42 also enhances the ability of SEPT9 binding to the assembly of cytoskeleton and filamentous structures, which stabilizes HIF1-α and c-Jun and promotes tumor angiogenesis and cell proliferation. (**C**) HBx induces storage of cellular lipids and disrupts lipid metabolism in liver cells. HBx hampers lipid catabolism by binding ACSF2, HADH, SCP2, PNLIP, and LCN1 and prevents lipid exocytosis by interaction with apoA-I. HBx also disrupts mitochondrial function and increases ADFP expression level via PPARγ activation, resulting in lipid accumulation in liver cells. The blue and red numbers represent the quantitative value in 12 M and 24 M *p21*^HBx/+^ mice on MS analysis, respectively.

We explored the HBx-interacting proteins in H22 cells transfected with lentivirus encoding 3 × FLAG-tagged HBx. A total of 162 proteins were identified from the repeated LC-MS analysis of affinity-purified proteins with anti-Flag antibody (see Supporting Information). These HBx-interacting proteins were mainly related to cytoskeleton (37/162), mitochondrial function (32/162), transcriptional regulation, cell cycle (19/162), oxidative phosphorylation (4/162), and RNA splicing (13/162) (Figure 3A, Supplementary Table S8). The cytoskeleton and mitochondrial function related proteins occupied about half of all the identified HBx-interacting proteins. These results were consistent with our quantitative MS analysis with *p21*^HBx/+^ model, which further confirmed that HBx-induced dysregulation was strongly related to cytoskeletal remodeling and mitochondrial dysfunction.

### Network of HBx-induced dysfunction for cytoskeleton remodeling and lipid metabolism

Utilizing quantitative MS result and information of HBx-interacting proteins, we rebuilt a network for the dysfunction of cytoskeleton remodeling and lipid metabolism induced by HBx (Supplementary Figure S3B and S3C). We found that HBx may trigger CDC42 signaling by activating RAS oncogene, resulting in activation of ROCK and PAK and then promoting the expression of CFL1, ERM, VIM, TLN and MYLK. CDC42 also enhanced the assembly of cytoskeleton and filamentous structures by increasing the ability of SEPT9 binding with CFL1 and Myosin [16]. SEPT9 can be localized to nucleus, where it stabilizes HIF1-α and c-Jun and promotes tumor angiogenesis and cell proliferation [17]. In addition, the interaction between HBx and MRRIP attenuates the phosphorylation process of cytoskeleton and facilitates stress fiber formation (Figure 3B) [18, 19]. We also notice that HBx may hamper lipid catabolism by binding SCP2, PNLIP, and LCN1 and prevents lipid exocytosis by interaction with apoA-I, or interacting with two rate-limiting enzymes involved in fatty acid *β*-oxidation, ACSF2 and HADH located in mitochondria [20–22]. Furthermore, HBx-induced inflammation reaction might increase ADFP expression level via PPARγ activation, resulting in lipid accumulation in liver cells (Figure 3C). Hence, we speculate that HBx induces the malignancy in liver cells through dysregulation of cytoskeletal remodeling and lipid metabolism. This is consistent with accumulation of HMOX1 in liver tissues of 24M *p21*^HBx/+^ mice, suggesting the severe effect of liver injury in the later stage of liver disease.

### Validation of the proteomic results by IHC and WB in p21^HBx/+^ mice Liver

**Figure 4:**
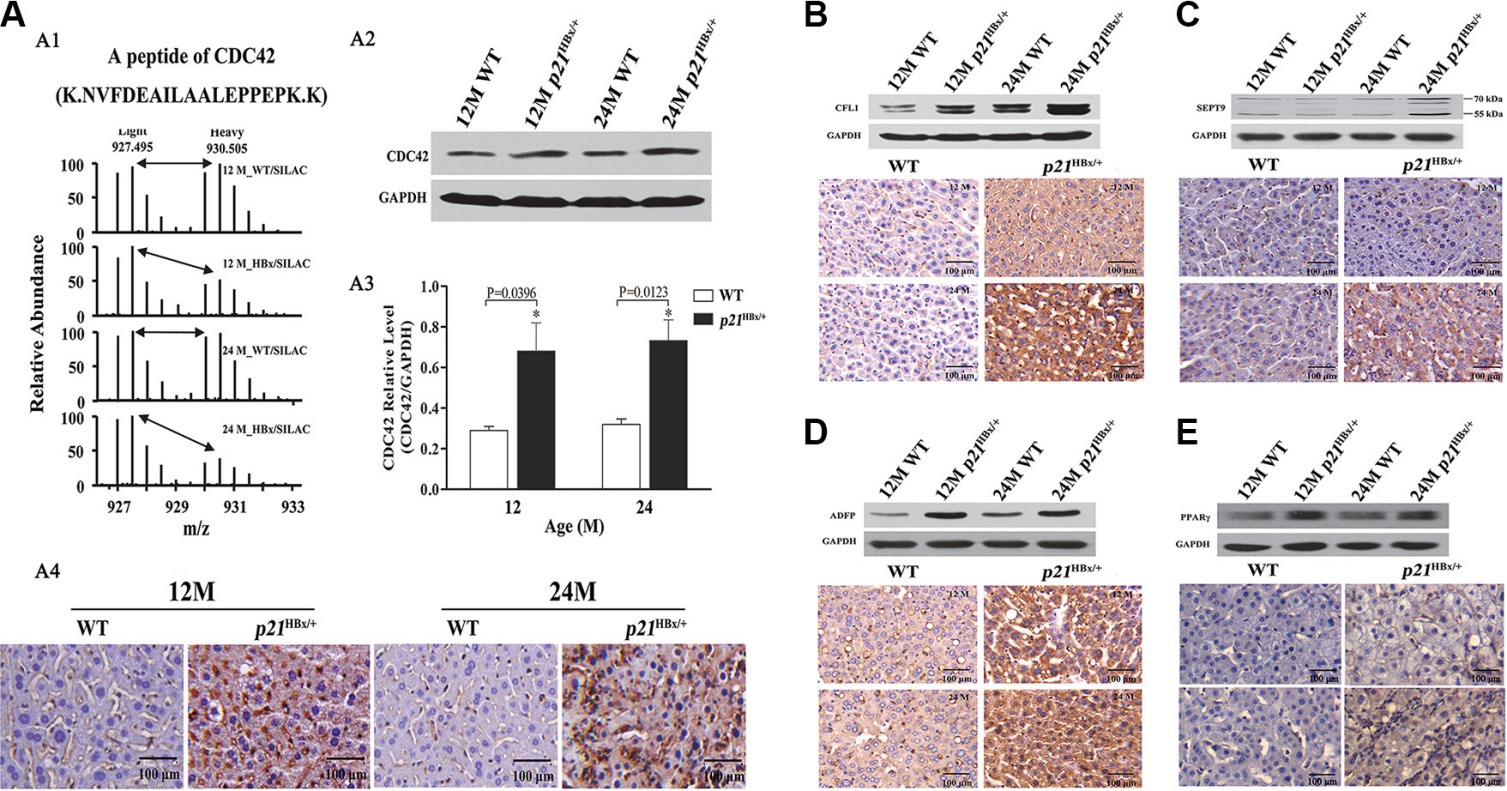
Validation for the amount changed protein CDC42, CFL1, ADFP and PPARγ in p21^HBx/+^mice and WT littermates by WB and IHC (**A**) Quantitative MS analysis of CDC42 abundance in 12M and 24M *p21*^HBx/+^ mice and their corresponding WT littermates. (A1) MS spectra and monoisotopic m/z values of a detected peptide from CDC42 protein are shown. (A2) WB analysis of CDC42 in 12M and 24M *p21*^HBx/+^ mice. GAPDH was used as the loading control. The experiment was repeated three times with similar results. (A3) Quantitative analysis of WB result for CDC42 using GAPDH as a control. Asterisk represents significant difference from control. (A4) IHC analysis of CDC42 in livers of 12M and 24M *p21*^HBx/+^ mice. Proteins of CFL1 (**B**), SEPT9 (**C**), ADFP (**D**), and PPARγ (**E**) in 12M and 24M *p21*^HBx/+^ mice and their WT littermates were analyzed by WB (upper panels) and IHC (lower panels). GAPDH was used as a loading control in WB.

As the activation of CDC42, CFL1, SEPT9, ADFP and PPARγ result in the cytoskeletal remodeling and lipid metabolism caused by transfected HBx in hepatocarcinogenesis, we further validated the quantitative results for these five proteins. As shown in Figure 4A1, Supplementary Tables S4 and S5, we manually checked the intensity of a representative peptide (NVFDEAILAALEPPEPK) for CDC42 in all compared samples. The relative abundance of peptide in both groups of *p21*^HBx/+^ mice was approximately 2-fold higher than that in their WT littermates. We then tested the same tissue samples used for SILAM MS study by IHC and WB analysis. WB found that CDC42 expression level in 12M and 24M *p21*^HBx/+^ mice was almost three fold higher than that in their WT littermates, which is slightly higher than what we found from quantitative MS (Figure 4A2 and 4A3). More importantly, the IHC results showed that the mean optical density (MOD) values of CDC42 in 12M and 24M *p21*^HBx/+^ mice and their WT littermates were respective 216.32 ± 21.11, 207.21 ± 19.32 and 11.31 ± 3.24, 8.22 ± 2.11. CDC42 was highly expressed in cytoplasmic and plasma membrane in both groups of *p21*^HBx/+^ mice, but it was expressed at a lower level in WT littermates (Figure 4A4).

CFL1 binds with both monomeric and filamentous actin and promotes cytoskeletal remodeling to increase cell elasticity. By manually checking the MS data, we found that CFL1 was expressed at an approximately 1.8-fold higher in 24M *p21*^HBx/+^ mice than that in WT littermates (Supplementary Figure S7A), which was the same as in large-scale datasets (Supplementary Tables S4 and S5). IHC and WB results further confirmed that CFL1 expression level in 12M and 24M *p21*^HBx/+^ mice was increased by approximately 2-fold (*p* = 0.0058) and 1.5-fold than in their WT littermates' groups (*p* = 0.0329), respectively (Figure 4B and Supplementary Figure S8A).

SEPT9 polymerizes into hetero-oligomeric protein complexes, resulting in formation of filaments, which could associate with cellular membranes, actin filaments, and microtubules. Actually, SEPT9 also belongs to GTP-binding proteins associated with filamentous structures and cytoskeleton formation, which performs as oncogenes in multiple types of cancer [23]. As the same, we found that SEPT9 increased about 3-fold in 24M *p21*^HBx/+^ than in 12M *p21*^HBx/+^ and their WT littermate mice (Supplementary Tables S4 and S5), which was also confirmed by manually checked MS data (Supplementary Figure S7B), IHC and WB with *p-value* of 0.2697 and 0.0024, respectively (Figure 4C and Supplementary Figure S8B).

ADFP is involved in lipid metabolism. In our SILAM MS study, we found that ADFP decreased by approximately 39% in 24M *p21*^HBx/+^ HBx compared with 12M *p21*^HBx/+^ mice (Supplementary Tables S4 and S5). Manual checking of these results showed that ADFP expression was about 3-fold higher in both 12M and 24M *p21*^HBx/+^ mice than in their WT littermates (Supplementary Figure S7C). Both IHC and WB analysis further confirmed that ADFP in *p21*^HBx/+^ mice was significantly increased compared with WT littermates (*p* = 0.0109 and 0.0173, respectively) (Figure 4D and Supplementary Figure S8C).

Although PPARγ had not been identified in our MS study, it was well established in the literature that it was involved in the regulation of lipid metabolism. The IHC and WB results showed that PPARγ increased in *p21*^HBx/+^ mice compared with their WT littermates (*p* = 0.0129 and 0.0171, respectively) (Figure 4E and Supplementary Figure S8D). This result further confirmed the dysregulation of lipid metabolism in HBx-induced liver disease.

### The HBx promote the accumulation of lipids by activation of PPARγ-induction leading the ADFP expression *in vitro*

**Figure 5:**
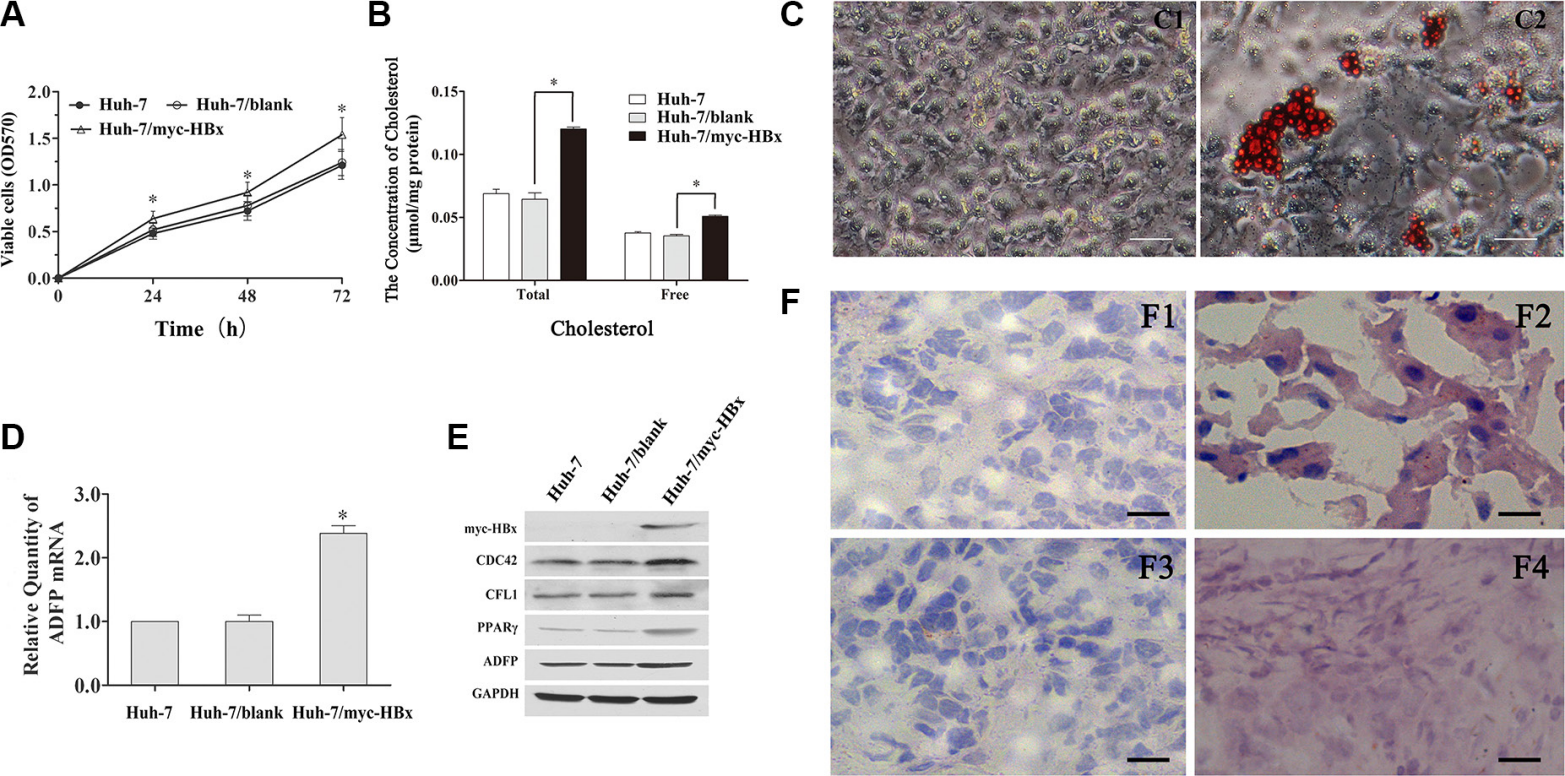
Biological function of HBx on cell viability, cytoskeleton remodeling and lipid metabolism *in vitro* (**A**) The HBx promotes the Huh-7 cells proliferation compared with the control group (*p < 0.05). (**B**) The total and free intercellular cholesterol concentration of Huh-7/myc-HBx cell were significantly increased compared with control (*p < 0.05). (**C**) The lipid droplet was accumulated in Huh-7/myc-HBx cells using the oil red O staining. (**D**) The ADFP mRNA level of Huh-7/myc-HBx cells was significantly increased compared with control group (*p < 0.05). (**E**) The cytoskeleton and lipid metabolism related proteins, including CDC42, CFL1, PPARγ and ADFP in Huh-7/myc-HBx cells were significantly increased compared with control group. (**F**) The lipid droplet was obviously accumulated in 12M and 24M p21^HBx/+^ mice using the oil red O staining.

The Water-soluble tetrazolium salt 1 (WST1) assay was performed to analyze the effects of HBx gene on the viability of Huh-7 cells. As shown in Figure 5A, proliferation ability of Huh-7/myc-HBx cells was higher compared with Huh-7 controls at 24, 48 and 72 h (*p < 0.05). The total and free cholesterol concentration of Huh-7, Huh-7/blank and Huh-7/myc-HBx cell lines were 0.0664 ± 0.0051, 0.0509 ± 0.0009, 0.1202 ± 0.0015 and 0.0354 ± 0.0011, 0.0274 ± 0.0015, 0.0479 ± 0.0010 μmol/mg protein, respectively (Figure 5B). The cholesterol amount of Huh-7/myc-HBx cell was significantly increased compared with that of Huh-7 or Huh-7/blank cells (^*^p < 0.05). Using oil red O staining, we found that the amounts of lipid in 12, 24M *p21*^HBx/+^ HBx was significantly increased compared with their WT littermates, and the amount of lipid droplets of Huh-7/myc-HBx cell was significantly increased compared with that of control group (Figure 5C and 5F).

Both of RT-qPCR and WB showed that ADFP mRNA and PPARγ protein level were significantly increased compared with those in Huh-7 control cell (Figure 5D and 5E), which further confirmed that up-regulation of expression of PPARγ by HBx induction might activate the transcription process of ADFP. Surprisingly, we also noticed that protein levels of all of CDC42, CFL1, PPARγ and ADFP in Huh-7/myc-HBx cell were significantly increased compared with those of control group. These results were highly consistent with the phenotype of *p21*^HBx/+^ mice.

### The CDC42 and ADFP were involved in the HBx-induced the dysregulation of cytoskeletal remodeling and lipid metabolism processes

**Figure 6:**
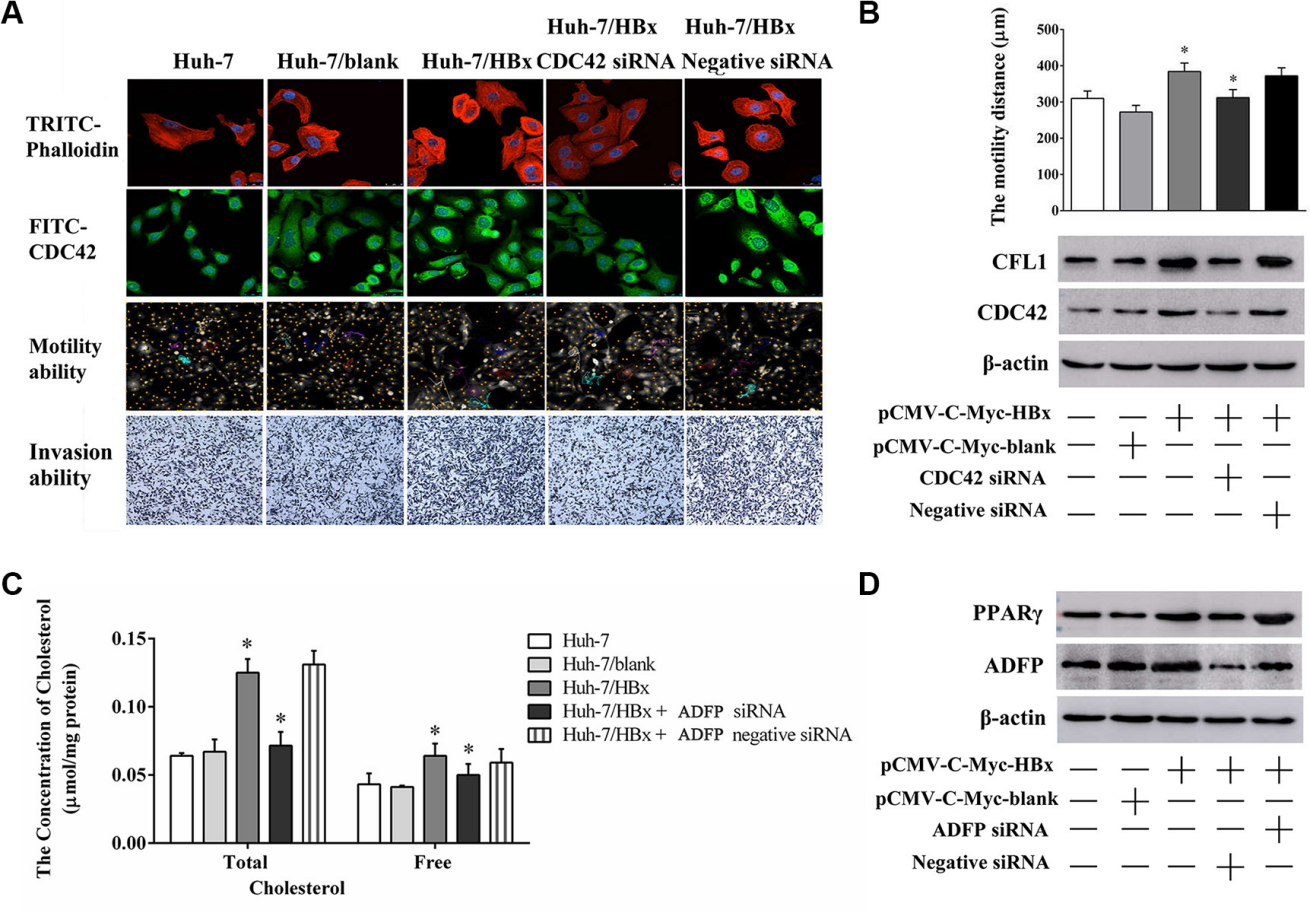
The changing of cell migration, invasion ability and lipid metabolism through knock down CDC42 or ADFP expression (**A**) The changing of F-Actin structure, CDC42 expression level, cell migration and invasion ability in Huh-7, Huh/HBx and Huh-7/HBx cells with CDC42 treatment. (**B**) Cells were transfected with pCMV-C-myc-HBx and CDC42 siRNA for 36 h, cells were collected and the lysates isolated and measured by western blot analysis using anti-CFL1 and anti-CDC42 antibodies. β-actin as a loading control. (**C**) The total and free intercellular cholesterol concentration of Huh-7 cells under different treatment. Knock down ADFP expression decreased the total and free intercellular cholesterol concentration in Huh-7/HBx cells (*p < 0.05). The total protein content as a calibration standard. (**D**) Cells were transfected with pCMV-C-myc-HBx and ADFP siRNA for 36 h, cells were collected and the lysates isolated and measured by western blot analysis using anti-PPARγ and anti-ADFP antibodies. β-actin as a loading control.

In Figure 6A, the phalloidin stains results shown that F-actin and CDC42 expression levels of Huh-7/HBx cells was significantly increase compared to those of Huh-7 and Huh-7/blank cells, however, those of the Huh-7/HBx cells with CDC42 siRNA treatment was significantly decreased compared to those of negative siRNA. Meanwhile, the mean migration distances of Huh-7, Huh-7/HBx, Huh-7/HBx cells, and Huh-7/HBx cells with CDC42 siRNA treatment were 310.21 ± 20.12, 284.32 ± 23.22 and 312.00 ± 22.17 μm. The migration and invasion ability of Huh-7/HBx cells was significantly higher than that of Huh-7, CDC42 knockdown significantly attenuated inhibited the migration and invasion ability of Huh-7/HBx cells. We also noticed that CDC42 knockdown could also attenuate the CFL1 overexpression trend of HBx induction (Figure 6B). These evidences showed that HBx promoted the dysregulation of cytoskeletal remodeling in HCC cells by activation of CDC42 signaling.

Otherwise, the total and free cholesterol concentrations of Huh-7, Huh-7/HBx cells and Huh-7/HBx cells with ADFP siRNA treatment were 0.0064 ± 0.0020, 0.1250 ± 0.009, 0.0715 ± 0.010 μmol/mg protein and 0.0432 ± 0.0080, 0.0640 ± 0.0090, 0.050 ± 0.0080 μmol/mg protein (Figure 6C). ADFP knockdown could decrease the cholesterol accumulation of overexpression HBx induction in Huh-7 cells. we also found that ADFP knockdown could decrease the PPARγ expression in Huh-7/HBx cells with ADFP siRNA treatment compared to that of Huh-7/HBx cells (Figure 6D). We regarded that CDC42, CFL1 and ADFP might promote the process of HBx-induced the inflammation and cancer in liver cells through dysregulation of cytoskeletal remodeling and lipid metabolism.

### Dysregulation of CFL1 and ADFP is conserved in HCC

**Figure 7:**
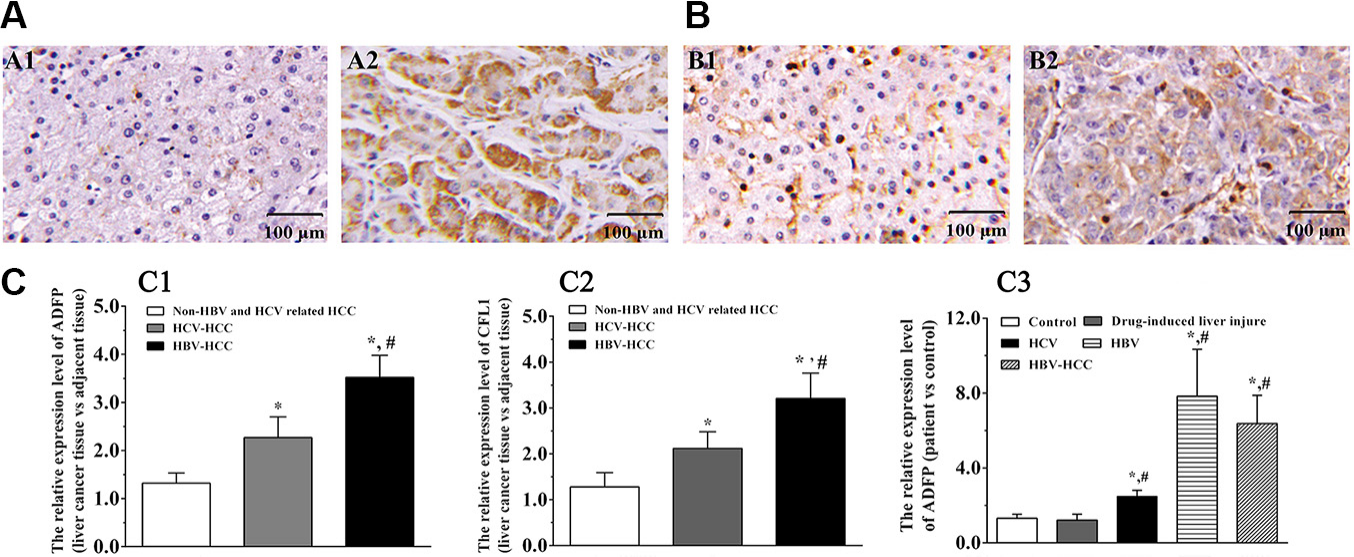
CFL1 and ADFP accumulated in human HBV-HCC liver samples (**A**–**B**) Proteins of ADFP (A1-2) and CFL1 (B1-2) in adjacent normal tissues and HBV-HCC tissues were analyzed by IHC, respectively. (**C**) Statistical analysis using Wilcoxon's signed-rank test. The ADFP and CFL1 expression levels in HBV-HCC tissues were significantly increased compared with that of control or non-HBV-HCC group, respectively (^*^p < 0.05, ^#^*p* < 0.05) (C1–2). The ADFP level in serums was significantly increased compared with control or non-HBV-HCC group (^*^p < 0.05, ^#^*p* < 0.05) (C3). The CFL1 was not detected in serums.

Among the validated proteins, CDC42 and SEPT9 had been found to be greatly increased in multiple tumor tissues, including liver, colorectal, and breast cancer and glioma [24, 25]. However, there is little information about the function of ADFP and CFL1 in the occurrence and development of HCC. We then asked whether these changes were also conserved in HCC patients. We performed IHC on ninety pairs of tissue samples from HCC patients, including 79 HBV-HCC and 11 non-HBV-HCC patients with antibodies against CFL1 and ADFP, respectively. The amount of ADFP protein in HBV-HCC patients was increased and located at cytoplasm compared with the adjacent nontumor tissue (Figure 7A). Similarly, the amount of CFL1 proteins was also increased and located at the cytoplasmic and plasma membrane (Figure 7B). The results also showed that CFL1 and ADFP were significantly increased in HBV and non-HBV-HCC compared with paired adjacent nontumor tissue (*p* < 0.01), suggesting the conservation of accumulation of these two proteins in HCC. Interestingly, we noticed that these two proteins accumulated even more dramatically in HBV-HCC patients than in the non-HBV-HCC group as well (*p* < 0.01) (Figure 7C1–C2 and Supplementary Figure S9), indicating the specificity and positive correlation of the dysregulation of these two proteins in HBV-HCC and non HBV-HCC.

We further confirmed these observations with serum samples for diagnosis purpose. The results showed that ADFP was significantly increased in serum of patients with liver disease compared with control group (*p* < 0.05) (Supplementary Figure 7C3 and Supplementary Figure S10). However, we did not notice any obvious difference between HBV and HBV-HCC groups (*p* > 0.05). Unfortunately, we did not detect CFL1 signal because of detection sensitivity for low molecular weight proteins in serum.

## DISCUSSION

HCC is a common and aggressive cancer that is positive correlation to chronic HBV infection. HBx has already been shown to be involved in process of hepatocarcinogenesis, however, the mechanisms remain obscure. To identify the direct function of HBx in liver disease, we applied protein-protein interaction analysis using co-immunoprecipitation and LC-MS with HBx-transfected H22 cells. Of 162 identified HBx-interacting proteins related to cytoskeleton, mitochondrial components, lipid metabolism, transcriptional regulation, and cell cycle, 47 had also been identified as HBx-interacting proteins in the previous study [26]. The other 115 proteins were firstly identified by this study even after stringent filtering, indicating the robust nature of our protein-protein interaction analysis. Among them, 37 of proteins belong to the largest functional group of cytoskeleton-related proteins. Interestingly, myosin phosphatase Rho interacting protein appeared in our dataset, which transports myosin phosphatase to inhibit cytoskeleton remodeling by reducing and redistributing stress fibers through activation of Rho signaling [27]. This was consistent with our SILAM data, in which the major differentially expressed proteins belong to cytoskeleton-remodeling pathway, including CDC42, ACTN2, CFL1, α-SMA, and TTN. This might be due to activation of CDC42 by HBx and Ras-related protein RAB1A, leading to the changes in expression levels of other cytoskeleton proteins [28]. We also noticed that accumulation of CFL1 was positively correlated with the progression of liver disease. The HBx interaction data suggested that CFL1 was directly binded by HBx. The activation of CDC42 and CFL1 by HBx had also been confirmed in HBx-transduced Huh-7 cells in which the malignant proliferation was induced and promoted. Meanwhile, we also further confirmed that knockdown CDC42 expression would decrease the migration and invasion abilities in Huh-7/HBx cells. In CDC42 signaling pathway, CFL1 is a terminal effector involved in cytoskeleton rearrangement and promoted actin polymerization/depolymerization. The accumulation of CFL1 might change cell morphology, motility, and junctions [29]. More importantly, we also confirmed that CFL1 was overexpressed in clinical HBV-HCC patient tissues as well. Both of these *in vivo* and *in vitro* results have demonstrated that accumulation of CFL1 induced by HBx was highly conserved and specifically related to the occurrence and development of liver disease.

In addition, we found four more cytoskeleton remodeling-related proteins accumulated in 24M *p21*^HBx/+^ samples, including SEPT9, COL14, BGN, and LGMN. SEPT9 belongs to the family of GTP-binding proteins, which enhances the formation of filamentous structures and cytoskeleton by serving as a scaffold protein to recruit other proteins to specific subcellular structures. The overexpression of SEPT9 was also reported in epithelial cells of human breast cancer under the stimulation of proliferation and invasion [30]. These results further support the hypothesis that dysregulation of cytoskeletal remodeling generates the occurrence and development of HBx-induced HCC. We also found that levels of SMAP, FKBP1A, and TTN were significantly changed in 12M *p21*^HBx/+^ mice, indicating that inflammatory reaction might be positive correlation to muscular contraction and muscle organ development. A recent study showed that the expression of SMAP was markedly upregulated in inflammation-activated hepatic stellate cells, leading to cytoskeletal remodeling and morphological change in these cells [31]. Consistent with these findings, we also found that the amount of total 15 proteins in the CDC42 signaling pathway were significantly changed in 24M *p21*^HBx/+^ mice, including CDC42 itself, RAB32, RAB24, HMOX1, IQGAP1,CFL1, TLN1, VIM, EZR, SEPT9, and MSN (Figure 3B). These results suggested that the additional cytoskeletal structures, including myofibrils, actin cytoskeleton and contractile fibers, were disordered in the HBx-induced HCC tissue.

The mitochondrion was the most important organelle in lipid metabolism. 32 proteins of HBx interactome were positive to mitochondrial function, including NDUFA5, NDUFA6, TIMM23, ATP5J2, and MTX2. Although we did not observe obvious dysregulation of mitochondrial function in inflammation stage in 12M *p21*^HBx/+^ samples, we did see multiple proteins heavily related to lipid metabolism with amount change, including ADFP, DHTKD1, AKR1C18, and CES2. Even worse, 18 of 97 differentially expressed proteins in HBx-induced 24M *p21*^HBx/+^ HCC mice were associated with mitochondrial dysfunction, including ADFP, DHTKD1, IVD, LDHD, and NDUFA5. Our results suggested that the disordered lipid metabolism and mitochondrial dysfunction might be important factors in HBx-induced HCC developed from inflammation.

ADFP (PLIN2), one of PLIN family member and well characterized protein in fatty liver disease, was increased in both 12M and 24M *p21*^HBx/+^ mice. In fact, both ADFP and PLIN1 had been identified on the surface of lipid droplet. These proteins recruit lipases or prevent the access of lipases into the lipid droplet, resulting in accumulation of lipids in the liver tissue [32]. Here, accumulation of ADFP, but not PLIN1, suggests a specific role of ADFP in HBx-induced liver disease. To further explore the molecular mechanism underlying ADFP upregulation in HBx-induced HCC in *p21*^HBx/+^ mice, we carefully analyzed the 12M and 24M quantitative MS datasets. We found that the Pex11c had increased in 12M *p21*^HBx/+^ mice. As a liver-specific protein, Pex11c stimulates the peroxisome biogenesis. The transcription of Pex11c is activated by PPARγ 42). Meanwhile, an increase in PPARγ on the promoter region of the ADFP gene could lead to the accumulation of lipids in liver tissues [33]. The transcription of ADFP was increased by the lipids, resulting in the further accumulation of lipids in liver cells [34]. The activation of PPARγ signaling and stimulation of ADFP transcription by HBx-induction were also confirmed in HBx-transduced Huh-7 cells. Except these, we also identified several other HBx interacting proteins related to lipid catabolism, including ACSF2, HADH, SCP2, PNLIP, and LCN1. Among them, ACSF2 and HADH were the rate-limiting enzymes involved in β-fatty acid oxidation, SCP2, PNLIP, and LCN1 were lipid binding proteins for intra- or inter-cellular lipid trafficking in lipid droplet [20–22]. Hence, HBx might induce the accumulation of lipids in liver tissues by reducing the amount of lipase penetrating into lipid droplet or inhibiting enzymatic activity. Zhang *et al*. also showed that HBx hampered efflux of cellular lipids through an interaction with apoA-I [26]. The accumulation of lipids in liver tissues would further aggravate an inflammatory reaction and eventually lead to HCC. We observed obvious accumulation of lipids in liver tissues of *p21*^HBx/+^ mice compared with their WT littermates, further demonstrating the relationship between the accumulation of lipids and HBx induction. Meanwhile, we also further confirmed that knockdown ADFP expression would attenuate the HBx-induced abnormal lipid metabolism in process Huh-7/HBx cells. In this study, HBx-induced lipid metabolism disorder network was successfully constructed to benefit the further understanding of the mechanism of HBV-induced liver steatosis in future.

In brief, liver inflammation and cancer caused by HBx-induced activation of CDC42 and PPARγ, and followed by disruption of cytoskeleton and lipid metabolism. More importantly, we found that ADFP was also specifically accumulated in serum of HBV, HBV-HCC and HCV patients. We will further appraise the consistency of the accumulation of ADFP in HBV and HCV patients by increasing the number of HCV samples in the future study.

## MATERIALS AND METHODS

### Reagents

Lys6-SILAC Mouse Diet was from Silantes company, and molecular weight of heavy labeled lysine was larger +6.0201 Da than light lysine in normal mouse diet (Germany). Rabbit monoclonal anti-CDC42(ab187643), anti-CFL1 (ab124979), anti-ADFP (ab108323), anti-SEPT9 (ab38314) and anti-PPARγ (ab19481) were from Abcam; Rabbit monoclonal anti-FLAG (F7425) and Oil Red O staining kits (MAK194) was from Sigma. Endoproteinase Lys-C was from Wako (Osaka, Japan) and trypsin was from Promega (Madison, WI). Matrigel was from BD Biosciences (356234) and Transwell chamber was from Corning company (3460). CDC42 siRNA (h) (sc-29256) and control siRNA (sc-37007) was from SantaCruz Biotechnology (Santa Cruz, CA) and ADFP siRNA (SR300083) was from Origene (Origene, MD). The Lipofectamine and FuGENE HD transfection reagent were from Invitrogen and Roche. The total and free cholesterol quantification kits (CY81058 and CY81061) were from ChaoYan Biotechnology (ChaoYan, China).

### Cell culture, mice, liver tissue and serum samples

Huh-7, HEK293T and H22 cells were cultured in H-DMEM containing 10% FBS at a 37°C in a humidified 5% CO_2_ atmosphere and were mycoplasma-free, respectively. One female, 6 month old F2 generation SILAM was generated as described previously [35–37]. Two matching female wild-type mice (C57Bl/6) were acquired from Laboratory Animal Center, Academy of Military Medical Sciences. The p21-HBx heterozygous (*p21*^HBx/+^) mice and their C57BL/6 WT littermates were collected at the age of 12 and 24 months, when the transgenic mice (27/76, 35.53 %, and 32/70, 45.71%) respectively developed mild inflammation and hepatocellular carcinoma. The liver tumor and peritumor tissues were obtained from 90 HCC patients without any treatment of chemo/radiotherapy before surgery at Tianjin Third Center Hospital (Supplementary Table S9). Serum samples were taken from patients with liver disease and volunteers with no known history of liver diseases (Supplementary Table S10). All HBV and HBV-HCC patients belonged to the Adrq+ serum type and C genotype (*n* = 90), and HCV patients belonged to the serum type II and genotype I (*n* = 24), which represented the main types of liver inflammation in East Asia population. non-HBV and HCV related the HCC patients were as the positive control (*n* = 21). All participants gave written informed consent for their participation. All liver samples in this study were examined by senior pathologists, and diagnoses were confirmed by immunohistochemistry or HE staining method (Supplementary Figure S11–S13). The application of *p21*^HBx/+^ heterozygous mice model is to avoid the disruption of p21-null at the same time. All the mouse liver tissues were dissected out, flushed with ice-cold PBS and immediately stored at −80°C. “*Guide for the Care and Use of Laboratory Animals*” (NIH) was followed in this study.

### The preparation of large scale MS samples

For labeling efficiency test, the liver tissue homogenates were harvested with lysis buffer (4% SDS, 100 mM Tris-HCl, 100 mM DTT and protein inhibitor cocktail) [38], followed by sonication and centrifugation and the supernatants were collected. The protein concentration in the total cell lysate (TCL) was balanced by Bradford assay and short SDS-PAGE stained with Coomassie Blue G-250. ~4 μg mouse liver SILAM sample was run on a short SDS-PAGE gel and then in-gel digested with Lys-C under the manufacture's guidance for 12 hours. Peptides were extracted three times with extraction buffer (5% formic acid and 50% acetonitrile) followed by the extraction with acetonitrile. The pooled extraction solution for each sample was dried with vacuum centrifugation for further LC-MS analysis.

At each stage, we pooled liver tissues (250 μg) from five mice with the same genotype together, including 12M and 24M *p21*^HBx/+^ transgenic mice, which was served as one sample for comparison. In addition, *p21*^HBx/+^ mice and their WT littermates were compared to avoid genetic background difference and lifespan issues. The liver tissue homogenates were lysed with the same lysis buffer (4% SDS, 100 mM Tris-HCl, 100 mM DTT and protein inhibitor cocktail) and followed by sonication and centrifugation. The supernatants were collected and the protein concentration in the TCL was determined by both of Bradford assay and short SDS-PAGE stained with Coomassie Blue G-250 as mentioned before. Equal amount of lysates from each tissue sample (250 μg) and heavy stable isotope amino acid labeled mouse (250 μg) were mixed and loaded into the each lane of 10 % SDS-PAGE gel. After electrophoresis, the gel was stained by Coomassie Blue G-250. Each lane was cut into 34 gel bands on the basis of the molecular weight and local protein amount, and then were sliced into small pieces. The gel was destained and in-gel digested overnight with 12.5 ng/μL of Lys-C as described previously. Peptides were extracted three times with extraction buffer (5% formic acid, 50% acetonitrile) followed by the extraction with acetonitrile. Meanwhile, we also performed the independent biological repeat experiment by 2D-LC separation strategy using the same pool samples strategy. From each group mixture, 500 μg of peptides were separated into 60 fractions by strong anion exchange as described previously [39]. All samples were dried using the vacuum centrifugation and stored at −80°C.

A mixture of heavy stable isotope amino acid labeled mouse liver TCLs was spiked in as a heavy-labeled internal reference, and yielded in total six experiment pairs: 12M *p21*^HBx/+^ mice/SILAM, 12M WT mice/SILAM, 24M *p21*^HBx/+^ mice/SILAM, 24M WT mice/SILAM, C57BL/6_1/SILAM, and C57BL/6_2/SILAM.

### LC-MS/MS experiments and data analysis

LC-MS/MS experiments were performed as described previously [39]. In brief, each peptide mixture was separated and analyzed by UPLC (nano AcquityUltra Performance LC, Waters)-MS/MS (LTQ OrbitrapVelos, Thermo Fisher Scientific) platform. Survey scans were operated in the orbitrap analyzer with the 30,000 resolution for target values of 1,000,000 ions in 300–1,600 m/z mass range. The 20 most intense ions were selected for fragmentation through collision induced dissociation (CID) in the LTQ. The 5,000 ions were accumulated over 25 ms as a maximum permitted filling time for each scan. The dynamic exclusion was set for 30 s to reduce the repeated fragmentation for precursor ions.

The MS/MS spectra from the *Mus musculusi* samples were searched by the MaxqQuant (version 1.5.3.8 build) search engine against a decoy database/composite target to appraise FDR [40]. The target proteins were derived from the Swiss-Prot mus musculus reference protein database (release 2015_08, 16717 query number) and the decoy proteins were produced from pseudo-reversed sequences of the target proteins. Search parameters: precursor ions were searched with an initial mass tolerance of 20 ppm. Only *b* and *y* ions were considered during the database search. Enzyme specificity was semi-LysC with two missed cleavages allowed. Up to two missed cleavages were allowed and peptides with at least 6 amino acids. The dynamic modifications for methionine oxidation (+ 15.99492) and heavy labeled lysine (+ 6.0201 Da) for SILAC samples, and the static modification for cysteine carbamidomethylation (57.021465) were allowed. The FDR of peptides and proteins less than 1% was accepted after appraising based on the number of accepted decoy hits.

For quantitative comparison between Heavy- and Light-labeling samples, we adopted SILAC quantification strategy with a minimum of two (H/L) ratio counts to ensure the protein intensity normalization [41, 42]. For quantitative comparison between *p21*^HBx/+^ mice and their WT littermate mice, we adopted the spike-in standard strategy [43–44].

### Bioinformatics analysis of differentially expressed proteins

The DAVID v6.7 was applied to analyze the biological enrichment within the protein list for the identified differentially expressed proteins [45]. The sets of 21 and 276 protein lists were performed by DAVID analysis. *Mus musculus* was selected as the background and species. Classified annotation was provided into the form of Gene Ontology (GO) and KEGG pathway. The threshold of hypothesis testing was 0.05 using the Benjamini-Hochberg FDR parameter.

### The profiling of global HBx interactome network

The HBx gene was knocked in the lentiviral transfer plasmid between XhoI and BamHI sites of pWPXLD-3FLAG and was cotransfected with transfer plasmid and helper plasmids (psPAX2 and pMD.G.2G) in HEK293T cells. The medium was collected after transfection for 36h and concentrated by ultracentrifugation, and then lentiviral vectors were applied to infect H22 cells. Cells were scraped in lysis buffer (50 mM Tris, 50 mM NaCl, 1 mM EDTA, 1% NP-40, 5% Glycerol). The supernatants were collected and incubated with monoclonal anti-FLAG antibody (Sigma) immobilized on Protein A/G-agarose beads for 2 h at 4°C. According to previous description, the seven bands from the lanes of HBx-3FLAG and 3FLAG were excised and in-gel digested overnight with 12.5 ng/μL of trypsin at 37°C. The samples were performed to mass spectrometry analysis as described previously.

### Immunohistochemistry (IHC) and western blot (WB)

The liver tissue specimens of mice and human were fixed with 4%(v/v) paraformaldehyde. The sections were deparaffinized and rehydrated. Nonspecific bindings were blocked with 10% goat serum. Sections were then incubated with primary antibodies overnight at 4°C. The hybridized sections were washed in PBS for 3 times followed by incubation with secondary antibody for 30 min. The signal was detected using a 3, 3**′**-diaminobenzidine (DAB) staining. Four representative horizons were captured by Olympus BX40 microscopy. The 10 digital images at 200 × magnification were captured in each section using the Olympus CX-31 microscope (Olympus). We analyzed the cases with equivocal, weak, and strong expression by IPP (version 6.0, Silver Spring, MD), using the method introduced by Xavier et al. Briefly, we assessed the protein expression levels by mean optical density (MOD) value, it was calculated by the ratio of integrated integral optical density (IOD) and Sum Area.

For serum proteins enrichment according to previous description, briefly, 10 μL serums samples were mixed with 20 μL dissolution buffer (8M urea, 20 mM DTT) and incubated for 30 min, and then added into IAA at 50 mM final concentration for 30 min, used ddH_2_O make up to 100 μL. The 1.5 mL cool acetone was added the this solution and incubated for 4 h at −20°C. Centrifugation was performed at 19,000g for 15 min at 4°C. The precipitation were incubated with dissolved buffer (70% acetonitrile, 12 mM HCl, pH 3.0). The supernatants were collected by centrifugation at 19,000 g for 15 min at 4°C and dried using vacuum centrifugation, and then the dried samples were dissolved with 100 μL ddH_2_O. For Western blot, 60 μg of protein from each individual sample was separated by SDS-PAGE and transferred onto PVDF membrane. The membrane was blocked in 5% nonfat milk in PBST for 1 h, incubated with primary antibody (1:1000 in 5% nonfat milk in PBST) for 1 h, and washed three times in PBST. The secondary antibody (1:10000 in 5% nonfat milk in PBST) was added for 1 h incubation. GAPDH was served as a loading control. The *Bandscan* 5.0 software was applied to measure the intensity.

### The analysis of HBx-induced cell proliferation activity using WST1

Cells were seeded in 96-well plates in Phenol Red-free H-DMEM medium, supplemented with 100 units/mL penicillin. The next day, treatment was delivered in Phenol Red-free H-DMEM medium to the existing medium in the well, resulting in a final serum concentration of 10% (v/v). The HBx gene was cloned into the pCMV-C-Myc plasmid between BamHI and XhoI sites, and the Huh-7 cells was transfected with pCMV-C-Myc-HBx (Huh-7/myc-HBx) or a pCMV-C-Myc blank vector (Huh-7/blank) using FuGene HD reagents according to the manufacturer's. After 24, 48 and 72 h, the WST assay (Roche Diagnostics) was operated according to the manufacturer's instructions. The absorbance value at 490 nm was detected using an ELISA microplate reader.

### Quantitation of adipose droplet and cholesterol by staining intracytoplasmic lipids with oil Red O and CHOD-PAP method

The Huh-7, Huh-7/blank and Huh-7/myc-HBx cells were seeded in six-well plates at a density of 2 × 10^4^ /mL in H-DMEM medium. After the cells grew to 80–90% confluence, cells were incubated with 0.5% oil red O in propylene glycol for 5 min, and then differentiated in 85% propylene glycol solution for 5 minutes. The frozen specimens of liver tissues of 12M and 24M p21^HBx/+^ mice and their WT littermates also were followed by above operation. The images at 200 × magnification were captured using the Nikon Ti-E microscope (Nikon), the density of adipose droplet was measured by Image-Pro Plus 6.0 software (Media Cybernetics, Inc., Bethesda, MD). The 1 × 10^6^ /mL cells were scraped in 300 μL PBS and crushed using the sonicator, the total and free cholesterol concentrations were measured by cholesterol oxidase p-aminophenazone (CHOD-PAP) kit (Beyotime, China), the absorbance value of reaction product at 570 nm was detected using an ELISA microplate reader. and the corresponding protein concentration as a calibration standard was detected using BCA method.

### Validation of quantitative MS result with RT-qPCR and western blot

The ADFP mRNA level was measured by RT-qPCR analysis. The density of 5 × 10^5^ cells/dish of Huh-7, Huh-7/blank, Huh-7/myc-HBx cells were seeded into culture dish. After 24 h, total RNA was isolated using the TRIzol reagent (Invitrogen, CA) according to the manufacturer's protocol. cDNA samples were synthesized by a PrimeScript^®^Strand cDNA Synthesis Kit (TaKaRa Shiga, Japan). GAPDH RNA level was as a semi-quantitative internal reference in real-time PCR analysis. The Real-time PCR was operated in a 25 μL reaction system with SYBR Green PCR Master Mix (Applied Biosystems, CA). Fluorescence was detected on an ABI StepOnePlus system. Primers for real-time PCR were: ADFP, 5′-TTG CAG TTG CCA ATA CCT ATG C-3′ (forward) and 5′-CCA GTC ACA GTA GTC GTC ACA -3′ (reverse), and GAPDH, 5′- GAC TTC AAC AGC AAC TCC -3′ (forward) and 5′- TAG CCA TAT TCA TTG TCA TAC C -3′ (reverse), respectively. Sample mRNAs were normalized to the respective GAPDH expression levels. The results were presented as fold induction. The protein amount of HBx, CDC42, CFL1, PPARγ and ADFP was measured and analyzed as described previously in Western blot part.

### Knock down CDC42 and ADFP, cell migration, invasion ability and lipid metabolism

The Huh-7 cells were cultured in 6-well plates at a density of 3 × 10^4^ cells per well and respectively co-transfected with pCMV/HBx vector and CDC42 siRNA, ADFP siRNA or r or a negative control siRNA using FuGene HD reagents. After 36 h of transfection, cells was applied for western blot analysis according previous description. A total of 1 × 10^4^ Cells was seeded onto the confocal plate (Nest, 801002) and treated according to previous description. cells were fixed in 4% pre-cooling paraformaldehyde for 10 min and then permeabilized with 0.5% Triton X-100 for 20 min at 37°C. cells were incubated with the TRITC Phalloidin labeling (1:500) (Yeasen, China) at 37°C for 30 min and followed by incubated with the 1 μg/ml DAPI for 5 minutes. cells were labeled with anti-CDC42 primary antibody (1:200) (Abcam, USA) and followed by incubated with DyLightTM^488^ secondary antibody for 1 hour at 37°C as well as dyed in 1 μg/ml DAPI for 5 min. Cells were visualized using confocal microscopy (Leica TCS SP8). The images were analyzed using IPP 6.0 software.

The migration ability of cells was assessed using holographic time-lapse imaging cytometer HoloMonitor M4 (Phase Holographic Imaging, Lund, Sweden). The cells treatment accorded to the previous description [46]. After 36 h of transfection, the HoloMonitor M4 system was used to measure the migration distance of cells. Images were captured once every 5 min and lasted 24 h, these data was analyzed the mean migration distance per cell by Holostudio 2.4 software (Phase Holographic Imaging, Sweden) . Each line represented migration distance of a single cell. The transwell assay was performed as described [47]. Briefly, invasion assay was proceeded using self-coating Matrigel (BD Biosciences, MD) on the upper surface of the chamber. The invaded cells at the lower surface of the chamber were fixed with 75% ethanol and stained with hematoxylin. 3 randomly selected eyesights of the fixed cells were captured and calculated the cell amount. The concentration of cholesterol was measured using CHOD-PAP method as previous description.

### Statistical analysis

Data are described as mean ± standard error (SEM). The comparisons between groups are carried out using one-way ANOVA with SPSS 10.0 software. Otherwise, the protein expression levels in tissues and serums samples were analyzed using Wilcoxon's signed-rank test. *p* value < 0.05 is considered statistically significant.

## SUPPLEMENTARY MATERIALS





















## Abbreviations

HBx: hepatitis B virus X-protein
HCC: hepatocellular carcinoma
SILAM: stable isotope labeling in mammals
HBV-HCC: HBV-associated HCC
HBV: hepatitis B virus
IHC: Immunohistochemistry
WB: western blot
CDC42: cell division cycle 42
CFL1: cofilin-1
PPARγ: peroxisome proliferator-activated receptor gamma
ADFP: adipose differentiation-related protein
SEPT9: septin-9
CHOD-PAP: cholesterol oxidase p-aminophenazone
GO: Gene Ontology
FDR: false discovery rate.
