# Supplementary material for: Hepatitis B virus X induces inflammation and cancer in mice liver through dysregulation of cytoskeletal remodeling and lipid metabolism
Source: Oncotarget. 2016 Sep 30;7(43):70559–74. doi: 10.18632/oncotarget.12372 (PMC5342574; doi:10.18632/oncotarget.12372)
Supplement: Supplementary file 1 [file oncotarget-07-70559-s001.pdf]

Hepatitis B virus X induces inflammation and cancer in mice liver through dysregulation of cytoskeletal remodeling and lipid metabolism

Supplementary Materials

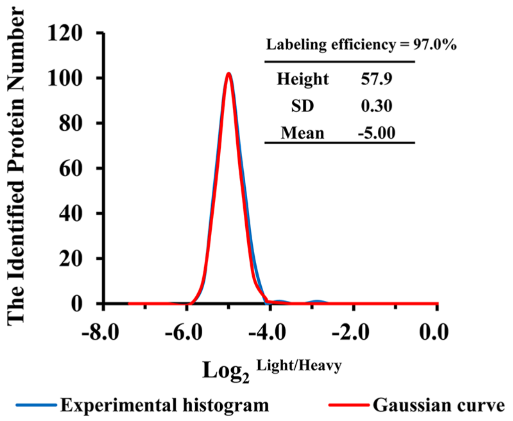

Supplementary Figure S1: Labeling efficiency of SILAM samples. Histogram of the population of proteins quantified by the log-transformed ratio of heavy versus light peptides representing protein labeling efficiency.

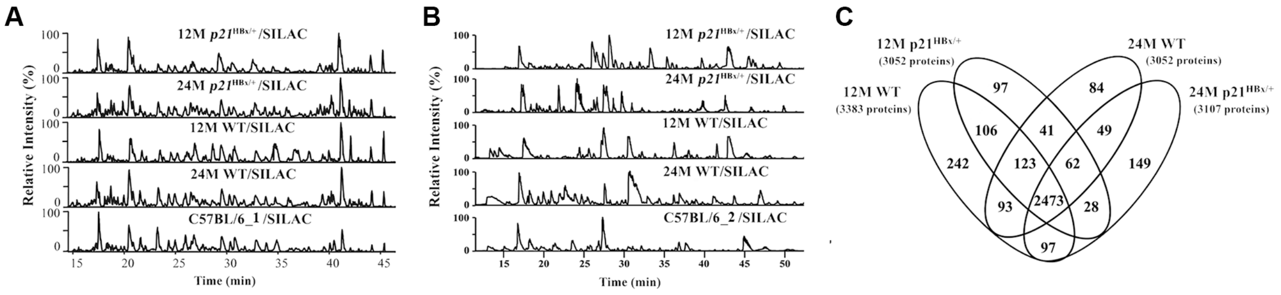

Supplementary Figure S2: Chromatograph and identification of quantitative proteomics study. (**A**) Chromatograms from five representative LC-MS runs using gel separation strategy. (**B**) Chromatograms from five representative LC-MS runs using 2D LC separation strategy. (**C**) Overlap comparison of all identified proteins in SILAM study. The number of total identified proteins (3893) and overlap among these proteomics datasets (2473).

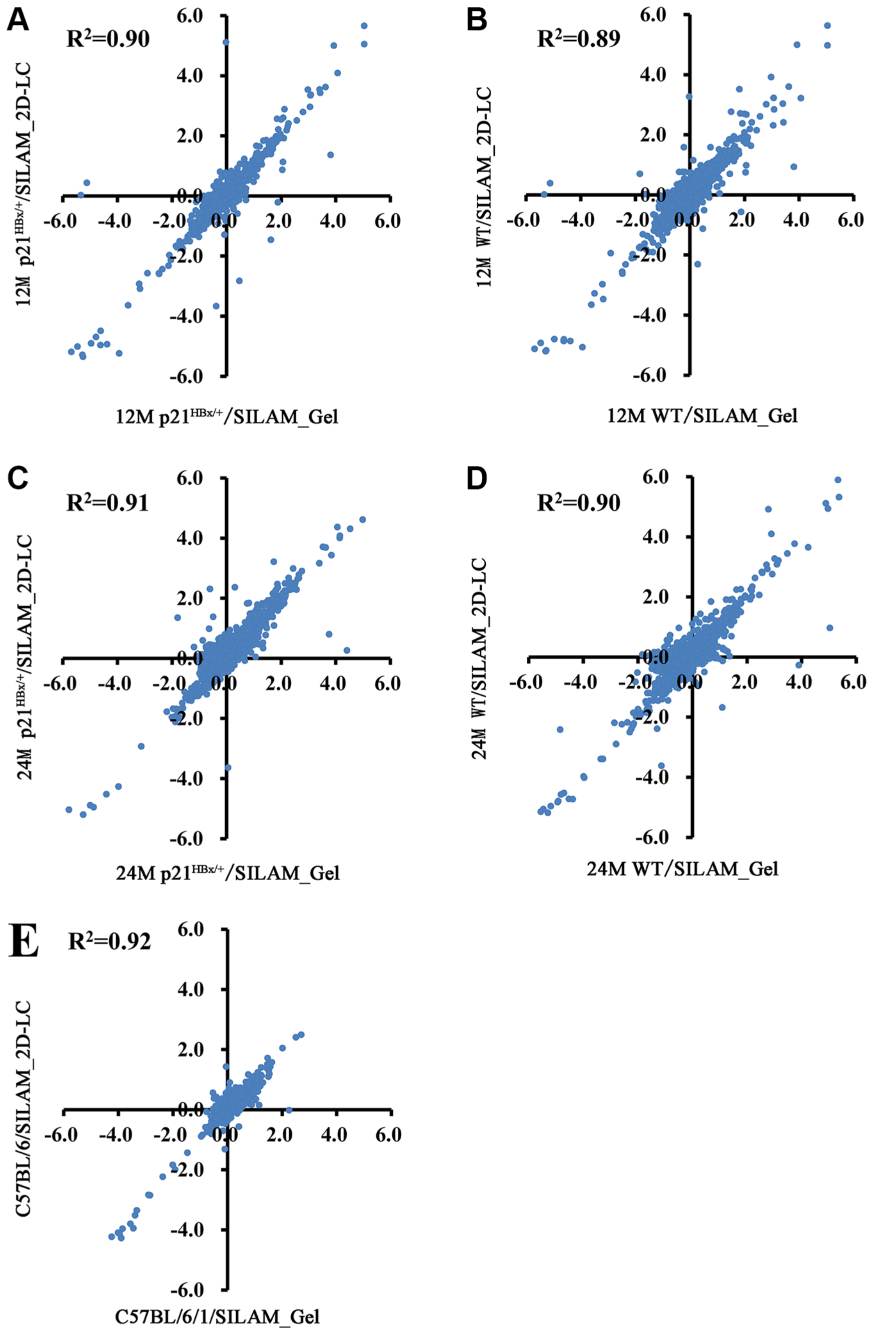

Supplementary Figure S3: The correlation coefficient (R^2) of quantitative data in independent biological repeat. (**A**) The correlation analysis of quantitative data between SDS-PAGE (n = 3303) and 2D-LC strategy (n = 3953) in the 12M *p21*$^{HBx/+}$ /SILAC, overlapping qualified protein number was 2854, R^2 = 0.90. (**B**) The correlation analysis of quantitative data between SDS-PAGE (n = 2853) and 2D-LC strategy (n = 4726) in the 12M WT/SILAC, overlapping qualified protein number was 2728, R^2 = 0.89. (**C**) The correlation analysis of quantitative data between SDS-PAGE (n = 3248) and 2D-LC strategy (n = 5226) in the 24M *p21*$^{HBx/+}$ /SILAC, overlapping qualified protein number was 2999, R^2 = 0.91. (**D**) The correlation analysis of quantitative data between SDS-PAGE (n = 3187) and 2D-LC strategy (n = 4433) in the 12M WT/SILAC, overlapping qualified protein number was 2899, R^2 = 0.90. (**E**) The correlation analysis of quantitative data between SDS-PAGE (n = 2784) and 2D-LC strategy (n = 4856) in the C57BL/6 /SILAC, overlapping qualified protein number was 2669, R^2 = 0.92.

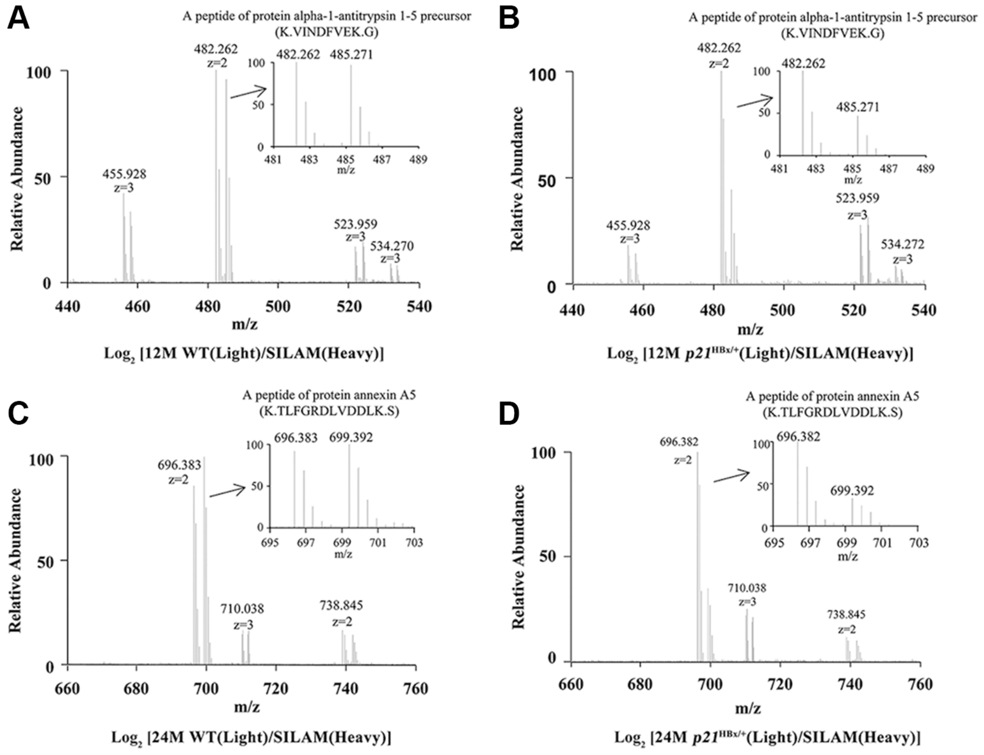

Supplementary Figure S4: Protein profiling of $p21^{HBx/+}$ mice and littermates by the SILAM approach. (**A**, **B**) Representative isotope-labeled peptide pairs in the tissue lysates from 12M mice and SILAM labeled internal standard mixed at 1:1 ratio. The light- and heavy-labeled peptides from the HBx related mice and SILAM labeled internal standard were distinguishable by different m/z. SILAM analysis of a randomly selected peptide precursor ion (VINDFVEK) from the protein alpha-1-antitrypsin 1-5 precursor on the 12M $p21^{HBx/+}$ and 12M WT mice. MS spectra and monoisotopic m/z values of the detected peptide from these two samples are shown. (**C**, **D**) The same as in panel (A and B) except the TCLs from the 24M mice and SILAM labeled internal standard. Another peptide pair (TLFGRDLVDDLK) from the protein Annexin A5 on the pairs of 24M $p21^{HBx/+}$ and 24M WT samples was showing similar trend as in (A and B).

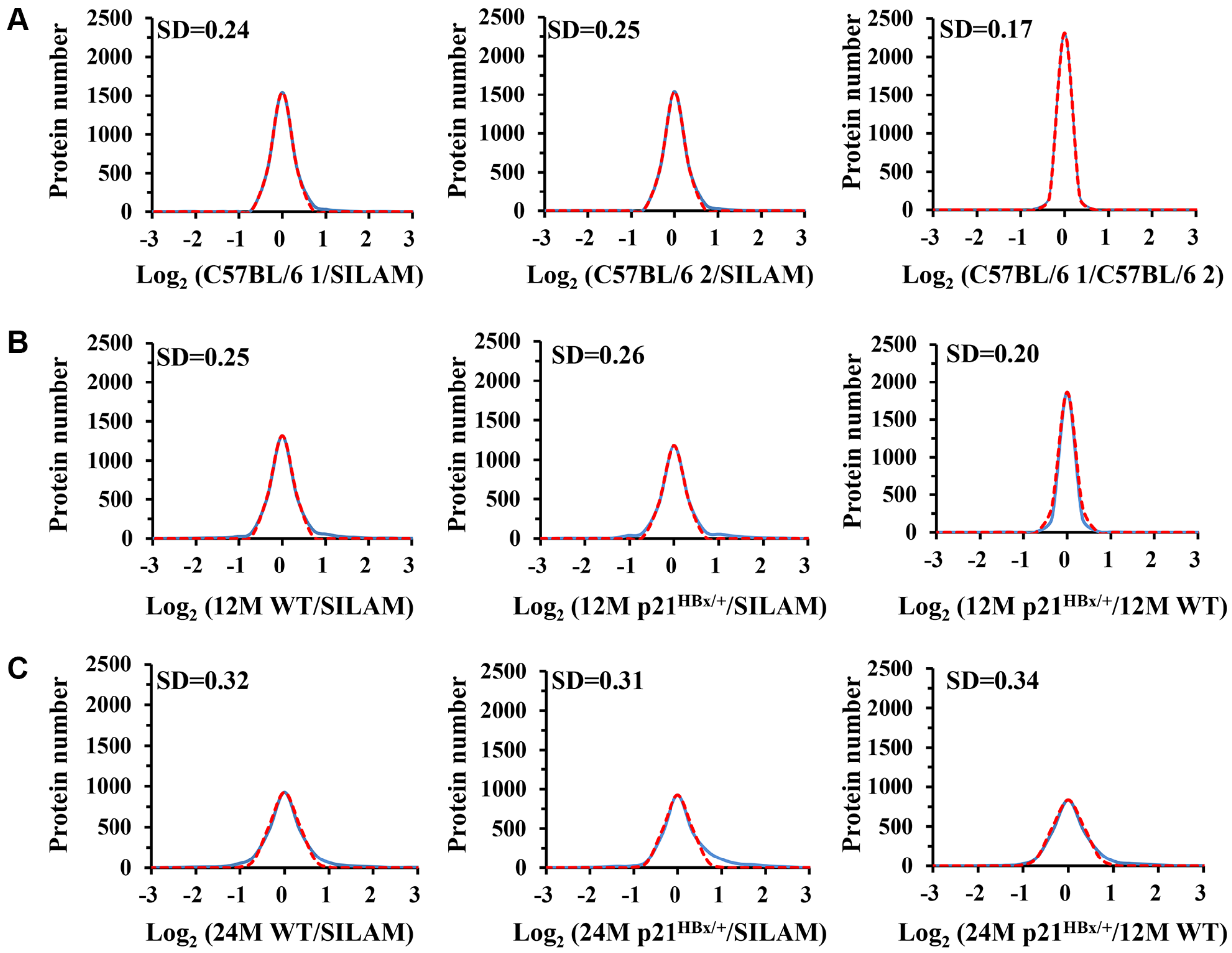

Supplementary Figure S5: Histograms of log2 ratios of quantified proteins in comparisons. (A1–3): Histograms of log2 ratios of quantified proteins in experimental comparisons, C57BL/6 1 vs the SILAM (n = 2784) (A1), C57BL/6 2 vs the SILAM (n = 4856) (A2), and C57BL/6 1 vs C57BL/6 2 (n = 2669) (A3). Corresponding SD values of these histograms were 0.24, 0.25 and 0.17, respectively. (B1-3): Histograms of log2 ratio of quantified proteins in experimental comparisons, 12M WT vs the SILAM (n = 2728) (B1), 12M $p21^{HBx/+}$ vs the SILAM (n = 2854) (B2), and 12M $p21^{HBx/+}$ mice vs their littermates (n = 2445) (B3). Corresponding SD values of these histograms were 0.25, 0.26 and 0.20, respectively. (C1-3): Histograms of log2 ratios of quantified proteins in experimental comparisons, 24M WT vs the SILAM (n = 2899) (C1), 24M $p21^{HBx/+}$ vs the SILAM (n = 2999) (C2), and 24M $p21^{HBx/+}$ mice vs their littermates (n = 2607) (C3). Corresponding SD values of these histograms were 0.32, 0.31 and 0.34, respectively.

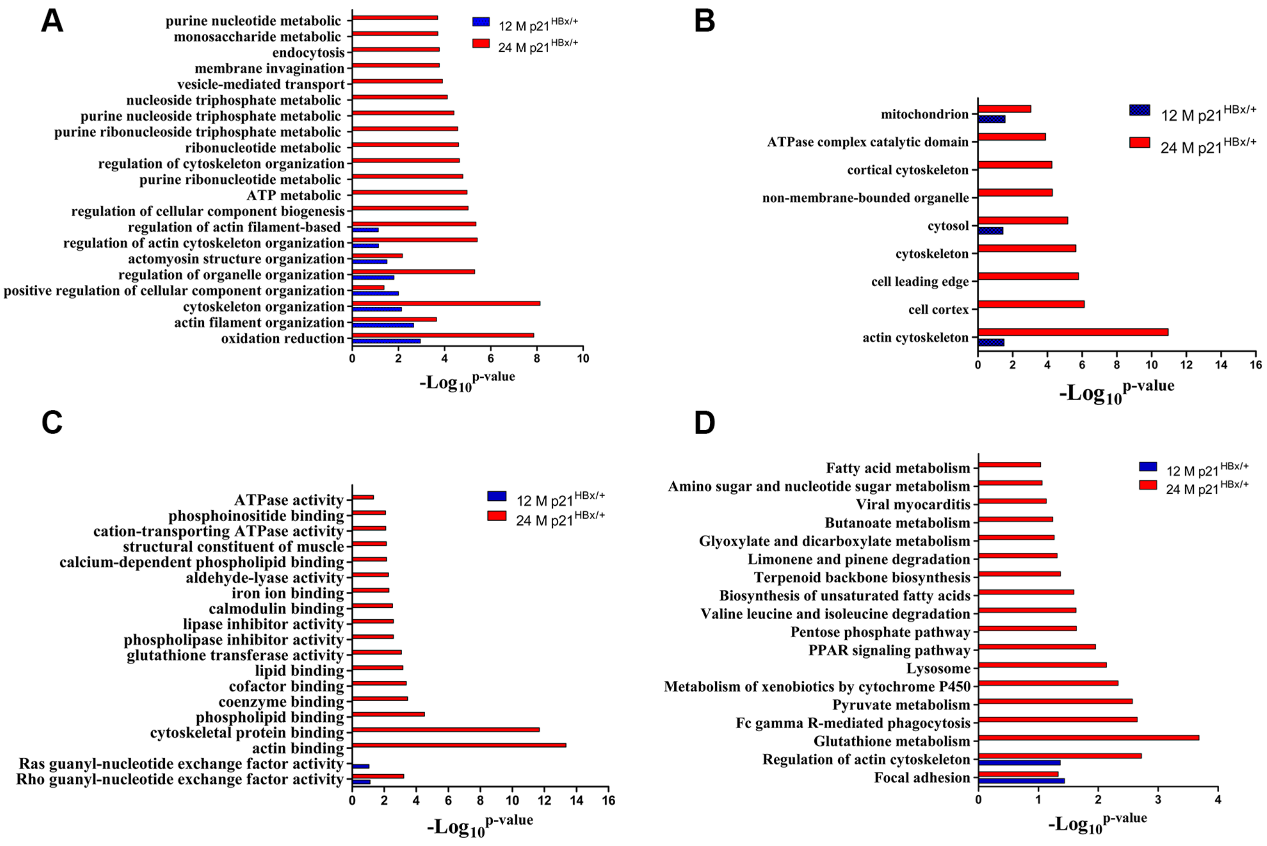

Supplementary Figure S6: Gene Ontology categories of Molecular Function enrichment and KEGG pathway enrichment of amount significantly altered proteins after the transfection of HBx gene. (A) Biological process. (B) Cellular component. (C) Molecular function. (D) KEGG pathway.

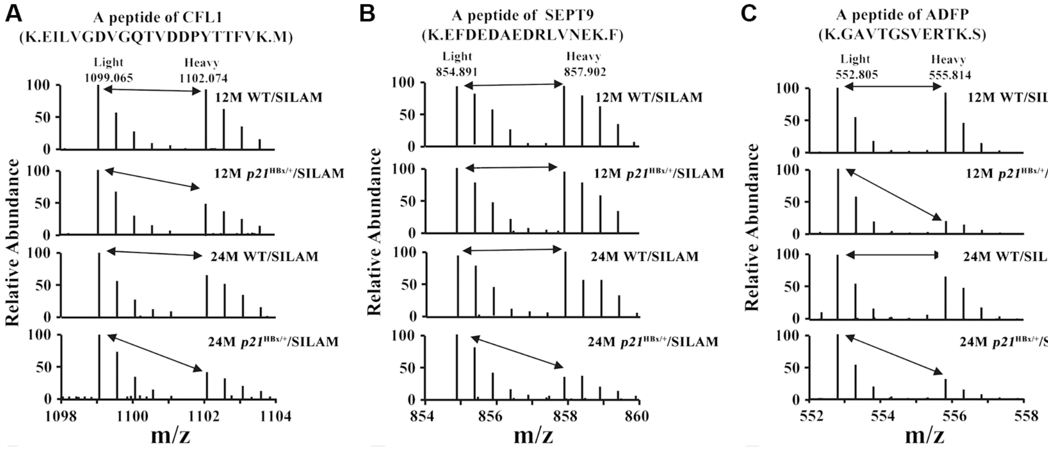

Supplementary Figure S7: Validation of CFL1, SEPT9 and ADFP with significant amount change selected from SILAM based quantitative proteomics. (**A**) A randomly selected precursor ion of peptide (EILVGDVDQTVDDPYTTFVK) from the protein CFL1 on the 12M, 24M *p21*$^{HBx/+}$ and their littermates. MS spectra and monoisotopic m/z values of the detected peptide from these two samples are shown. The relative abundance of the peptide in both groups of *p21*$^{HBx/+}$ mice was approximately 2-fold higher than that in their littermates. (**B**) A randomly selected precursor ion of peptide (EFDEDAEDRLVNEK) from the protein SEPT9 on the 12M, 24M *p21*$^{HBx/+}$ and their littermates. MS spectra and monoisotopic m/z values of the detected peptide from these two samples are shown. The relative abundance of the peptide in 24M *p21*$^{HBx/+}$ mice was approximately 2-fold higher than that in their littermates, however, that in 12M *p21*$^{HBx/+}$ mice was obviously changed. (**C**) A randomly selected precursor ion of peptide (GAVTGSVERTKS) from the protein ADFP on the 12M, 24M *p21*$^{HBx/+}$ and their littermates. MS spectra and monoisotopic m/z values of the detected peptide from these two samples are shown. The relative abundance of the peptide in both groups of *p21*$^{HBx/+}$ mice was approximately 3-fold higher than that in their littermates.

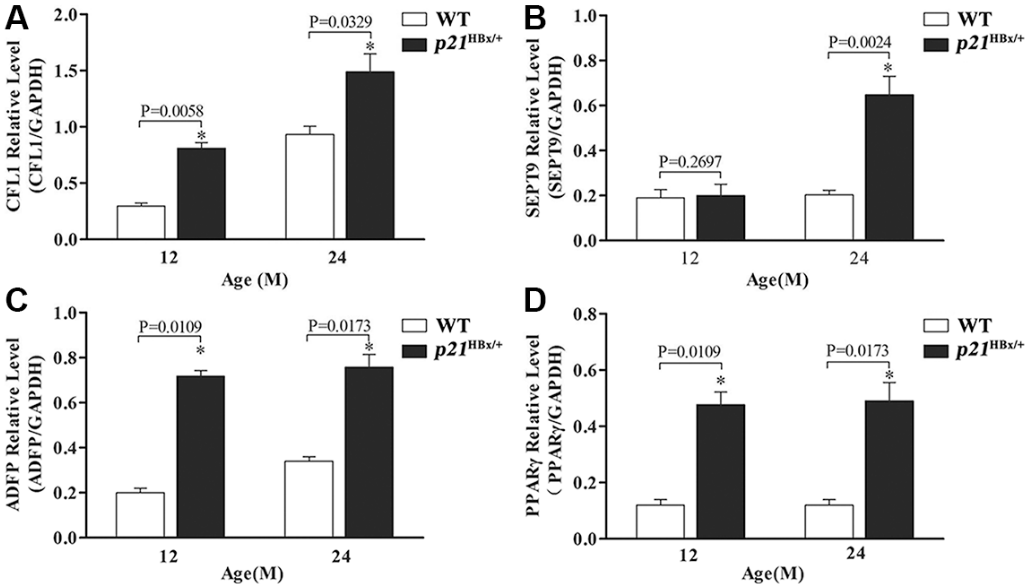

Supplementary Figure S8: Validation of the amount change of CFL1, SEPT9, ADFP and PPARγ in the 12M and 24M of p21$^{HBx/+}$ transgenic mice by WB. Levels of CFL1 (**A**), SEPT9 (**B**), ADFP (**C**), and PPARγ (**D**) in 12M and 24M *p21*$^{HBx/+}$ mice and their wild-type (WT) littermates were detected by WB method. GAPDH was used as a loading control in WB. Histograms represent a densitometry measurement of specific bands using the corresponding GAPDH level as a control. The asterisk represents the significant difference from control, $p < 0.05$.

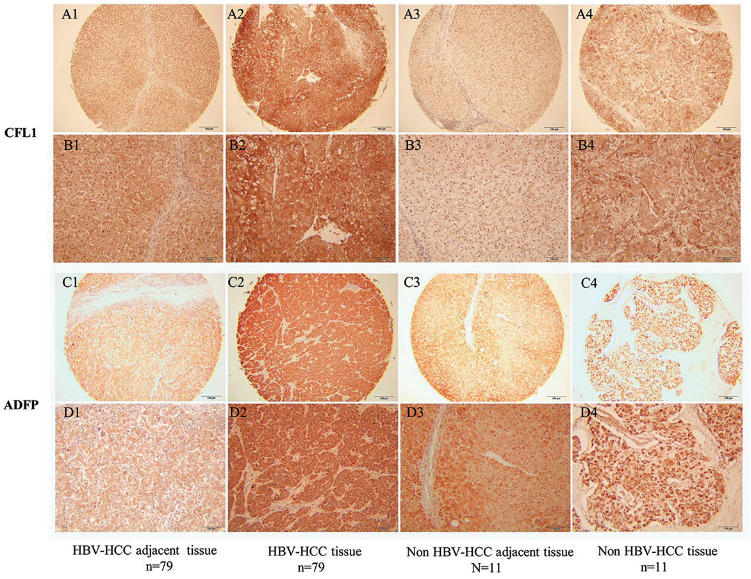

Supplementary Figure S9: Validation of the amount change of proteins CFL1 and ADFP affected by HBx with the clinical liver tissue samples by IHC. (**A** and **C**, **B** and **D**) The protein levels were detected by IHC analysis (original magnification100× and 200×). (A1 and B1, A2 and B2) The protein level of CFL1 in HBV-HCC (A2, B2) was increased compared with its adjacent paired normal tissue (A1, B1) (n = 79). (A3 and B3, A4 and B4) The protein level of CFL1 in Non HBV-HCC (A4, B4) was increased compared with its adjacent paired normal tissue (A3, B3) (n = 11). However, the protein level of CFL1 in HBV-HCC was increased compared with that in Non HBV-HCC. (C1 and D1, C2 and D2) The protein level of ADFP in HBV-HCC (C2, D2) was increased compared with its adjacent paired normal tissue (C1, D1) (n = 79). (C3 and D3, C4 and D4) The protein level of ADFP in Non HBV-HCC (C4, D4) was increased compared with its adjacent paired normal tissue (C3, D3) (n = 11). The protein level of ADFP in HBV-HCC was increased compared with that in Non HBV-HCC.

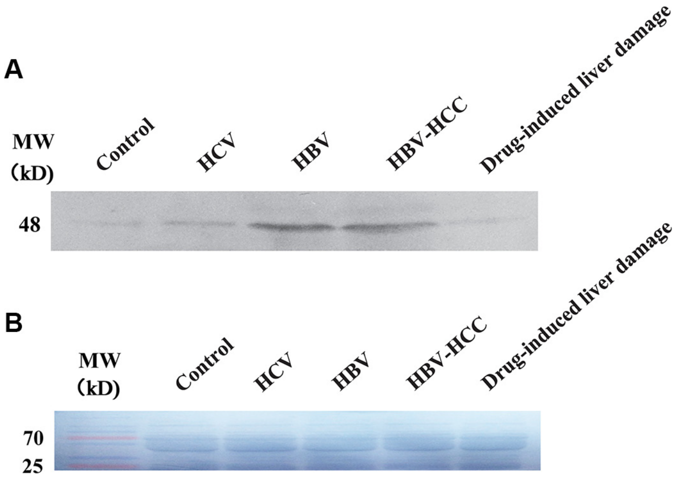

Supplementary Figure S10: Validation of the amount change of ADFP with the serum samples of the normal, HCV, HBV, HBV-HCC and drugs-induced liver damage patients by Western Blot. (**A**) The ADFP expression level was detected in the serum samples using WB method. The result showed that the ADFP expression levels in HBV and HBV-HCC samples were significantly increased compared with that in the normal, HCV and drug-induced liver damage group. (**B**) The nitrocellulose membrane transferred with serum samples was stained by 0.25 % alkaline-black solution. It showed that the amount of loading for each sample was almost equal to ensure the reliability of the results.

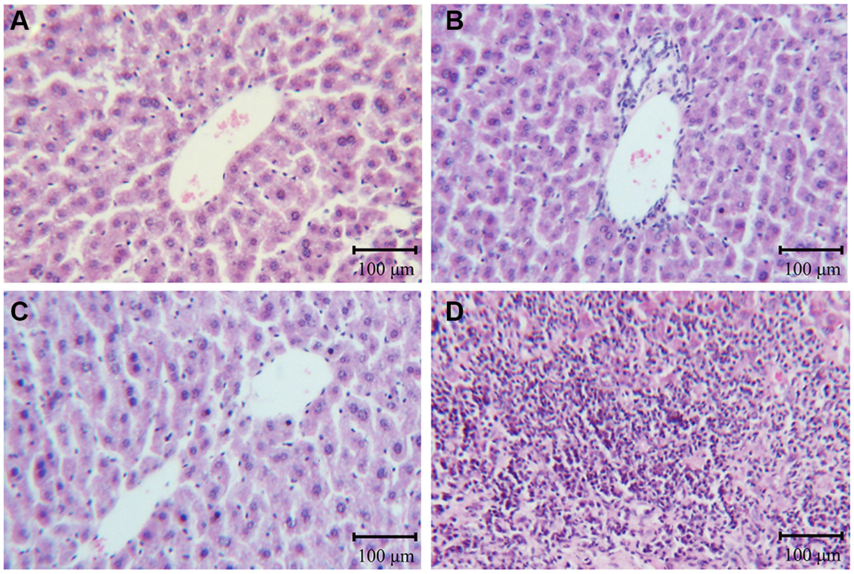

Supplementary Figure S11: The pathological features of 12M and 24M *p21*$^{HBx/+}$ mice and their WT littermates by HE staining. 12M WT (**A**) and 12M *p21*$^{HBx/+}$ mouse (**B**) liver tissue samples. 12M *p21*$^{HBx/+}$ samples show that inflammatory cells distributed in the liver portal area, representing the pathological feature of mild inflammation. 24M WT (**C**) and 24M *p21*$^{HBx/+}$ mouse (**D**) liver tissue samples. 24M *p21*$^{HBx/+}$ samples show that tumor tissues have malignant cells with the irregular cell morphology and nuclear atypia, which obviously damaged the normal structure of hepatic lobular, representing HCC pathological feature.

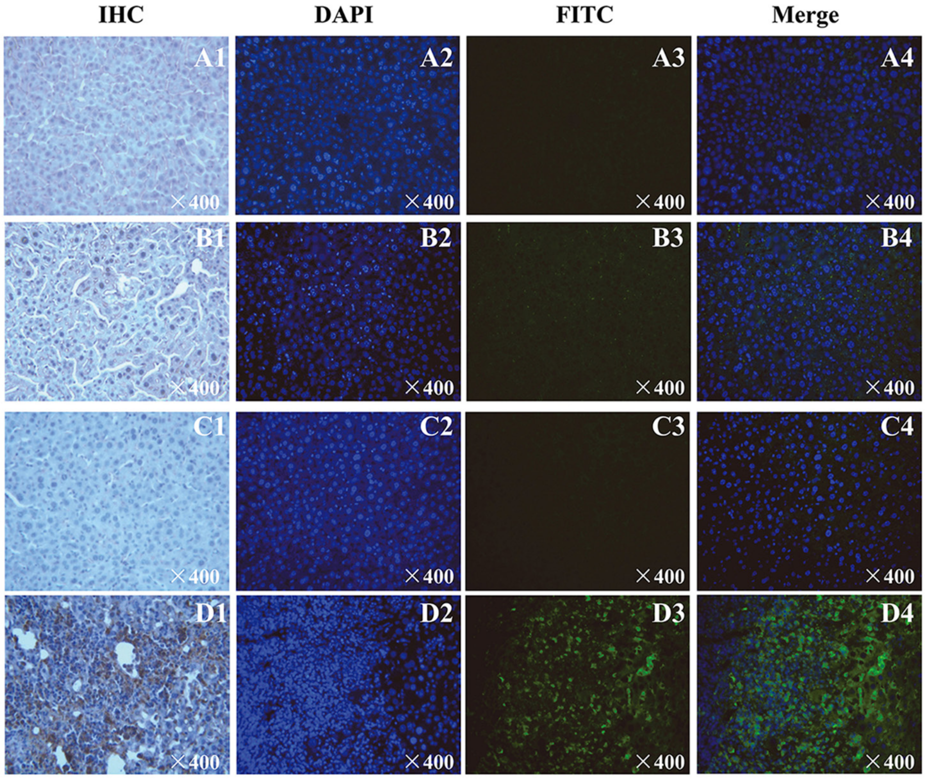

Supplementary Figure S12: The protein level of PCNA in 12M and 24M *p21*$^{HBx/+}$ mice has been significantly changed compared with their littermates. (**A**–**B**) 12M WT and 12M *p21*$^{HBx/+}$ mouse liver tissue samples, (**C**–**D**) 24M WT and 24M *p21*$^{HBx/+}$ mouse liver tissue samples. 1: The PCNA expression level was detected by IHC method. 2: The nucleus was highlighted with 4,6-diamidino-2 -phenylindole (DAPI)(blue). 3:The PCNA protein was labeled with FITC-IgG (green). Green dots under the expression of PCNA correspond to the carcinogenesis feature. 4: Merging the DAPI and FITC labeling feature under the same visual field.

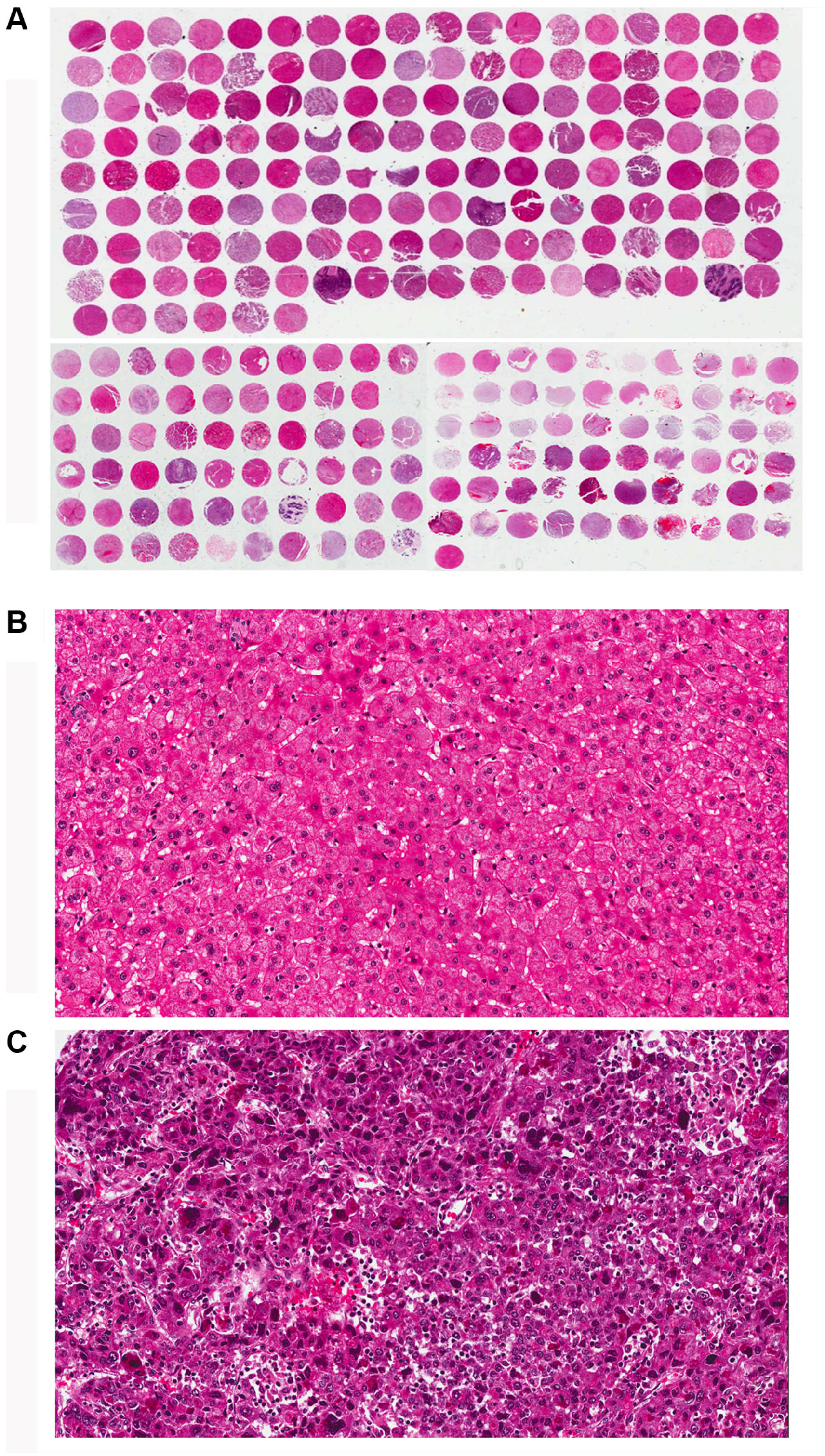

Supplementary Figure S13: The pathological features of tissues by HE staining. (**A**) Tissue microarray (×5). (**B**) Liver carcinoma (×200). (**C**) Tissue adjacent to carcinoma (×200)

Supplementary Tables S1: The quantitative data of proteomics for 12M HBx or 12 WT vs SILAC mice. See Supplementary_Tables_S1

Supplementary Tables S2 The quantitative data of proteomics for 24M HBx or 24 WT vs SILAC mice. See Supplementary_Tables_S2

Supplementary Tables S3 The quantitative data of proteomics for normal 1 or 2 mice vs SILAC mice. See Supplementary_Tables_S3

Supplementary Tables S4 The list for changed proteins identified from the comparison of 12M p21HBx+ samples and WT littermates.

Protein IDs	Gene name	Mol. weight [kDa]	(12 M Log2 HBX/ SILAM) GEL	(12 M Log2 HBX/ SILAM) 2D-LC	Mean value_HBx vs SILAM	SD	(12 M Log2 WT/ SILAM) GEL	(12 M Log2 WT/ SILAM) 2D-LC	Mean value_WT vs SILAM	SD	(12M Log2 HBx/12M WT)
P43883	PLIN2	46.65	1.83	1.79	1.83	0.02	-1.83	0.7	-0.56	1.78	2.39
P18760	CFL1	18.56	1.63	1.46	1.54	0.11	-1.63	0.01	-0.81	1.16	2.35
P60766	CDC42	21.26	1.08	1.02	1.05	0.04	-1.08	-0.02	-0.55	0.75	1.6
Q8K023	AKR1C18	37.18	0.29	0.15	0.22	0.1	0.11	-2.31	-1.1	1.71	1.32
Q9Z0M5	LIPA	45.33	2.05	0.87	1.46	0.83	0.25	0.75	0.5	0.35	0.96
Q9QUI0	RHOA	21.78	-0.02	5.12	2.55	3.63	0	3.26	1.63	2.3	0.92
P01872	IGHM	49.97	3.43	3.43	3.43	0	2.66	2.41	2.54	0.18	0.89
Q64FW2	RETSAT	67.33	2.24	2.3	2.27	0.04	1.53	1.63	1.58	0.07	0.69
Q03734	SERPINA3M	47.06	1.88	-0.22	0.83	1.48	0.88	-0.57	0.15	1.03	0.68
O55137	ACOT1	46.14	1.09	0.65	0.87	0.32	0.41	0.04	0.23	0.26	0.64
P29391	FTL1	20.8	0.13	0.44	0.28	0.22	0.69	1.15	0.92	0.33	-0.64
Q04857	COL6A1	108.49	-0.19	0.3	0.05	0.35	0.81	0.75	0.78	0.04	-0.73
Q9Z0R4	ITSN1	194.29	-0.64	-0.09	-0.36	0.39	0.79	0.02	0.41	0.55	-0.77
P27786	CYP17A1	57.64	-2.9	-2.58	-2.74	0.23	-1.94	-1.94	-1.94	0	-0.79
Q9R112	SQRDL	50.28	-0.99	-0.84	-0.92	0.1	-0.19	-0.05	-0.12	0.1	-0.8
A2ATU0	DHTKD1	102.79	0.76	0.69	0.72	0.05	1.56	1.58	1.57	0.01	-0.85
A2ASS6	TTN	3906.4	0.47	-2.83	-1.18	2.33	0.71	-0.24	0.23	0.67	-1.41
P21300	AKR1B7	35.99	-0.38	-3.67	-2.02	2.33	-0.8	-0.27	-0.54	0.37	-1.49
P01868	IGHG1	35.7	1.82	1.99	1.9	0.12	3.41	3.51	3.46	0.07	-1.56
Q9R257	HEBP1	21.07	-0.22	-0.08	-0.15	0.1	1.53	1.58	1.55	0.04	-1.7
P68134	ACTA1	42.05	-5.34	-3.02	-4.18	1.64	0.11	0.01	0.06	0.07	-4.24

Supplementary Tables S5 The list for changed proteins identified from the comparison of 24M p21HBx+ samples and WT littermates. See Supplementary_Tables_S5

Supplementary Tables S6 GO analysis for changing protein in 12M p21HBx+ samples compared with WT littermates new. See Supplementary_Tables_S6

Supplementary Tables S7 GO analysis for changing protein in 24M p21HBx+ samples compared with WT littermates. See Supplementary_Tables_S7

Supplementary Tables S8 GO analysis for HBx interacting proteins. See Supplementary_Tables_S8

Supplementary Tables S9 The biochemical characteristics of clinical tissue samples on HBV-HCC, non-HBV–HCC patients. See Supplementary_Tables_S9

Supplementary Tables S10 The biochemical characteristics of serum samples on HBV-HCC, non-HBV–HCC patients. See Supplementary_Tables_S10
