# Supplementary material for: Hepatitis B virus X induces inflammation and cancer in mice liver through dysregulation of cytoskeletal remodeling and lipid metabolism
Source: Oncotarget. 2016 Sep 30;7(43):70559–74. doi: 10.18632/oncotarget.12372 (PMC5342574; doi:10.18632/oncotarget.12372)
Supplement: Supplementary file 2 [file oncotarget-07-70559-s002.docx]

**supplemental Tables 1 The quantitative data of proteomics for 12M p21^HBx/+^ vs 12M WT mice**

| **Protein IDs** | **Gene name** | **Mol.**  **weight**  **[kDa]** | **Log2** （**12 M**  **HBX /SILAM**）  **GEL** | **Log2** （**12 M**  **HBX /SILAM**）  **2D-LC** | **Mean**  **value_HBx**  **vs SILAM** | **SD** | **Log2** （**12 M**  **WT/SILAM**）  **GEL** | **Log2** （**12 M WT**  **/SILAM**）**2D-LC** | **Mean**  **value_WT**  **vs SILAM** | **SD** | （**12M**  **Log2**  **HBx/12M WT**） |
| --- | --- | --- | --- | --- | --- | --- | --- | --- | --- | --- | --- |
| O54692 | ZW10 | 88.06 | 0.13 | -0.04 | 0.04 | 0.12 | 0.07 | 0.05 | 0.06 | 0.02 | -0.02 |
| Q8R151 | ZNFX1 | 218.83 | 0.17 | 0.03 | 0.10 | 0.10 | -0.03 | -0.09 | -0.06 | 0.04 | 0.17 |
| O88291 | ZNF326 | 65.23 | 0.35 | 0.00 | 0.18 | 0.25 | 0.21 | 0.05 | 0.13 | 0.11 | 0.05 |
| Q80W54 | ZMPSTE24 | 54.73 | 0.12 | 0.16 | 0.14 | 0.03 | 0.28 | 0.34 | 0.31 | 0.05 | -0.17 |
| Q810J8 | ZFYVE1 | 86.94 | 0.28 | 0.04 | 0.16 | 0.17 | -0.11 | -0.11 | -0.11 | 0.00 | 0.26 |
| O88532 | ZFR | 116.86 | -0.03 | -0.09 | -0.06 | 0.04 | -0.44 | -0.17 | -0.31 | 0.19 | 0.25 |
| Q5BLK4 | ZCCHC6 | 169.10 | -0.30 | 0.01 | -0.14 | 0.22 | -0.29 | 0.00 | -0.15 | 0.20 | 0.00 |
| Q3TIV5 | ZC3H15 | 48.33 | -0.14 | 0.00 | -0.07 | 0.10 | 0.00 | -0.02 | -0.01 | 0.01 | -0.06 |
| Q8BGC4 | ZADH2 | 40.53 | -0.05 | -0.04 | -0.05 | 0.01 | -0.13 | -0.03 | -0.08 | 0.07 | 0.04 |
| Q00899 | YY1 | 44.72 | -0.46 | -0.35 | -0.40 | 0.08 | -0.01 | -0.08 | -0.04 | 0.05 | -0.36 |
| P63101 | YWHAZ | 27.77 | 0.00 | 0.01 | 0.00 | 0.01 | 0.07 | 0.02 | 0.04 | 0.04 | -0.04 |

| P68254 | YWHAQ | 27.78 | 0.12 | 0.15 | 0.13 | 0.02 | 0.16 | 0.16 | 0.16 | 0.00 | -0.03 |
| --- | --- | --- | --- | --- | --- | --- | --- | --- | --- | --- | --- |
| P68510 | YWHAH | 28.21 | -0.23 | -0.26 | -0.24 | 0.02 | -0.24 | -0.30 | -0.27 | 0.04 | 0.03 |
| P61982 | YWHAG | 28.30 | 0.07 | 0.07 | 0.07 | 0.00 | -0.01 | 0.03 | 0.01 | 0.02 | 0.06 |
| P62259 | YWHAE | 29.17 | -0.10 | -0.11 | -0.11 | 0.01 | -0.10 | -0.09 | -0.10 | 0.01 | -0.01 |
| Q9CQV8 | YWHAB | 28.09 | -0.01 | -0.02 | -0.01 | 0.00 | -0.07 | -0.04 | -0.05 | 0.02 | 0.04 |
| Q8BYK6 | YTHDF3 | 63.96 | -0.49 | -0.15 | -0.32 | 0.25 | -0.22 | -0.08 | -0.15 | 0.10 | -0.17 |
|  |  |  | -0.42 | -0.19 | -0.31 | 0.16 | -0.29 | -0.01 | -0.15 | 0.20 | -0.16 |
| O88967 | YME1L1 | 80.03 | -0.03 | 0.06 | 0.01 | 0.06 | -0.19 | 0.06 | -0.06 | 0.18 | 0.07 |
| Q9CQW1 | YKT6 | 22.31 | -0.33 | -0.20 | -0.26 | 0.09 | -0.23 | -0.20 | -0.22 | 0.02 | -0.05 |
| Q91XB7 | YIF1A | 32.13 | 0.05 | 0.06 | 0.05 | 0.00 | -0.23 | 0.13 | -0.05 | 0.25 | 0.11 |
| P62960 | YBX1 | 35.73 | -0.18 | -0.25 | -0.21 | 0.05 | -0.22 | -0.26 | -0.24 | 0.03 | 0.02 |
| Q91WQ3 | YARS | 59.11 | -0.33 | -0.28 | -0.30 | 0.03 | -0.18 | -0.09 | -0.14 | 0.06 | -0.17 |
| Q3TNA1 | XYLB | 59.54 | 0.05 | -0.01 | 0.02 | 0.04 | 0.01 | 0.00 | 0.01 | 0.00 | 0.01 |

| Q9DBR1 | XRN2 | 108.69 | -0.14 | 0.05 | -0.05 | 0.13 | 0.09 | 0.05 | 0.07 | 0.03 | -0.12 |
| --- | --- | --- | --- | --- | --- | --- | --- | --- | --- | --- | --- |
| P23475 | XRCC6 | 69.48 | -0.07 | -0.20 | -0.14 | 0.09 | -0.14 | -0.10 | -0.12 | 0.02 | -0.02 |
| P27641 | XRCC5 | 83.06 | -0.05 | -0.12 | -0.09 | 0.05 | 0.03 | -0.02 | 0.00 | 0.03 | -0.09 |
| Q9CRT8 | XPOT | 109.73 | 0.02 | -0.30 | -0.14 | 0.23 | -0.29 | -0.17 | -0.23 | 0.09 | 0.09 |
| Q9EPK7 | XPO7 | 123.81 | 0.06 | -0.14 | -0.04 | 0.14 | -0.40 | -0.21 | -0.30 | 0.13 | 0.27 |
| Q6P5F9 | XPO1 | 123.09 | -0.05 | -0.15 | -0.10 | 0.07 | -0.05 | -0.08 | -0.07 | 0.02 | -0.03 |
| Q6P1B1 | XPNPEP1 | 69.59 | -0.46 | -0.51 | -0.48 | 0.04 | -0.36 | -0.52 | -0.44 | 0.11 | -0.04 |
| Q00519 | XDH | 146.56 | -0.37 | 0.05 | -0.16 | 0.30 | -0.49 | 0.02 | -0.23 | 0.36 | 0.07 |
| Q8CHP5 | WIBG | 22.69 | -0.18 | -0.18 | -0.18 | 0.00 | -0.19 | -0.01 | -0.10 | 0.13 | -0.08 |
| Q9ERF3 | WDR61 | 33.77 | -0.03 | -0.04 | -0.03 | 0.01 | -0.04 | -0.02 | -0.03 | 0.01 | 0.00 |
| P61965 | WDR5 | 36.59 | -0.21 | -0.39 | -0.30 | 0.13 | -0.32 | -0.29 | -0.31 | 0.03 | 0.01 |
| Q8BH57 | WDR48 | 76.01 | 0.02 | 0.13 | 0.07 | 0.08 | 0.07 | 0.09 | 0.08 | 0.02 | 0.00 |
| Q8K1X1 | WDR11 | 135.94 | 0.71 | -0.14 | 0.29 | 0.60 | -0.02 | -0.09 | -0.05 | 0.05 | 0.34 |

| O88342 | WDR1 | 66.41 | -0.13 | -0.14 | -0.13 | 0.01 | -0.01 | -0.11 | -0.06 | 0.07 | -0.07 |
| --- | --- | --- | --- | --- | --- | --- | --- | --- | --- | --- | --- |
| P97765 | WBP2 | 28.03 | -0.21 | 0.05 | -0.08 | 0.18 | 0.04 | 0.08 | 0.06 | 0.03 | -0.14 |
| Q91YD9 | WASL | 54.27 | -0.43 | -0.42 | -0.43 | 0.01 | -0.16 | -0.30 | -0.23 | 0.10 | -0.19 |
| Q8VDD8 | WASH1 | 51.66 | -0.48 | -0.11 | -0.30 | 0.26 | -0.26 | -0.08 | -0.17 | 0.13 | -0.12 |
|  |  | 54.07 | -0.81 | -0.01 | -0.41 | 0.56 | -0.36 | 0.08 | -0.14 | 0.32 | -0.27 |
| P32921 | WARS | 54.36 | -0.30 | -0.23 | -0.26 | 0.05 | -0.22 | -0.25 | -0.23 | 0.02 | -0.03 |
| Q8CC88 | VWA8 | 213.42 | -0.17 | -0.14 | -0.15 | 0.02 | -0.45 | -0.46 | -0.46 | 0.00 | 0.30 |
| Q99KC8 | VWA5A | 87.14 | -0.38 | -0.29 | -0.33 | 0.07 | -0.20 | -0.26 | -0.23 | 0.04 | -0.10 |
| Q8CCB4 | VPS53 | 94.42 | 0.30 | 0.04 | 0.17 | 0.18 | 0.20 | -0.01 | 0.09 | 0.15 | 0.07 |
| P46467 |  | 49.42 | -0.27 | -0.18 | -0.22 | 0.06 | -0.16 | -0.20 | -0.18 | 0.03 | -0.04 |
| P97390 | VPS45 | 65.05 | 0.33 | 0.15 | 0.24 | 0.13 | 0.14 | 0.22 | 0.18 | 0.06 | 0.06 |
| Q91XD6 | VPS36 | 43.74 | -0.28 | -0.22 | -0.25 | 0.04 | -0.08 | -0.12 | -0.10 | 0.03 | -0.15 |
| Q9EQH3 | VPS35 | 91.71 | 0.25 | 0.15 | 0.20 | 0.07 | 0.23 | 0.21 | 0.22 | 0.01 | -0.02 |

| Q9QZ88 | VPS29 | 20.50 | 0.10 | 0.24 | 0.17 | 0.10 | 0.08 | 0.21 | 0.15 | 0.09 | 0.02 |
| --- | --- | --- | --- | --- | --- | --- | --- | --- | --- | --- | --- |
| Q9D1C8 | VPS28 | 25.45 | -0.14 | -0.22 | -0.18 | 0.06 | -0.15 | -0.16 | -0.16 | 0.00 | -0.02 |
| P40336 | VPS26A | 38.11 | 0.04 | 0.08 | 0.06 | 0.03 | 0.07 | 0.15 | 0.11 | 0.06 | -0.05 |
| Q8BX70 | VPS13C | 420.08 | 0.47 | 0.30 | 0.38 | 0.12 | 0.08 | 0.24 | 0.16 | 0.11 | 0.22 |
| Q99KU0 | VMP1 | 45.96 | 0.03 | -0.04 | 0.00 | 0.05 | -0.03 | -0.04 | -0.03 | 0.01 | 0.03 |
| P20152 | VIM | 53.69 | -0.07 | 0.07 | 0.00 | 0.10 | 0.23 | 0.01 | 0.12 | 0.15 | -0.11 |
| Q60931 | VDAC3 | 30.75 | 0.14 | 0.18 | 0.16 | 0.03 | 0.12 | 0.20 | 0.16 | 0.05 | 0.00 |
| Q60930 | VDAC2 | 31.73 | -0.17 | -0.01 | -0.09 | 0.12 | 0.03 | -0.05 | -0.01 | 0.05 | -0.08 |
| Q60932 | VDAC1 | 32.35 | 0.06 | 0.07 | 0.06 | 0.01 | -0.02 | 0.01 | -0.01 | 0.02 | 0.07 |
| Q01853 | VCP | 89.32 | 0.14 | 0.08 | 0.11 | 0.04 | 0.01 | 0.01 | 0.01 | 0.00 | 0.10 |
| Q64727 | VCL | 116.72 | 0.23 | 0.28 | 0.25 | 0.03 | 0.30 | 0.33 | 0.32 | 0.02 | -0.06 |
| P61759 | VBP1 | 22.44 | -0.68 | -0.46 | -0.57 | 0.15 | -0.40 | -0.46 | -0.43 | 0.04 | -0.14 |
| Q62465 | VAT1 | 43.10 | 0.41 | 0.41 | 0.41 | 0.00 | 0.47 | 0.35 | 0.41 | 0.08 | 0.00 |

| Q9Z1Q9 | VARS | 140.21 | -0.30 | 0.13 | -0.09 | 0.31 | 0.09 | 0.11 | 0.10 | 0.01 | -0.19 |
| --- | --- | --- | --- | --- | --- | --- | --- | --- | --- | --- | --- |
| Q9QY76 | VAPB | 26.95 | 0.35 | 0.30 | 0.33 | 0.04 | 0.20 | 0.21 | 0.21 | 0.00 | 0.12 |
| Q9WV55 | VAPA | 27.86 | 0.26 | 0.23 | 0.25 | 0.02 | 0.19 | 0.19 | 0.19 | 0.00 | 0.05 |
| Q80WQ2 | VAC14 | 88.05 | -0.09 | -0.17 | -0.13 | 0.06 | -0.11 | -0.19 | -0.15 | 0.05 | 0.02 |
| Q9JI13 | UTP3 | 53.40 | -0.29 | 0.00 | -0.15 | 0.21 | -0.10 | -0.11 | -0.11 | 0.01 | -0.04 |
| P70398 | USP9X | 290.71 | 0.21 | 0.13 | 0.17 | 0.06 | 0.17 | 0.12 | 0.14 | 0.04 | 0.03 |
| Q80U87 | USP8 | 122.61 | -0.24 | -0.07 | -0.15 | 0.12 | -0.59 | 0.01 | -0.29 | 0.42 | 0.14 |
| Q6A4J8 | USP7 | 128.47 | -0.09 | -0.11 | -0.10 | 0.01 | 0.09 | 0.01 | 0.05 | 0.05 | -0.15 |
| P56399 | USP5 | 95.83 | -0.05 | -0.06 | -0.06 | 0.01 | -0.10 | -0.11 | -0.10 | 0.01 | 0.04 |
| Q8BY87 | USP47 | 157.45 | 0.85 | 0.98 | 0.91 | 0.09 | 0.69 | 0.93 | 0.81 | 0.17 | 0.10 |
| P35123 | USP4 | 108.34 | -0.70 | -0.32 | -0.51 | 0.27 | -0.38 | -0.18 | -0.28 | 0.15 | -0.23 |
| Q3TIX9 | USP39 | 65.15 | -0.12 | -0.24 | -0.18 | 0.08 | -0.19 | -0.17 | -0.18 | 0.02 | 0.00 |
| Q8R5H1 | USP15 | 112.32 | -0.34 | -0.40 | -0.37 | 0.04 | -0.28 | -0.29 | -0.29 | 0.01 | -0.08 |

| Q9JMA1 | USP14 | 56.00 | 0.03 | 0.01 | 0.02 | 0.01 | -0.05 | 0.00 | -0.02 | 0.04 | 0.05 |
| --- | --- | --- | --- | --- | --- | --- | --- | --- | --- | --- | --- |
| P52479 | USP10 | 87.02 | -0.22 | 0.26 | 0.02 | 0.34 | -0.28 | 0.35 | 0.04 | 0.44 | -0.01 |
| Q9Z1Z0 | USO1 | 106.98 | 0.06 | 0.05 | 0.05 | 0.00 | 0.03 | 0.06 | 0.05 | 0.02 | 0.01 |
| Q78IK2 | USMG5 | 6.38 | -0.06 | 0.16 | 0.05 | 0.15 | 0.08 | 0.09 | 0.08 | 0.01 | -0.04 |
| P51163 | UROS | 28.50 | 0.08 | 0.06 | 0.07 | 0.01 | 0.00 | -0.07 | -0.04 | 0.05 | 0.11 |
| P70697 | UROD | 40.69 | -0.12 | -0.17 | -0.14 | 0.04 | -0.21 | -0.14 | -0.17 | 0.05 | 0.03 |
| Q8VC12 | UROC1 | 74.59 | 0.09 | -0.06 | 0.02 | 0.11 | 0.03 | -0.05 | -0.01 | 0.06 | 0.03 |
| Q9CRB3 | URAH | 13.56 | 0.16 | 0.16 | 0.16 | 0.01 | 0.13 | 0.15 | 0.14 | 0.02 | 0.02 |
| Q9CQ69 | UQCRQ | 9.77 | -0.02 | -0.05 | -0.04 | 0.02 | 0.04 | 0.03 | 0.03 | 0.01 | -0.07 |
| Q9CR68 | UQCRFS1 | 29.37 | 0.39 | 0.44 | 0.42 | 0.03 | 0.36 | 0.49 | 0.43 | 0.10 | -0.01 |
| Q9DB77 | UQCRC2 | 48.23 | -0.02 | 0.10 | 0.04 | 0.09 | 0.03 | 0.12 | 0.08 | 0.06 | -0.04 |
| Q9CZ13 | UQCRC1 | 52.85 | 0.18 | 0.13 | 0.15 | 0.04 | 0.22 | 0.16 | 0.19 | 0.04 | -0.04 |
| Q9D855 | UQCRB | 13.53 | 0.03 | 0.13 | 0.08 | 0.07 | 0.04 | 0.16 | 0.10 | 0.08 | -0.02 |

| Q9EPU0 | UPF1 | 123.97 | -0.14 | -0.13 | -0.14 | 0.00 | -0.13 | -0.06 | -0.10 | 0.05 | -0.04 |
| --- | --- | --- | --- | --- | --- | --- | --- | --- | --- | --- | --- |
| Q8VC97 | UPB1 | 43.94 | 0.27 | 0.24 | 0.26 | 0.03 | 0.82 | 0.79 | 0.80 | 0.02 | -0.55 |
| P25688 | UOX | 35.04 | 0.23 | 0.21 | 0.22 | 0.02 | 0.42 | 0.33 | 0.37 | 0.06 | -0.16 |
| Q99KD5 | UNC45A | 103.45 | 0.36 | 0.24 | 0.30 | 0.08 | 0.38 | 0.28 | 0.33 | 0.08 | -0.03 |
| P13439 | UMPS | 52.29 | -0.77 | -0.68 | -0.72 | 0.06 | -0.73 | -0.69 | -0.71 | 0.03 | -0.01 |
| Q8JZZ0 | UGT3A2 | 59.67 | 0.09 | 0.19 | 0.14 | 0.08 | -0.08 | -0.01 | -0.05 | 0.05 | 0.18 |
| Q3UP75 | UGT3A1 | 59.70 | 1.18 | 1.11 | 1.15 | 0.05 | 0.53 | 1.04 | 0.78 | 0.36 | 0.36 |
| P17717 | UGT2B17 | 60.86 | 1.22 | 1.32 | 1.27 | 0.07 | 1.16 | 1.16 | 1.16 | 0.00 | 0.11 |
| Q8BWQ1 | UGT2A3 | 61.12 | 0.68 | 0.64 | 0.66 | 0.03 | 0.65 | 0.74 | 0.70 | 0.07 | -0.04 |
|  |  |  | 1.51 | 1.45 | 1.48 | 0.04 | 0.04 | 2.76 | 1.40 | 1.92 | 0.08 |
|  |  | 60.44 | 0.78 | 0.51 | 0.65 | 0.19 | 0.59 | 0.45 | 0.52 | 0.10 | 0.12 |
| Q63886 | UGT1A1 | 60.05 | -0.16 | -0.38 | -0.27 | 0.16 | -0.67 | -0.28 | -0.48 | 0.28 | 0.20 |
| Q91ZJ5 | UGP2 | 56.98 | -1.01 | -0.92 | -0.96 | 0.07 | -0.99 | -0.92 | -0.96 | 0.05 | -0.01 |

| Q6P5E4 | UGGT1 | 176.43 | -0.25 | -0.04 | -0.14 | 0.15 | -0.19 | -0.06 | -0.12 | 0.09 | -0.02 |
| --- | --- | --- | --- | --- | --- | --- | --- | --- | --- | --- | --- |
| O70475 | UGDH | 54.83 | 1.98 | 2.00 | 1.99 | 0.01 | 1.97 | 1.96 | 1.96 | 0.01 | 0.03 |
| Q99K23 | UFSP2 | 52.52 | 0.13 | 0.07 | 0.10 | 0.05 | 0.11 | 0.08 | 0.10 | 0.02 | 0.00 |
| P61961 | UFM1 | 9.12 | -0.22 | -0.20 | -0.21 | 0.01 | -0.27 | -0.19 | -0.23 | 0.06 | 0.03 |
| Q8CCJ3 | UFL1 | 89.52 | 0.01 | -0.15 | -0.07 | 0.11 | 0.01 | -0.04 | -0.01 | 0.04 | -0.06 |
| P70362 | UFD1L | 34.48 | 0.03 | 0.23 | 0.13 | 0.14 | 0.14 | 0.28 | 0.21 | 0.10 | -0.08 |
| Q9CR09 | UFC1 | 19.48 | -0.30 | -0.21 | -0.26 | 0.07 | -0.26 | -0.23 | -0.25 | 0.02 | -0.01 |
| Q9WUP7 | UCHL5 | 37.62 | -0.14 | -0.22 | -0.18 | 0.06 | -0.16 | -0.29 | -0.23 | 0.09 | 0.05 |
| Q9JKB1 |  | 26.15 | -0.08 | 0.01 | -0.03 | 0.06 | -0.18 | -0.12 | -0.15 | 0.04 | 0.11 |
| Q6P5G6 | UBXN7 | 52.16 | -0.30 | -0.27 | -0.28 | 0.02 | 0.13 | -0.05 | 0.04 | 0.13 | -0.32 |
| Q8VCH8 | UBXN4 | 56.47 | -0.15 | -0.11 | -0.13 | 0.03 | -0.23 | -0.22 | -0.23 | 0.01 | 0.10 |
| P25976 | UBTF | 89.51 | 0.33 | -0.13 | 0.10 | 0.32 | 0.55 | -0.08 | 0.23 | 0.45 | -0.13 |
| A2AN08 | UBR4 | 572.28 | 0.21 | -0.12 | 0.05 | 0.23 | -0.16 | -0.16 | -0.16 | 0.00 | 0.21 |

| Q5U430 | UBR3 | 212.75 | 0.10 | -0.15 | -0.03 | 0.17 | 0.19 | -0.11 | 0.04 | 0.21 | -0.06 |
| --- | --- | --- | --- | --- | --- | --- | --- | --- | --- | --- | --- |
| Q8R317 | UBQLN1 | 61.98 | -0.44 | -0.02 | -0.23 | 0.30 | -0.15 | 0.03 | -0.06 | 0.12 | -0.17 |
| Q8BGR9 | UBLCP1 | 36.84 | 0.03 | -0.10 | -0.03 | 0.09 | -0.06 | -0.05 | -0.05 | 0.01 | 0.02 |
| Q78JW9 | UBFD1 | 40.14 | -0.03 | -0.38 | -0.20 | 0.25 | -0.19 | -0.08 | -0.14 | 0.08 | -0.07 |
| E9Q735 | UBE4A | 118.20 | 0.37 | 0.43 | 0.40 | 0.04 | 0.44 | 0.36 | 0.40 | 0.05 | 0.00 |
| Q3UE37 | UBE2Z | 38.37 | -0.12 | -0.34 | -0.23 | 0.15 | -0.13 | -0.12 | -0.13 | 0.00 | -0.11 |
|  |  | 16.37 | -0.03 | 0.05 | 0.01 | 0.06 | -0.05 | -0.05 | -0.05 | 0.00 | 0.06 |
| P61089 | UBE2N | 17.14 | -0.22 | -0.08 | -0.15 | 0.10 | -0.21 | -0.15 | -0.18 | 0.04 | 0.03 |
| P68037 | UBE2L3 | 17.86 | -0.12 | -0.15 | -0.13 | 0.02 | -0.09 | -0.12 | -0.11 | 0.02 | -0.02 |
| Q8C7R4 | UBA6 | 117.96 | -0.34 | -0.48 | -0.41 | 0.10 | -0.42 | -0.54 | -0.48 | 0.08 | 0.07 |
| Q8VE47 | UBA5 | 44.79 | -0.48 | -0.41 | -0.44 | 0.05 | -0.47 | -0.29 | -0.38 | 0.13 | -0.06 |
| Q9Z1F9 | UBA2 | 70.57 | -0.13 | -0.13 | -0.13 | 0.00 | 0.01 | -0.09 | -0.04 | 0.07 | -0.10 |
| Q02053 | UBA1 | 117.81 | -0.04 | -0.15 | -0.09 | 0.07 | 0.02 | -0.06 | -0.02 | 0.05 | -0.07 |

| Q91YN5 | UAP1 | 58.61 | -0.32 | -0.26 | -0.29 | 0.04 | -0.16 | -0.14 | -0.15 | 0.01 | -0.14 |
| --- | --- | --- | --- | --- | --- | --- | --- | --- | --- | --- | --- |
| Q6NV83 | U2SURP | 118.26 | -0.06 | -0.11 | -0.09 | 0.04 | -0.33 | -0.01 | -0.17 | 0.23 | 0.08 |
| P26369 | U2AF2 | 53.52 | -0.34 | -0.12 | -0.23 | 0.15 | -0.41 | -0.10 | -0.26 | 0.22 | 0.03 |
| Q9JLT4 | TXNRD2 | 56.60 | 0.01 | 0.10 | 0.05 | 0.06 | -0.04 | 0.07 | 0.01 | 0.07 | 0.04 |
| Q9JMH6 | TXNRD1 | 67.08 | 0.31 | 0.39 | 0.35 | 0.06 | 0.38 | 0.39 | 0.39 | 0.00 | -0.04 |
| Q8CDN6 | TXNL1 | 32.24 | -0.10 | -0.16 | -0.13 | 0.04 | -0.17 | -0.25 | -0.21 | 0.06 | 0.08 |
| Q91W90 | TXNDC5 | 46.42 | -0.12 | -0.20 | -0.16 | 0.06 | -0.20 | -0.22 | -0.21 | 0.02 | 0.05 |
| Q9CQU0 | TXNDC12 | 19.05 | -0.26 | -0.29 | -0.27 | 0.02 | -0.36 | -0.21 | -0.28 | 0.10 | 0.01 |
| P97493 | TXN2 | 18.26 | 0.03 | 0.13 | 0.08 | 0.07 | 0.01 | 0.15 | 0.08 | 0.10 | 0.00 |
| P10639 | TXN | 11.68 | -0.04 | 0.08 | 0.02 | 0.09 | 0.05 | 0.04 | 0.05 | 0.01 | -0.02 |
| Q6PAM1 | TXLNA | 62.37 | -0.26 | -0.23 | -0.25 | 0.02 | -0.26 | -0.27 | -0.27 | 0.01 | 0.02 |
| Q91YR1 | TWF1 | 40.08 | 0.14 | 0.13 | 0.13 | 0.01 | 0.18 | 0.16 | 0.17 | 0.01 | -0.04 |
| Q8BFR5 | TUFM | 49.51 | 0.02 | 0.01 | 0.01 | 0.01 | -0.01 | 0.08 | 0.04 | 0.07 | -0.02 |

| Q922F4 | TUBB6 | 50.09 | -0.03 | -0.67 | -0.35 | 0.45 | -0.25 | -0.53 | -0.39 | 0.20 | 0.04 |
| --- | --- | --- | --- | --- | --- | --- | --- | --- | --- | --- | --- |
| P68372 | TUBB4B | 49.83 | 0.00 | 0.11 | 0.06 | 0.08 | -0.01 | -0.02 | -0.02 | 0.01 | 0.07 |
| P68368 | TUBA4A | 49.92 | -0.03 | 0.09 | 0.03 | 0.08 | 0.09 | 0.05 | 0.07 | 0.03 | -0.04 |
| P07309 | TTR | 15.78 | -0.20 | -0.18 | -0.19 | 0.01 | -0.51 | -0.34 | -0.42 | 0.12 | 0.23 |
| Q8BWP5 | TTPA | 32.01 | 0.15 | 0.18 | 0.17 | 0.03 | 0.08 | 0.09 | 0.08 | 0.01 | 0.08 |
| A2ASS6 | TTN | 3906.40 | 0.47 | -2.83 | -1.18 | 2.33 | 0.71 | -0.24 | 0.23 | 0.67 | -1.41 |
| Q3UDE2 | TTLL12 | 74.04 | -0.13 | -0.20 | -0.17 | 0.05 | 0.07 | -0.16 | -0.04 | 0.16 | -0.12 |
| Q8VE09 | TTC39C | 65.44 | 1.86 | 1.88 | 1.87 | 0.01 | 1.85 | 1.88 | 1.86 | 0.03 | 0.01 |
| A3KMP2 | TTC38 | 52.22 | 0.12 | -0.11 | 0.00 | 0.16 | -0.47 | -0.63 | -0.55 | 0.12 | 0.55 |
| Q91Z38 | TTC1 | 33.26 | -0.08 | -0.14 | -0.11 | 0.04 | -0.26 | -0.16 | -0.21 | 0.07 | 0.10 |
| P23591 | TSTA3 | 35.88 | 0.00 | 0.02 | 0.01 | 0.01 | -0.04 | 0.06 | 0.01 | 0.07 | 0.00 |
| P52196 | TST | 33.47 | 0.54 | 0.16 | 0.35 | 0.27 | 0.41 | 0.17 | 0.29 | 0.17 | 0.06 |
| Q62348 | TSN | 26.20 | -0.44 | -0.23 | -0.33 | 0.15 | -0.36 | -0.27 | -0.31 | 0.06 | -0.02 |

| Q9CZR8 | TSFM | 35.33 | -0.23 | -0.04 | -0.13 | 0.13 | -0.10 | -0.04 | -0.07 | 0.05 | -0.06 |
| --- | --- | --- | --- | --- | --- | --- | --- | --- | --- | --- | --- |
| Q8K1J6 | TRNT1 | 49.90 | 0.18 | -0.05 | 0.07 | 0.17 | 0.07 | -0.09 | -0.01 | 0.11 | 0.08 |
| Q62318 | TRIM28 | 88.85 | -0.35 | -0.27 | -0.31 | 0.06 | -0.18 | -0.18 | -0.18 | 0.00 | -0.13 |
| Q61510 | TRIM25 | 71.73 | 0.20 | 0.12 | 0.16 | 0.05 | 0.24 | 0.18 | 0.21 | 0.04 | -0.05 |
| Q8BVW3 | TRIM14 | 49.64 | -0.59 | -0.44 | -0.51 | 0.11 | -0.35 | -0.18 | -0.26 | 0.11 | -0.25 |
| B2RXC1 | TRAPPC11 | 128.39 | 0.13 | -0.08 | 0.03 | 0.15 | 0.16 | -0.02 | 0.07 | 0.12 | -0.04 |
| Q9CQN1 | TRAP1 | 80.21 | 0.15 | 0.12 | 0.14 | 0.02 | 0.12 | 0.12 | 0.12 | 0.00 | 0.02 |
| P63028 | TPT1 | 19.46 | -0.49 | -0.40 | -0.44 | 0.06 | -0.43 | -0.28 | -0.35 | 0.10 | -0.09 |
| F6ZDS4 | TPR | 273.99 | -0.16 | 0.03 | -0.06 | 0.13 | -0.01 | 0.08 | 0.04 | 0.06 | -0.10 |
| Q64514 | TPP2 | 139.88 | -0.24 | -0.14 | -0.19 | 0.07 | -0.09 | -0.10 | -0.10 | 0.01 | -0.09 |
| O55060 | TPMT | 27.59 | 0.32 | 0.22 | 0.27 | 0.07 | 0.59 | 0.67 | 0.63 | 0.06 | -0.36 |
| Q6IRU2 | TPM4 | 28.47 | -0.29 | -0.41 | -0.35 | 0.08 | -0.25 | -0.46 | -0.35 | 0.15 | 0.00 |
| P21107 | TPM3 | 32.99 | 0.01 | -0.01 | 0.00 | 0.02 | 0.06 | 0.02 | 0.04 | 0.03 | -0.04 |

| P58771 | TPM1 | 32.68 | -0.09 | -0.72 | -0.41 | 0.44 | -0.18 | -0.87 | -0.52 | 0.48 | 0.12 |
| --- | --- | --- | --- | --- | --- | --- | --- | --- | --- | --- | --- |
| P17751 | TPI1 | 32.19 | 0.10 | 0.07 | 0.09 | 0.02 | 0.13 | 0.13 | 0.13 | 0.00 | -0.05 |
| Q9CYZ2 | TPD52L2 | 24.04 | -0.09 | -0.07 | -0.08 | 0.02 | -0.05 | 0.06 | 0.00 | 0.08 | -0.09 |
| Q9EQJ0 | TPCN1 | 94.50 | 0.64 | 0.14 | 0.39 | 0.36 | 0.45 | 0.58 | 0.52 | 0.09 | -0.13 |
| Q9ER41 | TOR1B | 37.82 | 0.40 | 0.30 | 0.35 | 0.07 | 0.33 | 0.17 | 0.25 | 0.11 | 0.10 |
| Q8BYU6 | TOR1AIP2 | 54.50 | 0.17 | 0.26 | 0.21 | 0.06 | 0.18 | 0.24 | 0.21 | 0.04 | 0.00 |
| Q9ER39 | TOR1A | 37.83 | -0.11 | -0.18 | -0.14 | 0.05 | -0.10 | -0.11 | -0.11 | 0.01 | -0.03 |
| Q9Z321 | TOP3B | 96.95 | 0.18 | 0.05 | 0.12 | 0.10 | -0.15 | 0.09 | -0.03 | 0.17 | 0.14 |
| Q64511 | TOP2B | 181.91 | -0.06 | 0.00 | -0.03 | 0.05 | -0.11 | 0.04 | -0.03 | 0.10 | 0.01 |
| Q04750 | TOP1 | 90.88 | -0.12 | -0.20 | -0.16 | 0.06 | 0.13 | -0.19 | -0.03 | 0.23 | -0.13 |
| Q9CZW5 | TOMM70A | 67.59 | -0.01 | 0.08 | 0.03 | 0.06 | -0.04 | 0.03 | 0.00 | 0.05 | 0.03 |
| Q9QYA2 | TOMM40 | 37.90 | -0.36 | -0.04 | -0.20 | 0.23 | -0.72 | 0.00 | -0.36 | 0.51 | 0.16 |
| Q9CYG7 | TOMM34 | 34.28 | 0.05 | -0.23 | -0.09 | 0.20 | -0.25 | -0.26 | -0.26 | 0.01 | 0.17 |

| Q6P2B1 | TNPO3 | 104.17 | 0.07 | -0.20 | -0.06 | 0.19 | -0.08 | -0.10 | -0.09 | 0.01 | 0.03 |
| --- | --- | --- | --- | --- | --- | --- | --- | --- | --- | --- | --- |
| Q99LG2 | TNPO2 | 100.46 | -0.08 | -0.14 | -0.11 | 0.04 | 0.03 | -0.06 | -0.01 | 0.06 | -0.10 |
| Q8BFY9 | TNPO1 | 102.36 | 0.00 | -0.03 | -0.02 | 0.02 | 0.15 | 0.04 | 0.10 | 0.08 | -0.12 |
| Q9QZ47 | TNNT3 | 32.24 | -4.96 | -4.91 | -4.94 | 0.04 | -4.82 | -4.80 | -4.81 | 0.02 | -0.12 |
| P58871 | TNKS1BP1 | 181.82 | -0.91 | -0.76 | -0.83 | 0.11 | -0.82 | -0.33 | -0.58 | 0.35 | -0.26 |
| Q80YX1 | TNC | 231.80 | -0.80 | -0.66 | -0.73 | 0.09 | -0.77 | -0.64 | -0.71 | 0.09 | -0.02 |
| Q8BXZ1 | TMX3 | 51.85 | -0.12 | -0.04 | -0.08 | 0.06 | 0.19 | -0.04 | 0.07 | 0.17 | -0.15 |
| Q9D710 | TMX2 | 33.94 | -0.22 | -0.07 | -0.14 | 0.11 | -0.11 | -0.34 | -0.23 | 0.16 | 0.08 |
| Q8VBT0 | TMX1 | 31.40 | -0.35 | -0.27 | -0.31 | 0.05 | -0.36 | -0.39 | -0.37 | 0.02 | 0.06 |
| Q8BRH0 | TMTC3 | 104.20 | -0.29 | -0.13 | -0.21 | 0.12 | -0.21 | -0.12 | -0.16 | 0.06 | -0.05 |
| Q61029 | TMPO | 50.37 | -0.64 | -0.44 | -0.54 | 0.14 | -0.18 | -0.34 | -0.26 | 0.12 | -0.28 |
| Q9JHJ0 | TMOD3 | 39.50 | 0.10 | -0.02 | 0.04 | 0.08 | 0.07 | 0.07 | 0.07 | 0.00 | -0.03 |
| Q8VEK0 | TMEM30A | 41.06 | 0.57 | 0.57 | 0.57 | 0.00 | 0.61 | 0.67 | 0.64 | 0.04 | -0.06 |

| Q8BM55 | TMEM214 | 76.43 | -0.19 | -0.11 | -0.15 | 0.06 | -0.03 | -0.03 | -0.03 | 0.00 | -0.12 |
| --- | --- | --- | --- | --- | --- | --- | --- | --- | --- | --- | --- |
| Q8C1E7 | TMEM120A | 40.75 | 0.95 | 0.93 | 0.94 | 0.01 | 0.65 | 0.67 | 0.66 | 0.02 | 0.28 |
| Q9WUH1 | TMEM115 | 38.10 | 0.41 | -0.05 | 0.18 | 0.33 | 0.61 | 0.25 | 0.43 | 0.26 | -0.25 |
| Q9D1D4 | TMED10 | 24.91 | 0.46 | 0.19 | 0.33 | 0.19 | 0.08 | 0.21 | 0.15 | 0.09 | 0.18 |
| Q921L3 | TMCO1 | 21.18 | 0.28 | 0.36 | 0.32 | 0.06 | 0.33 | 0.40 | 0.36 | 0.05 | -0.04 |
| Q71LX4 | TLN2 | 253.62 | 0.86 | 0.72 | 0.79 | 0.10 | 0.86 | 0.80 | 0.83 | 0.04 | -0.04 |
| P26039 | TLN1 | 269.82 | -0.15 | -0.18 | -0.17 | 0.02 | -0.04 | -0.07 | -0.05 | 0.02 | -0.11 |
| P40142 | TKT | 67.63 | -0.15 | -0.27 | -0.21 | 0.09 | -0.17 | -0.33 | -0.25 | 0.11 | 0.04 |
| Q9QXY1 | TJP3 | 99.32 | -0.53 | -0.22 | -0.37 | 0.22 | -0.22 | -0.25 | -0.24 | 0.02 | -0.14 |
| Q9Z0U1 | TJP2 | 131.28 | -0.76 | -0.15 | -0.46 | 0.44 | -0.56 | -0.10 | -0.33 | 0.32 | -0.13 |
| P39447 | TJP1 | 194.74 | -0.66 | -0.16 | -0.41 | 0.35 | -0.60 | -0.11 | -0.36 | 0.34 | -0.05 |
| Q8BH58 | TIPRL | 31.25 | -0.32 | -0.41 | -0.37 | 0.07 | -0.34 | -0.39 | -0.36 | 0.04 | 0.00 |
| Q9D880 | TIMM50 | 39.78 | 0.03 | 0.11 | 0.07 | 0.05 | 0.06 | 0.08 | 0.07 | 0.02 | 0.00 |

| O35857 | TIMM44 | 51.09 | -0.14 | -0.04 | -0.09 | 0.07 | -0.23 | -0.03 | -0.13 | 0.14 | 0.04 |
| --- | --- | --- | --- | --- | --- | --- | --- | --- | --- | --- | --- |
| P62075 | TIMM13 | 10.46 | -0.02 | 0.11 | 0.04 | 0.09 | 0.07 | 0.08 | 0.07 | 0.01 | -0.03 |
| P70318 | TIAL1 | 43.39 | -0.20 | -0.17 | -0.18 | 0.02 | -0.28 | -0.14 | -0.21 | 0.10 | 0.03 |
| Q91YJ3 | THYN1 | 26.18 | -0.24 | -0.20 | -0.22 | 0.03 | -0.20 | -0.14 | -0.17 | 0.04 | -0.04 |
| Q99J36 | THUMPD1 | 38.88 | -0.08 | -0.17 | -0.13 | 0.07 | -0.05 | -0.02 | -0.03 | 0.02 | -0.10 |
| Q8JZL3 | THTPA | 24.26 | 0.20 | 0.43 | 0.32 | 0.16 | 0.25 | 0.38 | 0.31 | 0.09 | 0.00 |
| Q569Z6 | THRAP3 | 108.18 | -0.47 | -0.08 | -0.28 | 0.28 | -0.19 | -0.10 | -0.14 | 0.06 | -0.13 |
| Q8C1A5 | THOP1 | 78.03 | 0.02 | -0.03 | 0.00 | 0.04 | 0.00 | -0.08 | -0.04 | 0.06 | 0.04 |
| Q8BKT7 | THOC5 | 78.69 | -0.46 | -0.30 | -0.38 | 0.12 | -0.15 | -0.19 | -0.17 | 0.03 | -0.21 |
| Q8R3N6 | THOC1 | 75.44 | -0.23 | -0.13 | -0.18 | 0.07 | -0.10 | -0.12 | -0.11 | 0.02 | -0.07 |
| Q80W22 | THNSL2 | 54.19 | 0.02 | -0.01 | 0.01 | 0.03 | -0.16 | -0.09 | -0.13 | 0.05 | 0.13 |
| Q3UUI3 | THEM4 | 26.03 | 0.16 | 0.31 | 0.23 | 0.11 | 0.13 | 0.23 | 0.18 | 0.07 | 0.05 |
| P35441 | THBS1 | 129.65 | 0.23 | 0.55 | 0.39 | 0.23 | 0.44 | 0.48 | 0.46 | 0.03 | -0.07 |

|  |  |  | 0.93 | 0.85 | 0.89 | 0.05 | 0.77 | 0.75 | 0.76 | 0.01 | 0.13 |
| --- | --- | --- | --- | --- | --- | --- | --- | --- | --- | --- | --- |
| P21981 | TGM2 | 77.06 | -0.30 | -0.36 | -0.33 | 0.04 | -0.14 | -0.35 | -0.25 | 0.14 | -0.08 |
| Q9JLF6 | TGM1 | 89.83 | 0.46 | 0.21 | 0.33 | 0.18 | 0.43 | 0.29 | 0.36 | 0.10 | -0.03 |
| P82198 | TGFBI | 74.60 | 0.21 | 0.03 | 0.12 | 0.13 | 0.00 | 0.12 | 0.06 | 0.08 | 0.06 |
| Q62351 | TFRC | 85.73 | -0.19 | -0.37 | -0.28 | 0.13 | -0.14 | -0.39 | -0.26 | 0.18 | -0.01 |
| Q9JKX3 | TFR2 | 88.40 | 0.59 | 0.43 | 0.51 | 0.11 | 0.17 | 0.46 | 0.31 | 0.20 | 0.20 |
| P40630 | TFAM | 27.99 | -0.19 | -0.02 | -0.10 | 0.12 | -0.13 | 0.00 | -0.07 | 0.10 | -0.04 |
| Q921I1 | TF | 76.72 | -0.02 | -0.07 | -0.04 | 0.04 | -0.15 | -0.17 | -0.16 | 0.01 | 0.11 |
| Q5SSK3 | TEFM | 41.87 | -0.12 | 0.25 | 0.06 | 0.26 | 0.00 | 0.18 | 0.09 | 0.13 | -0.02 |
| Q9CY27 | TECR | 36.09 | -0.33 | -0.11 | -0.22 | 0.16 | -0.24 | -0.06 | -0.15 | 0.13 | -0.07 |
| P48776 | TDO2 | 47.76 | 1.65 | 1.62 | 1.64 | 0.03 | 1.50 | 1.61 | 1.55 | 0.08 | 0.08 |
| P11983 | TCP1 | 60.45 | -0.35 | -0.36 | -0.36 | 0.01 | -0.52 | -0.40 | -0.46 | 0.08 | 0.11 |
| Q8CGF7 | TCERG1 | 123.79 | -0.78 | -0.03 | -0.40 | 0.53 | -0.48 | 0.01 | -0.24 | 0.34 | -0.17 |

| P83940 | TCEB1 | 12.47 | -0.20 | -0.23 | -0.22 | 0.02 | -0.24 | -0.14 | -0.19 | 0.07 | -0.03 |
| --- | --- | --- | --- | --- | --- | --- | --- | --- | --- | --- | --- |
| P10711 | TCEA1 | 33.88 | -0.23 | -0.34 | -0.28 | 0.08 | -0.17 | -0.18 | -0.18 | 0.01 | -0.11 |
| Q91YM4 | TBRG4 | 71.51 | 0.06 | 0.11 | 0.08 | 0.03 | -0.03 | 0.17 | 0.07 | 0.14 | 0.02 |
| Q9R099 | TBL2 | 49.58 | 0.23 | 0.41 | 0.32 | 0.13 | -0.02 | 0.13 | 0.06 | 0.11 | 0.26 |
| Q8C5W3 | TBCEL | 48.03 | -0.24 | -0.27 | -0.25 | 0.02 | -0.33 | -0.33 | -0.33 | 0.00 | 0.08 |
| P48428 | TBCA | 12.76 | -0.30 | -0.06 | -0.18 | 0.16 | -0.29 | -0.24 | -0.27 | 0.04 | 0.09 |
| A3KGB4 | TBC1D8B | 127.89 | -0.21 | -0.21 | -0.21 | 0.00 | -0.18 | -0.19 | -0.18 | 0.00 | -0.02 |
| Q9CXF4 | TBC1D15 | 76.53 | 0.12 | 0.08 | 0.10 | 0.03 | 0.18 | 0.16 | 0.17 | 0.02 | -0.07 |
| Q6P8M1 | TATDN1 | 33.37 | -0.22 | -0.16 | -0.19 | 0.04 | 0.00 | -0.14 | -0.07 | 0.10 | -0.12 |
| Q8QZR1 | TAT | 50.57 | 1.97 | 2.06 | 2.01 | 0.06 | 1.68 | 1.78 | 1.73 | 0.07 | 0.28 |
| Q9D0R2 | TARS | 83.36 | -0.06 | -0.17 | -0.11 | 0.08 | -0.19 | -0.20 | -0.20 | 0.01 | 0.09 |
| Q921F2 | TARDBP | 44.55 | -0.21 | -0.11 | -0.16 | 0.07 | -0.15 | -0.07 | -0.11 | 0.06 | -0.05 |
| Q4VBD2 | TAPT1 | 63.89 | 0.40 | -0.59 | -0.09 | 0.70 | 0.19 | -0.19 | 0.00 | 0.27 | -0.10 |

| Q9R233 | TAPBP | 49.74 | -0.61 | -0.57 | -0.59 | 0.03 | -0.50 | -0.53 | -0.51 | 0.02 | -0.07 |
| --- | --- | --- | --- | --- | --- | --- | --- | --- | --- | --- | --- |
| Q8BYC6 | TAOK3 | 105.33 | -0.26 | -0.32 | -0.29 | 0.04 | -0.22 | -0.31 | -0.27 | 0.07 | -0.02 |
| P54797 | TANGO2 | 30.95 | -0.49 | -0.54 | -0.52 | 0.04 | -0.38 | -0.31 | -0.35 | 0.05 | -0.17 |
| Q93092 | TALDO1 | 37.39 | 0.39 | 0.24 | 0.31 | 0.11 | 0.23 | 0.21 | 0.22 | 0.01 | 0.10 |
| Q9WVA4 | TAGLN2 | 22.40 | -0.49 | -0.30 | -0.39 | 0.14 | -0.22 | -0.33 | -0.28 | 0.08 | -0.12 |
| P37804 | TAGLN | 22.58 | 0.61 | 0.62 | 0.61 | 0.01 | 0.98 | 0.55 | 0.77 | 0.30 | -0.15 |
| Q8K0Z7 | TACO1 | 32.31 | 0.36 | 0.38 | 0.37 | 0.02 | 0.44 | 0.39 | 0.42 | 0.03 | -0.05 |
| Q7TMK9 | SYNCRIP | 69.63 | 0.01 | 0.00 | 0.00 | 0.01 | 0.07 | 0.00 | 0.04 | 0.05 | -0.03 |
| Q9D5V6 | SYAP1 | 41.35 | -0.71 | -0.24 | -0.47 | 0.33 | -0.56 | -0.18 | -0.37 | 0.27 | -0.10 |
| Q6A028 | SWAP70 | 69.00 | -0.13 | -0.06 | -0.09 | 0.05 | 0.14 | 0.06 | 0.10 | 0.06 | -0.19 |
| Q8K4L3 | SVIL | 243.16 | -0.67 | -0.29 | -0.48 | 0.27 | -0.61 | -0.28 | -0.44 | 0.24 | -0.04 |
| Q64310 | SURF4 | 30.38 | 0.03 | -0.18 | -0.07 | 0.15 | 0.11 | -0.03 | 0.04 | 0.10 | -0.11 |
| Q80YD1 | SUPV3L1 | 87.00 | -0.02 | -0.06 | -0.04 | 0.03 | -0.05 | 0.09 | 0.02 | 0.10 | -0.06 |

| O55201 | SUPT5H | 120.66 | -0.85 | -0.13 | -0.49 | 0.51 | -0.65 | -0.03 | -0.34 | 0.44 | -0.15 |
| --- | --- | --- | --- | --- | --- | --- | --- | --- | --- | --- | --- |
| Q920B9 | SUPT16H | 119.82 | -0.56 | -0.29 | -0.43 | 0.19 | 0.01 | -0.09 | -0.04 | 0.07 | -0.39 |
| Q8R086 | SUOX | 60.76 | 0.41 | 0.44 | 0.42 | 0.02 | 0.35 | 0.29 | 0.32 | 0.04 | 0.10 |
| Q8BJS4 | SUN2 | 81.60 | 0.31 | 0.25 | 0.28 | 0.05 | 0.31 | 0.25 | 0.28 | 0.04 | 0.00 |
| P52843 | SULT2A1 | 33.21 | -5.12 | 0.44 | -2.34 | 3.93 | -4.91 | 0.38 | -2.26 | 3.74 | -0.08 |
| Q3UZZ6 | SULT1D1 | 35.08 | -0.72 | -0.73 | -0.72 | 0.01 | -0.50 | -0.47 | -0.49 | 0.02 | -0.23 |
| Q9D939 | SULT1C2 | 34.95 | -2.35 | -2.44 | -2.39 | 0.06 | -2.19 | -2.32 | -2.26 | 0.09 | -0.14 |
| Q9QWG7 | SULT1B1 | 34.90 | 0.22 | 0.30 | 0.26 | 0.06 | 0.31 | 0.27 | 0.29 | 0.02 | -0.03 |
| P52840 | SULT1A1 | 33.97 | -1.10 | -1.15 | -1.13 | 0.04 | -0.80 | -0.90 | -0.85 | 0.07 | -0.28 |
| Q9CX34 | SUGT1 | 38.16 | -0.32 | -0.36 | -0.34 | 0.03 | -0.40 | -0.33 | -0.36 | 0.06 | 0.02 |
| Q7TNE1 | SUGCT | 47.69 | 1.61 | 1.45 | 1.53 | 0.12 | 1.10 | 1.12 | 1.11 | 0.01 | 0.42 |
| Q9Z2I8 | SUCLG2 | 46.84 | 0.02 | 0.02 | 0.02 | 0.00 | -0.02 | 0.07 | 0.02 | 0.06 | -0.01 |
| Q9WUM5 | SUCLG1 | 36.15 | 0.07 | 0.00 | 0.04 | 0.05 | 0.02 | 0.01 | 0.01 | 0.01 | 0.02 |

| Q9Z2I9 | SUCLA2 | 50.11 | -0.04 | 0.01 | -0.02 | 0.03 | -0.03 | 0.03 | 0.00 | 0.04 | -0.02 |
| --- | --- | --- | --- | --- | --- | --- | --- | --- | --- | --- | --- |
| P11031 | SUB1 | 14.43 | -0.72 | -0.73 | -0.73 | 0.01 | -0.49 | -0.55 | -0.52 | 0.04 | -0.21 |
| Q60770 | STXBP3 | 67.94 | -0.07 | -0.05 | -0.06 | 0.01 | -0.01 | 0.05 | 0.02 | 0.04 | -0.08 |
| Q64324 | STXBP2 | 66.36 | 0.09 | -0.08 | 0.00 | 0.12 | -0.06 | -0.14 | -0.10 | 0.06 | 0.10 |
| O88983 | STX8 | 26.93 | -0.37 | -0.10 | -0.23 | 0.19 | -0.27 | -0.22 | -0.24 | 0.04 | 0.01 |
| O70439 | STX7 | 29.82 | -0.20 | 0.34 | 0.07 | 0.38 | 0.50 | -0.16 | 0.17 | 0.46 | -0.10 |
| Q8K1E0 | STX5 | 39.71 | -0.12 | -0.01 | -0.07 | 0.08 | -0.59 | -0.05 | -0.32 | 0.38 | 0.26 |
| P70452 | STX4 | 34.17 | -0.01 | -0.07 | -0.04 | 0.04 | 0.09 | 0.00 | 0.04 | 0.06 | -0.08 |
| Q8VDS8 | STX18 | 38.38 | -0.08 | 0.01 | -0.03 | 0.06 | 0.13 | -0.03 | 0.05 | 0.11 | -0.08 |
| Q9ER00 | STX12 | 31.20 | 0.15 | 0.06 | 0.10 | 0.07 | 0.04 | 0.03 | 0.03 | 0.00 | 0.07 |
| Q3TDQ1 | STT3B | 93.25 | -0.07 | -0.23 | -0.15 | 0.12 | -0.26 | -0.22 | -0.24 | 0.02 | 0.09 |
| P46978 | STT3A | 80.60 | 0.11 | 0.08 | 0.10 | 0.03 | 0.17 | 0.16 | 0.17 | 0.01 | -0.07 |
| Q9ERG2 | STRN3 | 87.15 | -0.33 | -0.28 | -0.31 | 0.04 | -0.29 | -0.21 | -0.25 | 0.06 | -0.06 |

| Q9Z1Z2 | STRAP | 38.44 | 0.10 | 0.09 | 0.10 | 0.01 | 0.09 | 0.06 | 0.08 | 0.02 | 0.02 |
| --- | --- | --- | --- | --- | --- | --- | --- | --- | --- | --- | --- |
| Q99JB2 | STOML2 | 38.38 | 0.21 | 0.26 | 0.24 | 0.03 | 0.16 | 0.18 | 0.17 | 0.02 | 0.07 |
| P54116 | STOM | 31.38 | -0.11 | 0.01 | -0.05 | 0.09 | -0.07 | 0.08 | 0.00 | 0.10 | -0.05 |
| Q99KH8 | STK24 | 47.95 | 0.08 | -0.12 | -0.02 | 0.14 | 0.06 | -0.06 | 0.00 | 0.08 | -0.02 |
| Q60864 | STIP1 | 62.58 | -0.33 | -0.25 | -0.29 | 0.05 | -0.33 | -0.32 | -0.32 | 0.00 | 0.03 |
| P70302 | STIM1 | 77.57 | 0.05 | 0.11 | 0.08 | 0.04 | 0.21 | 0.15 | 0.18 | 0.04 | -0.10 |
| Q923B6 | STEAP4 | 52.99 | 1.02 | 0.91 | 0.96 | 0.07 | 1.34 | 0.97 | 1.15 | 0.26 | -0.19 |
| Q8C7E7 | STBD1 | 36.13 | 0.59 | 0.58 | 0.58 | 0.01 | 0.73 | 0.66 | 0.70 | 0.05 | -0.11 |
| Q9Z108 | STAU1 | 53.92 | -0.51 | -0.05 | -0.28 | 0.33 | -0.44 | -0.12 | -0.28 | 0.23 | 0.00 |
|  |  | 90.00 | -0.86 | -0.78 | -0.82 | 0.05 | -0.74 | -0.69 | -0.71 | 0.03 | -0.10 |
| P42227 | STAT3 | 88.05 | 0.13 | 0.03 | 0.08 | 0.07 | 0.29 | 0.15 | 0.22 | 0.10 | -0.14 |
| P42225 | STAT1 | 87.20 | -1.20 | -1.30 | -1.25 | 0.07 | -0.91 | -1.25 | -1.08 | 0.24 | -0.17 |
| Q9JMD3 | STARD10 | 32.95 | 0.55 | 0.42 | 0.49 | 0.09 | 0.38 | 0.41 | 0.39 | 0.02 | 0.09 |

| Q8R4U0 | STAB2 | 277.53 | -0.53 | -0.61 | -0.57 | 0.06 | -0.45 | -0.42 | -0.43 | 0.03 | -0.13 |
| --- | --- | --- | --- | --- | --- | --- | --- | --- | --- | --- | --- |
| Q99L47 | ST13 | 41.66 | -0.17 | -0.18 | -0.18 | 0.00 | -0.31 | -0.27 | -0.29 | 0.03 | 0.11 |
| Q08943 | SSRP1 | 80.86 | -0.09 | -0.16 | -0.12 | 0.05 | 0.02 | -0.17 | -0.08 | 0.14 | -0.05 |
| Q62186 | SSR4 | 18.94 | 0.27 | 0.10 | 0.19 | 0.12 | 0.07 | 0.12 | 0.10 | 0.03 | 0.09 |
| Q9DCF9 | SSR3 | 21.06 | -0.06 | 0.20 | 0.07 | 0.18 | 0.10 | 0.21 | 0.16 | 0.08 | -0.09 |
| Q9CY50 | SSR1 | 32.07 | 0.29 | 0.13 | 0.21 | 0.11 | 0.05 | 0.17 | 0.11 | 0.08 | 0.10 |
| P32067 | SSB | 47.76 | -0.20 | -0.18 | -0.19 | 0.02 | -0.19 | -0.15 | -0.17 | 0.03 | -0.01 |
| O35326 | SRSF5 | 30.89 | 0.03 | -0.07 | -0.02 | 0.07 | -0.08 | -0.13 | -0.11 | 0.04 | 0.09 |
| Q8VE97 | SRSF4 | 55.98 | -0.01 | -0.15 | -0.08 | 0.10 | -0.03 | -0.23 | -0.13 | 0.14 | 0.05 |
| Q6PDM2 | SRSF1 | 27.74 | -0.06 | -0.13 | -0.09 | 0.05 | -0.04 | -0.19 | -0.11 | 0.11 | 0.02 |
| Q99MR6 | SRRT | 100.45 | 0.06 | 0.05 | 0.06 | 0.00 | 0.09 | 0.07 | 0.08 | 0.02 | -0.02 |
| Q9QZX7 | SRR | 36.36 | 0.51 | 0.49 | 0.50 | 0.01 | 0.38 | 0.31 | 0.34 | 0.05 | 0.15 |
| P47758 | SRPRB | 29.58 | 0.31 | 0.34 | 0.32 | 0.02 | 0.37 | 0.38 | 0.37 | 0.01 | -0.05 |

| Q9DBG7 | SRPR | 69.62 | 0.11 | 0.26 | 0.18 | 0.11 | 0.23 | 0.35 | 0.29 | 0.08 | -0.10 |
| --- | --- | --- | --- | --- | --- | --- | --- | --- | --- | --- | --- |
| Q8BMA6 | SRP68 | 70.57 | 0.01 | 0.02 | 0.01 | 0.00 | -0.01 | 0.03 | 0.01 | 0.03 | 0.00 |
| P14576 | SRP54 | 55.72 | -0.24 | -0.03 | -0.14 | 0.15 | -0.14 | -0.05 | -0.09 | 0.06 | -0.04 |
| P16254 | SRP14 | 12.51 | 0.27 | 0.40 | 0.33 | 0.09 | 0.03 | 0.24 | 0.13 | 0.15 | 0.20 |
| Q64674 | SRM | 34.00 | -0.17 | -0.22 | -0.20 | 0.03 | -0.22 | -0.23 | -0.23 | 0.01 | 0.03 |
| Q9R112 | SQRDL | 50.28 | -0.99 | -0.84 | -0.92 | 0.10 | -0.19 | -0.05 | -0.12 | 0.10 | -0.80 |
| Q62261 | SPTBN1 | 274.22 | -0.15 | -0.19 | -0.17 | 0.03 | 0.04 | -0.01 | 0.01 | 0.03 | -0.18 |
| P15508 | SPTB | 245.25 | 0.44 | 0.19 | 0.31 | 0.18 | 0.18 | 0.10 | 0.14 | 0.06 | 0.17 |
| P16546 | SPTAN1 | 284.59 | -0.23 | -0.06 | -0.15 | 0.12 | 0.07 | 0.10 | 0.09 | 0.03 | -0.23 |
| P08032 | SPTA1 | 279.86 | 0.58 | 0.23 | 0.40 | 0.24 | 0.11 | 0.18 | 0.14 | 0.05 | 0.26 |
| Q64105 | SPR | 27.88 | -0.14 | -0.13 | -0.14 | 0.01 | -0.13 | -0.14 | -0.13 | 0.01 | 0.00 |
| Q3ULF4 | SPG7 | 86.00 | 0.21 | 0.19 | 0.20 | 0.01 | 0.23 | 0.12 | 0.17 | 0.08 | 0.03 |
| Q9CYN2 | SPCS2 | 24.98 | -0.10 | 0.02 | -0.04 | 0.09 | 0.38 | 0.10 | 0.24 | 0.20 | -0.27 |

| Q58A65 | SPAG9 | 146.22 | -0.35 | -0.18 | -0.27 | 0.12 | -0.22 | -0.13 | -0.18 | 0.06 | -0.09 |
| --- | --- | --- | --- | --- | --- | --- | --- | --- | --- | --- | --- |
| Q64442 | SORD | 38.25 | 0.04 | -0.08 | -0.02 | 0.08 | -0.19 | -0.21 | -0.20 | 0.02 | 0.18 |
| Q3UTJ2 | SORBS2 | 132.35 | 0.04 | 0.23 | 0.13 | 0.13 | 0.11 | 0.41 | 0.26 | 0.21 | -0.13 |
| Q9QX47 | SON | 265.65 | -0.46 | 0.04 | -0.21 | 0.36 | -0.02 | -0.02 | -0.02 | 0.00 | -0.19 |
| P09671 | SOD2 | 24.60 | 0.16 | 0.28 | 0.22 | 0.09 | 0.17 | 0.29 | 0.23 | 0.09 | -0.01 |
| P08228 | SOD1 | 15.94 | 0.11 | 0.19 | 0.15 | 0.06 | -0.01 | 0.06 | 0.03 | 0.05 | 0.12 |
| O88908 | SOAT2 | 60.60 | -0.52 | -0.43 | -0.47 | 0.06 | -0.38 | -0.39 | -0.39 | 0.01 | -0.08 |
| Q91VH2 | SNX9 | 66.55 | -0.05 | -0.11 | -0.08 | 0.04 | -0.01 | -0.12 | -0.06 | 0.08 | -0.02 |
| Q6P8X1 | SNX6 | 46.65 | -0.23 | -0.25 | -0.24 | 0.01 | -0.15 | -0.21 | -0.18 | 0.04 | -0.06 |
| Q9D8U8 | SNX5 | 46.80 | -0.19 | -0.12 | -0.15 | 0.05 | -0.04 | -0.04 | -0.04 | 0.00 | -0.11 |
| Q9CWK8 | SNX2 | 58.47 | -0.15 | -0.13 | -0.14 | 0.02 | 0.06 | -0.09 | -0.01 | 0.10 | -0.13 |
| Q9WV80 | SNX1 | 58.95 | -0.60 | -0.44 | -0.52 | 0.11 | -0.22 | -0.17 | -0.19 | 0.03 | -0.32 |
| Q9CSN1 | SNW1 | 61.48 | -0.12 | -0.15 | -0.14 | 0.02 | 0.05 | -0.11 | -0.03 | 0.11 | -0.10 |

| Q99L88 | SNTB1 | 58.08 | 0.25 | 0.22 | 0.23 | 0.02 | 0.23 | 0.23 | 0.23 | 0.00 | 0.00 |
| --- | --- | --- | --- | --- | --- | --- | --- | --- | --- | --- | --- |
| Q9CQI7 | SNRPB2 | 25.32 | -0.11 | -0.11 | -0.11 | 0.00 | 0.15 | 0.07 | 0.11 | 0.05 | -0.21 |
| P57784 | SNRPA1 | 28.36 | -0.12 | -0.07 | -0.10 | 0.03 | -0.06 | 0.00 | -0.03 | 0.05 | -0.07 |
| Q62376 | SNRNP70 | 51.99 | -0.21 | -0.04 | -0.13 | 0.12 | 0.07 | -0.21 | -0.07 | 0.20 | -0.06 |
| Q6P4T2 | SNRNP200 | 244.54 | 0.02 | -0.18 | -0.08 | 0.14 | -0.09 | -0.15 | -0.12 | 0.04 | 0.04 |
| Q9CZ28 | SNF8 | 28.89 | -0.19 | -0.10 | -0.14 | 0.07 | -0.35 | -0.12 | -0.24 | 0.16 | 0.09 |
| Q78PY7 | SND1 | 102.09 | 0.42 | 0.36 | 0.39 | 0.04 | 0.42 | 0.39 | 0.40 | 0.02 | -0.02 |
| Q8R570 | SNAP47 | 46.52 | -0.08 | 0.14 | 0.03 | 0.15 | 0.04 | 0.06 | 0.05 | 0.01 | -0.02 |
| Q9ERB0 | SNAP29 | 29.57 | -0.49 | -0.56 | -0.52 | 0.05 | -0.68 | -0.36 | -0.52 | 0.22 | 0.00 |
| O09044 | SNAP23 | 23.26 | -0.16 | -0.10 | -0.13 | 0.04 | -0.02 | 0.08 | 0.03 | 0.07 | -0.16 |
| Q9CW03 | SMC3 | 141.55 | -0.37 | -0.22 | -0.30 | 0.11 | 0.02 | -0.12 | -0.05 | 0.10 | -0.25 |
| Q9CU62 | SMC1A | 143.23 | -0.29 | -0.16 | -0.23 | 0.09 | -0.57 | -0.12 | -0.34 | 0.32 | 0.11 |
| O54941 | SMARCE1 | 46.64 | 0.03 | -0.16 | -0.06 | 0.13 | -0.08 | -0.13 | -0.11 | 0.04 | 0.04 |

| Q6PDG5 | SMARCC2 | 132.60 | -0.28 | -0.19 | -0.23 | 0.07 | -0.33 | -0.12 | -0.23 | 0.15 | -0.01 |
| --- | --- | --- | --- | --- | --- | --- | --- | --- | --- | --- | --- |
| Q91ZW3 | SMARCA5 | 121.63 | -0.37 | -0.37 | -0.37 | 0.00 | -0.17 | -0.22 | -0.19 | 0.04 | -0.18 |
| Q6DIC0 | SMARCA2 | 180.25 | -0.12 | 0.13 | 0.00 | 0.17 | -0.07 | -0.06 | -0.07 | 0.01 | 0.07 |
| Q7TN29 | SMAP2 | 46.58 | -0.17 | -0.10 | -0.14 | 0.05 | -0.39 | -0.07 | -0.23 | 0.22 | 0.09 |
| Q9R0P4 | SMAP | 20.05 | -0.53 | -0.09 | -0.31 | 0.32 | -0.98 | -0.11 | -0.55 | 0.62 | 0.24 |
| Q8CH25 | SLTM | 116.92 | -0.13 | 0.03 | -0.05 | 0.12 | 0.11 | 0.16 | 0.14 | 0.03 | -0.19 |
| Q3URD3 | SLMAP | 96.93 | -0.06 | -0.12 | -0.09 | 0.04 | -0.20 | -0.11 | -0.15 | 0.06 | 0.06 |
| O54988 | SLK | 141.46 | -0.40 | -0.18 | -0.29 | 0.15 | -0.16 | -0.15 | -0.15 | 0.01 | -0.14 |
| Q8BXB6 | SLCO2B1 | 74.53 | 0.22 | -0.26 | -0.02 | 0.33 | -0.42 | -0.51 | -0.46 | 0.06 | 0.44 |
| Q9JJL3 | SLCO1B2 | 76.73 | 0.55 | 0.25 | 0.40 | 0.21 | 0.39 | 0.21 | 0.30 | 0.13 | 0.10 |
| P70441 | SLC9A3R1 | 38.60 | 0.01 | 0.18 | 0.09 | 0.12 | 0.10 | 0.22 | 0.16 | 0.09 | -0.07 |
| O88343 | SLC4A4 | 121.48 | 0.60 | 0.31 | 0.45 | 0.21 | 0.50 | 0.20 | 0.35 | 0.21 | 0.11 |
| P04919 | SLC4A1 | 103.13 | 0.27 | 0.28 | 0.27 | 0.01 | 0.29 | 0.23 | 0.26 | 0.04 | 0.01 |

| P10852 | SLC3A2 | 58.34 | -0.04 | -0.31 | -0.17 | 0.20 | -0.01 | -0.30 | -0.15 | 0.21 | -0.02 |
| --- | --- | --- | --- | --- | --- | --- | --- | --- | --- | --- | --- |
| Q8R1S9 | SLC38A4 | 60.46 | 1.55 | 1.70 | 1.63 | 0.10 | 1.61 | 1.80 | 1.71 | 0.14 | -0.08 |
| Q99J27 | SLC33A1 | 61.08 | -0.02 | -0.04 | -0.03 | 0.02 | -0.40 | -0.04 | -0.22 | 0.26 | 0.19 |
| P14246 | SLC2A2 | 57.11 | -0.32 | -0.58 | -0.45 | 0.18 | -0.13 | -0.30 | -0.21 | 0.12 | -0.24 |
| Q4LDG0 | SLC27A5 | 76.20 | 0.05 | 0.01 | 0.03 | 0.03 | 0.06 | 0.09 | 0.08 | 0.02 | -0.05 |
| O35488 | SLC27A2 | 70.42 | 0.01 | -0.07 | -0.03 | 0.06 | -0.16 | -0.24 | -0.20 | 0.06 | 0.17 |
| P58735 | SLC26A1 | 75.79 | -0.29 | -0.41 | -0.35 | 0.09 | 0.09 | -0.03 | 0.03 | 0.09 | -0.38 |
| P51881 | SLC25A5 | 32.93 | -0.01 | 0.03 | 0.01 | 0.02 | -0.01 | 0.02 | 0.00 | 0.02 | 0.01 |
| Q8R0Y8 | SLC25A42 | 35.24 | 0.55 | 0.52 | 0.53 | 0.02 | 0.52 | 0.39 | 0.46 | 0.09 | 0.08 |
| P48962 | SLC25A4 | 32.90 | -2.47 | -2.59 | -2.53 | 0.09 | -2.37 | -2.62 | -2.50 | 0.17 | -0.04 |
| Q8VEM8 | SLC25A3 | 39.63 | -0.04 | -0.04 | -0.04 | 0.00 | -0.04 | 0.01 | -0.01 | 0.04 | -0.03 |
| Q6GQS1 | SLC25A23 | 52.50 | 0.43 | 0.33 | 0.38 | 0.07 | 0.45 | 0.33 | 0.39 | 0.08 | -0.01 |
| Q9D6M3 | SLC25A22 | 34.67 | 0.14 | 0.03 | 0.09 | 0.08 | 0.15 | 0.21 | 0.18 | 0.04 | -0.09 |

| Q8BZ09 | SLC25A21 | 33.23 | 0.10 | 0.09 | 0.10 | 0.00 | 0.10 | 0.15 | 0.13 | 0.03 | -0.03 |
| --- | --- | --- | --- | --- | --- | --- | --- | --- | --- | --- | --- |
| Q9Z2Z6 | SLC25A20 | 33.03 | 0.39 | 0.33 | 0.36 | 0.05 | 0.08 | 0.19 | 0.14 | 0.08 | 0.23 |
| Q9WVD5 | SLC25A15 | 32.82 | 0.29 | 0.35 | 0.32 | 0.04 | 0.39 | 0.47 | 0.43 | 0.06 | -0.12 |
| Q9QXX4 | SLC25A13 | 74.47 | 0.44 | 0.38 | 0.41 | 0.04 | 0.48 | 0.38 | 0.43 | 0.07 | -0.02 |
| Q8BH59 | SLC25A12 | 74.57 | -0.20 | -0.24 | -0.22 | 0.03 | 0.11 | -0.05 | 0.03 | 0.11 | -0.25 |
| Q9CR62 | SLC25A11 | 34.16 | -0.37 | -0.26 | -0.31 | 0.08 | -0.32 | -0.25 | -0.28 | 0.05 | -0.03 |
| Q9QZD8 | SLC25A10 | 31.72 | 0.46 | 0.54 | 0.50 | 0.06 | 0.36 | 0.43 | 0.39 | 0.04 | 0.11 |
| Q8JZU2 | SLC25A1 | 33.93 | 0.34 | 0.39 | 0.37 | 0.04 | 0.28 | 0.41 | 0.35 | 0.09 | 0.02 |
| Q78KK3 | SLC22A18 | 43.01 | 0.39 | 0.22 | 0.30 | 0.12 | 0.32 | 0.45 | 0.38 | 0.09 | -0.08 |
| O08966 | SLC22A1 | 61.52 | -0.19 | -0.29 | -0.24 | 0.08 | -0.27 | -0.44 | -0.35 | 0.12 | 0.11 |
| O70451 | SLC16A7 | 52.60 | -0.78 | -0.48 | -0.63 | 0.21 | -0.61 | -0.86 | -0.74 | 0.18 | 0.10 |
| P53986 | SLC16A1 | 53.27 | -0.20 | -0.15 | -0.17 | 0.03 | -0.25 | -0.24 | -0.25 | 0.01 | 0.07 |
| Q9WVL3 | SLC12A7 | 119.48 | -0.08 | 0.02 | -0.03 | 0.08 | -0.16 | -0.05 | -0.11 | 0.08 | 0.08 |

| O08705 | SLC10A1 | 39.41 | -0.44 | -0.58 | -0.51 | 0.10 | -0.46 | -0.44 | -0.45 | 0.01 | -0.06 |
| --- | --- | --- | --- | --- | --- | --- | --- | --- | --- | --- | --- |
| Q9WTX5 | SKP1 | 18.67 | 0.03 | 0.03 | 0.03 | 0.00 | -0.03 | 0.02 | 0.00 | 0.03 | 0.03 |
| Q9CZU3 | SKIV2L2 | 117.64 | -0.25 | -0.35 | -0.30 | 0.07 | -0.01 | -0.21 | -0.11 | 0.14 | -0.19 |
| Q8VDQ8 | SIRT2 | 43.26 | -0.35 | -0.25 | -0.30 | 0.07 | -0.35 | -0.25 | -0.30 | 0.07 | 0.00 |
| Q9D5J6 | SHPK | 51.30 | -0.28 | -0.30 | -0.29 | 0.01 | -0.37 | -0.37 | -0.37 | 0.00 | 0.08 |
| P50431 | SHMT1 | 52.60 | -0.74 | -0.75 | -0.74 | 0.01 | -0.74 | -0.73 | -0.73 | 0.01 | -0.01 |
| Q9JK48 | SH3GLB1 | 40.86 | -0.30 | -0.31 | -0.30 | 0.01 | -0.27 | -0.30 | -0.28 | 0.02 | -0.02 |
| Q62419 | SH3GL1 | 41.52 | -0.92 | -0.55 | -0.73 | 0.27 | -0.59 | -0.35 | -0.47 | 0.17 | -0.26 |
| Q8BJU0 | SGTA | 34.32 | -0.49 | -0.35 | -0.42 | 0.10 | -0.54 | -0.41 | -0.48 | 0.09 | 0.06 |
| Q8R0X7 | SGPL1 | 63.68 | -0.28 | -0.15 | -0.21 | 0.09 | -0.12 | -0.19 | -0.15 | 0.05 | -0.06 |
| Q9QZS5 | SGK2 | 41.36 | 0.50 | 0.74 | 0.62 | 0.17 | 0.60 | 0.58 | 0.59 | 0.02 | 0.02 |
| Q925N0 | SFXN5 | 37.33 | 0.49 | 0.39 | 0.44 | 0.07 | 0.24 | 0.18 | 0.21 | 0.04 | 0.23 |
| Q925N2 | SFXN2 | 36.14 | -0.06 | 0.00 | -0.03 | 0.04 | -0.08 | -0.17 | -0.13 | 0.07 | 0.09 |

| Q99JR1 | SFXN1 | 35.65 | 0.64 | 0.64 | 0.64 | 0.00 | 0.63 | 0.62 | 0.62 | 0.01 | 0.02 |
| --- | --- | --- | --- | --- | --- | --- | --- | --- | --- | --- | --- |
| Q8VIJ6 | SFPQ | 75.44 | -0.22 | -0.14 | -0.18 | 0.06 | -0.20 | -0.03 | -0.12 | 0.12 | -0.06 |
| Q921M3 | SF3B3 | 135.55 | -0.01 | -0.21 | -0.11 | 0.14 | 0.07 | -0.08 | -0.01 | 0.11 | -0.11 |
| Q99NB9 | SF3B1 | 145.81 | -0.44 | -0.07 | -0.25 | 0.27 | -0.58 | 0.00 | -0.29 | 0.41 | 0.04 |
| Q9D554 | SF3A3 | 58.84 | 0.01 | -0.01 | 0.00 | 0.01 | 0.00 | 0.01 | 0.01 | 0.00 | -0.01 |
| Q8K4Z5 | SF3A1 | 88.54 | -0.19 | 0.01 | -0.09 | 0.14 | -0.14 | 0.08 | -0.03 | 0.15 | -0.06 |
| Q64213 | SF1 | 70.40 | -0.25 | -0.04 | -0.15 | 0.15 | -0.11 | 0.04 | -0.04 | 0.11 | -0.11 |
| Q9EQU5 | SET | 33.38 | 0.71 | 0.25 | 0.48 | 0.33 | 1.08 | 0.27 | 0.68 | 0.57 | -0.20 |
| P19324 | SERPINH1 | 46.53 | -1.60 | -1.75 | -1.68 | 0.11 | -1.58 | -1.82 | -1.70 | 0.17 | 0.02 |
| P97290 | SERPING1 | 55.58 | 0.20 | 0.23 | 0.22 | 0.02 | 0.17 | 0.14 | 0.15 | 0.02 | 0.07 |
| Q61247 | SERPINF2 | 54.97 | 0.05 | -0.04 | 0.01 | 0.06 | -0.14 | -0.09 | -0.12 | 0.03 | 0.12 |
| P32261 | SERPINC1 | 52.00 | -0.08 | -0.03 | -0.05 | 0.04 | -0.29 | -0.11 | -0.20 | 0.12 | 0.15 |
| Q60854 | SERPINB6 | 42.60 | -0.41 | -0.07 | -0.24 | 0.24 | -0.09 | -0.08 | -0.09 | 0.00 | -0.15 |

| Q9D154 | SERPINB1A | 42.57 | 2.01 | 1.93 | 1.97 | 0.05 | 2.16 | 2.07 | 2.12 | 0.06 | -0.15 |
| --- | --- | --- | --- | --- | --- | --- | --- | --- | --- | --- | --- |
| Q06770 | SERPINA6 | 44.77 | -1.01 | -1.36 | -1.19 | 0.25 | -1.26 | -1.38 | -1.32 | 0.09 | 0.14 |
| Q91WP6 | SERPINA3N | 46.72 | 0.71 | 0.90 | 0.80 | 0.13 | 0.84 | 1.04 | 0.94 | 0.14 | -0.14 |
| Q03734 | SERPINA3M | 47.06 | 1.88 | -0.22 | 0.83 | 1.48 | 0.88 | -0.57 | 0.15 | 1.03 | 0.68 |
| P07759 | SERPINA3K | 46.88 | 1.81 | 1.84 | 1.82 | 0.03 | 1.08 | 1.36 | 1.22 | 0.20 | 0.60 |
| P22599 | SERPINA1B | 45.97 | 0.89 | 0.74 | 0.82 | 0.11 | 0.53 | 0.54 | 0.53 | 0.01 | 0.28 |
| Q8R121 | SERPINA10 | 51.80 | 0.99 | 0.47 | 0.73 | 0.37 | 0.19 | 0.27 | 0.23 | 0.06 | 0.50 |
| Q9EPB5 | SERHL | 35.31 | -0.14 | -0.05 | -0.09 | 0.06 | -0.53 | -0.27 | -0.40 | 0.18 | 0.31 |
| Q9CY58 | SERBP1 | 44.71 | -0.37 | -0.14 | -0.25 | 0.16 | -0.09 | -0.10 | -0.10 | 0.00 | -0.16 |
| Q6P6M7 | SEPSECS | 55.33 | -0.97 | -0.89 | -0.93 | 0.06 | -0.92 | -0.79 | -0.86 | 0.09 | -0.08 |
| P97364 | SEPHS2 | 47.83 | 0.35 | 0.38 | 0.36 | 0.03 | 0.35 | 0.43 | 0.39 | 0.06 | -0.03 |
| Q8BH69 | SEPHS1 | 42.91 | 0.06 | 0.00 | 0.03 | 0.04 | -0.02 | 0.11 | 0.04 | 0.09 | -0.01 |
| Q9DBC0 | SELO | 74.22 | 0.69 | 0.56 | 0.63 | 0.09 | 0.68 | 0.63 | 0.66 | 0.03 | -0.03 |

| Q63836 | SELENBP2 | 52.61 | 1.42 | 1.39 | 1.41 | 0.02 | 1.08 | 1.18 | 1.13 | 0.07 | 0.28 |
| --- | --- | --- | --- | --- | --- | --- | --- | --- | --- | --- | --- |
| P17563 | SELENBP1 | 52.51 | -0.06 | 0.01 | -0.03 | 0.04 | 0.08 | 0.06 | 0.07 | 0.01 | -0.09 |
| Q9Z2G6 | SEL1L | 88.34 | -0.08 | -0.23 | -0.16 | 0.11 | -0.15 | -0.31 | -0.23 | 0.11 | 0.07 |
| Q8VHE0 | SEC63 | 87.87 | 0.41 | 0.42 | 0.41 | 0.00 | 0.36 | 0.39 | 0.38 | 0.02 | 0.04 |
| Q8BU14 | SEC62 | 45.58 | -0.15 | -0.15 | -0.15 | 0.00 | -0.19 | -0.26 | -0.23 | 0.05 | 0.08 |
| Q9CQS8 | SEC61B | 9.96 | 0.01 | -0.51 | -0.25 | 0.37 | -0.26 | -0.32 | -0.29 | 0.04 | 0.04 |
|  |  |  | -0.01 | -0.12 | -0.07 | 0.07 | -0.01 | 0.08 | 0.03 | 0.07 | -0.10 |
| Q3UPL0 | SEC31A | 133.57 | -0.26 | -0.03 | -0.14 | 0.16 | -0.24 | 0.04 | -0.10 | 0.20 | -0.04 |
| Q3U2P1 | SEC24A | 118.78 | 0.18 | 0.12 | 0.15 | 0.04 | 0.42 | 0.13 | 0.27 | 0.21 | -0.12 |
| Q6NZC7 | SEC23IP | 110.78 | -0.08 | 0.13 | 0.03 | 0.15 | -0.29 | 0.24 | -0.02 | 0.38 | 0.05 |
| Q9D662 | SEC23B | 86.44 | -0.12 | 0.06 | -0.03 | 0.13 | -0.11 | -0.22 | -0.16 | 0.08 | 0.13 |
| Q01405 | SEC23A | 86.16 | 0.28 | 0.04 | 0.16 | 0.16 | 0.27 | 0.18 | 0.22 | 0.06 | -0.06 |
| Q8R0F9 | SEC14L4 | 46.05 | 0.42 | 0.49 | 0.46 | 0.05 | 0.23 | 0.32 | 0.28 | 0.06 | 0.18 |

| Q99J08 | SEC14L2 | 46.30 | -0.10 | -0.10 | -0.10 | 0.00 | -0.23 | -0.19 | -0.21 | 0.03 | 0.11 |
| --- | --- | --- | --- | --- | --- | --- | --- | --- | --- | --- | --- |
| Q9D1M0 | SEC13 | 35.57 | 0.34 | 0.37 | 0.36 | 0.02 | 0.44 | 0.26 | 0.35 | 0.13 | 0.00 |
| Q8VBT2 | SDS | 34.59 | 0.76 | 0.67 | 0.71 | 0.06 | 0.79 | 0.85 | 0.82 | 0.04 | -0.11 |
| Q9CZB0 | SDHC | 18.38 | 0.10 | 0.08 | 0.09 | 0.01 | -0.06 | 0.18 | 0.06 | 0.17 | 0.04 |
| Q9CQA3 | SDHB | 31.81 | 0.01 | 0.01 | 0.01 | 0.00 | 0.17 | 0.12 | 0.14 | 0.04 | -0.13 |
| Q8K2B3 | SDHA | 72.59 | 0.20 | 0.06 | 0.13 | 0.10 | 0.30 | 0.16 | 0.23 | 0.10 | -0.10 |
| O35988 | SDC4 | 21.48 | 0.34 | 0.77 | 0.55 | 0.30 | 0.64 | 0.66 | 0.65 | 0.02 | -0.10 |
| Q3TMH2 | SCRN3 | 47.66 | 0.32 | 0.24 | 0.28 | 0.06 | 0.24 | 0.21 | 0.23 | 0.03 | 0.05 |
| Q920A5 | SCPEP1 | 50.96 | 1.02 | 1.06 | 1.04 | 0.03 | 0.90 | 1.08 | 0.99 | 0.12 | 0.05 |
| P32020 | SCP2 | 59.13 | 2.07 | 1.13 | 1.60 | 0.66 | 1.77 | 0.98 | 1.37 | 0.56 | 0.23 |
| Q9JLI6 | SCLY | 47.17 | -0.43 | -0.48 | -0.45 | 0.04 | -0.41 | -0.42 | -0.42 | 0.01 | -0.04 |
| Q8BRF7 | SCFD1 | 72.32 | -0.40 | -0.12 | -0.26 | 0.20 | -0.20 | -0.07 | -0.14 | 0.09 | -0.12 |
| P13516 | SCD1 | 41.05 | -0.72 | -0.64 | -0.68 | 0.06 | -0.51 | -0.46 | -0.48 | 0.03 | -0.20 |

| O35114 | SCARB2 | 54.04 | 1.00 | 0.92 | 0.96 | 0.05 | 0.73 | 0.71 | 0.72 | 0.02 | 0.24 |
| --- | --- | --- | --- | --- | --- | --- | --- | --- | --- | --- | --- |
| Q61009 | SCARB1 | 56.75 | -0.19 | -0.33 | -0.26 | 0.10 | -0.10 | -0.09 | -0.09 | 0.01 | -0.17 |
| Q8K021 | SCAMP1 | 38.03 | 0.29 | 0.38 | 0.34 | 0.06 | 0.22 | 0.38 | 0.30 | 0.11 | 0.04 |
| P70122 | SBDS | 28.78 | 0.10 | 0.09 | 0.10 | 0.01 | 0.13 | 0.18 | 0.16 | 0.04 | -0.06 |
| Q8K190 | SAYSD1 | 20.70 | -0.16 | 0.05 | -0.06 | 0.15 | -0.19 | 0.06 | -0.06 | 0.18 | 0.01 |
| Q9JLI8 | SART3 | 109.62 | -0.19 | -0.13 | -0.16 | 0.04 | -0.05 | -0.15 | -0.10 | 0.07 | -0.06 |
| P26638 | SARS | 58.39 | -0.02 | -0.07 | -0.04 | 0.04 | 0.02 | -0.01 | 0.00 | 0.02 | -0.05 |
| Q9D1J3 | SARNP | 23.53 | -0.35 | -0.25 | -0.30 | 0.07 | -0.18 | -0.10 | -0.14 | 0.06 | -0.16 |
| Q99LB7 | SARDH | 101.68 | 0.29 | 0.22 | 0.26 | 0.05 | 0.22 | 0.23 | 0.22 | 0.01 | 0.03 |
| Q9CQC9 | SAR1B | 22.38 | 0.05 | -0.04 | 0.00 | 0.06 | -0.10 | 0.00 | -0.05 | 0.07 | 0.06 |
| P36536 | SAR1A | 22.37 | -0.06 | 0.03 | -0.01 | 0.07 | -0.03 | 0.01 | -0.01 | 0.03 | 0.00 |
| Q8BGH2 | SAMM50 | 51.86 | 0.02 | 0.11 | 0.07 | 0.06 | -0.16 | 0.02 | -0.07 | 0.13 | 0.14 |
| Q60710 | SAMHD1 | 72.65 | -0.29 | -0.34 | -0.31 | 0.04 | -0.15 | -0.25 | -0.20 | 0.07 | -0.12 |

| Q80YR5 | SAFB2 | 111.84 | -0.41 | 0.00 | -0.21 | 0.29 | -0.27 | 0.04 | -0.12 | 0.22 | -0.09 |
| --- | --- | --- | --- | --- | --- | --- | --- | --- | --- | --- | --- |
| D3YXK2 | SAFB | 105.10 | -0.30 | -0.08 | -0.19 | 0.16 | -0.05 | 0.01 | -0.02 | 0.04 | -0.17 |
| Q9EP69 | SACM1L | 66.94 | 0.07 | 0.08 | 0.07 | 0.00 | 0.00 | 0.03 | 0.01 | 0.03 | 0.06 |
| P97352 | S100A13 | 11.16 | 0.12 | -0.23 | -0.06 | 0.25 | -0.31 | -0.21 | -0.26 | 0.07 | 0.21 |
| Q9CQK7 | RWDD1 | 27.79 | -0.01 | -0.02 | -0.02 | 0.01 | -0.14 | -0.04 | -0.09 | 0.07 | 0.07 |
| Q9WTM5 | RUVBL2 | 51.11 | -0.02 | -0.11 | -0.07 | 0.07 | 0.08 | -0.02 | 0.03 | 0.07 | -0.09 |
| P60122 | RUVBL1 | 50.21 | -0.12 | -0.19 | -0.15 | 0.05 | -0.11 | -0.09 | -0.10 | 0.01 | -0.06 |
| Q8BIJ7 | RUFY1 | 80.38 | 0.06 | -0.02 | 0.02 | 0.06 | 0.09 | 0.14 | 0.12 | 0.03 | -0.10 |
| Q924D0 | RTN4IP1 | 43.37 | 0.09 | 0.12 | 0.10 | 0.02 | -0.02 | 0.11 | 0.05 | 0.09 | 0.05 |
| Q99P72 | RTN4 | 126.61 | -1.27 | -0.65 | -0.96 | 0.43 | -0.58 | -0.69 | -0.63 | 0.07 | -0.32 |
| Q9ES97 | RTN3 | 103.88 | -0.24 | -0.02 | -0.13 | 0.15 | -0.25 | -0.05 | -0.15 | 0.14 | 0.02 |
| Q99K95 | RTFDC1 | 33.93 | 0.59 | 0.46 | 0.53 | 0.09 | 0.76 | 0.27 | 0.51 | 0.34 | 0.01 |
| Q99LF4 | RTCB | 55.25 | 0.00 | 0.09 | 0.04 | 0.06 | 0.06 | 0.12 | 0.09 | 0.04 | -0.05 |

| Q01730 | RSU1 | 31.55 | 0.19 | 0.23 | 0.21 | 0.03 | 0.25 | 0.19 | 0.22 | 0.04 | -0.01 |
| --- | --- | --- | --- | --- | --- | --- | --- | --- | --- | --- | --- |
| Q8BVY0 | RSL1D1 | 50.42 | -0.65 | -0.49 | -0.57 | 0.11 | -0.46 | -0.31 | -0.39 | 0.11 | -0.19 |
| P07742 | RRM1 | 90.21 | -1.06 | -1.07 | -1.07 | 0.01 | -0.83 | -1.01 | -0.92 | 0.12 | -0.15 |
| Q99PL5 | RRBP1 | 172.88 | -0.24 | -0.22 | -0.23 | 0.02 | -0.11 | -0.18 | -0.15 | 0.04 | -0.08 |
| P10833 | RRAS | 23.76 | 0.44 | 0.19 | 0.32 | 0.18 | 0.47 | 0.41 | 0.44 | 0.04 | -0.13 |
| P14206 | RPSA | 32.84 | 0.34 | -0.05 | 0.15 | 0.28 | 0.15 | 0.13 | 0.14 | 0.02 | 0.01 |
| Q6ZWN5 | RPS9 | 22.59 | 0.01 | 0.09 | 0.05 | 0.06 | 0.04 | 0.02 | 0.03 | 0.01 | 0.02 |
| P62242 | RPS8 | 24.21 | 0.04 | 0.12 | 0.08 | 0.05 | 0.09 | 0.13 | 0.11 | 0.03 | -0.03 |
| P62082 | RPS7 | 22.13 | 0.06 | 0.08 | 0.07 | 0.01 | 0.08 | 0.11 | 0.10 | 0.02 | -0.03 |
| P18653 | RPS6KA1 | 81.59 | -1.08 | -0.96 | -1.02 | 0.09 | -0.85 | -0.81 | -0.83 | 0.03 | -0.19 |
| P62754 | RPS6 | 28.68 | 0.25 | 0.13 | 0.19 | 0.09 | 0.10 | 0.07 | 0.08 | 0.02 | 0.11 |
| P97461 | RPS5 | 22.89 | 0.06 | 0.01 | 0.03 | 0.04 | 0.07 | 0.06 | 0.07 | 0.01 | -0.03 |
| P62702 | RPS4X | 29.60 | 0.02 | 0.04 | 0.03 | 0.02 | 0.02 | 0.06 | 0.04 | 0.03 | -0.01 |

| P97351 | RPS3A | 29.89 | 0.06 | 0.08 | 0.07 | 0.02 | 0.08 | 0.07 | 0.07 | 0.00 | -0.01 |
| --- | --- | --- | --- | --- | --- | --- | --- | --- | --- | --- | --- |
| P62908 | RPS3 | 26.67 | 0.04 | 0.06 | 0.05 | 0.01 | 0.09 | 0.06 | 0.07 | 0.02 | -0.02 |
| Q6ZWY3 | RPS27L | 9.48 | 0.13 | 0.34 | 0.23 | 0.15 | 0.05 | 0.25 | 0.15 | 0.14 | 0.08 |
| P62852 | RPS25 | 13.74 | -0.03 | 0.03 | 0.00 | 0.05 | -0.03 | 0.03 | 0.00 | 0.04 | -0.01 |
| P62849 | RPS24 | 15.42 | -0.01 | 0.03 | 0.01 | 0.03 | -0.05 | 0.01 | -0.02 | 0.04 | 0.03 |
| P62267 | RPS23 | 15.81 | 0.03 | 0.00 | 0.02 | 0.02 | 0.00 | 0.05 | 0.02 | 0.03 | -0.01 |
| Q9CQR2 | RPS21 | 9.14 | 0.33 | 0.11 | 0.22 | 0.16 | 0.07 | -0.07 | 0.00 | 0.10 | 0.22 |
| P60867 | RPS20 | 13.37 | -0.05 | 0.11 | 0.03 | 0.12 | -0.07 | 0.09 | 0.01 | 0.11 | 0.02 |
| P25444 | RPS2 | 31.23 | 0.01 | 0.04 | 0.02 | 0.03 | 0.12 | 0.01 | 0.06 | 0.08 | -0.04 |
| Q9CZX8 | RPS19 | 16.09 | -0.06 | 0.12 | 0.03 | 0.13 | 0.00 | 0.08 | 0.04 | 0.05 | -0.01 |
| P62270 | RPS18 | 17.72 | -0.02 | 0.04 | 0.01 | 0.04 | 0.01 | 0.09 | 0.05 | 0.05 | -0.04 |
| P63276 | RPS17 | 15.52 | -0.01 | -0.01 | -0.01 | 0.00 | 0.00 | 0.06 | 0.03 | 0.05 | -0.04 |
| P14131 | RPS16 | 16.45 | 0.03 | 0.12 | 0.07 | 0.07 | 0.14 | 0.13 | 0.13 | 0.01 | -0.06 |

| P62245 | RPS15A | 14.84 | -0.03 | 0.03 | 0.00 | 0.05 | 0.12 | 0.06 | 0.09 | 0.04 | -0.09 |
| --- | --- | --- | --- | --- | --- | --- | --- | --- | --- | --- | --- |
| P62843 | RPS15 | 17.04 | -0.03 | 0.14 | 0.06 | 0.12 | -0.04 | 0.16 | 0.06 | 0.14 | 0.00 |
| P62264 | RPS14 | 16.27 | -0.02 | 0.09 | 0.03 | 0.08 | -0.02 | 0.08 | 0.03 | 0.07 | 0.00 |
| P62301 | RPS13 | 17.22 | 0.04 | 0.06 | 0.05 | 0.02 | -0.07 | 0.03 | -0.02 | 0.08 | 0.07 |
| P63323 | RPS12 | 14.53 | 0.03 | 0.07 | 0.05 | 0.02 | -0.04 | 0.09 | 0.02 | 0.09 | 0.03 |
| P62281 | RPS11 | 18.43 | -0.06 | 0.09 | 0.02 | 0.11 | -0.06 | 0.05 | -0.01 | 0.07 | 0.02 |
| P63325 | RPS10 | 18.92 | -0.12 | 0.02 | -0.05 | 0.10 | 0.01 | 0.03 | 0.02 | 0.01 | -0.07 |
| Q9CSU0 | RPRD1B | 36.88 | -0.41 | -0.51 | -0.46 | 0.07 | -0.33 | -0.44 | -0.39 | 0.07 | -0.07 |
| Q9DBG6 | RPN2 | 69.06 | 0.14 | 0.08 | 0.11 | 0.04 | 0.08 | 0.10 | 0.09 | 0.01 | 0.02 |
| Q91YQ5 | RPN1 | 68.53 | 0.11 | 0.08 | 0.09 | 0.02 | 0.23 | 0.10 | 0.16 | 0.09 | -0.07 |
| P99027 | RPLP2 | 11.65 | -0.10 | 0.03 | -0.03 | 0.09 | -0.04 | 0.06 | 0.01 | 0.08 | -0.05 |
| P47955 | RPLP1 | 11.48 | -0.09 | 0.01 | -0.04 | 0.07 | -0.07 | 0.01 | -0.03 | 0.06 | -0.01 |
| P14869 | RPLP0 | 34.22 | 0.11 | 0.06 | 0.09 | 0.03 | 0.12 | 0.06 | 0.09 | 0.04 | 0.00 |

| P51410 | RPL9 | 21.88 | 0.01 | 0.05 | 0.03 | 0.03 | 0.01 | 0.04 | 0.03 | 0.03 | 0.00 |
| --- | --- | --- | --- | --- | --- | --- | --- | --- | --- | --- | --- |
| P62918 | RPL8 | 28.02 | -0.01 | 0.08 | 0.03 | 0.06 | 0.04 | 0.04 | 0.04 | 0.00 | 0.00 |
| P12970 | RPL7A | 29.98 | 0.02 | 0.05 | 0.03 | 0.02 | 0.03 | 0.03 | 0.03 | 0.00 | 0.00 |
| P14148 | RPL7 | 31.42 | -0.05 | 0.03 | -0.01 | 0.05 | -0.02 | 0.00 | -0.01 | 0.01 | 0.00 |
| P47911 | RPL6 | 33.51 | 0.01 | 0.02 | 0.01 | 0.00 | 0.06 | 0.02 | 0.04 | 0.03 | -0.03 |
| P47962 | RPL5 | 34.40 | 0.04 | 0.03 | 0.03 | 0.01 | 0.12 | 0.06 | 0.09 | 0.04 | -0.06 |
| Q9D8E6 | RPL4 | 47.15 | 0.05 | -0.03 | 0.01 | 0.06 | 0.10 | 0.03 | 0.06 | 0.05 | -0.05 |
| P47964 | RPL36 | 12.22 | -0.05 | 0.01 | -0.02 | 0.05 | -0.07 | -0.13 | -0.10 | 0.04 | 0.08 |
| O55142 | RPL35A | 12.55 | -0.16 | 0.05 | -0.06 | 0.14 | -0.18 | -0.01 | -0.09 | 0.12 | 0.04 |
| Q6ZWV7 | RPL35 | 14.55 | -0.08 | -0.06 | -0.07 | 0.01 | -0.01 | -0.05 | -0.03 | 0.02 | -0.04 |
| Q9D1R9 | RPL34 | 13.29 | 0.01 | 0.00 | 0.00 | 0.00 | -0.15 | 0.06 | -0.04 | 0.16 | 0.05 |
| P62911 | RPL32 | 15.86 | 0.02 | 0.24 | 0.13 | 0.15 | -0.05 | -0.11 | -0.08 | 0.04 | 0.21 |
| P62900 | RPL31 | 14.46 | -0.01 | 0.07 | 0.03 | 0.05 | -0.02 | 0.03 | 0.01 | 0.04 | 0.02 |

| P62889 | RPL30 | 12.78 | 0.13 | 0.03 | 0.08 | 0.07 | -0.06 | 0.16 | 0.05 | 0.16 | 0.03 |
| --- | --- | --- | --- | --- | --- | --- | --- | --- | --- | --- | --- |
| P27659 | RPL3 | 46.11 | 0.03 | 0.00 | 0.02 | 0.02 | 0.05 | 0.05 | 0.05 | 0.00 | -0.03 |
| P47915 | RPL29 | 17.59 | -0.03 | 0.16 | 0.06 | 0.13 | 0.01 | 0.04 | 0.02 | 0.02 | 0.04 |
| P41105 | RPL28 | 15.73 | -0.14 | 0.04 | -0.05 | 0.12 | -0.11 | -0.08 | -0.10 | 0.02 | 0.04 |
| P14115 | RPL27A | 16.61 | 0.01 | 0.06 | 0.04 | 0.04 | 0.01 | 0.05 | 0.03 | 0.03 | 0.01 |
| P61358 | RPL27 | 15.80 | -0.09 | 0.03 | -0.03 | 0.09 | -0.04 | 0.01 | -0.01 | 0.03 | -0.02 |
| P61255 | RPL26 | 17.26 | -0.05 | 0.02 | -0.01 | 0.05 | -0.05 | 0.07 | 0.01 | 0.09 | -0.03 |
| Q8BP67 | RPL24 | 17.78 | -0.06 | 0.10 | 0.02 | 0.11 | -0.08 | 0.14 | 0.03 | 0.15 | -0.01 |
| P62751 | RPL23A | 17.70 | -0.09 | 0.00 | -0.04 | 0.06 | -0.02 | 0.03 | 0.01 | 0.03 | -0.05 |
| P62830 | RPL23 | 14.87 | -0.06 | -0.06 | -0.06 | 0.00 | -0.06 | 0.01 | -0.02 | 0.05 | -0.04 |
| P67984 | RPL22 | 14.76 | 0.03 | 0.09 | 0.06 | 0.04 | 0.10 | 0.14 | 0.12 | 0.03 | -0.06 |
| O09167 | RPL21 | 18.56 | -0.10 | 0.01 | -0.04 | 0.08 | -0.05 | 0.05 | 0.00 | 0.07 | -0.05 |
| P84099 | RPL19 | 23.47 | 0.09 | 0.25 | 0.17 | 0.11 | 0.15 | -0.02 | 0.06 | 0.12 | 0.10 |

| P35980 | RPL18 | 21.64 | -0.06 | -0.04 | -0.05 | 0.02 | 0.02 | -0.38 | -0.18 | 0.28 | 0.12 |
| --- | --- | --- | --- | --- | --- | --- | --- | --- | --- | --- | --- |
| Q9CPR4 | RPL17 | 21.42 | -0.05 | 0.10 | 0.02 | 0.10 | -0.03 | 0.12 | 0.05 | 0.11 | -0.02 |
| Q9CZM2 | RPL15 | 24.15 | 0.14 | 0.25 | 0.19 | 0.08 | 0.07 | -0.02 | 0.03 | 0.06 | 0.17 |
| Q9CR57 | RPL14 | 23.56 | -0.11 | 0.03 | -0.04 | 0.10 | -0.02 | 0.07 | 0.02 | 0.06 | -0.06 |
| P19253 | RPL13A | 23.46 | -0.05 | 0.03 | -0.01 | 0.06 | -0.03 | 0.04 | 0.00 | 0.05 | -0.01 |
| P47963 | RPL13 | 24.31 | -0.01 | 0.10 | 0.04 | 0.08 | 0.03 | 0.07 | 0.05 | 0.03 | -0.01 |
| P35979 | RPL12 | 17.80 | -0.08 | 0.02 | -0.03 | 0.07 | -0.02 | 0.05 | 0.02 | 0.05 | -0.05 |
| Q9CXW4 | RPL11 | 20.25 | 0.03 | 0.06 | 0.05 | 0.03 | 0.02 | 0.12 | 0.07 | 0.07 | -0.02 |
| P53026 | RPL10A | 24.92 | -0.01 | 0.03 | 0.01 | 0.03 | 0.07 | 0.07 | 0.07 | 0.00 | -0.06 |
|  |  | 24.60 | 0.00 | 0.11 | 0.06 | 0.08 | 0.03 | 0.18 | 0.11 | 0.10 | -0.05 |
| P70336 | ROCK2 | 160.58 | -0.15 | -0.04 | -0.10 | 0.08 | 0.04 | -0.03 | 0.01 | 0.05 | -0.11 |
| P70335 | ROCK1 | 158.17 | 0.08 | -0.06 | 0.01 | 0.10 | -0.11 | 0.03 | -0.04 | 0.10 | 0.06 |
| Q99M28 | RNPS1 | 34.21 | 0.18 | -0.05 | 0.07 | 0.16 | -0.05 | -0.25 | -0.15 | 0.14 | 0.22 |

| Q8VCT3 | RNPEP | 72.42 | 0.02 | -0.07 | -0.03 | 0.06 | -0.06 | -0.21 | -0.13 | 0.10 | 0.11 |
| --- | --- | --- | --- | --- | --- | --- | --- | --- | --- | --- | --- |
| Q9D0L8 | RNMT | 53.29 | 0.38 | 0.30 | 0.34 | 0.06 | 0.49 | 0.26 | 0.37 | 0.16 | -0.03 |
| Q91VI7 | RNH1 | 49.82 | 0.21 | 0.11 | 0.16 | 0.07 | -0.02 | 0.07 | 0.03 | 0.07 | 0.13 |
| E9Q555 | RNF213 | 584.49 | -0.30 | -0.93 | -0.61 | 0.44 | -0.57 | -0.81 | -0.69 | 0.17 | 0.07 |
| Q5DTM8 | RNF20 | 113.52 | -0.22 | -0.24 | -0.23 | 0.02 | -0.07 | -0.06 | -0.07 | 0.01 | -0.16 |
| Q3UJU9 | RMDN3 | 52.03 | 0.17 | 0.41 | 0.29 | 0.17 | -0.06 | 0.18 | 0.06 | 0.17 | 0.23 |
| Q8BSE0 | RMDN2 | 47.02 | 0.27 | 0.32 | 0.29 | 0.03 | 0.09 | 0.13 | 0.11 | 0.03 | 0.18 |
| Q9DCV4 | RMDN1 | 35.00 | -0.11 | -0.08 | -0.09 | 0.03 | -0.06 | -0.04 | -0.05 | 0.02 | -0.04 |
| Q8JZN7 | RHOT2 | 69.07 | 0.65 | 0.23 | 0.44 | 0.30 | -0.23 | 0.22 | -0.01 | 0.32 | 0.45 |
| Q8BG51 | RHOT1 | 72.24 | 0.22 | 0.08 | 0.15 | 0.10 | 0.18 | 0.15 | 0.16 | 0.03 | -0.02 |
| Q9QUI0 | RHOA | 21.78 | -0.02 | 5.12 | 2.55 | 3.63 | 0.00 | 3.26 | 1.63 | 2.30 | 0.92 |
| Q64374 | RGN | 33.41 | -0.07 | -0.15 | -0.11 | 0.06 | -0.18 | -0.25 | -0.22 | 0.05 | 0.10 |
| Q9D8S4 | REXO2 | 26.74 | 0.95 | 1.18 | 1.07 | 0.16 | 0.93 | 1.16 | 1.05 | 0.16 | 0.02 |

| Q64FW2 | RETSAT | 67.33 | 2.24 | 2.30 | 2.27 | 0.04 | 1.53 | 1.63 | 1.58 | 0.07 | 0.69 |
| --- | --- | --- | --- | --- | --- | --- | --- | --- | --- | --- | --- |
| Q9JM62 | REEP6 | 22.20 | 0.52 | 0.65 | 0.59 | 0.09 | 0.53 | 0.52 | 0.53 | 0.00 | 0.06 |
| P26043 | RDX | 68.54 | 0.00 | 0.08 | 0.04 | 0.05 | -0.03 | 0.17 | 0.07 | 0.14 | -0.03 |
| O88451 | RDH7 | 35.66 | 0.10 | 0.10 | 0.10 | 0.00 | 0.00 | 0.10 | 0.05 | 0.07 | 0.05 |
| Q8CEE7 | RDH13 | 36.46 | -0.24 | -0.11 | -0.17 | 0.09 | 0.02 | -0.12 | -0.05 | 0.10 | -0.12 |
| Q9QYF1 | RDH11 | 35.15 | -0.71 | -0.72 | -0.72 | 0.01 | -0.88 | -0.75 | -0.81 | 0.09 | 0.09 |
| Q8VE37 | RCC1 | 44.93 | -0.14 | 0.10 | -0.02 | 0.17 | 0.26 | 0.16 | 0.21 | 0.07 | -0.23 |
| Q00915 | RBP1 | 15.85 | -1.22 | -1.13 | -1.17 | 0.06 | -0.72 | -0.79 | -0.75 | 0.05 | -0.42 |
| Q91YE7 | RBM5 | 92.31 | -0.61 | -0.46 | -0.53 | 0.11 | -0.60 | -0.48 | -0.54 | 0.09 | 0.01 |
| Q8VH51 | RBM39 | 59.41 | -0.08 | -0.22 | -0.15 | 0.10 | 0.10 | -0.16 | -0.03 | 0.19 | -0.12 |
| Q8JZX4 | RBM17 | 45.30 | -0.26 | -0.28 | -0.27 | 0.01 | -0.13 | -0.11 | -0.12 | 0.02 | -0.15 |
| Q8C2Q3 | RBM14 | 69.45 | -0.66 | -0.15 | -0.41 | 0.36 | -0.75 | -0.16 | -0.46 | 0.42 | 0.05 |
| Q8R1Q9 | RBKS | 34.12 | 0.33 | 0.29 | 0.31 | 0.03 | 0.08 | 0.32 | 0.20 | 0.17 | 0.11 |

| Q60973 | RBBP7 | 47.79 | -0.33 | -0.28 | -0.31 | 0.04 | -0.25 | -0.27 | -0.26 | 0.01 | -0.05 |
| --- | --- | --- | --- | --- | --- | --- | --- | --- | --- | --- | --- |
| Q60972 | RBBP4 | 47.66 | -0.39 | -0.23 | -0.31 | 0.11 | 0.07 | 0.01 | 0.04 | 0.04 | -0.35 |
| Q9D0I9 | RARS | 75.67 | 0.13 | 0.05 | 0.09 | 0.05 | 0.13 | 0.06 | 0.09 | 0.05 | 0.00 |
| Q9EQZ6 | RAPGEF4 | 115.49 | 1.62 | 1.40 | 1.51 | 0.15 | 1.78 | 1.59 | 1.68 | 0.13 | -0.17 |
| P46061 | RANGAP1 | 63.53 | -0.32 | -0.36 | -0.34 | 0.02 | -0.06 | -0.22 | -0.14 | 0.12 | -0.20 |
| Q9ERU9 | RANBP2 | 341.12 | -0.36 | 0.08 | -0.14 | 0.31 | -0.19 | 0.07 | -0.06 | 0.19 | -0.08 |
| P34022 | RANBP1 | 23.60 | -0.18 | -0.20 | -0.19 | 0.02 | -0.11 | -0.19 | -0.15 | 0.06 | -0.03 |
| P62827 | RAN | 24.42 | -0.41 | -0.33 | -0.37 | 0.06 | -0.29 | -0.17 | -0.23 | 0.08 | -0.14 |
| Q64012 | RALY | 33.19 | -0.39 | -0.25 | -0.32 | 0.10 | -0.37 | -0.18 | -0.28 | 0.13 | -0.04 |
| Q9JIW9 | RALB | 23.35 | -0.59 | -0.39 | -0.49 | 0.14 | -0.43 | -0.37 | -0.40 | 0.04 | -0.09 |
| P63321 | RALA | 23.55 | -0.23 | -0.02 | -0.12 | 0.15 | -0.07 | -0.04 | -0.05 | 0.02 | -0.07 |
| Q9EP71 | RAI14 | 108.85 | 0.06 | 0.25 | 0.16 | 0.13 | 0.08 | 0.26 | 0.17 | 0.12 | -0.01 |
| P54728 | RAD23B | 43.51 | -0.36 | -0.13 | -0.25 | 0.16 | -0.47 | -0.17 | -0.32 | 0.22 | 0.07 |

| P54726 | RAD23A | 39.71 | -0.42 | -0.28 | -0.35 | 0.10 | -0.51 | -0.28 | -0.39 | 0.17 | 0.04 |
| --- | --- | --- | --- | --- | --- | --- | --- | --- | --- | --- | --- |
| Q61550 | RAD21 | 72.08 | -0.14 | -0.16 | -0.15 | 0.01 | -0.25 | -0.17 | -0.21 | 0.06 | 0.06 |
| P63001 | RAC1;RAC2 | 21.45 | -0.36 | -0.07 | -0.22 | 0.21 | -0.11 | 0.05 | -0.03 | 0.11 | -0.19 |
| Q9JHK4 | RABGGTA | 64.99 | -0.08 | -0.02 | -0.05 | 0.04 | -0.26 | -0.10 | -0.18 | 0.11 | 0.13 |
| O35551 | RABEP1 | 99.52 | 0.20 | -0.10 | 0.05 | 0.21 | -0.50 | -0.12 | -0.31 | 0.27 | 0.36 |
| Q9R0M6 | RAB9A | 22.91 | 0.37 | 0.44 | 0.40 | 0.05 | 0.27 | 0.35 | 0.31 | 0.06 | 0.09 |
| P55258 | RAB8A | 23.67 | -0.21 | -0.17 | -0.19 | 0.03 | -0.20 | -0.09 | -0.15 | 0.08 | -0.04 |
| P51150 | RAB7A | 23.49 | -0.29 | -0.23 | -0.26 | 0.04 | -0.20 | -0.14 | -0.17 | 0.04 | -0.09 |
| P35279 |  | 23.59 | -0.04 | -0.07 | -0.05 | 0.03 | -0.03 | -0.03 | -0.03 | 0.00 | -0.03 |
| P35278 | RAB5C | 23.41 | -0.28 | -0.18 | -0.23 | 0.07 | -0.26 | -0.22 | -0.24 | 0.03 | 0.00 |
| P61021 | RAB5B | 23.71 | -0.15 | -0.17 | -0.16 | 0.01 | -0.10 | -0.06 | -0.08 | 0.03 | -0.08 |
| Q9CQD1 | RAB5A | 23.60 | -0.11 | 0.05 | -0.03 | 0.11 | -0.17 | 0.00 | -0.08 | 0.12 | 0.05 |
| Q6PHN9 | RAB35 | 23.03 | 0.17 | -0.09 | 0.04 | 0.19 | 0.14 | 0.13 | 0.14 | 0.01 | -0.10 |

| Q9CZE3 | RAB32 | 25.07 | -0.24 | -0.32 | -0.28 | 0.05 | -0.08 | -0.09 | -0.08 | 0.00 | -0.19 |
| --- | --- | --- | --- | --- | --- | --- | --- | --- | --- | --- | --- |
| P53994 | RAB2A | 23.55 | 0.27 | -0.12 | 0.07 | 0.27 | 0.17 | -0.10 | 0.03 | 0.19 | 0.04 |
| P35282 | RAB21 | 24.11 | -0.12 | -0.15 | -0.13 | 0.02 | -0.08 | -0.20 | -0.14 | 0.09 | 0.01 |
| Q9D1G1 | RAB1B | 22.19 | -0.30 | -0.24 | -0.27 | 0.04 | -0.24 | -0.25 | -0.25 | 0.01 | -0.02 |
| P62821 | RAB1A | 22.68 | -0.14 | -0.03 | -0.08 | 0.08 | -0.13 | -0.10 | -0.11 | 0.02 | 0.03 |
| P35293 | RAB18 | 23.04 | 0.18 | 0.27 | 0.23 | 0.07 | 0.45 | 0.32 | 0.39 | 0.10 | -0.16 |
| P35292 | RAB17 | 23.64 | -0.06 | -0.05 | -0.06 | 0.01 | -0.12 | -0.06 | -0.09 | 0.04 | 0.04 |
| Q91V41 | RAB14 | 23.90 | -0.07 | -0.01 | -0.04 | 0.04 | 0.04 | 0.14 | 0.09 | 0.07 | -0.13 |
| P46638 | RAB11B | 24.49 | -0.09 | -0.05 | -0.07 | 0.03 | -0.03 | -0.02 | -0.03 | 0.01 | -0.05 |
| P61027 | RAB10 | 22.54 | 0.00 | 0.08 | 0.04 | 0.06 | 0.04 | 0.08 | 0.06 | 0.03 | -0.02 |
| Q91X91 | QPRT | 31.53 | -0.09 | -0.17 | -0.13 | 0.06 | -0.02 | -0.05 | -0.03 | 0.02 | -0.10 |
| Q8BVI4 | QDPR | 25.57 | 0.11 | 0.18 | 0.15 | 0.05 | 0.11 | 0.17 | 0.14 | 0.04 | 0.01 |
| Q9WUB3 | PYGM | 97.29 | -2.48 | -2.57 | -2.53 | 0.06 | -2.53 | -2.56 | -2.54 | 0.02 | 0.02 |

| Q9ET01 | PYGL | 97.46 | -0.88 | -0.89 | -0.88 | 0.00 | -0.91 | -0.84 | -0.87 | 0.05 | -0.01 |
| --- | --- | --- | --- | --- | --- | --- | --- | --- | --- | --- | --- |
| Q8CI94 | PYGB | 96.73 | -2.04 | -2.14 | -2.09 | 0.07 | -1.99 | -2.08 | -2.03 | 0.07 | -0.06 |
| P42925 | PXMP2 | 22.27 | -0.10 | -0.13 | -0.11 | 0.02 | -0.03 | -0.04 | -0.04 | 0.00 | -0.08 |
| Q9D3U0 | PUS10 | 59.71 | 0.05 | -0.12 | -0.03 | 0.12 | -0.09 | 0.01 | -0.04 | 0.07 | 0.01 |
| P42669 | PURA | 34.88 | 0.32 | 0.36 | 0.34 | 0.02 | 0.15 | 0.33 | 0.24 | 0.13 | 0.10 |
| Q3UEB3 | PUF60 | 60.25 | -0.31 | -0.15 | -0.23 | 0.11 | -0.46 | -0.12 | -0.29 | 0.24 | 0.06 |
| Q8R2Y8 | PTRH2 | 19.53 | 0.24 | 0.27 | 0.26 | 0.02 | 0.19 | 0.32 | 0.26 | 0.09 | 0.00 |
| O54724 | PTRF | 43.95 | -0.45 | -0.32 | -0.39 | 0.09 | 0.00 | 0.05 | 0.02 | 0.03 | -0.41 |
| Q64487 | PTPRD | 214.41 | -0.40 | -0.14 | -0.27 | 0.19 | -0.52 | -0.33 | -0.43 | 0.13 | 0.16 |
| P06800 | PTPRC | 144.60 | -0.37 | -0.18 | -0.27 | 0.13 | -0.61 | -0.32 | -0.46 | 0.20 | 0.19 |
| P29351 | PTPN6 | 67.56 | -0.13 | -0.10 | -0.11 | 0.02 | -0.07 | -0.23 | -0.15 | 0.11 | 0.03 |
| P35235 | PTPN11 | 68.46 | 0.12 | -0.01 | 0.06 | 0.10 | 0.21 | -0.10 | 0.05 | 0.22 | 0.00 |
| Q9D0J8 | PTMS | 11.43 | 0.10 | 0.10 | 0.10 | 0.01 | 0.21 | 0.19 | 0.20 | 0.01 | -0.10 |

| Q8VDQ1 | PTGR2 | 38.02 | -0.15 | -0.16 | -0.15 | 0.01 | -0.25 | -0.22 | -0.24 | 0.02 | 0.08 |
| --- | --- | --- | --- | --- | --- | --- | --- | --- | --- | --- | --- |
| Q91YR9 | PTGR1 | 35.56 | -0.76 | -0.79 | -0.77 | 0.02 | -0.43 | -0.64 | -0.53 | 0.15 | -0.24 |
| Q9R0Q7 | PTGES3 | 18.72 | -0.24 | 0.80 | 0.28 | 0.74 | -0.23 | -0.18 | -0.21 | 0.04 | 0.49 |
| Q8BWM0 | PTGES2 | 43.32 | -0.01 | 0.12 | 0.06 | 0.09 | 0.10 | 0.16 | 0.13 | 0.05 | -0.07 |
| Q60866 | PTER | 39.22 | -0.78 | -0.82 | -0.80 | 0.02 | -0.79 | -0.72 | -0.76 | 0.05 | -0.04 |
| Q14C51 | PTCD3 | 77.80 | 0.19 | 0.19 | 0.19 | 0.00 | 0.13 | 0.18 | 0.15 | 0.03 | 0.04 |
| Q8R3K3 | PTCD2 | 43.84 | -0.39 | -0.27 | -0.33 | 0.09 | -0.36 | -0.11 | -0.23 | 0.18 | -0.10 |
| Q8BHD7 | PTBP3 | 56.70 | -0.05 | 0.14 | 0.04 | 0.14 | 0.20 | 0.23 | 0.22 | 0.02 | -0.17 |
| P17225 | PTBP1 | 56.48 | -0.15 | -0.06 | -0.10 | 0.06 | -0.02 | 0.00 | -0.01 | 0.01 | -0.09 |
| Q8R326 | PSPC1 | 58.76 | -0.14 | -0.09 | -0.11 | 0.04 | 0.28 | 0.13 | 0.20 | 0.10 | -0.32 |
| Q5SSW2 | PSME4 | 211.19 | 0.32 | 0.17 | 0.24 | 0.10 | 0.12 | 0.12 | 0.12 | 0.00 | 0.12 |
| P61290 | PSME3 | 29.51 | 0.01 | 0.13 | 0.07 | 0.09 | 0.11 | 0.10 | 0.11 | 0.00 | -0.04 |
| P97372 | PSME2 | 27.06 | -0.40 | -0.43 | -0.42 | 0.02 | -0.43 | -0.43 | -0.43 | 0.00 | 0.01 |

| P97371 | PSME1 | 28.67 | -0.46 | -0.38 | -0.42 | 0.06 | -0.39 | -0.38 | -0.39 | 0.01 | -0.04 |
| --- | --- | --- | --- | --- | --- | --- | --- | --- | --- | --- | --- |
| Q9CR00 | PSMD9 | 24.72 | -0.02 | 0.02 | 0.00 | 0.03 | -0.05 | 0.04 | -0.01 | 0.06 | 0.00 |
| Q9CX56 | PSMD8 | 39.93 | -0.23 | -0.30 | -0.27 | 0.04 | -0.30 | -0.22 | -0.26 | 0.06 | 0.00 |
| P26516 | PSMD7 | 36.54 | -0.11 | -0.16 | -0.14 | 0.04 | -0.23 | -0.18 | -0.21 | 0.03 | 0.07 |
| Q99JI4 | PSMD6 | 45.54 | -0.15 | -0.14 | -0.14 | 0.01 | -0.23 | -0.17 | -0.20 | 0.04 | 0.06 |
| O35226 | PSMD4 | 40.70 | -0.51 | -0.23 | -0.37 | 0.20 | -0.41 | -0.18 | -0.29 | 0.16 | -0.08 |
| P14685 | PSMD3 | 60.72 | -0.31 | -0.25 | -0.28 | 0.04 | -0.32 | -0.27 | -0.29 | 0.03 | 0.01 |
| Q8VDM4 | PSMD2 | 100.20 | -0.19 | -0.21 | -0.20 | 0.01 | -0.20 | -0.22 | -0.21 | 0.02 | 0.01 |
| O35593 | PSMD14 | 34.58 | -0.28 | -0.15 | -0.22 | 0.09 | -0.33 | -0.27 | -0.30 | 0.05 | 0.08 |
| Q9WVJ2 | PSMD13 | 42.81 | -0.16 | -0.16 | -0.16 | 0.01 | -0.24 | -0.26 | -0.25 | 0.01 | 0.09 |
| Q9D8W5 | PSMD12 | 52.90 | -0.17 | -0.16 | -0.16 | 0.00 | -0.16 | -0.19 | -0.17 | 0.02 | 0.01 |
| Q8BG32 | PSMD11 | 47.44 | -0.11 | -0.12 | -0.11 | 0.00 | -0.25 | -0.17 | -0.21 | 0.06 | 0.10 |
| Q3TXS7 | PSMD1 | 105.73 | -0.18 | -0.21 | -0.19 | 0.02 | -0.18 | -0.23 | -0.21 | 0.03 | 0.01 |

| P62334 | PSMC6 | 44.17 | -0.23 | -0.24 | -0.24 | 0.01 | -0.34 | -0.19 | -0.27 | 0.11 | 0.03 |
| --- | --- | --- | --- | --- | --- | --- | --- | --- | --- | --- | --- |
| P62196 | PSMC5 | 45.63 | -0.24 | -0.25 | -0.25 | 0.01 | -0.31 | -0.28 | -0.29 | 0.02 | 0.05 |
| P54775 | PSMC4 | 47.41 | -0.28 | -0.24 | -0.26 | 0.03 | -0.21 | -0.19 | -0.20 | 0.02 | -0.07 |
| O88685 | PSMC3 | 49.55 | -0.24 | -0.18 | -0.21 | 0.04 | -0.15 | -0.19 | -0.17 | 0.03 | -0.04 |
| P46471 | PSMC2 | 48.65 | -0.11 | -0.25 | -0.18 | 0.11 | -0.13 | -0.28 | -0.20 | 0.11 | 0.02 |
| P62192 | PSMC1 | 49.18 | -0.27 | -0.24 | -0.25 | 0.02 | -0.27 | -0.17 | -0.22 | 0.08 | -0.04 |
| P28063 | PSMB8 | 30.26 | -0.53 | -0.58 | -0.55 | 0.03 | -0.43 | -0.57 | -0.50 | 0.10 | -0.05 |
| P70195 | PSMB7 | 29.89 | -0.12 | -0.12 | -0.12 | 0.00 | -0.25 | -0.10 | -0.18 | 0.10 | 0.06 |
| O55234 | PSMB5 | 28.53 | -0.83 | -0.01 | -0.42 | 0.58 | -0.11 | -0.14 | -0.13 | 0.02 | -0.29 |
| Q9R1P1 | PSMB3 | 22.97 | -0.32 | -0.22 | -0.27 | 0.07 | -0.31 | -0.24 | -0.28 | 0.05 | 0.01 |
| Q9R1P3 | PSMB2 | 22.91 | -0.33 | -0.25 | -0.29 | 0.06 | -0.31 | -0.23 | -0.27 | 0.06 | -0.02 |
| O09061 | PSMB1 | 26.37 | -0.28 | -0.13 | -0.21 | 0.11 | -0.22 | -0.20 | -0.21 | 0.02 | 0.00 |
| Q9Z2U0 | PSMA7 | 27.86 | -0.24 | -0.15 | -0.19 | 0.06 | -0.24 | -0.22 | -0.23 | 0.01 | 0.04 |

| Q9QUM9 | PSMA6 | 27.37 | -0.29 | -0.17 | -0.23 | 0.09 | -0.26 | -0.22 | -0.24 | 0.02 | 0.01 |
| --- | --- | --- | --- | --- | --- | --- | --- | --- | --- | --- | --- |
| Q9Z2U1 | PSMA5 | 26.41 | -0.19 | -0.16 | -0.18 | 0.02 | -0.22 | -0.18 | -0.20 | 0.03 | 0.02 |
| Q9R1P0 | PSMA4 | 29.47 | -0.17 | -0.22 | -0.20 | 0.03 | -0.18 | -0.25 | -0.21 | 0.05 | 0.02 |
| O70435 | PSMA3 | 28.41 | -0.30 | -0.19 | -0.25 | 0.08 | -0.26 | -0.24 | -0.25 | 0.02 | 0.00 |
| P49722 | PSMA2 | 25.93 | -0.26 | -0.16 | -0.21 | 0.07 | -0.25 | -0.25 | -0.25 | 0.00 | 0.04 |
| Q9R1P4 | PSMA1 | 29.55 | -0.20 | -0.25 | -0.22 | 0.03 | -0.20 | -0.25 | -0.23 | 0.03 | 0.00 |
| Q99JF8 | PSIP1 | 59.70 | 0.01 | -0.06 | -0.02 | 0.05 | 0.27 | 0.05 | 0.16 | 0.15 | -0.18 |
| Q61207 | PSAP | 61.42 | 0.61 | 0.50 | 0.56 | 0.08 | 0.31 | 0.17 | 0.24 | 0.09 | 0.32 |
| Q8BIW1 | PRUNE | 50.24 | 0.58 | 0.51 | 0.54 | 0.05 | 0.18 | 0.26 | 0.22 | 0.05 | 0.32 |
| Q3TLH4 | PRRC2C | 310.89 | -0.20 | -0.36 | -0.28 | 0.11 | -0.33 | -0.14 | -0.24 | 0.14 | -0.05 |
| Q3UPH1 | PRRC1 | 46.30 | -0.30 | 0.00 | -0.15 | 0.21 | -0.69 | 0.02 | -0.33 | 0.50 | 0.19 |
| Q9D0M1 | PRPSAP1 | 39.43 | 0.04 | 0.13 | 0.09 | 0.06 | -0.24 | 0.05 | -0.10 | 0.21 | 0.19 |
| Q9D7G0 |  | 34.83 | -0.25 | -0.24 | -0.24 | 0.01 | -0.22 | -0.18 | -0.20 | 0.03 | -0.04 |

| Q99PV0 | PRPF8 | 273.61 | 0.08 | -0.18 | -0.05 | 0.19 | 0.07 | -0.21 | -0.07 | 0.20 | 0.02 |
| --- | --- | --- | --- | --- | --- | --- | --- | --- | --- | --- | --- |
| Q91YR7 | PRPF6 | 106.72 | -0.05 | -0.14 | -0.09 | 0.06 | -0.02 | -0.08 | -0.05 | 0.04 | -0.04 |
| Q9R1C7 | PRPF40A | 108.48 | -0.72 | -0.07 | -0.39 | 0.46 | -0.13 | -0.07 | -0.10 | 0.04 | -0.29 |
| Q8CCF0 | PRPF31 | 55.43 | -0.25 | -0.33 | -0.29 | 0.05 | -0.02 | -0.16 | -0.09 | 0.10 | -0.20 |
| Q922U1 | PRPF3 | 77.45 | -0.23 | -0.25 | -0.24 | 0.01 | -0.13 | -0.14 | -0.13 | 0.01 | -0.10 |
| Q99KP6 | PRPF19 | 55.24 | -0.01 | 0.03 | 0.01 | 0.03 | 0.10 | 0.03 | 0.07 | 0.05 | -0.05 |
| Q9Z2Y8 | PROSC | 30.05 | 0.34 | 0.34 | 0.34 | 0.00 | 0.37 | 0.35 | 0.36 | 0.02 | -0.02 |
| Q8VCZ9 | PRODH2 | 50.72 | -0.40 | -0.54 | -0.47 | 0.10 | -0.43 | -0.52 | -0.47 | 0.07 | 0.00 |
| Q9WU79 | PRODH | 68.04 | 0.04 | 0.15 | 0.10 | 0.08 | 0.25 | 0.27 | 0.26 | 0.01 | -0.16 |
| O08795 | PRKCSH | 58.79 | 0.03 | 0.02 | 0.03 | 0.01 | 0.09 | 0.04 | 0.06 | 0.04 | -0.04 |
| P12367 | PRKAR2A | 45.39 | 0.09 | 0.16 | 0.13 | 0.05 | 0.13 | 0.13 | 0.13 | 0.00 | 0.00 |
| O54950 | PRKAG1 | 37.52 | -0.69 | -0.70 | -0.70 | 0.01 | -0.71 | -0.72 | -0.71 | 0.00 | 0.02 |
| Q9R078 | PRKAB1 | 30.31 | -0.83 | -0.80 | -0.82 | 0.03 | -0.79 | -0.69 | -0.74 | 0.07 | -0.08 |

| Q8BRK8 | PRKAA2 | 62.02 | -0.73 | -0.95 | -0.84 | 0.16 | -0.70 | -0.75 | -0.72 | 0.04 | -0.12 |
| --- | --- | --- | --- | --- | --- | --- | --- | --- | --- | --- | --- |
| Q9QUR6 | PREP | 80.75 | -0.08 | -0.19 | -0.14 | 0.08 | -0.10 | -0.17 | -0.13 | 0.05 | -0.01 |
| Q9JK53 | PRELP | 43.29 | 0.20 | 0.48 | 0.34 | 0.20 | 0.65 | 0.71 | 0.68 | 0.04 | -0.34 |
| Q9WUQ2 | PREB | 45.44 | -0.02 | 0.19 | 0.08 | 0.15 | 0.01 | 0.20 | 0.10 | 0.13 | -0.02 |
| O08709 | PRDX6 | 24.87 | 0.14 | 0.17 | 0.15 | 0.02 | 0.07 | 0.07 | 0.07 | 0.00 | 0.08 |
| P99029 | PRDX5 | 21.90 | 0.41 | 0.55 | 0.48 | 0.09 | 0.36 | 0.51 | 0.43 | 0.10 | 0.05 |
| P20108 | PRDX3 | 28.13 | -0.13 | -0.04 | -0.09 | 0.07 | -0.13 | -0.01 | -0.07 | 0.09 | -0.01 |
| Q61171 | PRDX2 | 21.78 | 0.04 | 0.08 | 0.06 | 0.03 | 0.01 | 0.05 | 0.03 | 0.03 | 0.03 |
| P35700 | PRDX1 | 22.18 | -0.25 | -0.21 | -0.23 | 0.03 | -0.15 | -0.20 | -0.18 | 0.03 | -0.05 |
| O88531 | PPT1 | 34.49 | 1.11 | 1.16 | 1.14 | 0.04 | 0.78 | 0.85 | 0.82 | 0.05 | 0.32 |
| Q922D4 | PPP6R3 | 94.65 | -0.25 | -0.11 | -0.18 | 0.10 | -0.12 | -0.18 | -0.15 | 0.04 | -0.02 |
| Q60676 | PPP5C | 56.88 | -0.26 | -0.28 | -0.27 | 0.01 | -0.09 | -0.24 | -0.17 | 0.11 | -0.10 |
| P63328 | PPP3CA | 58.64 | -0.35 | -0.39 | -0.37 | 0.03 | -0.18 | -0.36 | -0.27 | 0.13 | -0.10 |

| Q61151 | PPP2R5E | 54.71 | -0.13 | 0.09 | -0.02 | 0.16 | 0.21 | 0.24 | 0.22 | 0.03 | -0.25 |
| --- | --- | --- | --- | --- | --- | --- | --- | --- | --- | --- | --- |
| Q6PD03 | PPP2R5A | 56.35 | -0.44 | -0.38 | -0.41 | 0.04 | -0.33 | -0.25 | -0.29 | 0.05 | -0.12 |
| P58389 | PPP2R4 | 36.71 | 0.03 | 0.06 | 0.05 | 0.02 | -0.07 | 0.00 | -0.03 | 0.05 | 0.08 |
| Q6P1F6 | PPP2R2A | 51.69 | -0.21 | -0.17 | -0.19 | 0.03 | -0.24 | -0.16 | -0.20 | 0.06 | 0.01 |
| Q7TNP2 | PPP2R1B | 65.93 | 0.52 | 0.51 | 0.51 | 0.01 | 0.44 | 0.40 | 0.42 | 0.03 | 0.10 |
| Q76MZ3 | PPP2R1A | 65.32 | -0.22 | -0.16 | -0.19 | 0.04 | -0.27 | -0.19 | -0.23 | 0.06 | 0.04 |
| Q3UM45 | PPP1R7 | 41.29 | -0.14 | -0.26 | -0.20 | 0.09 | -0.21 | -0.28 | -0.24 | 0.05 | 0.04 |
| Q3TDD9 | PPP1R21 | 88.34 | 0.14 | 0.21 | 0.18 | 0.05 | 0.36 | 0.16 | 0.26 | 0.14 | -0.08 |
| Q9DBR7 | PPP1R12A | 114.99 | 0.14 | -0.14 | 0.00 | 0.20 | -0.17 | -0.06 | -0.12 | 0.08 | 0.11 |
| Q80W00 | PPP1R10 | 94.37 | 0.12 | -0.17 | -0.03 | 0.21 | -0.27 | -0.01 | -0.14 | 0.19 | 0.11 |
| Q8BVQ5 | PPME1 | 42.26 | -0.20 | -0.18 | -0.19 | 0.01 | -0.28 | -0.16 | -0.22 | 0.08 | 0.03 |
| Q61074 | PPM1G | 58.73 | -0.20 | -0.12 | -0.16 | 0.06 | 0.54 | -0.06 | 0.24 | 0.42 | -0.39 |
| Q8CGA0 | PPM1F | 49.61 | 0.00 | 0.12 | 0.06 | 0.08 | -0.08 | 0.09 | 0.01 | 0.12 | 0.05 |

| P36993 | PPM1B | 42.80 | -0.21 | -0.15 | -0.18 | 0.05 | -0.23 | -0.22 | -0.23 | 0.01 | 0.05 |
| --- | --- | --- | --- | --- | --- | --- | --- | --- | --- | --- | --- |
| P49443 | PPM1A | 42.43 | 0.10 | 0.01 | 0.06 | 0.06 | -0.11 | -0.12 | -0.12 | 0.01 | 0.17 |
| Q9CR16 | PPID | 40.74 | 0.05 | 0.06 | 0.06 | 0.01 | 0.01 | 0.00 | 0.01 | 0.01 | 0.05 |
| P24369 | PPIB | 23.71 | -0.17 | -0.13 | -0.15 | 0.03 | -0.11 | 0.01 | -0.05 | 0.09 | -0.10 |
| P17742 | PPIA | 17.97 | 0.05 | 0.15 | 0.10 | 0.07 | 0.09 | 0.16 | 0.13 | 0.05 | -0.03 |
| Q8C8U0 | PPFIBP1 | 108.54 | -0.84 | -0.38 | -0.61 | 0.32 | -0.40 | -0.03 | -0.22 | 0.26 | -0.39 |
| Q8VDG5 | PPCS | 33.79 | -0.12 | -0.20 | -0.16 | 0.05 | -0.35 | -0.26 | -0.31 | 0.06 | 0.15 |
| Q91VM9 | PPA2 | 38.11 | 0.55 | 0.41 | 0.48 | 0.10 | 0.50 | 0.45 | 0.48 | 0.04 | 0.00 |
| Q9D819 | PPA1 | 32.67 | 0.25 | 0.19 | 0.22 | 0.04 | 0.18 | 0.12 | 0.15 | 0.05 | 0.07 |
| Q62009 | POSTN | 93.14 | -0.81 | -0.98 | -0.90 | 0.12 | -0.67 | -1.02 | -0.85 | 0.25 | -0.05 |
| P37040 | POR | 77.04 | 0.37 | 0.30 | 0.33 | 0.05 | 0.45 | 0.42 | 0.44 | 0.02 | -0.10 |
| Q62087 | PON3 | 39.35 | 0.29 | 0.21 | 0.25 | 0.06 | 0.15 | 0.29 | 0.22 | 0.10 | 0.03 |
| Q62086 | PON2 | 39.62 | 0.47 | 0.33 | 0.40 | 0.09 | 0.31 | 0.39 | 0.35 | 0.06 | 0.05 |

| P52430 | PON1 | 39.57 | -0.62 | -0.69 | -0.65 | 0.05 | -0.63 | -0.60 | -0.61 | 0.03 | -0.04 |
| --- | --- | --- | --- | --- | --- | --- | --- | --- | --- | --- | --- |
| Q8CFI7 | POLR2B | 133.91 | -0.11 | -0.14 | -0.13 | 0.03 | 0.05 | -0.12 | -0.03 | 0.12 | -0.09 |
| Q8BG81 | POLDIP3 | 46.13 | -0.25 | -0.36 | -0.31 | 0.07 | -0.05 | 0.00 | -0.03 | 0.04 | -0.28 |
| Q91VA6 | POLDIP2 | 41.87 | 0.06 | 0.22 | 0.14 | 0.11 | 0.02 | 0.15 | 0.08 | 0.09 | 0.06 |
| Q8K1R3 | PNPT1 | 85.68 | 0.03 | -0.09 | -0.03 | 0.08 | 0.07 | 0.03 | 0.05 | 0.03 | -0.08 |
| Q91XF0 | PNPO | 30.11 | 0.68 | 0.64 | 0.66 | 0.03 | 0.61 | 0.63 | 0.62 | 0.01 | 0.04 |
| P23492 | PNP | 32.28 | -0.34 | -0.32 | -0.33 | 0.01 | -0.25 | -0.27 | -0.26 | 0.02 | -0.07 |
| O35691 | PNN | 82.44 | -0.09 | -0.02 | -0.06 | 0.05 | -0.24 | -0.03 | -0.13 | 0.15 | 0.07 |
| Q9DC61 | PMPCA | 58.28 | 0.19 | 0.22 | 0.20 | 0.02 | 0.11 | 0.23 | 0.17 | 0.09 | 0.03 |
| Q9Z2M7 | PMM2 | 27.66 | -0.31 | -0.23 | -0.27 | 0.06 | -0.30 | -0.23 | -0.26 | 0.04 | 0.00 |
| Q8C165 | PM20D1 | 55.66 | -0.12 | 0.02 | -0.05 | 0.10 | 0.08 | 0.26 | 0.17 | 0.13 | -0.22 |
| B2RXS4 | PLXNB2 | 206.23 | 0.21 | 0.23 | 0.22 | 0.01 | 0.31 | 0.30 | 0.31 | 0.00 | -0.08 |
| Q99K51 | PLS3 | 70.74 | 0.08 | 0.06 | 0.07 | 0.01 | 0.24 | 0.12 | 0.18 | 0.09 | -0.11 |

| Q9DBG5 | PLIN3 | 47.26 | -0.18 | -0.28 | -0.23 | 0.07 | -0.25 | -0.27 | -0.26 | 0.01 | 0.03 |
| --- | --- | --- | --- | --- | --- | --- | --- | --- | --- | --- | --- |
| P43883 | PLIN2 | 46.65 | 1.83 | 1.79 | 1.83 | 0.02 | -1.83 | 0.70 | -0.56 | 1.78 | 2.39 |
| P20918 | PLG | 90.81 | -0.10 | -0.11 | -0.11 | 0.01 | -0.16 | -0.12 | -0.14 | 0.03 | 0.03 |
| Q9QXS1 | PLEC | 534.18 | 0.32 | 0.31 | 0.32 | 0.01 | 0.37 | 0.52 | 0.44 | 0.10 | -0.13 |
| Q8VCI0 | PLBD1 | 63.00 | 1.06 | 1.05 | 1.06 | 0.01 | 1.11 | 1.27 | 1.19 | 0.11 | -0.13 |
| P27612 | PLAA | 87.22 | 0.10 | -0.02 | 0.04 | 0.08 | -0.01 | -0.03 | -0.02 | 0.01 | 0.05 |
| P52480 | PKM | 57.84 | -0.44 | -0.60 | -0.52 | 0.11 | -0.35 | -0.58 | -0.46 | 0.16 | -0.05 |
| P53657 | PKLR | 62.31 | -1.50 | -1.51 | -1.51 | 0.01 | -1.91 | -1.90 | -1.91 | 0.01 | 0.40 |
| Q8K411 | PITRM1 | 117.37 | -0.03 | -0.14 | -0.08 | 0.08 | -0.01 | -0.16 | -0.09 | 0.11 | 0.00 |
| P53811 | PITPNB | 31.49 | -0.23 | -0.18 | -0.20 | 0.03 | -0.22 | -0.20 | -0.21 | 0.02 | 0.00 |
| P53810 | PITPNA | 31.89 | -0.07 | -0.07 | -0.07 | 0.00 | -0.01 | -0.13 | -0.07 | 0.09 | 0.00 |
| Q9D711 | PIR | 32.07 | 0.47 | 0.37 | 0.42 | 0.07 | 0.44 | 0.40 | 0.42 | 0.03 | 0.00 |
| Q9D826 | PIPOX | 43.85 | 0.04 | 0.07 | 0.06 | 0.02 | 0.18 | 0.26 | 0.22 | 0.06 | -0.16 |

| P26450 | PIK3R1 | 83.52 | -0.14 | -0.32 | -0.23 | 0.12 | -0.06 | -0.25 | -0.15 | 0.14 | -0.08 |
| --- | --- | --- | --- | --- | --- | --- | --- | --- | --- | --- | --- |
| Q6PF93 | PIK3C3 | 101.49 | 0.12 | -0.07 | 0.03 | 0.13 | -0.08 | 0.04 | -0.02 | 0.09 | 0.05 |
| Q9EQ32 | PIK3AP1 | 90.93 | -0.65 | -0.12 | -0.39 | 0.37 | 0.02 | -0.06 | -0.02 | 0.06 | -0.37 |
| O70570 | PIGR | 85.00 | 0.50 | 1.06 | 0.78 | 0.40 | 0.62 | 0.93 | 0.77 | 0.22 | 0.00 |
| Q7M6Y3 | PICALM | 71.54 | 0.22 | 0.28 | 0.25 | 0.04 | 0.14 | 0.22 | 0.18 | 0.05 | 0.07 |
| Q9DB26 | PHYHD1 | 32.52 | 0.02 | 0.05 | 0.04 | 0.02 | 0.02 | 0.07 | 0.05 | 0.03 | -0.01 |
| O35386 | PHYH | 38.61 | 1.19 | 1.28 | 1.24 | 0.06 | 1.15 | 1.20 | 1.17 | 0.04 | 0.06 |
| Q9DB30 | PHKG2 | 46.57 | -0.16 | -0.19 | -0.18 | 0.02 | -0.04 | -0.12 | -0.08 | 0.05 | -0.10 |
| O35129 | PHB2 | 33.30 | 0.27 | 0.24 | 0.25 | 0.03 | 0.18 | 0.18 | 0.18 | 0.00 | 0.07 |
| P67778 | PHB | 29.82 | 0.24 | 0.24 | 0.24 | 0.00 | 0.15 | 0.22 | 0.18 | 0.05 | 0.06 |
| O55022 | PGRMC1 | 21.69 | 0.46 | 0.22 | 0.34 | 0.17 | 0.49 | 0.32 | 0.41 | 0.12 | -0.07 |
| Q8CHP8 | PGP | 34.54 | 0.03 | 0.02 | 0.03 | 0.01 | -0.23 | -0.20 | -0.22 | 0.02 | 0.24 |
| Q9CYR6 | PGM3 | 59.45 | -0.05 | -0.08 | -0.06 | 0.02 | -0.42 | -0.17 | -0.29 | 0.18 | 0.23 |

| Q7TSV4 | PGM2 | 68.75 | 0.52 | 0.40 | 0.46 | 0.09 | 0.56 | 0.36 | 0.46 | 0.14 | 0.00 |
| --- | --- | --- | --- | --- | --- | --- | --- | --- | --- | --- | --- |
| Q9D0F9 | PGM1 | 61.42 | 0.09 | 0.12 | 0.11 | 0.02 | 0.06 | 0.01 | 0.04 | 0.04 | 0.07 |
| P09411 | PGK1 | 44.55 | -0.16 | -0.13 | -0.14 | 0.02 | -0.27 | -0.19 | -0.23 | 0.06 | 0.09 |
| Q9DCD0 | PGD | 53.25 | -0.84 | -0.91 | -0.87 | 0.05 | -1.00 | -1.05 | -1.02 | 0.04 | 0.15 |
| Q3UUQ7 | PGAP1 | 104.58 | 0.64 | 0.49 | 0.57 | 0.11 | 0.64 | 0.67 | 0.66 | 0.02 | -0.09 |
| Q9DBJ1 | PGAM1 | 28.83 | -0.04 | 0.77 | 0.37 | 0.57 | -0.12 | -0.05 | -0.09 | 0.04 | 0.45 |
| P47857 | PFKM | 85.27 | -0.75 | -0.81 | -0.78 | 0.05 | -0.81 | -0.85 | -0.83 | 0.03 | 0.05 |
| P12382 | PFKL | 85.36 | -0.45 | -0.57 | -0.51 | 0.09 | -0.48 | -0.57 | -0.53 | 0.06 | 0.02 |
| P70266 | PFKFB1 | 54.85 | -0.51 | -0.48 | -0.49 | 0.02 | -0.77 | -0.51 | -0.64 | 0.18 | 0.14 |
| O09012 | PEX5 | 70.76 | 0.34 | 0.15 | 0.25 | 0.14 | 0.08 | 0.18 | 0.13 | 0.07 | 0.12 |
| Q8VCI5 | PEX19 | 32.73 | 0.35 | 0.30 | 0.32 | 0.03 | 0.07 | 0.03 | 0.05 | 0.03 | 0.28 |
| Q9R0A0 | PEX14 | 41.21 | -0.23 | -0.02 | -0.12 | 0.15 | -0.28 | -0.11 | -0.19 | 0.12 | 0.07 |
| Q5BL07 | PEX1 | 141.43 | 0.09 | 0.27 | 0.18 | 0.13 | 0.52 | 0.14 | 0.33 | 0.27 | -0.15 |

| Q99MZ7 | PECR | 32.41 | 0.10 | 0.09 | 0.10 | 0.01 | 0.23 | 0.19 | 0.21 | 0.03 | -0.12 |
| --- | --- | --- | --- | --- | --- | --- | --- | --- | --- | --- | --- |
| P70296 | PEBP1 | 20.83 | 0.38 | 0.32 | 0.35 | 0.04 | 0.62 | 0.73 | 0.68 | 0.07 | -0.33 |
| Q9JIL4 | PDZK1 | 56.50 | -0.10 | -0.15 | -0.12 | 0.03 | -0.06 | -0.14 | -0.10 | 0.05 | -0.02 |
| Q8K183 | PDXK | 35.02 | 0.09 | -0.06 | 0.01 | 0.10 | -0.10 | -0.03 | -0.06 | 0.05 | 0.08 |
| Q99K01 | PDXDC1 | 87.33 | -0.11 | 0.18 | 0.04 | 0.20 | 0.12 | 0.26 | 0.19 | 0.10 | -0.15 |
| Q4VA53 | PDS5B | 164.42 | -0.25 | -0.31 | -0.28 | 0.04 | -0.34 | -0.20 | -0.27 | 0.09 | -0.01 |
| Q8CI51 | PDLIM5 | 63.30 | -0.65 | -0.51 | -0.58 | 0.10 | -0.52 | -0.44 | -0.48 | 0.06 | -0.10 |
| O70400 | PDLIM1 | 35.77 | -0.25 | -0.21 | -0.23 | 0.03 | -0.14 | -0.12 | -0.13 | 0.01 | -0.10 |
| Q9JK42 | PDK2 | 46.04 | 0.29 | 0.13 | 0.21 | 0.12 | -0.07 | 0.03 | -0.02 | 0.07 | 0.23 |
| Q8BFP9 | PDK1 | 48.99 | -0.43 | -0.34 | -0.38 | 0.06 | -0.65 | -0.52 | -0.59 | 0.09 | 0.20 |
| Q922R8 | PDIA6 | 48.10 | -0.13 | -0.16 | -0.15 | 0.02 | -0.04 | -0.11 | -0.08 | 0.04 | -0.07 |
| Q921X9 | PDIA5 | 59.27 | 0.33 | 0.28 | 0.30 | 0.04 | 0.37 | 0.42 | 0.39 | 0.04 | -0.09 |
| P08003 | PDIA4 | 71.98 | -0.12 | -0.21 | -0.17 | 0.06 | -0.12 | -0.22 | -0.17 | 0.07 | 0.00 |

| P27773 | PDIA3 | 56.68 | 0.01 | 0.04 | 0.02 | 0.02 | -0.02 | -0.01 | -0.01 | 0.01 | 0.03 |
| --- | --- | --- | --- | --- | --- | --- | --- | --- | --- | --- | --- |
| Q8BKZ9 | PDHX | 54.00 | -0.26 | -0.05 | -0.16 | 0.15 | -0.26 | -0.14 | -0.20 | 0.08 | 0.04 |
| Q9D051 | PDHB | 38.94 | -0.10 | -0.14 | -0.12 | 0.03 | -0.16 | -0.28 | -0.22 | 0.08 | 0.10 |
| P35486 | PDHA1 | 43.23 | -0.13 | -0.10 | -0.12 | 0.02 | -0.31 | -0.26 | -0.29 | 0.03 | 0.17 |
| Q9WU78 | PDCD6IP | 96.02 | -0.01 | 0.04 | 0.01 | 0.04 | -0.14 | 0.03 | -0.06 | 0.12 | 0.07 |
| Q61823 | PDCD4 | 51.70 | 0.08 | 0.44 | 0.26 | 0.25 | 0.57 | 0.57 | 0.57 | 0.00 | -0.31 |
| Q6NS46 | PDCD11 | 207.78 | -0.06 | -0.20 | -0.13 | 0.10 | 0.09 | -0.09 | 0.00 | 0.13 | -0.13 |
| Q3UHX2 | PDAP1 | 20.61 | -0.21 | 0.08 | -0.07 | 0.20 | -0.13 | 0.19 | 0.03 | 0.23 | -0.09 |
| Q922E4 | PCYT2 | 45.23 | -0.23 | -0.24 | -0.23 | 0.00 | -0.35 | -0.26 | -0.31 | 0.06 | 0.07 |
| P49586 | PCYT1A | 41.67 | -0.62 | -0.74 | -0.68 | 0.08 | -0.58 | -0.40 | -0.49 | 0.13 | -0.19 |
| Q9CQF9 | PCYOX1 | 56.49 | -0.28 | -0.20 | -0.24 | 0.06 | -0.19 | -0.28 | -0.23 | 0.06 | -0.01 |
| P53808 | PCTP | 24.79 | -0.53 | -0.48 | -0.51 | 0.04 | -0.82 | -0.72 | -0.77 | 0.07 | 0.26 |
| P59913 | PCMTD1 | 40.69 | -0.10 | 0.02 | -0.04 | 0.09 | 0.03 | 0.11 | 0.07 | 0.06 | -0.11 |

| Q9Z2V4 | PCK1 | 69.35 | 2.20 | 2.18 | 2.19 | 0.01 | 2.23 | 2.19 | 2.21 | 0.03 | -0.02 |
| --- | --- | --- | --- | --- | --- | --- | --- | --- | --- | --- | --- |
| Q99MN9 | PCCB | 58.41 | 0.15 | 0.18 | 0.17 | 0.02 | 0.14 | 0.21 | 0.17 | 0.05 | -0.01 |
| Q91ZA3 | PCCA | 79.92 | 0.28 | 0.24 | 0.26 | 0.02 | 0.26 | 0.19 | 0.22 | 0.05 | 0.04 |
| Q61990 |  | 38.22 | 0.08 | 0.02 | 0.05 | 0.04 | 0.01 | 0.10 | 0.05 | 0.06 | 0.00 |
| P60335 | PCBP1 | 37.50 | -0.14 | -0.05 | -0.09 | 0.07 | -0.02 | -0.02 | -0.02 | 0.00 | -0.08 |
| P61458 | PCBD1 | 11.99 | -0.43 | -0.47 | -0.45 | 0.02 | -0.52 | -0.49 | -0.50 | 0.02 | 0.05 |
| Q05920 | PC | 129.68 | -0.30 | -0.08 | -0.19 | 0.15 | -0.36 | -0.19 | -0.28 | 0.12 | 0.09 |
| Q9CXN7 | PBLD2 | 31.98 | -0.01 | 0.07 | 0.03 | 0.06 | -0.03 | 0.00 | -0.01 | 0.02 | 0.04 |
| Q9DCG6 | PBLD1 | 32.05 | 0.00 | 0.00 | 0.00 | 0.00 | -0.03 | 0.02 | -0.01 | 0.04 | 0.01 |
| P58501 | PAXBP1 | 104.83 | -0.01 | -0.06 | -0.03 | 0.04 | -0.04 | 0.06 | 0.01 | 0.07 | -0.04 |
| Q9EPC1 | PARVA | 42.33 | 0.29 | 0.27 | 0.28 | 0.02 | 0.37 | 0.28 | 0.32 | 0.07 | -0.04 |
| Q8CAS9 | PARP9 | 96.66 | -0.19 | -0.30 | -0.25 | 0.08 | -0.26 | -0.40 | -0.33 | 0.10 | 0.08 |
| P11103 | PARP1 | 113.10 | -0.55 | -0.60 | -0.58 | 0.04 | -0.23 | -0.48 | -0.36 | 0.17 | -0.22 |

| Q8VDG3 | PARN | 71.56 | -0.34 | -0.31 | -0.32 | 0.02 | -0.39 | -0.08 | -0.24 | 0.22 | -0.09 |
| --- | --- | --- | --- | --- | --- | --- | --- | --- | --- | --- | --- |
| Q5XJY4 | PARL | 41.96 | 0.10 | 0.16 | 0.13 | 0.05 | 0.14 | 0.25 | 0.20 | 0.07 | -0.06 |
| Q99LX0 | PARK7 | 20.02 | 0.21 | 0.27 | 0.24 | 0.05 | 0.10 | 0.19 | 0.15 | 0.06 | 0.09 |
| O88428 | PAPSS2 | 70.35 | -1.16 | -1.15 | -1.16 | 0.01 | -0.86 | -0.86 | -0.86 | 0.00 | -0.29 |
| Q80YV4 | PANK4 | 91.52 | -0.32 | -0.48 | -0.40 | 0.12 | -0.12 | -0.33 | -0.23 | 0.15 | -0.18 |
| Q9JHU2 | PALMD | 62.70 | -0.21 | -0.10 | -0.16 | 0.08 | -0.48 | -0.12 | -0.30 | 0.26 | 0.14 |
| Q9ET54 | PALLD | 152.13 | -0.79 | -0.44 | -0.62 | 0.25 | -0.87 | -0.52 | -0.69 | 0.25 | 0.08 |
| Q8CIN4 | PAK2 | 57.93 | -0.22 | -0.22 | -0.22 | 0.00 | -0.18 | -0.19 | -0.19 | 0.01 | -0.03 |
| Q9DCL9 | PAICS | 47.01 | 0.00 | -0.08 | -0.04 | 0.06 | -0.07 | 0.04 | -0.01 | 0.08 | -0.03 |
| P16331 | PAH | 51.90 | -0.03 | -0.03 | -0.03 | 0.00 | 0.04 | 0.04 | 0.04 | 0.01 | -0.07 |
| P63005 | PAFAH1B1 | 46.67 | 0.14 | -0.07 | 0.03 | 0.15 | -0.06 | -0.05 | -0.05 | 0.01 | 0.09 |
| Q8K2T8 | PAF1 | 60.52 | -0.33 | -0.16 | -0.25 | 0.12 | -0.29 | -0.10 | -0.20 | 0.14 | -0.05 |
| Q99JB8 | PACSIN3 | 48.58 | 0.55 | 0.62 | 0.58 | 0.06 | 0.56 | 0.67 | 0.62 | 0.08 | -0.03 |

| Q9WVE8 | PACSIN2 | 55.83 | -0.07 | 0.19 | 0.06 | 0.18 | 0.19 | 0.24 | 0.22 | 0.03 | -0.15 |
| --- | --- | --- | --- | --- | --- | --- | --- | --- | --- | --- | --- |
| Q8CCS6 | PABPN1 | 32.30 | -0.02 | -0.04 | -0.03 | 0.02 | 0.13 | 0.07 | 0.10 | 0.04 | -0.13 |
| P29341 | PABPC1 | 70.67 | 0.69 | 0.05 | 0.37 | 0.45 | 0.38 | 0.06 | 0.22 | 0.22 | 0.15 |
| P50580 | PA2G4 | 43.70 | 0.43 | 0.33 | 0.38 | 0.08 | 0.35 | 0.32 | 0.34 | 0.02 | 0.04 |
| P09103 | P4HB | 57.06 | -0.02 | -0.02 | -0.02 | 0.00 | -0.07 | -0.06 | -0.06 | 0.01 | 0.04 |
| Q60715 | P4HA1 | 60.91 | -0.75 | -0.83 | -0.79 | 0.05 | -0.41 | -1.10 | -0.75 | 0.48 | -0.03 |
| Q6P9R2 | OXSR1 | 58.21 | -0.01 | -0.03 | -0.02 | 0.01 | -0.10 | 0.04 | -0.03 | 0.10 | 0.01 |
| Q9D404 | OXSM | 48.63 | 0.48 | 0.38 | 0.43 | 0.06 | 0.42 | 0.28 | 0.35 | 0.10 | 0.08 |
| Q4KMM3 | OXR1 | 95.91 | 0.02 | 0.24 | 0.13 | 0.15 | 0.00 | 0.13 | 0.06 | 0.09 | 0.06 |
| Q8BGA9 | OXA1L | 48.22 | -0.20 | -0.24 | -0.22 | 0.03 | -0.69 | -0.24 | -0.47 | 0.32 | 0.25 |
| Q8K2H2 | OTUD6B | 33.76 | 0.14 | -0.11 | 0.01 | 0.17 | 0.12 | -0.01 | 0.05 | 0.09 | -0.04 |
| Q7TQI3 | OTUB1 | 31.27 | -0.15 | -0.07 | -0.11 | 0.06 | -0.11 | -0.15 | -0.13 | 0.03 | 0.02 |
| P11725 | OTC | 39.76 | 0.49 | 0.36 | 0.43 | 0.09 | 0.36 | 0.52 | 0.44 | 0.11 | -0.01 |

| Q62422 | OSTF1 | 23.78 | -0.20 | -0.14 | -0.17 | 0.04 | -0.19 | -0.26 | -0.22 | 0.05 | 0.05 |
| --- | --- | --- | --- | --- | --- | --- | --- | --- | --- | --- | --- |
| Q8BWU5 | OSGEP | 36.30 | -0.37 | -0.61 | -0.49 | 0.17 | -0.37 | -0.29 | -0.33 | 0.06 | -0.16 |
| Q3B7Z2 | OSBP | 88.80 | -0.11 | -0.12 | -0.11 | 0.01 | -0.08 | -0.04 | -0.06 | 0.02 | -0.05 |
| Q8K2C7 | OS9 | 76.11 | -0.17 | -0.28 | -0.23 | 0.07 | -0.14 | -0.29 | -0.21 | 0.10 | -0.01 |
| Q60590 | ORM1 | 23.90 | 1.62 | 1.39 | 1.51 | 0.17 | 1.21 | 1.25 | 1.23 | 0.03 | 0.28 |
| Q8K3K8 | OPTN | 67.02 | 0.79 | 0.90 | 0.84 | 0.08 | 0.66 | 0.60 | 0.63 | 0.04 | 0.21 |
| Q8K010 | OPLAH | 137.61 | 0.17 | 0.74 | 0.45 | 0.41 | 0.57 | 0.59 | 0.58 | 0.02 | -0.13 |
| P58281 | OPA1 | 111.34 | 0.39 | 0.25 | 0.32 | 0.09 | 0.18 | 0.22 | 0.20 | 0.03 | 0.12 |
| Q9CZ30 | OLA1 | 44.73 | 0.36 | 0.40 | 0.38 | 0.03 | 0.25 | 0.28 | 0.26 | 0.02 | 0.12 |
| Q8CGY8 | OGT | 116.95 | -0.03 | -0.29 | -0.16 | 0.18 | 0.03 | -0.30 | -0.13 | 0.23 | -0.03 |
| Q60597 | OGDH | 116.45 | 0.06 | -0.05 | 0.01 | 0.08 | 0.04 | -0.03 | 0.00 | 0.05 | 0.00 |
| Q9CRD0 | OCIAD1 | 27.61 | -0.06 | -0.15 | -0.10 | 0.07 | -0.17 | -0.17 | -0.17 | 0.00 | 0.06 |
| P29758 | OAT | 48.35 | 1.46 | 1.38 | 1.42 | 0.06 | 1.30 | 1.30 | 1.30 | 0.00 | 0.12 |

| Q8BJ71 | NUP93 | 93.28 | -0.22 | -0.24 | -0.23 | 0.02 | -0.13 | -0.09 | -0.11 | 0.03 | -0.12 |
| --- | --- | --- | --- | --- | --- | --- | --- | --- | --- | --- | --- |
| Q9QY81 | NUP210 | 204.10 | -0.30 | -0.56 | -0.43 | 0.18 | -0.11 | -0.11 | -0.11 | 0.00 | -0.31 |
| Q8BVU5 | NUDT9 | 38.60 | -0.20 | -0.32 | -0.26 | 0.08 | -0.16 | -0.30 | -0.23 | 0.10 | -0.03 |
| Q99P30 | NUDT7 | 26.86 | 1.37 | 1.36 | 1.37 | 0.01 | 1.28 | 1.31 | 1.29 | 0.03 | 0.07 |
| Q9JKX6 | NUDT5 | 23.98 | -0.26 | -0.21 | -0.23 | 0.03 | -0.27 | -0.23 | -0.25 | 0.03 | 0.02 |
| Q9CQF3 | NUDT21 | 26.24 | -0.15 | 0.04 | -0.05 | 0.13 | 0.01 | -0.12 | -0.05 | 0.10 | 0.00 |
| P11930 | NUDT19 | 40.32 | -0.34 | -0.33 | -0.34 | 0.00 | -0.21 | -0.22 | -0.22 | 0.01 | -0.12 |
| Q9DCN1 | NUDT12 | 51.51 | 0.20 | 0.34 | 0.27 | 0.10 | 0.16 | 0.24 | 0.20 | 0.06 | 0.07 |
| O35685 | NUDC | 38.36 | -0.27 | -0.21 | -0.24 | 0.04 | -0.27 | -0.21 | -0.24 | 0.04 | 0.00 |
| Q02819 | NUCB1 | 53.41 | -0.06 | -0.07 | -0.06 | 0.01 | 0.06 | 0.07 | 0.06 | 0.01 | -0.13 |
| Q9CWD8 | NUBPL | 34.14 | -0.10 | 0.01 | -0.05 | 0.08 | -0.03 | 0.10 | 0.03 | 0.10 | -0.08 |
| P54729 | NUB1 | 70.31 | -0.14 | -0.28 | -0.21 | 0.10 | -0.20 | -0.23 | -0.22 | 0.02 | 0.01 |
| Q61503 | NT5E | 63.86 | -0.91 | -0.83 | -0.87 | 0.05 | -1.12 | -1.07 | -1.10 | 0.04 | 0.22 |

| Q8C5P5 | NT5DC1 | 53.09 | -0.21 | -0.18 | -0.19 | 0.02 | -0.61 | -0.28 | -0.45 | 0.23 | 0.25 |
| --- | --- | --- | --- | --- | --- | --- | --- | --- | --- | --- | --- |
| Q9D020 | NT5C3A | 37.25 | 0.35 | -0.20 | 0.08 | 0.39 | 0.22 | -0.12 | 0.05 | 0.24 | 0.03 |
| Q1HFZ0 | NSUN2 | 85.45 | 0.07 | 0.10 | 0.08 | 0.02 | 0.14 | 0.17 | 0.15 | 0.03 | -0.07 |
| Q9CZ44 | NSFL1C | 40.71 | -0.24 | -0.32 | -0.28 | 0.05 | -0.36 | -0.29 | -0.32 | 0.05 | 0.04 |
| P46460 | NSF | 82.61 | 0.07 | -0.08 | -0.01 | 0.10 | 0.08 | -0.01 | 0.03 | 0.07 | -0.04 |
| Q9R1J0 | NSDHL | 40.69 | 0.08 | -0.02 | 0.03 | 0.07 | 0.13 | 0.13 | 0.13 | 0.00 | -0.10 |
| Q8BHG1 | NRD1 | 132.89 | -0.36 | -0.16 | -0.26 | 0.14 | 0.00 | -0.04 | -0.02 | 0.03 | -0.24 |
| Q9JI75 | NQO2 | 26.25 | -0.22 | -0.21 | -0.22 | 0.01 | -0.22 | -0.20 | -0.21 | 0.02 | -0.01 |
| Q64669 | NQO1 | 30.96 | -0.69 | -0.52 | -0.60 | 0.12 | -0.45 | -0.57 | -0.51 | 0.09 | -0.09 |
| Q61937 | NPM1 | 32.56 | -0.14 | -0.20 | -0.17 | 0.04 | -0.03 | -0.04 | -0.04 | 0.01 | -0.14 |
| P60670 | NPLOC4 | 68.02 | 0.24 | 0.24 | 0.24 | 0.00 | 0.22 | 0.14 | 0.18 | 0.06 | 0.06 |
| Q9DCJ9 | NPL | 35.13 | -0.16 | -0.18 | -0.17 | 0.02 | -0.22 | -0.22 | -0.22 | 0.00 | 0.05 |
| Q11011 | NPEPPS | 103.32 | 0.10 | 0.01 | 0.06 | 0.06 | 0.07 | -0.03 | 0.02 | 0.07 | 0.04 |

| Q6NSR8 | NPEPL1 | 55.94 | -0.14 | -0.10 | -0.12 | 0.03 | -0.11 | -0.07 | -0.09 | 0.03 | -0.03 |
| --- | --- | --- | --- | --- | --- | --- | --- | --- | --- | --- | --- |
| Q6DFW4 | NOP58 | 60.34 | 0.22 | 0.35 | 0.28 | 0.09 | 0.38 | 0.43 | 0.40 | 0.04 | -0.12 |
| Q9D6Z1 | NOP56 | 64.46 | 0.45 | 0.40 | 0.42 | 0.04 | 0.58 | 0.46 | 0.52 | 0.09 | -0.09 |
| Q99K48 | NONO | 54.54 | -0.11 | -0.12 | -0.12 | 0.01 | -0.09 | -0.06 | -0.07 | 0.03 | -0.05 |
| Q6GQT9 | NOMO1 | 133.42 | 0.09 | 0.25 | 0.17 | 0.11 | 0.23 | 0.19 | 0.21 | 0.03 | -0.04 |
| O70310 | NMT1 | 56.89 | 0.09 | 0.10 | 0.10 | 0.01 | 0.28 | 0.12 | 0.20 | 0.11 | -0.10 |
| Q8K2T1 | NMRAL1 | 34.38 | -0.75 | -0.71 | -0.73 | 0.03 | -0.29 | -0.32 | -0.31 | 0.02 | -0.42 |
| O35309 | NMI | 35.24 | -0.22 | -0.29 | -0.26 | 0.05 | -0.07 | -0.23 | -0.15 | 0.12 | -0.11 |
| Q01768 | NME2 | 17.36 | -0.08 | -0.02 | -0.05 | 0.05 | -0.15 | -0.05 | -0.10 | 0.07 | 0.05 |
| P15532 | NME1 | 17.21 | -0.10 | -0.07 | -0.09 | 0.02 | -0.16 | -0.07 | -0.12 | 0.07 | 0.03 |
| Q91YP2 | NLN | 80.43 | 0.19 | 0.02 | 0.11 | 0.12 | 0.11 | 0.04 | 0.07 | 0.05 | 0.03 |
| Q9JHW2 | NIT2 | 30.50 | -0.32 | -0.24 | -0.28 | 0.06 | -0.30 | -0.24 | -0.27 | 0.04 | -0.01 |
| O55125 | NIPSNAP1 | 33.36 | 0.42 | 0.42 | 0.42 | 0.00 | 0.43 | 0.44 | 0.43 | 0.01 | -0.01 |

| Q8BZW8 | NHLRC2 | 78.43 | 0.09 | -0.03 | 0.03 | 0.09 | -0.09 | -0.09 | -0.09 | 0.00 | 0.12 |
| --- | --- | --- | --- | --- | --- | --- | --- | --- | --- | --- | --- |
| Q9Z1J3 | NFS1 | 50.57 | 0.01 | 0.23 | 0.12 | 0.16 | 0.21 | 0.23 | 0.22 | 0.01 | -0.10 |
| Q8CCP0 | NEMF | 121.19 | -0.08 | -0.07 | -0.07 | 0.00 | -0.40 | -0.06 | -0.23 | 0.24 | 0.16 |
| Q8C4Y3 | NELFB | 65.64 | -0.15 | -0.10 | -0.13 | 0.04 | 0.10 | -0.18 | -0.04 | 0.20 | -0.09 |
| P46935 | NEDD4 | 102.71 | -0.09 | 0.00 | -0.05 | 0.06 | 0.14 | 0.07 | 0.11 | 0.05 | -0.15 |
| Q9D6J6 | NDUFV2 | 27.29 | 0.03 | 0.22 | 0.12 | 0.14 | 0.21 | 0.19 | 0.20 | 0.01 | -0.08 |
| Q91YT0 | NDUFV1 | 50.83 | 0.13 | 0.07 | 0.10 | 0.05 | 0.10 | 0.18 | 0.14 | 0.05 | -0.04 |
| Q8K3J1 | NDUFS8 | 24.04 | 0.09 | 0.31 | 0.20 | 0.15 | 0.18 | 0.34 | 0.26 | 0.11 | -0.06 |
| Q9CXZ1 | NDUFS4 | 19.78 | 0.06 | 0.08 | 0.07 | 0.01 | 0.14 | 0.12 | 0.13 | 0.01 | -0.06 |
| Q9DCT2 | NDUFS3 | 30.15 | 0.13 | 0.21 | 0.17 | 0.06 | 0.22 | 0.22 | 0.22 | 0.00 | -0.05 |
| Q91WD5 | NDUFS2 | 52.63 | 0.07 | 0.18 | 0.12 | 0.08 | 0.14 | 0.18 | 0.16 | 0.03 | -0.04 |
| Q91VD9 | NDUFS1 | 79.78 | 0.17 | 0.12 | 0.14 | 0.03 | 0.16 | 0.20 | 0.18 | 0.03 | -0.04 |
| Q9CQ54 | NDUFC2 | 14.16 | 0.03 | 0.12 | 0.08 | 0.07 | 0.15 | 0.11 | 0.13 | 0.03 | -0.05 |

| Q9CQJ8 | NDUFB9 | 21.98 | 0.11 | 0.13 | 0.12 | 0.02 | 0.10 | 0.13 | 0.11 | 0.02 | 0.00 |
| --- | --- | --- | --- | --- | --- | --- | --- | --- | --- | --- | --- |
| Q9D6J5 | NDUFB8 | 21.88 | 0.10 | 0.12 | 0.11 | 0.01 | 0.22 | 0.23 | 0.23 | 0.01 | -0.12 |
| Q9CR61 | NDUFB7 | 16.33 | 0.25 | 0.13 | 0.19 | 0.08 | 0.14 | 0.20 | 0.17 | 0.04 | 0.02 |
| Q9CQH3 | NDUFB5 | 21.71 | 0.09 | 0.11 | 0.10 | 0.01 | 0.09 | 0.15 | 0.12 | 0.04 | -0.02 |
| Q9CQC7 | NDUFB4 | 15.08 | 0.02 | 0.20 | 0.11 | 0.12 | 0.01 | 0.19 | 0.10 | 0.12 | 0.01 |
| Q9CQZ6 | NDUFB3 | 11.69 | -0.09 | 0.14 | 0.02 | 0.16 | 0.25 | 0.16 | 0.21 | 0.06 | -0.18 |
| O09111 | NDUFB11 | 17.44 | 0.21 | 0.10 | 0.16 | 0.08 | -0.02 | 0.27 | 0.13 | 0.20 | 0.03 |
| Q9DCS9 | NDUFB10 | 21.02 | 0.07 | 0.09 | 0.08 | 0.01 | 0.14 | 0.18 | 0.16 | 0.03 | -0.08 |
| A2AIL4 | NDUFAF6 | 38.36 | -0.23 | -0.33 | -0.28 | 0.07 | -0.04 | 0.04 | 0.00 | 0.06 | -0.28 |
| Q59J78 | NDUFAF2 | 19.63 | -0.14 | -0.09 | -0.12 | 0.04 | -0.35 | 0.01 | -0.17 | 0.26 | 0.05 |
| Q9CWX2 | NDUFAF1 | 37.81 | 0.18 | 0.22 | 0.20 | 0.03 | 0.10 | 0.16 | 0.13 | 0.04 | 0.07 |
| Q9CR21 | NDUFAB1 | 17.37 | 0.06 | -0.03 | 0.01 | 0.06 | 0.04 | 0.04 | 0.04 | 0.01 | -0.03 |
| Q9DC69 | NDUFA9 | 42.53 | 0.08 | 0.04 | 0.06 | 0.03 | 0.25 | 0.14 | 0.19 | 0.08 | -0.13 |

| Q9DCJ5 | NDUFA8 | 19.99 | 0.07 | 0.14 | 0.11 | 0.05 | 0.11 | 0.20 | 0.15 | 0.06 | -0.04 |
| --- | --- | --- | --- | --- | --- | --- | --- | --- | --- | --- | --- |
| Q9Z1P6 | NDUFA7 | 12.58 | 0.05 | 0.12 | 0.08 | 0.05 | 0.09 | 0.11 | 0.10 | 0.02 | -0.02 |
| Q9CQZ5 | NDUFA6 | 15.28 | 0.13 | 0.11 | 0.12 | 0.02 | -0.11 | 0.16 | 0.03 | 0.19 | 0.09 |
| Q9CPP6 | NDUFA5 | 13.36 | 0.09 | 0.16 | 0.13 | 0.05 | -0.03 | 0.16 | 0.06 | 0.13 | 0.06 |
| Q62425 | NDUFA4 | 9.33 | -0.19 | 0.02 | -0.09 | 0.14 | -0.02 | -0.04 | -0.03 | 0.02 | -0.06 |
| Q9CQ75 | NDUFA2 | 10.92 | 0.06 | 0.10 | 0.08 | 0.03 | 0.16 | 0.19 | 0.18 | 0.02 | -0.10 |
| Q7TMF3 | NDUFA12 | 17.09 | 0.01 | 0.07 | 0.04 | 0.04 | 0.02 | 0.16 | 0.09 | 0.10 | -0.05 |
| Q99LC3 | NDUFA10 | 40.60 | 0.13 | 0.12 | 0.12 | 0.01 | 0.14 | 0.20 | 0.17 | 0.04 | -0.05 |
| Q9QYG0 | NDRG2 | 40.79 | 0.02 | -0.11 | -0.05 | 0.09 | -0.21 | -0.15 | -0.18 | 0.05 | 0.13 |
| Q8VCM8 | NCLN | 62.91 | 0.36 | 0.49 | 0.43 | 0.09 | 0.18 | 0.24 | 0.21 | 0.04 | 0.22 |
| P09405 | NCL | 76.72 | -0.05 | -0.05 | -0.05 | 0.00 | 0.27 | 0.01 | 0.14 | 0.18 | -0.19 |
| P28660 | NCKAP1 | 128.78 | 0.13 | 0.10 | 0.12 | 0.02 | 0.29 | 0.15 | 0.22 | 0.09 | -0.10 |
| Q3UYV9 | NCBP1 | 91.93 | 0.04 | -0.22 | -0.09 | 0.18 | 0.05 | -0.06 | 0.00 | 0.08 | -0.09 |

| Q8K224 | NAT10 | 115.42 | 0.37 | 0.00 | 0.18 | 0.26 | 0.11 | 0.03 | 0.07 | 0.06 | 0.12 |
| --- | --- | --- | --- | --- | --- | --- | --- | --- | --- | --- | --- |
| Q99MD9 | NASP | 83.95 | -0.34 | -0.42 | -0.38 | 0.06 | -0.06 | -0.41 | -0.24 | 0.25 | -0.14 |
| Q8BP47 | NARS | 64.28 | 0.11 | 0.09 | 0.10 | 0.01 | 0.08 | 0.10 | 0.09 | 0.02 | 0.01 |
| Q8CC86 | NAPRT | 58.27 | -0.10 | -0.13 | -0.12 | 0.02 | -0.23 | -0.16 | -0.19 | 0.05 | 0.08 |
| Q9CWZ7 | NAPG | 34.73 | 0.09 | 0.07 | 0.08 | 0.01 | 0.06 | 0.04 | 0.05 | 0.01 | 0.03 |
| Q9DB05 | NAPA | 33.19 | -0.08 | -0.07 | -0.07 | 0.00 | 0.02 | -0.05 | -0.02 | 0.04 | -0.06 |
| Q78ZA7 | NAP1L4 | 42.68 | -0.01 | -0.03 | -0.02 | 0.01 | -0.13 | -0.19 | -0.16 | 0.04 | 0.14 |
| P28656 | NAP1L1 | 45.35 | -0.15 | 0.13 | -0.01 | 0.20 | -0.26 | -0.04 | -0.15 | 0.15 | 0.14 |
| Q99KQ4 | NAMPT | 55.45 | -0.30 | -0.28 | -0.29 | 0.02 | -0.26 | -0.23 | -0.25 | 0.02 | -0.05 |
| Q9QZ08 | NAGK | 37.27 | -0.16 | -0.39 | -0.27 | 0.16 | -0.36 | -0.33 | -0.34 | 0.02 | 0.07 |
| Q9QWR8 | NAGA | 47.23 | 0.95 | 0.98 | 0.96 | 0.02 | 0.88 | 0.96 | 0.92 | 0.05 | 0.04 |
| Q8VBW6 | NAE1 | 60.27 | -0.09 | -0.12 | -0.11 | 0.03 | -0.21 | -0.02 | -0.12 | 0.13 | 0.01 |
| Q8C5H8 | NADK2 | 50.86 | 0.32 | 0.34 | 0.33 | 0.01 | 0.39 | 0.32 | 0.35 | 0.05 | -0.03 |

| Q6PHQ8 | NAA35 | 83.31 | 0.18 | 0.03 | 0.10 | 0.11 | -0.05 | 0.01 | -0.02 | 0.04 | 0.12 |
| --- | --- | --- | --- | --- | --- | --- | --- | --- | --- | --- | --- |
| Q80UM3 | NAA15 | 100.96 | 0.11 | 0.08 | 0.09 | 0.02 | 0.12 | 0.12 | 0.12 | 0.00 | -0.02 |
| Q9QY36 |  | 26.52 | -0.13 | 0.05 | -0.04 | 0.12 | -0.13 | 0.03 | -0.05 | 0.11 | 0.01 |
| Q64331 | MYO6 | 146.41 | -0.15 | 0.29 | 0.07 | 0.31 | -0.37 | 0.18 | -0.10 | 0.39 | 0.17 |
| E9Q634 | MYO1E | 126.82 | -0.32 | -0.06 | -0.19 | 0.18 | -0.21 | -0.12 | -0.17 | 0.06 | -0.03 |
| Q5SYD0 | MYO1D | 116.08 | 0.12 | 0.13 | 0.12 | 0.01 | 0.25 | 0.18 | 0.21 | 0.05 | -0.09 |
| Q9WTI7 | MYO1C | 121.94 | 0.06 | 0.06 | 0.06 | 0.00 | 0.34 | 0.20 | 0.27 | 0.10 | -0.21 |
| P46735 | MYO1B | 128.56 | 0.44 | 0.49 | 0.46 | 0.03 | 0.58 | 0.61 | 0.60 | 0.02 | -0.13 |
| Q9JMH9 | MYO18A | 232.75 | 0.05 | -0.10 | -0.02 | 0.10 | -0.12 | 0.04 | -0.04 | 0.11 | 0.02 |
| Q6PDN3 | MYLK | 212.92 | -0.11 | -0.15 | -0.13 | 0.03 | -0.12 | -0.11 | -0.12 | 0.01 | -0.01 |
| Q60605 | MYL6 | 16.93 | -0.20 | -0.09 | -0.15 | 0.08 | -0.02 | -0.06 | -0.04 | 0.03 | -0.11 |
|  |  | 19.78 | -0.21 | -0.04 | -0.12 | 0.12 | -0.08 | 0.04 | -0.02 | 0.09 | -0.10 |
| P05977 | MYL1 | 20.59 | -5.26 | -5.36 | -5.31 | 0.07 | -4.98 | -5.18 | -5.08 | 0.14 | -0.23 |

| Q8VDD5 | MYH9 | 226.37 | -0.41 | -0.19 | -0.30 | 0.16 | -0.13 | -0.11 | -0.12 | 0.01 | -0.18 |
| --- | --- | --- | --- | --- | --- | --- | --- | --- | --- | --- | --- |
| P13542 | MYH8 | 222.70 | -3.94 | -5.24 | -4.59 | 0.93 | -4.69 | -5.07 | -4.88 | 0.27 | 0.29 |
| Q91Z83 | MYH7 | 222.88 | -4.38 | -4.94 | -4.66 | 0.40 | -4.86 | -4.86 | -4.86 | 0.00 | 0.20 |
| Q5SX39 | MYH4 | 222.86 | -5.46 | -5.01 | -5.24 | 0.32 | -5.07 | -4.93 | -5.00 | 0.10 | -0.24 |
| Q6URW6 | MYH14 | 228.58 | -0.11 | -0.15 | -0.13 | 0.03 | -0.07 | -0.07 | -0.07 | 0.00 | -0.06 |
| O08638 | MYH11 | 227.03 | 1.05 | 0.98 | 1.01 | 0.05 | 1.03 | 0.99 | 1.01 | 0.03 | 0.00 |
| Q61879 | MYH10 | 228.99 | 0.73 | 0.59 | 0.66 | 0.09 | 0.79 | 0.68 | 0.73 | 0.08 | -0.07 |
| Q5SX40 | MYH1 | 223.34 | -5.69 | -5.19 | -5.44 | 0.35 | -5.20 | -5.13 | -5.16 | 0.05 | -0.28 |
| Q9JK81 | MYG1 | 42.72 | 0.11 | 0.13 | 0.12 | 0.02 | 0.12 | 0.16 | 0.14 | 0.03 | -0.02 |
| Q7TPV4 | MYBBP1A | 152.04 | -0.62 | -0.34 | -0.48 | 0.19 | -0.48 | -0.20 | -0.34 | 0.20 | -0.14 |
| Q9EQK5 | MVP | 95.92 | 0.35 | 0.31 | 0.33 | 0.03 | 0.38 | 0.34 | 0.36 | 0.03 | -0.03 |
| Q9R008 | MVK | 41.88 | -0.11 | -0.18 | -0.14 | 0.05 | 0.03 | 0.05 | 0.04 | 0.01 | -0.19 |
| Q99JF5 | MVD | 44.07 | -1.14 | -1.27 | -1.20 | 0.10 | -0.94 | -1.19 | -1.07 | 0.17 | -0.14 |

| P16332 | MUT | 82.84 | 0.44 | 0.44 | 0.44 | 0.00 | 0.36 | 0.42 | 0.39 | 0.04 | 0.05 |
| --- | --- | --- | --- | --- | --- | --- | --- | --- | --- | --- | --- |
| P11589 | MUP2 | 20.66 | 3.93 | 5.00 | 4.46 | 0.76 | 4.83 | 4.99 | 4.91 | 0.12 | -0.45 |
| P28666 | MUG2 | 162.38 | 0.11 | 0.42 | 0.26 | 0.22 | 0.30 | 0.41 | 0.35 | 0.08 | -0.09 |
| P28665 | MUG1 | 165.30 | 1.03 | 1.24 | 1.14 | 0.15 | 1.01 | 1.16 | 1.09 | 0.11 | 0.05 |
| O88441 | MTX2 | 29.76 | 0.07 | 0.18 | 0.12 | 0.08 | -0.03 | 0.05 | 0.01 | 0.05 | 0.11 |
| P47802 | MTX1 | 35.62 | 0.06 | 0.09 | 0.07 | 0.02 | 0.03 | 0.02 | 0.03 | 0.01 | 0.05 |
| Q5HZI1 | MTUS1 | 134.38 | 0.22 | 0.06 | 0.14 | 0.11 | 0.53 | 0.26 | 0.40 | 0.20 | -0.25 |
| O08601 | MTTP | 99.10 | -0.03 | -0.11 | -0.07 | 0.06 | -0.24 | -0.25 | -0.25 | 0.00 | 0.18 |
| P03921 | MTND5 | 68.47 | 0.04 | 0.05 | 0.05 | 0.01 | 0.03 | 0.09 | 0.06 | 0.04 | -0.01 |
| Q9Z2C5 | MTM1 | 69.56 | 0.14 | -0.13 | 0.00 | 0.19 | 0.27 | 0.02 | 0.14 | 0.18 | -0.14 |
| Q9D110 | MTHFS | 23.20 | -0.17 | -0.08 | -0.12 | 0.06 | 0.02 | -0.04 | -0.01 | 0.04 | -0.11 |
| Q922D8 | MTHFD1 | 101.20 | -0.34 | -0.40 | -0.37 | 0.04 | -0.31 | -0.32 | -0.31 | 0.01 | -0.05 |
| Q80WJ7 | MTDH | 63.85 | -0.26 | -0.03 | -0.15 | 0.16 | -0.25 | -0.02 | -0.14 | 0.17 | -0.01 |

| Q791V5 | MTCH2 | 33.50 | 0.36 | 0.17 | 0.26 | 0.13 | 0.24 | 0.13 | 0.18 | 0.07 | 0.08 |
| --- | --- | --- | --- | --- | --- | --- | --- | --- | --- | --- | --- |
| Q9CQ65 | MTAP | 31.06 | -0.29 | -0.29 | -0.29 | 0.00 | -0.15 | -0.06 | -0.10 | 0.06 | -0.19 |
| Q9R190 | MTA2 | 75.03 | 0.02 | 0.10 | 0.06 | 0.06 | 0.05 | 0.08 | 0.06 | 0.02 | 0.00 |
| Q9D6Y7 | MSRA | 25.99 | 0.19 | 0.21 | 0.20 | 0.01 | 0.20 | 0.19 | 0.20 | 0.01 | 0.00 |
| P30204 | MSR1 | 50.17 | -0.86 | -0.82 | -0.84 | 0.03 | -0.51 | -0.55 | -0.53 | 0.03 | -0.31 |
| P26041 | MSN | 67.77 | -0.19 | -0.27 | -0.23 | 0.06 | 0.11 | -0.30 | -0.10 | 0.29 | -0.14 |
| Q9CRA4 | MSMO1 | 34.77 | 0.25 | -0.39 | -0.07 | 0.45 | 0.60 | 0.06 | 0.33 | 0.38 | -0.40 |
| Q9D6S7 | MRRF | 29.05 | -0.04 | 0.03 | -0.01 | 0.05 | -0.06 | 0.07 | 0.01 | 0.09 | -0.02 |
| Q9D7N3 | MRPS9 | 44.93 | 0.28 | 0.22 | 0.25 | 0.04 | 0.05 | 0.22 | 0.14 | 0.12 | 0.11 |
| Q80X85 | MRPS7 | 28.06 | 0.07 | 0.13 | 0.10 | 0.05 | 0.08 | 0.27 | 0.17 | 0.14 | -0.07 |
| Q99N87 | MRPS5 | 48.21 | 0.13 | 0.13 | 0.13 | 0.00 | 0.17 | 0.13 | 0.15 | 0.03 | -0.02 |
| Q8BJZ4 | MRPS35 | 35.98 | 0.11 | 0.16 | 0.13 | 0.03 | 0.00 | 0.18 | 0.09 | 0.12 | 0.05 |
| Q61733 | MRPS31 | 43.88 | -0.09 | 0.17 | 0.04 | 0.18 | 0.10 | 0.23 | 0.16 | 0.09 | -0.12 |

| Q8BK72 | MRPS27 | 47.78 | 0.16 | 0.15 | 0.15 | 0.00 | -0.03 | 0.14 | 0.05 | 0.12 | 0.10 |
| --- | --- | --- | --- | --- | --- | --- | --- | --- | --- | --- | --- |
| Q80ZS3 | MRPS26 | 23.44 | 0.33 | 0.36 | 0.34 | 0.02 | 0.16 | 0.26 | 0.21 | 0.07 | 0.13 |
| Q9CXW2 | MRPS22 | 41.19 | 0.10 | 0.21 | 0.16 | 0.08 | 0.09 | 0.17 | 0.13 | 0.05 | 0.03 |
| Q9DC71 | MRPS15 | 29.46 | -0.01 | 0.13 | 0.06 | 0.10 | 0.09 | 0.22 | 0.15 | 0.09 | -0.09 |
| Q9EQI8 | MRPL46 | 32.13 | -0.07 | -0.05 | -0.06 | 0.02 | 0.04 | 0.04 | 0.04 | 0.00 | -0.10 |
| Q9JKF7 | MRPL39 | 38.55 | 0.05 | -0.03 | 0.01 | 0.06 | -0.08 | 0.02 | -0.03 | 0.07 | 0.04 |
| Q99N93 | MRPL16 | 28.80 | -0.08 | 0.04 | -0.02 | 0.09 | -0.08 | -0.01 | -0.05 | 0.04 | 0.02 |
| Q9CPR5 | MRPL15 | 33.54 | -0.08 | -0.06 | -0.07 | 0.01 | 0.04 | 0.00 | 0.02 | 0.03 | -0.08 |
| Q9CQF0 | MRPL11 | 20.68 | -0.24 | 0.12 | -0.06 | 0.26 | -0.21 | 0.00 | -0.10 | 0.15 | 0.04 |
| Q99N96 | MRPL1 | 37.60 | 0.09 | -0.07 | 0.01 | 0.12 | 0.05 | -0.09 | -0.02 | 0.10 | 0.03 |
| Q61830 | MRC1 | 164.98 | -0.42 | -0.36 | -0.39 | 0.04 | -0.43 | -0.32 | -0.38 | 0.07 | -0.02 |
| Q99J99 | MPST | 33.02 | 0.18 | 0.05 | 0.12 | 0.09 | 0.51 | 0.45 | 0.48 | 0.04 | -0.37 |
| Q9JLB0 | MPP6 | 62.63 | -0.38 | -0.28 | -0.33 | 0.07 | -0.12 | -0.17 | -0.14 | 0.04 | -0.18 |

| Q924M7 | MPI | 46.58 | -0.18 | 0.11 | -0.04 | 0.21 | -0.13 | 0.07 | -0.03 | 0.14 | -0.01 |
| --- | --- | --- | --- | --- | --- | --- | --- | --- | --- | --- | --- |
| P23249 | MOV10 | 113.58 | -0.52 | -0.62 | -0.57 | 0.08 | -0.26 | -0.46 | -0.36 | 0.14 | -0.21 |
| Q9CWP6 | MOSPD2 | 59.85 | -0.12 | -0.08 | -0.10 | 0.03 | -0.49 | -0.24 | -0.37 | 0.17 | 0.27 |
| Q80UM7 | MOGS | 91.83 | 0.15 | 0.07 | 0.11 | 0.06 | 0.20 | 0.12 | 0.16 | 0.05 | -0.05 |
| Q5RKZ7 | MOCS1 | 69.86 | 0.19 | 0.63 | 0.41 | 0.31 | 0.20 | 0.60 | 0.40 | 0.28 | 0.01 |
| Q14CH1 | MOCOS | 95.01 | 0.14 | 0.13 | 0.13 | 0.01 | 0.10 | 0.23 | 0.17 | 0.09 | -0.03 |
|  |  | 25.09 | -0.26 | -0.13 | -0.19 | 0.09 | -0.30 | -0.22 | -0.26 | 0.06 | 0.07 |
| Q8C7H1 | MMAA | 45.93 | 0.15 | 0.14 | 0.14 | 0.01 | 0.08 | 0.08 | 0.08 | 0.00 | 0.06 |
| Q99J39 | MLYCD | 54.74 | 0.08 | 0.03 | 0.06 | 0.03 | 0.04 | -0.04 | 0.00 | 0.06 | 0.05 |
| Q9QZQ1 | MLLT4 | 206.50 | 0.30 | 0.48 | 0.39 | 0.13 | 0.30 | 0.58 | 0.44 | 0.20 | -0.06 |
| Q6ZQI3 | MLEC | 32.34 | 0.43 | 0.49 | 0.46 | 0.04 | 0.38 | 0.50 | 0.44 | 0.08 | 0.02 |
| Q8BI84 | MIA3 | 213.67 | -0.02 | 0.37 | 0.17 | 0.27 | -0.18 | 0.38 | 0.10 | 0.40 | 0.07 |
| Q91ZV0 | MIA2 | 57.78 | 0.00 | 0.63 | 0.32 | 0.45 | -0.25 | 0.73 | 0.24 | 0.70 | 0.08 |

| Q91VS7 | MGST1 | 17.55 | 1.16 | 1.28 | 1.22 | 0.08 | 0.88 | 1.11 | 0.99 | 0.16 | 0.23 |
| --- | --- | --- | --- | --- | --- | --- | --- | --- | --- | --- | --- |
| O35678 | MGLL | 33.39 | -0.60 | -0.56 | -0.58 | 0.03 | -0.99 | -1.01 | -1.00 | 0.01 | 0.42 |
| Q9EQQ9 | MGEA5 | 103.16 | -0.05 | 0.05 | 0.00 | 0.07 | -0.08 | 0.20 | 0.06 | 0.19 | -0.06 |
| Q80U63 | MFN2 | 86.19 | 0.38 | 0.40 | 0.39 | 0.02 | 0.58 | 0.41 | 0.50 | 0.12 | -0.11 |
| Q811U4 | MFN1 | 83.73 | 0.09 | 0.00 | 0.04 | 0.07 | -0.07 | 0.07 | 0.00 | 0.10 | 0.05 |
| Q9CQU1 | MFAP1 | 51.95 | -0.03 | -0.20 | -0.11 | 0.12 | -0.01 | 0.07 | 0.03 | 0.06 | -0.14 |
| Q9DD20 | METTL7B | 28.05 | -0.02 | 0.10 | 0.04 | 0.09 | 0.03 | 0.05 | 0.04 | 0.02 | 0.00 |
| O08663 | METAP2 | 52.92 | -0.23 | -0.25 | -0.24 | 0.01 | -0.26 | -0.22 | -0.24 | 0.03 | 0.00 |
| Q8BP48 | METAP1 | 43.22 | 0.46 | 0.45 | 0.45 | 0.01 | 0.42 | 0.47 | 0.45 | 0.03 | 0.01 |
| Q9ERE7 | MESDC2 | 25.21 | -0.28 | -0.19 | -0.23 | 0.06 | -0.18 | -0.15 | -0.16 | 0.02 | -0.07 |
| Q91VH6 | MEMO1 | 33.69 | 0.27 | 0.31 | 0.29 | 0.03 | 0.32 | 0.28 | 0.30 | 0.02 | -0.01 |
| Q9DCS3 | MECR | 40.34 | 0.21 | 0.36 | 0.29 | 0.10 | 0.28 | 0.51 | 0.40 | 0.17 | -0.11 |
| Q9Z2D6 | MECP2 | 52.31 | -0.28 | 0.21 | -0.04 | 0.35 | -0.25 | 0.35 | 0.05 | 0.43 | -0.08 |

| P06801 | ME1 | 63.95 | -1.15 | -1.15 | -1.15 | 0.00 | -1.73 | -1.69 | -1.71 | 0.03 | 0.56 |
| --- | --- | --- | --- | --- | --- | --- | --- | --- | --- | --- | --- |
| P08249 | MDH2 | 35.61 | 0.15 | 0.04 | 0.09 | 0.08 | 0.14 | 0.04 | 0.09 | 0.07 | 0.00 |
| P14152 | MDH1 | 36.51 | 0.00 | -0.06 | -0.03 | 0.04 | -0.01 | -0.05 | -0.03 | 0.02 | 0.00 |
| Q9CXD6 | MCUR1 | 37.85 | 0.21 | 0.17 | 0.19 | 0.03 | 0.27 | 0.16 | 0.22 | 0.08 | -0.03 |
| Q3UMR5 | MCU | 39.68 | 0.19 | -0.08 | 0.06 | 0.19 | 0.07 | 0.31 | 0.19 | 0.17 | -0.13 |
| Q8K5B2 | MCFD2 | 16.17 | 0.06 | -0.03 | 0.01 | 0.06 | -0.17 | -0.20 | -0.18 | 0.01 | 0.20 |
| Q9D1I5 | MCEE | 19.02 | -0.17 | 0.08 | -0.05 | 0.18 | -0.10 | 0.12 | 0.01 | 0.15 | -0.06 |
| Q3ULD5 | MCCC2 | 61.38 | 0.14 | 0.26 | 0.20 | 0.09 | 0.04 | 0.16 | 0.10 | 0.08 | 0.10 |
| Q99MR8 | MCCC1 | 79.34 | 0.36 | 0.25 | 0.30 | 0.08 | 0.24 | 0.16 | 0.20 | 0.06 | 0.11 |
| Q8R3F5 | MCAT | 41.93 | 0.11 | 0.08 | 0.09 | 0.02 | 0.08 | 0.24 | 0.16 | 0.11 | -0.06 |
| P39039 | MBL1 | 25.40 | 0.17 | 0.25 | 0.21 | 0.05 | -0.04 | 0.07 | 0.01 | 0.08 | 0.20 |
| Q8VCF0 | MAVS | 53.40 | -0.47 | -0.28 | -0.37 | 0.13 | -0.32 | -0.17 | -0.24 | 0.10 | -0.13 |
| Q8K310 | MATR3 | 94.63 | -0.08 | -0.02 | -0.05 | 0.04 | -0.02 | -0.03 | -0.02 | 0.00 | -0.03 |

| Q99LB6 | MAT2B | 37.39 | -0.04 | -0.06 | -0.05 | 0.01 | -0.14 | -0.08 | -0.11 | 0.04 | 0.05 |
| --- | --- | --- | --- | --- | --- | --- | --- | --- | --- | --- | --- |
| Q3THS6 | MAT2A | 43.69 | 0.45 | 0.39 | 0.42 | 0.04 | -0.11 | 0.13 | 0.01 | 0.17 | 0.41 |
| Q91X83 | MAT1A | 43.51 | -0.30 | -0.37 | -0.33 | 0.05 | -0.17 | -0.26 | -0.21 | 0.06 | -0.12 |
| Q68FL6 | MARS | 101.43 | 0.23 | 0.15 | 0.19 | 0.06 | 0.09 | 0.08 | 0.09 | 0.01 | 0.10 |
| P26645 | MARCKS | 29.66 | -0.89 | -0.77 | -0.83 | 0.09 | -0.37 | -0.48 | -0.43 | 0.08 | -0.40 |
| Q61166 | MAPRE1 | 30.02 | -0.52 | -0.31 | -0.41 | 0.15 | -0.29 | -0.35 | -0.32 | 0.04 | -0.09 |
| Q9WTU6 | MAPK9 | 48.19 | -0.50 | -0.22 | -0.36 | 0.20 | -0.04 | -0.11 | -0.08 | 0.05 | -0.28 |
| P63085 | MAPK1 | 41.28 | -0.14 | -0.15 | -0.14 | 0.00 | -0.18 | -0.16 | -0.17 | 0.01 | 0.02 |
| O88735 | MAP7 | 82.02 | -0.14 | 0.03 | -0.06 | 0.12 | 0.13 | 0.04 | 0.08 | 0.07 | -0.14 |
| P27546 | MAP4 | 117.43 | -0.83 | -0.30 | -0.56 | 0.38 | -0.55 | -0.22 | -0.38 | 0.23 | -0.18 |
| P47809 | MAP2K4 | 44.11 | -0.21 | -0.16 | -0.19 | 0.03 | -0.48 | -0.09 | -0.28 | 0.27 | 0.10 |
| Q63932 | MAP2K2 | 44.40 | 0.25 | 0.11 | 0.18 | 0.10 | 0.09 | 0.07 | 0.08 | 0.01 | 0.10 |
| P31938 | MAP2K1 | 43.47 | -0.02 | 0.11 | 0.04 | 0.09 | -0.03 | 0.06 | 0.01 | 0.06 | 0.03 |

| Q8BW75 | MAOB | 58.56 | -0.13 | -0.11 | -0.12 | 0.01 | -0.13 | -0.11 | -0.12 | 0.01 | 0.00 |
| --- | --- | --- | --- | --- | --- | --- | --- | --- | --- | --- | --- |
| Q64133 | MAOA | 59.60 | 0.65 | 0.47 | 0.56 | 0.13 | 0.34 | 0.37 | 0.35 | 0.02 | 0.21 |
| Q9CXI5 | MANF | 20.37 | -0.47 | -0.25 | -0.36 | 0.15 | -0.27 | -0.16 | -0.22 | 0.08 | -0.14 |
| Q8K2I4 | MANBA | 100.83 | 1.45 | 1.46 | 1.45 | 0.01 | 0.99 | 1.32 | 1.15 | 0.23 | 0.30 |
| O09159 | MAN2B1 | 114.65 | 0.44 | 0.17 | 0.30 | 0.19 | 0.22 | 0.25 | 0.24 | 0.02 | 0.07 |
| P27046 | MAN2A1 | 131.63 | 0.43 | 0.48 | 0.46 | 0.03 | 0.51 | 0.58 | 0.54 | 0.05 | -0.09 |
| P45700 | MAN1A1 | 73.28 | -0.12 | -0.03 | -0.08 | 0.07 | 0.00 | -0.05 | -0.03 | 0.04 | -0.05 |
| Q922B1 | MACROD1 | 35.29 | 0.07 | 0.18 | 0.12 | 0.08 | 0.12 | 0.19 | 0.16 | 0.05 | -0.03 |
| Q9QXZ0 | MACF1 | 831.87 | 0.20 | -0.03 | 0.09 | 0.16 | 0.08 | 0.07 | 0.08 | 0.00 | 0.01 |
| P97823 | LYPLA1 | 24.69 | -0.05 | 0.04 | 0.00 | 0.07 | -0.05 | 0.13 | 0.04 | 0.13 | -0.04 |
| P25911 | LYN | 58.81 | 0.10 | 0.08 | 0.09 | 0.01 | 0.03 | 0.15 | 0.09 | 0.09 | 0.00 |
| P51885 | LUM | 38.27 | 0.45 | 0.20 | 0.33 | 0.17 | 0.30 | 0.38 | 0.34 | 0.06 | -0.01 |
| Q7TNC4 | LUC7L2 | 46.58 | 0.24 | -0.01 | 0.11 | 0.18 | 0.11 | -0.11 | 0.00 | 0.16 | 0.11 |

| Q6A009 | LTN1 | 198.92 | 0.18 | -0.04 | 0.07 | 0.15 | 0.03 | 0.16 | 0.09 | 0.09 | -0.02 |
| --- | --- | --- | --- | --- | --- | --- | --- | --- | --- | --- | --- |
| P24527 | LTA4H | 69.05 | 0.14 | 0.05 | 0.09 | 0.06 | 0.05 | -0.03 | 0.01 | 0.06 | 0.08 |
| Q8BLN5 | LSS | 83.14 | -0.26 | -0.50 | -0.38 | 0.17 | 0.17 | -0.08 | 0.04 | 0.18 | -0.43 |
| Q8K2F8 | LSM14A | 50.55 | -0.27 | -0.06 | -0.16 | 0.15 | -0.37 | 0.03 | -0.17 | 0.28 | 0.00 |
| Q9D0R8 | LSM12 | 21.70 | 0.15 | 0.20 | 0.18 | 0.04 | 0.09 | 0.22 | 0.15 | 0.09 | 0.02 |
| Q3UZ39 | LRRFIP1 | 79.25 | -0.15 | -0.04 | -0.09 | 0.08 | -0.24 | 0.05 | -0.10 | 0.21 | 0.01 |
| Q922Q8 | LRRC59 | 34.88 | 0.32 | 0.38 | 0.35 | 0.04 | 0.28 | 0.36 | 0.32 | 0.06 | 0.03 |
| Q505F5 | LRRC47 | 63.59 | 0.06 | 0.08 | 0.07 | 0.01 | 0.24 | 0.00 | 0.12 | 0.17 | -0.04 |
| Q9CRC8 | LRRC40 | 68.08 | -0.42 | -0.31 | -0.36 | 0.08 | -0.19 | -0.31 | -0.25 | 0.09 | -0.11 |
| Q6PB66 | LRPPRC | 156.61 | -0.38 | -0.15 | -0.27 | 0.17 | -0.29 | -0.17 | -0.23 | 0.08 | -0.04 |
| P55302 | LRPAP1 | 42.22 | -0.18 | -0.11 | -0.15 | 0.05 | -0.24 | -0.09 | -0.16 | 0.11 | 0.02 |
| Q91ZX7 | LRP1 | 504.74 | -0.40 | -0.09 | -0.25 | 0.22 | -0.05 | 0.02 | -0.01 | 0.05 | -0.23 |
| Q8BFW7 | LPP | 65.89 | -0.32 | -0.11 | -0.21 | 0.15 | -0.33 | 0.01 | -0.16 | 0.24 | -0.05 |

| Q91YX5 | LPGAT1 | 43.09 | 0.57 | 0.34 | 0.45 | 0.16 | -0.10 | 0.03 | -0.03 | 0.09 | 0.48 |
| --- | --- | --- | --- | --- | --- | --- | --- | --- | --- | --- | --- |
| Q9DBN5 | LONP2 | 94.53 | 0.44 | 0.40 | 0.42 | 0.03 | 0.15 | 0.20 | 0.18 | 0.03 | 0.24 |
| Q8CGK3 | LONP1 | 105.84 | 0.09 | 0.17 | 0.13 | 0.06 | 0.10 | 0.16 | 0.13 | 0.04 | 0.00 |
| Q8C129 | LNPEP | 117.30 | -0.43 | -0.06 | -0.24 | 0.26 | -0.26 | -0.04 | -0.15 | 0.15 | -0.10 |
| P21619 | LMNB2 | 67.32 | 0.65 | 0.71 | 0.68 | 0.04 | 0.61 | 0.64 | 0.62 | 0.02 | 0.05 |
| P14733 | LMNB1 | 66.79 | -0.55 | -0.55 | -0.55 | 0.00 | -0.33 | -0.46 | -0.39 | 0.09 | -0.16 |
| P48678 | LMNA | 74.24 | 0.27 | 0.36 | 0.31 | 0.06 | 0.36 | 0.40 | 0.38 | 0.03 | -0.06 |
| Q9D0F3 | LMAN1 | 57.79 | -0.20 | -0.12 | -0.16 | 0.05 | -0.21 | -0.21 | -0.21 | 0.00 | 0.05 |
| Q9Z0M5 | LIPA | 45.33 | 2.05 | 0.87 | 1.46 | 0.83 | 0.25 | 0.75 | 0.50 | 0.35 | 0.96 |
| O88952 | LIN7C | 21.83 | 0.47 | 0.57 | 0.52 | 0.07 | 0.50 | 0.69 | 0.60 | 0.13 | -0.08 |
| Q8JZS0 | LIN7A | 25.99 | -0.21 | -0.09 | -0.15 | 0.09 | 0.00 | 0.00 | 0.00 | 0.00 | -0.15 |
| Q9ERG0 | LIMA1 | 84.06 | 0.13 | 0.35 | 0.24 | 0.16 | 0.23 | 0.27 | 0.25 | 0.03 | -0.01 |
| Q9D7I5 | LHPP | 29.14 | 0.14 | 0.23 | 0.18 | 0.06 | 0.28 | 0.28 | 0.28 | 0.01 | -0.10 |

| O89017 | LGMN | 49.37 | 0.53 | 0.49 | 0.51 | 0.03 | 0.63 | 0.66 | 0.64 | 0.02 | -0.13 |
| --- | --- | --- | --- | --- | --- | --- | --- | --- | --- | --- | --- |
| O08573 | LGALS9 | 40.04 | -0.56 | -0.52 | -0.54 | 0.03 | -0.42 | -0.55 | -0.49 | 0.09 | -0.06 |
| Q07797 | LGALS3BP | 64.49 | -0.33 | -0.57 | -0.45 | 0.17 | -0.37 | -0.87 | -0.62 | 0.36 | 0.17 |
| Q9Z2I0 | LETM1 | 82.99 | 0.41 | 0.36 | 0.39 | 0.04 | 0.34 | 0.25 | 0.30 | 0.06 | 0.09 |
| Q8C142 | LDLRAP1 | 33.98 | -0.02 | 0.17 | 0.08 | 0.13 | -0.02 | 0.06 | 0.02 | 0.06 | 0.06 |
| Q7TNG8 | LDHD | 51.85 | 1.73 | 1.71 | 1.72 | 0.01 | 1.79 | 1.86 | 1.82 | 0.05 | -0.10 |
| P16125 | LDHB | 36.57 | -0.92 | -1.04 | -0.98 | 0.09 | -0.94 | -0.02 | -0.48 | 0.65 | -0.50 |
| P06151 | LDHA | 36.50 | 0.00 | -0.05 | -0.03 | 0.03 | -0.13 | -0.17 | -0.15 | 0.03 | 0.12 |
| Q61233 | LCP1 | 70.15 | 0.23 | 0.23 | 0.23 | 0.00 | 0.37 | 0.18 | 0.27 | 0.14 | -0.05 |
| Q3UN02 | LCLAT1 | 44.40 | -0.04 | 0.03 | -0.01 | 0.05 | -0.13 | -0.03 | -0.08 | 0.07 | 0.07 |
| Q3U9G9 | LBR | 71.44 | -0.73 | -0.78 | -0.75 | 0.04 | -0.67 | -0.62 | -0.65 | 0.03 | -0.11 |
| Q61792 | LASP1 | 29.99 | -0.05 | 0.15 | 0.05 | 0.14 | 0.06 | 0.26 | 0.16 | 0.15 | -0.11 |
| Q8VDC0 | LARS2 | 101.48 | -0.04 | -0.11 | -0.07 | 0.05 | -0.20 | -0.18 | -0.19 | 0.01 | 0.12 |

| Q8BMJ2 | LARS | 134.19 | 0.02 | 0.05 | 0.04 | 0.02 | 0.10 | 0.11 | 0.10 | 0.00 | -0.06 |
| --- | --- | --- | --- | --- | --- | --- | --- | --- | --- | --- | --- |
| Q05CL8 | LARP7 | 64.80 | -0.15 | 0.09 | -0.03 | 0.17 | 0.17 | 0.07 | 0.12 | 0.07 | -0.15 |
| Q6ZQ58 | LARP1 | 121.12 | -0.75 | -0.31 | -0.53 | 0.31 | -0.70 | -0.23 | -0.47 | 0.33 | -0.06 |
| Q9CPY7 | LAP3 | 56.14 | 0.33 | 0.40 | 0.37 | 0.05 | 0.35 | 0.43 | 0.39 | 0.06 | -0.02 |
| O89112 | LANCL1 | 45.34 | 0.27 | 0.41 | 0.34 | 0.10 | 0.46 | 0.58 | 0.52 | 0.08 | -0.18 |
| P17047 | LAMP2 | 45.68 | 0.28 | 0.24 | 0.26 | 0.03 | 0.43 | 0.25 | 0.34 | 0.13 | -0.07 |
| P11438 | LAMP1 | 43.87 | 0.32 | 0.14 | 0.23 | 0.13 | 0.40 | 0.22 | 0.31 | 0.13 | -0.08 |
| P02468 | LAMC1 | 177.30 | -0.68 | -0.48 | -0.58 | 0.14 | -0.58 | -0.33 | -0.46 | 0.18 | -0.12 |
| Q99KR3 | LACTB2 | 32.75 | 0.35 | 0.33 | 0.34 | 0.02 | 0.33 | 0.32 | 0.33 | 0.01 | 0.01 |
| Q9EP89 | LACTB | 60.71 | 0.68 | 0.83 | 0.75 | 0.11 | 0.62 | 0.68 | 0.65 | 0.04 | 0.10 |
| Q9CXF0 | KYNU | 52.33 | 0.38 | 0.30 | 0.34 | 0.05 | 0.37 | 0.41 | 0.39 | 0.03 | -0.05 |
| Q61595 | KTN1 | 152.59 | 0.24 | -0.16 | 0.04 | 0.28 | 0.27 | -0.20 | 0.04 | 0.33 | 0.01 |
| P11679 | KRT8 | 54.57 | 1.22 | 1.13 | 1.18 | 0.06 | 1.37 | 1.23 | 1.30 | 0.10 | -0.12 |

| P70168 | KPNB1 | 97.18 | -0.23 | -0.26 | -0.24 | 0.02 | -0.16 | -0.18 | -0.17 | 0.02 | -0.07 |
| --- | --- | --- | --- | --- | --- | --- | --- | --- | --- | --- | --- |
| O35343 | KPNA4 | 57.92 | -0.18 | -0.02 | -0.10 | 0.11 | 0.01 | -0.07 | -0.03 | 0.05 | -0.07 |
| O35344 | KPNA3 | 57.77 | -0.17 | -0.11 | -0.14 | 0.05 | -0.13 | -0.17 | -0.15 | 0.03 | 0.01 |
| O08677 | KNG1 | 73.10 | 0.17 | 0.19 | 0.18 | 0.01 | -0.02 | -0.03 | -0.03 | 0.00 | 0.20 |
| Q91WN4 | KMO | 54.53 | 0.83 | 0.72 | 0.77 | 0.08 | 0.63 | 0.65 | 0.64 | 0.01 | 0.13 |
| Q61768 | KIF5B | 109.55 | -0.10 | -0.13 | -0.11 | 0.02 | 0.08 | -0.04 | 0.02 | 0.09 | -0.13 |
| Q9QXL2 | KIF21A | 186.53 | -0.02 | 0.32 | 0.15 | 0.24 | -0.18 | 0.08 | -0.05 | 0.18 | 0.20 |
| Q9EQW7 | KIF13A | 195.81 | 0.13 | -0.24 | -0.06 | 0.26 | 0.50 | 0.40 | 0.45 | 0.07 | -0.51 |
| Q8K2Q9 | KIAA1598 | 71.34 | 0.06 | 0.19 | 0.13 | 0.09 | 0.18 | 0.18 | 0.18 | 0.00 | -0.05 |
| Q3UMB9 | KIAA1033 | 136.37 | 0.21 | 0.00 | 0.11 | 0.15 | 0.19 | 0.01 | 0.10 | 0.12 | 0.01 |
| Q3U0V1 | KHSRP | 76.78 | -0.11 | -0.07 | -0.09 | 0.03 | 0.01 | 0.05 | 0.03 | 0.03 | -0.12 |
| P97328 | KHK | 32.75 | -0.69 | -0.59 | -0.64 | 0.07 | -0.88 | -0.85 | -0.86 | 0.02 | 0.22 |
| Q60749 | KHDRBS1 | 48.37 | -0.22 | -0.04 | -0.13 | 0.13 | -0.23 | -0.18 | -0.21 | 0.04 | 0.07 |

| Q9DCY0 | KEG1 | 33.72 | 2.27 | 2.41 | 2.34 | 0.10 | 2.32 | 2.41 | 2.37 | 0.06 | -0.03 |
| --- | --- | --- | --- | --- | --- | --- | --- | --- | --- | --- | --- |
| Q99MN1 | KARS | 67.84 | 0.03 | -0.06 | -0.02 | 0.06 | 0.03 | -0.04 | 0.00 | 0.05 | -0.02 |
| Q02257 | JUP | 81.80 | -0.20 | -0.08 | -0.14 | 0.09 | -0.15 | 0.08 | -0.04 | 0.16 | -0.10 |
| Q9DCX8 | IYD | 32.81 | -0.06 | -0.02 | -0.04 | 0.02 | -0.03 | -0.05 | -0.04 | 0.02 | 0.00 |
| Q9JHI5 | IVD | 46.33 | 0.34 | 0.38 | 0.36 | 0.03 | 0.13 | 0.22 | 0.17 | 0.07 | 0.19 |
| Q9Z0R4 | ITSN1 | 194.29 | -0.64 | -0.09 | -0.36 | 0.39 | 0.79 | 0.02 | 0.41 | 0.55 | -0.77 |
| Q9Z329 | ITPR2 | 307.47 | -0.39 | -0.27 | -0.33 | 0.08 | -0.22 | -0.26 | -0.24 | 0.02 | -0.09 |
| A6X935 | ITIH4 | 104.66 | 0.42 | 0.74 | 0.58 | 0.23 | 0.18 | 0.48 | 0.33 | 0.21 | 0.25 |
| Q61704 | ITIH3 | 99.36 | 0.75 | 0.82 | 0.79 | 0.05 | 0.48 | 0.77 | 0.63 | 0.20 | 0.16 |
| Q61703 | ITIH2 | 105.93 | 0.31 | 0.44 | 0.37 | 0.09 | -0.03 | 0.25 | 0.11 | 0.20 | 0.26 |
| Q61702 | ITIH1 | 101.07 | 0.33 | 0.32 | 0.32 | 0.01 | -0.14 | 0.31 | 0.09 | 0.32 | 0.24 |
| P09055 | ITGB1 | 88.23 | -0.41 | -0.10 | -0.25 | 0.22 | -0.05 | -0.04 | -0.05 | 0.01 | -0.20 |
| P43406 | ITGAV | 115.36 | -0.49 | -0.41 | -0.45 | 0.06 | -0.51 | -0.44 | -0.47 | 0.05 | 0.02 |

| Q3V3R4 | ITGA1 | 130.81 | -0.46 | -0.32 | -0.39 | 0.10 | -0.24 | -0.18 | -0.21 | 0.04 | -0.18 |
| --- | --- | --- | --- | --- | --- | --- | --- | --- | --- | --- | --- |
| Q8C0Z1 | ITFG3 | 60.58 | 0.67 | 0.55 | 0.61 | 0.08 | 0.32 | 0.53 | 0.43 | 0.15 | 0.19 |
| Q9CX00 | IST1 | 39.47 | -0.07 | -0.19 | -0.13 | 0.08 | -0.02 | -0.01 | -0.01 | 0.01 | -0.12 |
| Q9DCC7 | ISOC2B | 23.15 | -0.08 | 0.09 | 0.00 | 0.12 | -0.35 | -0.28 | -0.32 | 0.05 | 0.32 |
| Q91V64 | ISOC1 | 32.03 | -0.23 | -0.31 | -0.27 | 0.05 | -0.32 | -0.33 | -0.33 | 0.00 | 0.06 |
| Q60766 | IRGM1 | 46.55 | -0.38 | -0.43 | -0.40 | 0.04 | -0.40 | -0.38 | -0.39 | 0.02 | -0.01 |
| P70671 | IRF3 | 46.85 | -0.02 | -0.09 | -0.05 | 0.05 | -0.13 | -0.07 | -0.10 | 0.04 | 0.05 |
| Q3UQ44 | IQGAP2 | 180.53 | -0.03 | 0.21 | 0.09 | 0.17 | 0.09 | 0.25 | 0.17 | 0.12 | -0.07 |
| Q9JKF1 | IQGAP1 | 188.74 | -0.36 | -0.45 | -0.40 | 0.06 | -0.18 | -0.43 | -0.31 | 0.18 | -0.10 |
| Q91YE6 | IPO9 | 116.05 | -0.39 | -0.53 | -0.46 | 0.10 | -0.30 | -0.44 | -0.37 | 0.10 | -0.09 |
| Q9EPL8 | IPO7 | 119.49 | -0.35 | -0.45 | -0.40 | 0.07 | -0.35 | -0.11 | -0.23 | 0.17 | -0.17 |
| Q8BKC5 | IPO5 | 123.59 | -0.13 | -0.15 | -0.14 | 0.01 | -0.15 | -0.25 | -0.20 | 0.07 | 0.06 |
| Q6PCM2 | INTS6 | 99.66 | -0.36 | -0.12 | -0.24 | 0.17 | -0.39 | -0.16 | -0.28 | 0.16 | 0.04 |

| P15208 | INSR | 155.61 | 0.17 | 0.26 | 0.21 | 0.07 | -0.34 | 0.20 | -0.07 | 0.38 | 0.29 |
| --- | --- | --- | --- | --- | --- | --- | --- | --- | --- | --- | --- |
| P49442 | INPP1 | 43.35 | -0.52 | -0.46 | -0.49 | 0.04 | -0.48 | -0.44 | -0.46 | 0.02 | -0.03 |
| P40936 | INMT | 29.46 | -0.74 | -0.84 | -0.79 | 0.07 | -0.75 | -0.82 | -0.79 | 0.05 | 0.00 |
| P24547 | IMPDH2 | 55.81 | -0.63 | -0.60 | -0.61 | 0.02 | -0.56 | -0.55 | -0.56 | 0.01 | -0.06 |
| Q80V26 | IMPAD1 | 38.62 | -0.17 | -0.13 | -0.15 | 0.03 | 0.16 | 0.15 | 0.15 | 0.00 | -0.30 |
| O55023 | IMPA1 | 30.44 | 0.24 | 0.20 | 0.22 | 0.03 | 0.13 | 0.23 | 0.18 | 0.07 | 0.04 |
| Q8CAQ8 | IMMT | 83.90 | 0.04 | 0.07 | 0.06 | 0.02 | -0.17 | 0.00 | -0.08 | 0.12 | 0.14 |
| Q8BU33 | ILVBL | 68.16 | -0.11 | -0.05 | -0.08 | 0.04 | -0.31 | -0.16 | -0.24 | 0.11 | 0.15 |
| O55222 | ILK | 51.37 | 0.12 | 0.16 | 0.14 | 0.02 | 0.24 | 0.19 | 0.21 | 0.03 | -0.08 |
| Q9Z1X4 | ILF3 | 96.02 | 0.02 | -0.11 | -0.04 | 0.09 | 0.14 | -0.05 | 0.05 | 0.13 | -0.09 |
| Q9CXY6 | ILF2 | 43.06 | -0.17 | -0.08 | -0.12 | 0.06 | -0.14 | -0.01 | -0.08 | 0.09 | -0.05 |
| Q9Z1M8 | IK | 65.62 | -0.24 | -0.29 | -0.26 | 0.04 | -0.07 | -0.13 | -0.10 | 0.04 | -0.17 |
| Q9QZ85 | IIGP1 | 47.57 | -1.04 | -1.16 | -1.10 | 0.08 | -1.14 | -1.17 | -1.15 | 0.03 | 0.05 |

| P01872 | IGHM | 49.97 | 3.43 | 3.43 | 3.43 | 0.00 | 2.66 | 2.41 | 2.54 | 0.18 | 0.89 |
| --- | --- | --- | --- | --- | --- | --- | --- | --- | --- | --- | --- |
| P01868 | IGHG1 | 35.70 | 1.82 | 1.99 | 1.90 | 0.12 | 3.41 | 3.51 | 3.46 | 0.07 | -1.56 |
| P01867 | IGH-3 | 44.26 | 3.06 | 2.96 | 3.01 | 0.07 | 2.51 | 2.31 | 2.41 | 0.14 | 0.60 |
| P47877 | IGFBP2 | 32.85 | -0.03 | -0.03 | -0.03 | 0.00 | 0.04 | 0.37 | 0.20 | 0.23 | -0.23 |
| Q07113 | IGF2R | 273.81 | 0.24 | -0.34 | -0.05 | 0.41 | -0.04 | -0.20 | -0.12 | 0.11 | 0.07 |
| Q61249 | IGBP1 | 38.97 | -0.60 | -0.31 | -0.45 | 0.20 | -0.45 | -0.28 | -0.37 | 0.12 | -0.09 |
| Q8R5F7 | IFIH1 | 115.97 | -0.73 | -0.82 | -0.78 | 0.06 | -0.54 | -0.71 | -0.62 | 0.12 | -0.15 |
| Q9D8C4 | IFI35 | 31.88 | -0.38 | -0.43 | -0.41 | 0.04 | -0.23 | -0.27 | -0.25 | 0.03 | -0.16 |
| Q8R0J8 | IDNK | 19.98 | -0.02 | -0.13 | -0.08 | 0.08 | -0.22 | -0.11 | -0.17 | 0.08 | 0.09 |
| P58044 | IDI1 | 26.29 | -0.64 | -0.55 | -0.59 | 0.06 | -0.10 | -0.06 | -0.08 | 0.03 | -0.51 |
| P70404 | IDH3G | 42.79 | -0.35 | -0.45 | -0.40 | 0.07 | -0.15 | -0.31 | -0.23 | 0.11 | -0.17 |
| Q9D6R2 | IDH3A | 39.64 | -0.42 | -0.42 | -0.42 | 0.00 | -0.42 | -0.27 | -0.35 | 0.11 | -0.08 |
| P54071 | IDH2 | 50.91 | -0.64 | -0.57 | -0.60 | 0.05 | -0.53 | -0.49 | -0.51 | 0.03 | -0.09 |

| O88844 | IDH1 | 46.67 | -0.04 | -0.06 | -0.05 | 0.02 | -0.22 | -0.16 | -0.19 | 0.04 | 0.14 |
| --- | --- | --- | --- | --- | --- | --- | --- | --- | --- | --- | --- |
| Q9JHR7 | IDE | 117.77 | 0.08 | -0.04 | 0.02 | 0.09 | 0.09 | -0.01 | 0.04 | 0.07 | -0.02 |
| Q9DBD0 | ICA | 76.77 | 0.30 | 0.19 | 0.24 | 0.08 | -0.14 | 0.07 | -0.04 | 0.14 | 0.28 |
| Q8BIJ6 | IARS2 | 112.80 | 0.27 | 0.16 | 0.22 | 0.08 | 0.24 | 0.22 | 0.23 | 0.01 | -0.02 |
| Q8BU30 | IARS | 144.27 | -0.44 | 0.01 | -0.21 | 0.31 | -0.56 | 0.04 | -0.26 | 0.43 | 0.05 |
| Q9DB29 | IAH1 | 27.97 | -0.25 | -0.21 | -0.23 | 0.03 | -0.18 | -0.08 | -0.13 | 0.07 | -0.10 |
| Q9JKR6 | HYOU1 | 111.18 | -0.42 | 0.09 | -0.17 | 0.36 | -0.38 | 0.07 | -0.16 | 0.32 | -0.01 |
| Q5U5V2 | HYKK | 42.36 | -0.16 | -0.11 | -0.13 | 0.03 | -0.26 | -0.10 | -0.18 | 0.11 | 0.05 |
| Q7TMY8 | HUWE1 | 482.63 | -0.08 | -0.01 | -0.04 | 0.05 | -0.03 | -0.11 | -0.07 | 0.05 | 0.03 |
| Q8BGC0 | HTATSF1 | 86.24 | -0.01 | -0.10 | -0.05 | 0.06 | 0.05 | -0.10 | -0.02 | 0.11 | -0.03 |
| Q9Z2G9 | HTATIP2 | 26.87 | 0.23 | 0.11 | 0.17 | 0.08 | 0.16 | 0.01 | 0.09 | 0.11 | 0.08 |
| Q61699 | HSPH1 | 96.41 | 0.02 | -0.04 | -0.01 | 0.04 | -0.19 | -0.27 | -0.23 | 0.06 | 0.23 |
| Q05793 | HSPG2 | 398.29 | -0.33 | 0.14 | -0.10 | 0.34 | -0.18 | 0.08 | -0.05 | 0.18 | -0.05 |

| Q64433 | HSPE1 | 10.96 | -0.03 | 0.03 | 0.00 | 0.05 | -0.10 | 0.02 | -0.04 | 0.08 | 0.04 |
| --- | --- | --- | --- | --- | --- | --- | --- | --- | --- | --- | --- |
| P63038 | HSPD1 | 60.96 | 0.16 | 0.22 | 0.19 | 0.04 | 0.17 | 0.23 | 0.20 | 0.04 | -0.01 |
| P38647 | HSPA9 | 73.46 | 0.15 | 0.05 | 0.10 | 0.07 | 0.14 | 0.05 | 0.10 | 0.06 | 0.00 |
| P63017 | HSPA8 | 70.87 | -0.07 | -0.10 | -0.08 | 0.03 | -0.14 | -0.21 | -0.18 | 0.05 | 0.09 |
| P20029 | HSPA5 | 72.42 | 0.01 | -0.04 | -0.02 | 0.04 | -0.03 | -0.07 | -0.05 | 0.03 | 0.03 |
| P48722 | HSPA4L | 94.38 | -0.16 | -0.33 | -0.25 | 0.12 | -0.42 | -0.41 | -0.42 | 0.01 | 0.17 |
| Q61316 | HSPA4 | 94.13 | 0.09 | 0.01 | 0.05 | 0.06 | 0.04 | 0.02 | 0.03 | 0.01 | 0.02 |
|  |  | 70.08 | 0.64 | 0.58 | 0.61 | 0.04 | 0.20 | 0.12 | 0.16 | 0.05 | 0.45 |
| Q8BM72 | HSPA13 | 51.71 | 0.04 | -0.13 | -0.04 | 0.12 | -0.14 | -0.29 | -0.21 | 0.11 | 0.17 |
| P08113 | HSP90B1 | 92.48 | -0.17 | -0.18 | -0.18 | 0.01 | -0.10 | -0.13 | -0.12 | 0.02 | -0.06 |
| P11499 | HSP90AB1 | 83.28 | -0.26 | -0.28 | -0.27 | 0.02 | -0.29 | -0.34 | -0.31 | 0.03 | 0.04 |
| P07901 | HSP90AA1 | 84.79 | -0.10 | -0.14 | -0.12 | 0.03 | -0.20 | -0.27 | -0.24 | 0.05 | 0.12 |
| Q2TPA8 | HSDL2 | 54.21 | -0.13 | 0.08 | -0.02 | 0.14 | -0.28 | -0.12 | -0.20 | 0.11 | 0.18 |

| Q61694 | HSD3B5 | 41.89 | 5.04 | 5.05 | 5.05 | 0.00 | 4.87 | 4.97 | 4.92 | 0.07 | 0.13 |
| --- | --- | --- | --- | --- | --- | --- | --- | --- | --- | --- | --- |
|  |  | 42.03 | -0.17 | -0.18 | -0.18 | 0.01 | -0.31 | -0.30 | -0.30 | 0.01 | 0.12 |
| O88736 | HSD17B7 | 37.32 | 0.26 | 0.39 | 0.33 | 0.10 | 0.24 | 0.34 | 0.29 | 0.08 | 0.04 |
| Q9R092 | HSD17B6 | 36.10 | -1.11 | -0.21 | -0.66 | 0.64 | -0.48 | -0.22 | -0.35 | 0.19 | -0.31 |
| P51660 | HSD17B4 | 79.48 | 0.50 | 0.43 | 0.47 | 0.05 | 0.21 | 0.23 | 0.22 | 0.01 | 0.24 |
| P51658 | HSD17B2 | 41.84 | 0.12 | -0.09 | 0.01 | 0.15 | -0.01 | -0.03 | -0.02 | 0.01 | 0.04 |
| Q8VCR2 | HSD17B13 | 33.46 | 0.38 | 0.34 | 0.36 | 0.03 | 0.51 | 0.48 | 0.49 | 0.02 | -0.14 |
| O70503 | HSD17B12 | 34.74 | 0.36 | 0.27 | 0.31 | 0.07 | 0.06 | 0.09 | 0.07 | 0.02 | 0.24 |
| Q9EQ06 | HSD17B11 | 32.88 | -0.07 | -0.15 | -0.11 | 0.06 | -0.32 | -0.29 | -0.31 | 0.02 | 0.20 |
| P50172 | HSD11B1 | 32.36 | -0.29 | -0.27 | -0.28 | 0.02 | -0.14 | -0.15 | -0.14 | 0.01 | -0.14 |
| P52760 | HRSP12 | 14.26 | 0.06 | 0.15 | 0.10 | 0.06 | 0.11 | 0.21 | 0.16 | 0.07 | -0.06 |
| Q91X72 | HPX | 51.32 | 1.04 | 1.10 | 1.07 | 0.04 | 1.00 | 1.01 | 1.00 | 0.00 | 0.07 |
| P00493 | HPRT1 | 24.57 | 0.62 | 0.71 | 0.66 | 0.06 | 0.60 | 0.60 | 0.60 | 0.00 | 0.06 |

| Q8VCC1 | HPGD | 29.18 | -1.04 | -0.89 | -0.96 | 0.11 | -0.85 | -0.73 | -0.79 | 0.08 | -0.17 |
| --- | --- | --- | --- | --- | --- | --- | --- | --- | --- | --- | --- |
| P49429 | HPD | 45.05 | -0.09 | -0.01 | -0.05 | 0.06 | -0.01 | 0.04 | 0.02 | 0.04 | -0.07 |
| Q3TEA8 | HP1BP3 | 60.87 | -0.21 | -0.17 | -0.19 | 0.03 | -0.01 | -0.13 | -0.07 | 0.08 | -0.12 |
| Q61646 | HP | 38.75 | 1.90 | 2.21 | 2.05 | 0.22 | 1.37 | 1.90 | 1.64 | 0.37 | 0.42 |
| Q8BUK6 | HOOK3 | 83.22 | -0.22 | -0.30 | -0.26 | 0.06 | -0.26 | -0.31 | -0.29 | 0.04 | 0.02 |
| Q8BIL5 | HOOK1 | 84.44 | -0.08 | 0.11 | 0.02 | 0.13 | -0.19 | 0.03 | -0.08 | 0.16 | 0.10 |
| Q9DCU9 | HOGA1 | 34.64 | 0.17 | 0.15 | 0.16 | 0.01 | 0.09 | 0.17 | 0.13 | 0.06 | 0.02 |
| Q00PI9 | HNRNPUL2 | 84.94 | -0.32 | -0.23 | -0.27 | 0.06 | -0.14 | -0.18 | -0.16 | 0.03 | -0.11 |
| Q8VEK3 | HNRNPU | 87.92 | -0.37 | -0.19 | -0.28 | 0.13 | -0.25 | -0.18 | -0.22 | 0.05 | -0.07 |
| Q9D0E1 | HNRNPM | 77.65 | 0.03 | -0.09 | -0.03 | 0.09 | 0.28 | 0.02 | 0.15 | 0.18 | -0.18 |
| Q921F4 | HNRNPLL | 64.12 | -0.07 | -0.06 | -0.06 | 0.01 | 0.03 | -0.01 | 0.01 | 0.03 | -0.07 |
| Q8R081 | HNRNPL | 63.96 | -0.11 | -0.06 | -0.08 | 0.04 | 0.03 | 0.02 | 0.03 | 0.01 | -0.11 |
| P61979 | HNRNPK | 50.98 | 0.00 | -0.08 | -0.04 | 0.06 | 0.01 | -0.04 | -0.01 | 0.03 | -0.03 |

| P70333 |  | 49.28 | -0.19 | 0.12 | -0.03 | 0.21 | -0.03 | 0.23 | 0.10 | 0.19 | -0.14 |
| --- | --- | --- | --- | --- | --- | --- | --- | --- | --- | --- | --- |
| Q9Z2X1 | HNRNPF | 45.73 | -0.48 | -0.20 | -0.34 | 0.20 | -0.30 | -0.13 | -0.22 | 0.12 | -0.12 |
| Q9Z130 | HNRNPDL | 33.56 | 0.04 | -0.11 | -0.04 | 0.10 | 0.02 | -0.07 | -0.02 | 0.07 | -0.01 |
| Q60668 | HNRNPD | 38.35 | -0.09 | -0.16 | -0.12 | 0.05 | -0.03 | -0.05 | -0.04 | 0.01 | -0.08 |
| Q9Z204 | HNRNPC | 34.38 | -0.03 | -0.09 | -0.06 | 0.04 | -0.01 | -0.02 | -0.01 | 0.01 | -0.04 |
| Q99020 | HNRNPAB | 30.83 | -0.21 | -0.21 | -0.21 | 0.01 | -0.15 | -0.19 | -0.17 | 0.03 | -0.04 |
| Q8BG05 | HNRNPA3 | 39.65 | -0.08 | -0.15 | -0.11 | 0.05 | -0.07 | -0.10 | -0.09 | 0.02 | -0.03 |
| O88569 | HNRNPA2B1 | 37.40 | 0.08 | 0.01 | 0.05 | 0.05 | 0.14 | 0.05 | 0.09 | 0.06 | -0.05 |
| P49312 | HNRNPA1 | 34.20 | -0.19 | -0.14 | -0.16 | 0.03 | -0.03 | -0.12 | -0.08 | 0.06 | -0.09 |
| Q9CX86 | HNRNPA0 | 30.53 | -0.07 | -0.45 | -0.26 | 0.27 | -0.06 | -0.22 | -0.14 | 0.11 | -0.12 |
| Q91VF2 | HNMT | 33.66 | 0.64 | 0.61 | 0.63 | 0.02 | 0.60 | 0.58 | 0.59 | 0.01 | 0.04 |
| O70252 | HMOX2 | 35.74 | -0.19 | -0.25 | -0.22 | 0.04 | -0.08 | -0.12 | -0.10 | 0.03 | -0.12 |
| P14901 | HMOX1 | 32.93 | -0.12 | -0.07 | -0.10 | 0.04 | 0.06 | -0.30 | -0.12 | 0.26 | 0.02 |

| Q9JL35 | HMGN5 | 45.34 | -0.05 | -0.01 | -0.03 | 0.03 | 0.10 | 0.04 | 0.07 | 0.04 | -0.10 |
| --- | --- | --- | --- | --- | --- | --- | --- | --- | --- | --- | --- |
| P54869 | HMGCS2 | 56.82 | -0.15 | -0.29 | -0.22 | 0.10 | -0.38 | -0.51 | -0.44 | 0.10 | 0.22 |
| Q8JZK9 | HMGCS1 | 57.57 | -0.98 | -0.94 | -0.96 | 0.02 | -0.82 | -0.81 | -0.81 | 0.01 | -0.15 |
| P38060 | HMGCL | 34.24 | 0.08 | -0.11 | -0.01 | 0.14 | 0.19 | -0.08 | 0.06 | 0.19 | -0.07 |
| O54879 | HMGB3 | 23.01 | -0.47 | -0.49 | -0.48 | 0.02 | -0.57 | -0.43 | -0.50 | 0.10 | 0.02 |
| P30681 | HMGB2 | 24.16 | -1.10 | -1.10 | -1.10 | 0.00 | -0.87 | -1.13 | -1.00 | 0.18 | -0.10 |
| P63158 | HMGB1 | 24.89 | -0.44 | -0.37 | -0.40 | 0.05 | -0.32 | -0.26 | -0.29 | 0.05 | -0.11 |
| P22907 | HMBS | 39.34 | -0.40 | -0.19 | -0.29 | 0.15 | -0.12 | -0.20 | -0.16 | 0.06 | -0.14 |
| Q9D8V0 | HM13 | 41.75 | 0.00 | 0.02 | 0.01 | 0.01 | -0.23 | -0.16 | -0.19 | 0.04 | 0.20 |
| P17710 | HK1 | 108.30 | -0.06 | -0.09 | -0.07 | 0.02 | -0.20 | -0.12 | -0.16 | 0.06 | 0.09 |
| P62806 | HIST1H4A | 11.37 | -0.29 | -0.26 | -0.27 | 0.02 | -0.14 | -0.27 | -0.20 | 0.09 | -0.07 |
| P43274 | HIST1H1E | 21.98 | 0.22 | 0.22 | 0.22 | 0.00 | 0.51 | 0.33 | 0.42 | 0.12 | -0.20 |
| P43277 | HIST1H1D | 22.10 | -0.26 | -0.16 | -0.21 | 0.07 | -0.09 | -0.21 | -0.15 | 0.09 | -0.06 |

| P15864 | HIST1H1C | 21.27 | -0.39 | -0.25 | -0.32 | 0.10 | -0.09 | -0.34 | -0.22 | 0.18 | -0.10 |
| --- | --- | --- | --- | --- | --- | --- | --- | --- | --- | --- | --- |
| P43276 | HIST1H1B | 22.58 | -3.20 | -2.93 | -3.06 | 0.19 | -2.61 | -2.98 | -2.79 | 0.26 | -0.27 |
| P43275 | HIST1H1A | 21.79 | -1.79 | -1.70 | -1.75 | 0.06 | -1.37 | -1.77 | -1.57 | 0.29 | -0.17 |
| Q9JKY5 | HIP1R | 119.43 | 0.63 | 0.66 | 0.65 | 0.02 | 0.59 | 0.58 | 0.59 | 0.01 | 0.06 |
| Q9D0S9 | HINT2 | 17.32 | 0.53 | 0.61 | 0.57 | 0.06 | 0.42 | 0.76 | 0.59 | 0.24 | -0.02 |
| Q8QZS1 | HIBCH | 43.04 | -0.02 | 0.05 | 0.01 | 0.05 | -0.10 | 0.04 | -0.03 | 0.10 | 0.04 |
| Q99L13 | HIBADH | 35.44 | 0.28 | 0.33 | 0.30 | 0.04 | 0.28 | 0.34 | 0.31 | 0.04 | -0.01 |
| Q99LI8 | HGS | 86.01 | -0.52 | -0.02 | -0.27 | 0.35 | -0.62 | 0.03 | -0.29 | 0.46 | 0.03 |
| O09173 | HGD | 49.96 | 0.04 | 0.03 | 0.04 | 0.00 | 0.01 | 0.04 | 0.03 | 0.01 | 0.01 |
| P20060 | HEXB | 61.12 | 0.14 | 0.12 | 0.13 | 0.02 | 0.27 | 0.14 | 0.20 | 0.09 | -0.08 |
| Q6PAV2 | HERC4 | 118.41 | 0.67 | 0.23 | 0.45 | 0.31 | 0.66 | 0.19 | 0.42 | 0.33 | 0.03 |
| Q3U487 | HECTD3 | 97.35 | 0.12 | -0.09 | 0.02 | 0.14 | -0.29 | -0.16 | -0.23 | 0.09 | 0.24 |
| Q69ZR2 | HECTD1 | 290.08 | 0.00 | 0.31 | 0.16 | 0.22 | 0.33 | 0.38 | 0.35 | 0.03 | -0.19 |

| Q9R257 | HEBP1 | 21.07 | -0.22 | -0.08 | -0.15 | 0.10 | 1.53 | 1.58 | 1.55 | 0.04 | -1.70 |
| --- | --- | --- | --- | --- | --- | --- | --- | --- | --- | --- | --- |
| Q8VDJ3 | HDLBP | 141.74 | -0.35 | -0.01 | -0.18 | 0.24 | -0.30 | 0.07 | -0.12 | 0.26 | -0.07 |
| Q9CYW4 | HDHD3 | 28.03 | -0.02 | 0.08 | 0.03 | 0.07 | 0.24 | 0.34 | 0.29 | 0.07 | -0.26 |
| Q3UGR5 | HDHD2 | 28.73 | -0.23 | -0.23 | -0.23 | 0.00 | -0.36 | -0.34 | -0.35 | 0.01 | 0.12 |
| P51859 | HDGF | 26.27 | -0.17 | -0.02 | -0.09 | 0.11 | -0.29 | 0.00 | -0.15 | 0.20 | 0.05 |
| O09106 |  | 55.07 | -0.20 | -0.20 | -0.20 | 0.00 | -0.13 | -0.08 | -0.10 | 0.03 | -0.09 |
| P49710 | HCLS1 | 54.24 | -0.75 | -0.46 | -0.61 | 0.20 | -0.24 | -0.50 | -0.37 | 0.18 | -0.24 |
| Q61191 | HCFC1 | 210.43 | -1.03 | -0.67 | -0.85 | 0.25 | -0.46 | -0.20 | -0.33 | 0.18 | -0.52 |
| P02088 |  | 15.84 | 0.33 | 0.33 | 0.33 | 0.00 | 0.29 | 0.18 | 0.23 | 0.07 | 0.10 |
| P01942 | HBA | 15.09 | 0.41 | 0.32 | 0.37 | 0.06 | 0.40 | 0.21 | 0.30 | 0.13 | 0.06 |
| Q61035 | HARS | 57.43 | -0.14 | -0.16 | -0.15 | 0.01 | -0.13 | -0.11 | -0.12 | 0.02 | -0.04 |
| Q9WU19 | HAO1 | 41.00 | -0.44 | -0.46 | -0.45 | 0.01 | -0.07 | -0.05 | -0.06 | 0.01 | -0.39 |
| P35492 | HAL | 72.26 | 0.16 | 0.10 | 0.13 | 0.04 | 0.30 | 0.26 | 0.28 | 0.03 | -0.15 |

| Q99KB8 | HAGH | 34.08 | 0.00 | 0.04 | 0.02 | 0.02 | -0.05 | 0.04 | -0.01 | 0.06 | 0.03 |
| --- | --- | --- | --- | --- | --- | --- | --- | --- | --- | --- | --- |
| Q99JY0 | HADHB | 51.39 | -0.01 | -0.05 | -0.03 | 0.03 | -0.11 | -0.10 | -0.11 | 0.01 | 0.08 |
| Q8BMS1 | HADHA | 82.67 | 0.05 | -0.06 | -0.01 | 0.08 | -0.03 | -0.09 | -0.06 | 0.04 | 0.05 |
| Q61425 | HADH | 34.46 | -0.22 | -0.22 | -0.22 | 0.00 | -0.26 | -0.28 | -0.27 | 0.02 | 0.05 |
| Q9QXE0 | HACL1 | 63.66 | -0.51 | -0.53 | -0.52 | 0.02 | -0.54 | -0.50 | -0.52 | 0.03 | 0.00 |
| Q8K2C9 | HACD3 | 43.13 | -0.02 | 0.00 | -0.01 | 0.02 | -0.11 | -0.03 | -0.07 | 0.05 | 0.06 |
| Q9D3B1 | HACD2 | 28.40 | -0.37 | -0.21 | -0.29 | 0.12 | -0.49 | -0.19 | -0.34 | 0.21 | 0.05 |
| Q78JT3 | HAAO | 32.80 | 0.27 | 0.21 | 0.24 | 0.04 | 0.12 | 0.18 | 0.15 | 0.04 | 0.09 |
| Q8CFX1 | H6PD | 88.93 | 0.46 | 0.35 | 0.41 | 0.07 | 0.57 | 0.41 | 0.49 | 0.11 | -0.08 |
| P14428 | H2-K1 | 36.86 | -0.08 | -0.21 | -0.15 | 0.09 | -0.14 | -0.22 | -0.18 | 0.06 | 0.03 |
| P01899 | H2-D1;H2-L | 40.84 | -0.17 | -0.09 | -0.13 | 0.05 | -0.19 | -0.33 | -0.26 | 0.09 | 0.13 |
| Q9QZQ8 | H2AFY | 39.74 | 0.06 | 0.09 | 0.08 | 0.01 | 0.12 | 0.13 | 0.12 | 0.01 | -0.05 |
| P10922 | H1F0 | 20.86 | 0.16 | 0.25 | 0.20 | 0.06 | 0.38 | 0.35 | 0.37 | 0.02 | -0.17 |

| Q8VCB3 | GYS2 | 80.87 | -0.53 | -0.57 | -0.55 | 0.03 | -0.38 | -0.49 | -0.44 | 0.07 | -0.11 |
| --- | --- | --- | --- | --- | --- | --- | --- | --- | --- | --- | --- |
| P12265 | GUSB | 74.19 | 0.88 | 0.68 | 0.78 | 0.14 | 0.77 | 0.61 | 0.69 | 0.11 | 0.09 |
| P58710 | GULO | 50.48 | 0.22 | 0.06 | 0.14 | 0.11 | 0.16 | 0.10 | 0.13 | 0.04 | 0.01 |
| Q9ERL9 | GUCY1A3 | 77.59 | -0.37 | -0.71 | -0.54 | 0.23 | -0.48 | -0.56 | -0.52 | 0.06 | -0.02 |
| Q99ME9 | GTPBP4 | 74.11 | -0.03 | -0.16 | -0.09 | 0.09 | 0.03 | -0.12 | -0.05 | 0.10 | -0.05 |
| Q8BMQ2 | GTF3C4 | 91.61 | -0.01 | -0.25 | -0.13 | 0.16 | 0.07 | -0.02 | 0.03 | 0.07 | -0.16 |
| Q9ESZ8 | GTF2I | 112.26 | 0.26 | -0.45 | -0.09 | 0.50 | 0.52 | -0.34 | 0.09 | 0.61 | -0.19 |
| Q9WVL0 | GSTZ1 | 24.28 | -0.03 | 0.09 | 0.03 | 0.08 | -0.01 | 0.06 | 0.03 | 0.05 | 0.00 |
| Q61133 | GSTT2 | 27.63 | 0.15 | 0.27 | 0.21 | 0.08 | 0.47 | 0.52 | 0.49 | 0.03 | -0.28 |
| Q64471 | GSTT1 | 27.37 | -0.50 | -0.41 | -0.46 | 0.07 | -0.50 | -0.46 | -0.48 | 0.03 | 0.02 |
| P19157 | GSTP1 | 23.61 | 3.63 | 3.62 | 3.62 | 0.01 | 3.43 | 3.60 | 3.52 | 0.12 | 0.11 |
| O09131 | GSTO1 | 27.50 | -0.12 | -0.14 | -0.13 | 0.01 | -0.13 | -0.13 | -0.13 | 0.00 | 0.00 |
| Q80W21 | GSTM7 | 25.71 | -0.14 | -0.11 | -0.13 | 0.02 | -0.08 | -0.09 | -0.08 | 0.00 | -0.04 |

| O35660 | GSTM6 | 25.62 | 0.24 | 0.17 | 0.20 | 0.05 | 0.10 | 0.18 | 0.14 | 0.06 | 0.06 |
| --- | --- | --- | --- | --- | --- | --- | --- | --- | --- | --- | --- |
| P19639 | GSTM3 | 25.70 | -0.01 | -0.03 | -0.02 | 0.01 | 0.40 | 0.23 | 0.31 | 0.12 | -0.34 |
| P15626 | GSTM2 | 25.72 | 0.04 | 0.19 | 0.12 | 0.11 | 0.41 | 0.08 | 0.25 | 0.23 | -0.13 |
| P10649 | GSTM1 | 25.97 | 0.58 | 0.58 | 0.58 | 0.00 | 0.64 | 0.66 | 0.65 | 0.01 | -0.07 |
| Q9DCM2 | GSTK1 | 25.70 | 0.44 | 0.48 | 0.46 | 0.03 | 0.46 | 0.50 | 0.48 | 0.02 | -0.02 |
| P24472 | GSTA4 | 25.56 | 0.41 | 0.47 | 0.44 | 0.04 | 0.59 | 0.63 | 0.61 | 0.03 | -0.17 |
| P30115 | GSTA3 | 25.36 | 0.19 | 0.21 | 0.20 | 0.02 | 0.11 | 0.22 | 0.16 | 0.08 | 0.04 |
| P10648 | GSTA2 | 25.54 | 2.80 | 2.79 | 2.80 | 0.01 | 3.10 | 3.01 | 3.05 | 0.06 | -0.26 |
| P51855 | GSS | 52.25 | -0.24 | -0.22 | -0.23 | 0.01 | -0.08 | -0.11 | -0.09 | 0.02 | -0.14 |
| P47791 | GSR | 53.66 | 0.31 | 0.36 | 0.33 | 0.04 | 0.38 | 0.44 | 0.41 | 0.04 | -0.08 |
| Q8R050 |  | 68.63 | -0.09 | -0.05 | -0.07 | 0.02 | 0.02 | 0.00 | 0.01 | 0.02 | -0.08 |
| Q9D8T2 | GSDMDC1 | 53.24 | -0.13 | 0.00 | -0.07 | 0.09 | 0.17 | 0.14 | 0.15 | 0.02 | -0.22 |
| Q99LP6 | GRPEL1 | 24.31 | -0.05 | 0.03 | -0.01 | 0.05 | -0.03 | 0.09 | 0.03 | 0.08 | -0.04 |

| Q8VD04 | GRIPAP1 | 92.71 | 0.08 | 0.16 | 0.12 | 0.06 | 0.13 | 0.31 | 0.22 | 0.13 | -0.10 |
| --- | --- | --- | --- | --- | --- | --- | --- | --- | --- | --- | --- |
| P11352 | GPX1 | 22.33 | 1.05 | 1.19 | 1.12 | 0.10 | 1.11 | 1.26 | 1.19 | 0.10 | -0.06 |
| Q8BGT5 | GPT2 | 57.94 | 1.16 | 1.06 | 1.11 | 0.07 | 0.94 | 0.93 | 0.94 | 0.01 | 0.18 |
| Q8QZR5 | GPT | 55.14 | -0.04 | 0.05 | 0.00 | 0.06 | 0.02 | 0.20 | 0.11 | 0.12 | -0.11 |
| Q99LD4 | GPS1 | 53.44 | -0.20 | -0.05 | -0.12 | 0.10 | -0.10 | -0.05 | -0.07 | 0.03 | -0.05 |
| P06745 | GPI | 62.77 | -1.43 | -1.36 | -1.39 | 0.05 | -1.43 | -1.45 | -1.44 | 0.01 | 0.05 |
| Q8BUV3 | GPHN | 83.28 | 0.12 | 0.36 | 0.24 | 0.17 | 0.10 | 0.38 | 0.24 | 0.20 | 0.00 |
| Q64521 | GPD2 | 80.95 | -1.10 | -1.09 | -1.09 | 0.00 | -1.24 | -1.26 | -1.25 | 0.02 | 0.15 |
| Q3ULJ0 | GPD1L | 38.23 | -0.20 | -0.29 | -0.24 | 0.06 | -0.37 | -0.29 | -0.33 | 0.06 | 0.09 |
| P13707 | GPD1 | 37.57 | 0.16 | 0.12 | 0.14 | 0.03 | -0.16 | -0.18 | -0.17 | 0.01 | 0.31 |
| Q8C0L9 | GPCPD1 | 76.58 | 2.10 | 1.92 | 2.01 | 0.13 | 2.02 | 1.78 | 1.90 | 0.17 | 0.11 |
| P05202 | GOT2 | 47.41 | 0.28 | 0.13 | 0.20 | 0.10 | 0.07 | 0.17 | 0.12 | 0.07 | 0.08 |
| P05201 | GOT1 | 46.25 | -0.02 | -0.04 | -0.03 | 0.02 | 0.15 | 0.27 | 0.21 | 0.09 | -0.24 |

| Q99JX3 | GORASP2 | 47.04 | -0.25 | -0.02 | -0.13 | 0.16 | -0.11 | -0.04 | -0.08 | 0.05 | -0.06 |
| --- | --- | --- | --- | --- | --- | --- | --- | --- | --- | --- | --- |
| Q9CRA5 | GOLPH3 | 33.75 | -0.01 | 0.26 | 0.12 | 0.19 | 0.19 | 0.40 | 0.29 | 0.15 | -0.17 |
| Q8BXA1 | GOLIM4 | 76.78 | -0.26 | -0.03 | -0.14 | 0.16 | -0.10 | -0.05 | -0.08 | 0.03 | -0.07 |
| Q91VW5 | GOLGA4 | 257.56 | -0.08 | 0.04 | -0.02 | 0.08 | 0.08 | 0.15 | 0.12 | 0.05 | -0.13 |
| P55937 | GOLGA3 | 167.22 | -0.40 | 0.01 | -0.20 | 0.29 | -0.11 | 0.16 | 0.03 | 0.19 | -0.22 |
| Q921M4 | GOLGA2 | 113.28 | -0.48 | 0.02 | -0.23 | 0.35 | -0.03 | 0.09 | 0.03 | 0.09 | -0.26 |
| Q9JK38 | GNPNAT1 | 20.79 | -0.11 | -0.07 | -0.09 | 0.03 | 0.01 | 0.00 | 0.00 | 0.01 | -0.09 |
| O88958 | GNPDA1 | 32.55 | -0.06 | -0.26 | -0.16 | 0.14 | 0.02 | -0.04 | -0.01 | 0.04 | -0.15 |
| Q9QXF8 | GNMT | 32.68 | 0.72 | 0.63 | 0.67 | 0.06 | 0.73 | 0.75 | 0.74 | 0.01 | -0.07 |
| Q91WG8 | GNE | 79.20 | 0.30 | 0.23 | 0.27 | 0.05 | 0.48 | 0.47 | 0.48 | 0.00 | -0.21 |
| P68040 | GNB2L1 | 35.08 | 0.03 | 0.08 | 0.06 | 0.03 | 0.07 | 0.05 | 0.06 | 0.02 | 0.00 |
| P21279 | GNAQ | 42.16 | -0.03 | 0.07 | 0.02 | 0.07 | 0.00 | 0.08 | 0.04 | 0.06 | -0.02 |
| P08752 | GNAI2 | 40.49 | 0.03 | -0.07 | -0.02 | 0.07 | 0.02 | -0.09 | -0.03 | 0.08 | 0.01 |

| P27601 | GNA13 | 44.05 | 0.28 | 0.16 | 0.22 | 0.08 | 0.23 | 0.25 | 0.24 | 0.01 | -0.02 |
| --- | --- | --- | --- | --- | --- | --- | --- | --- | --- | --- | --- |
| P21278 | GNA11 | 42.02 | 0.09 | 0.04 | 0.06 | 0.04 | 0.03 | 0.07 | 0.05 | 0.03 | 0.01 |
| Q3THK7 | GMPS | 76.72 | -0.10 | -0.14 | -0.12 | 0.03 | -0.01 | -0.06 | -0.04 | 0.03 | -0.08 |
| Q5FW57 | GM4952 | 34.17 | -0.28 | -0.29 | -0.29 | 0.00 | -0.41 | -0.39 | -0.40 | 0.01 | 0.11 |
| Q60648 | GM2A | 20.82 | -0.55 | -0.28 | -0.41 | 0.19 | -0.34 | -0.07 | -0.20 | 0.19 | -0.21 |
| Q8QZY2 | GLYCTK | 55.29 | 0.25 | 0.30 | 0.27 | 0.03 | -0.18 | -0.07 | -0.13 | 0.08 | 0.40 |
| Q91XE0 | GLYAT | 34.10 | 1.00 | 0.91 | 0.96 | 0.06 | 0.97 | 0.91 | 0.94 | 0.04 | 0.02 |
| P15105 | GLUL | 42.12 | -0.02 | -0.06 | -0.04 | 0.03 | -0.14 | -0.12 | -0.13 | 0.01 | 0.09 |
| P26443 | GLUD1 | 61.34 | 0.07 | 0.16 | 0.11 | 0.06 | 0.07 | 0.11 | 0.09 | 0.03 | 0.02 |
| Q571F8 | GLS2 | 66.37 | 0.13 | 0.20 | 0.16 | 0.05 | 0.11 | 0.15 | 0.13 | 0.03 | 0.03 |
| Q9CQM9 | GLRX3 | 37.78 | -0.47 | -0.46 | -0.47 | 0.01 | -0.57 | -0.48 | -0.52 | 0.06 | 0.06 |
| Q9CPV4 | GLOD4 | 33.32 | 0.00 | 0.06 | 0.03 | 0.04 | 0.04 | 0.07 | 0.06 | 0.02 | -0.03 |
| Q9CPU0 | GLO1 | 20.81 | 1.11 | 1.19 | 1.15 | 0.05 | 0.81 | 0.88 | 0.85 | 0.05 | 0.30 |

| Q91W43 | GLDC | 113.27 | -0.25 | -0.18 | -0.22 | 0.05 | -0.18 | -0.20 | -0.19 | 0.02 | -0.03 |
| --- | --- | --- | --- | --- | --- | --- | --- | --- | --- | --- | --- |
| Q64516 | GK | 61.23 | -0.20 | -0.23 | -0.22 | 0.02 | -0.35 | -0.27 | -0.31 | 0.05 | 0.09 |
| P28230 | GJB1 | 32.00 | -0.56 | -0.27 | -0.42 | 0.20 | -0.62 | -0.30 | -0.46 | 0.23 | 0.04 |
| Q6Y7W8 | GIGYF2 | 149.19 | -0.33 | 0.04 | -0.14 | 0.26 | -0.40 | 0.07 | -0.16 | 0.33 | 0.02 |
| Q9QYC7 | GGCX | 87.19 | 0.13 | -0.04 | 0.04 | 0.12 | 0.40 | 0.11 | 0.26 | 0.21 | -0.21 |
| Q8R0H9 | GGA1 | 69.97 | -0.63 | -0.34 | -0.49 | 0.20 | -0.63 | -0.25 | -0.44 | 0.27 | -0.05 |
| P47856 | GFPT1 | 78.54 | -0.52 | -0.35 | -0.43 | 0.12 | -0.29 | -0.26 | -0.27 | 0.03 | -0.16 |
| Q8R2Q4 | GFM2 | 86.11 | 0.08 | 0.12 | 0.10 | 0.02 | -0.22 | 0.05 | -0.09 | 0.19 | 0.19 |
| Q8K0D5 | GFM1 | 83.55 | -0.14 | -0.16 | -0.15 | 0.02 | -0.26 | -0.23 | -0.24 | 0.02 | 0.10 |
| Q61598 | GDI2 | 50.54 | 0.04 | -0.02 | 0.01 | 0.04 | 0.00 | -0.05 | -0.02 | 0.04 | 0.03 |
| P50396 | GDI1 | 50.52 | -0.04 | 0.06 | 0.01 | 0.07 | -0.02 | 0.01 | 0.00 | 0.02 | 0.01 |
| Q9R111 | GDA | 51.01 | 0.52 | 0.34 | 0.43 | 0.13 | 0.39 | 0.31 | 0.35 | 0.06 | 0.08 |
| O09172 | GCLM | 30.53 | -0.05 | -0.09 | -0.07 | 0.03 | -0.05 | -0.05 | -0.05 | 0.00 | -0.02 |

| P97494 | GCLC | 72.57 | -0.05 | -0.16 | -0.11 | 0.08 | -0.19 | -0.22 | -0.20 | 0.02 | 0.10 |
| --- | --- | --- | --- | --- | --- | --- | --- | --- | --- | --- | --- |
| Q91X44 | GCKR | 64.68 | -0.56 | -0.49 | -0.52 | 0.06 | -0.39 | -0.43 | -0.41 | 0.02 | -0.11 |
| P52792 | GCK | 52.09 | 0.33 | 0.46 | 0.40 | 0.10 | 0.39 | 0.47 | 0.43 | 0.06 | -0.03 |
| Q05915 | GCH1 | 27.01 | -0.23 | -0.20 | -0.22 | 0.03 | -0.29 | -0.27 | -0.28 | 0.02 | 0.07 |
| Q60759 | GCDH | 48.61 | 0.15 | 0.36 | 0.26 | 0.14 | 0.06 | 0.20 | 0.13 | 0.10 | 0.12 |
| Q8CHG3 | GCC2 | 194.44 | 0.18 | 0.15 | 0.17 | 0.03 | 0.13 | 0.23 | 0.18 | 0.07 | -0.01 |
| O88986 | GCAT | 44.93 | 0.01 | 0.12 | 0.06 | 0.08 | 0.06 | 0.17 | 0.11 | 0.08 | -0.05 |
| P21614 | GC | 53.60 | 0.07 | 0.09 | 0.08 | 0.01 | -0.11 | -0.06 | -0.08 | 0.04 | 0.17 |
| Q9Z0E6 | GBP2 | 66.74 | -1.15 | -1.22 | -1.18 | 0.05 | -0.81 | -1.20 | -1.01 | 0.27 | -0.18 |
| Q9D6Y9 | GBE1 | 80.36 | 0.18 | 0.02 | 0.10 | 0.11 | 0.02 | -0.09 | -0.03 | 0.07 | 0.13 |
| O55126 | GBAS | 32.93 | -0.39 | -0.29 | -0.34 | 0.07 | -0.40 | -0.40 | -0.40 | 0.00 | 0.06 |
| P17439 | GBA | 57.62 | 1.01 | 1.09 | 1.05 | 0.06 | 1.09 | 0.90 | 0.99 | 0.13 | 0.06 |
| P11862 | GAS2 | 34.90 | 1.10 | 1.42 | 1.26 | 0.22 | 1.36 | 1.29 | 1.32 | 0.05 | -0.07 |

| Q64737 | GART | 107.50 | -0.43 | -0.32 | -0.37 | 0.08 | -0.21 | -0.16 | -0.19 | 0.04 | -0.19 |
| --- | --- | --- | --- | --- | --- | --- | --- | --- | --- | --- | --- |
| Q9CZD3 | GARS | 81.88 | -0.05 | -0.24 | -0.15 | 0.14 | 0.06 | -0.11 | -0.02 | 0.12 | -0.12 |
| Q6PAR5 | GAPVD1 | 162.40 | -0.41 | 0.05 | -0.18 | 0.32 | -0.28 | 0.07 | -0.11 | 0.25 | -0.07 |
| P16858 | GAPDH | 35.81 | -0.21 | -0.34 | -0.27 | 0.09 | -0.34 | -0.41 | -0.37 | 0.05 | 0.10 |
| Q8BHN3 | GANAB | 106.91 | -0.01 | 0.00 | 0.00 | 0.00 | 0.02 | -0.05 | -0.01 | 0.05 | 0.01 |
| Q03249 | GALT | 43.23 | -0.30 | -0.26 | -0.28 | 0.03 | -0.30 | -0.16 | -0.23 | 0.10 | -0.05 |
| Q8K157 | GALM | 37.80 | -0.09 | 0.00 | -0.04 | 0.06 | -0.08 | -0.06 | -0.07 | 0.02 | 0.03 |
| Q00612 | G6PDX | 59.26 | -0.98 | -0.75 | -0.87 | 0.16 | -0.64 | -0.90 | -0.77 | 0.18 | -0.09 |
| P97379 | G3BP2 | 54.09 | -0.02 | 0.10 | 0.04 | 0.08 | -0.15 | 0.11 | -0.02 | 0.18 | 0.06 |
| P97855 | G3BP1 | 51.83 | -0.31 | -0.04 | -0.17 | 0.19 | -0.12 | 0.01 | -0.06 | 0.09 | -0.11 |
| Q5RJY2 | G2E3 | 81.78 | 0.38 | 0.07 | 0.22 | 0.22 | -0.24 | 0.24 | 0.00 | 0.34 | 0.22 |
| Q8VDC1 | FYCO1 | 162.33 | -0.17 | -0.23 | -0.20 | 0.04 | -0.45 | -0.36 | -0.40 | 0.06 | 0.20 |
| Q61584 | FXR1 | 76.22 | 0.45 | -0.14 | 0.15 | 0.42 | 0.19 | -0.06 | 0.06 | 0.17 | 0.09 |

| P56959 | FUS | 52.67 | -0.50 | -0.19 | -0.35 | 0.22 | 0.29 | -0.11 | 0.09 | 0.28 | -0.44 |
| --- | --- | --- | --- | --- | --- | --- | --- | --- | --- | --- | --- |
| Q91WJ8 | FUBP1 | 68.54 | -0.22 | -0.33 | -0.28 | 0.08 | -0.12 | -0.27 | -0.19 | 0.11 | -0.09 |
| P29391 | FTL1 | 20.80 | 0.13 | 0.44 | 0.28 | 0.22 | 0.69 | 1.15 | 0.92 | 0.33 | -0.64 |
| P09528 | FTH1 | 21.07 | -0.39 | 0.14 | -0.13 | 0.37 | 0.21 | 0.76 | 0.48 | 0.39 | -0.61 |
| Q91XD4 | FTCD | 58.94 | -0.05 | 0.00 | -0.03 | 0.04 | -0.01 | 0.09 | 0.04 | 0.07 | -0.06 |
| Q8BX90 | FNDC3A | 131.96 | 0.30 | 0.07 | 0.18 | 0.16 | -0.58 | -0.01 | -0.29 | 0.40 | 0.47 |
| Q9ER35 | FN3K | 35.03 | 0.33 | 0.19 | 0.26 | 0.10 | 0.15 | 0.18 | 0.16 | 0.02 | 0.09 |
| P11276 | FN1 | 272.53 | -0.30 | -0.20 | -0.25 | 0.07 | 0.03 | -0.22 | -0.09 | 0.18 | -0.16 |
| P97872 | FMO5 | 60.00 | 0.76 | 0.78 | 0.77 | 0.01 | 0.76 | 0.75 | 0.75 | 0.01 | 0.02 |
| P97501 | FMO3 | 60.52 | -5.28 | -5.29 | -5.29 | 0.01 | -4.44 | -5.20 | -4.82 | 0.54 | -0.46 |
| Q8K2I3 | FMO2 | 60.97 | -1.28 | -1.11 | -1.19 | 0.12 | -0.88 | -1.08 | -0.98 | 0.14 | -0.21 |
| P50285 | FMO1 | 59.91 | -1.18 | -1.06 | -1.12 | 0.08 | -0.93 | -0.90 | -0.91 | 0.02 | -0.20 |
| Q60634 | FLOT2 | 47.04 | 0.07 | 0.12 | 0.09 | 0.03 | 0.41 | 0.27 | 0.34 | 0.10 | -0.25 |

| O08917 | FLOT1 | 47.51 | 0.47 | 0.16 | 0.31 | 0.22 | 0.38 | 0.28 | 0.33 | 0.07 | -0.02 |
| --- | --- | --- | --- | --- | --- | --- | --- | --- | --- | --- | --- |
| Q80X90 | FLNB | 277.82 | -0.12 | 0.01 | -0.06 | 0.09 | -0.08 | -0.05 | -0.07 | 0.02 | 0.01 |
| Q8BTM8 | FLNA | 281.22 | 0.02 | -0.13 | -0.06 | 0.11 | 0.03 | -0.01 | 0.01 | 0.03 | -0.07 |
| Q9JJ28 | FLII | 144.80 | -0.37 | 0.02 | -0.17 | 0.27 | -0.40 | 0.04 | -0.18 | 0.31 | 0.01 |
| Q8R123 | FLAD1 | 54.77 | -0.47 | 0.33 | -0.07 | 0.56 | 0.16 | 0.34 | 0.25 | 0.13 | -0.32 |
| O35465 | FKBP8 | 43.53 | -0.07 | 0.03 | -0.02 | 0.07 | -0.11 | -0.07 | -0.09 | 0.03 | 0.06 |
| Q64378 | FKBP5 | 50.97 | 0.07 | 0.18 | 0.13 | 0.08 | 0.22 | 0.27 | 0.24 | 0.03 | -0.12 |
| P30416 | FKBP4 | 51.57 | -0.70 | -0.71 | -0.70 | 0.01 | -0.75 | -0.72 | -0.73 | 0.02 | 0.03 |
| Q62446 | FKBP3 | 25.15 | 0.16 | 0.14 | 0.15 | 0.01 | 0.12 | 0.15 | 0.13 | 0.03 | 0.01 |
| P45878 | FKBP2 | 15.34 | 0.00 | 0.08 | 0.04 | 0.06 | -0.05 | -0.02 | -0.03 | 0.02 | 0.08 |
| P26883 | FKBP1A | 11.92 | -0.31 | -0.18 | -0.25 | 0.09 | -0.36 | -0.26 | -0.31 | 0.07 | 0.06 |
| Q6P9Q6 | FKBP15 | 132.96 | -0.62 | -0.18 | -0.40 | 0.32 | -0.45 | -0.01 | -0.23 | 0.31 | -0.17 |
| Q9CQ92 | FIS1 | 17.01 | 0.35 | 0.21 | 0.28 | 0.10 | 0.35 | 0.32 | 0.33 | 0.02 | -0.06 |

| Q9D824 | FIP1L1 | 64.96 | -0.48 | -0.42 | -0.45 | 0.04 | -0.26 | -0.18 | -0.22 | 0.06 | -0.23 |
| --- | --- | --- | --- | --- | --- | --- | --- | --- | --- | --- | --- |
| P97807 | FH | 54.36 | 0.45 | 0.35 | 0.40 | 0.08 | 0.41 | 0.45 | 0.43 | 0.03 | -0.03 |
| Q71KU9 | FGL1 | 36.44 | 1.04 | 1.03 | 1.04 | 0.01 | 1.02 | 1.09 | 1.06 | 0.04 | -0.02 |
| A2AJL3 | FGGY | 60.34 | -0.49 | -0.41 | -0.45 | 0.05 | -0.50 | -0.49 | -0.50 | 0.01 | 0.05 |
| Q8VCM7 | FGG | 49.39 | 0.24 | 0.42 | 0.33 | 0.13 | 0.13 | 0.17 | 0.15 | 0.03 | 0.18 |
| Q8K0E8 | FGB | 54.75 | 0.42 | 0.55 | 0.49 | 0.09 | 0.37 | 0.38 | 0.38 | 0.01 | 0.11 |
| E9PV24 | FGA | 87.43 | 0.40 | 0.38 | 0.39 | 0.01 | 0.29 | 0.25 | 0.27 | 0.02 | 0.12 |
| Q8K1B8 | FERMT3 | 75.63 | -0.04 | -0.05 | -0.04 | 0.01 | 0.41 | -0.10 | 0.15 | 0.36 | -0.20 |
| Q8CIB5 | FERMT2 | 77.80 | -0.23 | -0.34 | -0.28 | 0.08 | -0.15 | -0.20 | -0.17 | 0.03 | -0.11 |
| P22315 | FECH | 47.13 | 0.46 | 0.52 | 0.49 | 0.04 | 0.50 | 0.64 | 0.57 | 0.11 | -0.08 |
| Q61578 | FDXR | 54.20 | 0.36 | 0.44 | 0.40 | 0.06 | 0.44 | 0.32 | 0.38 | 0.08 | 0.01 |
| P46656 | FDX1 | 20.12 | 0.77 | 1.05 | 0.91 | 0.20 | 0.84 | 0.98 | 0.91 | 0.10 | 0.00 |
| Q920E5 | FDPS | 40.58 | -0.34 | -0.41 | -0.37 | 0.05 | -0.39 | -0.32 | -0.35 | 0.05 | -0.02 |

| Q3UQN2 | FCHO2 | 88.73 | -0.13 | -0.03 | -0.08 | 0.07 | -0.12 | 0.02 | -0.05 | 0.09 | -0.03 |
| --- | --- | --- | --- | --- | --- | --- | --- | --- | --- | --- | --- |
| Q61559 | FCGRT | 40.09 | 0.40 | 0.22 | 0.31 | 0.12 | 0.13 | 0.12 | 0.12 | 0.01 | 0.19 |
| P08101 | FCGR2 | 36.70 | 0.91 | 0.91 | 0.91 | 0.01 | 0.75 | 1.07 | 0.91 | 0.23 | 0.00 |
| Q9QXD6 | FBP1 | 36.91 | -0.49 | -0.56 | -0.53 | 0.05 | -0.53 | -0.57 | -0.55 | 0.03 | 0.03 |
| P35550 | FBL | 34.31 | 0.35 | 0.36 | 0.36 | 0.01 | 0.38 | 0.40 | 0.39 | 0.01 | -0.04 |
| P19096 | FASN | 272.43 | -2.09 | -1.97 | -2.03 | 0.09 | -2.07 | -1.98 | -2.02 | 0.07 | -0.01 |
| Q9WUA2 | FARSB | 65.70 | 0.37 | 0.29 | 0.33 | 0.06 | 0.31 | 0.26 | 0.29 | 0.04 | 0.04 |
| Q8C0C7 | FARSA | 57.60 | 0.29 | 0.25 | 0.27 | 0.02 | 0.26 | 0.24 | 0.25 | 0.01 | 0.02 |
| Q91VS8 | FARP2 | 121.28 | 0.66 | 0.73 | 0.70 | 0.05 | 0.87 | 0.87 | 0.87 | 0.00 | -0.18 |
| F8VPU2 | FARP1 | 118.87 | -0.36 | -0.14 | -0.25 | 0.15 | -0.24 | -0.01 | -0.13 | 0.16 | -0.13 |
| Q80VD1 | FAM98B | 45.35 | 0.03 | -0.19 | -0.08 | 0.15 | -0.25 | -0.12 | -0.19 | 0.10 | 0.11 |
| Q3TJZ6 | FAM98A | 55.06 | -0.01 | 0.20 | 0.10 | 0.15 | 0.03 | 0.30 | 0.16 | 0.19 | -0.06 |
| Q76LS9 | FAM63A | 51.23 | 0.50 | 0.45 | 0.47 | 0.03 | 0.45 | 0.36 | 0.41 | 0.06 | 0.07 |

| Q921M7 | FAM49B | 36.78 | -0.36 | -0.31 | -0.33 | 0.04 | -0.10 | -0.29 | -0.19 | 0.14 | -0.14 |
| --- | --- | --- | --- | --- | --- | --- | --- | --- | --- | --- | --- |
| Q9D8N2 | FAM45A | 40.42 | 0.04 | -0.03 | 0.00 | 0.05 | -0.10 | 0.01 | -0.05 | 0.07 | 0.05 |
| Q91VU0 | FAM3C | 24.75 | 0.03 | -0.05 | -0.01 | 0.06 | 0.01 | 0.09 | 0.05 | 0.06 | -0.06 |
| Q9CYH2 | FAM213A | 24.39 | -0.63 | -0.66 | -0.65 | 0.02 | -0.76 | -0.69 | -0.72 | 0.05 | 0.08 |
| Q6PGL7 | FAM21 | 145.31 | -0.34 | -0.07 | -0.21 | 0.19 | -0.26 | 0.01 | -0.12 | 0.19 | -0.08 |
| Q9D6U8 | FAM162A | 17.73 | 0.17 | 0.33 | 0.25 | 0.11 | 0.15 | 0.28 | 0.22 | 0.09 | 0.03 |
| Q3UW53 | FAM129A | 102.65 | -0.74 | -0.47 | -0.60 | 0.19 | -0.50 | -0.41 | -0.45 | 0.06 | -0.15 |
| Q6A0A9 | FAM120A | 121.64 | -0.66 | 0.03 | -0.32 | 0.48 | -0.34 | 0.02 | -0.16 | 0.26 | -0.15 |
| Q9D281 | FAM114A1 | 61.01 | -0.21 | -0.42 | -0.32 | 0.15 | -0.28 | -0.33 | -0.31 | 0.03 | -0.01 |
| Q8R0F8 | FAHD1 | 25.17 | 0.19 | 0.31 | 0.25 | 0.08 | 0.13 | 0.26 | 0.20 | 0.09 | 0.05 |
| P35505 | FAH | 46.18 | -0.02 | -0.04 | -0.03 | 0.01 | -0.14 | -0.04 | -0.09 | 0.07 | 0.06 |
| Q3TDN2 | FAF2 | 52.47 | 0.24 | 0.14 | 0.19 | 0.07 | 0.20 | 0.09 | 0.15 | 0.07 | 0.04 |
| P54731 | FAF1 | 73.86 | -0.18 | -0.27 | -0.22 | 0.06 | -0.18 | -0.18 | -0.18 | 0.00 | -0.04 |

| Q9Z0R9 | FADS2 | 52.39 | -1.05 | -0.95 | -1.00 | 0.07 | -0.94 | -0.94 | -0.94 | 0.00 | -0.06 |
| --- | --- | --- | --- | --- | --- | --- | --- | --- | --- | --- | --- |
| Q920L1 | FADS1 | 52.32 | -0.86 | -1.06 | -0.96 | 0.14 | -1.11 | -1.02 | -1.07 | 0.06 | 0.11 |
| Q05816 | FABP5 | 15.14 | -1.66 | -1.66 | -1.66 | 0.00 | -1.64 | -1.63 | -1.63 | 0.01 | -0.02 |
| P55050 | FABP2 | 15.13 | -0.58 | -0.54 | -0.56 | 0.03 | -1.05 | -1.03 | -1.04 | 0.02 | 0.48 |
| P12710 | FABP1 | 14.25 | -0.32 | -0.13 | -0.22 | 0.13 | -0.58 | -0.50 | -0.54 | 0.06 | 0.31 |
| O08914 | FAAH | 63.22 | 0.05 | 0.08 | 0.06 | 0.02 | 0.00 | 0.10 | 0.05 | 0.07 | 0.01 |
| O88783 | F5 | 247.23 | -0.24 | 0.08 | -0.08 | 0.23 | -0.29 | 0.07 | -0.11 | 0.25 | 0.03 |
| P19221 | F2 | 70.27 | 0.21 | 0.24 | 0.23 | 0.02 | 0.03 | -0.03 | 0.00 | 0.04 | 0.22 |
| O88792 | F11R | 32.42 | 0.45 | 0.17 | 0.31 | 0.20 | 0.37 | 0.28 | 0.32 | 0.07 | -0.02 |
| P26040 | EZR | 69.41 | -0.03 | 0.01 | -0.01 | 0.03 | 0.01 | -0.02 | -0.01 | 0.02 | 0.00 |
| Q9JHI7 | EXOSC9 | 48.94 | 0.25 | -0.13 | 0.06 | 0.27 | 0.19 | 0.09 | 0.14 | 0.07 | -0.08 |
| Q6PGF7 | EXOC8 | 81.03 | 0.03 | 0.21 | 0.12 | 0.13 | -0.06 | 0.09 | 0.01 | 0.11 | 0.11 |
| O35250 | EXOC7 | 79.96 | 0.10 | 0.06 | 0.08 | 0.03 | 0.28 | 0.07 | 0.18 | 0.14 | -0.10 |

| O35382 | EXOC4 | 110.54 | 0.17 | 0.06 | 0.11 | 0.07 | -0.11 | 0.04 | -0.04 | 0.10 | 0.15 |
| --- | --- | --- | --- | --- | --- | --- | --- | --- | --- | --- | --- |
| Q6KAR6 | EXOC3 | 86.45 | 0.32 | 0.09 | 0.20 | 0.17 | 0.17 | 0.12 | 0.14 | 0.03 | 0.06 |
| Q9D4H1 | EXOC2 | 103.96 | 0.08 | 0.04 | 0.06 | 0.03 | 0.14 | 0.04 | 0.09 | 0.07 | -0.03 |
| Q61545 | EWSR1 | 68.46 | -0.59 | -0.15 | -0.37 | 0.31 | -0.37 | 0.01 | -0.18 | 0.27 | -0.19 |
| Q8BWU8 | ETNPPL | 55.50 | 0.22 | 0.40 | 0.31 | 0.13 | 0.36 | 0.49 | 0.42 | 0.09 | -0.11 |
| Q921G7 | ETFDH | 68.09 | 0.11 | 0.19 | 0.15 | 0.06 | 0.12 | 0.08 | 0.10 | 0.03 | 0.05 |
| Q9DCW4 | ETFB | 27.62 | 0.02 | 0.03 | 0.02 | 0.00 | 0.02 | 0.02 | 0.02 | 0.00 | 0.00 |
| Q99LC5 | ETFA | 35.01 | -0.01 | 0.02 | 0.01 | 0.02 | 0.00 | 0.01 | 0.01 | 0.01 | 0.00 |
| Q8BWY3 | ETF1 | 49.03 | -0.10 | 0.00 | -0.05 | 0.07 | 0.00 | 0.01 | 0.00 | 0.01 | -0.05 |
| Q3TZZ7 | ESYT2 | 94.14 | 0.28 | -0.20 | 0.04 | 0.34 | 0.13 | -0.27 | -0.07 | 0.28 | 0.12 |
| Q9R0P3 | ESD | 31.32 | 0.05 | -0.06 | -0.01 | 0.08 | -0.13 | -0.14 | -0.14 | 0.01 | 0.13 |
| Q9D1Q6 | ERP44 | 46.85 | -0.01 | -0.19 | -0.10 | 0.13 | -0.10 | -0.09 | -0.09 | 0.01 | -0.01 |
| P57759 | ERP29 | 28.82 | -0.08 | -0.12 | -0.10 | 0.03 | -0.25 | -0.22 | -0.24 | 0.02 | 0.14 |

| Q8R180 | ERO1L | 54.08 | -0.23 | -0.20 | -0.22 | 0.02 | -0.18 | -0.26 | -0.22 | 0.06 | 0.00 |
| --- | --- | --- | --- | --- | --- | --- | --- | --- | --- | --- | --- |
| Q8BFZ9 | ERLIN2 | 37.87 | -0.13 | -0.12 | -0.12 | 0.01 | -0.13 | -0.10 | -0.12 | 0.02 | -0.01 |
| Q91X78 | ERLIN1 | 38.94 | 0.00 | 0.00 | 0.00 | 0.00 | 0.22 | 0.21 | 0.21 | 0.00 | -0.22 |
| Q9CQE7 | ERGIC3 | 43.21 | -0.10 | -0.32 | -0.21 | 0.15 | -0.16 | -0.22 | -0.19 | 0.04 | -0.02 |
| Q9DC16 | ERGIC1 | 32.56 | 0.30 | 0.34 | 0.32 | 0.03 | 0.42 | 0.43 | 0.42 | 0.01 | -0.10 |
| Q99MI1 | ERC1 | 128.33 | 0.20 | 0.47 | 0.33 | 0.19 | 0.28 | 0.41 | 0.35 | 0.09 | -0.01 |
| Q9EQH2 | ERAP1 | 106.60 | -0.18 | -0.28 | -0.23 | 0.07 | -0.20 | -0.19 | -0.19 | 0.01 | -0.04 |
| Q99K30 | EPS8L2 | 82.23 | 0.72 | 0.66 | 0.69 | 0.04 | 0.77 | 0.74 | 0.75 | 0.02 | -0.06 |
| Q60902 | EPS15L1 | 99.31 | -0.20 | 0.00 | -0.10 | 0.14 | -0.02 | 0.04 | 0.01 | 0.04 | -0.11 |
| P42567 | EPS15 | 98.47 | -0.11 | 0.11 | 0.00 | 0.16 | 0.18 | 0.16 | 0.17 | 0.02 | -0.17 |
| Q8CGC7 | EPRS | 170.08 | -0.02 | 0.13 | 0.05 | 0.11 | 0.03 | 0.12 | 0.08 | 0.06 | -0.02 |
| Q8R0W0 | EPPK1 | 723.30 | 0.39 | 0.39 | 0.39 | 0.00 | 0.74 | 0.67 | 0.70 | 0.05 | -0.31 |
| Q80VP1 | EPN1 | 60.21 | 0.36 | 0.35 | 0.36 | 0.01 | 0.25 | 0.41 | 0.33 | 0.11 | 0.03 |

| P34914 | EPHX2 | 62.52 | 0.89 | 0.92 | 0.90 | 0.02 | 0.65 | 0.71 | 0.68 | 0.04 | 0.23 |
| --- | --- | --- | --- | --- | --- | --- | --- | --- | --- | --- | --- |
| Q9D379 | EPHX1 | 52.58 | 1.61 | 1.46 | 1.53 | 0.11 | 1.40 | 1.40 | 1.40 | 0.00 | 0.13 |
| P49222 | EPB42 | 76.76 | 0.45 | 0.29 | 0.37 | 0.11 | 0.32 | 0.16 | 0.24 | 0.11 | 0.13 |
| Q8BGS1 | EPB41L5 | 81.64 | 0.00 | 0.28 | 0.14 | 0.20 | 0.23 | 0.53 | 0.38 | 0.21 | -0.24 |
| O70318 | EPB41L2 | 109.94 | -0.88 | -0.35 | -0.62 | 0.37 | -0.46 | -0.18 | -0.32 | 0.20 | -0.29 |
| P48193 | EPB41 | 95.91 | 0.07 | 0.28 | 0.18 | 0.15 | 0.12 | 0.24 | 0.18 | 0.09 | 0.00 |
| Q9WUZ9 | ENTPD5 | 47.10 | 0.94 | 0.90 | 0.92 | 0.03 | 0.76 | 0.78 | 0.77 | 0.01 | 0.15 |
| P06802 | ENPP1 | 103.17 | 0.14 | 0.29 | 0.22 | 0.11 | 0.36 | 0.40 | 0.38 | 0.03 | -0.16 |
| P16406 | ENPEP | 107.96 | -0.54 | -0.11 | -0.33 | 0.31 | -0.36 | -0.20 | -0.28 | 0.11 | -0.05 |
| P21550 | ENO3 | 47.02 | -3.61 | -3.64 | -3.63 | 0.02 | -3.61 | -3.65 | -3.63 | 0.03 | 0.01 |
| P17182 | ENO1 | 47.14 | -0.40 | -0.44 | -0.42 | 0.03 | -0.53 | -0.58 | -0.55 | 0.03 | 0.13 |
| O08600 | ENDOG | 32.19 | -0.94 | -0.78 | -0.86 | 0.11 | -0.62 | -0.57 | -0.60 | 0.03 | -0.26 |
| Q3UMY5 | EML4 | 110.03 | -0.20 | -0.17 | -0.18 | 0.02 | -0.04 | 0.11 | 0.04 | 0.11 | -0.22 |

| O70378 | EMC8 | 23.35 | -0.08 | 0.21 | 0.06 | 0.20 | -0.09 | 0.11 | 0.01 | 0.14 | 0.05 |
| --- | --- | --- | --- | --- | --- | --- | --- | --- | --- | --- | --- |
| Q99KI3 | EMC3 | 29.98 | 0.15 | 0.18 | 0.17 | 0.02 | -0.04 | 0.04 | 0.00 | 0.06 | 0.17 |
| Q9CRD2 | EMC2 | 34.93 | 0.18 | 0.22 | 0.20 | 0.03 | 0.23 | 0.12 | 0.17 | 0.07 | 0.03 |
| Q8C7X2 | EMC1 | 111.60 | 0.20 | 0.11 | 0.15 | 0.06 | 0.27 | 0.12 | 0.19 | 0.11 | -0.04 |
| Q9JLJ4 | ELOVL2 | 34.21 | -1.15 | -1.23 | -1.19 | 0.05 | -1.32 | -1.30 | -1.31 | 0.02 | 0.12 |
| P70372 | ELAVL1 | 36.17 | -0.09 | -0.11 | -0.10 | 0.02 | -0.06 | -0.11 | -0.08 | 0.04 | -0.02 |
| Q80Y81 | ELAC2 | 92.72 | -0.30 | -0.29 | -0.30 | 0.01 | -0.12 | -0.24 | -0.18 | 0.09 | -0.12 |
| Q05D44 | EIF5B | 137.61 | -0.50 | -0.06 | -0.28 | 0.31 | -0.48 | -0.03 | -0.26 | 0.32 | -0.03 |
| P59325 | EIF5 | 48.97 | 0.30 | 0.25 | 0.27 | 0.04 | 0.23 | 0.20 | 0.21 | 0.02 | 0.06 |
| Q62448 | EIF4G2 | 102.10 | -0.46 | -0.01 | -0.23 | 0.32 | -0.43 | 0.05 | -0.19 | 0.34 | -0.05 |
| Q6NZJ6 | EIF4G1 | 176.07 | -0.28 | 0.02 | -0.13 | 0.21 | -0.18 | -0.09 | -0.14 | 0.07 | 0.01 |
| P63073 | EIF4E | 25.05 | 0.13 | 0.19 | 0.16 | 0.04 | 0.16 | 0.13 | 0.15 | 0.02 | 0.02 |
| Q8BGD9 | EIF4B | 68.84 | -0.04 | 0.00 | -0.02 | 0.03 | -0.13 | 0.20 | 0.04 | 0.23 | -0.06 |

| Q91VC3 | EIF4A3 | 46.84 | -0.14 | -0.11 | -0.13 | 0.02 | 0.09 | -0.06 | 0.01 | 0.11 | -0.14 |
| --- | --- | --- | --- | --- | --- | --- | --- | --- | --- | --- | --- |
| P10630 | EIF4A2 | 46.40 | 0.33 | 0.37 | 0.35 | 0.03 | 0.36 | 0.35 | 0.35 | 0.01 | -0.01 |
| P60843 | EIF4A1 | 46.15 | 0.18 | 0.17 | 0.18 | 0.01 | 0.13 | 0.12 | 0.12 | 0.01 | 0.05 |
| Q99JX4 | EIF3M | 42.52 | -0.06 | 0.02 | -0.02 | 0.05 | 0.03 | 0.07 | 0.05 | 0.02 | -0.07 |
| Q8QZY1 | EIF3L | 66.61 | 0.05 | 0.04 | 0.05 | 0.01 | 0.11 | 0.03 | 0.07 | 0.05 | -0.02 |
| Q9DBZ5 | EIF3K | 25.09 | 0.03 | 0.19 | 0.11 | 0.11 | -0.01 | 0.12 | 0.06 | 0.09 | 0.06 |
| Q3UGC7 |  | 29.34 | 0.08 | 0.11 | 0.09 | 0.02 | 0.00 | 0.02 | 0.01 | 0.01 | 0.08 |
| Q9QZD9 | EIF3I | 36.46 | 0.05 | 0.03 | 0.04 | 0.01 | -0.03 | 0.04 | 0.00 | 0.05 | 0.04 |
| Q91WK2 | EIF3H | 39.83 | 0.00 | 0.00 | 0.00 | 0.00 | 0.04 | 0.02 | 0.03 | 0.01 | -0.04 |
| Q9Z1D1 | EIF3G | 35.64 | -0.04 | 0.13 | 0.04 | 0.12 | 0.08 | 0.09 | 0.09 | 0.00 | -0.04 |
| P60229 | EIF3E | 52.22 | 0.10 | 0.08 | 0.09 | 0.02 | 0.08 | 0.03 | 0.05 | 0.04 | 0.04 |
| O70194 | EIF3D | 63.99 | 0.05 | -0.03 | 0.01 | 0.05 | 0.08 | 0.06 | 0.07 | 0.01 | -0.06 |
| Q8R1B4 | EIF3C | 105.53 | -0.03 | 0.04 | 0.01 | 0.04 | 0.00 | 0.02 | 0.01 | 0.01 | 0.00 |

| Q8JZQ9 | EIF3B | 91.37 | 0.13 | 0.13 | 0.13 | 0.00 | 0.16 | 0.11 | 0.14 | 0.03 | -0.01 |
| --- | --- | --- | --- | --- | --- | --- | --- | --- | --- | --- | --- |
| P23116 | EIF3A | 161.93 | -0.21 | 0.08 | -0.06 | 0.20 | -0.10 | 0.13 | 0.02 | 0.16 | -0.08 |
| Q99L45 | EIF2S2 | 38.09 | 0.05 | 0.04 | 0.05 | 0.01 | -0.01 | 0.07 | 0.03 | 0.06 | 0.02 |
| Q6ZWX6 | EIF2S1 | 36.11 | 0.01 | 0.05 | 0.03 | 0.03 | -0.07 | -0.04 | -0.06 | 0.02 | 0.08 |
| Q61749 | EIF2B4 | 57.62 | -0.36 | -0.24 | -0.30 | 0.08 | -0.43 | -0.12 | -0.28 | 0.21 | -0.02 |
| Q99LD9 | EIF2B2 | 38.90 | 0.21 | 0.00 | 0.10 | 0.15 | -0.07 | -0.02 | -0.04 | 0.04 | 0.15 |
| Q99LC8 | EIF2B1 | 33.82 | -0.12 | -0.07 | -0.10 | 0.03 | -0.14 | -0.04 | -0.09 | 0.07 | 0.00 |
| Q8BJW6 | EIF2A | 64.40 | -0.30 | 0.02 | -0.14 | 0.22 | -0.07 | 0.07 | 0.00 | 0.10 | -0.14 |
| Q9CXU9 | EIF1B;EIF1 | 12.82 | 0.42 | 0.46 | 0.44 | 0.03 | 0.47 | 0.29 | 0.38 | 0.12 | 0.06 |
| Q8BMJ3 |  | 16.46 | 0.09 | 0.12 | 0.11 | 0.02 | 0.09 | 0.04 | 0.07 | 0.03 | 0.04 |
| Q9DBM2 | EHHADH | 78.30 | 0.53 | 0.45 | 0.49 | 0.06 | 0.10 | -0.02 | 0.04 | 0.08 | 0.45 |
| Q9QXY6 | EHD3 | 60.82 | -0.11 | 0.05 | -0.03 | 0.11 | -0.01 | 0.19 | 0.09 | 0.14 | -0.12 |
| Q9WVK4 | EHD1 | 60.60 | 0.01 | -0.05 | -0.02 | 0.04 | -0.03 | 0.00 | -0.02 | 0.02 | 0.00 |

| Q01279 | EGFR | 134.85 | 1.07 | 1.33 | 1.20 | 0.18 | 0.99 | 1.51 | 1.25 | 0.37 | -0.05 |
| --- | --- | --- | --- | --- | --- | --- | --- | --- | --- | --- | --- |
| O08810 | EFTUD2 | 109.36 | -0.10 | -0.15 | -0.12 | 0.04 | 0.07 | -0.06 | 0.00 | 0.09 | -0.13 |
| Q8C0D5 | EFTUD1 | 125.78 | -0.21 | 0.03 | -0.09 | 0.17 | -0.12 | -0.05 | -0.09 | 0.05 | 0.00 |
| Q8BG67 | EFR3A | 92.61 | 0.55 | 0.42 | 0.48 | 0.10 | 0.25 | 0.29 | 0.27 | 0.03 | 0.21 |
| Q9D8Y0 | EFHD2 | 26.79 | 0.24 | 0.40 | 0.32 | 0.11 | 0.47 | 0.50 | 0.48 | 0.02 | -0.17 |
| Q9JHW4 | EEFSEC | 63.54 | 0.28 | 0.42 | 0.35 | 0.10 | 0.41 | 0.48 | 0.45 | 0.05 | -0.10 |
| P58252 | EEF2 | 95.31 | -0.22 | -0.17 | -0.19 | 0.04 | -0.22 | -0.16 | -0.19 | 0.04 | 0.00 |
| Q9D8N0 | EEF1G | 50.06 | 0.25 | 0.17 | 0.21 | 0.06 | 0.24 | 0.17 | 0.20 | 0.05 | 0.01 |
| P57776 | EEF1D | 31.29 | 0.23 | 0.21 | 0.22 | 0.01 | 0.18 | 0.21 | 0.19 | 0.02 | 0.03 |
| O70251 | EEF1B | 24.69 | 0.17 | 0.17 | 0.17 | 0.00 | 0.19 | 0.16 | 0.18 | 0.02 | -0.01 |
| P10126 | EEF1A1 | 50.11 | -0.07 | -0.15 | -0.11 | 0.06 | -0.12 | -0.21 | -0.16 | 0.06 | 0.05 |
| Q8BL66 | EEA1 | 160.91 | -0.21 | 0.08 | -0.07 | 0.21 | -0.01 | 0.13 | 0.06 | 0.10 | -0.13 |
| Q9JMG1 | EDF1 | 16.37 | -0.38 | -0.25 | -0.32 | 0.09 | -0.35 | -0.22 | -0.29 | 0.09 | -0.03 |

| Q6PDI5 | ECM29 | 203.70 | -0.06 | -0.17 | -0.12 | 0.08 | -0.14 | -0.10 | -0.12 | 0.03 | 0.00 |
| --- | --- | --- | --- | --- | --- | --- | --- | --- | --- | --- | --- |
| Q61508 | ECM1 | 62.83 | 0.11 | -0.05 | 0.03 | 0.12 | 0.13 | 0.02 | 0.08 | 0.08 | -0.05 |
| Q9WUR2 | ECI2 | 43.27 | 0.01 | 0.01 | 0.01 | 0.00 | -0.13 | -0.05 | -0.09 | 0.06 | 0.10 |
| P42125 | ECI1 | 32.25 | 0.07 | 0.12 | 0.09 | 0.04 | -0.04 | -0.04 | -0.04 | 0.00 | 0.13 |
| Q8BH95 | ECHS1 | 31.47 | -0.04 | 0.00 | -0.02 | 0.03 | 0.00 | 0.05 | 0.03 | 0.03 | -0.05 |
| Q9D7J9 | ECHDC3 | 32.40 | -0.04 | -0.10 | -0.07 | 0.04 | 0.01 | 0.08 | 0.04 | 0.06 | -0.12 |
| Q9D9V3 | ECHDC1 | 35.47 | -0.21 | -0.08 | -0.15 | 0.09 | -0.25 | -0.08 | -0.17 | 0.12 | 0.02 |
| O35459 | ECH1 | 36.12 | -0.13 | -0.03 | -0.08 | 0.07 | -0.35 | -0.28 | -0.32 | 0.05 | 0.23 |
| Q4PZA2 | ECE1 | 87.07 | -0.34 | -0.44 | -0.39 | 0.07 | -0.67 | -0.52 | -0.59 | 0.11 | 0.20 |
| Q6PDL0 | DYNC1LI2 | 54.22 | -0.57 | -0.10 | -0.34 | 0.34 | -0.58 | -0.27 | -0.42 | 0.22 | 0.08 |
| Q8R1Q8 | DYNC1LI1 | 56.61 | -0.13 | -0.07 | -0.10 | 0.04 | -0.14 | -0.13 | -0.14 | 0.01 | 0.04 |
| O88487 | DYNC1I2 | 68.39 | -0.05 | -0.08 | -0.06 | 0.02 | -0.14 | -0.08 | -0.11 | 0.05 | 0.05 |
| Q9JHU4 | DYNC1H1 | 532.04 | -0.01 | -0.09 | -0.05 | 0.06 | 0.02 | -0.04 | -0.01 | 0.04 | -0.04 |

| Q3UIR3 | DTX3L | 83.04 | -0.21 | -0.28 | -0.24 | 0.05 | -0.23 | -0.31 | -0.27 | 0.06 | 0.02 |
| --- | --- | --- | --- | --- | --- | --- | --- | --- | --- | --- | --- |
| Q9R0P5 | DSTN | 18.52 | 0.17 | 0.09 | 0.13 | 0.06 | 0.11 | 0.13 | 0.12 | 0.02 | 0.01 |
| E9Q557 | DSP | 332.91 | -0.13 | -0.16 | -0.14 | 0.02 | 0.02 | -0.03 | -0.01 | 0.03 | -0.14 |
| O55111 | DSG2 | 122.38 | -1.13 | -0.49 | -0.81 | 0.46 | -1.15 | -0.38 | -0.77 | 0.55 | -0.04 |
| O35075 | DSCR3 | 32.97 | -0.01 | -0.05 | -0.03 | 0.03 | 0.01 | 0.05 | 0.03 | 0.02 | -0.06 |
| Q9QXB9 | DRG2 | 40.72 | -0.03 | -0.09 | -0.06 | 0.05 | -0.11 | -0.18 | -0.15 | 0.04 | 0.09 |
| P32233 | DRG1 | 40.51 | 0.01 | -0.08 | -0.03 | 0.06 | 0.02 | 0.00 | 0.01 | 0.01 | -0.04 |
| O08553 | DPYSL2 | 62.28 | -0.26 | 0.02 | -0.12 | 0.20 | 0.07 | 0.00 | 0.03 | 0.04 | -0.16 |
| Q9EQF5 | DPYS | 56.72 | -0.53 | -0.46 | -0.49 | 0.05 | -0.20 | -0.11 | -0.16 | 0.06 | -0.33 |
| Q8CHR6 | DPYD | 111.25 | 0.32 | 0.20 | 0.26 | 0.09 | 0.32 | 0.25 | 0.29 | 0.05 | -0.03 |
| Q9ET22 | DPP7 | 56.25 | 1.28 | 1.32 | 1.30 | 0.02 | 1.27 | 1.27 | 1.27 | 0.00 | 0.03 |
| P28843 | DPP4 | 87.44 | 0.40 | 0.39 | 0.40 | 0.01 | 0.35 | 0.37 | 0.36 | 0.01 | 0.04 |
| Q99KK7 | DPP3 | 82.90 | 0.06 | -0.06 | 0.00 | 0.08 | 0.05 | -0.04 | 0.01 | 0.07 | 0.00 |

| O70152 | DPM1 | 29.17 | -0.39 | -0.08 | -0.23 | 0.22 | -0.48 | -0.11 | -0.30 | 0.27 | 0.06 |
| --- | --- | --- | --- | --- | --- | --- | --- | --- | --- | --- | --- |
| Q9Z2W0 | DNPEP | 52.21 | -0.25 | -0.20 | -0.22 | 0.04 | -0.28 | -0.41 | -0.35 | 0.09 | 0.12 |
| P39054 | DNM2 | 98.14 | -0.07 | 0.04 | -0.02 | 0.08 | 0.10 | 0.08 | 0.09 | 0.01 | -0.11 |
| Q8K1M6 | DNM1L | 82.66 | -0.07 | -0.09 | -0.08 | 0.01 | -0.01 | -0.11 | -0.06 | 0.07 | -0.01 |
| Q6NZB0 | DNAJC8 | 29.81 | -0.62 | -0.50 | -0.56 | 0.09 | -0.10 | -0.40 | -0.25 | 0.21 | -0.31 |
| P60904 | DNAJC5 | 22.10 | 0.69 | 0.13 | 0.41 | 0.40 | 0.54 | 0.25 | 0.40 | 0.21 | 0.02 |
| Q91YW3 | DNAJC3 | 57.46 | -0.36 | -0.27 | -0.31 | 0.07 | -0.39 | -0.25 | -0.32 | 0.10 | 0.01 |
| A2ALW5 | DNAJC25 | 41.94 | -0.20 | -0.05 | -0.13 | 0.11 | -0.24 | -0.15 | -0.19 | 0.07 | 0.06 |
| P54103 | DNAJC2 | 71.72 | 0.10 | 0.00 | 0.05 | 0.07 | -0.11 | 0.08 | -0.01 | 0.13 | 0.06 |
| Q5U458 | DNAJC11 | 63.23 | 0.28 | 0.06 | 0.17 | 0.15 | 0.09 | 0.10 | 0.10 | 0.01 | 0.08 |
| Q61712 | DNAJC1 | 63.87 | -0.20 | -0.22 | -0.21 | 0.01 | 0.00 | -0.12 | -0.06 | 0.08 | -0.15 |
| Q9D832 | DNAJB4 | 37.78 | 0.18 | 0.07 | 0.13 | 0.07 | 0.08 | 0.06 | 0.07 | 0.01 | 0.06 |
| Q9QYI4 | DNAJB12 | 41.99 | 0.37 | 0.21 | 0.29 | 0.11 | 0.33 | 0.14 | 0.23 | 0.13 | 0.05 |

| Q99KV1 | DNAJB11 | 40.56 | 0.11 | 0.08 | 0.09 | 0.02 | 0.05 | 0.08 | 0.07 | 0.02 | 0.03 |
| --- | --- | --- | --- | --- | --- | --- | --- | --- | --- | --- | --- |
| Q9QYJ3 | DNAJB1 | 38.17 | -0.20 | 0.11 | -0.04 | 0.21 | 0.01 | 0.07 | 0.04 | 0.04 | -0.09 |
| Q99M87 | DNAJA3 | 52.44 | 0.24 | 0.17 | 0.21 | 0.05 | 0.35 | 0.24 | 0.30 | 0.08 | -0.09 |
| Q9QYJ0 | DNAJA2 | 45.75 | 0.07 | -0.01 | 0.03 | 0.05 | -0.03 | -0.14 | -0.08 | 0.08 | 0.12 |
| P63037 | DNAJA1 | 44.87 | 0.18 | -0.03 | 0.08 | 0.15 | -0.19 | -0.17 | -0.18 | 0.01 | 0.26 |
| Q9DBT9 | DMGDH | 97.25 | 0.72 | 0.60 | 0.66 | 0.09 | 0.85 | 0.68 | 0.77 | 0.12 | -0.11 |
| Q9D2G2 | DLST | 48.99 | 0.02 | 0.17 | 0.09 | 0.11 | 0.07 | 0.15 | 0.11 | 0.05 | -0.02 |
| Q811D0 | DLG1 | 100.12 | 0.04 | 0.09 | 0.06 | 0.03 | 0.13 | 0.27 | 0.20 | 0.10 | -0.14 |
| O08749 | DLD | 54.27 | 0.02 | 0.03 | 0.03 | 0.01 | 0.01 | 0.07 | 0.04 | 0.05 | -0.01 |
| Q8BMF4 | DLAT | 67.94 | -0.33 | -0.25 | -0.29 | 0.06 | -0.34 | -0.27 | -0.30 | 0.05 | 0.02 |
| Q9ESX5 | DKC1 | 57.40 | 0.31 | 0.31 | 0.31 | 0.00 | 0.40 | 0.32 | 0.36 | 0.06 | -0.05 |
| O70566 | DIAPH2 | 124.87 | 0.14 | -0.25 | -0.06 | 0.27 | 0.04 | -0.15 | -0.06 | 0.14 | 0.00 |
| O08808 | DIAPH1 | 139.34 | -0.80 | -0.30 | -0.55 | 0.35 | -0.46 | -0.10 | -0.28 | 0.25 | -0.27 |

| O70133 | DHX9 | 149.47 | -0.32 | 0.06 | -0.13 | 0.27 | -0.34 | 0.11 | -0.12 | 0.32 | -0.02 |
| --- | --- | --- | --- | --- | --- | --- | --- | --- | --- | --- | --- |
| Q99PU8 | DHX30 | 136.67 | 0.36 | -0.01 | 0.18 | 0.26 | 0.04 | 0.08 | 0.06 | 0.03 | 0.12 |
| Q6PGC1 | DHX29 | 153.97 | -0.21 | 0.10 | -0.05 | 0.22 | -0.20 | 0.13 | -0.04 | 0.24 | -0.02 |
| O35286 | DHX15 | 91.01 | -0.28 | -0.10 | -0.19 | 0.12 | -0.07 | 0.08 | 0.00 | 0.10 | -0.19 |
| A2ATU0 | DHTKD1 | 102.79 | 0.76 | 0.69 | 0.72 | 0.05 | 1.56 | 1.58 | 1.57 | 0.01 | -0.85 |
| Q99J47 | DHRS7B | 34.99 | 0.50 | 0.28 | 0.39 | 0.15 | 0.34 | 0.25 | 0.29 | 0.06 | 0.10 |
| Q9CXR1 | DHRS7 | 38.17 | -0.58 | -0.66 | -0.62 | 0.06 | -0.64 | -0.59 | -0.62 | 0.03 | 0.00 |
| Q99LB2 | DHRS4 | 29.88 | 0.44 | 0.46 | 0.45 | 0.02 | 0.28 | 0.27 | 0.28 | 0.01 | 0.18 |
| O88876 | DHRS3 | 33.65 | -0.35 | -0.22 | -0.28 | 0.09 | -0.48 | -0.43 | -0.46 | 0.03 | 0.17 |
| Q3U0B3 | DHRS11 | 28.27 | 0.33 | 0.48 | 0.40 | 0.10 | 0.42 | 0.66 | 0.54 | 0.17 | -0.14 |
| Q99L04 | DHRS1 | 34.01 | 0.46 | 0.43 | 0.45 | 0.02 | 0.18 | 0.22 | 0.20 | 0.02 | 0.24 |
| P00375 | DHFR | 21.61 | -0.59 | -0.41 | -0.50 | 0.12 | -0.53 | -0.42 | -0.48 | 0.08 | -0.03 |
| Q9DBB8 | DHDH | 36.30 | 0.34 | 0.23 | 0.28 | 0.07 | 0.12 | 0.21 | 0.16 | 0.06 | 0.12 |

| Q8VCH6 | DHCR24 | 60.11 | -0.42 | -0.26 | -0.34 | 0.11 | 0.05 | -0.23 | -0.09 | 0.20 | -0.25 |
| --- | --- | --- | --- | --- | --- | --- | --- | --- | --- | --- | --- |
| P31001 | DES | 53.50 | -0.41 | -0.25 | -0.33 | 0.11 | -0.45 | -0.49 | -0.47 | 0.03 | 0.14 |
| Q91YP3 | DERA | 34.98 | -0.20 | -0.18 | -0.19 | 0.02 | -0.12 | -0.07 | -0.10 | 0.04 | -0.09 |
| Q570Y9 | DEPTOR | 46.12 | -0.35 | 0.47 | 0.06 | 0.58 | 0.04 | 0.38 | 0.21 | 0.24 | -0.15 |
| Q9CQJ6 | DENR | 22.17 | 0.23 | 0.22 | 0.23 | 0.01 | 0.13 | 0.21 | 0.17 | 0.06 | 0.06 |
| Q7TNV0 | DEK | 43.16 | -0.27 | -0.41 | -0.34 | 0.10 | -0.31 | -0.45 | -0.38 | 0.10 | 0.04 |
| Q9WV68 | DECR2 | 31.30 | -0.26 | -0.66 | -0.46 | 0.28 | -0.50 | -0.40 | -0.45 | 0.07 | -0.02 |
| Q9CQ62 | DECR1 | 36.21 | 0.03 | 0.09 | 0.06 | 0.05 | -0.19 | -0.14 | -0.17 | 0.04 | 0.23 |
| P54823 | DDX6 | 54.19 | -0.14 | 0.20 | 0.03 | 0.24 | -0.09 | 0.22 | 0.06 | 0.22 | -0.03 |
| Q6Q899 | DDX58 | 105.97 | -0.65 | -0.88 | -0.76 | 0.17 | -0.70 | -0.79 | -0.75 | 0.06 | -0.02 |
| Q61656 | DDX5 | 69.29 | -0.22 | -0.05 | -0.13 | 0.13 | -0.02 | 0.03 | 0.01 | 0.03 | -0.14 |
| Q810A7 | DDX42 | 101.96 | 0.18 | -0.13 | 0.02 | 0.22 | 0.29 | -0.05 | 0.12 | 0.24 | -0.10 |
|  |  | 73.10 | -0.36 | 0.08 | -0.14 | 0.31 | -0.15 | -0.07 | -0.11 | 0.06 | -0.03 |

| Q9Z1N5 | DDX39B | 49.04 | -0.01 | -0.06 | -0.04 | 0.03 | -0.01 | 0.07 | 0.03 | 0.05 | -0.07 |
| --- | --- | --- | --- | --- | --- | --- | --- | --- | --- | --- | --- |
| Q8VDW0 | DDX39A | 49.07 | -0.27 | -0.22 | -0.24 | 0.03 | -0.25 | -0.07 | -0.16 | 0.13 | -0.08 |
| Q9JIK5 | DDX21 | 93.55 | -0.08 | -0.07 | -0.07 | 0.01 | 0.08 | 0.03 | 0.06 | 0.03 | -0.13 |
| Q61655 | DDX19A | 53.93 | -0.22 | -0.17 | -0.20 | 0.03 | -0.20 | -0.11 | -0.16 | 0.06 | -0.04 |
| Q8K363 | DDX18 | 74.18 | -0.40 | -0.40 | -0.40 | 0.00 | -0.16 | -0.16 | -0.16 | 0.00 | -0.24 |
| Q91VR5 | DDX1 | 82.50 | 0.19 | 0.12 | 0.16 | 0.05 | 0.26 | 0.08 | 0.17 | 0.13 | -0.02 |
| Q80WW9 | DDRGK1 | 35.98 | -0.04 | -0.03 | -0.03 | 0.01 | 0.10 | -0.10 | 0.00 | 0.14 | -0.04 |
| O54734 | DDOST | 49.03 | 0.05 | 0.03 | 0.04 | 0.02 | 0.02 | 0.08 | 0.05 | 0.04 | -0.01 |
| A2ADY9 | DDI2 | 44.59 | -0.09 | -0.15 | -0.12 | 0.04 | -0.16 | -0.15 | -0.16 | 0.01 | 0.04 |
| Q80Y98 | DDHD2 | 79.58 | 0.85 | 1.01 | 0.93 | 0.11 | 0.24 | 0.50 | 0.37 | 0.19 | 0.56 |
| O88533 | DDC | 53.87 | 0.31 | 0.38 | 0.34 | 0.05 | 0.24 | 0.35 | 0.30 | 0.08 | 0.05 |
| Q3U1J4 | DDB1 | 126.85 | 0.17 | -0.03 | 0.07 | 0.15 | 0.10 | -0.04 | 0.03 | 0.10 | 0.04 |
| Q9CWS0 | DDAH1 | 31.38 | 0.38 | 0.29 | 0.34 | 0.06 | 0.03 | 0.05 | 0.04 | 0.01 | 0.29 |

| Q91X52 | DCXR | 25.75 | 0.17 | 0.19 | 0.18 | 0.02 | 0.23 | 0.25 | 0.24 | 0.02 | -0.06 |
| --- | --- | --- | --- | --- | --- | --- | --- | --- | --- | --- | --- |
| Q9QZ73 | DCUN1D1 | 30.10 | -0.42 | -0.08 | -0.25 | 0.24 | -0.08 | -0.08 | -0.08 | 0.00 | -0.17 |
| Q99KJ8 | DCTN2 | 44.12 | -0.21 | -0.20 | -0.20 | 0.01 | -0.17 | -0.19 | -0.18 | 0.02 | -0.02 |
| O08788 | DCTN1 | 141.67 | -0.49 | -0.23 | -0.36 | 0.18 | -0.56 | -0.15 | -0.36 | 0.29 | -0.01 |
| Q9DAR7 | DCPS | 38.99 | -0.31 | -0.33 | -0.32 | 0.01 | -0.23 | -0.26 | -0.24 | 0.02 | -0.08 |
| P28654 | DCN | 39.81 | 0.00 | 0.24 | 0.12 | 0.17 | 0.25 | 0.53 | 0.39 | 0.20 | -0.28 |
| P53395 | DBT | 53.25 | 0.18 | 0.14 | 0.16 | 0.03 | 0.17 | 0.28 | 0.22 | 0.08 | -0.06 |
| Q62418 | DBNL | 48.70 | -0.50 | -0.24 | -0.37 | 0.18 | -0.19 | -0.07 | -0.13 | 0.08 | -0.24 |
| P31786 | DBI | 10.00 | -0.21 | -0.07 | -0.14 | 0.10 | -0.33 | -0.26 | -0.30 | 0.05 | 0.16 |
| Q922B2 | DARS | 57.15 | -0.04 | 0.04 | 0.00 | 0.06 | -0.14 | -0.12 | -0.13 | 0.01 | 0.13 |
| Q9ER88 | DAP3 | 44.70 | 0.30 | 0.23 | 0.27 | 0.05 | 0.12 | 0.13 | 0.12 | 0.00 | 0.14 |
| Q8VC30 | DAK | 59.69 | -0.66 | -0.65 | -0.66 | 0.01 | -0.89 | -0.92 | -0.91 | 0.02 | 0.25 |
| Q62165 | DAG1 | 96.90 | 0.16 | 0.05 | 0.10 | 0.08 | 0.22 | 0.18 | 0.20 | 0.03 | -0.09 |

| Q9D172 | D10JHU81E | 28.09 | 0.05 | 0.11 | 0.08 | 0.04 | 0.18 | 0.16 | 0.17 | 0.01 | -0.09 |
| --- | --- | --- | --- | --- | --- | --- | --- | --- | --- | --- | --- |
| O88962 | CYP8B1 | 57.71 | 1.37 | 1.36 | 1.37 | 0.01 | 1.10 | 1.08 | 1.09 | 0.01 | 0.28 |
| Q60991 | CYP7B1 | 58.47 | 5.04 | 5.66 | 5.35 | 0.43 | 5.50 | 5.63 | 5.57 | 0.09 | -0.22 |
| Q8K0C4 | CYP51A1 | 56.78 | -0.28 | -0.14 | -0.21 | 0.10 | -0.15 | -0.12 | -0.13 | 0.02 | -0.08 |
| Q9DBW0 | CYP4V2 | 60.94 | 1.45 | 1.52 | 1.48 | 0.05 | 1.36 | 1.44 | 1.40 | 0.06 | 0.08 |
| Q9EP75 | CYP4F14 | 59.80 | -0.24 | -0.23 | -0.23 | 0.00 | -0.23 | -0.09 | -0.16 | 0.10 | -0.07 |
| O35728 | CYP4A14 | 58.72 | 1.61 | 1.68 | 1.64 | 0.05 | 1.00 | 1.16 | 1.08 | 0.12 | 0.56 |
| O88833 | CYP4A10 | 58.33 | 1.78 | 1.92 | 1.85 | 0.09 | 1.36 | 1.47 | 1.42 | 0.07 | 0.43 |
| O09158 | CYP3A25 | 58.12 | 0.82 | 0.97 | 0.90 | 0.11 | 0.84 | 0.92 | 0.88 | 0.06 | 0.02 |
| Q64464 | CYP3A13 | 57.49 | 0.44 | 0.41 | 0.43 | 0.02 | 0.39 | 0.47 | 0.43 | 0.05 | 0.00 |
| Q64459 | CYP3A11 | 57.85 | 0.06 | 0.14 | 0.10 | 0.06 | 0.12 | 0.24 | 0.18 | 0.08 | -0.08 |
| Q9JKJ9 | CYP39A1 | 53.57 | -2.12 | -2.32 | -2.22 | 0.14 | -2.09 | -2.11 | -2.10 | 0.02 | -0.12 |
| O54749 | CYP2J5 | 57.78 | 0.58 | 0.60 | 0.59 | 0.02 | 0.54 | 0.60 | 0.57 | 0.04 | 0.02 |

| P33267 | CYP2F2 | 55.95 | 1.26 | 1.14 | 1.20 | 0.09 | 1.12 | 1.09 | 1.10 | 0.02 | 0.10 |
| --- | --- | --- | --- | --- | --- | --- | --- | --- | --- | --- | --- |
| Q05421 | CYP2E1 | 56.80 | 0.69 | 0.69 | 0.69 | 0.00 | 0.37 | 0.47 | 0.42 | 0.07 | 0.27 |
| P11714 | CYP2D9 | 56.95 | 1.85 | 2.56 | 2.21 | 0.50 | 2.11 | 2.70 | 2.41 | 0.42 | -0.20 |
| Q8CIM7 | CYP2D26 | 56.98 | -0.26 | -0.22 | -0.24 | 0.03 | -0.28 | -0.28 | -0.28 | 0.00 | 0.04 |
|  |  |  | 0.31 | 0.09 | 0.20 | 0.16 | 0.22 | 0.08 | 0.15 | 0.10 | 0.05 |
| Q91W64 | CYP2C70 | 56.02 | -0.41 | -0.40 | -0.41 | 0.01 | -0.32 | -0.14 | -0.23 | 0.13 | -0.18 |
| Q6XVG2 | CYP2C54 | 55.86 | 0.35 | 0.32 | 0.34 | 0.02 | 0.44 | 0.48 | 0.46 | 0.03 | -0.12 |
| Q91X77 | CYP2C50 | 55.76 | 0.24 | 0.21 | 0.22 | 0.02 | 0.20 | 0.01 | 0.10 | 0.13 | 0.12 |
| P56657 | CYP2C40 | 55.76 | -1.85 | -1.66 | -1.76 | 0.13 | -1.57 | -1.76 | -1.67 | 0.13 | -0.09 |
| P56654 | CYP2C37 | 55.61 | -0.48 | -0.44 | -0.46 | 0.03 | -0.66 | -0.54 | -0.60 | 0.08 | 0.14 |
| Q64458 | CYP2C29 | 55.72 | 0.16 | 0.16 | 0.16 | 0.00 | 0.40 | 0.45 | 0.43 | 0.03 | -0.27 |
| P12790 | CYP2B9 | 55.74 | -4.61 | -4.49 | -4.55 | 0.08 | -4.24 | -4.81 | -4.52 | 0.40 | -0.02 |
| P20852 | CYP2A5 | 56.74 | -0.43 | -0.71 | -0.57 | 0.19 | -0.34 | -0.39 | -0.36 | 0.04 | -0.21 |

| P56593 | CYP2A12 | 56.18 | -0.37 | -0.35 | -0.36 | 0.01 | -0.51 | -0.49 | -0.50 | 0.01 | 0.14 |
| --- | --- | --- | --- | --- | --- | --- | --- | --- | --- | --- | --- |
| Q9DBG1 | CYP27A1 | 60.72 | 1.41 | 1.42 | 1.42 | 0.01 | 1.33 | 1.48 | 1.41 | 0.11 | 0.01 |
| P00186 | CYP1A2 | 58.18 | 0.59 | 0.72 | 0.65 | 0.09 | 0.56 | 0.70 | 0.63 | 0.10 | 0.03 |
| P27786 | CYP17A1 | 57.64 | -2.90 | -2.58 | -2.74 | 0.23 | -1.94 | -1.94 | -1.94 | 0.00 | -0.79 |
| Q7TMB8 | CYFIP1 | 145.24 | 0.11 | 0.20 | 0.15 | 0.06 | 0.25 | 0.06 | 0.15 | 0.13 | 0.00 |
| P62897 | CYCS | 11.61 | -0.05 | 0.00 | -0.02 | 0.03 | -0.06 | 0.00 | -0.03 | 0.04 | 0.01 |
| Q9D0M3 | CYC1 | 35.33 | 0.12 | 0.11 | 0.11 | 0.01 | 0.10 | 0.20 | 0.15 | 0.07 | -0.03 |
| Q9DCN2 | CYB5R3 | 34.13 | 0.03 | 0.10 | 0.07 | 0.05 | -0.03 | 0.04 | 0.01 | 0.04 | 0.06 |
| Q9CQX2 | CYB5B | 16.32 | 0.31 | 0.39 | 0.35 | 0.06 | 0.26 | 0.38 | 0.32 | 0.09 | 0.03 |
| P56395 | CYB5A | 15.24 | 0.10 | 0.11 | 0.10 | 0.01 | 0.52 | 0.20 | 0.36 | 0.23 | -0.26 |
| P97792 | CXADR | 39.95 | -0.18 | -0.46 | -0.32 | 0.20 | -0.26 | -0.18 | -0.22 | 0.06 | -0.10 |
| Q3TKY6 | CWC27 | 53.54 | -0.49 | -0.49 | -0.49 | 0.00 | -0.30 | -0.38 | -0.34 | 0.06 | -0.15 |
| Q9D8X1 | CUTC | 29.00 | 0.10 | 0.27 | 0.19 | 0.12 | 0.16 | 0.37 | 0.26 | 0.15 | -0.08 |

| Q9D5V5 | CUL5 | 90.97 | 0.08 | -0.04 | 0.02 | 0.08 | 0.05 | -0.05 | 0.00 | 0.07 | 0.01 |
| --- | --- | --- | --- | --- | --- | --- | --- | --- | --- | --- | --- |
| Q3TCH7 | CUL4A | 87.75 | -0.28 | -0.39 | -0.33 | 0.08 | -0.37 | -0.39 | -0.38 | 0.01 | 0.05 |
| Q9JLV5 | CUL3 | 88.95 | 0.12 | 0.11 | 0.12 | 0.01 | 0.09 | 0.13 | 0.11 | 0.03 | 0.01 |
| Q9D4H8 | CUL2 | 86.88 | 0.14 | 0.00 | 0.07 | 0.10 | 0.03 | 0.04 | 0.03 | 0.01 | 0.04 |
| Q9WTX6 | CUL1 | 89.69 | 0.07 | 0.01 | 0.04 | 0.04 | 0.04 | 0.05 | 0.05 | 0.00 | -0.01 |
| Q60598 | CTTN | 61.25 | 0.11 | 0.05 | 0.08 | 0.04 | 0.38 | 0.27 | 0.33 | 0.08 | -0.25 |
| Q9WUU7 | CTSZ | 34.00 | 0.84 | 0.96 | 0.90 | 0.08 | 0.85 | 1.10 | 0.97 | 0.17 | -0.08 |
| P06797 | CTSL | 37.55 | 0.39 | 0.37 | 0.38 | 0.02 | 0.75 | 0.74 | 0.74 | 0.00 | -0.36 |
| P49935 | CTSH | 37.17 | 0.81 | 0.66 | 0.74 | 0.10 | 0.60 | 0.55 | 0.57 | 0.04 | 0.16 |
| P18242 | CTSD | 44.95 | 0.72 | 0.64 | 0.68 | 0.05 | 0.59 | 0.50 | 0.54 | 0.06 | 0.14 |
| P10605 | CTSB | 37.28 | 0.40 | 0.48 | 0.44 | 0.05 | 0.47 | 0.55 | 0.51 | 0.05 | -0.07 |
| P16675 | CTSA | 53.84 | 0.88 | 0.96 | 0.92 | 0.06 | 0.94 | 0.89 | 0.91 | 0.03 | 0.00 |
| P30999 | CTNND1 | 104.92 | 0.02 | 0.17 | 0.10 | 0.11 | 0.29 | 0.24 | 0.26 | 0.04 | -0.17 |

| Q9CWL8 | CTNNBL1 | 64.98 | 0.13 | -0.13 | 0.00 | 0.18 | 0.12 | -0.04 | 0.04 | 0.11 | -0.04 |
| --- | --- | --- | --- | --- | --- | --- | --- | --- | --- | --- | --- |
| Q02248 | CTNNB1 | 85.47 | -0.03 | 0.05 | 0.01 | 0.06 | 0.02 | 0.15 | 0.09 | 0.09 | -0.08 |
| P26231 | CTNNA1 | 100.11 | 0.16 | 0.09 | 0.12 | 0.05 | 0.27 | 0.18 | 0.22 | 0.06 | -0.10 |
| Q6PEE2 | CTIF | 67.83 | 0.12 | 0.24 | 0.18 | 0.09 | 0.30 | 0.24 | 0.27 | 0.04 | -0.09 |
| Q8VCN5 | CTH | 43.57 | 1.10 | 1.11 | 1.11 | 0.01 | 1.16 | 1.18 | 1.17 | 0.02 | -0.07 |
| Q8R311 | CTAGE5 | 87.72 | 0.27 | 0.38 | 0.33 | 0.07 | 0.01 | 0.28 | 0.15 | 0.20 | 0.18 |
| P97315 | CSRP1 | 20.58 | 0.06 | -0.05 | 0.00 | 0.08 | 0.37 | 0.01 | 0.19 | 0.25 | -0.18 |
| O54833 | CSNK2A2 | 41.22 | -0.16 | 0.07 | -0.04 | 0.16 | 0.40 | 0.05 | 0.22 | 0.25 | -0.26 |
| Q60737 | CSNK2A1 | 45.13 | -0.11 | 0.10 | 0.00 | 0.15 | -0.10 | 0.06 | -0.02 | 0.11 | 0.02 |
| P41241 | CSK | 50.72 | 0.10 | 0.18 | 0.14 | 0.06 | 0.06 | 0.10 | 0.08 | 0.03 | 0.06 |
| Q9ERK4 | CSE1L | 110.45 | -0.07 | -0.18 | -0.12 | 0.08 | -0.14 | -0.14 | -0.14 | 0.00 | 0.01 |
| Q91W50 | CSDE1 | 88.79 | -0.13 | 0.10 | -0.01 | 0.17 | 0.06 | 0.14 | 0.10 | 0.05 | -0.12 |
| Q9DBE0 | CSAD | 55.14 | 3.41 | 3.54 | 3.48 | 0.09 | 2.91 | 3.03 | 2.97 | 0.08 | 0.50 |

| Q9CZU6 | CS | 51.74 | -0.18 | -0.11 | -0.14 | 0.05 | -0.26 | -0.22 | -0.24 | 0.03 | 0.10 |
| --- | --- | --- | --- | --- | --- | --- | --- | --- | --- | --- | --- |
| P47199 | CRYZ | 35.27 | 0.15 | 0.04 | 0.10 | 0.08 | -0.04 | 0.01 | -0.02 | 0.03 | 0.11 |
| Q99KP3 | CRYL1 | 35.21 | 1.35 | 1.83 | 1.59 | 0.34 | 1.63 | 1.70 | 1.67 | 0.05 | -0.07 |
| Q9DC50 | CROT | 70.26 | 0.25 | 0.11 | 0.18 | 0.10 | -0.18 | -0.17 | -0.17 | 0.00 | 0.35 |
| P47941 | CRKL | 33.83 | -0.05 | -0.35 | -0.20 | 0.21 | -0.31 | -0.20 | -0.25 | 0.08 | 0.05 |
| Q9DCT8 | CRIP2 | 22.73 | -0.41 | -0.45 | -0.43 | 0.03 | -0.31 | -0.16 | -0.23 | 0.10 | -0.20 |
| Q9CYA0 | CRELD2 | 38.22 | 0.10 | 0.01 | 0.05 | 0.07 | 0.00 | -0.05 | -0.03 | 0.04 | 0.08 |
| P47934 | CRAT | 70.84 | -0.05 | 0.05 | 0.00 | 0.07 | -0.41 | -0.27 | -0.34 | 0.09 | 0.34 |
| P52825 | CPT2 | 73.98 | 0.50 | 0.40 | 0.45 | 0.07 | 0.43 | 0.32 | 0.38 | 0.07 | 0.07 |
| P97742 | CPT1A | 88.25 | 0.03 | -0.09 | -0.03 | 0.09 | -0.06 | -0.06 | -0.06 | 0.00 | 0.03 |
| Q8C196 | CPS1 | 164.62 | 0.07 | 0.49 | 0.28 | 0.30 | 0.27 | 0.69 | 0.48 | 0.29 | -0.20 |
| Q9WVJ3 | CPQ | 51.81 | 1.09 | 1.08 | 1.08 | 0.01 | 0.90 | 0.84 | 0.87 | 0.04 | 0.21 |
| Q8BFS6 | CPPED1 | 35.25 | -0.28 | -0.26 | -0.27 | 0.01 | -0.22 | -0.19 | -0.21 | 0.02 | -0.07 |

| P36552 | CPOX | 49.71 | -0.11 | -0.14 | -0.12 | 0.03 | -0.08 | 0.13 | 0.03 | 0.15 | -0.15 |
| --- | --- | --- | --- | --- | --- | --- | --- | --- | --- | --- | --- |
| Q8BT60 | CPNE3 | 59.58 | -0.21 | -0.11 | -0.16 | 0.07 | -0.10 | -0.05 | -0.08 | 0.03 | -0.09 |
| Q8C166 | CPNE1 | 58.89 | -0.26 | -0.18 | -0.22 | 0.05 | -0.24 | -0.31 | -0.27 | 0.05 | 0.06 |
| Q61147 | CP | 121.15 | 0.13 | 0.40 | 0.26 | 0.19 | 0.30 | 0.30 | 0.30 | 0.00 | -0.04 |
| P48771 | COX7A2 | 9.29 | -0.06 | 0.15 | 0.05 | 0.15 | -0.03 | 0.14 | 0.06 | 0.12 | -0.01 |
| Q9CPQ1 | COX6C | 8.47 | -0.13 | 0.02 | -0.06 | 0.11 | -0.03 | 0.01 | -0.01 | 0.03 | -0.05 |
| P19536 | COX5B | 13.81 | -0.25 | -0.04 | -0.14 | 0.15 | -0.01 | 0.05 | 0.02 | 0.04 | -0.16 |
| P12787 | COX5A | 16.10 | -0.03 | -0.04 | -0.03 | 0.01 | 0.00 | 0.08 | 0.04 | 0.06 | -0.07 |
| P19783 | COX4I1 | 19.53 | -0.02 | 0.04 | 0.01 | 0.04 | 0.02 | 0.07 | 0.04 | 0.04 | -0.04 |
| Q9CQI6 | COTL1 | 15.94 | -0.61 | -0.32 | -0.46 | 0.20 | -0.11 | -0.43 | -0.27 | 0.22 | -0.20 |
| Q9D2V7 | CORO7 | 100.81 | 0.11 | 0.03 | 0.07 | 0.06 | 0.03 | 0.12 | 0.07 | 0.06 | 0.00 |
| Q9WUM4 | CORO1C | 53.12 | -0.09 | -0.01 | -0.05 | 0.05 | 0.08 | -0.03 | 0.03 | 0.07 | -0.08 |
| Q9WUM3 | CORO1B | 53.91 | 0.01 | 0.03 | 0.02 | 0.01 | 0.15 | 0.02 | 0.08 | 0.09 | -0.06 |

| O89053 | CORO1A | 50.99 | -0.13 | -0.24 | -0.19 | 0.08 | 0.51 | -0.21 | 0.15 | 0.51 | -0.34 |
| --- | --- | --- | --- | --- | --- | --- | --- | --- | --- | --- | --- |
| Q8K1Z0 | COQ9 | 35.08 | -0.21 | -0.27 | -0.24 | 0.04 | -0.28 | -0.20 | -0.24 | 0.06 | 0.00 |
| Q8R1S0 | COQ6 | 51.39 | 0.24 | 0.04 | 0.14 | 0.14 | 0.13 | 0.09 | 0.11 | 0.03 | 0.03 |
| Q9CXI0 | COQ5 | 37.34 | -0.12 | -0.14 | -0.13 | 0.01 | -0.11 | 0.16 | 0.03 | 0.19 | -0.16 |
| Q8BMS4 | COQ3 | 40.96 | -0.15 | 0.10 | -0.03 | 0.18 | 0.00 | 0.04 | 0.02 | 0.02 | -0.05 |
| Q9JHH9 | COPZ2 | 22.93 | -0.52 | -0.25 | -0.39 | 0.19 | -0.36 | -0.31 | -0.33 | 0.04 | -0.05 |
| Q9CZ04 | COPS7A | 30.22 | -0.22 | -0.19 | -0.21 | 0.02 | -0.16 | -0.12 | -0.14 | 0.03 | -0.07 |
| O35864 | COPS5 | 37.55 | -0.13 | -0.24 | -0.19 | 0.08 | -0.27 | -0.26 | -0.27 | 0.01 | 0.08 |
| O88544 | COPS4 | 46.28 | -0.19 | -0.15 | -0.17 | 0.03 | -0.25 | -0.21 | -0.23 | 0.03 | 0.06 |
| O88543 | COPS3 | 47.83 | -0.11 | -0.14 | -0.13 | 0.02 | -0.17 | -0.24 | -0.21 | 0.05 | 0.08 |
| P61202 | COPS2 | 51.60 | -0.19 | -0.20 | -0.19 | 0.01 | -0.24 | -0.24 | -0.24 | 0.00 | 0.05 |
| Q9QXK3 | COPG2 | 97.68 | 0.05 | 0.02 | 0.04 | 0.02 | 0.08 | 0.01 | 0.04 | 0.05 | 0.00 |
| Q9QZE5 | COPG1 | 97.51 | 0.15 | 0.16 | 0.15 | 0.01 | 0.03 | 0.10 | 0.07 | 0.05 | 0.09 |

| O89079 | COPE | 34.57 | 0.22 | 0.16 | 0.19 | 0.04 | 0.22 | 0.12 | 0.17 | 0.07 | 0.03 |
| --- | --- | --- | --- | --- | --- | --- | --- | --- | --- | --- | --- |
| O55029 | COPB2 | 102.45 | 0.23 | 0.10 | 0.17 | 0.09 | 0.22 | 0.13 | 0.17 | 0.07 | -0.01 |
| Q9JIF7 | COPB1 | 107.06 | 0.25 | 0.08 | 0.16 | 0.12 | 0.09 | 0.14 | 0.12 | 0.04 | 0.04 |
| Q8CIE6 | COPA | 138.43 | -0.24 | 0.06 | -0.09 | 0.21 | -0.09 | 0.10 | 0.01 | 0.13 | -0.10 |
| O88587 | COMT | 29.49 | 0.10 | 0.52 | 0.31 | 0.30 | 0.04 | 0.41 | 0.23 | 0.27 | 0.08 |
| Q9CQ02 | COMMD4 | 21.86 | -0.07 | -0.06 | -0.06 | 0.00 | 0.05 | 0.02 | 0.04 | 0.02 | -0.10 |
| Q8BXC6 | COMMD2 | 22.85 | -0.26 | -0.18 | -0.22 | 0.06 | -0.04 | -0.14 | -0.09 | 0.07 | -0.13 |
| Q8K297 | COLGALT1 | 71.06 | -0.02 | 0.00 | -0.01 | 0.01 | 0.07 | -0.04 | 0.02 | 0.08 | -0.02 |
| Q02788 | COL6A2 | 110.33 | -0.27 | 0.46 | 0.10 | 0.52 | 0.58 | 0.76 | 0.67 | 0.12 | -0.57 |
| Q04857 | COL6A1 | 108.49 | -0.19 | 0.30 | 0.05 | 0.35 | 0.81 | 0.75 | 0.78 | 0.04 | -0.73 |
| Q9EQG9 | COL4A3BP | 71.11 | -0.91 | -0.73 | -0.82 | 0.12 | -0.87 | -0.69 | -0.78 | 0.13 | -0.04 |
| Q80X19 | COL14A1 | 193.01 | -0.73 | -0.73 | -0.73 | 0.00 | -0.38 | -0.52 | -0.45 | 0.10 | -0.28 |
| Q8C0L8 | COG5 | 91.39 | 0.41 | 0.19 | 0.30 | 0.16 | 0.02 | 0.07 | 0.04 | 0.04 | 0.26 |

| Q3UMF0 | COBLL1 | 137.38 | 0.07 | 0.50 | 0.29 | 0.30 | 0.40 | 0.68 | 0.54 | 0.20 | -0.25 |
| --- | --- | --- | --- | --- | --- | --- | --- | --- | --- | --- | --- |
| Q9DBL7 | COASY | 62.02 | 0.09 | 0.18 | 0.14 | 0.06 | -0.07 | 0.02 | -0.03 | 0.07 | 0.16 |
| Q9DAU1 | CNPY3 | 30.54 | -0.18 | -0.17 | -0.18 | 0.00 | -0.24 | -0.50 | -0.37 | 0.19 | 0.19 |
| P16330 | CNP | 47.12 | 0.04 | 0.09 | 0.07 | 0.03 | 0.45 | 0.29 | 0.37 | 0.12 | -0.30 |
| Q6ZQ08 | CNOT1 | 266.80 | 0.16 | 0.04 | 0.10 | 0.08 | -0.24 | 0.05 | -0.10 | 0.20 | 0.19 |
| Q9DAW9 | CNN3 | 36.43 | -0.65 | -0.45 | -0.55 | 0.14 | -0.19 | -0.26 | -0.23 | 0.06 | -0.33 |
| Q9D1A2 | CNDP2 | 52.77 | -0.55 | -0.46 | -0.51 | 0.06 | -0.52 | -0.58 | -0.55 | 0.04 | 0.04 |
| Q3U5Q7 | CMPK2 | 50.04 | -1.20 | -1.03 | -1.11 | 0.12 | -1.15 | -1.22 | -1.18 | 0.05 | 0.07 |
| Q9DBP5 | CMPK1 | 22.17 | 0.12 | 0.18 | 0.15 | 0.05 | 0.04 | 0.05 | 0.04 | 0.00 | 0.10 |
| Q8CHQ9 | CML2 | 26.42 | 1.13 | 1.43 | 1.28 | 0.21 | 1.01 | 1.39 | 1.20 | 0.27 | 0.08 |
| Q8R1G2 | CMBL | 27.90 | 0.41 | 0.38 | 0.40 | 0.02 | 0.26 | 0.34 | 0.30 | 0.06 | 0.10 |
| Q99KK2 | CMAS | 48.06 | 0.63 | 0.57 | 0.60 | 0.05 | 0.58 | 0.54 | 0.56 | 0.03 | 0.04 |
| Q61419 | CMAH | 66.94 | 0.04 | 0.04 | 0.04 | 0.01 | 0.24 | 0.19 | 0.22 | 0.04 | -0.18 |

| Q8R4N0 | CLYBL | 37.55 | 0.39 | 0.51 | 0.45 | 0.08 | 0.35 | 0.52 | 0.43 | 0.12 | 0.02 |
| --- | --- | --- | --- | --- | --- | --- | --- | --- | --- | --- | --- |
| Q5SW19 | CLUH | 148.07 | -0.11 | 0.07 | -0.02 | 0.13 | 0.02 | 0.13 | 0.08 | 0.07 | -0.10 |
| Q06890 | CLU | 51.66 | 1.18 | 1.16 | 1.17 | 0.02 | 1.24 | 0.88 | 1.06 | 0.25 | 0.11 |
| Q68FD5 | CLTC | 191.55 | -0.19 | 0.04 | -0.07 | 0.16 | -0.04 | 0.12 | 0.04 | 0.11 | -0.11 |
| Q6IRU5 | CLTB | 25.17 | 0.39 | 0.42 | 0.41 | 0.02 | 0.42 | 0.48 | 0.45 | 0.05 | -0.04 |
| O08585 | CLTA | 25.60 | 0.02 | -0.22 | -0.10 | 0.17 | 0.03 | 0.11 | 0.07 | 0.06 | -0.17 |
| Q9JHS4 | CLPX | 69.23 | 0.02 | 0.10 | 0.06 | 0.05 | -0.06 | 0.10 | 0.02 | 0.12 | 0.04 |
| Q8VBZ3 | CLPTM1 | 75.29 | 0.16 | 0.00 | 0.08 | 0.11 | 0.15 | -0.01 | 0.07 | 0.12 | 0.02 |
| O88696 | CLPP | 29.80 | 0.62 | 0.72 | 0.67 | 0.07 | 0.41 | 0.63 | 0.52 | 0.16 | 0.15 |
| Q60649 | CLPB | 76.00 | -0.13 | -0.12 | -0.13 | 0.01 | -0.11 | -0.18 | -0.15 | 0.05 | 0.02 |
| Q8C5W0 | CLMN | 117.23 | -0.28 | 0.36 | 0.04 | 0.46 | 0.11 | 0.40 | 0.25 | 0.21 | -0.21 |
| Q922J3 | CLIP1 | 155.81 | -0.57 | -0.05 | -0.31 | 0.37 | -0.45 | -0.08 | -0.26 | 0.27 | -0.04 |
| Q99KN9 | CLINT1 | 68.51 | -0.31 | -0.15 | -0.23 | 0.11 | -0.73 | -0.14 | -0.44 | 0.42 | 0.21 |

| Q9QYB1 | CLIC4 | 28.73 | 0.09 | 0.05 | 0.07 | 0.02 | 0.19 | 0.16 | 0.17 | 0.02 | -0.10 |
| --- | --- | --- | --- | --- | --- | --- | --- | --- | --- | --- | --- |
| Q9Z1Q5 | CLIC1 | 27.01 | 0.04 | 0.00 | 0.02 | 0.03 | 0.05 | -0.09 | -0.02 | 0.10 | 0.04 |
| P70194 | CLEC4F | 61.27 | -0.61 | -0.73 | -0.67 | 0.08 | -0.40 | -0.62 | -0.51 | 0.16 | -0.16 |
| Q99LI2 | CLCC1 | 60.62 | -0.47 | -0.29 | -0.38 | 0.13 | -0.29 | -0.28 | -0.29 | 0.01 | -0.10 |
| Q04447 | CKB | 42.71 | -0.46 | -0.52 | -0.49 | 0.04 | -0.22 | -0.48 | -0.35 | 0.19 | -0.14 |
| Q8BMK4 | CKAP4 | 63.69 | -1.29 | -0.96 | -1.13 | 0.23 | -0.77 | -0.95 | -0.86 | 0.12 | -0.27 |
| Q9CQB5 | CISD2 | 15.24 | 0.28 | 0.02 | 0.15 | 0.18 | -0.01 | 0.02 | 0.01 | 0.02 | 0.14 |
| Q91WS0 | CISD1 | 12.10 | 0.03 | 0.09 | 0.06 | 0.04 | 0.00 | 0.05 | 0.03 | 0.04 | 0.03 |
| Q8WTY4 | CIAPIN1 | 33.43 | -0.60 | -0.22 | -0.41 | 0.27 | 0.11 | -0.03 | 0.04 | 0.10 | -0.45 |
| Q9CY57 | CHTOP | 26.59 | -0.40 | -0.18 | -0.29 | 0.15 | -0.30 | -0.21 | -0.25 | 0.06 | -0.04 |
| Q9D1P4 | CHORDC1 | 37.35 | -0.08 | -0.31 | -0.20 | 0.16 | -0.26 | -0.36 | -0.31 | 0.07 | 0.11 |
| Q9CQ10 | CHMP3 | 25.22 | 0.09 | 0.10 | 0.10 | 0.01 | 0.09 | 0.08 | 0.08 | 0.01 | 0.01 |
| Q9DB34 | CHMP2A | 25.13 | -0.13 | -0.12 | -0.12 | 0.01 | -0.23 | -0.16 | -0.19 | 0.05 | 0.07 |

| O35744 | CHIL3 | 44.46 | 2.98 | 3.53 | 3.26 | 0.39 | 3.26 | 3.91 | 3.59 | 0.46 | -0.33 |
| --- | --- | --- | --- | --- | --- | --- | --- | --- | --- | --- | --- |
| Q922Q9 | CHID1 | 44.91 | 0.01 | 0.01 | 0.01 | 0.00 | -0.12 | -0.37 | -0.25 | 0.17 | 0.25 |
| Q8CGZ0 | CHERP | 106.17 | -0.04 | -0.07 | -0.06 | 0.02 | -0.17 | 0.03 | -0.07 | 0.14 | 0.02 |
| Q8BJ64 | CHDH | 66.41 | 0.16 | 0.19 | 0.18 | 0.02 | 0.11 | 0.18 | 0.15 | 0.05 | 0.03 |
| Q6PDQ2 | CHD4 | 217.75 | 0.02 | -0.14 | -0.06 | 0.11 | 0.11 | -0.05 | 0.03 | 0.11 | -0.09 |
| Q9CRB9 | CHCHD3 | 26.33 | 0.02 | 0.10 | 0.06 | 0.06 | -0.09 | 0.03 | -0.03 | 0.09 | 0.09 |
| P59242 | CGN | 136.45 | -0.54 | 0.03 | -0.25 | 0.41 | -0.03 | -0.07 | -0.05 | 0.03 | -0.20 |
| P45591 | CFL2 | 18.71 | -0.10 | -0.02 | -0.06 | 0.06 | -0.09 | 0.04 | -0.03 | 0.09 | -0.03 |
| P18760 | CFL1 | 18.56 | 1.63 | 1.46 | 1.54 | 0.11 | -1.63 | 0.01 | -0.81 | 1.16 | 2.35 |
| P06909 | CFH | 139.14 | -0.17 | 0.10 | -0.03 | 0.19 | -0.18 | 0.10 | -0.04 | 0.20 | 0.00 |
| P04186 | CFB | 85.00 | 0.27 | 0.00 | 0.14 | 0.19 | -0.11 | -0.19 | -0.15 | 0.06 | 0.28 |
| Q8VCU1 | CES3B | 63.35 | 1.62 | 1.75 | 1.68 | 0.09 | 1.93 | 1.94 | 1.94 | 0.01 | -0.25 |
| Q63880 | CES3A | 63.32 | 1.18 | 1.08 | 1.13 | 0.07 | 0.99 | 1.11 | 1.05 | 0.09 | 0.08 |

| Q8BK48 | CES2E | 62.32 | -0.06 | -0.07 | -0.07 | 0.01 | -0.44 | -0.42 | -0.43 | 0.02 | 0.37 |
| --- | --- | --- | --- | --- | --- | --- | --- | --- | --- | --- | --- |
| Q91WG0 | CES2C | 62.47 | 3.09 | 3.36 | 3.22 | 0.19 | 2.85 | 2.84 | 2.85 | 0.01 | 0.38 |
| Q8QZR3 | CES2A | 61.94 | 1.26 | 1.36 | 1.31 | 0.07 | 1.32 | 1.48 | 1.40 | 0.11 | -0.09 |
| Q64176 | CES1E | 61.58 | 1.06 | 1.10 | 1.08 | 0.03 | 0.87 | 0.95 | 0.91 | 0.06 | 0.17 |
| Q8VCT4 | CES1D | 61.79 | 0.03 | 0.00 | 0.01 | 0.02 | -0.11 | -0.14 | -0.12 | 0.02 | 0.14 |
| P23953 | CES1C | 61.06 | -0.27 | -0.37 | -0.32 | 0.07 | -0.70 | -0.66 | -0.68 | 0.03 | 0.36 |
| Q8VCC2 | CES1 | 62.68 | -0.44 | -0.45 | -0.45 | 0.01 | -0.49 | -0.53 | -0.51 | 0.03 | 0.06 |
| Q924Z4 | CERS2 | 45.02 | -0.59 | -0.35 | -0.47 | 0.17 | -0.75 | -0.25 | -0.50 | 0.35 | 0.03 |
| Q9CXS4 | CENPV | 27.54 | 0.44 | 0.44 | 0.44 | 0.01 | 0.66 | 0.58 | 0.62 | 0.06 | -0.18 |
| P28659 | CELF1 | 52.11 | -0.14 | -0.11 | -0.13 | 0.02 | -0.16 | 0.07 | -0.04 | 0.16 | -0.08 |
| Q4VAA2 | CDV3 | 29.73 | -0.37 | 0.33 | -0.02 | 0.49 | -0.22 | -0.25 | -0.24 | 0.03 | 0.21 |
| P60334 | CDO1 | 23.03 | -0.93 | -0.91 | -0.92 | 0.01 | -0.55 | -0.59 | -0.57 | 0.03 | -0.35 |
| Q91WE6 | CDKAL1 | 65.29 | 0.01 | -0.18 | -0.08 | 0.14 | -0.31 | -0.13 | -0.22 | 0.13 | 0.14 |

| Q64261 | CDK6 | 37.03 | -0.83 | -1.10 | -0.96 | 0.19 | -1.01 | -1.26 | -1.13 | 0.17 | 0.17 |
| --- | --- | --- | --- | --- | --- | --- | --- | --- | --- | --- | --- |
| Q99LM2 | CDK5RAP3 | 56.99 | -0.03 | -0.17 | -0.10 | 0.10 | -0.01 | -0.11 | -0.06 | 0.07 | -0.04 |
| P49615 | CDK5 | 33.29 | -0.10 | -0.05 | -0.07 | 0.03 | -0.17 | 0.10 | -0.04 | 0.19 | -0.04 |
| P15116 | CDH2 | 99.80 | -0.27 | 0.38 | 0.05 | 0.46 | 0.15 | 0.40 | 0.27 | 0.18 | -0.22 |
| P09803 | CDH1 | 98.26 | -0.87 | -0.65 | -0.76 | 0.16 | -0.51 | -0.43 | -0.47 | 0.06 | -0.29 |
| Q8JZM7 | CDC73 | 60.58 | -0.74 | -0.23 | -0.49 | 0.36 | -0.28 | -0.13 | -0.20 | 0.10 | -0.28 |
| Q6A068 | CDC5L | 92.19 | -0.04 | -0.03 | -0.03 | 0.00 | 0.15 | 0.05 | 0.10 | 0.07 | -0.13 |
| Q7TT50 | CDC42BPB | 194.75 | 0.01 | 0.18 | 0.09 | 0.12 | 0.25 | 0.19 | 0.22 | 0.04 | -0.13 |
| P60766 | CDC42 | 21.26 | 1.08 | 1.02 | 1.05 | 0.04 | -1.08 | -0.02 | -0.55 | 0.75 | 1.60 |
| Q61081 | CDC37 | 44.59 | -0.21 | -0.25 | -0.23 | 0.03 | -0.25 | -0.27 | -0.26 | 0.02 | 0.03 |
| P40237 | CD82 | 29.63 | 0.45 | 0.25 | 0.35 | 0.15 | 0.68 | 0.43 | 0.56 | 0.17 | -0.21 |
| Q61735 | CD47 | 33.10 | -0.10 | -0.05 | -0.07 | 0.03 | 0.13 | -0.05 | 0.04 | 0.12 | -0.12 |
| Q08857 | CD36 | 52.70 | 0.07 | -0.15 | -0.04 | 0.15 | -0.19 | -0.43 | -0.31 | 0.17 | 0.27 |

| Q9JLQ0 | CD2AP | 70.45 | -0.77 | -0.41 | -0.59 | 0.25 | -0.49 | -0.30 | -0.40 | 0.14 | -0.19 |
| --- | --- | --- | --- | --- | --- | --- | --- | --- | --- | --- | --- |
| P42932 | CCT8 | 59.56 | -0.42 | -0.38 | -0.40 | 0.03 | -0.52 | -0.43 | -0.47 | 0.06 | 0.07 |
| P80313 | CCT7 | 59.65 | -0.43 | -0.39 | -0.41 | 0.03 | -0.51 | -0.43 | -0.47 | 0.05 | 0.06 |
| Q61390 | CCT6B | 58.18 | -0.47 | -0.32 | -0.39 | 0.10 | -0.51 | -0.45 | -0.48 | 0.04 | 0.09 |
| P80317 | CCT6A | 58.00 | -0.38 | -0.38 | -0.38 | 0.00 | -0.41 | -0.44 | -0.43 | 0.02 | 0.05 |
| P80316 | CCT5 | 59.62 | -0.42 | -0.37 | -0.40 | 0.04 | -0.55 | -0.47 | -0.51 | 0.06 | 0.11 |
| P80315 | CCT4 | 58.07 | -0.48 | -0.40 | -0.44 | 0.06 | -0.52 | -0.44 | -0.48 | 0.06 | 0.04 |
| P80318 | CCT3 | 60.63 | -0.45 | -0.33 | -0.39 | 0.08 | -0.44 | -0.44 | -0.44 | 0.00 | 0.06 |
| P80314 | CCT2 | 57.48 | -0.47 | -0.40 | -0.44 | 0.05 | -0.49 | -0.42 | -0.46 | 0.05 | 0.02 |
| Q9WU84 | CCS | 28.91 | -0.04 | 0.17 | 0.06 | 0.14 | 0.09 | 0.25 | 0.17 | 0.11 | -0.11 |
| Q7TQK5 | CCDC93 | 72.60 | 0.05 | 0.00 | 0.02 | 0.03 | -0.02 | 0.07 | 0.02 | 0.06 | 0.00 |
| Q8C3X2 | CCDC90B | 29.60 | -0.22 | -0.12 | -0.17 | 0.07 | -0.07 | -0.08 | -0.07 | 0.01 | -0.09 |
| Q9D024 | CCDC47 | 55.84 | 0.19 | 0.23 | 0.21 | 0.03 | 0.08 | 0.20 | 0.14 | 0.08 | 0.06 |

| Q78PG9 | CCDC25 | 24.48 | -0.17 | -0.02 | -0.09 | 0.11 | -0.18 | -0.04 | -0.11 | 0.10 | 0.01 |
| --- | --- | --- | --- | --- | --- | --- | --- | --- | --- | --- | --- |
| Q9JIG7 | CCDC22 | 70.84 | 0.19 | -0.07 | 0.06 | 0.18 | 0.02 | 0.22 | 0.12 | 0.15 | -0.06 |
| Q8C7V8 | CCDC134 | 26.49 | -0.09 | 0.10 | 0.00 | 0.13 | -0.10 | 0.09 | -0.01 | 0.13 | 0.01 |
| Q9D8X2 | CCDC124 | 25.35 | 0.09 | 0.25 | 0.17 | 0.11 | 0.29 | 0.12 | 0.20 | 0.12 | -0.03 |
| Q71RI9 | CCBL2 | 51.13 | -0.86 | -0.87 | -0.87 | 0.01 | -0.90 | -0.89 | -0.89 | 0.01 | 0.03 |
| Q8BTY1 | CCBL1 | 47.56 | 0.00 | -0.03 | -0.01 | 0.02 | 0.19 | 0.21 | 0.20 | 0.02 | -0.22 |
| Q8VDP4 | CCAR2 | 103.00 | -0.26 | -0.17 | -0.21 | 0.06 | -0.19 | -0.06 | -0.12 | 0.10 | -0.09 |
| Q8CH18 | CCAR1 | 132.06 | -0.53 | -0.10 | -0.31 | 0.30 | 0.20 | -0.03 | 0.08 | 0.16 | -0.40 |
| P83917 | CBX1 | 21.42 | -0.44 | -0.55 | -0.49 | 0.08 | -0.33 | -0.29 | -0.31 | 0.03 | -0.18 |
| Q91WT9 | CBS | 61.54 | 0.23 | 0.21 | 0.22 | 0.01 | 0.30 | 0.39 | 0.34 | 0.06 | -0.12 |
| Q91VT4 | CBR4 | 25.41 | 0.04 | 0.14 | 0.09 | 0.06 | 0.02 | 0.09 | 0.06 | 0.05 | 0.03 |
| P48758 | CBR1 | 30.64 | -0.74 | -0.67 | -0.70 | 0.05 | -0.67 | -0.64 | -0.66 | 0.02 | -0.04 |
| P24270 | CAT | 59.80 | 0.08 | 0.07 | 0.07 | 0.00 | 0.12 | 0.11 | 0.12 | 0.01 | -0.05 |

| P51125 | CAST | 84.92 | -0.29 | -0.07 | -0.18 | 0.16 | -0.20 | -0.06 | -0.13 | 0.10 | -0.05 |
| --- | --- | --- | --- | --- | --- | --- | --- | --- | --- | --- | --- |
| O89110 | CASP8 | 55.36 | -0.20 | -0.14 | -0.17 | 0.05 | -0.44 | -0.23 | -0.33 | 0.15 | 0.16 |
| O08738 | CASP6 | 31.60 | -0.40 | -0.42 | -0.41 | 0.01 | -0.40 | -0.18 | -0.29 | 0.16 | -0.12 |
| P70677 | CASP3 | 31.47 | -0.49 | -0.52 | -0.50 | 0.02 | -0.31 | -0.39 | -0.35 | 0.05 | -0.16 |
| O70589 | CASK | 105.11 | 0.90 | 0.90 | 0.90 | 0.00 | 0.99 | 0.98 | 0.98 | 0.00 | -0.09 |
| Q9ER72 | CARS | 94.86 | -0.19 | -0.20 | -0.20 | 0.01 | -0.34 | -0.36 | -0.35 | 0.01 | 0.15 |
| P47757 | CAPZB | 31.35 | -0.13 | -0.12 | -0.12 | 0.01 | -0.04 | -0.12 | -0.08 | 0.06 | -0.04 |
| P47754 | CAPZA2 | 32.97 | -0.01 | -0.04 | -0.03 | 0.02 | 0.09 | 0.07 | 0.08 | 0.02 | -0.11 |
| P47753 | CAPZA1 | 32.94 | -0.21 | -0.26 | -0.24 | 0.03 | -0.36 | -0.16 | -0.26 | 0.14 | 0.02 |
| Q60865 | CAPRIN1 | 78.17 | -0.14 | -0.10 | -0.12 | 0.02 | -0.31 | -0.14 | -0.23 | 0.12 | 0.11 |
| O08529 | CAPN2 | 79.87 | 0.08 | -0.05 | 0.02 | 0.09 | -0.06 | -0.10 | -0.08 | 0.03 | 0.10 |
| O35350 | CAPN1 | 82.11 | 0.62 | 0.52 | 0.57 | 0.07 | 0.61 | 0.44 | 0.52 | 0.12 | 0.05 |
| P24452 | CAPG | 39.24 | 0.18 | 0.14 | 0.16 | 0.03 | 0.79 | 0.08 | 0.43 | 0.50 | -0.27 |

| P40124 | CAP1 | 51.56 | -0.32 | -0.21 | -0.27 | 0.08 | -0.20 | -0.25 | -0.23 | 0.04 | -0.04 |
| --- | --- | --- | --- | --- | --- | --- | --- | --- | --- | --- | --- |
| P35564 | CANX | 67.28 | -0.08 | -0.12 | -0.10 | 0.03 | -0.12 | -0.10 | -0.11 | 0.02 | 0.01 |
| Q6ZQ38 | CAND1 | 136.33 | 0.09 | 0.09 | 0.09 | 0.00 | 0.18 | 0.10 | 0.14 | 0.06 | -0.05 |
| O35887 | CALU | 37.06 | -0.39 | -0.39 | -0.39 | 0.00 | -0.35 | -0.24 | -0.30 | 0.08 | -0.09 |
| P14211 | CALR | 47.99 | 0.07 | 0.10 | 0.08 | 0.02 | 0.08 | 0.07 | 0.07 | 0.01 | 0.01 |
|  |  | 16.84 | 0.23 | 0.01 | 0.12 | 0.15 | 0.19 | 0.11 | 0.15 | 0.05 | -0.03 |
| B2RQC6 | CAD | 243.24 | -0.03 | -0.83 | -0.43 | 0.57 | -0.39 | -0.05 | -0.22 | 0.24 | -0.21 |
| Q9CXW3 | CACYBP | 26.51 | -0.37 | -0.42 | -0.39 | 0.04 | -0.58 | -0.51 | -0.55 | 0.05 | 0.15 |
| Q9DB16 | CAB39L | 39.11 | -0.01 | -0.24 | -0.12 | 0.16 | -0.12 | -0.17 | -0.15 | 0.04 | 0.02 |
| Q06138 | CAB39 | 39.84 | 0.15 | -0.06 | 0.04 | 0.15 | 0.01 | 0.06 | 0.04 | 0.03 | 0.01 |
| P23589 | CA5A | 34.07 | 0.00 | 0.03 | 0.02 | 0.02 | 0.19 | 0.23 | 0.21 | 0.03 | -0.20 |
| P16015 | CA3 | 29.37 | 1.12 | 1.07 | 1.10 | 0.03 | 0.91 | 0.94 | 0.92 | 0.02 | 0.17 |
| P00920 | CA2 | 29.03 | -0.11 | -0.05 | -0.08 | 0.05 | -0.17 | -0.25 | -0.21 | 0.05 | 0.13 |

| P13634 | CA1 | 28.33 | 1.30 | 1.39 | 1.35 | 0.06 | 1.46 | 1.44 | 1.45 | 0.01 | -0.11 |
| --- | --- | --- | --- | --- | --- | --- | --- | --- | --- | --- | --- |
| P06684 | C5 | 188.88 | 1.29 | 1.04 | 1.17 | 0.17 | 0.91 | 1.00 | 0.95 | 0.07 | 0.21 |
| P01029 | C4B | 192.91 | -0.02 | 0.07 | 0.03 | 0.07 | -0.14 | -0.05 | -0.10 | 0.06 | 0.13 |
| P01027 | C3 | 186.48 | 0.09 | 0.09 | 0.09 | 0.00 | 0.01 | 0.03 | 0.02 | 0.02 | 0.07 |
| O35658 | C1QBP | 31.01 | 0.57 | 0.53 | 0.55 | 0.03 | 0.61 | 0.56 | 0.59 | 0.04 | -0.03 |
| Q91VK1 | BZW2 | 48.06 | -0.35 | 0.01 | -0.17 | 0.25 | -0.32 | -0.14 | -0.23 | 0.13 | 0.07 |
| Q9CQC6 | BZW1 | 48.04 | -0.22 | -0.09 | -0.15 | 0.09 | -0.21 | -0.04 | -0.13 | 0.12 | -0.03 |
| Q9CQH7 | BTF3L4 | 17.27 | 0.25 | 0.30 | 0.27 | 0.03 | 0.12 | 0.16 | 0.14 | 0.03 | 0.13 |
| Q64152 | BTF3 | 22.03 | -0.01 | 0.05 | 0.02 | 0.04 | -0.11 | -0.02 | -0.06 | 0.06 | 0.08 |
| Q8CIF4 | BTD | 58.15 | 0.64 | 0.58 | 0.61 | 0.04 | 0.52 | 0.58 | 0.55 | 0.04 | 0.05 |
| Q8R2Q8 | BST2 | 19.15 | -1.06 | -1.11 | -1.09 | 0.03 | -1.44 | -1.13 | -1.29 | 0.22 | 0.20 |
| P18572 | BSG | 42.44 | -0.35 | -0.40 | -0.38 | 0.04 | -0.35 | -0.39 | -0.37 | 0.03 | -0.01 |
| Q80Y55 | BSDC1 | 46.95 | 0.17 | 0.82 | 0.50 | 0.46 | 0.19 | 0.16 | 0.18 | 0.02 | 0.32 |

| Q8K2Q7 | BROX | 46.20 | -0.26 | -0.14 | -0.20 | 0.09 | -0.46 | -0.22 | -0.34 | 0.17 | 0.14 |
| --- | --- | --- | --- | --- | --- | --- | --- | --- | --- | --- | --- |
| Q9Z0S1 | BPNT1 | 33.20 | 0.30 | 0.26 | 0.28 | 0.03 | 0.02 | 0.17 | 0.09 | 0.11 | 0.18 |
| Q8R164 | BPHL | 32.85 | -0.02 | 0.09 | 0.04 | 0.08 | -0.06 | 0.10 | 0.02 | 0.11 | 0.02 |
| P15327 | BPGM | 29.98 | 0.27 | 0.43 | 0.35 | 0.11 | 0.26 | 0.21 | 0.24 | 0.04 | 0.11 |
| Q6QD59 | BNIP1 | 26.18 | -0.08 | -0.31 | -0.20 | 0.16 | -0.37 | -0.17 | -0.27 | 0.14 | 0.07 |
| Q9CY64 | BLVRA | 33.52 | -0.07 | -0.18 | -0.13 | 0.08 | -0.03 | -0.17 | -0.10 | 0.10 | -0.03 |
| Q8R016 | BLMH | 52.51 | -0.56 | -0.58 | -0.57 | 0.01 | -0.52 | -0.53 | -0.52 | 0.00 | -0.05 |
| O35490 | BHMT | 45.02 | 0.98 | 0.92 | 0.95 | 0.04 | 0.98 | 0.95 | 0.97 | 0.02 | -0.02 |
| P28653 | BGN | 41.64 | -0.17 | 0.62 | 0.22 | 0.56 | 0.38 | 0.76 | 0.57 | 0.27 | -0.35 |
| Q80XN0 | BDH1 | 38.30 | -0.97 | -0.98 | -0.98 | 0.01 | -1.05 | -1.08 | -1.07 | 0.02 | 0.09 |
| Q8K019 | BCLAF1 | 106.00 | -0.23 | -0.01 | -0.12 | 0.15 | 0.09 | 0.12 | 0.10 | 0.02 | -0.23 |
| P59017 | BCL2L13 | 46.72 | -0.17 | 0.13 | -0.02 | 0.21 | -0.13 | 0.01 | -0.06 | 0.10 | 0.04 |
| Q6P3A8 | BCKDHB | 42.88 | -0.07 | -0.10 | -0.08 | 0.02 | 0.37 | 0.33 | 0.35 | 0.03 | -0.44 |

| P50136 | BCKDHA | 50.37 | 0.12 | 0.03 | 0.07 | 0.06 | -0.14 | 0.45 | 0.16 | 0.42 | -0.09 |
| --- | --- | --- | --- | --- | --- | --- | --- | --- | --- | --- | --- |
| Q03311 | BCHE | 68.46 | -0.53 | -0.52 | -0.53 | 0.01 | -0.64 | -0.67 | -0.65 | 0.03 | 0.13 |
| Q9CWI3 | BCCIP | 35.94 | -0.45 | -0.26 | -0.35 | 0.13 | -0.55 | -0.22 | -0.38 | 0.23 | 0.03 |
| Q61335 | BCAP31 | 27.96 | 0.21 | 0.20 | 0.21 | 0.00 | 0.20 | 0.21 | 0.20 | 0.00 | 0.00 |
| Q61334 | BCAP29 | 27.96 | 0.59 | 0.54 | 0.56 | 0.04 | 0.36 | 0.52 | 0.44 | 0.12 | 0.12 |
| Q924Y0 | BBOX1 | 44.70 | -0.50 | -0.37 | -0.43 | 0.10 | -0.53 | -0.51 | -0.52 | 0.02 | 0.09 |
| O54962 | BANF1 | 10.10 | -0.25 | -0.32 | -0.28 | 0.05 | -0.23 | -0.35 | -0.29 | 0.09 | 0.01 |
| Q9Z1R2 | BAG6 | 121.04 | -0.60 | -0.28 | -0.44 | 0.23 | -0.46 | -0.13 | -0.29 | 0.24 | -0.15 |
| Q8CI32 | BAG5 | 50.94 | 0.02 | -0.19 | -0.08 | 0.15 | 0.06 | -0.11 | -0.02 | 0.12 | -0.06 |
| Q9JLV1 | BAG3 | 61.86 | -0.07 | 0.22 | 0.07 | 0.21 | -0.13 | 0.17 | 0.02 | 0.21 | 0.05 |
| Q60739 | BAG1 | 39.74 | -0.16 | -0.22 | -0.19 | 0.04 | -0.28 | -0.26 | -0.27 | 0.02 | 0.07 |
| Q91X34 | BAAT | 46.48 | 0.61 | 0.65 | 0.63 | 0.02 | 0.62 | 0.73 | 0.68 | 0.08 | -0.04 |
| P01887 | B2M | 13.78 | -0.15 | -0.16 | -0.16 | 0.00 | -0.34 | -0.24 | -0.29 | 0.07 | 0.13 |

| Q64726 | AZGP1 | 35.33 | 0.98 | 0.98 | 0.98 | 0.00 | 0.82 | 0.88 | 0.85 | 0.05 | 0.13 |
| --- | --- | --- | --- | --- | --- | --- | --- | --- | --- | --- | --- |
| P70295 | AUP1 | 46.12 | -0.23 | -0.06 | -0.14 | 0.12 | -0.10 | -0.07 | -0.08 | 0.02 | -0.06 |
| Q9JLZ3 | AUH | 33.40 | 0.46 | 0.46 | 0.46 | 0.00 | 0.38 | 0.41 | 0.40 | 0.03 | 0.07 |
| P28658 | ATXN10 | 53.71 | 0.44 | 0.51 | 0.48 | 0.05 | 0.28 | 0.21 | 0.24 | 0.05 | 0.23 |
| Q811I0 | ATPAF1 | 36.35 | 0.05 | -0.08 | -0.01 | 0.09 | -0.03 | -0.09 | -0.06 | 0.04 | 0.05 |
| Q8BVE3 | ATP6V1H | 55.85 | -0.21 | -0.21 | -0.21 | 0.01 | -0.13 | -0.16 | -0.14 | 0.02 | -0.07 |
| P50518 |  | 26.16 | -0.18 | -0.13 | -0.15 | 0.03 | -0.09 | -0.17 | -0.13 | 0.06 | -0.03 |
| P57746 | ATP6V1D | 28.37 | -0.18 | -0.20 | -0.19 | 0.01 | -0.02 | -0.22 | -0.12 | 0.14 | -0.07 |
| Q9Z1G3 | ATP6V1C1 | 43.89 | 0.00 | -0.09 | -0.04 | 0.07 | 0.03 | -0.11 | -0.04 | 0.10 | 0.00 |
| P62814 | ATP6V1B2 | 56.55 | -0.34 | -0.20 | -0.27 | 0.09 | -0.14 | -0.20 | -0.17 | 0.04 | -0.10 |
| P50516 | ATP6V1A | 68.33 | -0.16 | -0.26 | -0.21 | 0.07 | -0.10 | -0.24 | -0.17 | 0.10 | -0.04 |
| P51863 | ATP6V0D1 | 40.30 | 0.66 | 0.69 | 0.68 | 0.02 | 0.80 | 0.70 | 0.75 | 0.07 | -0.07 |
| P63082 | ATP6V0C | 15.81 | 0.26 | 0.39 | 0.32 | 0.09 | 0.48 | 0.52 | 0.50 | 0.03 | -0.18 |

| Q9DB20 | ATP5O | 23.36 | 0.02 | 0.09 | 0.05 | 0.05 | 0.07 | 0.12 | 0.10 | 0.04 | -0.05 |
| --- | --- | --- | --- | --- | --- | --- | --- | --- | --- | --- | --- |
| Q9CPQ8 | ATP5L | 11.42 | 0.01 | 0.12 | 0.07 | 0.08 | 0.05 | 0.07 | 0.06 | 0.01 | 0.00 |
| Q06185 | ATP5I | 8.24 | -0.02 | 0.12 | 0.05 | 0.10 | 0.12 | 0.21 | 0.16 | 0.06 | -0.11 |
| Q9DCX2 | ATP5H | 18.75 | 0.09 | 0.05 | 0.07 | 0.03 | 0.12 | 0.13 | 0.13 | 0.01 | -0.06 |
| Q9CQQ7 | ATP5F1 | 28.95 | 0.07 | 0.14 | 0.10 | 0.05 | 0.06 | 0.16 | 0.11 | 0.07 | 0.00 |
| Q91VR2 | ATP5C1 | 32.89 | 0.07 | 0.07 | 0.07 | 0.00 | 0.08 | 0.14 | 0.11 | 0.04 | -0.04 |
| P56480 | ATP5B | 56.30 | 0.07 | 0.08 | 0.08 | 0.00 | 0.09 | 0.14 | 0.12 | 0.03 | -0.04 |
| Q03265 | ATP5A1 | 59.75 | 0.01 | 0.07 | 0.04 | 0.04 | 0.10 | 0.10 | 0.10 | 0.00 | -0.07 |
| G5E829 | ATP2B1 | 134.75 | -0.14 | -0.17 | -0.16 | 0.02 | 0.00 | -0.14 | -0.07 | 0.10 | -0.09 |
| O55143 | ATP2A2 | 114.86 | -0.04 | -0.15 | -0.09 | 0.08 | -0.14 | -0.14 | -0.14 | 0.00 | 0.05 |
| Q8R429 | ATP2A1 | 109.42 | -4.62 | -4.97 | -4.79 | 0.25 | -4.74 | -4.87 | -4.81 | 0.09 | 0.01 |
| P97370 | ATP1B3 | 31.78 | 0.69 | 0.59 | 0.64 | 0.07 | 0.59 | 0.56 | 0.57 | 0.02 | 0.07 |
| P14094 | ATP1B1 | 35.19 | 0.22 | 0.08 | 0.15 | 0.10 | 0.27 | 0.21 | 0.24 | 0.04 | -0.09 |

| Q9WV27 | ATP1A4 | 114.89 | 0.30 | 0.19 | 0.25 | 0.07 | 0.36 | 0.48 | 0.42 | 0.09 | -0.18 |
| --- | --- | --- | --- | --- | --- | --- | --- | --- | --- | --- | --- |
| Q8VDN2 | ATP1A1 | 112.98 | 0.26 | 0.12 | 0.19 | 0.10 | 0.32 | 0.31 | 0.32 | 0.00 | -0.12 |
| Q9EPE9 | ATP13A1 | 132.39 | -0.02 | -0.03 | -0.03 | 0.01 | 0.14 | -0.03 | 0.06 | 0.13 | -0.08 |
| Q9QZW0 | ATP11C | 129.24 | 0.49 | 0.46 | 0.48 | 0.03 | 0.52 | 0.69 | 0.60 | 0.12 | -0.13 |
| O08997 | ATOX1 | 7.34 | -0.13 | -0.09 | -0.11 | 0.03 | -0.21 | -0.42 | -0.32 | 0.15 | 0.21 |
| Q91YH5 | ATL3 | 60.57 | 0.09 | 0.14 | 0.12 | 0.03 | -0.03 | 0.12 | 0.04 | 0.11 | 0.08 |
| Q6PA06 | ATL2 | 66.22 | -0.06 | 0.17 | 0.06 | 0.16 | 0.14 | 0.10 | 0.12 | 0.03 | -0.06 |
| Q9CWJ9 | ATIC | 64.22 | -0.18 | -0.20 | -0.19 | 0.01 | -0.15 | -0.14 | -0.15 | 0.01 | -0.04 |
| Q8BP56 | ATHL1 | 76.49 | -0.14 | 0.08 | -0.03 | 0.16 | -0.36 | 0.06 | -0.15 | 0.30 | 0.12 |
| Q9D906 | ATG7 | 77.52 | 0.13 | 0.01 | 0.07 | 0.08 | 0.08 | 0.04 | 0.06 | 0.03 | 0.01 |
| Q9CPX6 | ATG3 | 35.80 | -0.40 | -0.38 | -0.39 | 0.01 | -0.38 | -0.44 | -0.41 | 0.05 | 0.01 |
| Q925I1 | ATAD3 | 66.74 | 0.23 | 0.21 | 0.22 | 0.02 | 0.26 | 0.32 | 0.29 | 0.05 | -0.07 |
| Q9D5T0 | ATAD1 | 40.74 | 0.18 | 0.09 | 0.14 | 0.06 | 0.06 | 0.12 | 0.09 | 0.04 | 0.05 |

| P16460 | ASS1 | 46.58 | 0.24 | 0.26 | 0.25 | 0.01 | 0.36 | 0.40 | 0.38 | 0.03 | -0.13 |
| --- | --- | --- | --- | --- | --- | --- | --- | --- | --- | --- | --- |
| Q8BSY0 | ASPH | 83.04 | -0.19 | -0.06 | -0.13 | 0.09 | -0.24 | -0.12 | -0.18 | 0.08 | 0.06 |
| O54984 | ASNA1 | 38.82 | -0.07 | -0.19 | -0.13 | 0.08 | -0.15 | -0.12 | -0.13 | 0.02 | 0.01 |
| Q91YI0 | ASL | 51.74 | 0.27 | 0.30 | 0.28 | 0.02 | 0.34 | 0.37 | 0.36 | 0.02 | -0.07 |
| P24721 | ASGR2 | 34.91 | -0.18 | -0.28 | -0.23 | 0.07 | -0.19 | -0.22 | -0.21 | 0.02 | -0.02 |
| P34927 | ASGR1 | 32.59 | -0.32 | -0.18 | -0.25 | 0.10 | -0.12 | -0.17 | -0.14 | 0.03 | -0.11 |
| E9PZJ8 | ASCC3 | 250.55 | 0.45 | 0.11 | 0.28 | 0.24 | -0.07 | 0.01 | -0.03 | 0.05 | 0.31 |
| Q9WV54 | ASAH1 | 44.67 | 0.83 | 0.82 | 0.83 | 0.00 | 0.70 | 0.62 | 0.66 | 0.06 | 0.17 |
| Q91WU5 | AS3MT | 41.79 | -0.37 | -0.39 | -0.38 | 0.01 | -0.32 | -0.37 | -0.35 | 0.04 | -0.03 |
| Q9D898 | ARPC5L | 16.98 | 0.45 | 0.33 | 0.39 | 0.08 | 0.39 | 0.37 | 0.38 | 0.01 | 0.01 |
| Q9CPW4 | ARPC5 | 16.29 | -0.25 | -0.08 | -0.17 | 0.12 | -0.05 | -0.06 | -0.06 | 0.01 | -0.11 |
| Q9JM76 | ARPC3 | 20.52 | -0.10 | -0.01 | -0.06 | 0.07 | 0.02 | 0.06 | 0.04 | 0.03 | -0.09 |
| Q9CVB6 | ARPC2 | 34.36 | 0.00 | 0.02 | 0.01 | 0.02 | 0.09 | 0.06 | 0.07 | 0.03 | -0.06 |

| Q9WV32 | ARPC1B | 41.06 | 0.02 | -0.14 | -0.06 | 0.11 | 0.02 | -0.09 | -0.03 | 0.08 | -0.03 |
| --- | --- | --- | --- | --- | --- | --- | --- | --- | --- | --- | --- |
| A6H630 | ARMT1 | 50.55 | 0.06 | -0.15 | -0.04 | 0.15 | 0.03 | 0.01 | 0.02 | 0.02 | -0.07 |
| Q9WUL7 | ARL3 | 20.49 | 0.09 | 0.06 | 0.08 | 0.02 | 0.02 | 0.10 | 0.06 | 0.06 | 0.02 |
| P61211 | ARL1 | 20.41 | 0.35 | 0.15 | 0.25 | 0.14 | 0.19 | 0.26 | 0.23 | 0.04 | 0.03 |
| Q8R4H2 | ARHGEF12 | 172.35 | -0.15 | 0.25 | 0.05 | 0.28 | -0.07 | 0.20 | 0.06 | 0.19 | -0.02 |
| Q61599 | ARHGDIB | 22.85 | -0.19 | -0.17 | -0.18 | 0.01 | -0.10 | -0.33 | -0.22 | 0.17 | 0.04 |
| Q99PT1 | ARHGDIA | 23.41 | -0.32 | -0.18 | -0.25 | 0.10 | -0.13 | -0.14 | -0.13 | 0.01 | -0.12 |
| Q5FWK3 | ARHGAP1 | 50.41 | -0.55 | -0.31 | -0.43 | 0.17 | -0.14 | -0.25 | -0.20 | 0.08 | -0.23 |
| Q3UL36 | ARGLU1 | 32.89 | -0.18 | -0.04 | -0.11 | 0.11 | -0.14 | -0.28 | -0.21 | 0.09 | 0.10 |
| Q61176 | ARG1 | 34.81 | 0.40 | 0.30 | 0.35 | 0.08 | 0.43 | 0.40 | 0.42 | 0.02 | -0.07 |
| A2A5R2 | ARFGEF2 | 202.24 | -0.45 | -0.13 | -0.29 | 0.22 | -0.21 | -0.13 | -0.17 | 0.06 | -0.12 |
| Q99K28 | ARFGAP2 | 56.60 | -0.31 | -0.07 | -0.19 | 0.17 | -0.12 | 0.01 | -0.05 | 0.09 | -0.14 |
| Q9EPJ9 | ARFGAP1 | 45.29 | -0.56 | -0.31 | -0.44 | 0.18 | -0.32 | -0.12 | -0.22 | 0.14 | -0.22 |

| P84084 | ARF5 | 20.53 | 0.00 | -0.01 | -0.01 | 0.01 | -0.08 | -0.05 | -0.06 | 0.02 | 0.06 |
| --- | --- | --- | --- | --- | --- | --- | --- | --- | --- | --- | --- |
|  |  | 20.75 | -0.04 | 0.02 | -0.01 | 0.04 | -0.02 | 0.04 | 0.01 | 0.04 | -0.02 |
| Q5XJY5 | ARCN1 | 57.23 | -0.11 | 0.02 | -0.04 | 0.09 | 0.00 | 0.07 | 0.04 | 0.05 | -0.08 |
| Q9JJJ3 | AQP9 | 31.76 | 1.00 | 0.76 | 0.88 | 0.17 | 1.01 | 0.65 | 0.83 | 0.25 | 0.05 |
| Q8K3H0 | APPL1 | 79.33 | -0.39 | -0.53 | -0.46 | 0.10 | -0.50 | -0.28 | -0.39 | 0.16 | -0.07 |
| Q78IK4 | APOOL | 29.26 | -0.12 | -0.05 | -0.09 | 0.05 | -0.22 | -0.16 | -0.19 | 0.05 | 0.10 |
| P08226 | APOE | 35.87 | 0.10 | 0.11 | 0.10 | 0.01 | -0.22 | -0.17 | -0.19 | 0.04 | 0.29 |
| E9Q414 | APOB | 509.43 | 0.17 | 0.11 | 0.14 | 0.05 | -0.03 | -0.04 | -0.04 | 0.01 | 0.18 |
| Q8C7G5 | APOA5 | 41.26 | 0.68 | 0.55 | 0.61 | 0.09 | 0.21 | 0.48 | 0.35 | 0.19 | 0.27 |
| P06728 | APOA4 | 45.03 | -0.13 | -0.18 | -0.15 | 0.04 | -0.26 | -0.29 | -0.27 | 0.02 | 0.12 |
| Q8K4Z3 | APOA1BP | 30.97 | -0.08 | 0.08 | 0.00 | 0.11 | 0.08 | 0.04 | 0.06 | 0.03 | -0.06 |
| Q00623 | APOA1 | 30.62 | -0.02 | 0.07 | 0.03 | 0.07 | -0.18 | -0.15 | -0.17 | 0.02 | 0.19 |
| Q9D7N9 | APMAP | 46.43 | -0.06 | -0.03 | -0.05 | 0.02 | -0.08 | 0.00 | -0.04 | 0.05 | -0.01 |

| Q9WVQ5 | APIP | 26.95 | 0.12 | 0.14 | 0.13 | 0.01 | 0.30 | 0.10 | 0.20 | 0.14 | -0.07 |
| --- | --- | --- | --- | --- | --- | --- | --- | --- | --- | --- | --- |
| O35841 | API5 | 56.78 | -0.07 | -0.09 | -0.08 | 0.01 | 0.06 | -0.05 | 0.00 | 0.08 | -0.09 |
| P28352 | APEX1 | 35.49 | -0.03 | -0.02 | -0.02 | 0.01 | 0.01 | 0.00 | 0.00 | 0.01 | -0.03 |
| Q8R146 | APEH | 81.58 | -0.11 | -0.20 | -0.15 | 0.06 | -0.13 | -0.11 | -0.12 | 0.01 | -0.03 |
| Q9DCR2 | AP3S1 | 21.73 | -0.11 | -0.02 | -0.07 | 0.07 | 0.13 | 0.10 | 0.12 | 0.02 | -0.18 |
| O54774 | AP3D1 | 135.08 | -0.35 | -0.02 | -0.19 | 0.23 | -0.10 | 0.04 | -0.03 | 0.09 | -0.16 |
| Q9Z1T1 | AP3B1 | 122.74 | 0.13 | 0.03 | 0.08 | 0.07 | 0.30 | 0.04 | 0.17 | 0.18 | -0.09 |
| P84091 | AP2M1 | 49.65 | 0.20 | 0.12 | 0.16 | 0.06 | 0.22 | 0.21 | 0.22 | 0.00 | -0.06 |
| Q9DBG3 | AP2B1 | 104.58 | 0.11 | 0.18 | 0.15 | 0.04 | 0.19 | 0.15 | 0.17 | 0.03 | -0.02 |
| P17427 | AP2A2 | 104.02 | 0.13 | 0.17 | 0.15 | 0.03 | 0.11 | 0.13 | 0.12 | 0.02 | 0.03 |
| P17426 | AP2A1 | 107.66 | 0.05 | 0.10 | 0.07 | 0.04 | -0.07 | 0.14 | 0.03 | 0.15 | 0.04 |
| P35585 | AP1M1 | 48.54 | 0.02 | -0.18 | -0.08 | 0.14 | 0.01 | -0.10 | -0.04 | 0.08 | -0.04 |
| P22892 | AP1G1 | 91.35 | -0.09 | -0.12 | -0.11 | 0.02 | -0.18 | -0.10 | -0.14 | 0.06 | 0.03 |

| O35643 | AP1B1 | 103.93 | -0.14 | -0.16 | -0.15 | 0.01 | -0.03 | -0.04 | -0.04 | 0.01 | -0.12 |
| --- | --- | --- | --- | --- | --- | --- | --- | --- | --- | --- | --- |
| G3X982 | AOX3 | 146.90 | 2.07 | 2.60 | 2.34 | 0.37 | 2.31 | 2.67 | 2.49 | 0.26 | -0.16 |
| Q5SGK3 | AOX2 | 147.91 | 2.00 | 2.55 | 2.27 | 0.39 | 2.25 | 2.69 | 2.47 | 0.32 | -0.20 |
| O54754 | AOX1 | 146.68 | 1.45 | 1.86 | 1.66 | 0.29 | 1.26 | 1.84 | 1.55 | 0.41 | 0.11 |
| Q07076 | ANXA7 | 49.93 | 0.04 | 0.37 | 0.20 | 0.23 | -0.01 | 0.26 | 0.12 | 0.20 | 0.08 |
| P14824 | ANXA6 | 75.88 | -0.34 | -0.37 | -0.36 | 0.02 | -0.18 | -0.32 | -0.25 | 0.10 | -0.11 |
| P48036 | ANXA5 | 35.75 | -0.08 | -0.03 | -0.05 | 0.03 | -0.13 | -0.23 | -0.18 | 0.07 | 0.12 |
| P97429 | ANXA4 | 35.92 | -0.35 | -0.34 | -0.35 | 0.01 | -0.21 | -0.28 | -0.25 | 0.05 | -0.10 |
| O35639 | ANXA3 | 36.38 | -0.31 | -0.32 | -0.31 | 0.00 | -0.25 | -0.34 | -0.29 | 0.07 | -0.02 |
| P07356 | ANXA2 | 38.68 | -0.12 | -0.13 | -0.13 | 0.00 | 0.02 | -0.16 | -0.07 | 0.13 | -0.06 |
| P97384 | ANXA11 | 54.08 | -0.24 | -0.05 | -0.14 | 0.13 | -0.10 | -0.03 | -0.07 | 0.05 | -0.08 |
| P10107 | ANXA1 | 38.73 | -0.06 | 0.04 | -0.01 | 0.07 | 0.10 | 0.02 | 0.06 | 0.05 | -0.07 |
| P97449 | ANPEP | 109.65 | -0.22 | 0.23 | 0.00 | 0.32 | -0.51 | 0.09 | -0.21 | 0.42 | 0.21 |

| P97822 | ANP32E | 29.62 | -0.12 | -0.12 | -0.12 | 0.00 | 0.04 | -0.15 | -0.06 | 0.13 | -0.06 |
| --- | --- | --- | --- | --- | --- | --- | --- | --- | --- | --- | --- |
| O35381 | ANP32A | 28.54 | -0.01 | -0.15 | -0.08 | 0.11 | 0.04 | -0.03 | 0.01 | 0.05 | -0.09 |
| Q810B6 | ANKFY1 | 128.65 | 0.02 | -0.08 | -0.03 | 0.07 | 0.12 | -0.01 | 0.06 | 0.09 | -0.08 |
| Q02357 | ANK1 | 204.22 | 0.23 | 0.29 | 0.26 | 0.04 | 0.08 | 0.20 | 0.14 | 0.08 | 0.12 |
| Q8CFA2 | AMT | 44.01 | -0.42 | -0.38 | -0.40 | 0.03 | -0.24 | -0.16 | -0.20 | 0.06 | -0.20 |
| Q9DBT5 | AMPD2 | 92.02 | -0.83 | -0.68 | -0.76 | 0.11 | -0.79 | -0.65 | -0.72 | 0.09 | -0.04 |
| Q9DBA8 | AMDHD1 | 46.49 | -0.40 | -0.40 | -0.40 | 0.00 | -0.29 | -0.27 | -0.28 | 0.02 | -0.12 |
| O09174 | AMACR | 41.70 | 0.04 | -0.01 | 0.01 | 0.04 | -0.03 | 0.05 | 0.01 | 0.05 | 0.01 |
| O08583 | ALYREF | 26.94 | -0.18 | -0.14 | -0.16 | 0.02 | 0.07 | 0.03 | 0.05 | 0.03 | -0.21 |
| Q9DB25 | ALG5 | 36.79 | 0.20 | 0.06 | 0.13 | 0.10 | 0.03 | 0.09 | 0.06 | 0.05 | 0.07 |
| P05063 | ALDOC | 39.39 | 0.15 | 0.09 | 0.12 | 0.04 | 0.27 | 0.25 | 0.26 | 0.01 | -0.14 |
| Q91Y97 | ALDOB | 39.51 | -0.37 | -0.57 | -0.47 | 0.15 | -0.52 | -0.69 | -0.60 | 0.12 | 0.14 |
| P05064 | ALDOA | 39.36 | -0.54 | -0.52 | -0.53 | 0.02 | -0.42 | -0.48 | -0.45 | 0.04 | -0.08 |

| Q9JLJ2 | ALDH9A1 | 53.51 | -0.07 | -0.06 | -0.06 | 0.01 | -0.14 | -0.11 | -0.13 | 0.02 | 0.06 |
| --- | --- | --- | --- | --- | --- | --- | --- | --- | --- | --- | --- |
| Q8BH00 | ALDH8A1 | 53.66 | -0.16 | -0.11 | -0.14 | 0.03 | -0.18 | -0.12 | -0.15 | 0.04 | 0.01 |
| Q9DBF1 | ALDH7A1 | 58.86 | 1.00 | 1.21 | 1.11 | 0.15 | 1.04 | 1.18 | 1.11 | 0.10 | -0.01 |
| Q9EQ20 | ALDH6A1 | 57.92 | 0.40 | 0.41 | 0.41 | 0.01 | 0.36 | 0.43 | 0.39 | 0.05 | 0.02 |
| Q8BWF0 | ALDH5A1 | 55.97 | 0.29 | 0.31 | 0.30 | 0.02 | 0.17 | 0.23 | 0.20 | 0.05 | 0.10 |
| Q8CHT0 | ALDH4A1 | 61.84 | 0.03 | -0.05 | -0.01 | 0.05 | -0.02 | 0.01 | -0.01 | 0.02 | -0.01 |
| P47740 | ALDH3A2 | 53.97 | 0.20 | 0.34 | 0.27 | 0.10 | -0.26 | -0.20 | -0.23 | 0.04 | 0.50 |
| P47738 | ALDH2 | 56.54 | 0.35 | 0.29 | 0.32 | 0.04 | 0.26 | 0.17 | 0.22 | 0.06 | 0.10 |
| Q8R0Y6 | ALDH1L1 | 98.71 | -0.14 | -0.11 | -0.12 | 0.03 | -0.07 | 0.02 | -0.03 | 0.06 | -0.10 |
| Q9CZS1 | ALDH1B1 | 57.55 | -0.22 | -0.14 | -0.18 | 0.05 | 0.23 | 0.32 | 0.28 | 0.06 | -0.46 |
| O35945 | ALDH1A7 | 54.59 | 1.06 | 1.09 | 1.08 | 0.02 | 0.95 | 0.99 | 0.97 | 0.03 | 0.10 |
| P24549 | ALDH1A1 | 54.47 | 1.24 | 1.37 | 1.30 | 0.09 | 1.10 | 1.25 | 1.18 | 0.10 | 0.12 |
| Q61490 | ALCAM | 65.09 | -0.15 | -0.12 | -0.14 | 0.02 | 0.01 | -0.13 | -0.06 | 0.10 | -0.07 |

| P07724 | ALB | 68.69 | -0.08 | -0.12 | -0.10 | 0.03 | -0.18 | -0.28 | -0.23 | 0.07 | 0.13 |
| --- | --- | --- | --- | --- | --- | --- | --- | --- | --- | --- | --- |
| P10518 | ALAD | 36.02 | -0.19 | -0.28 | -0.24 | 0.06 | -0.24 | 0.00 | -0.12 | 0.17 | -0.12 |
| Q60823 | AKT2 | 55.74 | -0.15 | -0.23 | -0.19 | 0.06 | -0.32 | -0.14 | -0.23 | 0.13 | 0.04 |
| Q8CG76 | AKR7A2 | 40.61 | 0.58 | 0.58 | 0.58 | 0.00 | 0.77 | 0.52 | 0.65 | 0.17 | -0.07 |
| Q9DCT1 | AKR1E2 | 34.46 | -0.20 | -0.13 | -0.16 | 0.05 | -0.26 | -0.39 | -0.32 | 0.09 | 0.16 |
| Q8VCX1 | AKR1D1 | 37.29 | -0.56 | -0.69 | -0.63 | 0.09 | -0.49 | -0.54 | -0.52 | 0.03 | -0.11 |
| P70694 | AKR1C6 | 37.05 | 0.05 | -0.05 | 0.00 | 0.07 | 0.00 | -0.07 | -0.03 | 0.05 | 0.03 |
| Q8K023 | AKR1C18 | 37.18 | 0.29 | 0.15 | 0.22 | 0.10 | 0.11 | -2.31 | -1.10 | 1.71 | 1.32 |
| Q8VC28 | AKR1C13 | 37.06 | 0.48 | 0.38 | 0.43 | 0.08 | 0.27 | 0.23 | 0.25 | 0.03 | 0.18 |
| P45377 | AKR1B8 | 36.12 | 0.01 | -0.04 | -0.01 | 0.04 | -0.18 | 0.03 | -0.07 | 0.15 | 0.06 |
| P21300 | AKR1B7 | 35.99 | -0.38 | -3.67 | -2.02 | 2.33 | -0.80 | -0.27 | -0.54 | 0.37 | -1.49 |
| P45376 | AKR1B1 | 35.73 | -0.29 | -0.57 | -0.43 | 0.19 | -0.17 | -0.37 | -0.27 | 0.14 | -0.16 |
| Q9JII6 | AKR1A1 | 36.59 | 0.79 | 0.71 | 0.75 | 0.06 | 0.56 | 0.58 | 0.57 | 0.01 | 0.18 |

| Q9DBR0 | AKAP8 | 76.29 | -0.04 | 0.21 | 0.08 | 0.18 | 0.06 | 0.18 | 0.12 | 0.08 | -0.04 |
| --- | --- | --- | --- | --- | --- | --- | --- | --- | --- | --- | --- |
| Q9WTQ5 | AKAP12 | 180.69 | -1.33 | -0.83 | -1.08 | 0.36 | -0.94 | -0.66 | -0.80 | 0.20 | -0.28 |
| O08715 | AKAP1 | 92.19 | -0.45 | -0.01 | -0.23 | 0.31 | -0.32 | -0.06 | -0.19 | 0.18 | -0.03 |
| Q9WUR9 | AK4 | 25.06 | -0.07 | -0.02 | -0.04 | 0.03 | -0.15 | -0.18 | -0.16 | 0.02 | 0.12 |
| Q9WTP7 | AK3 | 25.43 | 0.28 | 0.33 | 0.31 | 0.03 | 0.29 | 0.37 | 0.33 | 0.06 | -0.02 |
| Q9WTP6 | AK2 | 26.47 | 0.03 | 0.04 | 0.04 | 0.01 | -0.14 | -0.03 | -0.08 | 0.08 | 0.12 |
| P31230 | AIMP1 | 34.00 | 0.00 | 0.08 | 0.04 | 0.05 | -0.07 | 0.00 | -0.03 | 0.05 | 0.07 |
| Q8BUE4 | AIFM2 | 40.63 | 0.53 | 0.20 | 0.36 | 0.23 | -0.02 | 0.05 | 0.01 | 0.05 | 0.35 |
| Q9Z0X1 | AIFM1 | 66.77 | 0.01 | -0.02 | -0.01 | 0.02 | 0.06 | -0.04 | 0.01 | 0.08 | -0.02 |
| Q8BK64 | AHSA1 | 38.12 | -0.22 | -0.29 | -0.25 | 0.05 | -0.37 | -0.36 | -0.36 | 0.01 | 0.11 |
| Q80SW1 | AHCYL1 | 58.95 | -0.06 | 0.04 | -0.01 | 0.07 | 0.04 | 0.07 | 0.05 | 0.02 | -0.06 |
| P50247 | AHCY | 47.69 | 0.16 | 0.22 | 0.19 | 0.04 | 0.23 | 0.27 | 0.25 | 0.03 | -0.06 |
| Q3UEG6 | AGXT2 | 57.11 | 0.50 | 0.47 | 0.48 | 0.02 | 0.58 | 0.66 | 0.62 | 0.06 | -0.14 |

| O35423 | AGXT | 45.91 | -0.04 | -0.23 | -0.13 | 0.14 | 0.08 | 0.09 | 0.09 | 0.01 | -0.22 |
| --- | --- | --- | --- | --- | --- | --- | --- | --- | --- | --- | --- |
| Q8K2C8 | AGPAT6 | 52.18 | 0.80 | 1.02 | 0.91 | 0.16 | 0.88 | 0.91 | 0.90 | 0.02 | 0.02 |
| Q9D517 | AGPAT3 | 43.30 | -0.29 | -0.31 | -0.30 | 0.02 | -0.26 | -0.26 | -0.26 | 0.01 | -0.04 |
| Q8K3K7 | AGPAT2 | 31.01 | 0.01 | 0.14 | 0.07 | 0.10 | -0.20 | -0.04 | -0.12 | 0.11 | 0.20 |
| Q9ESW4 | AGK | 46.98 | 0.03 | 0.05 | 0.04 | 0.01 | 0.05 | 0.05 | 0.05 | 0.00 | -0.01 |
| Q8K2K6 | AGFG1 | 58.04 | -0.53 | -0.22 | -0.38 | 0.22 | -0.36 | -0.05 | -0.20 | 0.22 | -0.17 |
| Q64191 | AGA | 37.02 | 0.14 | 0.33 | 0.24 | 0.13 | 0.26 | 0.01 | 0.13 | 0.18 | 0.10 |
| Q8K4H1 | AFMID | 34.23 | 0.04 | 0.09 | 0.06 | 0.03 | 0.66 | 0.73 | 0.70 | 0.05 | -0.63 |
| O89020 | AFM | 69.38 | -0.22 | -0.27 | -0.25 | 0.04 | -0.51 | -0.48 | -0.50 | 0.02 | 0.25 |
| Q8JZQ2 | AFG3L2 | 89.52 | 0.10 | 0.02 | 0.06 | 0.06 | 0.07 | 0.10 | 0.08 | 0.02 | -0.02 |
| Q920A7 | AFG3L1 | 87.05 | 0.14 | 0.26 | 0.20 | 0.09 | 0.46 | 0.15 | 0.30 | 0.22 | -0.10 |
| P28650 | ADSSL1 | 50.25 | -0.03 | 0.03 | 0.00 | 0.04 | 0.74 | 0.13 | 0.44 | 0.43 | -0.44 |
| P46664 | ADSS | 50.02 | 0.12 | -0.11 | 0.01 | 0.16 | 0.01 | -0.12 | -0.05 | 0.09 | 0.06 |

| P54822 | ADSL | 54.87 | -0.51 | -0.43 | -0.47 | 0.06 | -0.32 | -0.43 | -0.38 | 0.07 | -0.09 |
| --- | --- | --- | --- | --- | --- | --- | --- | --- | --- | --- | --- |
| Q9JKV1 | ADRM1 | 42.06 | -0.47 | -0.21 | -0.34 | 0.18 | -0.40 | -0.25 | -0.33 | 0.11 | -0.02 |
| P55264 | ADK | 40.15 | 0.01 | -0.05 | -0.02 | 0.04 | -0.02 | -0.02 | -0.02 | 0.00 | 0.00 |
| Q8R0N6 | ADHFE1 | 49.94 | 0.25 | 0.32 | 0.29 | 0.05 | 0.07 | 0.20 | 0.14 | 0.09 | 0.15 |
| P28474 | ADH5 | 39.55 | 0.17 | 0.02 | 0.10 | 0.11 | 0.16 | 0.05 | 0.11 | 0.07 | -0.01 |
| P00329 | ADH1 | 39.77 | -0.59 | -0.67 | -0.63 | 0.06 | -0.71 | -0.78 | -0.75 | 0.05 | 0.12 |
| Q9QYC0 | ADD1 | 80.65 | -0.44 | -0.25 | -0.34 | 0.14 | 0.07 | -0.21 | -0.07 | 0.20 | -0.27 |
| Q60936 | ADCK3 | 71.74 | -0.76 | -0.62 | -0.69 | 0.10 | -0.71 | -0.47 | -0.59 | 0.17 | -0.10 |
| Q91XE4 | ACY3 | 35.29 | -0.14 | -0.11 | -0.12 | 0.02 | -0.25 | -0.12 | -0.19 | 0.09 | 0.06 |
| Q99JW2 | ACY1 | 45.78 | -0.48 | -0.40 | -0.44 | 0.06 | -0.20 | -0.21 | -0.20 | 0.00 | -0.23 |
| Q99JY9 | ACTR3 | 47.36 | -0.05 | -0.08 | -0.06 | 0.01 | -0.07 | 0.06 | -0.01 | 0.09 | -0.06 |
| P61161 | ACTR2 | 44.76 | 0.04 | 0.04 | 0.04 | 0.00 | 0.02 | 0.09 | 0.05 | 0.05 | -0.01 |
| Q8R5C5 | ACTR1B | 42.28 | -0.10 | -0.16 | -0.13 | 0.04 | -0.17 | -0.11 | -0.14 | 0.05 | 0.01 |

| P61164 | ACTR1A | 42.61 | -0.15 | -0.23 | -0.19 | 0.06 | -0.11 | -0.15 | -0.13 | 0.03 | -0.06 |
| --- | --- | --- | --- | --- | --- | --- | --- | --- | --- | --- | --- |
| P57780 | ACTN4 | 104.98 | 0.20 | 0.15 | 0.17 | 0.03 | 0.21 | 0.19 | 0.20 | 0.01 | -0.02 |
| Q7TPR4 | ACTN1 | 103.07 | -0.19 | -0.20 | -0.19 | 0.01 | -0.13 | -0.18 | -0.16 | 0.03 | -0.03 |
| P63260 |  | 41.79 | 0.02 | -0.04 | -0.01 | 0.04 | 0.12 | 0.00 | 0.06 | 0.08 | -0.07 |
| P68134 | ACTA1 | 42.05 | -5.34 | -3.02 | -4.18 | 1.64 | 0.11 | 0.01 | 0.06 | 0.07 | -4.24 |
| Q14DH7 | ACSS3 | 74.52 | -0.98 | -0.88 | -0.93 | 0.07 | -1.51 | -1.51 | -1.51 | 0.00 | 0.58 |
| Q9QXG4 | ACSS2 | 78.86 | -0.76 | -0.83 | -0.79 | 0.05 | -0.85 | -0.70 | -0.77 | 0.10 | -0.02 |
| Q8BGA8 | ACSM5 | 64.33 | 0.97 | 1.03 | 1.00 | 0.04 | 0.98 | 1.06 | 1.02 | 0.06 | -0.01 |
| Q3UNX5 | ACSM3 | 65.62 | 0.27 | 0.26 | 0.27 | 0.01 | 0.04 | 0.13 | 0.08 | 0.07 | 0.18 |
| Q91VA0 | ACSM1 | 64.76 | 0.64 | 0.61 | 0.63 | 0.03 | 0.70 | 0.72 | 0.71 | 0.01 | -0.08 |
| Q8JZR0 | ACSL5 | 76.21 | -0.76 | -0.79 | -0.77 | 0.02 | -0.79 | -0.88 | -0.84 | 0.07 | 0.06 |
| Q9QUJ7 | ACSL4 | 79.08 | -0.20 | -0.28 | -0.24 | 0.05 | 0.23 | 0.16 | 0.19 | 0.05 | -0.43 |
| Q9CZW4 | ACSL3 | 80.49 | -0.56 | -0.28 | -0.42 | 0.20 | -0.69 | -0.77 | -0.73 | 0.06 | 0.31 |

| P41216 | ACSL1 | 77.95 | 1.04 | 0.96 | 1.00 | 0.05 | 0.79 | 0.78 | 0.78 | 0.01 | 0.22 |
| --- | --- | --- | --- | --- | --- | --- | --- | --- | --- | --- | --- |
| Q3URE1 | ACSF3 | 65.08 | 0.13 | 0.01 | 0.07 | 0.08 | -0.03 | 0.06 | 0.01 | 0.06 | 0.06 |
| Q8VCW8 | ACSF2 | 67.95 | 0.83 | 0.78 | 0.80 | 0.03 | 0.68 | 0.77 | 0.72 | 0.06 | 0.08 |
| Q8BP40 | ACP6 | 47.62 | 0.23 | 0.38 | 0.31 | 0.10 | 0.40 | 0.46 | 0.43 | 0.04 | -0.13 |
| Q9QXD1 | ACOX2 | 76.86 | 0.61 | 0.57 | 0.59 | 0.03 | 0.87 | 0.96 | 0.91 | 0.07 | -0.32 |
| Q9R0H0 | ACOX1 | 74.65 | 0.62 | 0.68 | 0.65 | 0.04 | 0.26 | 0.32 | 0.29 | 0.04 | 0.36 |
| P58137 | ACOT8 | 35.83 | 0.35 | 0.38 | 0.36 | 0.02 | 0.16 | 0.28 | 0.22 | 0.08 | 0.14 |
| Q9QYR7 | ACOT3 | 47.49 | -1.35 | -1.29 | -1.32 | 0.04 | -1.85 | -1.91 | -1.88 | 0.04 | 0.56 |
| Q9QYR9 | ACOT2 | 49.66 | -0.10 | -0.14 | -0.12 | 0.03 | -0.72 | -0.72 | -0.72 | 0.00 | 0.60 |
| Q9CQR4 | ACOT13 | 15.18 | -0.62 | -0.51 | -0.56 | 0.08 | -0.58 | -0.54 | -0.56 | 0.03 | 0.00 |
| Q9DBK0 | ACOT12 | 61.76 | -0.05 | 0.03 | -0.01 | 0.06 | -0.15 | -0.10 | -0.13 | 0.03 | 0.12 |
| O55137 | ACOT1 | 46.14 | 1.09 | 0.65 | 0.87 | 0.32 | 0.41 | 0.04 | 0.23 | 0.26 | 0.64 |
| Q99KI0 | ACO2 | 85.46 | -0.03 | -0.14 | -0.09 | 0.07 | -0.06 | -0.14 | -0.10 | 0.05 | 0.01 |

| P28271 | ACO1 | 98.12 | 0.11 | -0.01 | 0.05 | 0.09 | 0.02 | 0.01 | 0.02 | 0.00 | 0.03 |
| --- | --- | --- | --- | --- | --- | --- | --- | --- | --- | --- | --- |
| A2AKK5 | ACNAT1 | 46.07 | -0.51 | -0.53 | -0.52 | 0.01 | -0.39 | -0.27 | -0.33 | 0.09 | -0.19 |
| Q91V92 | ACLY | 119.73 | -1.82 | -1.71 | -1.76 | 0.08 | -1.89 | -1.73 | -1.81 | 0.11 | 0.05 |
| Q9JIX8 | ACIN1 | 150.72 | -0.35 | 0.03 | -0.16 | 0.27 | -0.42 | 0.17 | -0.12 | 0.42 | -0.03 |
| Q5XG73 | ACBD5 | 56.61 | 0.01 | 0.32 | 0.16 | 0.22 | 0.07 | 0.24 | 0.15 | 0.12 | 0.01 |
| Q8BMP6 | ACBD3 | 60.18 | -0.12 | 0.02 | -0.05 | 0.10 | 0.09 | 0.08 | 0.09 | 0.01 | -0.14 |
| Q8CAY6 | ACAT2 | 41.30 | 0.02 | -0.08 | -0.03 | 0.07 | -0.16 | -0.06 | -0.11 | 0.07 | 0.08 |
| Q8QZT1 | ACAT1 | 44.82 | -0.41 | -0.43 | -0.42 | 0.01 | -0.60 | -0.61 | -0.60 | 0.01 | 0.18 |
| P50544 | ACADVL | 70.88 | -0.16 | -0.07 | -0.11 | 0.07 | -0.13 | -0.13 | -0.13 | 0.00 | 0.02 |
| Q9DBL1 | ACADSB | 47.87 | 0.90 | 0.93 | 0.92 | 0.01 | 0.87 | 0.90 | 0.89 | 0.03 | 0.03 |
| Q07417 | ACADS | 44.89 | 0.39 | 0.25 | 0.32 | 0.10 | 0.08 | 0.14 | 0.11 | 0.05 | 0.21 |
| P45952 | ACADM | 46.48 | 0.31 | 0.31 | 0.31 | 0.00 | 0.21 | 0.35 | 0.28 | 0.10 | 0.03 |
| P51174 | ACADL | 47.91 | -0.37 | -0.35 | -0.36 | 0.01 | -0.50 | -0.45 | -0.47 | 0.04 | 0.11 |

| Q8JZN5 | ACAD9 | 68.72 | 0.18 | 0.17 | 0.17 | 0.00 | 0.06 | 0.14 | 0.10 | 0.06 | 0.08 |
| --- | --- | --- | --- | --- | --- | --- | --- | --- | --- | --- | --- |
| Q80XL6 | ACAD11 | 87.37 | 0.37 | 0.27 | 0.32 | 0.07 | 0.12 | 0.08 | 0.10 | 0.04 | 0.22 |
| Q8K370 | ACAD10 | 118.98 | -0.13 | -0.12 | -0.12 | 0.01 | 0.07 | 0.15 | 0.11 | 0.05 | -0.23 |
| E9Q4Z2 | ACACB | 275.75 | -0.92 | -1.08 | -1.00 | 0.12 | -0.95 | -1.05 | -1.00 | 0.06 | 0.00 |
| Q5SWU9 | ACACA | 265.25 | -1.35 | -1.51 | -1.43 | 0.12 | -1.52 | -1.59 | -1.55 | 0.05 | 0.12 |
| Q8BWT1 | ACAA2 | 41.83 | 0.26 | 0.28 | 0.27 | 0.01 | 0.16 | 0.20 | 0.18 | 0.03 | 0.08 |
| Q8VCH0 | ACAA1B | 44.00 | 0.61 | 0.48 | 0.55 | 0.10 | 0.11 | 0.04 | 0.07 | 0.05 | 0.47 |
| Q69ZX8 | ABLIM3 | 77.63 | 0.62 | 1.05 | 0.84 | 0.30 | 0.92 | 0.46 | 0.69 | 0.33 | 0.15 |
| Q8K4G5 | ABLIM1 | 96.80 | -0.16 | -0.09 | -0.12 | 0.05 | 0.06 | 0.11 | 0.09 | 0.03 | -0.21 |
| Q8R2Y0 | ABHD6 | 38.20 | 0.58 | 0.36 | 0.47 | 0.15 | -0.01 | -0.01 | -0.01 | 0.00 | 0.48 |
| Q8VCR7 | ABHD14B | 22.45 | 0.04 | 0.14 | 0.09 | 0.07 | 0.07 | 0.13 | 0.10 | 0.04 | -0.01 |
| Q99LR1 | ABHD12 | 45.27 | 0.08 | -0.02 | 0.03 | 0.07 | 0.04 | -0.03 | 0.01 | 0.05 | 0.02 |
| Q7TMS5 | ABCG2 | 72.98 | 1.20 | 1.29 | 1.24 | 0.07 | 0.99 | 0.98 | 0.99 | 0.01 | 0.26 |

| Q8K268 | ABCF3 | 79.86 | 0.18 | -0.13 | 0.03 | 0.22 | -0.25 | 0.04 | -0.10 | 0.21 | 0.13 |
| --- | --- | --- | --- | --- | --- | --- | --- | --- | --- | --- | --- |
| Q99LE6 | ABCF2 | 71.78 | 0.13 | 0.10 | 0.11 | 0.02 | 0.07 | 0.14 | 0.11 | 0.05 | 0.00 |
| Q6P542 | ABCF1 | 94.94 | -0.07 | -0.07 | -0.07 | 0.01 | -0.08 | -0.09 | -0.08 | 0.01 | 0.01 |
| P61222 | ABCE1 | 67.31 | 0.17 | 0.10 | 0.14 | 0.05 | 0.16 | 0.09 | 0.13 | 0.05 | 0.01 |
| P55096 | ABCD3 | 75.47 | 0.17 | 0.11 | 0.14 | 0.04 | -0.06 | -0.21 | -0.13 | 0.11 | 0.27 |
| Q61285 | ABCD2 | 83.48 | -3.17 | -3.09 | -3.13 | 0.06 | -2.84 | -3.47 | -3.15 | 0.45 | 0.02 |
| Q9R1S7 | ABCC6 | 164.86 | -0.14 | -0.23 | -0.19 | 0.06 | -0.38 | -0.23 | -0.30 | 0.11 | 0.11 |
| B2RX12 | ABCC3 | 169.12 | 1.70 | 1.56 | 1.63 | 0.10 | 1.48 | 1.51 | 1.50 | 0.02 | 0.13 |
| Q8VI47 | ABCC2 | 173.67 | 0.55 | 0.42 | 0.48 | 0.09 | 0.51 | 0.56 | 0.54 | 0.04 | -0.05 |
| Q9CXJ4 | ABCB8 | 78.00 | 0.04 | -0.16 | -0.06 | 0.14 | -0.16 | -0.06 | -0.11 | 0.07 | 0.05 |
| Q61102 | ABCB7 | 82.58 | 0.02 | -0.06 | -0.02 | 0.06 | 0.16 | 0.11 | 0.13 | 0.03 | -0.15 |
| P21440 | ABCB4 | 140.38 | 0.32 | 0.46 | 0.39 | 0.10 | 0.23 | 0.28 | 0.26 | 0.04 | 0.13 |
| Q9QY30 | ABCB11 | 146.75 | 0.04 | 0.15 | 0.10 | 0.08 | 0.23 | 0.29 | 0.26 | 0.05 | -0.16 |

| Q8K440 | ABCA8B | 183.04 | -0.35 | -0.67 | -0.51 | 0.23 | -0.23 | -0.31 | -0.27 | 0.05 | -0.24 |
| --- | --- | --- | --- | --- | --- | --- | --- | --- | --- | --- | --- |
| Q8K441 | ABCA6 | 183.28 | 0.41 | 0.30 | 0.36 | 0.08 | 0.46 | 0.40 | 0.43 | 0.05 | -0.07 |
| P61922 | ABAT | 56.45 | 0.76 | 0.68 | 0.72 | 0.05 | 0.74 | 0.71 | 0.73 | 0.02 | -0.01 |
| Q99K67 | AASS | 102.97 | 0.74 | 0.93 | 0.84 | 0.13 | 0.84 | 1.02 | 0.93 | 0.13 | -0.09 |
| Q3THG9 | AARSD1 | 44.97 | -0.06 | -0.25 | -0.15 | 0.14 | -0.24 | -0.30 | -0.27 | 0.04 | 0.12 |
| Q8BGQ7 | AARS | 106.91 | 0.21 | 0.11 | 0.16 | 0.07 | 0.21 | 0.12 | 0.17 | 0.07 | 0.00 |
| Q9WVM8 | AADAT | 47.60 | 0.85 | 0.94 | 0.90 | 0.07 | 0.95 | 1.10 | 1.03 | 0.10 | -0.13 |
| Q99PG0 | AADAC | 45.25 | -0.08 | -0.07 | -0.07 | 0.01 | -0.26 | -0.21 | -0.23 | 0.04 | 0.16 |
| Q9D2R0 | AACS | 75.20 | -0.86 | -0.93 | -0.90 | 0.05 | -0.72 | -0.76 | -0.74 | 0.02 | -0.16 |
| Q61838 | A2M | 165.85 | 0.48 | 0.71 | 0.59 | 0.17 | 0.16 | 0.31 | 0.24 | 0.11 | 0.36 |
| Q5YD48 | A1CF | 65.68 | 0.11 | 0.31 | 0.21 | 0.14 | 0.11 | 0.26 | 0.19 | 0.10 | 0.02 |
| Q8C1B7 | 42258 | 49.69 | -0.26 | -0.11 | -0.19 | 0.10 | 0.01 | -0.14 | -0.06 | 0.11 | -0.12 |
| Q80UG5 | 42256 | 65.57 | -0.35 | -0.30 | -0.32 | 0.04 | -0.50 | -0.40 | -0.45 | 0.07 | 0.12 |

| Q8CHH9 | 42255 | 49.81 | -0.07 | -0.08 | -0.08 | 0.01 | 0.08 | -0.11 | -0.02 | 0.14 | -0.06 |
| --- | --- | --- | --- | --- | --- | --- | --- | --- | --- | --- | --- |
| O55131 | 42254 | 50.55 | -0.04 | -0.10 | -0.07 | 0.05 | -0.01 | -0.10 | -0.05 | 0.06 | -0.02 |
| P28661 | 42251 | 54.94 | -0.22 | -0.10 | -0.16 | 0.09 | 0.10 | 0.01 | 0.06 | 0.07 | -0.22 |
| P42208 | 42249 | 41.53 | -0.11 | -0.11 | -0.11 | 0.00 | -0.12 | -0.15 | -0.14 | 0.02 | 0.02 |
| Q922Q1 | 42065 | 38.19 | 0.05 | 0.02 | 0.03 | 0.02 | 0.10 | 0.08 | 0.09 | 0.01 | -0.06 |
| P01864 | 0 | 36.60 | 4.07 | 4.09 | 4.08 | 0.01 | 3.63 | 3.22 | 3.42 | 0.29 | 0.66 |
