# Supplementary material for: Hepatitis B virus X induces inflammation and cancer in mice liver through dysregulation of cytoskeletal remodeling and lipid metabolism
Source: Oncotarget. 2016 Sep 30;7(43):70559–74. doi: 10.18632/oncotarget.12372 (PMC5342574; doi:10.18632/oncotarget.12372)
Supplement: Supplementary file 3 [file oncotarget-07-70559-s003.docx]

**supplemental Tables 2 The quantitative data of proteomics for 24M p21HBx/+ or 24 WT vs SILAM mice**

| **Protein IDs** | **Gene name** | **Mol.**  **weight**  **[kDa]** | **24M**  **Log2**  **HBX /SILAM**  **GEL** | **24M**  **Log2**  **HBX /SILAM**  **2D-LC** | **Mean**  **value_HBx**  **vs SILAM** | **SD** | **24M**  **Log2**  **WT/SILAM**  **GEL** | **24 M**  **Log2**  **WT /SILAM**  **2D-LC** | **Mean**  **value_WT**  **vs SILAM** | **SD** | （**24M**  **Log2**  **HBx/24M WT**） |
| --- | --- | --- | --- | --- | --- | --- | --- | --- | --- | --- | --- |
| P97501 | FMO3 | 60.515 | -1.50 | -1.30 | -1.40 | 0.14 | -4.81 | -4.58 | -4.70 | 0.17 | 3.29 |
| P24452 | CAPG | 39.24 | 4.15 | 4.01 | 4.08 | 0.10 | 0.98 | 1.11 | 1.04 | 0.09 | 3.03 |
| P43276 | HIST1H1B | 22.576 | -0.03 | -0.18 | -0.11 | 0.11 | -2.80 | -2.90 | -2.85 | 0.07 | 2.74 |
| P19639 | GSTM3 | 25.701 | 2.76 | 2.91 | 2.83 | 0.10 | 0.16 | 0.24 | 0.20 | 0.05 | 2.64 |
| P12790 | CYP2B9 | 55.74 | -1.59 | -1.65 | -1.62 | 0.04 | -3.99 | -3.97 | -3.98 | 0.01 | 2.36 |
| P43275 | HIST1H1A | 21.785 | 0.81 | 0.63 | 0.72 | 0.13 | -1.69 | -1.51 | -1.60 | 0.12 | 2.31 |
| P14901 | HMOX1 | 32.928 | 2.52 | 2.51 | 2.52 | 0.00 | 0.06 | 0.36 | 0.21 | 0.21 | 2.30 |
| P21300 | AKR1B7 | 35.988 | 0.67 | 0.24 | 0.46 | 0.31 | -1.29 | -2.39 | -1.84 | 0.78 | 2.30 |
| O89053 | CORO1A | 50.989 | 2.61 | 2.65 | 2.63 | 0.03 | 0.26 | 0.46 | 0.36 | 0.14 | 2.27 |
| Q00612 | G6PDX | 59.262 | 1.69 | 1.75 | 1.72 | 0.04 | -0.49 | -0.44 | -0.47 | 0.03 | 2.19 |
| P20152 | VIM | 53.687 | 2.12 | 1.99 | 2.06 | 0.09 | -0.21 | -0.02 | -0.11 | 0.13 | 2.17 |

| Q61599 | ARHGDIB | 22.851 | 2.03 | 2.13 | 2.08 | 0.07 | -0.13 | 0.07 | -0.03 | 0.14 | 2.11 |
| --- | --- | --- | --- | --- | --- | --- | --- | --- | --- | --- | --- |
| P52480 | PKM | 57.844 | 2.05 | 1.80 | 1.93 | 0.17 | -0.05 | -0.18 | -0.12 | 0.09 | 2.04 |
| P39749 | FEN1 | 42.314 | 1.27 | 1.23 | 1.25 | 0.03 | -1.06 | -0.51 | -0.78 | 0.38 | 2.04 |
| Q3U5Q7 | CMPK2 | 50.036 | 0.90 | 0.74 | 0.82 | 0.11 | -1.12 | -1.25 | -1.19 | 0.09 | 2.00 |
| P37804 | TAGLN | 22.576 | 2.09 | 2.16 | 2.12 | 0.05 | 0.10 | 0.21 | 0.15 | 0.07 | 1.97 |
| Q9CQI6 | COTL1 | 15.944 | 1.89 | 1.80 | 1.85 | 0.07 | -0.09 | -0.12 | -0.10 | 0.03 | 1.95 |
| P06800 | PTPRC | 144.6 | 1.88 | 2.36 | 2.12 | 0.34 | 0.06 | 0.38 | 0.22 | 0.22 | 1.90 |
| Q9Z0E6 | GBP2 | 66.739 | 0.78 | 0.81 | 0.80 | 0.02 | -0.96 | -1.18 | -1.07 | 0.16 | 1.87 |
| P29351 | PTPN6 | 67.558 | 1.50 | 1.82 | 1.66 | 0.23 | -0.33 | -0.05 | -0.19 | 0.20 | 1.84 |
| P10107 | ANXA1 | 38.734 | 2.33 | 2.33 | 2.33 | 0.00 | 0.24 | 0.74 | 0.49 | 0.35 | 1.84 |
| Q9WVA4 | TAGLN2 | 22.395 | 1.73 | 1.74 | 1.73 | 0.00 | -0.13 | 0.01 | -0.06 | 0.10 | 1.79 |
| Q08857 | CD36 | 52.697 | 0.80 | 0.95 | 0.88 | 0.10 | -0.92 | -0.88 | -0.90 | 0.03 | 1.77 |
| Q04447 | CKB | 42.713 | 1.55 | 1.37 | 1.46 | 0.13 | -0.25 | -0.36 | -0.31 | 0.08 | 1.77 |

| Q9R0X4 | ACOT9 | 50.56 | 1.83 | 1.82 | 1.83 | 0.01 | 0.02 | 0.11 | 0.07 | 0.06 | 1.76 |
| --- | --- | --- | --- | --- | --- | --- | --- | --- | --- | --- | --- |
| Q80X19 | COL14A1 | 193.01 | 0.86 | 1.48 | 1.17 | 0.44 | -0.68 | -0.51 | -0.59 | 0.12 | 1.76 |
| P19324 | SERPINH1 | 46.533 | 0.06 | -0.45 | -0.19 | 0.36 | -1.92 | -1.97 | -1.94 | 0.03 | 1.75 |
| Q8JZK9 | HMGCS1 | 57.568 | -0.15 | -0.10 | -0.12 | 0.03 | -1.99 | -1.74 | -1.86 | 0.18 | 1.74 |
| Q60715 | P4HA1 | 60.909 | 0.76 | 0.70 | 0.73 | 0.04 | -0.97 | -1.04 | -1.01 | 0.05 | 1.74 |
| P26645 | MARCKS | 29.661 | 1.27 | 1.21 | 1.24 | 0.04 | -0.59 | -0.38 | -0.49 | 0.15 | 1.73 |
| Q9JKF1 | IQGAP1 | 188.74 | 1.71 | 1.75 | 1.73 | 0.03 | -0.03 | 0.07 | 0.02 | 0.07 | 1.71 |
| Q8R2Q8 | BST2 | 19.152 | 0.65 | 0.06 | 0.36 | 0.41 | -1.42 | -1.26 | -1.34 | 0.11 | 1.70 |
| Q99P72 | RTN4 | 126.61 | 0.71 | 0.71 | 0.71 | 0.00 | -1.19 | -0.77 | -0.98 | 0.29 | 1.69 |
| Q8BMK4 | CKAP4 | 63.691 | 1.02 | 1.04 | 1.03 | 0.01 | -0.64 | -0.61 | -0.62 | 0.02 | 1.66 |
| Q60854 | SERPINB6 | 42.598 | 1.59 | 1.77 | 1.68 | 0.13 | -0.27 | 0.39 | 0.06 | 0.47 | 1.62 |
| P30681 | HMGB2 | 24.162 | 1.03 | 1.04 | 1.04 | 0.00 | -0.57 | -0.56 | -0.57 | 0.00 | 1.60 |
| P42225 | STAT1 | 87.196 | 0.46 | 0.42 | 0.44 | 0.03 | -1.11 | -1.17 | -1.14 | 0.04 | 1.58 |

| P26041 | MSN | 67.766 | 1.88 | 1.60 | 1.74 | 0.20 | 0.19 | 0.18 | 0.18 | 0.01 | 1.56 |
| --- | --- | --- | --- | --- | --- | --- | --- | --- | --- | --- | --- |
| P48036 | ANXA5 | 35.752 | 1.35 | 1.12 | 1.24 | 0.16 | -0.29 | -0.34 | -0.32 | 0.04 | 1.55 |
| P10852 | SLC3A2 | 58.336 | 1.48 | 1.80 | 1.64 | 0.22 | 0.09 | 0.09 | 0.09 | 0.00 | 1.55 |
| Q62351 | TFRC | 85.73 | 1.20 | 0.98 | 1.09 | 0.16 | -0.31 | -0.59 | -0.45 | 0.20 | 1.54 |
| P26040 | EZR | 69.406 | 1.50 | 1.55 | 1.53 | 0.04 | -0.36 | 0.35 | 0.00 | 0.50 | 1.53 |
| P23780 | GLB1 | 73.12 | 0.99 | 1.20 | 1.09 | 0.15 | -0.58 | -0.29 | -0.43 | 0.21 | 1.53 |
| P82198 | TGFBI | 74.596 | 1.82 | 2.07 | 1.94 | 0.17 | 0.60 | 0.27 | 0.43 | 0.23 | 1.51 |
| Q9D154 | SERPINB1A | 42.574 | 2.14 | 2.25 | 2.19 | 0.08 | 0.55 | 0.82 | 0.69 | 0.19 | 1.51 |
| Q99LR1 | ABHD12 | 45.269 | 1.39 | 1.36 | 1.37 | 0.02 | -0.25 | 0.04 | -0.11 | 0.21 | 1.48 |
| Q8BTM8 | FLNA | 281.22 | 1.68 | 1.96 | 1.82 | 0.19 | 0.21 | 0.47 | 0.34 | 0.18 | 1.48 |
| P31725 | S100A9 | 13.049 | 3.64 | 3.69 | 3.66 | 0.04 | 2.14 | 2.25 | 2.19 | 0.08 | 1.47 |
| Q9JLQ0 | CD2AP | 70.449 | 0.45 | 0.54 | 0.50 | 0.07 | -1.48 | -0.39 | -0.93 | 0.77 | 1.43 |
| Q62393 | TPD52 | 24.313 | 1.12 | 1.23 | 1.17 | 0.08 | -0.63 | 0.15 | -0.24 | 0.55 | 1.41 |

| Q62523 | ZYX | 60.545 | 1.36 | 1.45 | 1.40 | 0.06 | -0.20 | 0.19 | -0.01 | 0.28 | 1.41 |
| --- | --- | --- | --- | --- | --- | --- | --- | --- | --- | --- | --- |
| Q9DCJ9 | NPL | 35.13 | 1.46 | 1.44 | 1.45 | 0.01 | 0.07 | 0.02 | 0.04 | 0.04 | 1.41 |
| Q9R0Y5 | AK1 | 21.539 | -2.00 | -1.95 | -1.98 | 0.03 | -3.36 | -3.39 | -3.37 | 0.03 | 1.40 |
| P35441 | THBS1 | 129.65 | 2.44 | 2.99 | 2.72 | 0.39 | 1.16 | 1.49 | 1.32 | 0.23 | 1.39 |
| Q9EQP2 | EHD4 | 61.48 | 1.14 | 1.23 | 1.18 | 0.06 | -0.25 | -0.16 | -0.21 | 0.07 | 1.39 |
| Q9R0E2 | PLOD1 | 83.594 | 1.02 | 1.13 | 1.08 | 0.08 | -0.27 | -0.35 | -0.31 | 0.06 | 1.39 |
| P02469 | LAMB1 | 197.09 | -0.46 | 0.04 | -0.21 | 0.35 | -1.65 | -1.48 | -1.56 | 0.12 | 1.36 |
| P97287 | MCL1 | 35.217 | 0.59 | 0.62 | 0.60 | 0.02 | -1.16 | -0.34 | -0.75 | 0.58 | 1.35 |
|  |  | 42.009 | 1.94 | 1.82 | 1.88 | 0.09 | 0.51 | 0.56 | 0.54 | 0.04 | 1.35 |
| P51125 | CAST | 84.921 | -0.14 | 0.40 | 0.13 | 0.38 | -1.68 | -0.71 | -1.20 | 0.69 | 1.33 |
| Q9D1A2 | CNDP2 | 52.767 | 0.65 | 0.57 | 0.61 | 0.06 | -0.78 | -0.66 | -0.72 | 0.09 | 1.32 |
| Q9JJU8 | SH3BGRL | 12.811 | 1.08 | 1.02 | 1.05 | 0.04 | -0.34 | -0.20 | -0.27 | 0.10 | 1.32 |
| Q9QYR7 | ACOT3 | 47.489 | -0.75 | -0.65 | -0.70 | 0.07 | -2.16 | -1.85 | -2.01 | 0.22 | 1.31 |

| P07356 | ANXA2 | 38.676 | 1.44 | 1.19 | 1.31 | 0.18 | 0.07 | -0.02 | 0.02 | 0.06 | 1.29 |
| --- | --- | --- | --- | --- | --- | --- | --- | --- | --- | --- | --- |
| O35660 | GSTM6 | 25.621 | 0.80 | 0.86 | 0.83 | 0.04 | -0.49 | -0.43 | -0.46 | 0.05 | 1.29 |
| Q8CI94 | PYGB | 96.729 | -0.40 | -0.45 | -0.43 | 0.04 | -1.67 | -1.74 | -1.70 | 0.05 | 1.28 |
| Q61285 | ABCD2 | 83.482 | -1.06 | -1.16 | -1.11 | 0.07 | -1.14 | -3.62 | -2.38 | 1.76 | 1.27 |
| P45376 | AKR1B1 | 35.732 | 1.29 | 1.13 | 1.21 | 0.11 | 0.02 | -0.10 | -0.04 | 0.09 | 1.25 |
| P28666 | MUG2 | 162.38 | -0.31 | 0.02 | -0.14 | 0.23 | -1.57 | -1.20 | -1.38 | 0.26 | 1.24 |
| P15864 | HIST1H1C | 21.266 | 1.05 | 1.03 | 1.04 | 0.01 | -0.20 | -0.17 | -0.18 | 0.02 | 1.22 |
| Q9CZE3 | RAB32 | 25.068 | 1.14 | 1.03 | 1.09 | 0.08 | -0.14 | -0.13 | -0.14 | 0.00 | 1.22 |
| Q8CHH9 | 42255 | 49.812 | 1.31 | 1.20 | 1.26 | 0.07 | -0.07 | 0.14 | 0.04 | 0.15 | 1.22 |
| Q9CWP6 | MOSPD2 | 59.854 | 0.35 | 0.49 | 0.42 | 0.10 | -1.22 | -0.37 | -0.79 | 0.60 | 1.22 |
| Q9JI11 | STK4;STK3 | 55.541 | 0.72 | 0.92 | 0.82 | 0.14 | -0.53 | -0.25 | -0.39 | 0.20 | 1.21 |
| Q64669 | NQO1 | 30.959 | 0.70 | 0.65 | 0.67 | 0.03 | -0.58 | -0.48 | -0.53 | 0.08 | 1.20 |
| P31001 | DES | 53.497 | 0.55 | 0.67 | 0.61 | 0.08 | -0.75 | -0.44 | -0.59 | 0.22 | 1.20 |

| P70194 | CLEC4F | 61.268 | 1.31 | 1.49 | 1.40 | 0.13 | 0.22 | 0.17 | 0.20 | 0.04 | 1.20 |
| --- | --- | --- | --- | --- | --- | --- | --- | --- | --- | --- | --- |
| Q9Z1G3 | ATP6V1C1 | 43.887 | 1.35 | 1.18 | 1.26 | 0.12 | 0.08 | 0.04 | 0.06 | 0.03 | 1.20 |
| Q9R233 | TAPBP | 49.736 | 0.53 | 0.66 | 0.59 | 0.09 | -0.68 | -0.53 | -0.60 | 0.11 | 1.20 |
| Q62418 | DBNL | 48.699 | 0.67 | 0.75 | 0.71 | 0.05 | -0.85 | -0.12 | -0.49 | 0.51 | 1.19 |
| Q3U9G9 | LBR | 71.439 | 0.39 | 0.36 | 0.38 | 0.02 | -0.97 | -0.64 | -0.80 | 0.23 | 1.18 |
| Q8VCC2 | CES1 | 62.679 | 0.11 | 0.00 | 0.06 | 0.08 | -1.16 | -1.07 | -1.12 | 0.06 | 1.17 |
| Q9JK53 | PRELP | 43.292 | 1.69 | 1.82 | 1.76 | 0.09 | 0.45 | 0.75 | 0.60 | 0.21 | 1.16 |
| Q8CIN4 | PAK2 | 57.93 | 0.56 | 0.68 | 0.62 | 0.08 | -0.81 | -0.26 | -0.53 | 0.39 | 1.16 |
| Q61233 | LCP1 | 70.148 | 1.55 | 1.58 | 1.57 | 0.02 | 0.46 | 0.37 | 0.41 | 0.07 | 1.15 |
| P48962 | SLC25A4 | 32.904 | -1.16 | -1.19 | -1.18 | 0.02 | -2.24 | -2.39 | -2.31 | 0.11 | 1.14 |
| Q00915 | RBP1 | 15.846 | 0.38 | 0.58 | 0.48 | 0.14 | -0.66 | -0.65 | -0.65 | 0.01 | 1.13 |
| Q6P5D8 | SMCHD1 | 225.65 | 0.38 | 0.61 | 0.49 | 0.16 | -0.55 | -0.73 | -0.64 | 0.13 | 1.13 |
| P05064 | ALDOA | 39.355 | 0.93 | 0.69 | 0.81 | 0.17 | -0.26 | -0.35 | -0.31 | 0.06 | 1.12 |

| P13516 | SCD1 | 41.046 | -0.16 | -0.21 | -0.19 | 0.04 | -1.38 | -1.23 | -1.31 | 0.11 | 1.12 |
| --- | --- | --- | --- | --- | --- | --- | --- | --- | --- | --- | --- |
| Q62422 | OSTF1 | 23.782 | 0.86 | 0.81 | 0.83 | 0.03 | -0.34 | -0.21 | -0.27 | 0.10 | 1.11 |
| O70439 | STX7 | 29.82 | 1.22 | 1.02 | 1.12 | 0.14 | 0.17 | -0.14 | 0.01 | 0.21 | 1.11 |
| Q9DCD0 | PGD | 53.247 | -0.05 | -0.21 | -0.13 | 0.11 | -1.27 | -1.19 | -1.23 | 0.05 | 1.10 |
| O35639 | ANXA3 | 36.384 | 1.12 | 0.98 | 1.05 | 0.10 | -0.04 | -0.05 | -0.05 | 0.00 | 1.10 |
| Q9Z1Q5 | CLIC1 | 27.013 | 1.34 | 1.31 | 1.32 | 0.02 | 0.25 | 0.19 | 0.22 | 0.04 | 1.10 |
| Q8K297 | COLGALT1 | 71.06 | 1.07 | 0.97 | 1.02 | 0.07 | -0.20 | 0.04 | -0.08 | 0.17 | 1.10 |
| Q9WTQ5 | AKAP12 | 180.69 | -0.64 | -0.11 | -0.37 | 0.37 | -2.09 | -0.82 | -1.45 | 0.89 | 1.08 |
| O70400 | PDLIM1 | 35.774 | 0.93 | 0.81 | 0.87 | 0.09 | -0.32 | -0.09 | -0.21 | 0.16 | 1.08 |
| Q6IRU2 | TPM4 | 28.467 | 1.12 | 1.03 | 1.08 | 0.06 | 0.05 | -0.03 | 0.01 | 0.06 | 1.06 |
| Q62465 | VAT1 | 43.096 | 1.77 | 1.64 | 1.70 | 0.09 | 0.56 | 0.73 | 0.65 | 0.12 | 1.06 |
| Q14DH7 | ACSS3 | 74.517 | -1.17 | -1.18 | -1.18 | 0.01 | -2.18 | -2.28 | -2.23 | 0.07 | 1.05 |
| P97792 | CXADR | 39.947 | -0.13 | -0.06 | -0.09 | 0.05 | -1.60 | -0.68 | -1.14 | 0.65 | 1.04 |

| P40124 | CAP1 | 51.564 | 0.81 | 0.91 | 0.86 | 0.07 | -0.28 | -0.06 | -0.17 | 0.16 | 1.03 |
| --- | --- | --- | --- | --- | --- | --- | --- | --- | --- | --- | --- |
| Q9D8B3 |  | 24.936 | 0.72 | 0.81 | 0.77 | 0.07 | -0.75 | 0.25 | -0.25 | 0.71 | 1.02 |
| Q8C129 | LNPEP | 117.3 | 0.81 | 1.13 | 0.97 | 0.22 | -0.10 | 0.01 | -0.04 | 0.07 | 1.01 |
| Q61029 | TMPO | 50.372 | 0.48 | 0.42 | 0.45 | 0.05 | -0.72 | -0.40 | -0.56 | 0.23 | 1.01 |
| Q9QYR9 | ACOT2 | 49.656 | 0.57 | 0.53 | 0.55 | 0.03 | -0.44 | -0.48 | -0.46 | 0.03 | 1.00 |
| P62814 | ATP6V1B2 | 56.55 | 0.76 | 0.84 | 0.80 | 0.06 | -0.37 | -0.03 | -0.20 | 0.24 | 1.00 |
| Q8R0H9 | GGA1 | 69.971 | -0.07 | 0.53 | 0.23 | 0.42 | -1.26 | -0.27 | -0.76 | 0.70 | 1.00 |
| Q61166 | MAPRE1 | 30.016 | 0.57 | 0.42 | 0.50 | 0.10 | -0.63 | -0.36 | -0.50 | 0.19 | 0.99 |
| P58044 | IDI1 | 26.289 | 0.34 | 0.40 | 0.37 | 0.04 | -0.66 | -0.59 | -0.62 | 0.05 | 0.99 |
| P51885 | LUM | 38.265 | 0.99 | 1.37 | 1.18 | 0.27 | 0.07 | 0.32 | 0.19 | 0.18 | 0.99 |
| Q61503 | NT5E | 63.864 | 0.17 | 0.17 | 0.17 | 0.00 | -1.29 | -0.33 | -0.81 | 0.68 | 0.98 |
| P99024 | TUBB5 | 49.67 | 0.51 | 0.78 | 0.65 | 0.19 | -0.62 | -0.05 | -0.34 | 0.41 | 0.98 |
| Q80UG5 | 42256 | 65.574 | 0.09 | 0.31 | 0.20 | 0.16 | -0.99 | -0.56 | -0.78 | 0.31 | 0.98 |

| Q07797 | LGALS3BP | 64.49 | 0.65 | 0.85 | 0.75 | 0.14 | 0.02 | -0.48 | -0.23 | 0.35 | 0.98 |
| --- | --- | --- | --- | --- | --- | --- | --- | --- | --- | --- | --- |
| Q9JK48 | SH3GLB1 | 40.855 | 0.65 | 0.80 | 0.72 | 0.11 | -0.40 | -0.11 | -0.25 | 0.20 | 0.98 |
| O88456 | CAPNS1 | 28.463 | 0.72 | 0.65 | 0.68 | 0.05 | -0.59 | 0.01 | -0.29 | 0.43 | 0.97 |
| P47740 | ALDH3A2 | 53.97 | 0.13 | 0.25 | 0.19 | 0.08 | -0.89 | -0.67 | -0.78 | 0.16 | 0.97 |
| Q9QUJ7 | ACSL4 | 79.076 | 0.82 | 0.76 | 0.79 | 0.04 | -0.24 | -0.12 | -0.18 | 0.08 | 0.97 |
| P21981 | TGM2 | 77.06 | 0.89 | 0.79 | 0.84 | 0.07 | -0.10 | -0.16 | -0.13 | 0.04 | 0.97 |
| P28653 | BGN | 41.639 | 0.76 | 1.83 | 1.29 | 0.75 | -0.08 | 0.74 | 0.33 | 0.58 | 0.97 |
| Q8R1Q8 | DYNC1LI1 | 56.614 | 0.33 | 0.20 | 0.27 | 0.09 | -1.13 | -0.26 | -0.70 | 0.62 | 0.96 |
| P05063 | ALDOC | 39.394 | 1.10 | 1.08 | 1.09 | 0.01 | 0.19 | 0.07 | 0.13 | 0.08 | 0.96 |
| O08553 | DPYSL2 | 62.277 | 0.97 | 1.18 | 1.08 | 0.15 | 0.01 | 0.24 | 0.13 | 0.16 | 0.95 |
| P00493 | HPRT1 | 24.57 | 1.19 | 1.28 | 1.24 | 0.06 | 0.24 | 0.34 | 0.29 | 0.07 | 0.95 |
| P10648 | GSTA2 | 25.542 | 3.54 | 3.71 | 3.62 | 0.12 | 2.56 | 2.80 | 2.68 | 0.17 | 0.95 |
| Q9D8Y0 | EFHD2 | 26.791 | 1.41 | 1.36 | 1.39 | 0.03 | 0.25 | 0.65 | 0.45 | 0.28 | 0.94 |

| Q8K2Q9 | KIAA1598 | 71.342 | 0.24 | 0.68 | 0.46 | 0.31 | -0.88 | -0.08 | -0.48 | 0.57 | 0.94 |
| --- | --- | --- | --- | --- | --- | --- | --- | --- | --- | --- | --- |
| Q9D8U8 | SNX5 | 46.797 | 0.93 | 0.89 | 0.91 | 0.02 | -0.15 | 0.10 | -0.03 | 0.17 | 0.94 |
| Q9Z2D6 | MECP2 | 52.307 | 0.73 | 1.05 | 0.89 | 0.23 | -0.81 | 0.73 | -0.04 | 1.09 | 0.93 |
| Q9WVK4 | EHD1 | 60.602 | 0.92 | 0.97 | 0.94 | 0.03 | -0.12 | 0.14 | 0.01 | 0.19 | 0.93 |
| Q8BK48 | CES2E | 62.317 | -0.20 | -0.24 | -0.22 | 0.03 | -1.17 | -1.11 | -1.14 | 0.04 | 0.92 |
| P68134 | ACTA1 | 42.051 | 1.11 | 1.01 | 1.06 | 0.07 | 0.13 | 0.14 | 0.13 | 0.00 | 0.92 |
| A3KMP2 | TTC38 | 52.223 | 0.15 | -0.08 | 0.03 | 0.16 | -0.81 | -0.95 | -0.88 | 0.10 | 0.92 |
| Q99PT1 | ARHGDIA | 23.407 | 0.87 | 0.91 | 0.89 | 0.03 | -0.07 | 0.03 | -0.02 | 0.07 | 0.91 |
| Q9WV32 | ARPC1B | 41.063 | 1.18 | 0.86 | 1.02 | 0.22 | 0.19 | 0.04 | 0.11 | 0.10 | 0.91 |
| Q5FWK3 | ARHGAP1 | 50.41 | 0.76 | 0.67 | 0.71 | 0.06 | -0.32 | -0.06 | -0.19 | 0.18 | 0.90 |
| Q9QZ08 | NAGK | 37.268 | 0.78 | 0.67 | 0.72 | 0.07 | -0.30 | -0.05 | -0.18 | 0.18 | 0.90 |
| Q80WJ7 | MTDH | 63.845 | -0.22 | 0.12 | -0.05 | 0.24 | -1.49 | -0.40 | -0.94 | 0.77 | 0.89 |
| P50518 | ATP6V1E1 | 26.157 | 0.98 | 0.84 | 0.91 | 0.10 | 0.02 | 0.03 | 0.02 | 0.01 | 0.89 |

| O08915 | AIP | 37.605 | 0.71 | 0.48 | 0.59 | 0.16 | -0.39 | -0.21 | -0.30 | 0.13 | 0.89 |
| --- | --- | --- | --- | --- | --- | --- | --- | --- | --- | --- | --- |
| O88342 | WDR1 | 66.406 | 0.95 | 0.94 | 0.94 | 0.01 | 0.11 | 0.00 | 0.05 | 0.07 | 0.89 |
| Q9CX80 | CYGB | 21.465 | 1.96 | 1.91 | 1.93 | 0.04 | 0.93 | 1.17 | 1.05 | 0.18 | 0.88 |
| Q9WUM4 | CORO1C | 53.12 | 0.96 | 0.87 | 0.91 | 0.07 | 0.07 | 0.00 | 0.03 | 0.05 | 0.88 |
| Q62419 | SH3GL1 | 41.518 | -0.03 | 0.04 | 0.00 | 0.05 | -1.29 | -0.46 | -0.88 | 0.59 | 0.88 |
| P11438 | LAMP1 | 43.865 | 1.74 | 1.09 | 1.42 | 0.46 | 0.69 | 0.38 | 0.54 | 0.22 | 0.88 |
| Q8VD75 | HIP1 | 115.2 | 1.02 | 1.40 | 1.21 | 0.27 | 0.49 | 0.18 | 0.33 | 0.22 | 0.87 |
| P62962 | PFN1 | 14.957 | 0.64 | 0.73 | 0.69 | 0.07 | -0.20 | -0.16 | -0.18 | 0.03 | 0.87 |
| Q8BVY0 | RSL1D1 | 50.421 | -0.28 | -0.28 | -0.28 | 0.00 | -1.27 | -1.02 | -1.15 | 0.18 | 0.87 |
| O08539 | BIN1 | 64.469 | 0.62 | 0.64 | 0.63 | 0.02 | -0.56 | 0.08 | -0.24 | 0.45 | 0.87 |
| Q9CXL3 |  | 22.168 | 0.74 | 0.96 | 0.85 | 0.15 | -0.20 | 0.16 | -0.02 | 0.25 | 0.87 |
| O70318 | EPB41L2 | 109.94 | -0.07 | 0.50 | 0.22 | 0.40 | -1.20 | -0.10 | -0.65 | 0.78 | 0.86 |
| P68510 | YWHAH | 28.211 | 0.77 | 0.70 | 0.74 | 0.05 | -0.10 | -0.15 | -0.13 | 0.03 | 0.86 |

| Q60710 | SAMHD1 | 72.65 | 0.83 | 0.96 | 0.90 | 0.09 | 0.05 | 0.03 | 0.04 | 0.02 | 0.85 |
| --- | --- | --- | --- | --- | --- | --- | --- | --- | --- | --- | --- |
| Q9CPW4 | ARPC5 | 16.288 | 0.82 | 0.82 | 0.82 | 0.01 | -0.19 | 0.12 | -0.03 | 0.22 | 0.85 |
| Q9D5V6 | SYAP1 | 41.349 | 0.14 | 0.19 | 0.16 | 0.04 | -1.02 | -0.36 | -0.69 | 0.46 | 0.85 |
| O09159 | MAN2B1 | 114.65 | 1.15 | 1.06 | 1.11 | 0.06 | 0.32 | 0.20 | 0.26 | 0.09 | 0.85 |
| P11276 | FN1 | 272.53 | 0.86 | 1.05 | 0.95 | 0.13 | 0.03 | 0.18 | 0.11 | 0.11 | 0.85 |
| P10833 | RRAS | 23.764 | 1.17 | 0.99 | 1.08 | 0.12 | -0.50 | 0.97 | 0.23 | 1.03 | 0.85 |
| P18760 | CFL1 | 18.559 | 0.76 | 0.91 | 0.83 | 0.11 | -0.03 | 0.01 | -0.01 | 0.03 | 0.85 |
| P63260 |  | 41.792 | 1.16 | 1.02 | 1.09 | 0.10 | 0.29 | 0.21 | 0.25 | 0.06 | 0.84 |
| O08638 | MYH11 | 227.03 | 1.16 | 1.68 | 1.42 | 0.37 | 0.49 | 0.67 | 0.58 | 0.12 | 0.84 |
| P63082 | ATP6V0C | 15.808 | 1.65 | 1.72 | 1.69 | 0.05 | 0.72 | 0.98 | 0.85 | 0.19 | 0.84 |
| Q9WVJ3 | CPQ | 51.813 | 1.04 | 0.96 | 1.00 | 0.05 | 0.08 | 0.25 | 0.17 | 0.12 | 0.83 |
| Q9JHU2 | PALMD | 62.699 | -0.12 | 0.00 | -0.06 | 0.09 | -1.34 | -0.44 | -0.89 | 0.64 | 0.83 |
| Q60605 | MYL6 | 16.93 | 0.78 | 1.02 | 0.90 | 0.17 | 0.02 | 0.11 | 0.07 | 0.06 | 0.83 |

| Q8BFW7 | LPP | 65.89 | 0.26 | 0.45 | 0.35 | 0.14 | -0.88 | -0.07 | -0.47 | 0.58 | 0.83 |
| --- | --- | --- | --- | --- | --- | --- | --- | --- | --- | --- | --- |
| P26039 | TLN1 | 269.82 | 0.83 | 1.33 | 1.08 | 0.35 | 0.11 | 0.40 | 0.26 | 0.20 | 0.83 |
| Q8VDD5 | MYH9 | 226.37 | 0.74 | 1.04 | 0.89 | 0.21 | -0.04 | 0.17 | 0.06 | 0.15 | 0.82 |
| Q920E5 | FDPS | 40.581 | 0.39 | 0.14 | 0.27 | 0.18 | -0.59 | -0.52 | -0.55 | 0.05 | 0.82 |
| Q61792 | LASP1 | 29.994 | 0.66 | 0.70 | 0.68 | 0.03 | -0.38 | 0.11 | -0.14 | 0.35 | 0.82 |
| O88958 | GNPDA1 | 32.549 | 0.92 | 0.83 | 0.87 | 0.07 | 0.12 | 0.00 | 0.06 | 0.08 | 0.81 |
| Q6PDN3 | MYLK | 212.92 | 0.44 | 0.84 | 0.64 | 0.28 | -0.30 | -0.05 | -0.18 | 0.18 | 0.81 |
| Q9EQ61 | PES1 | 67.795 | 0.34 | 0.17 | 0.25 | 0.12 | -0.61 | -0.51 | -0.56 | 0.07 | 0.81 |
| P42208 | 42249 | 41.525 | 0.72 | 0.48 | 0.60 | 0.17 | -0.25 | -0.17 | -0.21 | 0.05 | 0.81 |
| Q9WV80 | SNX1 | 58.951 | 0.40 | 0.58 | 0.49 | 0.13 | -0.63 | 0.01 | -0.31 | 0.45 | 0.80 |
| O88983 | STX8 | 26.925 | 0.31 | 0.52 | 0.41 | 0.15 | -0.52 | -0.25 | -0.39 | 0.19 | 0.80 |
| Q8C650 | 42257 | 52.422 | 0.55 | 0.34 | 0.45 | 0.15 | -0.44 | -0.26 | -0.35 | 0.13 | 0.80 |
| O88833 | CYP4A10 | 58.33 | 1.50 | 1.55 | 1.52 | 0.04 | 0.63 | 0.82 | 0.72 | 0.13 | 0.80 |

| P11031 | SUB1 | 14.427 | 0.33 | 0.31 | 0.32 | 0.01 | -0.46 | -0.50 | -0.48 | 0.04 | 0.80 |
| --- | --- | --- | --- | --- | --- | --- | --- | --- | --- | --- | --- |
| Q9CWK8 | SNX2 | 58.47 | 0.79 | 0.73 | 0.76 | 0.05 | -0.13 | 0.07 | -0.03 | 0.14 | 0.79 |
| Q8K2K6 | AGFG1 | 58.042 | -0.47 | 0.44 | -0.02 | 0.64 | -1.41 | -0.20 | -0.80 | 0.86 | 0.78 |
| Q8VBT0 | TMX1 | 31.395 | 0.32 | 0.30 | 0.31 | 0.01 | -0.54 | -0.41 | -0.47 | 0.09 | 0.78 |
| O08529 | CAPN2 | 79.871 | 0.61 | 0.47 | 0.54 | 0.10 | -0.16 | -0.33 | -0.24 | 0.13 | 0.78 |
| Q9JLV1 | BAG3 | 61.859 | -0.19 | 0.05 | -0.07 | 0.17 | -1.43 | -0.27 | -0.85 | 0.82 | 0.78 |
| Q9Z1R2 | BAG6 | 121.04 | -0.15 | 0.31 | 0.08 | 0.32 | -1.10 | -0.30 | -0.70 | 0.57 | 0.78 |
| P28656 | NAP1L1 | 45.345 | 0.60 | 0.52 | 0.56 | 0.05 | -0.25 | -0.18 | -0.22 | 0.05 | 0.78 |
| P15626 | GSTM2 | 25.716 | 0.89 | 1.04 | 0.97 | 0.10 | 0.18 | 0.21 | 0.20 | 0.02 | 0.77 |
| P50516 | ATP6V1A | 68.325 | 0.77 | 0.76 | 0.76 | 0.01 | 0.04 | -0.05 | -0.01 | 0.06 | 0.77 |
| Q7TNE1 | SUGCT | 47.69 | 1.25 | 1.15 | 1.20 | 0.07 | 0.52 | 0.34 | 0.43 | 0.13 | 0.77 |
| Q3THS6 | MAT2A | 43.688 | 1.05 | 0.52 | 0.79 | 0.37 | 0.00 | 0.04 | 0.02 | 0.03 | 0.77 |
| Q04857 | COL6A1 | 108.49 | 1.76 | 1.84 | 1.80 | 0.06 | 1.22 | 0.84 | 1.03 | 0.26 | 0.77 |

| Q64455 | PTPRJ | 136.77 | 0.68 | 0.76 | 0.72 | 0.06 | 0.01 | -0.10 | -0.05 | 0.08 | 0.77 |
| --- | --- | --- | --- | --- | --- | --- | --- | --- | --- | --- | --- |
| P52840 | SULT1A1 | 33.974 | -0.03 | -0.13 | -0.08 | 0.07 | -0.86 | -0.83 | -0.84 | 0.02 | 0.76 |
| Q9Z2C5 | MTM1 | 69.558 | 0.86 | 0.64 | 0.75 | 0.15 | -0.01 | -0.02 | -0.01 | 0.01 | 0.76 |
|  |  | 44.12 | 0.81 | 0.49 | 0.65 | 0.23 | -0.31 | 0.08 | -0.12 | 0.28 | 0.76 |
| Q9CY64 | BLVRA | 33.524 | 0.77 | 0.70 | 0.73 | 0.05 | 0.10 | -0.16 | -0.03 | 0.19 | 0.76 |
| P07742 | RRM1 | 90.209 | -0.21 | -0.21 | -0.21 | 0.00 | -0.85 | -1.09 | -0.97 | 0.17 | 0.76 |
| O89110 | CASP8 | 55.356 | 0.15 | 0.20 | 0.18 | 0.04 | -0.66 | -0.51 | -0.58 | 0.11 | 0.76 |
| O89017 | LGMN | 49.372 | 1.80 | 1.69 | 1.74 | 0.07 | 0.96 | 1.01 | 0.99 | 0.03 | 0.76 |
| P63280 | UBE2I | 18.007 | 0.37 | 0.47 | 0.42 | 0.07 | -0.45 | -0.22 | -0.33 | 0.16 | 0.75 |
| O54724 | PTRF | 43.953 | 0.37 | 0.39 | 0.38 | 0.02 | -0.54 | -0.21 | -0.37 | 0.23 | 0.75 |
| Q61735 | CD47 | 33.097 | 1.09 | 0.84 | 0.97 | 0.18 | 0.27 | 0.16 | 0.21 | 0.07 | 0.75 |
| Q91WJ8 | FUBP1 | 68.539 | 0.20 | 0.25 | 0.22 | 0.04 | -0.57 | -0.49 | -0.53 | 0.06 | 0.75 |
|  |  | 19.779 | 0.86 | 0.85 | 0.85 | 0.01 | -0.05 | 0.26 | 0.10 | 0.22 | 0.75 |

| P60766 | CDC42 | 21.258 | 0.82 | 0.75 | 0.79 | 0.05 | -0.01 | 0.10 | 0.04 | 0.08 | 0.74 |
| --- | --- | --- | --- | --- | --- | --- | --- | --- | --- | --- | --- |
| Q921I1 | TF | 76.723 | 0.80 | 0.85 | 0.82 | 0.03 | 0.06 | 0.11 | 0.08 | 0.03 | 0.74 |
| Q9EPJ9 | ARFGAP1 | 45.288 | -0.16 | -0.02 | -0.09 | 0.10 | -1.14 | -0.52 | -0.83 | 0.44 | 0.74 |
| Q8BVQ5 | PPME1 | 42.256 | 0.12 | 0.12 | 0.12 | 0.00 | -0.98 | -0.25 | -0.61 | 0.51 | 0.73 |
| Q8C166 | CPNE1 | 58.886 | 0.41 | 0.26 | 0.34 | 0.10 | -0.44 | -0.35 | -0.40 | 0.06 | 0.73 |
| P43277 | HIST1H1D | 22.099 | 0.81 | 0.71 | 0.76 | 0.07 | -0.09 | 0.15 | 0.03 | 0.17 | 0.73 |
| Q7TN29 | SMAP2 | 46.577 | -0.23 | 0.21 | -0.01 | 0.31 | -1.35 | -0.12 | -0.74 | 0.87 | 0.73 |
| Q99KQ4 | NAMPT | 55.446 | 0.32 | 0.39 | 0.36 | 0.05 | -0.51 | -0.23 | -0.37 | 0.20 | 0.73 |
| Q9R1J0 | NSDHL | 40.685 | 0.53 | 0.31 | 0.42 | 0.16 | -0.37 | -0.24 | -0.30 | 0.09 | 0.73 |
| Q05816 | FABP5 | 15.137 | -0.96 | -1.11 | -1.04 | 0.11 | -1.69 | -1.83 | -1.76 | 0.10 | 0.72 |
| Q00519 | XDH | 146.56 | 0.13 | 0.43 | 0.28 | 0.21 | -0.70 | -0.18 | -0.44 | 0.36 | 0.72 |
| Q61696 |  |  | -0.05 | -0.01 | -0.03 | 0.03 | -0.65 | -0.85 | -0.75 | 0.14 | 0.72 |
| Q9QYA2 | TOMM40 | 37.895 | 0.40 | 0.01 | 0.20 | 0.28 | -1.04 | 0.01 | -0.52 | 0.74 | 0.72 |

| P49312 | HNRNPA1 | 34.196 | 0.82 | 0.70 | 0.76 | 0.08 | 0.08 | 0.02 | 0.05 | 0.04 | 0.72 |
| --- | --- | --- | --- | --- | --- | --- | --- | --- | --- | --- | --- |
| Q05793 | HSPG2 | 398.29 | 0.60 | 1.00 | 0.80 | 0.28 | -0.10 | 0.27 | 0.08 | 0.26 | 0.72 |
| P06801 | ME1 | 63.953 | -1.79 | -1.83 | -1.81 | 0.03 | -2.86 | -2.19 | -2.52 | 0.47 | 0.71 |
| Q64012 | RALY | 33.188 | 0.53 | 0.40 | 0.46 | 0.09 | -0.42 | -0.08 | -0.25 | 0.24 | 0.71 |
| P53810 | PITPNA | 31.893 | 0.82 | 0.79 | 0.80 | 0.02 | 0.14 | 0.04 | 0.09 | 0.07 | 0.71 |
| Q9R0P4 | SMAP | 20.046 | 0.25 | 0.35 | 0.30 | 0.07 | -0.83 | 0.00 | -0.41 | 0.58 | 0.71 |
|  |  |  | -0.58 | -0.63 | -0.61 | 0.04 | -1.38 | -1.25 | -1.32 | 0.09 | 0.71 |
| P25911 | LYN | 58.812 | 0.91 | 0.85 | 0.88 | 0.04 | 0.08 | 0.26 | 0.17 | 0.13 | 0.71 |
| Q8K0C4 | CYP51A1 | 56.775 | 0.09 | 0.25 | 0.17 | 0.11 | -0.65 | -0.42 | -0.53 | 0.17 | 0.71 |
| Q3UTJ2 | SORBS2 | 132.35 | 0.07 | 0.70 | 0.38 | 0.45 | -0.84 | 0.19 | -0.33 | 0.73 | 0.71 |
| Q9EQ32 | PIK3AP1 | 90.927 | 0.32 | 0.67 | 0.49 | 0.25 | -0.63 | 0.19 | -0.22 | 0.58 | 0.71 |
| Q9JJ28 | FLII | 144.8 | 0.27 | 0.47 | 0.37 | 0.14 | -0.73 | 0.05 | -0.34 | 0.56 | 0.71 |
| O55131 | 42254 | 50.549 | 0.86 | 0.66 | 0.76 | 0.15 | 0.07 | 0.03 | 0.05 | 0.03 | 0.71 |

| P47753 | CAPZA1 | 32.939 | 0.60 | 0.48 | 0.54 | 0.08 | -0.28 | -0.05 | -0.16 | 0.16 | 0.70 |
| --- | --- | --- | --- | --- | --- | --- | --- | --- | --- | --- | --- |
| Q07113 | IGF2R | 273.81 | 0.42 | 0.33 | 0.37 | 0.06 | -0.11 | -0.55 | -0.33 | 0.31 | 0.70 |
| Q8CI51 | PDLIM5 | 63.299 | -0.37 | -0.10 | -0.24 | 0.19 | -1.03 | -0.84 | -0.94 | 0.13 | 0.70 |
| Q5SWU9 | ACACA | 265.25 | -1.54 | -1.12 | -1.33 | 0.30 | -2.06 | -1.99 | -2.02 | 0.05 | 0.70 |
| O88712 | CTBP1 | 47.744 | 0.44 | 0.44 | 0.44 | 0.00 | -0.42 | -0.09 | -0.25 | 0.23 | 0.70 |
| Q6P8X1 | SNX6 | 46.648 | 0.66 | 0.45 | 0.56 | 0.15 | -0.19 | -0.09 | -0.14 | 0.07 | 0.70 |
| Q91YR1 | TWF1 | 40.079 | 0.88 | 0.69 | 0.78 | 0.13 | 0.01 | 0.17 | 0.09 | 0.11 | 0.70 |
| Q8BVE3 | ATP6V1H | 55.854 | 0.53 | 0.52 | 0.52 | 0.01 | -0.30 | -0.04 | -0.17 | 0.18 | 0.70 |
| Q6A028 | SWAP70 | 68.995 | 0.97 | 1.05 | 1.01 | 0.06 | 0.34 | 0.30 | 0.32 | 0.03 | 0.69 |
| Q8R0X7 | SGPL1 | 63.676 | 0.33 | 0.37 | 0.35 | 0.02 | -0.48 | -0.19 | -0.34 | 0.21 | 0.69 |
| P21279 | GNAQ | 42.158 | 1.18 | 0.47 | 0.83 | 0.50 | 0.13 | 0.15 | 0.14 | 0.01 | 0.69 |
| Q61249 | IGBP1 | 38.97 | 0.01 | -0.03 | -0.01 | 0.02 | -1.03 | -0.36 | -0.70 | 0.47 | 0.69 |
| Q8VCI0 | PLBD1 | 62.998 | 2.30 | 2.32 | 2.31 | 0.01 | 1.59 | 1.66 | 1.62 | 0.05 | 0.68 |

| P14428 | H2-K1 | 36.855 | 1.01 | 0.48 | 0.75 | 0.38 | 0.27 | -0.15 | 0.06 | 0.29 | 0.68 |
| --- | --- | --- | --- | --- | --- | --- | --- | --- | --- | --- | --- |
| Q9ER00 | STX12 | 31.195 | 0.87 | 0.69 | 0.78 | 0.12 | 0.07 | 0.12 | 0.10 | 0.03 | 0.68 |
| Q9DBM2 | EHHADH | 78.301 | 0.39 | 0.34 | 0.37 | 0.03 | -0.31 | -0.32 | -0.32 | 0.01 | 0.68 |
| P61164 | ACTR1A | 42.613 | 0.43 | 0.39 | 0.41 | 0.03 | -0.36 | -0.18 | -0.27 | 0.12 | 0.68 |
| Q60749 | KHDRBS1 | 48.37 | 0.29 | 0.50 | 0.40 | 0.15 | -0.55 | -0.02 | -0.28 | 0.38 | 0.68 |
| Q9DAW9 | CNN3 | 36.428 | 0.09 | 0.01 | 0.05 | 0.06 | -0.73 | -0.53 | -0.63 | 0.14 | 0.68 |
| Q9DCR2 | AP3S1 | 21.732 | 0.47 | 0.39 | 0.43 | 0.06 | -0.26 | -0.24 | -0.25 | 0.01 | 0.68 |
| Q64459 | CYP3A11 | 57.854 | 0.31 | 0.08 | 0.20 | 0.16 | -0.50 | -0.46 | -0.48 | 0.03 | 0.68 |
| Q9WV68 | DECR2 | 31.3 | -0.39 | -0.24 | -0.31 | 0.11 | -1.15 | -0.83 | -0.99 | 0.23 | 0.68 |
| Q9CVB6 | ARPC2 | 34.357 | 0.90 | 0.84 | 0.87 | 0.05 | 0.19 | 0.20 | 0.19 | 0.01 | 0.68 |
| Q6PGL7 | FAM21 | 145.31 | -0.08 | 0.29 | 0.10 | 0.26 | -0.99 | -0.15 | -0.57 | 0.59 | 0.67 |
| Q9R1T2 | SAE1 | 38.62 | 0.79 | 0.62 | 0.70 | 0.12 | -0.06 | 0.13 | 0.03 | 0.13 | 0.67 |
| Q9Z130 | HNRNPDL | 33.558 | 0.91 | 0.75 | 0.83 | 0.11 | 0.27 | 0.05 | 0.16 | 0.15 | 0.67 |

| P63101 | YWHAZ | 27.771 | 0.79 | 0.63 | 0.71 | 0.12 | 0.06 | 0.02 | 0.04 | 0.03 | 0.67 |
| --- | --- | --- | --- | --- | --- | --- | --- | --- | --- | --- | --- |
| Q60973 | RBBP7 | 47.789 | 0.46 | 0.25 | 0.35 | 0.15 | -0.29 | -0.34 | -0.32 | 0.04 | 0.67 |
| P13020 | GSN | 85.941 | -0.06 | 0.79 | 0.37 | 0.60 | -0.42 | -0.19 | -0.30 | 0.16 | 0.67 |
| P97371 | PSME1 | 28.673 | 0.05 | 0.10 | 0.07 | 0.03 | -0.65 | -0.55 | -0.60 | 0.07 | 0.67 |
| Q8C1B7 | 42258 | 49.694 | 0.86 | 0.70 | 0.78 | 0.11 | 0.18 | 0.06 | 0.12 | 0.08 | 0.66 |
| Q9EQK5 | MVP | 95.923 | 0.90 | 1.01 | 0.96 | 0.07 | 0.34 | 0.25 | 0.29 | 0.06 | 0.66 |
| Q60598 | CTTN | 61.249 | 0.66 | 0.62 | 0.64 | 0.02 | -0.19 | 0.14 | -0.02 | 0.24 | 0.66 |
| Q3UPH1 | PRRC1 | 46.297 | 0.26 | -0.01 | 0.12 | 0.19 | -1.06 | -0.02 | -0.54 | 0.74 | 0.66 |
| P08752 | GNAI2 | 40.489 | 0.92 | 0.85 | 0.89 | 0.05 | 0.29 | 0.16 | 0.22 | 0.10 | 0.66 |
| Q80YX1 | TNC | 231.8 | 0.09 | 0.87 | 0.48 | 0.55 | -0.58 | 0.22 | -0.18 | 0.56 | 0.66 |
| Q7TSV4 | PGM2 | 68.747 | 1.43 | 1.27 | 1.35 | 0.11 | 0.78 | 0.60 | 0.69 | 0.13 | 0.66 |
| P57759 | ERP29 | 28.823 | 0.43 | 0.47 | 0.45 | 0.03 | -0.29 | -0.12 | -0.21 | 0.12 | 0.66 |
| P20852 | CYP2A5 | 56.74 | 0.02 | 0.60 | 0.31 | 0.41 | -0.37 | -0.32 | -0.35 | 0.03 | 0.66 |

| Q9QXG4 | ACSS2 | 78.861 | -0.60 | -0.54 | -0.57 | 0.04 | -1.37 | -1.08 | -1.22 | 0.20 | 0.65 |
| --- | --- | --- | --- | --- | --- | --- | --- | --- | --- | --- | --- |
| Q9Z2G9 | HTATIP2 | 26.87 | 0.75 | 0.47 | 0.61 | 0.20 | -0.01 | -0.05 | -0.03 | 0.03 | 0.64 |
| Q9Z1F9 | UBA2 | 70.568 | 0.90 | 0.63 | 0.77 | 0.19 | 0.16 | 0.09 | 0.13 | 0.05 | 0.64 |
| Q78ZA7 | NAP1L4 | 42.679 | 0.44 | 0.34 | 0.39 | 0.07 | -0.28 | -0.23 | -0.25 | 0.03 | 0.64 |
| Q99KJ8 | DCTN2 | 44.116 | 0.46 | 0.22 | 0.34 | 0.17 | -0.37 | -0.23 | -0.30 | 0.10 | 0.64 |
| P35278 | RAB5C | 23.412 | 0.43 | 0.51 | 0.47 | 0.05 | -0.25 | -0.09 | -0.17 | 0.11 | 0.64 |
| Q8K2F8 | LSM14A | 50.545 | 0.13 | 0.11 | 0.12 | 0.01 | -0.98 | -0.06 | -0.52 | 0.65 | 0.64 |
| P17710 | HK1 | 108.3 | 0.60 | 0.96 | 0.78 | 0.25 | 0.08 | 0.21 | 0.15 | 0.09 | 0.64 |
| P97449 | ANPEP | 109.65 | -0.06 | 0.40 | 0.17 | 0.33 | -0.84 | -0.09 | -0.46 | 0.53 | 0.63 |
| P24472 | GSTA4 | 25.564 | 0.71 | 0.81 | 0.76 | 0.07 | 0.10 | 0.16 | 0.13 | 0.05 | 0.63 |
| Q3U7R1 | ESYT1 | 121.55 | 0.79 | 1.15 | 0.97 | 0.26 | 0.32 | 0.36 | 0.34 | 0.02 | 0.63 |
| Q8K2C7 | OS9 | 76.107 | -0.10 | -0.27 | -0.18 | 0.12 | -0.79 | -0.83 | -0.81 | 0.03 | 0.63 |
| Q61937 | NPM1 | 32.56 | 0.37 | 0.19 | 0.28 | 0.12 | -0.49 | -0.21 | -0.35 | 0.19 | 0.63 |

| A2AGT5 | CKAP5 | 225.63 | -0.25 | 0.27 | 0.01 | 0.37 | -1.16 | -0.07 | -0.62 | 0.77 | 0.63 |
| --- | --- | --- | --- | --- | --- | --- | --- | --- | --- | --- | --- |
| Q9ERG0 | LIMA1 | 84.059 | 0.30 | 0.69 | 0.50 | 0.27 | -0.28 | 0.02 | -0.13 | 0.21 | 0.62 |
| P70677 | CASP3 | 31.474 | -0.04 | -0.03 | -0.03 | 0.00 | -0.73 | -0.59 | -0.66 | 0.10 | 0.62 |
| Q61292 | LAMB2 | 196.58 | 0.67 | 0.90 | 0.78 | 0.16 | -0.06 | 0.38 | 0.16 | 0.31 | 0.62 |
| Q64324 | STXBP2 | 66.357 | 0.61 | 0.84 | 0.72 | 0.16 | 0.11 | 0.10 | 0.10 | 0.01 | 0.62 |
| O35350 | CAPN1 | 82.105 | 1.12 | 1.05 | 1.08 | 0.05 | 0.45 | 0.47 | 0.46 | 0.02 | 0.62 |
| Q9QWR8 | NAGA | 47.234 | 1.39 | 1.34 | 1.36 | 0.04 | 0.65 | 0.83 | 0.74 | 0.13 | 0.62 |
| Q8C142 | LDLRAP1 | 33.975 | 0.31 | 0.38 | 0.34 | 0.05 | -0.63 | 0.09 | -0.27 | 0.51 | 0.62 |
| P53808 | PCTP | 24.785 | -0.42 | -0.37 | -0.39 | 0.03 | -1.14 | -0.89 | -1.01 | 0.17 | 0.62 |
| Q99KN9 | CLINT1 | 68.512 | -0.16 | 0.08 | -0.04 | 0.17 | -1.21 | -0.11 | -0.66 | 0.78 | 0.62 |
| Q9Z2G6 | SEL1L | 88.339 | 0.17 | 0.01 | 0.09 | 0.11 | -0.60 | -0.46 | -0.53 | 0.10 | 0.62 |
| Q99JY3 | GIMAP4 | 24.554 | 0.28 | 0.13 | 0.20 | 0.10 | -0.43 | -0.39 | -0.41 | 0.03 | 0.62 |
| Q62452 |  | 60.007 | 2.56 | 2.29 | 2.43 | 0.20 | 3.90 | -0.28 | 1.81 | 2.95 | 0.61 |

| P61161 | ACTR2 | 44.76 | 0.77 | 0.79 | 0.78 | 0.01 | 0.11 | 0.21 | 0.16 | 0.07 | 0.61 |
| --- | --- | --- | --- | --- | --- | --- | --- | --- | --- | --- | --- |
| Q8QZY2 | GLYCTK | 55.292 | -0.14 | -0.07 | -0.10 | 0.05 | -0.89 | -0.54 | -0.71 | 0.25 | 0.61 |
| O55111 | DSG2 | 122.38 | -0.94 | -0.37 | -0.66 | 0.40 | -2.01 | -0.52 | -1.27 | 1.06 | 0.61 |
| P51150 | RAB7A | 23.489 | 0.49 | 0.44 | 0.46 | 0.03 | -0.19 | -0.10 | -0.15 | 0.06 | 0.61 |
| Q80WQ2 | VAC14 | 88.047 | 0.20 | 0.34 | 0.27 | 0.09 | -0.35 | -0.33 | -0.34 | 0.01 | 0.61 |
| O08997 | ATOX1 | 7.3384 | 0.11 | 0.45 | 0.28 | 0.25 | -0.30 | -0.36 | -0.33 | 0.04 | 0.61 |
| Q8C5W3 | TBCEL | 48.031 | -0.07 | -0.22 | -0.14 | 0.11 | -0.79 | -0.71 | -0.75 | 0.06 | 0.61 |
| P70336 | ROCK2 | 160.58 | 0.62 | 0.88 | 0.75 | 0.18 | 0.05 | 0.23 | 0.14 | 0.13 | 0.61 |
| Q8BGT5 | GPT2 | 57.943 | 1.11 | 1.13 | 1.12 | 0.02 | 0.40 | 0.63 | 0.51 | 0.16 | 0.61 |
| Q99KC8 | VWA5A | 87.142 | 0.15 | 0.35 | 0.25 | 0.14 | -0.56 | -0.14 | -0.35 | 0.29 | 0.61 |
| Q02788 | COL6A2 | 110.33 | 1.33 | 1.77 | 1.55 | 0.31 | 0.97 | 0.92 | 0.95 | 0.04 | 0.61 |
| O70252 | HMOX2 | 35.738 | 0.85 | 0.67 | 0.76 | 0.13 | 0.14 | 0.19 | 0.16 | 0.04 | 0.60 |
| Q9QYF1 | RDH11 | 35.147 | -0.49 | -0.50 | -0.50 | 0.01 | -1.17 | -1.03 | -1.10 | 0.10 | 0.60 |

| Q9WUZ9 | ENTPD5 | 47.101 | 1.07 | 0.94 | 1.01 | 0.09 | 0.31 | 0.51 | 0.41 | 0.14 | 0.60 |
| --- | --- | --- | --- | --- | --- | --- | --- | --- | --- | --- | --- |
| P34022 | RANBP1 | 23.596 | 0.52 | 0.30 | 0.41 | 0.15 | -0.18 | -0.19 | -0.18 | 0.00 | 0.60 |
| Q7TNV0 | DEK | 43.158 | 0.25 | 0.23 | 0.24 | 0.01 | -0.50 | -0.21 | -0.36 | 0.21 | 0.60 |
| P16675 | CTSA | 53.844 | 1.68 | 1.62 | 1.65 | 0.04 | 1.10 | 1.02 | 1.06 | 0.05 | 0.60 |
| Q922F4 | TUBB6 | 50.09 | 0.51 | 0.20 | 0.36 | 0.22 | -0.35 | -0.13 | -0.24 | 0.16 | 0.60 |
| P19096 | FASN | 272.43 | -1.96 | -1.67 | -1.81 | 0.20 | -2.58 | -2.24 | -2.41 | 0.24 | 0.60 |
| Q8VCT3 | RNPEP | 72.415 | 0.51 | 0.32 | 0.41 | 0.13 | -0.14 | -0.23 | -0.18 | 0.07 | 0.60 |
| Q9Z1G4 | ATP6V0A1 | 96.466 | 0.88 | 1.00 | 0.94 | 0.08 | 0.37 | 0.33 | 0.35 | 0.03 | 0.59 |
| Q61830 | MRC1 | 164.98 | 0.44 | 0.68 | 0.56 | 0.17 | -0.05 | -0.01 | -0.03 | 0.03 | 0.59 |
| Q99KH8 |  | 47.953 | 0.46 | 0.55 | 0.51 | 0.06 | -0.19 | 0.01 | -0.09 | 0.14 | 0.59 |
| Q8BVU5 | NUDT9 | 38.604 | 0.37 | 0.28 | 0.33 | 0.06 | -0.14 | -0.38 | -0.26 | 0.17 | 0.59 |
| P21107 | TPM3 | 32.994 | 0.87 | 0.82 | 0.84 | 0.03 | 0.31 | 0.19 | 0.25 | 0.08 | 0.59 |
| P14733 | LMNB1 | 66.785 | 0.35 | 0.45 | 0.40 | 0.07 | -0.11 | -0.26 | -0.19 | 0.11 | 0.59 |

| P48758 | CBR1 | 30.641 | 0.00 | -0.09 | -0.05 | 0.06 | -0.70 | -0.57 | -0.64 | 0.09 | 0.59 |
| --- | --- | --- | --- | --- | --- | --- | --- | --- | --- | --- | --- |
| Q8CHP5 | WIBG | 22.689 | 0.14 | -0.08 | 0.03 | 0.16 | -0.89 | -0.23 | -0.56 | 0.46 | 0.59 |
| Q91WP6 | SERPINA3N | 46.717 | 1.75 | 2.18 | 1.97 | 0.31 | 1.38 | 1.37 | 1.38 | 0.00 | 0.59 |
| P54823 | DDX6 | 54.191 | 0.20 | 0.46 | 0.33 | 0.18 | -0.75 | 0.24 | -0.25 | 0.70 | 0.59 |
| Q8R2V5 | ADAP2 | 43.989 | 1.16 | 1.18 | 1.17 | 0.02 | 0.56 | 0.62 | 0.59 | 0.04 | 0.58 |
| Q61334 | BCAP29 | 27.964 | 1.22 | 1.12 | 1.17 | 0.07 | 0.59 | 0.59 | 0.59 | 0.00 | 0.58 |
| Q9ER41 | TOR1B | 37.817 | 0.65 | 0.73 | 0.69 | 0.06 | 0.06 | 0.15 | 0.11 | 0.06 | 0.58 |
| P47757 | CAPZB | 31.345 | 0.64 | 0.60 | 0.62 | 0.03 | 0.05 | 0.03 | 0.04 | 0.01 | 0.57 |
| P58771 | TPM1 | 32.68 | 0.49 | 0.21 | 0.35 | 0.20 | -0.02 | -0.42 | -0.22 | 0.28 | 0.57 |
| P01837 |  | 11.778 | 4.53 | 4.31 | 4.42 | 0.15 | 2.79 | 4.91 | 3.85 | 1.50 | 0.57 |
| P47754 | CAPZA2 | 32.967 | 0.95 | 0.65 | 0.80 | 0.21 | 0.19 | 0.26 | 0.23 | 0.05 | 0.57 |
| P27546 | MAP4 | 117.43 | -0.47 | 0.43 | -0.02 | 0.64 | -1.02 | -0.16 | -0.59 | 0.61 | 0.57 |
| Q05D44 | EIF5B | 137.61 | -0.62 | 0.04 | -0.29 | 0.47 | -1.53 | -0.20 | -0.86 | 0.94 | 0.57 |

| O55126 | GBAS | 32.932 | 0.15 | 0.11 | 0.13 | 0.02 | -0.45 | -0.43 | -0.44 | 0.02 | 0.57 |
| --- | --- | --- | --- | --- | --- | --- | --- | --- | --- | --- | --- |
| Q99JR8 | SMARCD2 | 59.084 | -0.02 | 0.42 | 0.20 | 0.31 | -0.83 | 0.09 | -0.37 | 0.66 | 0.57 |
| Q61749 | EIF2B4 | 57.624 | -0.24 | -0.09 | -0.16 | 0.10 | -1.12 | -0.34 | -0.73 | 0.56 | 0.57 |
| P97372 | PSME2 | 27.057 | -0.03 | 0.05 | 0.01 | 0.06 | -0.59 | -0.52 | -0.55 | 0.04 | 0.56 |
| Q9WU84 | CCS | 28.911 | 0.58 | 0.26 | 0.42 | 0.23 | -0.22 | -0.06 | -0.14 | 0.11 | 0.56 |
| Q3TDD9 | PPP1R21 | 88.337 | 0.59 | 0.64 | 0.62 | 0.03 | -0.07 | 0.18 | 0.05 | 0.18 | 0.56 |
| Q8JZX4 | RBM17 | 45.303 | 0.30 | 0.20 | 0.25 | 0.07 | -0.52 | -0.10 | -0.31 | 0.30 | 0.56 |
| P02468 | LAMC1 | 177.3 | -0.19 | 0.26 | 0.03 | 0.32 | -0.70 | -0.35 | -0.53 | 0.24 | 0.56 |
| Q9WUB3 | PYGM | 97.285 | -1.71 | -1.98 | -1.84 | 0.19 | -2.30 | -2.50 | -2.40 | 0.14 | 0.56 |
| Q9JKC8 | AP3M1 | 46.936 | 0.40 | 0.31 | 0.36 | 0.06 | -0.43 | 0.03 | -0.20 | 0.32 | 0.56 |
| P32067 | SSB | 47.756 | 0.38 | 0.34 | 0.36 | 0.03 | -0.31 | -0.09 | -0.20 | 0.16 | 0.56 |
| O88487 | DYNC1I2 | 68.393 | 0.30 | 0.29 | 0.29 | 0.01 | -0.36 | -0.17 | -0.26 | 0.13 | 0.56 |
| O55222 | ILK | 51.373 | 0.99 | 0.98 | 0.98 | 0.01 | 0.36 | 0.50 | 0.43 | 0.09 | 0.55 |

| P08030 | APRT | 19.724 | 0.67 | 0.60 | 0.63 | 0.05 | -0.03 | 0.19 | 0.08 | 0.15 | 0.55 |
| --- | --- | --- | --- | --- | --- | --- | --- | --- | --- | --- | --- |
| Q8BWN8 | ACOT4 | 46.48 | -0.02 | -0.18 | -0.10 | 0.11 | -0.57 | -0.73 | -0.65 | 0.11 | 0.55 |
| O35887 | CALU | 37.063 | 0.22 | 0.10 | 0.16 | 0.09 | -0.54 | -0.24 | -0.39 | 0.21 | 0.55 |
| Q9CX86 | HNRNPA0 | 30.53 | 0.57 | 0.36 | 0.47 | 0.15 | -0.05 | -0.12 | -0.08 | 0.05 | 0.55 |
| Q9DC23 | DNAJC10 | 90.582 | 0.08 | 0.22 | 0.15 | 0.10 | -0.43 | -0.37 | -0.40 | 0.05 | 0.55 |
| Q9WVL3 | SLC12A7 | 119.48 | 0.28 | 0.38 | 0.33 | 0.07 | -0.28 | -0.15 | -0.21 | 0.09 | 0.55 |
| Q6Q899 | DDX58 | 105.97 | -0.24 | 0.13 | -0.06 | 0.26 | -0.61 | -0.59 | -0.60 | 0.01 | 0.54 |
| Q9D020 | NT5C3A | 37.252 | 0.77 | 0.58 | 0.68 | 0.13 | 0.22 | 0.06 | 0.14 | 0.11 | 0.54 |
| O35326 | SRSF5 | 30.891 | 0.77 | 0.61 | 0.69 | 0.12 | 0.18 | 0.13 | 0.15 | 0.04 | 0.54 |
| Q9CY57 | CHTOP | 26.585 | 0.49 | 0.41 | 0.45 | 0.05 | -0.05 | -0.13 | -0.09 | 0.05 | 0.54 |
| Q6P9Q6 | FKBP15 | 132.96 | -0.57 | 0.45 | -0.06 | 0.72 | -1.16 | -0.04 | -0.60 | 0.79 | 0.54 |
| Q9JM76 | ARPC3 | 20.524 | 0.74 | 0.65 | 0.70 | 0.06 | 0.02 | 0.31 | 0.16 | 0.21 | 0.54 |
| P06745 | GPI | 62.766 | -1.11 | -1.01 | -1.06 | 0.08 | -1.71 | -1.47 | -1.59 | 0.17 | 0.53 |

| Q9Z211 | PEX11A | 28.153 | 1.29 | 1.25 | 1.27 | 0.03 | 0.66 | 0.82 | 0.74 | 0.11 | 0.53 |
| --- | --- | --- | --- | --- | --- | --- | --- | --- | --- | --- | --- |
| Q99NB9 | SF3B1 | 145.81 | 0.01 | 0.39 | 0.20 | 0.27 | -0.79 | 0.13 | -0.33 | 0.65 | 0.53 |
| Q9Z1N5 | DDX39B | 49.035 | 0.74 | 0.57 | 0.66 | 0.12 | 0.03 | 0.22 | 0.13 | 0.14 | 0.53 |
| Q60648 | GM2A | 20.824 | 0.28 | 0.19 | 0.23 | 0.06 | -0.37 | -0.23 | -0.30 | 0.10 | 0.53 |
| Q8CCS6 | PABPN1 | 32.296 | 0.60 | 0.59 | 0.60 | 0.01 | -0.02 | 0.15 | 0.07 | 0.12 | 0.53 |
| Q8R0W0 | EPPK1 | 723.3 | 1.46 | 1.86 | 1.66 | 0.29 | 1.06 | 1.21 | 1.13 | 0.11 | 0.53 |
|  |  | 54.073 | -0.64 | 0.99 | 0.17 | 1.15 | -1.02 | 0.32 | -0.35 | 0.95 | 0.53 |
| Q60590 | ORM1 | 23.895 | 1.93 | 2.08 | 2.00 | 0.11 | 1.33 | 1.62 | 1.48 | 0.20 | 0.53 |
| P54116 | STOM | 31.375 | 0.80 | 0.85 | 0.83 | 0.04 | 0.22 | 0.38 | 0.30 | 0.12 | 0.53 |
| Q60766 | IRGM1 | 46.551 | 0.11 | 0.20 | 0.15 | 0.06 | -0.32 | -0.42 | -0.37 | 0.06 | 0.52 |
| Q9D0L8 | RNMT | 53.291 | 0.96 | 0.57 | 0.77 | 0.28 | 0.25 | 0.24 | 0.24 | 0.01 | 0.52 |
| Q3TEA8 | HP1BP3 | 60.866 | 0.53 | 0.61 | 0.57 | 0.05 | -0.04 | 0.13 | 0.05 | 0.12 | 0.52 |
| Q8BKE6 | CYP20A1 | 52.149 | 0.98 | 0.45 | 0.72 | 0.37 | 0.36 | 0.03 | 0.19 | 0.24 | 0.52 |

| O35286 | DHX15 | 91.006 | 0.22 | 0.39 | 0.31 | 0.12 | -0.55 | 0.11 | -0.22 | 0.46 | 0.52 |
| --- | --- | --- | --- | --- | --- | --- | --- | --- | --- | --- | --- |
| P56593 | CYP2A12 | 56.179 | -0.36 | -0.50 | -0.43 | 0.10 | -1.03 | -0.86 | -0.95 | 0.13 | 0.52 |
| Q6URW6 | MYH14 | 228.58 | 0.59 | 0.03 | 0.31 | 0.39 | -0.11 | -0.28 | -0.19 | 0.12 | 0.51 |
| Q61510 | TRIM25 | 71.726 | 0.36 | 0.43 | 0.39 | 0.05 | -0.21 | -0.02 | -0.11 | 0.14 | 0.51 |
| Q01730 | RSU1 | 31.55 | 1.03 | 0.94 | 0.98 | 0.06 | 0.49 | 0.47 | 0.48 | 0.02 | 0.51 |
| P61759 | VBP1 | 22.435 | 0.00 | 0.01 | 0.00 | 0.01 | -0.63 | -0.37 | -0.50 | 0.18 | 0.51 |
| B2RXS4 | PLXNB2 | 206.23 | 0.74 | 0.88 | 0.81 | 0.10 | 0.26 | 0.35 | 0.31 | 0.06 | 0.51 |
| Q9D281 | FAM114A1 | 61.012 | -0.14 | -0.07 | -0.11 | 0.05 | -0.85 | -0.37 | -0.61 | 0.34 | 0.51 |
| P32921 | WARS | 54.357 | 0.31 | 0.15 | 0.23 | 0.11 | -0.29 | -0.25 | -0.27 | 0.03 | 0.50 |
| Q8BHD7 | PTBP3 | 56.7 | 0.35 | 0.64 | 0.50 | 0.21 | -0.24 | 0.22 | -0.01 | 0.33 | 0.50 |
| E9Q634 | MYO1E | 126.82 | 0.19 | 0.09 | 0.14 | 0.07 | -0.66 | -0.06 | -0.36 | 0.42 | 0.50 |
| O08677 | KNG1 | 73.101 | 0.60 | 0.81 | 0.70 | 0.15 | 0.09 | 0.30 | 0.20 | 0.15 | 0.50 |
| P46467 | VPS4B | 49.419 | 0.29 | 0.20 | 0.24 | 0.06 | -0.34 | -0.18 | -0.26 | 0.12 | 0.50 |

| O08788 | DCTN1 | 141.67 | -0.19 | 0.32 | 0.06 | 0.36 | -0.75 | -0.13 | -0.44 | 0.44 | 0.50 |
| --- | --- | --- | --- | --- | --- | --- | --- | --- | --- | --- | --- |
| P31230 | AIMP1 | 33.997 | 0.05 | 0.02 | 0.03 | 0.02 | -0.70 | -0.23 | -0.47 | 0.33 | 0.50 |
| P29699 | AHSG | 37.325 | -0.07 | -0.44 | -0.26 | 0.26 | -0.76 | -0.76 | -0.76 | 0.00 | 0.50 |
| Q8BGR9 | UBLCP1 | 36.836 | 0.61 | 0.55 | 0.58 | 0.04 | 0.12 | 0.04 | 0.08 | 0.06 | 0.50 |
| Q6ZQK5 | ACAP2 | 87.21 | 0.30 | 0.39 | 0.35 | 0.06 | -0.04 | -0.27 | -0.15 | 0.17 | 0.50 |
| Q62318 | TRIM28 | 88.846 | -0.11 | 0.33 | 0.11 | 0.31 | -0.70 | -0.09 | -0.39 | 0.43 | 0.50 |
| Q8K019 | BCLAF1 | 106 | 0.32 | 0.48 | 0.40 | 0.11 | -0.23 | 0.03 | -0.10 | 0.19 | 0.50 |
| Q9QUI0 | RHOA | 21.782 | 0.47 | 0.58 | 0.53 | 0.08 | -0.05 | 0.10 | 0.03 | 0.11 | 0.50 |
| Q9CQC6 | BZW1 | 48.043 | 0.28 | 0.15 | 0.21 | 0.09 | -0.44 | -0.13 | -0.28 | 0.22 | 0.50 |
| O55137 | ACOT1 | 46.135 | 0.60 | 0.47 | 0.54 | 0.09 | 0.12 | -0.04 | 0.04 | 0.11 | 0.50 |
| Q9JMA1 | USP14 | 56.001 | 0.22 | 0.22 | 0.22 | 0.00 | -0.34 | -0.21 | -0.28 | 0.09 | 0.50 |
| Q62383 | SUPT6H | 199.08 | 0.39 | 0.54 | 0.46 | 0.11 | -0.26 | 0.19 | -0.03 | 0.32 | 0.49 |
| Q78PG9 | CCDC25 | 24.48 | 0.04 | -0.06 | -0.01 | 0.07 | -0.66 | -0.35 | -0.50 | 0.22 | 0.49 |

| O88428 | PAPSS2 | 70.35 | -0.60 | -0.58 | -0.59 | 0.01 | -1.09 | -1.08 | -1.08 | 0.01 | 0.49 |
| --- | --- | --- | --- | --- | --- | --- | --- | --- | --- | --- | --- |
| Q9DBG5 | PLIN3 | 47.262 | -0.03 | 0.22 | 0.10 | 0.18 | -0.60 | -0.18 | -0.39 | 0.30 | 0.49 |
| P52792 | GCK | 52.088 | 0.76 | 0.89 | 0.82 | 0.09 | 0.23 | 0.43 | 0.33 | 0.14 | 0.49 |
| Q91ZW3 | SMARCA5 | 121.63 | 0.13 | 0.45 | 0.29 | 0.23 | -0.23 | -0.17 | -0.20 | 0.04 | 0.49 |
| O70503 | HSD17B12 | 34.741 | 0.03 | 0.07 | 0.05 | 0.03 | -0.50 | -0.37 | -0.44 | 0.09 | 0.49 |
| Q07456 | AMBP | 39.029 | 0.72 | 0.65 | 0.68 | 0.05 | 0.26 | 0.13 | 0.19 | 0.10 | 0.49 |
| Q76MZ3 | PPP2R1A | 65.322 | 0.18 | 0.16 | 0.17 | 0.01 | -0.44 | -0.20 | -0.32 | 0.17 | 0.49 |
| P51859 | HDGF | 26.268 | 0.41 | 0.21 | 0.31 | 0.14 | -0.35 | 0.00 | -0.18 | 0.25 | 0.48 |
| Q01339 | APOH | 38.618 | 0.50 | 0.21 | 0.35 | 0.21 | -0.22 | -0.04 | -0.13 | 0.13 | 0.48 |
| Q61703 | ITIH2 | 105.93 | 0.29 | 0.75 | 0.52 | 0.32 | -0.29 | 0.37 | 0.04 | 0.46 | 0.48 |
| P28654 | DCN | 39.809 | 1.05 | 1.41 | 1.23 | 0.26 | 0.71 | 0.79 | 0.75 | 0.05 | 0.48 |
| Q9Z0U1 | TJP2 | 131.28 | -0.63 | 0.06 | -0.28 | 0.49 | -1.26 | -0.28 | -0.77 | 0.69 | 0.48 |
| Q8C8U0 | PPFIBP1 | 108.54 | -0.50 | 0.22 | -0.14 | 0.51 | -1.40 | 0.16 | -0.62 | 1.10 | 0.48 |

| Q9DBE0 | CSAD | 55.144 | 2.47 | 2.40 | 2.43 | 0.05 | 1.92 | 1.98 | 1.95 | 0.04 | 0.48 |
| --- | --- | --- | --- | --- | --- | --- | --- | --- | --- | --- | --- |
| Q9D1J3 | SARNP | 23.532 | 0.40 | 0.25 | 0.33 | 0.10 | -0.24 | -0.07 | -0.15 | 0.12 | 0.48 |
| O70152 | DPM1 | 29.174 | -0.15 | 0.12 | -0.01 | 0.19 | -0.84 | -0.15 | -0.49 | 0.49 | 0.48 |
| Q99PG0 | AADAC | 45.25 | -0.43 | -0.45 | -0.44 | 0.01 | -1.06 | -0.78 | -0.92 | 0.20 | 0.48 |
| P13707 | GPD1 | 37.572 | -0.20 | -0.36 | -0.28 | 0.12 | -0.75 | -0.77 | -0.76 | 0.01 | 0.48 |
| Q3UN02 | LCLAT1 | 44.399 | 0.19 | 0.14 | 0.16 | 0.04 | -0.31 | -0.31 | -0.31 | 0.01 | 0.47 |
| Q8CCF0 | PRPF31 | 55.429 | 0.55 | 0.23 | 0.39 | 0.22 | -0.11 | -0.06 | -0.08 | 0.04 | 0.47 |
| Q8R151 | ZNFX1 | 218.83 | 0.02 | 0.42 | 0.22 | 0.29 | -0.34 | -0.16 | -0.25 | 0.13 | 0.47 |
| P40936 | INMT | 29.459 | -1.07 | -1.25 | -1.16 | 0.12 | -1.60 | -1.67 | -1.63 | 0.05 | 0.47 |
|  |  |  | 0.16 | 0.05 | 0.10 | 0.08 | -0.38 | -0.36 | -0.37 | 0.01 | 0.47 |
| P12265 | GUSB | 74.194 | 1.18 | 1.11 | 1.14 | 0.05 | 0.75 | 0.60 | 0.67 | 0.11 | 0.47 |
| Q8BGD9 | EIF4B | 68.839 | -0.01 | -0.06 | -0.03 | 0.03 | -0.78 | -0.23 | -0.50 | 0.39 | 0.47 |
| Q9CSU0 | RPRD1B | 36.883 | 0.12 | -0.14 | -0.01 | 0.18 | -0.58 | -0.37 | -0.48 | 0.15 | 0.47 |

| P51863 | ATP6V0D1 | 40.301 | 1.25 | 1.07 | 1.16 | 0.13 | 0.70 | 0.69 | 0.70 | 0.01 | 0.47 |
| --- | --- | --- | --- | --- | --- | --- | --- | --- | --- | --- | --- |
| Q923B6 | STEAP4 | 52.993 | 2.11 | 1.84 | 1.98 | 0.19 | 1.63 | 1.39 | 1.51 | 0.17 | 0.47 |
| Q9CQ10 | CHMP3 | 25.219 | 0.46 | 0.31 | 0.39 | 0.10 | -0.20 | 0.04 | -0.08 | 0.17 | 0.47 |
| Q9JHJ0 | TMOD3 | 39.502 | 0.61 | 0.64 | 0.63 | 0.02 | 0.16 | 0.17 | 0.16 | 0.01 | 0.46 |
| E9Q555 | RNF213 | 584.49 | -0.10 | 0.30 | 0.10 | 0.28 | 0.22 | -0.96 | -0.37 | 0.83 | 0.46 |
| Q7TNP2 | PPP2R1B | 65.934 | 0.87 | 0.62 | 0.74 | 0.17 | 0.28 | 0.28 | 0.28 | 0.00 | 0.46 |
| P41233 | ABCA1 | 253.91 | 0.41 | 0.22 | 0.32 | 0.13 | -0.11 | -0.18 | -0.15 | 0.05 | 0.46 |
| Q4PZA2 | ECE1 | 87.072 | -0.13 | -0.50 | -0.31 | 0.26 | -0.89 | -0.66 | -0.78 | 0.16 | 0.46 |
| Q6A0A9 | FAM120A | 121.64 | -0.64 | 0.21 | -0.22 | 0.60 | -1.25 | -0.10 | -0.68 | 0.81 | 0.46 |
| P97429 | ANXA4 | 35.915 | 0.45 | 0.16 | 0.30 | 0.21 | -0.10 | -0.21 | -0.16 | 0.08 | 0.46 |
| Q9CZ44 | NSFL1C | 40.709 | -0.15 | -0.36 | -0.25 | 0.14 | -0.92 | -0.51 | -0.71 | 0.29 | 0.46 |
| Q9CYR6 | PGM3 | 59.452 | -0.17 | -0.17 | -0.17 | 0.00 | -0.62 | -0.64 | -0.63 | 0.01 | 0.46 |
| Q9R008 | MVK | 41.877 | 0.53 | 0.37 | 0.45 | 0.11 | 0.03 | -0.05 | -0.01 | 0.06 | 0.46 |

| Q9D0F3 | LMAN1 | 57.788 | -0.25 | -0.42 | -0.33 | 0.12 | -0.82 | -0.75 | -0.79 | 0.05 | 0.45 |
| --- | --- | --- | --- | --- | --- | --- | --- | --- | --- | --- | --- |
| Q6KAR6 | EXOC3 | 86.454 | 0.57 | 0.54 | 0.56 | 0.02 | 0.05 | 0.16 | 0.10 | 0.07 | 0.45 |
| P97930 | DTYMK | 23.914 | 0.23 | 0.28 | 0.26 | 0.04 | -0.23 | -0.16 | -0.20 | 0.05 | 0.45 |
| Q8VCH0 | ACAA1B | 43.995 | 0.28 | 0.00 | 0.14 | 0.19 | -0.31 | -0.32 | -0.31 | 0.01 | 0.45 |
| Q61147 | CP | 121.15 | 1.11 | 1.64 | 1.37 | 0.38 | 0.81 | 1.04 | 0.92 | 0.16 | 0.45 |
| Q9CU62 | SMC1A | 143.23 | 0.10 | 0.27 | 0.18 | 0.12 | -0.60 | 0.07 | -0.27 | 0.48 | 0.45 |
| Q7TPV4 | MYBBP1A | 152.04 | -0.01 | 0.16 | 0.08 | 0.13 | -0.51 | -0.24 | -0.37 | 0.19 | 0.45 |
| Q61879 | MYH10 | 228.99 | 1.02 | 1.46 | 1.24 | 0.31 | 0.72 | 0.86 | 0.79 | 0.10 | 0.45 |
| Q922E4 | PCYT2 | 45.234 | -0.05 | -0.16 | -0.11 | 0.08 | -0.59 | -0.52 | -0.55 | 0.04 | 0.45 |
| Q9QXZ0 | MACF1 | 831.87 | 0.45 | 0.65 | 0.55 | 0.15 | -0.06 | 0.26 | 0.10 | 0.23 | 0.45 |
| P00329 | ADH1 | 39.771 | -0.52 | -0.63 | -0.57 | 0.08 | -0.97 | -1.08 | -1.02 | 0.08 | 0.45 |
| P54103 | DNAJC2 | 71.721 | 0.44 | 0.46 | 0.45 | 0.01 | 0.02 | 0.00 | 0.01 | 0.02 | 0.44 |
| O35226 | PSMD4 | 40.703 | -0.33 | -0.11 | -0.22 | 0.16 | -1.05 | -0.29 | -0.67 | 0.54 | 0.44 |

| A2AN08 | UBR4 | 572.28 | 0.13 | 0.25 | 0.19 | 0.09 | -0.40 | -0.11 | -0.26 | 0.21 | 0.44 |
| --- | --- | --- | --- | --- | --- | --- | --- | --- | --- | --- | --- |
| Q569Z6 | THRAP3 | 108.18 | -0.09 | 0.24 | 0.07 | 0.23 | -0.62 | -0.12 | -0.37 | 0.36 | 0.44 |
| Q9CWZ7 | NAPG | 34.732 | 0.52 | 0.51 | 0.51 | 0.01 | 0.10 | 0.04 | 0.07 | 0.04 | 0.44 |
| Q61207 | PSAP | 61.422 | 0.94 | 1.05 | 1.00 | 0.07 | 0.63 | 0.48 | 0.56 | 0.10 | 0.44 |
| Q64191 | AGA | 37.022 | 0.80 | 0.68 | 0.74 | 0.08 | 0.35 | 0.25 | 0.30 | 0.07 | 0.44 |
| Q920L1 | FADS1 | 52.322 | -0.96 | -1.01 | -0.99 | 0.04 | -1.65 | -1.20 | -1.43 | 0.32 | 0.44 |
| Q9DBR1 | XRN2 | 108.69 | 0.22 | 0.70 | 0.46 | 0.34 | -0.18 | 0.22 | 0.02 | 0.28 | 0.44 |
| P18242 | CTSD | 44.953 | 1.27 | 1.33 | 1.30 | 0.04 | 0.84 | 0.88 | 0.86 | 0.02 | 0.44 |
| Q3UM45 | PPP1R7 | 41.291 | 0.19 | 0.02 | 0.10 | 0.12 | -0.39 | -0.28 | -0.33 | 0.08 | 0.44 |
| O08663 | METAP2 | 52.921 | 0.17 | 0.09 | 0.13 | 0.06 | -0.38 | -0.24 | -0.31 | 0.10 | 0.44 |
| Q6PD03 | PPP2R5A | 56.346 | -0.07 | -0.10 | -0.08 | 0.02 | -0.61 | -0.43 | -0.52 | 0.12 | 0.44 |
| Q7TPR4 | ACTN1 | 103.07 | 0.54 | 0.57 | 0.56 | 0.02 | 0.20 | 0.05 | 0.12 | 0.11 | 0.44 |
| P47856 | GFPT1 | 78.538 | 0.03 | -0.10 | -0.04 | 0.09 | -0.52 | -0.43 | -0.47 | 0.07 | 0.44 |

| O54941 | SMARCE1 | 46.638 | 0.21 | 0.41 | 0.31 | 0.14 | -0.32 | 0.07 | -0.12 | 0.27 | 0.44 |
| --- | --- | --- | --- | --- | --- | --- | --- | --- | --- | --- | --- |
| P10649 | GSTM1 | 25.97 | 1.10 | 1.12 | 1.11 | 0.01 | 0.62 | 0.73 | 0.68 | 0.08 | 0.44 |
| Q6NZB0 | DNAJC8 | 29.812 | 0.12 | 0.14 | 0.13 | 0.02 | -0.36 | -0.26 | -0.31 | 0.07 | 0.44 |
| P62960 | YBX1 | 35.73 | 0.15 | -0.01 | 0.07 | 0.11 | -0.46 | -0.27 | -0.36 | 0.14 | 0.43 |
| P09405 | NCL | 76.722 | 0.49 | 0.44 | 0.47 | 0.04 | 0.01 | 0.06 | 0.04 | 0.03 | 0.43 |
| P46460 | NSF | 82.613 | 0.62 | 0.50 | 0.56 | 0.09 | 0.16 | 0.09 | 0.12 | 0.05 | 0.43 |
| P21614 | GC | 53.6 | 0.06 | 0.09 | 0.08 | 0.02 | -0.44 | -0.27 | -0.35 | 0.11 | 0.43 |
| Q64516 | GK | 61.227 | -0.33 | -0.25 | -0.29 | 0.06 | -0.82 | -0.62 | -0.72 | 0.14 | 0.43 |
| P45377 | AKR1B8 | 36.12 | 0.78 | 0.69 | 0.73 | 0.06 | 0.30 | 0.30 | 0.30 | 0.00 | 0.43 |
| P07724 | ALB | 68.692 | 0.21 | 0.19 | 0.20 | 0.02 | -0.18 | -0.28 | -0.23 | 0.07 | 0.43 |
| P63085 | MAPK1 | 41.275 | 0.29 | 0.25 | 0.27 | 0.03 | -0.19 | -0.13 | -0.16 | 0.04 | 0.43 |
| Q9CXY6 | ILF2 | 43.062 | 0.56 | 0.40 | 0.48 | 0.12 | 0.05 | 0.05 | 0.05 | 0.00 | 0.43 |
| P23506 | PCMT1 | 24.634 | 0.05 | 0.05 | 0.05 | 0.00 | -0.47 | -0.29 | -0.38 | 0.13 | 0.43 |

| Q9CQW1 | YKT6 | 22.314 | 0.18 | 0.11 | 0.14 | 0.05 | -0.32 | -0.25 | -0.28 | 0.05 | 0.43 |
| --- | --- | --- | --- | --- | --- | --- | --- | --- | --- | --- | --- |
| Q9WV54 | ASAH1 | 44.669 | 1.64 | 1.59 | 1.61 | 0.04 | 1.23 | 1.14 | 1.19 | 0.07 | 0.43 |
| Q9D2V7 | CORO7 | 100.81 | 0.68 | 0.87 | 0.77 | 0.13 | 0.12 | 0.58 | 0.35 | 0.33 | 0.43 |
| Q8K5B2 | MCFD2 | 16.168 | -0.29 | -0.44 | -0.37 | 0.10 | -0.76 | -0.82 | -0.79 | 0.04 | 0.43 |
| Q8BHL8 | PSMF1 | 29.664 | 0.03 | -0.04 | 0.00 | 0.05 | -0.57 | -0.29 | -0.43 | 0.20 | 0.43 |
| O70325 | GPX4 | 22.228 | 0.81 | 0.92 | 0.86 | 0.08 | 0.31 | 0.57 | 0.44 | 0.19 | 0.43 |
| Q8CJG0 | AGO2 | 97.303 | 0.57 | 0.30 | 0.43 | 0.19 | 0.14 | -0.12 | 0.01 | 0.19 | 0.42 |
| P97379 | G3BP2 | 54.087 | -0.07 | 0.19 | 0.06 | 0.18 | -0.44 | -0.29 | -0.37 | 0.11 | 0.42 |
| P70333 | HNRNPH2 | 49.279 | 0.52 | 0.66 | 0.59 | 0.10 | -0.29 | 0.62 | 0.17 | 0.65 | 0.42 |
| Q91YD9 | WASL | 54.274 | -0.20 | -0.06 | -0.13 | 0.10 | -0.72 | -0.39 | -0.55 | 0.23 | 0.42 |
| Q62448 | EIF4G2 | 102.1 | -0.39 | 0.12 | -0.14 | 0.36 | -1.08 | -0.03 | -0.56 | 0.75 | 0.42 |
| Q58A65 | SPAG9 | 146.22 | -0.15 | 0.31 | 0.08 | 0.33 | -0.55 | -0.13 | -0.34 | 0.29 | 0.42 |
| Q8VCH8 | UBXN4 | 56.471 | -0.10 | -0.12 | -0.11 | 0.01 | -0.63 | -0.42 | -0.53 | 0.15 | 0.42 |

| P62806 | HIST1H4A | 11.367 | 0.43 | 0.49 | 0.46 | 0.04 | 0.06 | 0.02 | 0.04 | 0.03 | 0.42 |
| --- | --- | --- | --- | --- | --- | --- | --- | --- | --- | --- | --- |
| Q8K4Z5 | SF3A1 | 88.544 | -0.23 | 0.35 | 0.06 | 0.41 | -0.75 | 0.03 | -0.36 | 0.55 | 0.42 |
| Q9D273 | MMAB | 26.273 | 0.06 | 0.18 | 0.12 | 0.09 | -0.52 | -0.06 | -0.29 | 0.33 | 0.42 |
| Q9JI90 | RNF14 | 54.926 | -0.09 | -0.04 | -0.07 | 0.04 | -0.52 | -0.44 | -0.48 | 0.05 | 0.42 |
| O54984 | ASNA1 | 38.822 | 0.32 | 0.13 | 0.22 | 0.14 | -0.20 | -0.18 | -0.19 | 0.01 | 0.41 |
| Q08943 | SSRP1 | 80.859 | 0.51 | 0.21 | 0.36 | 0.22 | -0.04 | -0.07 | -0.05 | 0.02 | 0.41 |
| Q922B2 | DARS | 57.147 | -0.12 | -0.04 | -0.08 | 0.06 | -0.61 | -0.38 | -0.49 | 0.16 | 0.41 |
| P52332 | JAK1 | 133.37 | 0.24 | 0.60 | 0.42 | 0.25 | -0.04 | 0.05 | 0.00 | 0.06 | 0.41 |
| Q9EPC1 | PARVA | 42.329 | 0.98 | 0.66 | 0.82 | 0.22 | 0.35 | 0.46 | 0.41 | 0.08 | 0.41 |
| Q3UYV9 | NCBP1 | 91.926 | 0.44 | 0.29 | 0.37 | 0.11 | -0.14 | 0.05 | -0.05 | 0.13 | 0.41 |
| Q8R3N6 | THOC1 | 75.435 | 0.23 | 0.25 | 0.24 | 0.01 | -0.05 | -0.29 | -0.17 | 0.17 | 0.41 |
| Q80VD1 | FAM98B | 45.349 | 0.44 | 0.28 | 0.36 | 0.12 | -0.21 | 0.11 | -0.05 | 0.23 | 0.41 |
| Q7TMB8 | CYFIP1 | 145.24 | 0.56 | 0.92 | 0.74 | 0.25 | 0.35 | 0.32 | 0.33 | 0.02 | 0.41 |

| P55302 | LRPAP1 | 42.215 | 0.20 | 0.02 | 0.11 | 0.12 | -0.35 | -0.25 | -0.30 | 0.07 | 0.41 |
| --- | --- | --- | --- | --- | --- | --- | --- | --- | --- | --- | --- |
| P49442 | INPP1 | 43.346 | -0.31 | -0.48 | -0.40 | 0.11 | -0.88 | -0.72 | -0.80 | 0.11 | 0.41 |
| P54775 | PSMC4 | 47.408 | 0.01 | -0.12 | -0.05 | 0.09 | -0.56 | -0.36 | -0.46 | 0.14 | 0.41 |
| P26369 | U2AF2 | 53.516 | -0.17 | 0.25 | 0.04 | 0.30 | -0.69 | -0.04 | -0.37 | 0.46 | 0.41 |
| O88792 | F11R | 32.423 | 0.92 | 0.55 | 0.74 | 0.26 | 0.28 | 0.39 | 0.33 | 0.08 | 0.41 |
| O70451 | SLC16A7 | 52.603 | 0.08 | -0.19 | -0.06 | 0.19 | -0.71 | -0.22 | -0.47 | 0.35 | 0.41 |
| Q9D0M0 | EXOSC7 | 31.825 | 0.60 | 0.17 | 0.38 | 0.31 | -0.10 | 0.06 | -0.02 | 0.12 | 0.41 |
| P10605 | CTSB | 37.279 | 1.10 | 1.14 | 1.12 | 0.02 | 0.70 | 0.73 | 0.72 | 0.02 | 0.40 |
| O54962 | BANF1 | 10.102 | 0.15 | 0.12 | 0.14 | 0.02 | -0.17 | -0.36 | -0.27 | 0.13 | 0.40 |
| P12382 | PFKL | 85.359 | 0.18 | 0.04 | 0.11 | 0.10 | -0.30 | -0.29 | -0.29 | 0.00 | 0.40 |
| Q9WUP7 | UCHL5 | 37.616 | 0.07 | -0.04 | 0.01 | 0.08 | -0.40 | -0.39 | -0.39 | 0.01 | 0.40 |
| Q71RI9 | CCBL2 | 51.126 | -0.57 | -0.60 | -0.59 | 0.02 | -1.00 | -0.97 | -0.99 | 0.02 | 0.40 |
| P42932 | CCT8 | 59.555 | -0.34 | -0.37 | -0.36 | 0.02 | -0.90 | -0.62 | -0.76 | 0.20 | 0.40 |

| Q8BG05 | HNRNPA3 | 39.652 | 0.54 | 0.39 | 0.46 | 0.11 | 0.04 | 0.09 | 0.06 | 0.04 | 0.40 |
| --- | --- | --- | --- | --- | --- | --- | --- | --- | --- | --- | --- |
| Q61598 | GDI2 | 50.537 | 0.42 | 0.19 | 0.31 | 0.16 | -0.05 | -0.13 | -0.09 | 0.06 | 0.40 |
| Q8BSY0 | ASPH | 83.041 | -0.15 | 0.13 | -0.01 | 0.20 | -0.64 | -0.18 | -0.41 | 0.32 | 0.40 |
| P70168 | KPNB1 | 97.183 | 0.26 | 0.18 | 0.22 | 0.05 | -0.14 | -0.22 | -0.18 | 0.06 | 0.40 |
| P08226 | APOE | 35.866 | 0.02 | -0.15 | -0.07 | 0.12 | -0.49 | -0.44 | -0.47 | 0.04 | 0.40 |
| Q9D287 | BCAS2 | 26.131 | 0.68 | 0.40 | 0.54 | 0.20 | 0.21 | 0.07 | 0.14 | 0.10 | 0.40 |
| Q9JHU4 | DYNC1H1 | 532.04 | 0.10 | 0.42 | 0.26 | 0.22 | -0.24 | -0.04 | -0.14 | 0.14 | 0.40 |
| Q99JF8 | PSIP1 | 59.696 | 0.48 | 0.18 | 0.33 | 0.21 | -0.18 | 0.04 | -0.07 | 0.15 | 0.40 |
| Q9QXE7 |  |  | 0.83 | 0.29 | 0.56 | 0.38 | 0.20 | 0.13 | 0.17 | 0.05 | 0.40 |
| O88736 | HSD17B7 | 37.316 | 0.49 | 0.45 | 0.47 | 0.03 | 0.01 | 0.14 | 0.08 | 0.09 | 0.40 |
| Q8C0M9 | ASRGL1 | 33.95 | 0.03 | 0.43 | 0.23 | 0.29 | -0.33 | 0.00 | -0.17 | 0.23 | 0.40 |
| Q64471 | GSTT1 | 27.374 | -0.32 | -0.27 | -0.30 | 0.04 | -0.75 | -0.64 | -0.69 | 0.08 | 0.40 |
| P62821 | RAB1A | 22.677 | 0.20 | 0.20 | 0.20 | 0.00 | -0.24 | -0.15 | -0.20 | 0.06 | 0.39 |

| Q9JIK5 | DDX21 | 93.55 | 0.24 | 0.48 | 0.36 | 0.17 | -0.10 | 0.04 | -0.03 | 0.09 | 0.39 |
| --- | --- | --- | --- | --- | --- | --- | --- | --- | --- | --- | --- |
| P63005 | PAFAH1B1 | 46.67 | 0.47 | 0.28 | 0.37 | 0.13 | -0.04 | 0.00 | -0.02 | 0.03 | 0.39 |
| O35841 | API5 | 56.784 | 0.64 | 0.41 | 0.52 | 0.17 | 0.17 | 0.09 | 0.13 | 0.05 | 0.39 |
| Q9Z2U1 | PSMA5 | 26.411 | 0.13 | -0.06 | 0.04 | 0.13 | -0.37 | -0.34 | -0.35 | 0.02 | 0.39 |
| Q5XG73 | ACBD5 | 56.613 | -0.17 | 0.06 | -0.05 | 0.17 | -0.81 | -0.08 | -0.45 | 0.52 | 0.39 |
| Q06890 | CLU | 51.655 | 0.82 | 1.85 | 1.33 | 0.73 | 0.91 | 0.97 | 0.94 | 0.04 | 0.39 |
| P28352 | APEX1 | 35.49 | 0.62 | 0.43 | 0.52 | 0.14 | 0.15 | 0.12 | 0.13 | 0.02 | 0.39 |
| Q8CCB4 | VPS53 | 94.422 | 0.70 | 0.48 | 0.59 | 0.16 | 0.31 | 0.09 | 0.20 | 0.15 | 0.39 |
| Q8BJU0 | SGTA | 34.322 | -0.31 | -0.34 | -0.32 | 0.02 | -0.81 | -0.61 | -0.71 | 0.14 | 0.39 |
| Q61390 | CCT6B | 58.184 | -0.29 | -0.36 | -0.32 | 0.04 | -0.82 | -0.60 | -0.71 | 0.16 | 0.39 |
| Q9CQT1 | MRI1 | 39.41 | 0.21 | 0.13 | 0.17 | 0.06 | -0.22 | -0.20 | -0.21 | 0.01 | 0.38 |
| Q8JZR0 | ACSL5 | 76.205 | -0.73 | -0.75 | -0.74 | 0.02 | -1.01 | -1.24 | -1.12 | 0.16 | 0.38 |
| P09411 | PGK1 | 44.55 | 0.00 | -0.07 | -0.04 | 0.05 | -0.43 | -0.40 | -0.42 | 0.02 | 0.38 |

| Q9CQV8 | YWHAB | 28.086 | 0.44 | 0.40 | 0.42 | 0.02 | 0.01 | 0.06 | 0.04 | 0.04 | 0.38 |
| --- | --- | --- | --- | --- | --- | --- | --- | --- | --- | --- | --- |
| Q99KP6 | PRPF19 | 55.238 | 0.63 | 0.47 | 0.55 | 0.11 | 0.12 | 0.21 | 0.16 | 0.06 | 0.38 |
| Q80X90 | FLNB | 277.82 | 0.07 | 0.36 | 0.21 | 0.21 | -0.27 | -0.07 | -0.17 | 0.14 | 0.38 |
| P24547 | IMPDH2 | 55.814 | -0.35 | -0.35 | -0.35 | 0.00 | -0.88 | -0.59 | -0.73 | 0.21 | 0.38 |
| P17182 | ENO1 | 47.14 | -0.16 | -0.42 | -0.29 | 0.19 | -0.65 | -0.70 | -0.67 | 0.04 | 0.38 |
| P70122 | SBDS | 28.78 | 0.43 | 0.42 | 0.42 | 0.01 | 0.08 | 0.00 | 0.04 | 0.06 | 0.38 |
| Q9D8T2 | GSDMDC1 | 53.237 | 0.48 | 0.41 | 0.44 | 0.05 | -0.08 | 0.21 | 0.06 | 0.20 | 0.38 |
| P61290 | PSME3 | 29.506 | 0.43 | 0.40 | 0.41 | 0.02 | 0.04 | 0.03 | 0.03 | 0.01 | 0.38 |
| Q61656 | DDX5 | 69.289 | 0.26 | 0.56 | 0.41 | 0.21 | -0.15 | 0.20 | 0.03 | 0.25 | 0.38 |
| Q3UL36 | ARGLU1 | 32.887 | 0.19 | -0.08 | 0.05 | 0.19 | -0.31 | -0.35 | -0.33 | 0.03 | 0.38 |
| Q8CDN6 | TXNL1 | 32.237 | 0.26 | 0.06 | 0.16 | 0.14 | -0.15 | -0.30 | -0.22 | 0.11 | 0.38 |
| Q8CHP8 | PGP | 34.54 | 0.21 | 0.08 | 0.15 | 0.09 | -0.24 | -0.23 | -0.23 | 0.01 | 0.38 |
| Q3U2P1 | SEC24A | 118.78 | -0.23 | 0.04 | -0.09 | 0.19 | -0.99 | 0.04 | -0.47 | 0.73 | 0.38 |

| Q8BKC5 | IPO5 | 123.59 | 0.00 | 0.16 | 0.08 | 0.11 | -0.24 | -0.37 | -0.30 | 0.09 | 0.38 |
| --- | --- | --- | --- | --- | --- | --- | --- | --- | --- | --- | --- |
| P54071 | IDH2 | 50.906 | 0.07 | -0.01 | 0.03 | 0.05 | -0.43 | -0.27 | -0.35 | 0.11 | 0.38 |
| Q9CQU0 | TXNDC12 | 19.048 | 0.09 | 0.01 | 0.05 | 0.05 | -0.25 | -0.41 | -0.33 | 0.11 | 0.38 |
| O35345 | KPNA6 | 59.964 | 0.13 | -0.02 | 0.06 | 0.11 | -0.47 | -0.16 | -0.32 | 0.22 | 0.38 |
| Q9D8C4 | IFI35 | 31.875 | 0.16 | 0.10 | 0.13 | 0.05 | -0.20 | -0.29 | -0.25 | 0.07 | 0.38 |
| Q9Z2U0 | PSMA7 | 27.855 | 0.07 | -0.06 | 0.01 | 0.09 | -0.38 | -0.35 | -0.37 | 0.02 | 0.38 |
| P62869 | TCEB2 | 13.17 | 0.32 | 0.11 | 0.22 | 0.15 | -0.20 | -0.11 | -0.16 | 0.06 | 0.38 |
| Q6A068 | CDC5L | 92.188 | 0.31 | 0.39 | 0.35 | 0.05 | -0.16 | 0.11 | -0.02 | 0.19 | 0.37 |
| Q64378 | FKBP5 | 50.966 | 0.96 | 1.04 | 1.00 | 0.06 | 0.49 | 0.76 | 0.63 | 0.19 | 0.37 |
| O09044 | SNAP23 | 23.261 | 0.12 | 0.35 | 0.23 | 0.16 | -0.26 | -0.02 | -0.14 | 0.17 | 0.37 |
| Q9QUM9 | PSMA6 | 27.372 | 0.00 | 0.00 | 0.00 | 0.00 | -0.43 | -0.32 | -0.37 | 0.07 | 0.37 |
| Q9QXB9 | DRG2 | 40.718 | 0.13 | 0.08 | 0.10 | 0.03 | -0.28 | -0.26 | -0.27 | 0.02 | 0.37 |
| Q9DB25 | ALG5 | 36.79 | 0.49 | 0.28 | 0.39 | 0.15 | 0.08 | -0.05 | 0.02 | 0.09 | 0.37 |

| Q9D6Y9 | GBE1 | 80.363 | -0.07 | -0.05 | -0.06 | 0.01 | -0.34 | -0.52 | -0.43 | 0.13 | 0.37 |
| --- | --- | --- | --- | --- | --- | --- | --- | --- | --- | --- | --- |
| Q60865 | CAPRIN1 | 78.168 | -0.79 | -0.14 | -0.47 | 0.46 | -1.44 | -0.24 | -0.84 | 0.85 | 0.37 |
| O35945 | ALDH1A7 | 54.587 | 1.19 | 1.24 | 1.22 | 0.03 | 0.76 | 0.93 | 0.85 | 0.12 | 0.37 |
| P08101 |  | 36.695 | 1.08 | 1.52 | 1.30 | 0.31 | 0.85 | 1.01 | 0.93 | 0.11 | 0.37 |
| P36993 | PPM1B | 42.795 | -0.09 | -0.21 | -0.15 | 0.08 | -0.57 | -0.47 | -0.52 | 0.07 | 0.37 |
| P23953 | CES1C | 61.055 | -0.30 | -0.23 | -0.26 | 0.04 | -0.90 | -0.36 | -0.63 | 0.38 | 0.37 |
| Q9D7N9 | APMAP | 46.434 | 0.22 | 0.05 | 0.13 | 0.12 | -0.37 | -0.10 | -0.23 | 0.19 | 0.37 |
|  |  |  | -0.10 | 0.20 | 0.05 | 0.21 | -0.72 | 0.08 | -0.32 | 0.56 | 0.37 |
| Q924M7 | MPI | 46.575 | 0.37 | 0.16 | 0.27 | 0.15 | -0.17 | -0.03 | -0.10 | 0.10 | 0.37 |
| P56959 | FUS | 52.673 | 0.38 | 0.49 | 0.44 | 0.08 | 0.20 | -0.07 | 0.07 | 0.19 | 0.37 |
| Q9CY58 | SERBP1 | 44.714 | -0.05 | -0.15 | -0.10 | 0.07 | -0.65 | -0.29 | -0.47 | 0.25 | 0.37 |
| P11983 | TCP1 | 60.448 | -0.29 | -0.34 | -0.31 | 0.04 | -0.81 | -0.55 | -0.68 | 0.18 | 0.37 |
| Q3TIX9 | USP39 | 65.146 | 0.25 | 0.27 | 0.26 | 0.02 | -0.26 | 0.04 | -0.11 | 0.22 | 0.37 |

| Q7M6Y3 | PICALM | 71.543 | 0.32 | 0.50 | 0.41 | 0.13 | -0.10 | 0.19 | 0.04 | 0.20 | 0.37 |
| --- | --- | --- | --- | --- | --- | --- | --- | --- | --- | --- | --- |
| Q06770 | SERPINA6 | 44.769 | -0.64 | -0.97 | -0.81 | 0.23 | -0.90 | -1.45 | -1.17 | 0.39 | 0.36 |
| Q9DCT8 | CRIP2 | 22.727 | 0.05 | 0.11 | 0.08 | 0.05 | -0.41 | -0.16 | -0.28 | 0.17 | 0.36 |
|  |  |  | 0.47 | 0.48 | 0.48 | 0.01 | 0.11 | 0.12 | 0.11 | 0.01 | 0.36 |
| P50396 | GDI1 | 50.521 | 0.25 | 0.23 | 0.24 | 0.02 | -0.14 | -0.10 | -0.12 | 0.03 | 0.36 |
| Q9D1G1 | RAB1B | 22.187 | 0.09 | 0.04 | 0.07 | 0.03 | -0.36 | -0.23 | -0.30 | 0.09 | 0.36 |
| P17225 | PTBP1 | 56.477 | 0.26 | 0.40 | 0.33 | 0.10 | -0.20 | 0.13 | -0.03 | 0.23 | 0.36 |
| Q8VCM7 | FGG | 49.391 | 0.74 | 0.95 | 0.84 | 0.15 | 0.37 | 0.59 | 0.48 | 0.16 | 0.36 |
| Q8CGF7 | TCERG1 | 123.79 | -0.75 | 0.39 | -0.18 | 0.81 | -1.26 | 0.17 | -0.54 | 1.01 | 0.36 |
| Q9JKV1 | ADRM1 | 42.06 | -0.14 | -0.25 | -0.20 | 0.08 | -0.74 | -0.37 | -0.56 | 0.26 | 0.36 |
| Q99020 | HNRNPAB | 30.831 | 0.38 | 0.31 | 0.34 | 0.05 | -0.01 | -0.02 | -0.02 | 0.01 | 0.36 |
| Q61655 | DDX19A | 53.932 | 0.07 | 0.00 | 0.04 | 0.06 | -0.45 | -0.20 | -0.33 | 0.18 | 0.36 |
| Q9R1P3 | PSMB2 | 22.906 | -0.10 | -0.08 | -0.09 | 0.01 | -0.47 | -0.43 | -0.45 | 0.03 | 0.36 |

| Q9CWJ9 | ATIC | 64.217 | 0.14 | 0.09 | 0.12 | 0.03 | -0.18 | -0.30 | -0.24 | 0.08 | 0.36 |
| --- | --- | --- | --- | --- | --- | --- | --- | --- | --- | --- | --- |
| Q7TQI3 | OTUB1 | 31.27 | 0.22 | 0.21 | 0.22 | 0.01 | -0.11 | -0.17 | -0.14 | 0.04 | 0.36 |
| Q9WUM3 | CORO1B | 53.912 | 0.49 | 0.82 | 0.66 | 0.24 | 0.38 | 0.22 | 0.30 | 0.11 | 0.36 |
| P35235 | PTPN11 | 68.46 | 0.18 | 0.20 | 0.19 | 0.02 | -0.22 | -0.11 | -0.17 | 0.08 | 0.36 |
| O35593 | PSMD14 | 34.577 | -0.09 | -0.07 | -0.08 | 0.02 | -0.49 | -0.38 | -0.43 | 0.08 | 0.36 |
| Q8BU33 | ILVBL | 68.155 | -0.01 | 0.19 | 0.09 | 0.14 | -0.33 | -0.19 | -0.26 | 0.10 | 0.35 |
| P57716 | NCSTN | 78.491 | 0.59 | 0.47 | 0.53 | 0.09 | 0.28 | 0.06 | 0.17 | 0.15 | 0.35 |
| Q8CH25 | SLTM | 116.92 | 0.24 | 0.39 | 0.32 | 0.11 | -0.13 | 0.06 | -0.04 | 0.14 | 0.35 |
| Q80SW1 | AHCYL1 | 58.951 | 0.18 | 0.28 | 0.23 | 0.07 | -0.27 | 0.02 | -0.13 | 0.21 | 0.35 |
| Q8C0L8 | COG5 | 91.39 | 0.56 | 0.39 | 0.47 | 0.12 | 0.07 | 0.17 | 0.12 | 0.07 | 0.35 |
| Q6PDQ2 | CHD4 | 217.75 | 0.26 | 0.49 | 0.38 | 0.16 | -0.11 | 0.16 | 0.03 | 0.19 | 0.35 |
| Q61508 | ECM1 | 62.832 | 0.90 | 0.57 | 0.74 | 0.23 | 0.43 | 0.34 | 0.39 | 0.07 | 0.35 |
| Q921H8 | ACAA1A | 43.953 | 0.68 | 0.18 | 0.43 | 0.36 | 0.39 | -0.23 | 0.08 | 0.43 | 0.35 |

| Q8R180 | ERO1L | 54.084 | 0.23 | 0.12 | 0.18 | 0.08 | -0.14 | -0.21 | -0.17 | 0.05 | 0.35 |
| --- | --- | --- | --- | --- | --- | --- | --- | --- | --- | --- | --- |
| Q61990 | PCBP2 | 38.221 | 0.23 | 0.24 | 0.23 | 0.01 | -0.15 | -0.08 | -0.12 | 0.05 | 0.35 |
| P49935 | CTSH | 37.17 | 1.03 | 1.01 | 1.02 | 0.02 | 0.61 | 0.73 | 0.67 | 0.08 | 0.35 |
| Q8VCF0 | MAVS | 53.398 | -0.66 | -0.41 | -0.53 | 0.18 | -1.01 | -0.74 | -0.88 | 0.19 | 0.35 |
| Q8K0E8 | FGB | 54.752 | 1.07 | 1.15 | 1.11 | 0.06 | 0.63 | 0.91 | 0.77 | 0.20 | 0.35 |
| P47934 | CRAT | 70.839 | 0.22 | 0.24 | 0.23 | 0.02 | -0.12 | -0.11 | -0.12 | 0.01 | 0.35 |
| Q7TNC4 | LUC7L2 | 46.582 | 0.68 | 0.28 | 0.48 | 0.28 | 0.26 | 0.01 | 0.14 | 0.18 | 0.35 |
| P09055 | ITGB1 | 88.231 | 0.18 | 0.70 | 0.44 | 0.37 | -0.06 | 0.25 | 0.10 | 0.22 | 0.35 |
| Q99M28 | RNPS1 | 34.208 | 0.25 | -0.01 | 0.12 | 0.18 | -0.22 | -0.23 | -0.22 | 0.01 | 0.35 |
| Q03311 | BCHE | 68.461 | -0.10 | -0.24 | -0.17 | 0.10 | -0.40 | -0.63 | -0.51 | 0.16 | 0.34 |
| O35343 | KPNA4 | 57.922 | 0.15 | 0.06 | 0.10 | 0.07 | -0.32 | -0.16 | -0.24 | 0.11 | 0.34 |
| E9PV24 | FGA | 87.428 | 0.85 | 0.82 | 0.83 | 0.02 | 0.42 | 0.55 | 0.49 | 0.09 | 0.34 |
| P58501 | PAXBP1 | 104.83 | -0.09 | 0.20 | 0.06 | 0.20 | -0.70 | 0.13 | -0.29 | 0.59 | 0.34 |

| Q8BUE4 | AIFM2 | 40.634 | 0.48 | 0.15 | 0.31 | 0.23 | 0.04 | -0.10 | -0.03 | 0.10 | 0.34 |
| --- | --- | --- | --- | --- | --- | --- | --- | --- | --- | --- | --- |
| O35864 | COPS5 | 37.548 | 0.04 | -0.05 | -0.01 | 0.07 | -0.36 | -0.33 | -0.35 | 0.02 | 0.34 |
| Q99JI4 | PSMD6 | 45.536 | 0.10 | -0.09 | 0.01 | 0.13 | -0.37 | -0.30 | -0.34 | 0.05 | 0.34 |
| Q8BSL7 |  |  | 0.50 | 0.37 | 0.43 | 0.10 | 0.06 | 0.13 | 0.09 | 0.05 | 0.34 |
| Q99L47 | ST13 | 41.655 | 0.05 | -0.12 | -0.03 | 0.13 | -0.47 | -0.28 | -0.37 | 0.14 | 0.34 |
| P70362 | UFD1L | 34.481 | 0.22 | 0.10 | 0.16 | 0.08 | -0.26 | -0.11 | -0.18 | 0.11 | 0.34 |
| P11103 | PARP1 | 113.1 | 0.05 | 0.10 | 0.08 | 0.04 | -0.19 | -0.33 | -0.26 | 0.10 | 0.34 |
| P63028 | TPT1 | 19.462 | -0.10 | -0.05 | -0.08 | 0.04 | -0.40 | -0.43 | -0.42 | 0.02 | 0.34 |
| Q9D8W5 | PSMD12 | 52.895 | 0.04 | -0.08 | -0.02 | 0.08 | -0.43 | -0.29 | -0.36 | 0.10 | 0.34 |
| O09061 | PSMB1 | 26.372 | -0.05 | -0.06 | -0.06 | 0.01 | -0.45 | -0.34 | -0.40 | 0.08 | 0.34 |
| Q6P9R2 | OXSR1 | 58.213 | 0.08 | 0.21 | 0.15 | 0.09 | -0.47 | 0.09 | -0.19 | 0.40 | 0.34 |
| Q8K268 | ABCF3 | 79.864 | -0.43 | 0.09 | -0.17 | 0.37 | -0.94 | -0.07 | -0.51 | 0.61 | 0.34 |
| Q922U1 | PRPF3 | 77.454 | 0.22 | 0.20 | 0.21 | 0.01 | -0.20 | -0.06 | -0.13 | 0.10 | 0.34 |

| P54822 | ADSL | 54.866 | -0.07 | -0.13 | -0.10 | 0.04 | -0.47 | -0.40 | -0.44 | 0.05 | 0.34 |
| --- | --- | --- | --- | --- | --- | --- | --- | --- | --- | --- | --- |
| P17439 | GBA | 57.621 | 1.06 | 1.08 | 1.07 | 0.01 | 0.67 | 0.80 | 0.73 | 0.09 | 0.34 |
| Q3UEB3 | PUF60 | 60.248 | -0.13 | 0.21 | 0.04 | 0.24 | -0.63 | 0.05 | -0.29 | 0.48 | 0.33 |
| Q80YR5 | SAFB2 | 111.84 | -0.27 | 0.15 | -0.06 | 0.30 | -0.81 | 0.03 | -0.39 | 0.60 | 0.33 |
| Q99MD9 | NASP | 83.953 | -0.04 | 0.12 | 0.04 | 0.11 | -0.36 | -0.22 | -0.29 | 0.10 | 0.33 |
| Q91X77 | CYP2C50 | 55.764 | 0.02 | -0.06 | -0.02 | 0.06 | -0.49 | -0.22 | -0.36 | 0.19 | 0.33 |
| Q5BLK4 | ZCCHC6 | 169.1 | -0.75 | 0.35 | -0.20 | 0.77 | -0.92 | -0.14 | -0.53 | 0.56 | 0.33 |
| P46664 | ADSS | 50.02 | 0.21 | 0.07 | 0.14 | 0.10 | -0.11 | -0.26 | -0.19 | 0.11 | 0.33 |
| P39054 | DNM2 | 98.144 | 0.34 | 0.55 | 0.44 | 0.15 | 0.03 | 0.19 | 0.11 | 0.11 | 0.33 |
| Q9DCN2 | CYB5R3 | 34.127 | 0.16 | 0.06 | 0.11 | 0.07 | -0.24 | -0.21 | -0.22 | 0.02 | 0.33 |
| Q8BG32 | PSMD11 | 47.436 | 0.02 | -0.03 | -0.01 | 0.04 | -0.43 | -0.25 | -0.34 | 0.13 | 0.33 |
| Q8VD04 | GRIPAP1 | 92.714 | 0.51 | 0.54 | 0.52 | 0.02 | 0.00 | 0.38 | 0.19 | 0.26 | 0.33 |
| Q9ER72 | CARS | 94.859 | -0.38 | -0.44 | -0.41 | 0.05 | -0.76 | -0.72 | -0.74 | 0.03 | 0.33 |

| P62852 | RPS25 | 13.742 | -0.11 | -0.04 | -0.07 | 0.05 | -0.09 | -0.72 | -0.40 | 0.45 | 0.33 |
| --- | --- | --- | --- | --- | --- | --- | --- | --- | --- | --- | --- |
| Q8BWY3 | ETF1 | 49.03 | 0.35 | 0.14 | 0.24 | 0.15 | -0.07 | -0.11 | -0.09 | 0.03 | 0.33 |
| Q60930 | VDAC2 | 31.732 | 0.44 | 0.38 | 0.41 | 0.04 | -0.01 | 0.17 | 0.08 | 0.12 | 0.33 |
| Q61081 | CDC37 | 44.593 | 0.03 | -0.16 | -0.07 | 0.13 | -0.42 | -0.36 | -0.39 | 0.04 | 0.33 |
| Q00623 | APOA1 | 30.615 | 0.10 | 0.07 | 0.09 | 0.02 | -0.31 | -0.17 | -0.24 | 0.10 | 0.33 |
| Q64511 | TOP2B | 181.91 | 0.18 | 0.40 | 0.29 | 0.15 | -0.21 | 0.14 | -0.04 | 0.25 | 0.33 |
| Q6P1B1 | XPNPEP1 | 69.59 | 0.04 | -0.11 | -0.03 | 0.11 | -0.27 | -0.44 | -0.36 | 0.12 | 0.33 |
| Q9Z2W0 | DNPEP | 52.206 | -0.11 | -0.30 | -0.21 | 0.13 | -0.64 | -0.42 | -0.53 | 0.16 | 0.33 |
| Q9Z0R4 | ITSN1 | 194.29 | 1.13 | 0.48 | 0.81 | 0.46 | 0.71 | 0.25 | 0.48 | 0.33 | 0.33 |
| Q9DBJ1 | PGAM1 | 28.832 | 0.11 | 0.00 | 0.05 | 0.07 | -0.26 | -0.29 | -0.27 | 0.02 | 0.33 |
| Q6P542 | ABCF1 | 94.944 | -0.14 | -0.12 | -0.13 | 0.02 | -0.55 | -0.35 | -0.45 | 0.14 | 0.32 |
| Q9CXF4 | TBC1D15 | 76.526 | 0.44 | 0.51 | 0.48 | 0.05 | -0.02 | 0.33 | 0.15 | 0.25 | 0.32 |
| Q9R111 | GDA | 51.012 | 1.00 | 0.76 | 0.88 | 0.17 | 0.58 | 0.53 | 0.55 | 0.04 | 0.32 |

| Q99LB6 | MAT2B | 37.392 | 0.30 | 0.21 | 0.25 | 0.07 | -0.08 | -0.06 | -0.07 | 0.01 | 0.32 |
| --- | --- | --- | --- | --- | --- | --- | --- | --- | --- | --- | --- |
| Q9DB34 | CHMP2A | 25.134 | 0.23 | 0.02 | 0.12 | 0.15 | -0.17 | -0.23 | -0.20 | 0.04 | 0.32 |
| Q9DB05 | NAPA | 33.189 | 0.45 | 0.15 | 0.30 | 0.21 | 0.00 | -0.04 | -0.02 | 0.02 | 0.32 |
| Q8R5C5 | ACTR1B | 42.281 | 0.11 | 0.10 | 0.10 | 0.01 | -0.28 | -0.16 | -0.22 | 0.08 | 0.32 |
| P07309 | TTR | 15.776 | -0.39 | -0.38 | -0.39 | 0.01 | -0.82 | -0.60 | -0.71 | 0.15 | 0.32 |
| Q60668 | HNRNPD | 38.354 | 0.44 | 0.42 | 0.43 | 0.01 | 0.06 | 0.17 | 0.11 | 0.08 | 0.32 |
| P51855 | GSS | 52.246 | 0.21 | 0.11 | 0.16 | 0.07 | -0.21 | -0.11 | -0.16 | 0.08 | 0.32 |
| Q02257 | JUP | 81.8 | 0.11 | 0.25 | 0.18 | 0.10 | -0.52 | 0.24 | -0.14 | 0.54 | 0.32 |
| Q9CX56 | PSMD8 | 39.93 | -0.04 | -0.13 | -0.09 | 0.06 | -0.42 | -0.39 | -0.40 | 0.02 | 0.32 |
| P63321 | RALA | 23.553 | 0.27 | 0.09 | 0.18 | 0.12 | -0.12 | -0.15 | -0.14 | 0.02 | 0.32 |
| Q9ES97 | RTN3 | 103.88 | 0.14 | 0.27 | 0.21 | 0.09 | -0.28 | 0.06 | -0.11 | 0.24 | 0.32 |
| Q9QXL2 | KIF21A | 186.53 | -0.37 | 0.28 | -0.04 | 0.45 | -0.69 | -0.04 | -0.36 | 0.46 | 0.32 |
| Q9R1P1 | PSMB3 | 22.965 | -0.11 | -0.04 | -0.08 | 0.05 | -0.41 | -0.38 | -0.39 | 0.02 | 0.32 |

| Q8VCX1 | AKR1D1 | 37.289 | -0.37 | -0.47 | -0.42 | 0.07 | -0.71 | -0.77 | -0.74 | 0.04 | 0.32 |
| --- | --- | --- | --- | --- | --- | --- | --- | --- | --- | --- | --- |
| P54729 | NUB1 | 70.306 | -0.01 | 0.11 | 0.05 | 0.09 | -0.25 | -0.28 | -0.27 | 0.03 | 0.32 |
| Q9EQU5 | SET | 33.377 | 0.90 | 0.75 | 0.82 | 0.10 | 0.69 | 0.33 | 0.51 | 0.25 | 0.31 |
| Q9R1P4 | PSMA1 | 29.546 | 0.04 | -0.10 | -0.03 | 0.09 | -0.35 | -0.33 | -0.34 | 0.02 | 0.31 |
| P80314 | CCT2 | 57.477 | -0.35 | -0.37 | -0.36 | 0.01 | -0.77 | -0.59 | -0.68 | 0.13 | 0.31 |
| P83917 | CBX1 | 21.418 | 0.17 | 0.17 | 0.17 | 0.00 | -0.15 | -0.13 | -0.14 | 0.01 | 0.31 |
| Q8BJW6 | EIF2A | 64.403 | -0.12 | 0.06 | -0.03 | 0.13 | -0.60 | -0.08 | -0.34 | 0.37 | 0.31 |
| P31938 | MAP2K1 | 43.474 | 0.31 | 0.26 | 0.29 | 0.04 | -0.13 | 0.08 | -0.02 | 0.15 | 0.31 |
| Q9EQH2 | ERAP1 | 106.6 | 0.03 | 0.19 | 0.11 | 0.11 | -0.15 | -0.26 | -0.20 | 0.08 | 0.31 |
| Q9ER39 | TOR1A | 37.829 | 0.31 | 0.17 | 0.24 | 0.10 | -0.10 | -0.05 | -0.07 | 0.03 | 0.31 |
| P62196 | PSMC5 | 45.626 | -0.03 | -0.09 | -0.06 | 0.04 | -0.41 | -0.33 | -0.37 | 0.06 | 0.31 |
| Q78JW9 | UBFD1 | 40.143 | 0.00 | -0.21 | -0.10 | 0.15 | -0.44 | -0.38 | -0.41 | 0.04 | 0.31 |
| P80317 | CCT6A | 58.004 | -0.32 | -0.36 | -0.34 | 0.03 | -0.71 | -0.59 | -0.65 | 0.08 | 0.31 |

| P70302 | STIM1 | 77.566 | 0.22 | 0.43 | 0.32 | 0.15 | -0.31 | 0.33 | 0.01 | 0.45 | 0.31 |
| --- | --- | --- | --- | --- | --- | --- | --- | --- | --- | --- | --- |
| P21550 | ENO3 | 47.024 | -3.12 | -2.93 | -3.03 | 0.13 | -3.28 | -3.39 | -3.33 | 0.08 | 0.31 |
| P70671 | IRF3 | 46.852 | -0.03 | -0.11 | -0.07 | 0.05 | -0.52 | -0.24 | -0.38 | 0.20 | 0.31 |
| P32261 | SERPINC1 | 52.003 | 0.03 | 0.09 | 0.06 | 0.05 | -0.32 | -0.18 | -0.25 | 0.10 | 0.31 |
| P49722 | PSMA2 | 25.926 | -0.07 | -0.09 | -0.08 | 0.01 | -0.45 | -0.32 | -0.38 | 0.10 | 0.31 |
| P43406 | ITGAV | 115.36 | 0.08 | 0.59 | 0.34 | 0.36 | 0.05 | 0.01 | 0.03 | 0.03 | 0.31 |
| P35279 | RAB6A | 23.59 | 0.33 | 0.11 | 0.22 | 0.15 | -0.19 | 0.02 | -0.09 | 0.15 | 0.31 |
| Q8VEK3 | HNRNPU | 87.917 | -0.15 | 0.31 | 0.08 | 0.32 | -0.42 | -0.02 | -0.22 | 0.28 | 0.31 |
| Q9JMH6 | TXNRD1 | 67.083 | 0.73 | 0.61 | 0.67 | 0.09 | 0.31 | 0.42 | 0.36 | 0.08 | 0.31 |
| Q921F2 | TARDBP | 44.547 | 0.29 | 0.27 | 0.28 | 0.01 | -0.20 | 0.15 | -0.03 | 0.25 | 0.31 |
| Q9WTM5 | RUVBL2 | 51.112 | 0.35 | 0.27 | 0.31 | 0.06 | -0.05 | 0.06 | 0.01 | 0.07 | 0.31 |
| E9Q4Z2 | ACACB | 275.75 | -1.28 | -1.00 | -1.14 | 0.20 | -1.44 | -1.45 | -1.45 | 0.01 | 0.30 |
| Q8VCI5 | PEX19 | 32.733 | 0.13 | 0.02 | 0.08 | 0.08 | -0.25 | -0.21 | -0.23 | 0.03 | 0.30 |

| Q9R1P0 | PSMA4 | 29.47 | -0.09 | -0.06 | -0.08 | 0.02 | -0.36 | -0.40 | -0.38 | 0.03 | 0.30 |
| --- | --- | --- | --- | --- | --- | --- | --- | --- | --- | --- | --- |
| Q8VDP4 | CCAR2 | 103 | -0.10 | 0.31 | 0.11 | 0.29 | -0.46 | 0.07 | -0.20 | 0.37 | 0.30 |
| Q62095 | DDX3Y | 73.427 | 0.05 | -0.12 | -0.03 | 0.12 | -0.56 | -0.10 | -0.33 | 0.33 | 0.30 |
| Q91V92 | ACLY | 119.73 | -2.19 | -1.77 | -1.98 | 0.29 | -2.39 | -2.18 | -2.28 | 0.15 | 0.30 |
| P60122 | RUVBL1 | 50.213 | 0.32 | 0.26 | 0.29 | 0.04 | -0.04 | 0.02 | -0.01 | 0.04 | 0.30 |
| P29758 | OAT | 48.354 | 1.27 | 1.16 | 1.22 | 0.08 | 0.94 | 0.89 | 0.92 | 0.03 | 0.30 |
| Q9CZD3 | GARS | 81.877 | 0.38 | 0.23 | 0.31 | 0.11 | 0.09 | -0.07 | 0.01 | 0.12 | 0.30 |
| O08585 | CLTA | 25.604 | 0.57 | 0.46 | 0.51 | 0.08 | 0.19 | 0.24 | 0.21 | 0.04 | 0.30 |
| P54731 | FAF1 | 73.862 | -0.09 | -0.10 | -0.10 | 0.01 | -0.63 | -0.16 | -0.40 | 0.34 | 0.30 |
| Q9D8N2 | FAM45A | 40.42 | 0.49 | 0.23 | 0.36 | 0.18 | 0.03 | 0.10 | 0.06 | 0.05 | 0.30 |
| Q99K48 | NONO | 54.54 | 0.31 | 0.28 | 0.29 | 0.02 | -0.08 | 0.07 | 0.00 | 0.11 | 0.30 |
| O08583 | ALYREF | 26.94 | 0.57 | 0.35 | 0.46 | 0.16 | 0.16 | 0.17 | 0.16 | 0.01 | 0.30 |
| Q8BFZ9 | ERLIN2 | 37.872 | 0.41 | 0.24 | 0.32 | 0.12 | 0.03 | 0.03 | 0.03 | 0.00 | 0.30 |

| G5E829 | ATP2B1 | 134.75 | 0.25 | 0.37 | 0.31 | 0.09 | 0.07 | -0.04 | 0.02 | 0.07 | 0.29 |
| --- | --- | --- | --- | --- | --- | --- | --- | --- | --- | --- | --- |
| Q91YP2 | NLN | 80.428 | 0.49 | 0.31 | 0.40 | 0.13 | 0.17 | 0.04 | 0.11 | 0.09 | 0.29 |
| P63158 | HMGB1 | 24.893 | 0.31 | 0.26 | 0.28 | 0.03 | -0.09 | 0.06 | -0.01 | 0.11 | 0.29 |
| Q923D2 | BLVRB | 22.197 | 0.65 | 0.69 | 0.67 | 0.03 | 0.34 | 0.40 | 0.37 | 0.05 | 0.29 |
| Q91W50 | CSDE1 | 88.79 | -0.02 | 0.15 | 0.07 | 0.12 | -0.41 | -0.04 | -0.23 | 0.27 | 0.29 |
| Q64176 | CES1E | 61.581 | 0.75 | 0.75 | 0.75 | 0.00 | 0.46 | 0.45 | 0.45 | 0.00 | 0.29 |
| Q6ZQ58 | LARP1 | 121.12 | -0.81 | -0.22 | -0.51 | 0.42 | -1.21 | -0.40 | -0.80 | 0.58 | 0.29 |
| Q9CWS0 | DDAH1 | 31.381 | 0.10 | -0.03 | 0.04 | 0.09 | -0.26 | -0.25 | -0.26 | 0.01 | 0.29 |
| P27601 | GNA13 | 44.054 | 0.85 | 0.65 | 0.75 | 0.14 | 0.49 | 0.42 | 0.46 | 0.05 | 0.29 |
| Q64010 | CRK | 33.814 | 0.31 | 0.09 | 0.20 | 0.16 | -0.16 | -0.02 | -0.09 | 0.10 | 0.29 |
| Q9CX34 | SUGT1 | 38.158 | -0.11 | -0.23 | -0.17 | 0.08 | -0.50 | -0.42 | -0.46 | 0.06 | 0.29 |
| O54950 | PRKAG1 | 37.52 | -0.32 | -0.45 | -0.39 | 0.09 | -0.64 | -0.72 | -0.68 | 0.06 | 0.29 |
| Q9WTI7 | MYO1C | 121.94 | 0.61 | 0.85 | 0.73 | 0.17 | 0.48 | 0.39 | 0.44 | 0.06 | 0.29 |

| A2A5R2 | ARFGEF2 | 202.24 | -0.02 | 0.22 | 0.10 | 0.18 | -0.36 | -0.03 | -0.19 | 0.23 | 0.29 |
| --- | --- | --- | --- | --- | --- | --- | --- | --- | --- | --- | --- |
| P16858 | GAPDH | 35.81 | -0.01 | -0.21 | -0.11 | 0.14 | -0.30 | -0.50 | -0.40 | 0.14 | 0.29 |
| Q8R016 | BLMH | 52.511 | -0.42 | -0.41 | -0.41 | 0.00 | -0.71 | -0.70 | -0.70 | 0.00 | 0.29 |
| P54728 | RAD23B | 43.512 | -0.61 | -0.18 | -0.40 | 0.31 | -1.08 | -0.29 | -0.69 | 0.56 | 0.29 |
| Q8VDS8 | STX18 | 38.381 | 0.33 | 0.11 | 0.22 | 0.16 | 0.01 | -0.14 | -0.07 | 0.11 | 0.29 |
| Q99K23 | UFSP2 | 52.515 | 0.32 | 0.18 | 0.25 | 0.10 | -0.12 | 0.04 | -0.04 | 0.12 | 0.29 |
| P40142 | TKT | 67.63 | -0.03 | -0.12 | -0.07 | 0.07 | -0.18 | -0.55 | -0.36 | 0.26 | 0.29 |
| Q9CQF3 | NUDT21 | 26.24 | 0.45 | 0.29 | 0.37 | 0.12 | 0.04 | 0.12 | 0.08 | 0.05 | 0.29 |
| Q6PDM2 | SRSF1 | 27.744 | 0.40 | 0.15 | 0.28 | 0.18 | 0.09 | -0.12 | -0.01 | 0.15 | 0.29 |
| P80315 | CCT4 | 58.066 | -0.28 | -0.39 | -0.34 | 0.08 | -0.68 | -0.57 | -0.63 | 0.08 | 0.29 |
| Q99KK7 | DPP3 | 82.897 | 0.23 | 0.11 | 0.17 | 0.09 | -0.07 | -0.16 | -0.11 | 0.06 | 0.29 |
| Q91V76 |  | 34.995 | -0.23 | -0.35 | -0.29 | 0.09 | -0.50 | -0.65 | -0.57 | 0.11 | 0.29 |
| P97384 | ANXA11 | 54.079 | -0.20 | 0.18 | -0.01 | 0.27 | -0.55 | -0.04 | -0.29 | 0.36 | 0.29 |

| Q3UFF7 | LYPLAL1 | 26.354 | -0.21 | -0.23 | -0.22 | 0.02 | -0.80 | -0.20 | -0.50 | 0.42 | 0.28 |
| --- | --- | --- | --- | --- | --- | --- | --- | --- | --- | --- | --- |
| P48722 | HSPA4L | 94.381 | -0.30 | -0.32 | -0.31 | 0.02 | -0.51 | -0.68 | -0.59 | 0.12 | 0.28 |
| P80316 | CCT5 | 59.623 | -0.35 | -0.39 | -0.37 | 0.03 | -0.73 | -0.57 | -0.65 | 0.11 | 0.28 |
| Q80Y55 | BSDC1 | 46.952 | 0.16 | 0.39 | 0.27 | 0.16 | -0.26 | 0.24 | -0.01 | 0.36 | 0.28 |
| Q9D8S4 | REXO2 | 26.738 | 1.10 | 1.14 | 1.12 | 0.03 | 0.82 | 0.85 | 0.84 | 0.02 | 0.28 |
| Q922D4 | PPP6R3 | 94.652 | -0.27 | 0.21 | -0.03 | 0.34 | -0.38 | -0.24 | -0.31 | 0.10 | 0.28 |
| Q3TC93 | HS1BP3 | 43.691 | 0.51 | 0.52 | 0.51 | 0.00 | 0.25 | 0.22 | 0.23 | 0.02 | 0.28 |
| Q99LI2 | CLCC1 | 60.621 | -0.20 | -0.08 | -0.14 | 0.09 | -0.51 | -0.33 | -0.42 | 0.13 | 0.28 |
| P80313 | CCT7 | 59.652 | -0.31 | -0.36 | -0.34 | 0.04 | -0.64 | -0.59 | -0.62 | 0.03 | 0.28 |
| P61027 | RAB10 | 22.541 | 0.45 | 0.38 | 0.41 | 0.05 | 0.15 | 0.11 | 0.13 | 0.03 | 0.28 |
| Q8R1B4 | EIF3C | 105.53 | 0.01 | 0.03 | 0.02 | 0.01 | -0.37 | -0.15 | -0.26 | 0.16 | 0.28 |
| B8JK39 | ITGA9 | 114.41 | 0.19 | 0.39 | 0.29 | 0.14 | 0.00 | 0.03 | 0.01 | 0.02 | 0.28 |
| Q9D0E1 | HNRNPM | 77.648 | 0.43 | 0.36 | 0.39 | 0.05 | 0.12 | 0.11 | 0.11 | 0.01 | 0.28 |

| P97390 | VPS45 | 65.052 | 0.43 | 0.34 | 0.38 | 0.07 | 0.15 | 0.05 | 0.10 | 0.07 | 0.28 |
| --- | --- | --- | --- | --- | --- | --- | --- | --- | --- | --- | --- |
| Q9JLN9 | MTOR | 288.79 | -0.02 | 0.29 | 0.14 | 0.22 | -0.40 | 0.12 | -0.14 | 0.37 | 0.28 |
| P68037 | UBE2L3 | 17.861 | 0.12 | 0.24 | 0.18 | 0.09 | -0.14 | -0.06 | -0.10 | 0.05 | 0.28 |
| Q9CXW3 | CACYBP | 26.51 | -0.65 | -0.45 | -0.55 | 0.14 | -0.84 | -0.81 | -0.83 | 0.02 | 0.28 |
| Q6NZC7 | SEC23IP | 110.78 | -0.27 | 0.34 | 0.04 | 0.44 | -0.91 | 0.43 | -0.24 | 0.94 | 0.28 |
| Q8CI32 | BAG5 | 50.942 | 0.50 | 0.12 | 0.31 | 0.27 | 0.13 | -0.07 | 0.03 | 0.14 | 0.28 |
| Q9DCL9 | PAICS | 47.006 | 0.31 | 0.19 | 0.25 | 0.09 | -0.14 | 0.09 | -0.03 | 0.16 | 0.27 |
| Q9Z1X4 | ILF3 | 96.02 | 0.55 | 0.43 | 0.49 | 0.09 | 0.29 | 0.15 | 0.22 | 0.10 | 0.27 |
| Q99JX3 | GORASP2 | 47.038 | -0.24 | 0.02 | -0.11 | 0.19 | -0.77 | 0.01 | -0.38 | 0.55 | 0.27 |
| Q9QUR6 | PREP | 80.751 | 0.03 | -0.11 | -0.04 | 0.10 | -0.25 | -0.37 | -0.31 | 0.09 | 0.27 |
| Q9JLV5 | CUL3 | 88.947 | 0.29 | 0.21 | 0.25 | 0.06 | -0.13 | 0.09 | -0.02 | 0.15 | 0.27 |
| O88908 | SOAT2 | 60.597 | -0.19 | -0.39 | -0.29 | 0.14 | -0.67 | -0.45 | -0.56 | 0.15 | 0.27 |
| Q8C2Q3 | RBM14 | 69.448 | 0.10 | 0.06 | 0.08 | 0.02 | -0.27 | -0.11 | -0.19 | 0.11 | 0.27 |

| Q99L45 | EIF2S2 | 38.092 | 0.22 | 0.01 | 0.11 | 0.15 | -0.25 | -0.06 | -0.15 | 0.14 | 0.27 |
| --- | --- | --- | --- | --- | --- | --- | --- | --- | --- | --- | --- |
| Q62086 | PON2 | 39.617 | 0.47 | 0.44 | 0.46 | 0.02 | 0.19 | 0.19 | 0.19 | 0.00 | 0.27 |
| P14685 | PSMD3 | 60.718 | -0.05 | -0.16 | -0.10 | 0.08 | -0.44 | -0.30 | -0.37 | 0.10 | 0.27 |
| Q9D832 | DNAJB4 | 37.781 | 0.32 | 0.11 | 0.22 | 0.15 | -0.20 | 0.09 | -0.05 | 0.21 | 0.27 |
| Q9WVQ5 | APIP | 26.949 | 0.44 | 0.35 | 0.39 | 0.06 | 0.15 | 0.10 | 0.12 | 0.04 | 0.27 |
| P60335 | PCBP1 | 37.497 | 0.02 | 0.14 | 0.08 | 0.09 | -0.25 | -0.13 | -0.19 | 0.09 | 0.27 |
| Q505F5 | LRRC47 | 63.589 | 0.09 | 0.27 | 0.18 | 0.13 | -0.15 | -0.03 | -0.09 | 0.09 | 0.27 |
| P55096 | ABCD3 | 75.474 | -0.39 | -0.27 | -0.33 | 0.08 | -0.47 | -0.72 | -0.60 | 0.17 | 0.27 |
| Q9D0R8 | LSM12 | 21.701 | 0.24 | 0.33 | 0.28 | 0.07 | 0.00 | 0.03 | 0.02 | 0.02 | 0.27 |
| Q8R050 |  | 68.625 | -0.13 | 0.05 | -0.04 | 0.13 | -0.54 | -0.07 | -0.31 | 0.33 | 0.27 |
| Q8BUK6 | HOOK3 | 83.217 | -0.27 | -0.23 | -0.25 | 0.03 | -0.62 | -0.41 | -0.52 | 0.15 | 0.27 |
| P19221 | F2 | 70.268 | -0.06 | 0.37 | 0.16 | 0.31 | -0.22 | 0.00 | -0.11 | 0.16 | 0.27 |
| Q9Z108 | STAU1 | 53.924 | -0.54 | -0.23 | -0.38 | 0.22 | -0.98 | -0.32 | -0.65 | 0.47 | 0.27 |

| Q8BGS1 | EPB41L5 | 81.635 | 0.29 | 0.31 | 0.30 | 0.01 | -0.21 | 0.29 | 0.04 | 0.35 | 0.26 |
| --- | --- | --- | --- | --- | --- | --- | --- | --- | --- | --- | --- |
| Q5SYD0 | MYO1D | 116.08 | 0.49 | 0.76 | 0.63 | 0.19 | 0.39 | 0.34 | 0.36 | 0.03 | 0.26 |
| Q99ME9 | GTPBP4 | 74.112 | 0.38 | 0.31 | 0.35 | 0.05 | 0.13 | 0.04 | 0.08 | 0.07 | 0.26 |
| Q8R2Y0 | ABHD6 | 38.204 | -0.03 | -0.14 | -0.08 | 0.08 | -0.31 | -0.38 | -0.34 | 0.05 | 0.26 |
| Q99PL5 | RRBP1 | 172.88 | -0.14 | -0.11 | -0.13 | 0.02 | -0.39 | -0.40 | -0.39 | 0.01 | 0.26 |
| Q8CIB5 | FERMT2 | 77.8 | 0.21 | 0.10 | 0.16 | 0.08 | -0.14 | -0.07 | -0.10 | 0.05 | 0.26 |
| Q8K1J6 | TRNT1 | 49.895 | 0.50 | 0.32 | 0.41 | 0.12 | 0.31 | -0.01 | 0.15 | 0.22 | 0.26 |
| Q9Z1Z2 | STRAP | 38.442 | 0.32 | 0.17 | 0.24 | 0.11 | -0.04 | 0.01 | -0.02 | 0.04 | 0.26 |
| Q64737 | GART | 107.5 | -0.09 | -0.08 | -0.08 | 0.01 | -0.45 | -0.23 | -0.34 | 0.15 | 0.26 |
| Q9QYJ0 | DNAJA2 | 45.745 | 0.16 | -0.02 | 0.07 | 0.13 | -0.14 | -0.23 | -0.19 | 0.06 | 0.26 |
| Q8C7G5 | APOA5 | 41.262 | 0.84 | 0.50 | 0.67 | 0.24 | 0.47 | 0.35 | 0.41 | 0.09 | 0.26 |
| O54774 | AP3D1 | 135.08 | 0.02 | 0.39 | 0.20 | 0.26 | -0.16 | 0.05 | -0.05 | 0.15 | 0.26 |
| Q8K1X1 | WDR11 | 135.94 | 0.09 | 0.30 | 0.20 | 0.15 | -0.04 | -0.09 | -0.06 | 0.04 | 0.26 |

| Q91Z38 | TTC1 | 33.263 | 0.08 | -0.07 | 0.00 | 0.10 | -0.22 | -0.29 | -0.25 | 0.05 | 0.26 |
| --- | --- | --- | --- | --- | --- | --- | --- | --- | --- | --- | --- |
| Q9CZU6 | CS | 51.736 | -0.11 | -0.23 | -0.17 | 0.08 | -0.50 | -0.35 | -0.43 | 0.11 | 0.26 |
| Q9D662 | SEC23B | 86.436 | 0.08 | -0.10 | -0.01 | 0.13 | -0.49 | -0.04 | -0.26 | 0.32 | 0.25 |
| Q9CYZ2 | TPD52L2 | 24.043 | 0.12 | 0.21 | 0.17 | 0.06 | -0.19 | 0.02 | -0.08 | 0.15 | 0.25 |
| Q9WTX6 | CUL1 | 89.69 | 0.21 | 0.22 | 0.22 | 0.00 | -0.11 | 0.04 | -0.04 | 0.11 | 0.25 |
| P83887 | TUBG1 | 51.1 | 0.22 | 0.18 | 0.20 | 0.03 | 0.13 | -0.23 | -0.05 | 0.25 | 0.25 |
| P61087 | UBE2K | 22.406 | 0.04 | -0.11 | -0.04 | 0.11 | -0.36 | -0.22 | -0.29 | 0.09 | 0.25 |
| P47941 | CRKL | 33.83 | -0.15 | -0.05 | -0.10 | 0.07 | -0.38 | -0.32 | -0.35 | 0.04 | 0.25 |
| Q9QZE7 | TSNAX | 32.926 | 0.10 | 0.19 | 0.14 | 0.06 | -0.04 | -0.18 | -0.11 | 0.10 | 0.25 |
| Q63886 | UGT1A1 | 60.047 | -0.08 | -0.45 | -0.27 | 0.27 | -0.25 | -0.78 | -0.52 | 0.37 | 0.25 |
| Q64FW2 | RETSAT | 67.334 | 1.62 | 1.64 | 1.63 | 0.01 | 1.27 | 1.48 | 1.38 | 0.15 | 0.25 |
| P10639 | TXN | 11.675 | 0.15 | 0.17 | 0.16 | 0.02 | -0.12 | -0.06 | -0.09 | 0.04 | 0.25 |
| P62192 | PSMC1 | 49.184 | -0.08 | -0.06 | -0.07 | 0.01 | -0.40 | -0.24 | -0.32 | 0.11 | 0.25 |

| Q93092 | TALDO1 | 37.387 | 0.77 | 0.50 | 0.63 | 0.19 | 0.48 | 0.29 | 0.38 | 0.13 | 0.25 |
| --- | --- | --- | --- | --- | --- | --- | --- | --- | --- | --- | --- |
| Q3UM29 | COG7 | 86.072 | 0.32 | 0.28 | 0.30 | 0.03 | 0.03 | 0.07 | 0.05 | 0.02 | 0.25 |
| P57780 | ACTN4 | 104.98 | 0.73 | 0.65 | 0.69 | 0.05 | 0.49 | 0.38 | 0.44 | 0.08 | 0.25 |
| Q9CZ28 | SNF8 | 28.886 | 0.25 | -0.04 | 0.11 | 0.21 | -0.19 | -0.10 | -0.14 | 0.06 | 0.25 |
| Q922Y1 | UBXN1 | 33.572 | -0.06 | -0.03 | -0.04 | 0.02 | -0.30 | -0.28 | -0.29 | 0.01 | 0.25 |
| Q8C4Y3 | NELFB | 65.636 | 0.54 | 0.34 | 0.44 | 0.14 | 0.28 | 0.11 | 0.19 | 0.12 | 0.25 |
| Q9D5V5 | CUL5 | 90.973 | 0.12 | 0.08 | 0.10 | 0.03 | -0.21 | -0.08 | -0.15 | 0.09 | 0.25 |
| P10711 | TCEA1 | 33.88 | 0.05 | 0.02 | 0.04 | 0.02 | -0.30 | -0.12 | -0.21 | 0.13 | 0.25 |
| Q8VBW6 | NAE1 | 60.273 | 0.15 | 0.10 | 0.13 | 0.04 | -0.30 | 0.05 | -0.12 | 0.25 | 0.25 |
| Q3URD3 | SLMAP | 96.932 | 0.07 | 0.52 | 0.30 | 0.32 | -0.03 | 0.13 | 0.05 | 0.11 | 0.25 |
| Q9JII6 | AKR1A1 | 36.586 | 0.70 | 0.48 | 0.59 | 0.16 | 0.40 | 0.28 | 0.34 | 0.09 | 0.25 |
| O35344 | KPNA3 | 57.772 | 0.02 | -0.16 | -0.07 | 0.13 | -0.42 | -0.22 | -0.32 | 0.14 | 0.25 |
| P42227 | STAT3 | 88.053 | 0.46 | 0.48 | 0.47 | 0.01 | 0.16 | 0.29 | 0.23 | 0.09 | 0.24 |

| O88685 | PSMC3 | 49.548 | 0.09 | -0.05 | 0.02 | 0.10 | -0.21 | -0.24 | -0.22 | 0.02 | 0.24 |
| --- | --- | --- | --- | --- | --- | --- | --- | --- | --- | --- | --- |
| O35465 | FKBP8 | 43.528 | -0.05 | -0.05 | -0.05 | 0.00 | -0.48 | -0.11 | -0.29 | 0.26 | 0.24 |
| Q5DTM8 | RNF20 | 113.52 | -0.24 | 0.25 | 0.01 | 0.35 | -0.42 | -0.06 | -0.24 | 0.25 | 0.24 |
| Q3TCH7 | CUL4A | 87.752 | -0.45 | -0.31 | -0.38 | 0.10 | -0.71 | -0.54 | -0.62 | 0.12 | 0.24 |
| P70452 | STX4 | 34.165 | 0.39 | 0.36 | 0.37 | 0.02 | 0.15 | 0.11 | 0.13 | 0.03 | 0.24 |
| Q921F4 | HNRNPLL | 64.124 | 0.27 | 0.26 | 0.27 | 0.01 | -0.05 | 0.10 | 0.02 | 0.10 | 0.24 |
| P40336 | VPS26A | 38.113 | 0.51 | 0.36 | 0.43 | 0.11 | 0.10 | 0.29 | 0.19 | 0.14 | 0.24 |
| Q60823 | AKT2 | 55.741 | 0.08 | -0.24 | -0.08 | 0.23 | -0.53 | -0.12 | -0.32 | 0.30 | 0.24 |
| Q8BT60 | CPNE3 | 59.584 | 0.34 | 0.10 | 0.22 | 0.17 | -0.07 | 0.03 | -0.02 | 0.07 | 0.24 |
| P62827 | RAN | 24.423 | 0.02 | 0.04 | 0.03 | 0.01 | -0.24 | -0.17 | -0.21 | 0.05 | 0.24 |
| Q8R0N6 | ADHFE1 | 49.937 | 0.23 | 0.12 | 0.17 | 0.07 | -0.17 | 0.03 | -0.07 | 0.14 | 0.24 |
| Q9CQU1 | MFAP1 | 51.954 | 0.61 | 0.04 | 0.33 | 0.40 | 0.07 | 0.10 | 0.08 | 0.02 | 0.24 |
| Q62165 | DAG1 | 96.904 | 0.70 | 0.43 | 0.57 | 0.19 | 0.39 | 0.26 | 0.33 | 0.09 | 0.24 |

| Q9WVJ2 | PSMD13 | 42.809 | -0.05 | -0.07 | -0.06 | 0.01 | -0.34 | -0.27 | -0.30 | 0.05 | 0.24 |
| --- | --- | --- | --- | --- | --- | --- | --- | --- | --- | --- | --- |
| Q64727 | VCL | 116.72 | 0.38 | 0.95 | 0.66 | 0.40 | 0.31 | 0.53 | 0.42 | 0.15 | 0.24 |
| Q8BU30 | IARS | 144.27 | -0.42 | -0.14 | -0.28 | 0.20 | -0.88 | -0.16 | -0.52 | 0.51 | 0.24 |
| Q9CQ60 | PGLS | 27.254 | 0.36 | 0.22 | 0.29 | 0.10 | 0.07 | 0.03 | 0.05 | 0.03 | 0.24 |
| Q91VC3 | EIF4A3 | 46.839 | 0.50 | 0.34 | 0.42 | 0.11 | 0.28 | 0.08 | 0.18 | 0.14 | 0.24 |
| Q9QZQ8 | H2AFY | 39.735 | 0.79 | 0.57 | 0.68 | 0.15 | 0.50 | 0.38 | 0.44 | 0.09 | 0.24 |
| P70372 | ELAVL1 | 36.169 | 0.36 | 0.22 | 0.29 | 0.10 | 0.06 | 0.04 | 0.05 | 0.02 | 0.24 |
| Q3B7Z2 | OSBP | 88.796 | 0.20 | 0.20 | 0.20 | 0.00 | -0.03 | -0.04 | -0.03 | 0.01 | 0.24 |
| P46935 | NEDD4 | 102.71 | -0.23 | 0.25 | 0.01 | 0.34 | -0.43 | -0.02 | -0.22 | 0.29 | 0.24 |
| P35123 | USP4 | 108.34 | -0.85 | -0.16 | -0.51 | 0.49 | -1.17 | -0.32 | -0.74 | 0.60 | 0.24 |
| Q62093 | SRSF2 | 25.476 | 0.21 | 0.12 | 0.17 | 0.06 | -0.01 | -0.13 | -0.07 | 0.09 | 0.24 |
| P26883 | FKBP1A | 11.922 | 0.18 | 0.12 | 0.15 | 0.04 | -0.20 | 0.02 | -0.09 | 0.15 | 0.24 |
| P61202 | COPS2 | 51.596 | -0.04 | -0.09 | -0.06 | 0.03 | -0.35 | -0.25 | -0.30 | 0.08 | 0.24 |

| P62334 | PSMC6 | 44.172 | -0.03 | -0.10 | -0.06 | 0.05 | -0.32 | -0.28 | -0.30 | 0.03 | 0.24 |
| --- | --- | --- | --- | --- | --- | --- | --- | --- | --- | --- | --- |
| O35685 | NUDC | 38.358 | -0.06 | -0.15 | -0.11 | 0.06 | -0.47 | -0.22 | -0.34 | 0.17 | 0.23 |
| P09803 | CDH1 | 98.255 | -0.99 | -0.50 | -0.74 | 0.34 | -1.23 | -0.72 | -0.98 | 0.36 | 0.23 |
| Q9QZ85 | IIGP1 | 47.571 | -0.77 | -0.90 | -0.83 | 0.09 | -1.10 | -1.03 | -1.07 | 0.05 | 0.23 |
| Q3TDQ1 | STT3B | 93.245 | 0.14 | -0.16 | -0.01 | 0.21 | -0.32 | -0.17 | -0.25 | 0.10 | 0.23 |
| P26638 | SARS | 58.388 | 0.21 | 0.07 | 0.14 | 0.10 | -0.11 | -0.07 | -0.09 | 0.03 | 0.23 |
| Q8BMF4 | DLAT | 67.941 | -0.34 | -0.16 | -0.25 | 0.12 | -0.50 | -0.46 | -0.48 | 0.03 | 0.23 |
| P18572 | BSG | 42.444 | -0.03 | -0.23 | -0.13 | 0.14 | -0.48 | -0.24 | -0.36 | 0.17 | 0.23 |
| Q9D379 | EPHX1 | 52.576 | 1.63 | 1.48 | 1.55 | 0.11 | 1.38 | 1.27 | 1.33 | 0.07 | 0.23 |
| Q9DBZ5 | EIF3K | 25.086 | 0.24 | 0.19 | 0.21 | 0.04 | -0.09 | 0.06 | -0.01 | 0.11 | 0.23 |
| Q8BK63 | CSNK1A1 | 38.914 | 0.49 | 0.35 | 0.42 | 0.10 | 0.17 | 0.21 | 0.19 | 0.03 | 0.23 |
| P27786 | CYP17A1 | 57.637 | -1.38 | -1.39 | -1.39 | 0.01 | -1.69 | -1.54 | -1.62 | 0.10 | 0.23 |
| Q9D7M1 | GID8 | 26.778 | 0.15 | 0.13 | 0.14 | 0.02 | -0.26 | 0.09 | -0.09 | 0.25 | 0.23 |

| P17751 | TPI1 | 32.191 | 0.34 | 0.32 | 0.33 | 0.01 | 0.10 | 0.11 | 0.10 | 0.01 | 0.23 |
| --- | --- | --- | --- | --- | --- | --- | --- | --- | --- | --- | --- |
| Q9D024 | CCDC47 | 55.843 | 0.27 | 0.20 | 0.23 | 0.06 | -0.12 | 0.13 | 0.01 | 0.18 | 0.23 |
| Q61035 | HARS | 57.432 | 0.13 | 0.02 | 0.07 | 0.08 | -0.19 | -0.12 | -0.15 | 0.05 | 0.23 |
| Q9CR09 | UFC1 | 19.481 | -0.14 | -0.17 | -0.15 | 0.02 | -0.32 | -0.43 | -0.38 | 0.08 | 0.22 |
| O08808 | DIAPH1 | 139.34 | -0.66 | 0.23 | -0.22 | 0.63 | -0.88 | 0.00 | -0.44 | 0.63 | 0.22 |
| Q3UIR3 | DTX3L | 83.043 | -0.18 | -0.10 | -0.14 | 0.06 | -0.43 | -0.29 | -0.36 | 0.09 | 0.22 |
| Q91W90 | TXNDC5 | 46.415 | -0.04 | -0.20 | -0.12 | 0.12 | -0.35 | -0.34 | -0.34 | 0.01 | 0.22 |
| P52479 | USP10 | 87.021 | -0.96 | 0.05 | -0.46 | 0.72 | -1.46 | 0.10 | -0.68 | 1.10 | 0.22 |
| Q3TIV5 | ZC3H15 | 48.327 | -0.27 | -0.26 | -0.26 | 0.00 | -0.66 | -0.31 | -0.49 | 0.25 | 0.22 |
| Q9ESW4 | AGK | 46.975 | 0.29 | 0.08 | 0.18 | 0.15 | -0.18 | 0.11 | -0.04 | 0.20 | 0.22 |
| Q9Z204 | HNRNPC | 34.384 | 0.34 | 0.20 | 0.27 | 0.10 | 0.02 | 0.08 | 0.05 | 0.05 | 0.22 |
| P53986 | SLC16A1 | 53.267 | -0.07 | -0.06 | -0.06 | 0.00 | -0.43 | -0.14 | -0.29 | 0.21 | 0.22 |
| Q9EP69 | SACM1L | 66.943 | 0.16 | 0.14 | 0.15 | 0.02 | -0.10 | -0.05 | -0.07 | 0.03 | 0.22 |

| Q9JLF6 | TGM1 | 89.825 | 0.99 | 1.01 | 1.00 | 0.01 | 0.82 | 0.74 | 0.78 | 0.05 | 0.22 |
| --- | --- | --- | --- | --- | --- | --- | --- | --- | --- | --- | --- |
| P46471 | PSMC2 | 48.647 | 0.01 | -0.13 | -0.06 | 0.10 | -0.23 | -0.34 | -0.28 | 0.08 | 0.22 |
| Q9D9V3 | ECHDC1 | 35.467 | -0.35 | -0.40 | -0.38 | 0.03 | -0.73 | -0.46 | -0.59 | 0.20 | 0.22 |
| Q6NZJ6 | EIF4G1 | 176.07 | -0.74 | 0.03 | -0.36 | 0.55 | -0.94 | -0.21 | -0.57 | 0.52 | 0.22 |
| O88844 | IDH1 | 46.674 | -0.23 | -0.28 | -0.25 | 0.03 | -0.53 | -0.41 | -0.47 | 0.08 | 0.22 |
| Q8R081 | HNRNPL | 63.963 | 0.42 | 0.20 | 0.31 | 0.15 | 0.15 | 0.04 | 0.09 | 0.08 | 0.22 |
| Q8BHG1 | NRD1 | 132.89 | 0.06 | 0.21 | 0.14 | 0.10 | -0.07 | -0.09 | -0.08 | 0.02 | 0.22 |
| P04186 | CFB | 85.004 | -0.05 | 0.02 | -0.02 | 0.05 | -0.17 | -0.29 | -0.23 | 0.09 | 0.22 |
|  |  | 26.151 | 0.02 | -0.13 | -0.05 | 0.11 | -0.23 | -0.31 | -0.27 | 0.06 | 0.22 |
| Q3V3R4 | ITGA1 | 130.81 | 0.28 | 0.57 | 0.43 | 0.20 | 0.13 | 0.29 | 0.21 | 0.11 | 0.22 |
| Q3U0V1 | KHSRP | 76.775 | 0.32 | 0.41 | 0.36 | 0.07 | 0.02 | 0.27 | 0.15 | 0.18 | 0.22 |
| P60843 | EIF4A1 | 46.153 | 0.34 | 0.19 | 0.26 | 0.11 | 0.05 | 0.04 | 0.05 | 0.01 | 0.22 |
| O35250 | EXOC7 | 79.959 | 0.46 | 0.39 | 0.42 | 0.04 | 0.22 | 0.20 | 0.21 | 0.02 | 0.21 |

| Q99LD4 | GPS1 | 53.442 | 0.19 | -0.01 | 0.09 | 0.14 | -0.08 | -0.17 | -0.12 | 0.07 | 0.21 |
| --- | --- | --- | --- | --- | --- | --- | --- | --- | --- | --- | --- |
| Q6PGC1 | DHX29 | 153.97 | -0.23 | 0.06 | -0.09 | 0.21 | -0.51 | -0.10 | -0.30 | 0.29 | 0.21 |
| P00375 | DHFR | 21.606 | -0.30 | -0.33 | -0.31 | 0.02 | -0.61 | -0.44 | -0.53 | 0.12 | 0.21 |
| Q9ERE7 | MESDC2 | 25.206 | 0.15 | -0.08 | 0.03 | 0.16 | -0.19 | -0.16 | -0.18 | 0.02 | 0.21 |
| P56657 | CYP2C40 | 55.763 | -1.69 | -1.69 | -1.69 | 0.00 | -1.94 | -1.86 | -1.90 | 0.06 | 0.21 |
| P06728 | APOA4 | 45.029 | 0.24 | 0.06 | 0.15 | 0.13 | -0.11 | -0.02 | -0.06 | 0.07 | 0.21 |
| Q9JHK4 | RABGGTA | 64.989 | -0.16 | -0.15 | -0.16 | 0.00 | -0.42 | -0.31 | -0.37 | 0.08 | 0.21 |
| Q8CIM7 | CYP2D26 | 56.975 | 0.07 | -0.19 | -0.06 | 0.18 | -0.26 | -0.28 | -0.27 | 0.01 | 0.21 |
| Q91X72 | HPX | 51.317 | 1.58 | 1.93 | 1.76 | 0.25 | 1.54 | 1.55 | 1.54 | 0.00 | 0.21 |
| Q571F8 | GLS2 | 66.365 | -0.23 | -0.28 | -0.26 | 0.04 | -0.54 | -0.40 | -0.47 | 0.10 | 0.21 |
| Q9CSN1 | SNW1 | 61.475 | 0.02 | 0.24 | 0.13 | 0.16 | -0.16 | 0.00 | -0.08 | 0.11 | 0.21 |
| P80318 | CCT3 | 60.629 | -0.28 | -0.37 | -0.32 | 0.06 | -0.49 | -0.58 | -0.53 | 0.07 | 0.21 |
| P56654 | CYP2C37 | 55.606 | -1.08 | -0.84 | -0.96 | 0.18 | -1.36 | -0.98 | -1.17 | 0.26 | 0.21 |

| O70435 | PSMA3 | 28.405 | -0.10 | -0.06 | -0.08 | 0.03 | -0.25 | -0.34 | -0.29 | 0.06 | 0.21 |
| --- | --- | --- | --- | --- | --- | --- | --- | --- | --- | --- | --- |
| Q9DC50 | CROT | 70.264 | -0.28 | -0.32 | -0.30 | 0.03 | -0.42 | -0.61 | -0.51 | 0.14 | 0.21 |
| Q6PAM1 | TXLNA | 62.368 | -0.29 | -0.32 | -0.30 | 0.02 | -0.63 | -0.39 | -0.51 | 0.18 | 0.21 |
| P63001 | RAC1 | 21.45 | 0.00 | 0.66 | 0.33 | 0.47 | 0.03 | 0.21 | 0.12 | 0.13 | 0.21 |
| Q8BH86 |  | 66.365 | 0.17 | 0.15 | 0.16 | 0.01 | -0.06 | -0.04 | -0.05 | 0.01 | 0.21 |
| Q6A009 | LTN1 | 198.92 | 0.02 | 0.24 | 0.13 | 0.16 | -0.18 | 0.03 | -0.08 | 0.15 | 0.21 |
| Q8QZR5 | GPT | 55.142 | 0.08 | -0.01 | 0.03 | 0.06 | -0.30 | -0.04 | -0.17 | 0.18 | 0.21 |
|  |  |  | -0.72 | -0.49 | -0.61 | 0.16 | -0.94 | -0.69 | -0.81 | 0.18 | 0.21 |
| Q63932 | MAP2K2 | 44.402 | 0.27 | 0.02 | 0.14 | 0.17 | -0.08 | -0.05 | -0.06 | 0.02 | 0.21 |
| O35309 | NMI | 35.235 | 0.03 | -0.01 | 0.01 | 0.03 | -0.19 | -0.21 | -0.20 | 0.02 | 0.21 |
| Q8BKZ9 | PDHX | 53.998 | -0.14 | -0.19 | -0.16 | 0.04 | -0.42 | -0.32 | -0.37 | 0.07 | 0.21 |
| Q6XVG2 | CYP2C54 | 55.857 | 0.43 | -0.05 | 0.19 | 0.34 | 0.20 | -0.23 | -0.01 | 0.30 | 0.21 |
| Q91V41 | RAB14 | 23.897 | 0.23 | 0.18 | 0.20 | 0.04 | -0.09 | 0.09 | 0.00 | 0.13 | 0.20 |

| Q60737 | CSNK2A1 | 45.133 | -0.17 | 0.08 | -0.04 | 0.18 | -0.49 | -0.01 | -0.25 | 0.34 | 0.20 |
| --- | --- | --- | --- | --- | --- | --- | --- | --- | --- | --- | --- |
| Q6ZQ08 | CNOT1 | 266.8 | -0.05 | 0.36 | 0.15 | 0.29 | -0.23 | 0.13 | -0.05 | 0.26 | 0.20 |
| Q8VDQ8 | SIRT2 | 43.256 | -0.21 | -0.08 | -0.15 | 0.09 | -0.46 | -0.24 | -0.35 | 0.16 | 0.20 |
| P61982 | YWHAG | 28.302 | 0.25 | 0.19 | 0.22 | 0.05 | 0.01 | 0.02 | 0.02 | 0.01 | 0.20 |
| Q9R0A0 | PEX14 | 41.207 | -0.22 | -0.27 | -0.25 | 0.03 | -0.70 | -0.20 | -0.45 | 0.36 | 0.20 |
| P15532 | NME1 | 17.208 | -0.11 | -0.14 | -0.12 | 0.02 | -0.39 | -0.26 | -0.32 | 0.09 | 0.20 |
| Q91WT8 | RBM47 | 64.061 | -0.31 | 0.34 | 0.01 | 0.46 | -0.94 | 0.56 | -0.19 | 1.06 | 0.20 |
| Q9DCN1 | NUDT12 | 51.51 | 0.29 | 0.17 | 0.23 | 0.08 | -0.03 | 0.10 | 0.03 | 0.10 | 0.20 |
| P97855 | G3BP1 | 51.828 | -0.20 | 0.03 | -0.09 | 0.16 | -0.47 | -0.10 | -0.28 | 0.26 | 0.20 |
| P97765 | WBP2 | 28.032 | 0.07 | 0.34 | 0.20 | 0.19 | -0.39 | 0.40 | 0.00 | 0.56 | 0.20 |
| P57784 | SNRPA1 | 28.357 | 0.25 | 0.35 | 0.30 | 0.07 | 0.05 | 0.14 | 0.10 | 0.06 | 0.20 |
| Q64520 | GUK1 | 21.918 | 0.17 | 0.17 | 0.17 | 0.00 | 0.09 | -0.15 | -0.03 | 0.17 | 0.20 |
| P16330 | CNP | 47.123 | 1.07 | 1.11 | 1.09 | 0.03 | 0.75 | 1.03 | 0.89 | 0.19 | 0.20 |

| P26516 | PSMD7 | 36.539 | 0.09 | -0.13 | -0.02 | 0.16 | -0.19 | -0.24 | -0.22 | 0.03 | 0.20 |
| --- | --- | --- | --- | --- | --- | --- | --- | --- | --- | --- | --- |
| P16627 | HSPA1L | 70.636 | 0.04 | -0.22 | -0.09 | 0.19 | -0.17 | -0.40 | -0.29 | 0.16 | 0.20 |
| Q8C7R4 | UBA6 | 117.96 | -0.38 | -0.06 | -0.22 | 0.22 | -0.26 | -0.57 | -0.42 | 0.22 | 0.20 |
| P68254 | YWHAQ | 27.778 | 0.49 | 0.38 | 0.43 | 0.08 | 0.25 | 0.22 | 0.24 | 0.02 | 0.20 |
| Q9JLI8 | SART3 | 109.62 | 0.02 | 0.28 | 0.15 | 0.18 | -0.18 | 0.09 | -0.05 | 0.19 | 0.20 |
| P09528 | FTH1 | 21.066 | 1.13 | 1.79 | 1.46 | 0.46 | 0.68 | 1.84 | 1.26 | 0.82 | 0.20 |
| Q8CAS9 | PARP9 | 96.658 | -0.18 | -0.18 | -0.18 | 0.00 | -0.37 | -0.39 | -0.38 | 0.02 | 0.20 |
| P27773 | PDIA3 | 56.678 | 0.16 | 0.00 | 0.08 | 0.11 | -0.13 | -0.11 | -0.12 | 0.01 | 0.20 |
| Q62348 | TSN | 26.201 | 0.13 | 0.16 | 0.15 | 0.02 | 0.03 | -0.13 | -0.05 | 0.11 | 0.20 |
| Q99KI3 | EMC3 | 29.98 | -0.03 | 0.10 | 0.04 | 0.09 | -0.30 | -0.02 | -0.16 | 0.20 | 0.20 |
| Q9CQM9 | GLRX3 | 37.778 | -0.32 | -0.32 | -0.32 | 0.00 | -0.56 | -0.47 | -0.52 | 0.07 | 0.20 |
| Q9CQK7 | RWDD1 | 27.785 | -0.01 | 0.05 | 0.02 | 0.04 | -0.16 | -0.19 | -0.17 | 0.02 | 0.20 |
| O88543 | COPS3 | 47.832 | 0.00 | -0.09 | -0.04 | 0.06 | -0.22 | -0.26 | -0.24 | 0.03 | 0.20 |

| Q91WK2 | EIF3H | 39.832 | 0.24 | 0.04 | 0.14 | 0.14 | -0.03 | -0.08 | -0.06 | 0.04 | 0.20 |
| --- | --- | --- | --- | --- | --- | --- | --- | --- | --- | --- | --- |
| Q91YE6 | IPO9 | 116.05 | -0.27 | -0.07 | -0.17 | 0.14 | -0.36 | -0.37 | -0.37 | 0.01 | 0.19 |
| Q9CQF9 | PCYOX1 | 56.494 | -0.08 | -0.02 | -0.05 | 0.04 | -0.32 | -0.17 | -0.24 | 0.11 | 0.19 |
| Q8K310 | MATR3 | 94.629 | 0.09 | 0.29 | 0.19 | 0.14 | -0.12 | 0.11 | -0.01 | 0.16 | 0.19 |
| Q1HFZ0 | NSUN2 | 85.451 | 0.30 | 0.41 | 0.35 | 0.08 | 0.11 | 0.20 | 0.16 | 0.06 | 0.19 |
| Q9CQ02 | COMMD4 | 21.86 | 0.22 | 0.25 | 0.24 | 0.02 | 0.01 | 0.07 | 0.04 | 0.05 | 0.19 |
| Q6ZWX6 | EIF2S1 | 36.108 | 0.10 | 0.00 | 0.05 | 0.07 | -0.16 | -0.13 | -0.15 | 0.02 | 0.19 |
| Q9CQC9 | SAR1B | 22.382 | -0.03 | -0.07 | -0.05 | 0.03 | -0.30 | -0.18 | -0.24 | 0.09 | 0.19 |
| Q03734 |  | 47.064 | 0.77 | 0.55 | 0.66 | 0.16 | 0.86 | 0.07 | 0.47 | 0.56 | 0.19 |
| Q8BWU5 | OSGEP | 36.3 | 0.18 | 0.14 | 0.16 | 0.03 | 0.07 | -0.13 | -0.03 | 0.14 | 0.19 |
| Q99KP3 | CRYL1 | 35.208 | 1.95 | 1.81 | 1.88 | 0.10 | 1.58 | 1.79 | 1.69 | 0.15 | 0.19 |
| Q9CZ30 | OLA1 | 44.729 | 0.26 | 0.17 | 0.22 | 0.07 | -0.01 | 0.06 | 0.03 | 0.05 | 0.19 |
| Q9Z1T1 | AP3B1 | 122.74 | 0.42 | 0.35 | 0.39 | 0.06 | 0.26 | 0.13 | 0.19 | 0.10 | 0.19 |

| Q9CQR6 | PPP6C | 35.159 | 0.03 | 0.04 | 0.03 | 0.01 | -0.06 | -0.25 | -0.16 | 0.13 | 0.19 |
| --- | --- | --- | --- | --- | --- | --- | --- | --- | --- | --- | --- |
| Q9Z1D1 | EIF3G | 35.638 | 0.06 | 0.11 | 0.09 | 0.04 | -0.18 | -0.02 | -0.10 | 0.11 | 0.19 |
| Q9CXR1 | DHRS7 | 38.167 | -0.17 | -0.25 | -0.21 | 0.05 | -0.39 | -0.41 | -0.40 | 0.01 | 0.19 |
| Q64514 | TPP2 | 139.88 | -0.68 | -0.21 | -0.44 | 0.34 | -0.67 | -0.59 | -0.63 | 0.06 | 0.19 |
| Q9CQD1 | RAB5A | 23.598 | 0.10 | 0.30 | 0.20 | 0.14 | 0.03 | 0.00 | 0.01 | 0.02 | 0.19 |
| D3YXK2 | SAFB | 105.1 | -0.11 | 0.23 | 0.06 | 0.24 | -0.32 | 0.07 | -0.13 | 0.28 | 0.19 |
| Q60972 | RBBP4 | 47.655 | 0.34 | 0.17 | 0.25 | 0.12 | 0.03 | 0.10 | 0.06 | 0.04 | 0.19 |
| Q9Z0S1 | BPNT1 | 33.196 | 0.04 | 0.16 | 0.10 | 0.08 | -0.15 | -0.02 | -0.09 | 0.09 | 0.19 |
| Q99L88 | SNTB1 | 58.081 | 0.27 | 0.32 | 0.30 | 0.04 | 0.00 | 0.22 | 0.11 | 0.15 | 0.19 |
| Q3UGR5 | HDHD2 | 28.73 | -0.52 | -0.32 | -0.42 | 0.14 | -0.53 | -0.69 | -0.61 | 0.11 | 0.19 |
| Q924Y0 | BBOX1 | 44.698 | -0.49 | -0.59 | -0.54 | 0.07 | -0.74 | -0.71 | -0.73 | 0.03 | 0.19 |
| P54726 | RAD23A | 39.706 | -0.78 | -0.18 | -0.48 | 0.43 | -0.95 | -0.38 | -0.67 | 0.40 | 0.19 |
| Q9R0H0 | ACOX1 | 74.648 | 0.43 | 0.33 | 0.38 | 0.07 | 0.27 | 0.13 | 0.20 | 0.10 | 0.18 |

| Q9DBP5 | CMPK1 | 22.165 | 0.13 | 0.07 | 0.10 | 0.04 | -0.11 | -0.07 | -0.09 | 0.03 | 0.18 |
| --- | --- | --- | --- | --- | --- | --- | --- | --- | --- | --- | --- |
| Q8BJZ4 | MRPS35 | 35.975 | 0.18 | 0.16 | 0.17 | 0.01 | -0.10 | 0.06 | -0.02 | 0.11 | 0.18 |
| Q61733 | MRPS31 | 43.88 | 0.20 | 0.05 | 0.12 | 0.11 | -0.19 | 0.07 | -0.06 | 0.18 | 0.18 |
| Q9D939 | SULT1C2 | 34.953 | -1.88 | -2.12 | -2.00 | 0.17 | -2.15 | -2.21 | -2.18 | 0.04 | 0.18 |
| Q8VCR7 | ABHD14B | 22.45 | -0.04 | -0.01 | -0.02 | 0.02 | -0.47 | 0.05 | -0.21 | 0.36 | 0.18 |
| Q61704 | ITIH3 | 99.357 | 1.10 | 1.63 | 1.36 | 0.37 | 0.86 | 1.50 | 1.18 | 0.45 | 0.18 |
| Q9QZD9 | EIF3I | 36.46 | 0.17 | 0.09 | 0.13 | 0.05 | -0.11 | 0.00 | -0.05 | 0.08 | 0.18 |
| Q6P5F9 | XPO1 | 123.09 | 0.17 | 0.28 | 0.23 | 0.08 | 0.06 | 0.03 | 0.04 | 0.03 | 0.18 |
| P51881 | SLC25A5 | 32.931 | 0.15 | 0.16 | 0.16 | 0.01 | -0.02 | -0.03 | -0.03 | 0.01 | 0.18 |
| Q8CHQ9 | CML2 | 26.424 | 0.87 | 1.23 | 1.05 | 0.25 | 0.48 | 1.25 | 0.87 | 0.55 | 0.18 |
| O35490 | BHMT | 45.02 | 1.00 | 0.92 | 0.96 | 0.06 | 0.80 | 0.76 | 0.78 | 0.02 | 0.18 |
| Q9WUL7 | ARL3 | 20.486 | 0.33 | 0.26 | 0.30 | 0.06 | 0.03 | 0.20 | 0.11 | 0.13 | 0.18 |
| P48678 | LMNA | 74.237 | 0.88 | 0.79 | 0.83 | 0.07 | 0.60 | 0.70 | 0.65 | 0.07 | 0.18 |

| Q9CQ92 | FIS1 | 17.008 | 0.40 | 0.34 | 0.37 | 0.04 | 0.10 | 0.28 | 0.19 | 0.13 | 0.18 |
| --- | --- | --- | --- | --- | --- | --- | --- | --- | --- | --- | --- |
| Q99LE6 | ABCF2 | 71.781 | 0.13 | 0.16 | 0.15 | 0.02 | -0.14 | 0.07 | -0.03 | 0.15 | 0.18 |
| Q9EQH3 | VPS35 | 91.712 | 0.45 | 0.43 | 0.44 | 0.01 | 0.20 | 0.32 | 0.26 | 0.08 | 0.18 |
| Q8BU14 | SEC62 | 45.58 | -0.24 | -0.37 | -0.31 | 0.09 | -0.48 | -0.49 | -0.49 | 0.01 | 0.18 |
| Q9CZ04 | COPS7A | 30.224 | -0.07 | -0.08 | -0.08 | 0.01 | -0.28 | -0.23 | -0.26 | 0.04 | 0.18 |
| Q9WTL7 | LYPLA2 | 24.794 | 0.24 | 0.18 | 0.21 | 0.04 | 0.08 | -0.01 | 0.03 | 0.07 | 0.18 |
| P97370 | ATP1B3 | 31.775 | 0.87 | 0.87 | 0.87 | 0.00 | 0.77 | 0.62 | 0.69 | 0.11 | 0.18 |
| Q9CQR4 | ACOT13 | 15.183 | -0.52 | -0.35 | -0.44 | 0.12 | -0.66 | -0.56 | -0.61 | 0.07 | 0.18 |
| O70475 | UGDH | 54.831 | 2.07 | 1.85 | 1.96 | 0.16 | 1.78 | 1.79 | 1.78 | 0.01 | 0.18 |
| Q62261 | SPTBN1 | 274.22 | 0.12 | 0.48 | 0.30 | 0.25 | 0.04 | 0.21 | 0.12 | 0.12 | 0.17 |
| Q62376 | SNRNP70 | 51.991 | 0.24 | 0.19 | 0.21 | 0.03 | 0.21 | -0.13 | 0.04 | 0.24 | 0.17 |
| Q9QZQ1 | MLLT4 | 206.5 | -0.21 | 0.69 | 0.24 | 0.64 | -0.38 | 0.51 | 0.07 | 0.63 | 0.17 |
| Q5XJY5 | ARCN1 | 57.229 | -0.04 | 0.04 | 0.00 | 0.06 | -0.36 | 0.02 | -0.17 | 0.27 | 0.17 |

| Q8BH58 | TIPRL | 31.253 | -0.07 | -0.12 | -0.09 | 0.03 | -0.36 | -0.17 | -0.27 | 0.13 | 0.17 |
| --- | --- | --- | --- | --- | --- | --- | --- | --- | --- | --- | --- |
| Q06138 | CAB39 | 39.842 | 0.29 | 0.20 | 0.25 | 0.07 | 0.11 | 0.04 | 0.08 | 0.04 | 0.17 |
| P27612 | PLAA | 87.22 | 0.02 | -0.03 | 0.00 | 0.04 | -0.32 | -0.03 | -0.18 | 0.20 | 0.17 |
| Q9R190 | MTA2 | 75.029 | 0.24 | 0.42 | 0.33 | 0.12 | 0.13 | 0.19 | 0.16 | 0.04 | 0.17 |
| P97872 | FMO5 | 60 | 0.97 | 0.91 | 0.94 | 0.04 | 0.76 | 0.78 | 0.77 | 0.02 | 0.17 |
| P10126 | EEF1A1 | 50.113 | -0.04 | -0.23 | -0.14 | 0.14 | -0.21 | -0.40 | -0.31 | 0.14 | 0.17 |
| Q8R1S9 | SLC38A4 | 60.463 | 1.89 | 1.73 | 1.81 | 0.11 | 1.38 | 1.90 | 1.64 | 0.36 | 0.17 |
| O70310 | NMT1 | 56.888 | 0.24 | 0.22 | 0.23 | 0.01 | 0.05 | 0.07 | 0.06 | 0.01 | 0.17 |
| P31786 | DBI | 10 | -0.80 | -0.18 | -0.49 | 0.44 | -0.72 | -0.60 | -0.66 | 0.08 | 0.17 |
| O55143 | ATP2A2 | 114.86 | -0.04 | 0.06 | 0.01 | 0.07 | -0.10 | -0.22 | -0.16 | 0.09 | 0.17 |
| P50285 | FMO1 | 59.914 | -0.79 | -0.72 | -0.75 | 0.05 | -1.07 | -0.78 | -0.92 | 0.21 | 0.17 |
| Q3UHD6 | SNX27 | 60.988 | 0.60 | 0.28 | 0.44 | 0.23 | 0.33 | 0.21 | 0.27 | 0.09 | 0.17 |
| P24527 | LTA4H | 69.05 | 0.13 | 0.15 | 0.14 | 0.01 | 0.13 | -0.18 | -0.03 | 0.22 | 0.17 |

| Q99JX4 | EIF3M | 42.516 | 0.26 | 0.05 | 0.15 | 0.15 | 0.03 | -0.06 | -0.01 | 0.07 | 0.17 |
| --- | --- | --- | --- | --- | --- | --- | --- | --- | --- | --- | --- |
| Q8VC30 | DAK | 59.69 | -0.87 | -0.92 | -0.90 | 0.04 | -1.02 | -1.10 | -1.06 | 0.06 | 0.17 |
| Q9CQH7 | BTF3L4 | 17.27 | 0.16 | 0.11 | 0.14 | 0.04 | -0.08 | 0.01 | -0.03 | 0.06 | 0.17 |
| Q11011 | NPEPPS | 103.32 | 0.23 | 0.19 | 0.21 | 0.03 | 0.09 | 0.00 | 0.04 | 0.06 | 0.17 |
| Q8JZY2 | COMMD10 | 22.812 | 0.21 | 0.22 | 0.21 | 0.00 | -0.05 | 0.15 | 0.05 | 0.14 | 0.17 |
| P42567 | EPS15 | 98.47 | 0.31 | 0.54 | 0.43 | 0.17 | 0.32 | 0.20 | 0.26 | 0.09 | 0.17 |
| O88544 | COPS4 | 46.284 | -0.04 | -0.06 | -0.05 | 0.02 | -0.22 | -0.21 | -0.21 | 0.01 | 0.17 |
| P07901 | HSP90AA1 | 84.787 | -0.27 | -0.34 | -0.31 | 0.05 | -0.44 | -0.51 | -0.47 | 0.05 | 0.17 |
| Q6P4T2 | SNRNP200 | 244.54 | -0.04 | 0.41 | 0.18 | 0.32 | -0.09 | 0.12 | 0.02 | 0.15 | 0.17 |
| Q60770 | STXBP3 | 67.942 | 0.20 | 0.47 | 0.33 | 0.19 | 0.19 | 0.14 | 0.17 | 0.04 | 0.17 |
| P25444 | RPS2 | 31.231 | 0.09 | -0.10 | 0.00 | 0.13 | -0.18 | -0.15 | -0.17 | 0.02 | 0.16 |
| O35969 | GAMT | 26.336 | -1.23 | -1.30 | -1.26 | 0.04 | -1.42 | -1.44 | -1.43 | 0.01 | 0.16 |
| P17047 | LAMP2 | 45.681 | 0.52 | 0.62 | 0.57 | 0.07 | 0.51 | 0.31 | 0.41 | 0.14 | 0.16 |

| Q8CHG3 | GCC2 | 194.44 | 0.22 | 0.37 | 0.29 | 0.11 | 0.18 | 0.08 | 0.13 | 0.07 | 0.16 |
| --- | --- | --- | --- | --- | --- | --- | --- | --- | --- | --- | --- |
| Q9Z1Q9 | VARS | 140.21 | -0.10 | 0.25 | 0.07 | 0.25 | -0.13 | -0.04 | -0.09 | 0.06 | 0.16 |
| B2RY56 | RBM25 | 99.551 | -0.10 | 0.23 | 0.07 | 0.23 | -0.29 | 0.10 | -0.10 | 0.28 | 0.16 |
| Q8VCM8 | NCLN | 62.907 | 0.31 | 0.36 | 0.34 | 0.04 | 0.20 | 0.15 | 0.18 | 0.03 | 0.16 |
| P11499 | HSP90AB1 | 83.28 | -0.31 | -0.40 | -0.35 | 0.06 | -0.51 | -0.53 | -0.52 | 0.02 | 0.16 |
| Q8BWJ3 | PHKA2 | 138.49 | -0.84 | -0.35 | -0.60 | 0.35 | -0.99 | -0.52 | -0.76 | 0.33 | 0.16 |
| Q02053 | UBA1 | 117.81 | 0.08 | 0.15 | 0.12 | 0.04 | 0.00 | -0.09 | -0.05 | 0.06 | 0.16 |
| P35564 | CANX | 67.277 | 0.09 | -0.03 | 0.03 | 0.09 | -0.10 | -0.16 | -0.13 | 0.04 | 0.16 |
| Q8BYU6 | TOR1AIP2 | 54.495 | 0.23 | 0.34 | 0.29 | 0.08 | 0.04 | 0.21 | 0.13 | 0.12 | 0.16 |
| P45878 | FKBP2 | 15.344 | -0.03 | -0.02 | -0.03 | 0.00 | -0.22 | -0.15 | -0.19 | 0.05 | 0.16 |
| Q9Z0R9 | FADS2 | 52.387 | -1.29 | -1.40 | -1.35 | 0.08 | -1.56 | -1.46 | -1.51 | 0.07 | 0.16 |
| Q6Y7W8 | GIGYF2 | 149.19 | -1.01 | -0.11 | -0.56 | 0.63 | -1.18 | -0.26 | -0.72 | 0.65 | 0.16 |
| Q9WTX5 | SKP1 | 18.672 | 0.14 | 0.04 | 0.09 | 0.07 | 0.00 | -0.13 | -0.06 | 0.09 | 0.16 |

|  |  | 29.343 | 0.02 | -0.10 | -0.04 | 0.09 | -0.38 | -0.02 | -0.20 | 0.26 | 0.16 |
| --- | --- | --- | --- | --- | --- | --- | --- | --- | --- | --- | --- |
| Q8BYC6 | TAOK3 | 105.33 | -0.39 | -0.11 | -0.25 | 0.20 | -0.47 | -0.35 | -0.41 | 0.09 | 0.16 |
| F8VPU2 | FARP1 | 118.87 | -0.63 | -0.01 | -0.32 | 0.44 | -0.82 | -0.13 | -0.47 | 0.49 | 0.16 |
| P62259 | YWHAE | 29.174 | 0.13 | 0.07 | 0.10 | 0.04 | -0.03 | -0.08 | -0.05 | 0.04 | 0.16 |
| P16125 | LDHB | 36.572 | -0.67 | -0.16 | -0.42 | 0.36 | -0.45 | -0.70 | -0.57 | 0.18 | 0.16 |
| Q61712 | DNAJC1 | 63.869 | -0.02 | -0.16 | -0.09 | 0.10 | -0.29 | -0.21 | -0.25 | 0.06 | 0.15 |
| P61979 | HNRNPK | 50.976 | 0.27 | 0.23 | 0.25 | 0.03 | 0.06 | 0.13 | 0.09 | 0.05 | 0.15 |
| P56399 | USP5 | 95.832 | 0.02 | 0.02 | 0.02 | 0.00 | -0.11 | -0.16 | -0.13 | 0.03 | 0.15 |
| Q8VBZ3 | CLPTM1 | 75.29 | -0.06 | 0.09 | 0.02 | 0.11 | -0.29 | 0.02 | -0.14 | 0.22 | 0.15 |
| Q3TPX4 | EXOC5 | 81.737 | 0.73 | 0.55 | 0.64 | 0.13 | 0.74 | 0.23 | 0.49 | 0.36 | 0.15 |
| Q64152 | BTF3 | 22.031 | 0.04 | -0.05 | -0.01 | 0.06 | -0.18 | -0.14 | -0.16 | 0.03 | 0.15 |
| Q9DAR7 | DCPS | 38.988 | 0.24 | 0.08 | 0.16 | 0.11 | 0.01 | 0.01 | 0.01 | 0.00 | 0.15 |
| P63017 | HSPA8 | 70.87 | 0.01 | -0.08 | -0.03 | 0.07 | -0.09 | -0.28 | -0.18 | 0.13 | 0.15 |

| Q9CWL8 | CTNNBL1 | 64.979 | 0.68 | 0.54 | 0.61 | 0.10 | 0.58 | 0.34 | 0.46 | 0.17 | 0.15 |
| --- | --- | --- | --- | --- | --- | --- | --- | --- | --- | --- | --- |
| Q8R4H2 | ARHGEF12 | 172.35 | -0.35 | 0.37 | 0.01 | 0.51 | -0.39 | 0.11 | -0.14 | 0.36 | 0.15 |
| Q6PDG5 | SMARCC2 | 132.6 | -0.27 | 0.33 | 0.03 | 0.43 | -0.33 | 0.08 | -0.12 | 0.29 | 0.15 |
| Q8K183 | PDXK | 35.015 | -0.06 | -0.20 | -0.13 | 0.11 | -0.25 | -0.31 | -0.28 | 0.04 | 0.15 |
| P55258 | RAB8A | 23.668 | -0.06 | -0.04 | -0.05 | 0.01 | -0.31 | -0.08 | -0.20 | 0.16 | 0.15 |
| Q9D554 | SF3A3 | 58.841 | 0.51 | 0.37 | 0.44 | 0.10 | 0.33 | 0.25 | 0.29 | 0.06 | 0.15 |
| Q9CRD0 | OCIAD1 | 27.61 | -0.31 | -0.29 | -0.30 | 0.02 | -0.54 | -0.36 | -0.45 | 0.12 | 0.15 |
| Q99L04 | DHRS1 | 34.005 | 0.20 | 0.06 | 0.13 | 0.10 | 0.04 | -0.07 | -0.02 | 0.08 | 0.15 |
| Q61545 | EWSR1 | 68.461 | -1.19 | 0.38 | -0.41 | 1.11 | -1.11 | -0.01 | -0.56 | 0.78 | 0.15 |
| Q9D906 | ATG7 | 77.519 | 0.17 | 0.09 | 0.13 | 0.06 | 0.02 | -0.05 | -0.02 | 0.04 | 0.15 |
| Q6A4J8 | USP7 | 128.47 | 0.30 | 0.47 | 0.38 | 0.12 | 0.33 | 0.14 | 0.24 | 0.14 | 0.15 |
| Q9D6R2 | IDH3A | 39.638 | -0.09 | -0.14 | -0.12 | 0.03 | -0.25 | -0.28 | -0.27 | 0.02 | 0.15 |
| Q3ULJ0 | GPD1L | 38.225 | -0.04 | -0.13 | -0.08 | 0.06 | -0.28 | -0.18 | -0.23 | 0.08 | 0.15 |

| Q61335 | BCAP31 | 27.956 | 0.44 | 0.30 | 0.37 | 0.10 | 0.20 | 0.25 | 0.22 | 0.04 | 0.15 |
| --- | --- | --- | --- | --- | --- | --- | --- | --- | --- | --- | --- |
| Q6NV83 | U2SURP | 118.26 | -0.16 | 0.22 | 0.03 | 0.27 | -0.25 | 0.02 | -0.12 | 0.19 | 0.15 |
| Q99K01 | PDXDC1 | 87.334 | 0.16 | 0.30 | 0.23 | 0.10 | -0.30 | 0.46 | 0.08 | 0.53 | 0.15 |
| Q8CAQ8 | IMMT | 83.899 | -0.12 | -0.08 | -0.10 | 0.03 | -0.51 | 0.01 | -0.25 | 0.37 | 0.15 |
| Q8CFI7 | POLR2B | 133.91 | 0.12 | 0.39 | 0.26 | 0.19 | 0.17 | 0.05 | 0.11 | 0.08 | 0.15 |
| Q8VDM4 | PSMD2 | 100.2 | 0.00 | -0.10 | -0.05 | 0.07 | -0.09 | -0.30 | -0.19 | 0.15 | 0.15 |
| P20108 | PRDX3 | 28.127 | -0.02 | 0.09 | 0.03 | 0.08 | -0.18 | -0.04 | -0.11 | 0.10 | 0.15 |
| Q9ERF3 | WDR61 | 33.772 | 0.03 | 0.07 | 0.05 | 0.03 | -0.12 | -0.07 | -0.10 | 0.04 | 0.15 |
| P70398 | USP9X | 290.71 | -0.06 | 0.45 | 0.20 | 0.36 | -0.06 | 0.17 | 0.05 | 0.17 | 0.14 |
| Q8CC88 | VWA8 | 213.42 | -0.80 | -0.60 | -0.70 | 0.14 | -0.94 | -0.75 | -0.84 | 0.13 | 0.14 |
| O88533 | DDC | 53.873 | 0.10 | 0.09 | 0.09 | 0.00 | -0.07 | -0.03 | -0.05 | 0.03 | 0.14 |
| Q9R0P3 | ESD | 31.319 | -0.05 | -0.24 | -0.14 | 0.13 | -0.25 | -0.32 | -0.29 | 0.05 | 0.14 |
| Q8BFS6 | CPPED1 | 35.247 | 0.13 | 0.02 | 0.08 | 0.08 | 0.03 | -0.17 | -0.07 | 0.14 | 0.14 |

| Q62087 | PON3 | 39.351 | 0.42 | 0.42 | 0.42 | 0.00 | 0.23 | 0.33 | 0.28 | 0.07 | 0.14 |
| --- | --- | --- | --- | --- | --- | --- | --- | --- | --- | --- | --- |
| O54754 | AOX1 | 146.68 | 1.07 | 1.28 | 1.18 | 0.15 | 0.70 | 1.37 | 1.03 | 0.47 | 0.14 |
| Q8BGC0 | HTATSF1 | 86.239 | 0.09 | 0.18 | 0.13 | 0.06 | 0.01 | -0.03 | -0.01 | 0.03 | 0.14 |
| Q6NSR8 | NPEPL1 | 55.939 | 0.14 | -0.03 | 0.06 | 0.12 | -0.09 | -0.08 | -0.08 | 0.01 | 0.14 |
| Q8R570 | SNAP47 | 46.524 | -0.40 | -0.33 | -0.36 | 0.05 | -0.70 | -0.31 | -0.50 | 0.28 | 0.14 |
| P47791 | GSR | 53.662 | 0.55 | 0.57 | 0.56 | 0.01 | 0.32 | 0.52 | 0.42 | 0.14 | 0.14 |
| P70335 | ROCK1 | 158.17 | 0.10 | 0.27 | 0.19 | 0.12 | -0.01 | 0.11 | 0.05 | 0.09 | 0.14 |
| Q9CZX8 | RPS19 | 16.085 | -0.04 | -0.01 | -0.03 | 0.02 | -0.13 | -0.19 | -0.16 | 0.04 | 0.14 |
| P52430 | PON1 | 39.565 | -0.47 | -0.54 | -0.50 | 0.05 | -0.66 | -0.62 | -0.64 | 0.03 | 0.14 |
| Q02819 | NUCB1 | 53.408 | 0.23 | 0.12 | 0.18 | 0.08 | -0.04 | 0.13 | 0.04 | 0.12 | 0.14 |
| O08715 | AKAP1 | 92.194 | -0.93 | -0.29 | -0.61 | 0.45 | -1.30 | -0.19 | -0.74 | 0.78 | 0.13 |
| Q9R078 | PRKAB1 | 30.308 | -0.57 | -0.50 | -0.53 | 0.05 | -0.67 | -0.66 | -0.67 | 0.01 | 0.13 |
| Q9ER88 | DAP3 | 44.699 | 0.26 | 0.15 | 0.20 | 0.08 | 0.07 | 0.07 | 0.07 | 0.00 | 0.13 |

| P22599 | SERPINA1B | 45.974 | 0.41 | 0.68 | 0.54 | 0.19 | 0.30 | 0.53 | 0.41 | 0.16 | 0.13 |
| --- | --- | --- | --- | --- | --- | --- | --- | --- | --- | --- | --- |
| P23116 | EIF3A | 161.93 | -0.10 | 0.12 | 0.01 | 0.15 | -0.27 | 0.03 | -0.12 | 0.22 | 0.13 |
| Q569Z5 | DDX46 | 117.45 | 0.21 | 0.23 | 0.22 | 0.02 | 0.17 | 0.00 | 0.09 | 0.12 | 0.13 |
| Q8BP47 | NARS | 64.279 | 0.13 | 0.10 | 0.12 | 0.03 | 0.00 | -0.03 | -0.02 | 0.02 | 0.13 |
| Q9ESE1 | LRBA | 317.06 | -0.13 | 0.13 | 0.00 | 0.18 | -0.45 | 0.20 | -0.13 | 0.46 | 0.13 |
| O88967 | YME1L1 | 80.027 | -0.05 | -0.07 | -0.06 | 0.02 | -0.37 | 0.00 | -0.19 | 0.26 | 0.13 |
| Q64674 | SRM | 33.995 | -0.21 | -0.26 | -0.23 | 0.03 | -0.31 | -0.42 | -0.36 | 0.07 | 0.13 |
| Q00PI9 | HNRNPUL2 | 84.939 | -0.23 | 0.41 | 0.09 | 0.46 | -0.09 | 0.02 | -0.04 | 0.08 | 0.13 |
| Q9EP71 | RAI14 | 108.85 | -0.19 | 0.54 | 0.18 | 0.51 | -0.31 | 0.40 | 0.05 | 0.50 | 0.13 |
| Q3UPL0 | SEC31A | 133.57 | -0.62 | 0.02 | -0.30 | 0.45 | -0.92 | 0.06 | -0.43 | 0.69 | 0.13 |
| Q9CZU3 | SKIV2L2 | 117.64 | -0.09 | 0.34 | 0.12 | 0.30 | -0.01 | 0.00 | 0.00 | 0.00 | 0.13 |
| P58252 | EEF2 | 95.313 | -0.06 | -0.13 | -0.09 | 0.05 | -0.17 | -0.27 | -0.22 | 0.07 | 0.13 |
| P53994 | RAB2A | 23.547 | 0.23 | 0.01 | 0.12 | 0.15 | 0.15 | -0.17 | -0.01 | 0.23 | 0.13 |

| Q9DD20 | METTL7B | 28.049 | -0.15 | -0.07 | -0.11 | 0.06 | -0.30 | -0.17 | -0.24 | 0.10 | 0.12 |
| --- | --- | --- | --- | --- | --- | --- | --- | --- | --- | --- | --- |
| Q8R0F9 | SEC14L4 | 46.053 | 0.14 | 0.24 | 0.19 | 0.07 | -0.05 | 0.18 | 0.06 | 0.16 | 0.12 |
| Q8C7V8 | CCDC134 | 26.494 | 0.15 | 0.19 | 0.17 | 0.03 | 0.06 | 0.03 | 0.05 | 0.02 | 0.12 |
| P46061 | RANGAP1 | 63.53 | -0.12 | -0.07 | -0.09 | 0.03 | -0.23 | -0.20 | -0.22 | 0.02 | 0.12 |
| Q9DCT1 | AKR1E2 | 34.46 | -0.06 | -0.29 | -0.17 | 0.16 | -0.22 | -0.38 | -0.30 | 0.11 | 0.12 |
| P68372 | TUBB4B | 49.83 | 0.26 | 0.19 | 0.22 | 0.05 | 0.21 | -0.01 | 0.10 | 0.16 | 0.12 |
| Q8BHG2 |  | 18.02 | -0.14 | -0.21 | -0.18 | 0.05 | -0.26 | -0.35 | -0.30 | 0.06 | 0.12 |
| P47964 | RPL36 | 12.215 | -0.10 | -0.08 | -0.09 | 0.01 | -0.10 | -0.32 | -0.21 | 0.15 | 0.12 |
| Q8BY87 | USP47 | 157.45 | 0.67 | 0.83 | 0.75 | 0.11 | 0.43 | 0.82 | 0.63 | 0.28 | 0.12 |
| P35550 | FBL | 34.306 | 0.61 | 0.50 | 0.56 | 0.08 | 0.39 | 0.48 | 0.44 | 0.06 | 0.12 |
| P28474 | ADH5 | 39.547 | 0.18 | 0.00 | 0.09 | 0.13 | -0.02 | -0.04 | -0.03 | 0.02 | 0.12 |
| Q9CYH2 | FAM213A | 24.394 | -0.80 | -0.79 | -0.79 | 0.00 | -0.89 | -0.94 | -0.91 | 0.03 | 0.12 |
| P70404 | IDH3G | 42.785 | -0.09 | -0.19 | -0.14 | 0.07 | -0.24 | -0.29 | -0.26 | 0.03 | 0.12 |

| Q9D2R0 | AACS | 75.199 | -1.04 | -1.24 | -1.14 | 0.15 | -1.11 | -1.41 | -1.26 | 0.21 | 0.12 |
| --- | --- | --- | --- | --- | --- | --- | --- | --- | --- | --- | --- |
| O08601 | MTTP | 99.098 | -0.31 | -0.34 | -0.33 | 0.02 | -0.43 | -0.46 | -0.45 | 0.02 | 0.12 |
| P09103 | P4HB | 57.058 | -0.02 | -0.16 | -0.09 | 0.10 | -0.19 | -0.22 | -0.21 | 0.02 | 0.12 |
| P17742 | PPIA | 17.971 | -0.12 | 0.35 | 0.11 | 0.33 | -0.06 | 0.05 | 0.00 | 0.08 | 0.12 |
| Q80U95 | UBE3C | 123.97 | -0.46 | 0.11 | -0.17 | 0.40 | -0.63 | 0.05 | -0.29 | 0.48 | 0.12 |
| P35486 | PDHA1 | 43.231 | -0.25 | -0.35 | -0.30 | 0.07 | -0.49 | -0.35 | -0.42 | 0.10 | 0.12 |
| Q8K190 | SAYSD1 | 20.703 | -0.17 | -0.08 | -0.12 | 0.06 | -0.37 | -0.10 | -0.24 | 0.19 | 0.12 |
| P59017 | BCL2L13 | 46.719 | -0.72 | -0.02 | -0.37 | 0.50 | -1.07 | 0.10 | -0.49 | 0.83 | 0.12 |
| P17563 | SELENBP1 | 52.513 | 0.28 | 0.17 | 0.23 | 0.08 | -0.18 | 0.40 | 0.11 | 0.41 | 0.11 |
| Q64331 | MYO6 | 146.41 | -0.60 | -0.04 | -0.32 | 0.40 | -0.84 | -0.03 | -0.43 | 0.57 | 0.11 |
| Q920B9 | SUPT16H | 119.82 | -0.04 | 0.36 | 0.16 | 0.28 | 0.01 | 0.08 | 0.05 | 0.05 | 0.11 |
| Q9D8V0 | HM13 | 41.747 | -0.21 | -0.17 | -0.19 | 0.03 | -0.54 | -0.07 | -0.30 | 0.34 | 0.11 |
| Q9CQI7 | SNRPB2 | 25.323 | 0.51 | 0.34 | 0.43 | 0.12 | 0.22 | 0.40 | 0.31 | 0.13 | 0.11 |

| Q4KMM3 | OXR1 | 95.911 | -0.40 | 0.33 | -0.04 | 0.52 | -0.40 | 0.11 | -0.15 | 0.36 | 0.11 |
| --- | --- | --- | --- | --- | --- | --- | --- | --- | --- | --- | --- |
| Q8VIJ6 | SFPQ | 75.441 | -0.26 | 0.14 | -0.06 | 0.28 | -0.43 | 0.09 | -0.17 | 0.37 | 0.11 |
| Q9R0M6 | RAB9A | 22.909 | 0.33 | 0.30 | 0.31 | 0.03 | 0.14 | 0.26 | 0.20 | 0.09 | 0.11 |
| P06151 | LDHA | 36.498 | 0.10 | -0.05 | 0.03 | 0.11 | 0.00 | -0.17 | -0.08 | 0.12 | 0.11 |
| Q921M4 | GOLGA2 | 113.28 | -0.21 | 0.14 | -0.03 | 0.25 | -0.40 | 0.11 | -0.14 | 0.36 | 0.11 |
| P35700 | PRDX1 | 22.176 | -0.05 | -0.20 | -0.13 | 0.11 | -0.21 | -0.26 | -0.23 | 0.04 | 0.11 |
| P15327 | BPGM | 29.978 | 0.80 | 0.76 | 0.78 | 0.03 | 0.66 | 0.68 | 0.67 | 0.01 | 0.11 |
| Q91X34 | BAAT | 46.481 | 0.65 | 0.52 | 0.58 | 0.09 | 0.40 | 0.54 | 0.47 | 0.10 | 0.11 |
| Q9CWI3 | BCCIP | 35.942 | 0.61 | 0.03 | 0.32 | 0.41 | 0.43 | -0.01 | 0.21 | 0.31 | 0.11 |
| Q9D711 | PIR | 32.066 | 0.68 | 0.53 | 0.61 | 0.11 | 0.50 | 0.50 | 0.50 | 0.00 | 0.11 |
| Q8BMP6 | ACBD3 | 60.18 | 0.22 | 0.24 | 0.23 | 0.01 | 0.08 | 0.18 | 0.13 | 0.07 | 0.11 |
| Q8VCH6 | DHCR24 | 60.112 | -0.15 | -0.07 | -0.11 | 0.05 | -0.34 | -0.10 | -0.22 | 0.17 | 0.11 |
| Q80W21 | GSTM7 | 25.709 | 0.11 | 0.15 | 0.13 | 0.03 | 0.01 | 0.04 | 0.03 | 0.03 | 0.11 |

| Q9WUQ2 | PREB | 45.437 | 0.33 | 0.14 | 0.24 | 0.13 | 0.12 | 0.14 | 0.13 | 0.02 | 0.11 |
| --- | --- | --- | --- | --- | --- | --- | --- | --- | --- | --- | --- |
| Q9D880 | TIMM50 | 39.776 | 0.32 | 0.08 | 0.20 | 0.17 | -0.01 | 0.20 | 0.10 | 0.15 | 0.11 |
| Q14C51 | PTCD3 | 77.795 | 0.21 | 0.06 | 0.13 | 0.11 | 0.04 | 0.01 | 0.02 | 0.02 | 0.11 |
| Q64133 | MAOA | 59.601 | 0.54 | 0.52 | 0.53 | 0.02 | 0.51 | 0.35 | 0.43 | 0.11 | 0.11 |
| Q8VCT4 | CES1D | 61.787 | -0.31 | -0.42 | -0.37 | 0.08 | -0.39 | -0.55 | -0.47 | 0.11 | 0.11 |
| Q99LF4 | RTCB | 55.249 | 0.20 | 0.14 | 0.17 | 0.04 | 0.04 | 0.09 | 0.07 | 0.03 | 0.11 |
| Q91X44 | GCKR | 64.679 | -0.15 | -0.17 | -0.16 | 0.01 | -0.20 | -0.33 | -0.26 | 0.09 | 0.11 |
| Q8BK72 | MRPS27 | 47.778 | 0.20 | 0.01 | 0.10 | 0.13 | 0.01 | -0.02 | 0.00 | 0.02 | 0.11 |
| P13439 | UMPS | 52.292 | -0.33 | -0.38 | -0.36 | 0.04 | -0.51 | -0.41 | -0.46 | 0.07 | 0.10 |
| P14211 | CALR | 47.994 | 0.22 | 0.08 | 0.15 | 0.10 | 0.05 | 0.04 | 0.04 | 0.00 | 0.10 |
| Q99LG2 | TNPO2 | 100.46 | 0.03 | 0.02 | 0.03 | 0.01 | -0.08 | -0.07 | -0.08 | 0.01 | 0.10 |
| Q9ERU9 | RANBP2 | 341.12 | -0.35 | 0.13 | -0.11 | 0.34 | -0.72 | 0.30 | -0.21 | 0.72 | 0.10 |
| Q9WUU7 | CTSZ | 33.996 | 1.09 | 1.10 | 1.09 | 0.01 | 0.70 | 1.28 | 0.99 | 0.41 | 0.10 |

| Q810B6 | ANKFY1 | 128.65 | -0.12 | 0.54 | 0.21 | 0.47 | 0.06 | 0.16 | 0.11 | 0.07 | 0.10 |
| --- | --- | --- | --- | --- | --- | --- | --- | --- | --- | --- | --- |
| Q8BRF7 | SCFD1 | 72.322 | -0.29 | -0.10 | -0.20 | 0.13 | -0.50 | -0.10 | -0.30 | 0.28 | 0.10 |
| Q68FD5 | CLTC | 191.55 | 0.22 | 0.38 | 0.30 | 0.12 | 0.13 | 0.27 | 0.20 | 0.10 | 0.10 |
| Q02248 | CTNNB1 | 85.47 | 0.19 | 0.14 | 0.17 | 0.04 | -0.02 | 0.15 | 0.07 | 0.12 | 0.10 |
| O35678 | MGLL | 33.387 | -0.91 | -1.09 | -1.00 | 0.12 | -1.00 | -1.20 | -1.10 | 0.13 | 0.10 |
| Q8BL66 | EEA1 | 160.91 | -0.05 | 0.28 | 0.12 | 0.23 | -0.13 | 0.16 | 0.02 | 0.20 | 0.10 |
| Q9CRD2 | EMC2 | 34.934 | 0.08 | 0.12 | 0.10 | 0.03 | -0.02 | 0.02 | 0.00 | 0.03 | 0.10 |
| Q8WTY4 | CIAPIN1 | 33.429 | 0.01 | 0.11 | 0.06 | 0.07 | -0.18 | 0.09 | -0.04 | 0.19 | 0.10 |
| Q3TXS7 | PSMD1 | 105.73 | -0.10 | -0.07 | -0.09 | 0.02 | -0.11 | -0.26 | -0.19 | 0.11 | 0.10 |
| Q7TQK5 | CCDC93 | 72.602 | 0.36 | 0.27 | 0.32 | 0.06 | 0.28 | 0.15 | 0.22 | 0.10 | 0.10 |
| Q9CXE7 | TMED5 | 26.172 | 0.39 | 0.27 | 0.33 | 0.09 | 0.15 | 0.31 | 0.23 | 0.12 | 0.10 |
| A6H630 | ARMT1 | 50.548 | -0.09 | -0.03 | -0.06 | 0.04 | -0.27 | -0.05 | -0.16 | 0.16 | 0.10 |
| Q9ESX5 | DKC1 | 57.401 | 0.64 | 0.51 | 0.57 | 0.09 | 0.57 | 0.37 | 0.47 | 0.14 | 0.10 |

| O88952 | LIN7C | 21.834 | 0.83 | 0.78 | 0.81 | 0.03 | 0.61 | 0.80 | 0.71 | 0.13 | 0.10 |
| --- | --- | --- | --- | --- | --- | --- | --- | --- | --- | --- | --- |
| A0JNU3 | ASPG | 60.594 | -0.72 | -1.19 | -0.96 | 0.33 | -1.25 | -0.87 | -1.06 | 0.27 | 0.10 |
| Q9D0M1 | PRPSAP1 | 39.431 | -0.10 | -0.08 | -0.09 | 0.01 | -0.10 | -0.28 | -0.19 | 0.13 | 0.10 |
| P51660 | HSD17B4 | 79.481 | 0.20 | 0.18 | 0.19 | 0.02 | 0.04 | 0.14 | 0.09 | 0.07 | 0.10 |
| P62242 | RPS8 | 24.205 | 0.19 | 0.08 | 0.13 | 0.08 | 0.02 | 0.05 | 0.03 | 0.02 | 0.10 |
| Q80UM3 | NAA15 | 100.96 | 0.21 | 0.15 | 0.18 | 0.04 | 0.20 | -0.03 | 0.08 | 0.16 | 0.10 |
| P48193 | EPB41 | 95.91 | -0.32 | 0.46 | 0.07 | 0.55 | -0.50 | 0.44 | -0.03 | 0.66 | 0.10 |
| P50580 | PA2G4 | 43.698 | 0.46 | 0.24 | 0.35 | 0.16 | 0.31 | 0.19 | 0.25 | 0.08 | 0.10 |
|  |  | 16.46 | 0.25 | 0.17 | 0.21 | 0.06 | 0.16 | 0.05 | 0.11 | 0.08 | 0.10 |
| Q61768 | KIF5B | 109.55 | -0.04 | 0.36 | 0.16 | 0.29 | 0.08 | 0.04 | 0.06 | 0.03 | 0.10 |
| P30999 | CTNND1 | 104.92 | 0.16 | 0.38 | 0.27 | 0.15 | 0.02 | 0.33 | 0.17 | 0.22 | 0.10 |
| Q99MN1 | KARS | 67.839 | 0.08 | 0.02 | 0.05 | 0.04 | 0.01 | -0.11 | -0.05 | 0.09 | 0.10 |
| Q99J08 | SEC14L2 | 46.3 | -0.35 | -0.44 | -0.40 | 0.06 | -0.56 | -0.43 | -0.49 | 0.09 | 0.10 |

| Q7TMK9 | SYNCRIP | 69.632 | 0.07 | 0.20 | 0.13 | 0.09 | 0.10 | -0.03 | 0.03 | 0.10 | 0.10 |
| --- | --- | --- | --- | --- | --- | --- | --- | --- | --- | --- | --- |
| Q9Z2X1 | HNRNPF | 45.729 | 0.14 | 0.29 | 0.22 | 0.11 | 0.25 | -0.01 | 0.12 | 0.19 | 0.10 |
| Q8C0C7 | FARSA | 57.598 | 0.21 | 0.02 | 0.11 | 0.13 | 0.12 | -0.08 | 0.02 | 0.14 | 0.10 |
| Q9D0D3 | MTPAP | 65.229 | -0.11 | -0.05 | -0.08 | 0.04 | -0.47 | 0.12 | -0.18 | 0.42 | 0.10 |
| P60229 | EIF3E | 52.22 | 0.16 | 0.09 | 0.13 | 0.05 | 0.09 | -0.03 | 0.03 | 0.08 | 0.09 |
| Q8K1M6 | DNM1L | 82.657 | -0.23 | -0.20 | -0.22 | 0.02 | -0.35 | -0.27 | -0.31 | 0.05 | 0.09 |
| Q9JL35 | HMGN5 | 45.343 | 0.26 | 0.14 | 0.20 | 0.09 | 0.06 | 0.15 | 0.11 | 0.06 | 0.09 |
| Q9EPU4 | CPSF1 | 160.82 | 0.48 | 0.41 | 0.44 | 0.05 | 0.38 | 0.32 | 0.35 | 0.04 | 0.09 |
| Q9R099 | TBL2 | 49.583 | 0.04 | 0.03 | 0.03 | 0.01 | -0.15 | 0.03 | -0.06 | 0.13 | 0.09 |
| O09174 | AMACR | 41.704 | 0.00 | -0.09 | -0.04 | 0.06 | -0.14 | -0.14 | -0.14 | 0.00 | 0.09 |
| P62281 | RPS11 | 18.431 | -0.06 | 0.00 | -0.03 | 0.04 | -0.22 | -0.02 | -0.12 | 0.14 | 0.09 |
| P61021 | RAB5B | 23.707 | -0.05 | 0.06 | 0.01 | 0.08 | -0.17 | 0.00 | -0.09 | 0.12 | 0.09 |
| P60670 | NPLOC4 | 68.016 | 0.20 | 0.14 | 0.17 | 0.04 | 0.09 | 0.07 | 0.08 | 0.01 | 0.09 |

| Q9D1P4 | CHORDC1 | 37.35 | -0.31 | -0.40 | -0.35 | 0.07 | -0.34 | -0.55 | -0.45 | 0.15 | 0.09 |
| --- | --- | --- | --- | --- | --- | --- | --- | --- | --- | --- | --- |
| Q9DCC7 | ISOC2B | 23.151 | -0.26 | -0.44 | -0.35 | 0.13 | -0.30 | -0.58 | -0.44 | 0.19 | 0.09 |
| Q8BJ71 | NUP93 | 93.28 | 0.07 | 0.10 | 0.09 | 0.02 | -0.07 | 0.06 | -0.01 | 0.09 | 0.09 |
| O08795 | PRKCSH | 58.792 | 0.07 | 0.01 | 0.04 | 0.04 | -0.07 | -0.03 | -0.05 | 0.03 | 0.09 |
| P49443 | PPM1A | 42.432 | 0.09 | 0.00 | 0.04 | 0.06 | -0.06 | -0.03 | -0.05 | 0.02 | 0.09 |
| P11862 | GAS2 | 34.9 | 1.51 | 1.43 | 1.47 | 0.06 | 1.22 | 1.54 | 1.38 | 0.23 | 0.09 |
| Q921M3 | SF3B3 | 135.55 | 0.22 | 0.39 | 0.30 | 0.12 | 0.25 | 0.17 | 0.21 | 0.05 | 0.09 |
| Q3TNA1 | XYLB | 59.543 | 0.23 | 0.03 | 0.13 | 0.14 | 0.00 | 0.08 | 0.04 | 0.06 | 0.09 |
| O55142 | RPL35A | 12.554 | -0.06 | -0.10 | -0.08 | 0.03 | -0.27 | -0.08 | -0.17 | 0.14 | 0.09 |
| Q8BK64 | AHSA1 | 38.117 | -0.50 | -0.43 | -0.46 | 0.05 | -0.56 | -0.56 | -0.56 | 0.00 | 0.09 |
| P53657 | PKLR | 62.308 | -1.88 | -1.93 | -1.90 | 0.04 | -2.03 | -1.96 | -1.99 | 0.05 | 0.09 |
| Q9QXY6 | EHD3 | 60.82 | 0.17 | 0.20 | 0.19 | 0.02 | -0.14 | 0.33 | 0.10 | 0.33 | 0.09 |
| P53026 | RPL10A | 24.916 | 0.11 | -0.01 | 0.05 | 0.09 | -0.06 | -0.02 | -0.04 | 0.03 | 0.09 |

| Q91VF2 | HNMT | 33.664 | 0.55 | 0.60 | 0.58 | 0.04 | 0.45 | 0.53 | 0.49 | 0.06 | 0.09 |
| --- | --- | --- | --- | --- | --- | --- | --- | --- | --- | --- | --- |
| Q91X83 | MAT1A | 43.508 | 0.36 | 0.09 | 0.23 | 0.19 | 0.20 | 0.08 | 0.14 | 0.09 | 0.09 |
| Q8JZN5 | ACAD9 | 68.721 | 0.24 | 0.14 | 0.19 | 0.07 | 0.02 | 0.19 | 0.10 | 0.12 | 0.09 |
| Q91VR5 | DDX1 | 82.499 | 0.17 | 0.15 | 0.16 | 0.01 | 0.05 | 0.09 | 0.07 | 0.03 | 0.09 |
| Q9CRB9 | CHCHD3 | 26.334 | -0.03 | -0.12 | -0.07 | 0.07 | -0.27 | -0.05 | -0.16 | 0.15 | 0.09 |
| P63073 | EIF4E | 25.053 | 0.20 | 0.13 | 0.16 | 0.05 | 0.06 | 0.09 | 0.07 | 0.03 | 0.09 |
| Q91YM4 | TBRG4 | 71.513 | 0.06 | -0.03 | 0.01 | 0.07 | -0.21 | 0.06 | -0.08 | 0.19 | 0.09 |
| Q9JLJ2 | ALDH9A1 | 53.514 | 0.04 | -0.21 | -0.09 | 0.18 | -0.20 | -0.14 | -0.17 | 0.04 | 0.09 |
| Q99PV0 | PRPF8 | 273.61 | 0.03 | 0.40 | 0.21 | 0.26 | 0.20 | 0.05 | 0.13 | 0.11 | 0.09 |
| Q3UHX2 | PDAP1 | 20.605 | -0.05 | -0.08 | -0.06 | 0.02 | -0.38 | 0.08 | -0.15 | 0.32 | 0.09 |
| Q9CQE8 |  | 28.152 | 0.18 | 0.16 | 0.17 | 0.02 | 0.07 | 0.10 | 0.08 | 0.02 | 0.09 |
| P62918 | RPL8 | 28.024 | 0.13 | -0.04 | 0.04 | 0.12 | -0.03 | -0.05 | -0.04 | 0.01 | 0.09 |
| O70378 | EMC8 | 23.348 | 0.08 | 0.09 | 0.09 | 0.01 | -0.09 | 0.09 | 0.00 | 0.13 | 0.09 |

| P41105 | RPL28 | 15.733 | -0.15 | -0.06 | -0.10 | 0.07 | -0.22 | -0.16 | -0.19 | 0.05 | 0.09 |
| --- | --- | --- | --- | --- | --- | --- | --- | --- | --- | --- | --- |
| Q68FL6 | MARS | 101.43 | 0.07 | -0.01 | 0.03 | 0.05 | -0.02 | -0.09 | -0.06 | 0.05 | 0.09 |
| Q6NS46 | PDCD11 | 207.78 | -0.19 | 0.21 | 0.01 | 0.29 | -0.04 | -0.11 | -0.07 | 0.05 | 0.09 |
| Q3UMF0 | COBLL1 | 137.38 | -0.23 | 0.46 | 0.11 | 0.48 | -0.39 | 0.45 | 0.03 | 0.59 | 0.08 |
| Q9CX00 | IST1 | 39.468 | -0.01 | 0.22 | 0.10 | 0.16 | -0.02 | 0.05 | 0.02 | 0.05 | 0.08 |
| P62754 | RPS6 | 28.68 | 0.20 | 0.03 | 0.12 | 0.12 | 0.05 | 0.02 | 0.03 | 0.02 | 0.08 |
| Q9Z2M7 | PMM2 | 27.656 | -0.14 | -0.28 | -0.21 | 0.10 | -0.32 | -0.27 | -0.29 | 0.03 | 0.08 |
| Q91ZX7 | LRP1 | 504.74 | 0.24 | 0.45 | 0.34 | 0.15 | 0.20 | 0.33 | 0.26 | 0.09 | 0.08 |
| Q3UDE2 | TTLL12 | 74.042 | 0.15 | 0.14 | 0.14 | 0.01 | 0.36 | -0.24 | 0.06 | 0.43 | 0.08 |
| Q9JMH9 | MYO18A | 232.75 | -0.26 | 0.38 | 0.06 | 0.45 | -0.28 | 0.24 | -0.02 | 0.37 | 0.08 |
| Q9JIG7 | CCDC22 | 70.843 | 0.30 | 0.18 | 0.24 | 0.09 | 0.05 | 0.26 | 0.16 | 0.15 | 0.08 |
| P68040 | GNB2L1 | 35.076 | 0.12 | -0.02 | 0.05 | 0.10 | -0.05 | -0.01 | -0.03 | 0.02 | 0.08 |
| Q9CR67 | TMEM33 | 28.031 | -0.32 | -0.25 | -0.28 | 0.05 | -0.25 | -0.48 | -0.36 | 0.16 | 0.08 |

| Q3UUQ7 | PGAP1 | 104.58 | 0.79 | 0.80 | 0.79 | 0.01 | 0.82 | 0.61 | 0.71 | 0.15 | 0.08 |
| --- | --- | --- | --- | --- | --- | --- | --- | --- | --- | --- | --- |
| P57776 | EEF1D | 31.293 | 0.25 | 0.08 | 0.16 | 0.12 | 0.08 | 0.09 | 0.08 | 0.00 | 0.08 |
| Q8QZY1 | EIF3L | 66.612 | 0.14 | 0.06 | 0.10 | 0.06 | 0.08 | -0.05 | 0.02 | 0.09 | 0.08 |
| Q04750 | TOP1 | 90.875 | 0.01 | 0.02 | 0.02 | 0.01 | -0.02 | -0.11 | -0.06 | 0.06 | 0.08 |
| P01027 | C3 | 186.48 | 0.08 | 0.29 | 0.19 | 0.14 | 0.08 | 0.13 | 0.11 | 0.04 | 0.08 |
| Q99LX0 | PARK7 | 20.021 | 0.14 | 0.15 | 0.14 | 0.01 | 0.00 | 0.13 | 0.06 | 0.09 | 0.08 |
| Q8CH18 | CCAR1 | 132.06 | -0.45 | 0.26 | -0.09 | 0.50 | -0.35 | 0.00 | -0.17 | 0.24 | 0.08 |
| Q9D7N3 | MRPS9 | 44.929 | 0.16 | 0.10 | 0.13 | 0.04 | 0.02 | 0.08 | 0.05 | 0.04 | 0.08 |
| Q3THG9 | AARSD1 | 44.97 | -0.13 | -0.30 | -0.22 | 0.12 | -0.27 | -0.32 | -0.29 | 0.04 | 0.08 |
| P62849 | RPS24 | 15.423 | -0.01 | -0.06 | -0.04 | 0.04 | -0.17 | -0.07 | -0.12 | 0.07 | 0.08 |
| P55264 | ADK | 40.148 | 0.22 | -0.02 | 0.10 | 0.17 | 0.06 | -0.01 | 0.02 | 0.05 | 0.08 |
| Q9CZM2 | RPL15 | 24.146 | 0.11 | -0.14 | -0.02 | 0.18 | -0.04 | -0.15 | -0.09 | 0.08 | 0.08 |
| P60867 | RPS20 | 13.373 | 0.01 | -0.01 | 0.00 | 0.02 | -0.11 | -0.03 | -0.07 | 0.06 | 0.08 |

| Q80W54 | ZMPSTE24 | 54.734 | 0.56 | 0.31 | 0.44 | 0.17 | 0.39 | 0.33 | 0.36 | 0.04 | 0.08 |
| --- | --- | --- | --- | --- | --- | --- | --- | --- | --- | --- | --- |
| Q8C7E7 | STBD1 | 36.127 | 0.49 | 0.38 | 0.43 | 0.08 | 0.30 | 0.42 | 0.36 | 0.08 | 0.08 |
| Q9D1Q6 | ERP44 | 46.852 | -0.14 | -0.20 | -0.17 | 0.04 | -0.29 | -0.19 | -0.24 | 0.07 | 0.08 |
| P51410 | RPL9 | 21.881 | 0.06 | -0.01 | 0.03 | 0.05 | -0.08 | -0.02 | -0.05 | 0.04 | 0.07 |
| P34884 | MIF | 12.504 | -0.27 | 0.20 | -0.04 | 0.33 | -0.15 | -0.07 | -0.11 | 0.06 | 0.07 |
| Q9CXW2 | MRPS22 | 41.192 | 0.14 | 0.12 | 0.13 | 0.01 | 0.08 | 0.03 | 0.06 | 0.04 | 0.07 |
| Q5SWD9 | TSR1 | 92.104 | -0.23 | -0.13 | -0.18 | 0.07 | -0.25 | -0.26 | -0.25 | 0.01 | 0.07 |
| Q9D8X1 | CUTC | 29.003 | 0.53 | 0.48 | 0.50 | 0.03 | 0.40 | 0.46 | 0.43 | 0.04 | 0.07 |
| P16331 | PAH | 51.899 | 0.06 | -0.16 | -0.05 | 0.16 | -0.14 | -0.10 | -0.12 | 0.03 | 0.07 |
| Q78IK4 | APOOL | 29.26 | -0.24 | -0.30 | -0.27 | 0.05 | -0.46 | -0.23 | -0.34 | 0.16 | 0.07 |
| P35979 | RPL12 | 17.804 | -0.09 | -0.04 | -0.07 | 0.04 | -0.19 | -0.09 | -0.14 | 0.08 | 0.07 |
| Q8CI04 | COG3 | 93.282 | 0.36 | 0.38 | 0.37 | 0.01 | 0.31 | 0.29 | 0.30 | 0.01 | 0.07 |
| Q99K28 | ARFGAP2 | 56.598 | -0.47 | -0.17 | -0.32 | 0.22 | -0.71 | -0.07 | -0.39 | 0.45 | 0.07 |

| O09012 | PEX5 | 70.755 | 0.00 | -0.17 | -0.09 | 0.12 | -0.22 | -0.09 | -0.15 | 0.09 | 0.07 |
| --- | --- | --- | --- | --- | --- | --- | --- | --- | --- | --- | --- |
| Q921L3 | TMCO1 | 21.175 | 0.30 | 0.25 | 0.27 | 0.04 | 0.04 | 0.37 | 0.21 | 0.24 | 0.07 |
| O08573 | LGALS9 | 40.035 | -0.32 | -0.44 | -0.38 | 0.08 | -0.40 | -0.50 | -0.45 | 0.07 | 0.07 |
| P97351 | RPS3A | 29.885 | 0.13 | -0.01 | 0.06 | 0.10 | -0.02 | 0.01 | -0.01 | 0.02 | 0.06 |
| P08032 | SPTA1 | 279.86 | 0.82 | 0.95 | 0.88 | 0.09 | 0.72 | 0.92 | 0.82 | 0.14 | 0.06 |
| Q60864 | STIP1 | 62.581 | -0.30 | -0.33 | -0.31 | 0.02 | -0.32 | -0.43 | -0.37 | 0.08 | 0.06 |
| P61222 | ABCE1 | 67.314 | 0.00 | 0.03 | 0.02 | 0.02 | -0.02 | -0.07 | -0.05 | 0.04 | 0.06 |
| Q9QZ73 | DCUN1D1 | 30.097 | -0.02 | -0.02 | -0.02 | 0.00 | -0.10 | -0.06 | -0.08 | 0.03 | 0.06 |
| P08003 | PDIA4 | 71.982 | 0.02 | -0.26 | -0.12 | 0.20 | -0.10 | -0.26 | -0.18 | 0.11 | 0.06 |
| Q64442 | SORD | 38.249 | -0.18 | -0.38 | -0.28 | 0.14 | -0.31 | -0.38 | -0.34 | 0.05 | 0.06 |
| P70195 | PSMB7 | 29.891 | -0.20 | -0.23 | -0.22 | 0.02 | -0.30 | -0.26 | -0.28 | 0.03 | 0.06 |
| Q61171 | PRDX2 | 21.778 | 0.31 | 0.22 | 0.26 | 0.07 | 0.22 | 0.18 | 0.20 | 0.03 | 0.06 |
| P21440 | ABCB4 | 140.38 | 0.12 | 0.38 | 0.25 | 0.18 | 0.10 | 0.28 | 0.19 | 0.12 | 0.06 |

| P32233 | DRG1 | 40.512 | -0.13 | -0.24 | -0.18 | 0.08 | -0.26 | -0.23 | -0.25 | 0.02 | 0.06 |
| --- | --- | --- | --- | --- | --- | --- | --- | --- | --- | --- | --- |
| P62908 | RPS3 | 26.674 | 0.15 | -0.02 | 0.06 | 0.12 | 0.01 | 0.00 | 0.00 | 0.01 | 0.06 |
| P62267 | RPS23 | 15.807 | 0.00 | -0.04 | -0.02 | 0.03 | -0.09 | -0.08 | -0.08 | 0.01 | 0.06 |
| Q91VH6 | MEMO1 | 33.692 | 0.03 | 0.18 | 0.11 | 0.10 | -0.08 | 0.17 | 0.04 | 0.18 | 0.06 |
| Q80W22 | THNSL2 | 54.187 | -0.09 | -0.21 | -0.15 | 0.09 | -0.22 | -0.20 | -0.21 | 0.01 | 0.06 |
| Q8BWP5 | TTPA | 32.014 | 0.05 | 0.05 | 0.05 | 0.00 | -0.04 | 0.03 | -0.01 | 0.05 | 0.06 |
| P14115 | RPL27A | 16.605 | 0.02 | 0.01 | 0.02 | 0.01 | -0.02 | -0.06 | -0.04 | 0.03 | 0.06 |
| P84091 | AP2M1 | 49.654 | 0.45 | 0.37 | 0.41 | 0.05 | 0.36 | 0.35 | 0.35 | 0.01 | 0.06 |
| Q9D0F9 | PGM1 | 61.417 | 0.02 | -0.02 | 0.00 | 0.03 | -0.01 | -0.11 | -0.06 | 0.07 | 0.06 |
|  |  | 24.604 | -0.01 | 0.02 | 0.00 | 0.02 | -0.15 | 0.04 | -0.06 | 0.13 | 0.06 |
| P19253 | RPL13A | 23.464 | -0.01 | 0.03 | 0.01 | 0.03 | -0.04 | -0.05 | -0.04 | 0.00 | 0.06 |
| Q01853 | VCP | 89.321 | 0.13 | -0.02 | 0.06 | 0.11 | 0.09 | -0.10 | 0.00 | 0.13 | 0.06 |
| Q3U1J4 | DDB1 | 126.85 | -0.17 | 0.12 | -0.03 | 0.21 | -0.03 | -0.14 | -0.08 | 0.07 | 0.06 |

| Q9D7G0 | PRPS1 | 34.834 | -0.26 | -0.32 | -0.29 | 0.04 | -0.27 | -0.42 | -0.35 | 0.10 | 0.06 |
| --- | --- | --- | --- | --- | --- | --- | --- | --- | --- | --- | --- |
| P45591 | CFL2 | 18.709 | 0.07 | -0.03 | 0.02 | 0.07 | -0.04 | -0.02 | -0.03 | 0.01 | 0.06 |
| Q9JI75 | NQO2 | 26.248 | -0.33 | -0.27 | -0.30 | 0.04 | -0.37 | -0.33 | -0.35 | 0.03 | 0.05 |
| Q6A026 | PDS5A | 150.33 | 0.07 | 0.35 | 0.21 | 0.19 | 0.46 | -0.15 | 0.16 | 0.43 | 0.05 |
| Q8BFY9 | TNPO1 | 102.36 | 0.11 | 0.13 | 0.12 | 0.01 | 0.07 | 0.06 | 0.06 | 0.01 | 0.05 |
| Q8BRK8 | PRKAA2 | 62.022 | -0.76 | -0.97 | -0.87 | 0.15 | -0.95 | -0.89 | -0.92 | 0.04 | 0.05 |
| Q8BGQ7 | AARS | 106.91 | 0.12 | 0.17 | 0.14 | 0.03 | 0.15 | 0.03 | 0.09 | 0.09 | 0.05 |
| P14869 | RPLP0 | 34.216 | 0.17 | -0.01 | 0.08 | 0.13 | 0.07 | -0.01 | 0.03 | 0.06 | 0.05 |
| Q60902 | EPS15L1 | 99.307 | -0.42 | 0.26 | -0.08 | 0.48 | -0.29 | 0.03 | -0.13 | 0.22 | 0.05 |
| P47963 | RPL13 | 24.305 | 0.03 | -0.08 | -0.03 | 0.08 | -0.06 | -0.10 | -0.08 | 0.03 | 0.05 |
| P27659 | RPL3 | 46.109 | 0.05 | -0.05 | 0.00 | 0.07 | -0.06 | -0.04 | -0.05 | 0.01 | 0.05 |
| P14576 | SRP54 | 55.72 | -0.17 | -0.25 | -0.21 | 0.05 | -0.30 | -0.22 | -0.26 | 0.06 | 0.05 |
| Q3THK7 | GMPS | 76.723 | 0.15 | 0.05 | 0.10 | 0.07 | 0.15 | -0.05 | 0.05 | 0.14 | 0.05 |

| Q9R1C7 | PRPF40A | 108.48 | -0.47 | 0.17 | -0.15 | 0.45 | -0.32 | -0.08 | -0.20 | 0.17 | 0.05 |
| --- | --- | --- | --- | --- | --- | --- | --- | --- | --- | --- | --- |
| Q9QZD8 | SLC25A10 | 31.715 | 0.23 | 0.16 | 0.19 | 0.05 | 0.14 | 0.14 | 0.14 | 0.00 | 0.05 |
| Q9DBN5 | LONP2 | 94.525 | -0.05 | -0.13 | -0.09 | 0.06 | -0.10 | -0.17 | -0.14 | 0.05 | 0.05 |
| Q9CW03 | SMC3 | 141.55 | 0.01 | 0.27 | 0.14 | 0.19 | 0.12 | 0.06 | 0.09 | 0.04 | 0.05 |
| O09131 | GSTO1 | 27.497 | -0.16 | -0.26 | -0.21 | 0.06 | -0.24 | -0.28 | -0.26 | 0.03 | 0.05 |
| P12970 | RPL7A | 29.976 | -0.02 | 0.04 | 0.01 | 0.05 | -0.05 | -0.03 | -0.04 | 0.01 | 0.05 |
| Q60932 | VDAC1 | 32.351 | 0.20 | 0.02 | 0.11 | 0.12 | 0.07 | 0.06 | 0.06 | 0.01 | 0.05 |
| Q01405 | SEC23A | 86.161 | 0.14 | 0.16 | 0.15 | 0.01 | 0.09 | 0.11 | 0.10 | 0.02 | 0.05 |
| Q9ERK4 | CSE1L | 110.45 | -0.03 | 0.02 | -0.01 | 0.04 | 0.03 | -0.14 | -0.05 | 0.12 | 0.05 |
| Q91YP3 | DERA | 34.975 | -0.09 | -0.23 | -0.16 | 0.10 | -0.17 | -0.23 | -0.20 | 0.04 | 0.05 |
| Q64521 | GPD2 | 80.953 | -1.30 | -1.32 | -1.31 | 0.02 | -1.26 | -1.45 | -1.36 | 0.13 | 0.05 |
| Q925N2 | SFXN2 | 36.141 | 0.07 | 0.02 | 0.04 | 0.03 | 0.05 | -0.06 | 0.00 | 0.08 | 0.05 |
| Q05915 | GCH1 | 27.014 | -0.42 | -0.40 | -0.41 | 0.01 | -0.51 | -0.40 | -0.45 | 0.08 | 0.05 |

| Q61316 | HSPA4 | 94.132 | -0.01 | 0.04 | 0.01 | 0.04 | 0.04 | -0.11 | -0.03 | 0.11 | 0.05 |
| --- | --- | --- | --- | --- | --- | --- | --- | --- | --- | --- | --- |
| Q9DBG7 | SRPR | 69.622 | 0.08 | 0.13 | 0.11 | 0.03 | -0.14 | 0.26 | 0.06 | 0.29 | 0.04 |
| Q9QWG7 | SULT1B1 | 34.901 | 0.31 | 0.22 | 0.27 | 0.06 | 0.19 | 0.26 | 0.22 | 0.05 | 0.04 |
| Q8JZQ9 | EIF3B | 91.369 | 0.16 | 0.07 | 0.12 | 0.06 | 0.15 | -0.01 | 0.07 | 0.11 | 0.04 |
| O35381 | ANP32A | 28.537 | 0.28 | 0.25 | 0.26 | 0.02 | 0.23 | 0.21 | 0.22 | 0.01 | 0.04 |
| O54879 | HMGB3 | 23.01 | -0.30 | -0.32 | -0.31 | 0.02 | -0.47 | -0.24 | -0.35 | 0.16 | 0.04 |
| P62900 | RPL31 | 14.463 | -0.04 | -0.04 | -0.04 | 0.00 | -0.11 | -0.06 | -0.09 | 0.04 | 0.04 |
| Q8VDJ3 | HDLBP | 141.74 | -0.29 | -0.07 | -0.18 | 0.16 | -0.41 | -0.03 | -0.22 | 0.27 | 0.04 |
| P35492 | HAL | 72.257 | -0.02 | -0.09 | -0.05 | 0.05 | -0.05 | -0.15 | -0.10 | 0.07 | 0.04 |
| P27641 | XRCC5 | 83.056 | 0.24 | 0.16 | 0.20 | 0.05 | 0.24 | 0.07 | 0.16 | 0.12 | 0.04 |
| Q8R317 | UBQLN1 | 61.976 | -0.38 | -0.11 | -0.24 | 0.19 | -0.41 | -0.16 | -0.29 | 0.17 | 0.04 |
| P30115 | GSTA3 | 25.36 | 0.11 | 0.12 | 0.11 | 0.01 | 0.08 | 0.07 | 0.07 | 0.00 | 0.04 |
| P84099 | RPL19 | 23.466 | 0.05 | -0.11 | -0.03 | 0.12 | -0.01 | -0.13 | -0.07 | 0.08 | 0.04 |

| Q64464 | CYP3A13 | 57.492 | 0.61 | 0.48 | 0.54 | 0.09 | 0.36 | 0.65 | 0.50 | 0.21 | 0.04 |
| --- | --- | --- | --- | --- | --- | --- | --- | --- | --- | --- | --- |
| Q91YW3 | DNAJC3 | 57.463 | -0.47 | -0.50 | -0.48 | 0.02 | -0.59 | -0.46 | -0.52 | 0.09 | 0.04 |
| P43274 | HIST1H1E | 21.977 | 0.82 | 0.66 | 0.74 | 0.11 | 0.79 | 0.61 | 0.70 | 0.13 | 0.04 |
| Q9EPE9 | ATP13A1 | 132.39 | -0.25 | 0.06 | -0.09 | 0.22 | -0.18 | -0.09 | -0.13 | 0.06 | 0.04 |
| Q9JIX8 | ACIN1 | 150.72 | -0.26 | 0.43 | 0.09 | 0.49 | -0.08 | 0.17 | 0.05 | 0.18 | 0.04 |
| P47199 | CRYZ | 35.268 | 0.03 | -0.12 | -0.05 | 0.11 | -0.04 | -0.13 | -0.09 | 0.07 | 0.04 |
| Q8BH69 | SEPHS1 | 42.906 | 0.25 | 0.09 | 0.17 | 0.11 | 0.10 | 0.16 | 0.13 | 0.04 | 0.04 |
| P14131 | RPS16 | 16.445 | 0.00 | 0.00 | 0.00 | 0.00 | -0.09 | 0.01 | -0.04 | 0.08 | 0.04 |
| Q9CR57 | RPL14 | 23.564 | -0.09 | -0.03 | -0.06 | 0.04 | -0.17 | -0.03 | -0.10 | 0.10 | 0.04 |
| P13634 | CA1 | 28.33 | 2.33 | 2.28 | 2.30 | 0.03 | 2.19 | 2.34 | 2.26 | 0.10 | 0.04 |
| P12367 | PRKAR2A | 45.389 | -0.04 | -0.04 | -0.04 | 0.00 | -0.19 | 0.03 | -0.08 | 0.16 | 0.04 |
| P35585 | AP1M1 | 48.542 | 0.22 | 0.12 | 0.17 | 0.07 | 0.25 | 0.02 | 0.13 | 0.17 | 0.04 |
| P97494 | GCLC | 72.571 | -0.38 | -0.57 | -0.48 | 0.14 | -0.49 | -0.54 | -0.51 | 0.04 | 0.04 |

| Q8K2C9 | HACD3 | 43.131 | -0.15 | -0.10 | -0.12 | 0.03 | -0.16 | -0.16 | -0.16 | 0.00 | 0.04 |
| --- | --- | --- | --- | --- | --- | --- | --- | --- | --- | --- | --- |
| Q9DBG6 | RPN2 | 69.062 | 0.10 | 0.03 | 0.06 | 0.06 | -0.02 | 0.07 | 0.03 | 0.07 | 0.04 |
| Q9CY50 | SSR1 | 32.065 | 0.16 | -0.06 | 0.05 | 0.16 | 0.05 | -0.02 | 0.01 | 0.05 | 0.04 |
| B2RQC6 | CAD | 243.24 | -0.52 | -0.34 | -0.43 | 0.13 | -0.35 | -0.59 | -0.47 | 0.17 | 0.04 |
| O54988 | SLK | 141.46 | -0.35 | 0.21 | -0.07 | 0.39 | -0.39 | 0.17 | -0.11 | 0.40 | 0.04 |
| O89112 | LANCL1 | 45.341 | 0.59 | 0.48 | 0.54 | 0.08 | 0.51 | 0.49 | 0.50 | 0.01 | 0.04 |
| O35114 | SCARB2 | 54.043 | 0.83 | 1.01 | 0.92 | 0.12 | 0.89 | 0.87 | 0.88 | 0.02 | 0.04 |
| P28230 | GJB1 | 32.003 | -0.39 | -0.41 | -0.40 | 0.01 | -0.70 | -0.17 | -0.44 | 0.38 | 0.04 |
| Q8BI84 | MIA3 | 213.67 | -0.31 | 0.31 | 0.00 | 0.44 | -0.56 | 0.48 | -0.04 | 0.73 | 0.04 |
| Q80VP1 | EPN1 | 60.211 | 0.05 | 0.52 | 0.28 | 0.33 | -0.07 | 0.57 | 0.25 | 0.45 | 0.04 |
| P49222 | EPB42 | 76.755 | 1.08 | 0.86 | 0.97 | 0.16 | 1.06 | 0.81 | 0.93 | 0.17 | 0.04 |
| P39447 | TJP1 | 194.74 | -0.90 | 0.13 | -0.38 | 0.73 | -0.91 | 0.08 | -0.42 | 0.70 | 0.03 |
| P22892 | AP1G1 | 91.349 | 0.17 | 0.00 | 0.09 | 0.12 | 0.07 | 0.04 | 0.05 | 0.02 | 0.03 |

| Q9DBG3 | AP2B1 | 104.58 | 0.10 | 0.35 | 0.22 | 0.17 | 0.10 | 0.28 | 0.19 | 0.13 | 0.03 |
| --- | --- | --- | --- | --- | --- | --- | --- | --- | --- | --- | --- |
| Q6ZWN5 | RPS9 | 22.591 | 0.02 | -0.03 | -0.01 | 0.03 | -0.05 | -0.03 | -0.04 | 0.01 | 0.03 |
| Q01768 | NME2 | 17.363 | -0.53 | -0.17 | -0.35 | 0.26 | -0.45 | -0.32 | -0.38 | 0.09 | 0.03 |
| Q9DBL7 | COASY | 62.022 | -0.11 | -0.13 | -0.12 | 0.01 | -0.25 | -0.05 | -0.15 | 0.14 | 0.03 |
| Q99MI1 | ERC1 | 128.33 | -0.27 | 0.43 | 0.08 | 0.49 | -0.35 | 0.45 | 0.05 | 0.57 | 0.03 |
| Q9D0R2 | TARS | 83.355 | -0.30 | -0.38 | -0.34 | 0.05 | -0.44 | -0.30 | -0.37 | 0.09 | 0.03 |
| P53811 | PITPNB | 31.487 | -0.23 | -0.19 | -0.21 | 0.03 | -0.19 | -0.29 | -0.24 | 0.07 | 0.03 |
| Q9CZR8 | TSFM | 35.334 | 0.08 | -0.05 | 0.02 | 0.09 | -0.11 | 0.08 | -0.02 | 0.13 | 0.03 |
| Q61490 | ALCAM | 65.091 | -0.05 | 0.22 | 0.08 | 0.19 | 0.08 | 0.03 | 0.05 | 0.03 | 0.03 |
| Q8K0D5 | GFM1 | 83.549 | -0.32 | -0.32 | -0.32 | 0.00 | -0.41 | -0.29 | -0.35 | 0.08 | 0.03 |
| Q9QXK3 | COPG2 | 97.679 | 0.05 | 0.00 | 0.02 | 0.04 | -0.01 | -0.01 | -0.01 | 0.00 | 0.03 |
| Q3U3R4 | LMF1 | 65.877 | -0.09 | -0.15 | -0.12 | 0.04 | -0.27 | -0.03 | -0.15 | 0.17 | 0.03 |
| P62301 | RPS13 | 17.222 | -0.09 | 0.00 | -0.04 | 0.06 | -0.09 | -0.05 | -0.07 | 0.03 | 0.03 |

| P35980 | RPL18 | 21.644 | 0.05 | -0.43 | -0.19 | 0.34 | -0.04 | -0.39 | -0.22 | 0.25 | 0.03 |
| --- | --- | --- | --- | --- | --- | --- | --- | --- | --- | --- | --- |
| P14206 | RPSA | 32.838 | 0.01 | -0.23 | -0.11 | 0.17 | -0.21 | -0.06 | -0.13 | 0.11 | 0.03 |
| P61255 | RPL26 | 17.258 | -0.02 | -0.05 | -0.04 | 0.02 | -0.08 | -0.05 | -0.07 | 0.02 | 0.03 |
| Q8BP67 | RPL24 | 17.779 | -0.08 | 0.03 | -0.03 | 0.08 | -0.19 | 0.08 | -0.05 | 0.19 | 0.03 |
| P70266 | PFKFB1 | 54.849 | -0.96 | -0.95 | -0.96 | 0.01 | -1.17 | -0.80 | -0.98 | 0.26 | 0.03 |
| P37040 | POR | 77.043 | 0.66 | 0.60 | 0.63 | 0.05 | 0.59 | 0.62 | 0.61 | 0.03 | 0.03 |
| P47738 | ALDH2 | 56.537 | 0.52 | 0.29 | 0.40 | 0.16 | 0.42 | 0.34 | 0.38 | 0.06 | 0.03 |
| Q9D1R9 | RPL34 | 13.293 | -0.01 | -0.11 | -0.06 | 0.07 | -0.05 | -0.13 | -0.09 | 0.05 | 0.03 |
| Q8R5H1 | USP15 | 112.32 | -0.62 | -0.19 | -0.41 | 0.30 | -0.48 | -0.38 | -0.43 | 0.07 | 0.03 |
| Q60866 | PTER | 39.218 | -0.40 | -0.54 | -0.47 | 0.10 | -0.47 | -0.51 | -0.49 | 0.03 | 0.03 |
| Q9DB16 | CAB39L | 39.105 | -0.24 | -0.21 | -0.22 | 0.02 | -0.20 | -0.30 | -0.25 | 0.07 | 0.03 |
| Q8R311 | CTAGE5 | 87.717 | -0.39 | 0.20 | -0.10 | 0.42 | -0.52 | 0.28 | -0.12 | 0.56 | 0.02 |
| P47955 | RPLP1 | 11.475 | -0.10 | -0.08 | -0.09 | 0.01 | -0.18 | -0.04 | -0.11 | 0.10 | 0.02 |

| O35459 | ECH1 | 36.118 | -0.23 | -0.34 | -0.29 | 0.08 | -0.32 | -0.30 | -0.31 | 0.01 | 0.02 |
| --- | --- | --- | --- | --- | --- | --- | --- | --- | --- | --- | --- |
| Q02357 | ANK1 | 204.22 | 0.59 | 0.90 | 0.75 | 0.21 | 0.49 | 0.96 | 0.72 | 0.34 | 0.02 |
| P63037 | DNAJA1 | 44.868 | -0.27 | -0.45 | -0.36 | 0.13 | -0.38 | -0.39 | -0.38 | 0.00 | 0.02 |
| Q80X85 | MRPS7 | 28.062 | 0.00 | 0.12 | 0.06 | 0.09 | 0.01 | 0.06 | 0.04 | 0.03 | 0.02 |
| Q8CC86 | NAPRT | 58.265 | -0.34 | -0.17 | -0.26 | 0.12 | -0.35 | -0.22 | -0.28 | 0.09 | 0.02 |
| Q6PDI5 | ECM29 | 203.7 | -0.20 | -0.05 | -0.12 | 0.10 | -0.13 | -0.15 | -0.14 | 0.01 | 0.02 |
| Q8VDG5 | PPCS | 33.794 | -0.30 | -0.52 | -0.41 | 0.15 | -0.34 | -0.52 | -0.43 | 0.13 | 0.02 |
| Q8C0D5 | EFTUD1 | 125.78 | -0.35 | 0.06 | -0.15 | 0.29 | -0.24 | -0.09 | -0.17 | 0.11 | 0.02 |
| Q8CCJ3 | UFL1 | 89.519 | -0.08 | -0.24 | -0.16 | 0.11 | -0.21 | -0.14 | -0.18 | 0.05 | 0.02 |
| P48776 | TDO2 | 47.756 | 1.17 | 1.12 | 1.15 | 0.04 | 1.05 | 1.20 | 1.13 | 0.11 | 0.02 |
| P05977 | MYL1 | 20.594 | -5.25 | -5.20 | -5.22 | 0.04 | -5.30 | -5.18 | -5.24 | 0.08 | 0.02 |
| Q62446 | FKBP3 | 25.147 | -0.07 | 0.11 | 0.02 | 0.13 | -0.04 | 0.04 | 0.00 | 0.06 | 0.02 |
| Q9CR16 | PPID | 40.742 | -0.13 | -0.24 | -0.18 | 0.08 | -0.24 | -0.16 | -0.20 | 0.05 | 0.02 |

| P14824 | ANXA6 | 75.884 | -0.06 | -0.04 | -0.05 | 0.02 | 0.09 | -0.22 | -0.06 | 0.22 | 0.02 |
| --- | --- | --- | --- | --- | --- | --- | --- | --- | --- | --- | --- |
| Q9CZG3 | COMMD8 | 20.852 | 0.17 | 0.29 | 0.23 | 0.09 | 0.21 | 0.22 | 0.21 | 0.01 | 0.02 |
| Q9CXW4 | RPL11 | 20.252 | -0.04 | 0.05 | 0.00 | 0.06 | 0.01 | -0.03 | -0.01 | 0.03 | 0.02 |
| Q8K2T1 | NMRAL1 | 34.376 | 0.07 | -0.02 | 0.02 | 0.07 | -0.03 | 0.05 | 0.01 | 0.06 | 0.01 |
| Q61699 | HSPH1 | 96.406 | -0.63 | -0.55 | -0.59 | 0.06 | -0.54 | -0.68 | -0.61 | 0.10 | 0.01 |
| Q9CR00 | PSMD9 | 24.72 | -0.03 | 0.03 | 0.00 | 0.05 | -0.02 | 0.00 | -0.01 | 0.02 | 0.01 |
| P97328 | KHK | 32.75 | -0.86 | -0.92 | -0.89 | 0.04 | -0.93 | -0.87 | -0.90 | 0.04 | 0.01 |
| Q8C3X2 | CCDC90B | 29.596 | 0.18 | 0.08 | 0.13 | 0.07 | 0.12 | 0.12 | 0.12 | 0.00 | 0.01 |
| Q9DBD0 | ICA | 76.765 | -0.01 | -0.07 | -0.04 | 0.04 | -0.12 | 0.02 | -0.05 | 0.10 | 0.01 |
| Q80WW9 | DDRGK1 | 35.977 | 0.03 | -0.30 | -0.14 | 0.23 | -0.31 | 0.01 | -0.15 | 0.23 | 0.01 |
| P61358 | RPL27 | 15.798 | -0.11 | -0.08 | -0.10 | 0.02 | -0.16 | -0.06 | -0.11 | 0.07 | 0.01 |
| P67984 | RPL22 | 14.759 | 0.00 | 0.04 | 0.02 | 0.03 | -0.05 | 0.06 | 0.00 | 0.07 | 0.01 |
| O88876 | DHRS3 | 33.652 | -0.94 | -0.58 | -0.76 | 0.25 | -0.82 | -0.72 | -0.77 | 0.07 | 0.01 |

HIST1H2BC;HIST2H2BB;HIST1H2BH;HIST1H2BM

| Q91YI0 | ASL | 51.739 | 0.53 | 0.28 | 0.41 | 0.18 | 0.35 | 0.44 | 0.39 | 0.06 | 0.01 |
| --- | --- | --- | --- | --- | --- | --- | --- | --- | --- | --- | --- |
| Q6ZWY9 |  | 13.906 | 0.25 | 0.56 | 0.40 | 0.22 | 0.58 | 0.21 | 0.39 | 0.27 | 0.01 |
| Q8BYH7 | TBC1D17 | 72.859 | 0.01 | 0.26 | 0.14 | 0.18 | 0.04 | 0.21 | 0.13 | 0.11 | 0.01 |
| Q9JKF7 | MRPL39 | 38.549 | 0.12 | -0.08 | 0.02 | 0.14 | -0.02 | 0.04 | 0.01 | 0.04 | 0.01 |
| Q7TMS5 | ABCG2 | 72.977 | 0.31 | 0.50 | 0.41 | 0.13 | 0.15 | 0.64 | 0.40 | 0.35 | 0.01 |
| Q570Y9 | DEPTOR | 46.119 | 0.03 | 0.14 | 0.08 | 0.08 | -0.35 | 0.49 | 0.07 | 0.59 | 0.01 |
| Q8JZZ0 | UGT3A2 | 59.672 | -0.26 | -0.35 | -0.30 | 0.06 | -0.38 | -0.24 | -0.31 | 0.10 | 0.01 |
| P24549 | ALDH1A1 | 54.467 | 1.00 | 1.01 | 1.00 | 0.00 | 0.94 | 1.05 | 0.99 | 0.07 | 0.01 |
| Q60960 | KPNA1 | 60.182 | -0.08 | 0.06 | -0.01 | 0.10 | -0.07 | 0.04 | -0.01 | 0.08 | 0.01 |
| Q9CQJ6 | DENR | 22.166 | 0.24 | 0.11 | 0.18 | 0.09 | 0.16 | 0.18 | 0.17 | 0.01 | 0.01 |
| Q3UMY5 | EML4 | 110.03 | 0.19 | 0.07 | 0.13 | 0.08 | 0.01 | 0.24 | 0.12 | 0.17 | 0.01 |
| Q9D051 | PDHB | 38.937 | -0.20 | -0.24 | -0.22 | 0.03 | -0.15 | -0.29 | -0.22 | 0.10 | 0.01 |
| Q9JLI6 | SCLY | 47.173 | -0.26 | -0.33 | -0.30 | 0.05 | -0.35 | -0.25 | -0.30 | 0.08 | 0.00 |

| Q9D3B1 | HACD2 | 28.402 | -0.29 | -0.44 | -0.36 | 0.10 | -0.52 | -0.22 | -0.37 | 0.21 | 0.00 |
| --- | --- | --- | --- | --- | --- | --- | --- | --- | --- | --- | --- |
| Q6PHQ8 | NAA35 | 83.305 | -0.25 | -0.13 | -0.19 | 0.09 | -0.20 | -0.18 | -0.19 | 0.01 | 0.00 |
| Q9DBA8 | AMDHD1 | 46.488 | -0.52 | -0.67 | -0.60 | 0.10 | -0.68 | -0.52 | -0.60 | 0.12 | 0.00 |
| Q9D404 | OXSM | 48.627 | 0.61 | 0.28 | 0.44 | 0.23 | 0.51 | 0.37 | 0.44 | 0.09 | 0.00 |
| Q9DBH5 | LMAN2 | 40.429 | -0.12 | -0.24 | -0.18 | 0.09 | -0.12 | -0.24 | -0.18 | 0.08 | 0.00 |
| P70694 | AKR1C6 | 37.047 | -0.05 | -0.27 | -0.16 | 0.16 | -0.05 | -0.28 | -0.16 | 0.16 | 0.00 |
| P23591 | TSTA3 | 35.877 | 0.01 | 0.03 | 0.02 | 0.01 | -0.05 | 0.09 | 0.02 | 0.10 | 0.00 |
| Q8VC28 | AKR1C13 | 37.057 | 0.03 | -0.04 | 0.00 | 0.05 | 0.03 | -0.04 | -0.01 | 0.05 | 0.00 |
| Q9QYC7 | GGCX | 87.194 | 0.24 | 0.26 | 0.25 | 0.01 | 0.04 | 0.46 | 0.25 | 0.30 | 0.00 |
| Q8R1Q9 | RBKS | 34.119 | 0.44 | 0.35 | 0.40 | 0.07 | 0.44 | 0.34 | 0.39 | 0.07 | 0.00 |
| Q9WV55 | VAPA | 27.855 | 0.29 | 0.23 | 0.26 | 0.04 | 0.20 | 0.32 | 0.26 | 0.08 | 0.00 |
| Q9DCX8 | IYD | 32.814 | 0.07 | -0.06 | 0.00 | 0.09 | 0.10 | -0.10 | 0.00 | 0.15 | 0.00 |
| P63323 | RPS12 | 14.525 | -0.04 | -0.03 | -0.03 | 0.01 | 0.01 | -0.08 | -0.04 | 0.07 | 0.00 |

| Q6GQT9 | NOMO1 | 133.42 | -0.14 | 0.22 | 0.04 | 0.26 | 0.02 | 0.05 | 0.04 | 0.02 | 0.00 |
| --- | --- | --- | --- | --- | --- | --- | --- | --- | --- | --- | --- |
| Q8CGC7 | EPRS | 170.08 | -0.31 | 0.02 | -0.14 | 0.24 | -0.23 | -0.06 | -0.15 | 0.12 | 0.00 |
| Q9JLJ4 | ELOVL2 | 34.207 | -1.84 | -1.68 | -1.76 | 0.11 | -1.67 | -1.86 | -1.76 | 0.14 | 0.00 |
| Q80ZS3 | MRPS26 | 23.443 | 0.15 | 0.14 | 0.14 | 0.01 | 0.17 | 0.11 | 0.14 | 0.04 | 0.00 |
| Q9D819 | PPA1 | 32.667 | 0.25 | -0.01 | 0.12 | 0.19 | 0.25 | 0.00 | 0.12 | 0.18 | 0.00 |
| Q8K021 | SCAMP1 | 38.028 | 0.46 | 0.44 | 0.45 | 0.01 | 0.35 | 0.55 | 0.45 | 0.14 | 0.00 |
| Q9D517 | AGPAT3 | 43.295 | -0.01 | -0.26 | -0.14 | 0.18 | -0.03 | -0.24 | -0.14 | 0.15 | 0.00 |
| Q9EQI8 | MRPL46 | 32.131 | 0.13 | 0.06 | 0.10 | 0.04 | 0.15 | 0.04 | 0.10 | 0.07 | 0.00 |
| Q91YH5 | ATL3 | 60.574 | 0.42 | 0.27 | 0.34 | 0.10 | 0.61 | 0.09 | 0.35 | 0.37 | 0.00 |
| Q8R1V4 | TMED4 | 26.022 | 0.08 | 0.35 | 0.22 | 0.19 | 0.26 | 0.18 | 0.22 | 0.06 | 0.00 |
| Q64105 | SPR | 27.883 | -0.19 | -0.16 | -0.17 | 0.02 | -0.16 | -0.18 | -0.17 | 0.02 | 0.00 |
| P62264 | RPS14 | 16.273 | -0.04 | -0.01 | -0.02 | 0.02 | -0.06 | 0.02 | -0.02 | 0.05 | 0.00 |
| P23475 | XRCC6 | 69.483 | 0.32 | 0.10 | 0.21 | 0.16 | 0.30 | 0.13 | 0.21 | 0.12 | 0.00 |

| Q61550 | RAD21 | 72.082 | -0.28 | 0.25 | -0.02 | 0.38 | -0.19 | 0.16 | -0.01 | 0.25 | 0.00 |
| --- | --- | --- | --- | --- | --- | --- | --- | --- | --- | --- | --- |
| P70441 | SLC9A3R1 | 38.6 | 0.18 | 0.05 | 0.12 | 0.09 | -0.03 | 0.27 | 0.12 | 0.22 | 0.00 |
| Q9EP75 | CYP4F14 | 59.8 | -0.36 | -0.39 | -0.38 | 0.03 | -0.47 | -0.28 | -0.37 | 0.13 | 0.00 |
| O09167 | RPL21 | 18.562 | -0.07 | -0.08 | -0.08 | 0.00 | -0.10 | -0.04 | -0.07 | 0.04 | 0.00 |
| Q80Y81 | ELAC2 | 92.718 | -0.18 | -0.23 | -0.20 | 0.04 | -0.30 | -0.09 | -0.20 | 0.15 | 0.00 |
| Q9D8E6 | RPL4 | 47.153 | 0.08 | -0.09 | 0.00 | 0.11 | 0.08 | -0.07 | 0.00 | 0.10 | -0.01 |
| P14148 | RPL7 | 31.419 | -0.15 | -0.11 | -0.13 | 0.03 | -0.15 | -0.09 | -0.12 | 0.04 | -0.01 |
| Q9QY76 | VAPB | 26.946 | 0.11 | 0.12 | 0.12 | 0.00 | 0.07 | 0.17 | 0.12 | 0.07 | -0.01 |
| Q99M87 | DNAJA3 | 52.443 | 0.13 | -0.09 | 0.02 | 0.15 | 0.22 | -0.16 | 0.03 | 0.27 | -0.01 |
| Q9DBK0 | ACOT12 | 61.761 | -0.34 | -0.25 | -0.29 | 0.07 | -0.31 | -0.26 | -0.29 | 0.04 | -0.01 |
| Q9JHL1 | SLC9A3R2 | 37.402 | -0.04 | -0.29 | -0.16 | 0.18 | -0.27 | -0.04 | -0.15 | 0.16 | -0.01 |
| Q91ZV0 | MIA2 | 57.776 | -0.87 | 0.60 | -0.14 | 1.04 | -0.83 | 0.58 | -0.13 | 1.00 | -0.01 |
| P62702 | RPS4X | 29.597 | -0.07 | 0.00 | -0.04 | 0.05 | -0.05 | -0.01 | -0.03 | 0.03 | -0.01 |

| P08113 | HSP90B1 | 92.475 | -0.22 | -0.25 | -0.24 | 0.02 | -0.20 | -0.25 | -0.23 | 0.04 | -0.01 |
| --- | --- | --- | --- | --- | --- | --- | --- | --- | --- | --- | --- |
| P47911 | RPL6 | 33.509 | 0.07 | -0.04 | 0.01 | 0.07 | 0.09 | -0.04 | 0.02 | 0.10 | -0.01 |
| Q9CQR2 | RPS21 | 9.1413 | 0.05 | 0.00 | 0.02 | 0.04 | -0.06 | 0.13 | 0.03 | 0.13 | -0.01 |
| P01942 | HBA | 15.085 | 0.72 | 0.74 | 0.73 | 0.01 | 0.75 | 0.74 | 0.74 | 0.01 | -0.01 |
| E9Q414 | APOB | 509.43 | -0.50 | -0.03 | -0.26 | 0.34 | -0.30 | -0.21 | -0.25 | 0.07 | -0.01 |
| Q3UMB9 | KIAA1033 | 136.37 | 0.18 | 0.36 | 0.27 | 0.12 | 0.28 | 0.28 | 0.28 | 0.00 | -0.01 |
| Q99N87 | MRPS5 | 48.206 | 0.24 | 0.00 | 0.12 | 0.17 | 0.21 | 0.05 | 0.13 | 0.12 | -0.01 |
| P97461 | RPS5 | 22.889 | 0.04 | 0.01 | 0.03 | 0.03 | 0.12 | -0.05 | 0.04 | 0.12 | -0.01 |
| P21619 | LMNB2 | 67.317 | 0.78 | 0.76 | 0.77 | 0.01 | 0.70 | 0.87 | 0.78 | 0.12 | -0.01 |
| P62889 | RPL30 | 12.784 | -0.13 | -0.02 | -0.08 | 0.07 | -0.15 | 0.02 | -0.06 | 0.12 | -0.01 |
| Q99K51 | PLS3 | 70.741 | 0.18 | 0.19 | 0.19 | 0.01 | 0.26 | 0.14 | 0.20 | 0.08 | -0.01 |
| P59913 | PCMTD1 | 40.693 | 0.20 | -0.04 | 0.08 | 0.17 | 0.01 | 0.18 | 0.10 | 0.12 | -0.01 |
| Q9CZ42 | CARKD | 36.717 | -0.04 | -0.19 | -0.12 | 0.10 | -0.14 | -0.06 | -0.10 | 0.06 | -0.01 |

| Q99KV1 | DNAJB11 | 40.555 | -0.04 | -0.13 | -0.08 | 0.07 | -0.06 | -0.08 | -0.07 | 0.01 | -0.01 |
| --- | --- | --- | --- | --- | --- | --- | --- | --- | --- | --- | --- |
| Q6P6M7 | SEPSECS | 55.325 | -0.83 | -1.09 | -0.96 | 0.18 | -0.91 | -0.98 | -0.94 | 0.05 | -0.01 |
| O35075 | DSCR3 | 32.97 | 0.42 | 0.26 | 0.34 | 0.11 | 0.43 | 0.28 | 0.35 | 0.10 | -0.01 |
| P00186 | CYP1A2 | 58.183 | 0.42 | 0.43 | 0.43 | 0.01 | 0.36 | 0.52 | 0.44 | 0.12 | -0.01 |
| P63276 | RPS17 | 15.524 | -0.12 | -0.06 | -0.09 | 0.05 | -0.10 | -0.05 | -0.07 | 0.04 | -0.02 |
| Q99N96 | MRPL1 | 37.596 | 0.12 | -0.20 | -0.04 | 0.22 | 0.11 | -0.16 | -0.02 | 0.19 | -0.02 |
| A6X935 | ITIH4 | 104.66 | 0.07 | 0.83 | 0.45 | 0.54 | 0.05 | 0.89 | 0.47 | 0.60 | -0.02 |
| O08709 | PRDX6 | 24.87 | -0.03 | -0.05 | -0.04 | 0.02 | -0.02 | -0.04 | -0.03 | 0.01 | -0.02 |
| P47915 | RPL29 | 17.587 | -0.01 | -0.15 | -0.08 | 0.09 | -0.03 | -0.10 | -0.07 | 0.05 | -0.02 |
| O88569 |  | 37.402 | 0.39 | 0.20 | 0.30 | 0.14 | 0.39 | 0.23 | 0.31 | 0.11 | -0.02 |
| P50431 | SHMT1 | 52.6 | -0.83 | -0.95 | -0.89 | 0.08 | -0.90 | -0.85 | -0.88 | 0.04 | -0.02 |
| Q9EPU0 | UPF1 | 123.97 | -0.54 | -0.08 | -0.31 | 0.32 | -0.50 | -0.08 | -0.29 | 0.30 | -0.02 |
| Q9Z1Z0 | USO1 | 106.98 | 0.07 | 0.02 | 0.05 | 0.04 | 0.12 | 0.01 | 0.07 | 0.08 | -0.02 |

| O35728 | CYP4A14 | 58.719 | 1.38 | 1.14 | 1.26 | 0.17 | 1.23 | 1.32 | 1.28 | 0.06 | -0.02 |
| --- | --- | --- | --- | --- | --- | --- | --- | --- | --- | --- | --- |
| P61022 | CHP1 | 22.432 | 0.48 | 0.48 | 0.48 | 0.00 | 0.49 | 0.51 | 0.50 | 0.01 | -0.02 |
| Q14CH1 | MOCOS | 95.012 | 0.05 | 0.20 | 0.12 | 0.10 | 0.16 | 0.12 | 0.14 | 0.02 | -0.02 |
| P62911 | RPL32 | 15.86 | -0.13 | -0.26 | -0.19 | 0.09 | 0.03 | -0.39 | -0.18 | 0.30 | -0.02 |
| Q61133 | GSTT2 | 27.634 | 0.51 | 0.41 | 0.46 | 0.07 | 0.39 | 0.57 | 0.48 | 0.13 | -0.02 |
| O88829 | ST3GAL5 | 47.359 | 1.24 | 1.37 | 1.31 | 0.09 | 1.33 | 1.33 | 1.33 | 0.00 | -0.02 |
| Q9D0J8 | PTMS | 11.43 | 0.07 | 0.08 | 0.07 | 0.01 | 0.08 | 0.11 | 0.09 | 0.02 | -0.02 |
| Q8BVI4 | QDPR | 25.57 | -0.13 | -0.06 | -0.10 | 0.05 | -0.09 | -0.06 | -0.07 | 0.02 | -0.02 |
| P16546 | SPTAN1 | 284.59 | -0.03 | 0.30 | 0.13 | 0.24 | 0.07 | 0.24 | 0.15 | 0.12 | -0.02 |
| Q9JKJ9 | CYP39A1 | 53.574 | -1.80 | -1.98 | -1.89 | 0.13 | -1.86 | -1.87 | -1.87 | 0.01 | -0.02 |
| P07310 | CKM | 43.044 | -4.87 | -4.95 | -4.91 | 0.06 | -4.94 | -4.83 | -4.88 | 0.08 | -0.02 |
| Q9D0I9 | RARS | 75.673 | 0.02 | 0.08 | 0.05 | 0.05 | 0.17 | -0.03 | 0.07 | 0.14 | -0.02 |
| A3KGB4 | TBC1D8B | 127.89 | -0.48 | -0.19 | -0.33 | 0.20 | -0.34 | -0.28 | -0.31 | 0.05 | -0.02 |

|  |  |  | 0.19 | 0.45 | 0.32 | 0.18 | 0.20 | 0.48 | 0.34 | 0.20 | -0.02 |
| --- | --- | --- | --- | --- | --- | --- | --- | --- | --- | --- | --- |
| Q8VDQ1 | PTGR2 | 38.015 | -0.33 | -0.32 | -0.33 | 0.00 | -0.31 | -0.29 | -0.30 | 0.01 | -0.02 |
| Q8K4Z3 | APOA1BP | 30.972 | 0.00 | -0.01 | 0.00 | 0.00 | -0.02 | 0.06 | 0.02 | 0.06 | -0.02 |
| Q6IRU5 | CLTB | 25.171 | 0.50 | 0.42 | 0.46 | 0.05 | 0.53 | 0.44 | 0.49 | 0.06 | -0.02 |
| Q9QZX7 | SRR | 36.358 | 0.05 | -0.04 | 0.01 | 0.06 | -0.04 | 0.10 | 0.03 | 0.10 | -0.02 |
| Q9QYG0 | NDRG2 | 40.789 | -0.27 | -0.49 | -0.38 | 0.16 | -0.29 | -0.42 | -0.36 | 0.09 | -0.02 |
| O55029 | COPB2 | 102.45 | 0.13 | 0.11 | 0.12 | 0.02 | 0.15 | 0.14 | 0.14 | 0.00 | -0.02 |
| P26231 | CTNNA1 | 100.11 | 0.42 | 0.32 | 0.37 | 0.07 | 0.47 | 0.32 | 0.40 | 0.11 | -0.02 |
| P59242 | CGN | 136.45 | -0.66 | -0.29 | -0.47 | 0.27 | -0.70 | -0.20 | -0.45 | 0.36 | -0.03 |
| Q3TMH2 | SCRN3 | 47.661 | -0.03 | -0.18 | -0.10 | 0.11 | -0.16 | 0.00 | -0.08 | 0.11 | -0.03 |
| Q9CQX2 | CYB5B | 16.318 | -0.19 | 0.06 | -0.06 | 0.18 | -0.02 | -0.06 | -0.04 | 0.03 | -0.03 |
| Q91WU5 | AS3MT | 41.793 | 0.06 | -0.13 | -0.04 | 0.13 | 0.05 | -0.08 | -0.01 | 0.09 | -0.03 |
| Q3TJZ6 | FAM98A | 55.055 | -0.09 | 0.09 | 0.00 | 0.12 | -0.12 | 0.17 | 0.03 | 0.21 | -0.03 |

| Q8K1Z0 | COQ9 | 35.082 | -0.20 | -0.27 | -0.24 | 0.05 | -0.20 | -0.22 | -0.21 | 0.01 | -0.03 |
| --- | --- | --- | --- | --- | --- | --- | --- | --- | --- | --- | --- |
| Q9D5T0 | ATAD1 | 40.744 | 0.13 | -0.08 | 0.02 | 0.14 | 0.05 | 0.05 | 0.05 | 0.00 | -0.03 |
| P20029 | HSPA5 | 72.421 | -0.08 | -0.26 | -0.17 | 0.13 | -0.08 | -0.21 | -0.15 | 0.09 | -0.03 |
| Q9EPL8 | IPO7 | 119.49 | -0.64 | -0.20 | -0.42 | 0.31 | -0.37 | -0.41 | -0.39 | 0.03 | -0.03 |
| Q9EPK7 | XPO7 | 123.81 | -0.10 | -0.30 | -0.20 | 0.14 | -0.08 | -0.27 | -0.17 | 0.14 | -0.03 |
| Q8BWQ6 |  | 109.08 | 0.10 | 0.35 | 0.22 | 0.18 | 0.30 | 0.20 | 0.25 | 0.08 | -0.03 |
| P49615 | CDK5 | 33.288 | -0.05 | 0.03 | -0.01 | 0.06 | 0.10 | -0.06 | 0.02 | 0.11 | -0.03 |
| P47857 | PFKM | 85.268 | -1.01 | -1.02 | -1.01 | 0.00 | -1.03 | -0.94 | -0.98 | 0.06 | -0.03 |
| Q9Z2Y8 | PROSC | 30.048 | 0.41 | 0.28 | 0.35 | 0.09 | 0.39 | 0.36 | 0.38 | 0.02 | -0.03 |
| O70194 | EIF3D | 63.988 | -0.09 | 0.07 | -0.01 | 0.11 | 0.11 | -0.06 | 0.02 | 0.12 | -0.03 |
| P62270 | RPS18 | 17.718 | -0.10 | 0.02 | -0.04 | 0.08 | -0.07 | 0.06 | -0.01 | 0.09 | -0.03 |
| Q8VDM6 | HNRNPUL1 | 96.001 | 0.01 | 0.35 | 0.18 | 0.24 | 0.40 | 0.01 | 0.21 | 0.28 | -0.03 |
| O35643 | AP1B1 | 103.93 | -0.11 | 0.09 | -0.01 | 0.14 | -0.15 | 0.19 | 0.02 | 0.24 | -0.03 |

| P15508 | SPTB | 245.25 | 0.42 | 0.89 | 0.65 | 0.33 | 0.56 | 0.82 | 0.69 | 0.18 | -0.03 |
| --- | --- | --- | --- | --- | --- | --- | --- | --- | --- | --- | --- |
| Q8VHE0 | SEC63 | 87.869 | 0.33 | 0.20 | 0.26 | 0.09 | 0.38 | 0.22 | 0.30 | 0.11 | -0.03 |
| P28271 | ACO1 | 98.124 | -0.09 | -0.14 | -0.11 | 0.03 | 0.00 | -0.16 | -0.08 | 0.11 | -0.03 |
| P14152 | MDH1 | 36.511 | -0.06 | -0.20 | -0.13 | 0.10 | -0.02 | -0.16 | -0.09 | 0.10 | -0.03 |
| O70251 | EEF1B | 24.693 | 0.13 | 0.02 | 0.07 | 0.07 | 0.13 | 0.09 | 0.11 | 0.03 | -0.04 |
| Q80XL6 | ACAD11 | 87.365 | 0.09 | 0.06 | 0.08 | 0.02 | 0.08 | 0.14 | 0.11 | 0.04 | -0.04 |
| P61458 | PCBD1 | 11.985 | -0.63 | -0.53 | -0.58 | 0.08 | -0.56 | -0.52 | -0.54 | 0.02 | -0.04 |
| P62082 | RPS7 | 22.127 | -0.10 | 0.05 | -0.03 | 0.11 | 0.00 | 0.02 | 0.01 | 0.01 | -0.04 |
| P08249 | MDH2 | 35.611 | 0.27 | 0.01 | 0.14 | 0.18 | 0.30 | 0.05 | 0.18 | 0.18 | -0.04 |
| Q61702 | ITIH1 | 101.07 | 0.15 | 0.37 | 0.26 | 0.16 | 0.03 | 0.56 | 0.30 | 0.37 | -0.04 |
| Q99LB2 | DHRS4 | 29.884 | 0.20 | 0.12 | 0.16 | 0.06 | 0.19 | 0.21 | 0.20 | 0.01 | -0.04 |
|  |  | 15.84 | 0.61 | 0.71 | 0.66 | 0.07 | 0.76 | 0.64 | 0.70 | 0.08 | -0.04 |
| P70349 | HINT1 | 13.777 | -0.48 | 0.02 | -0.23 | 0.35 | -0.21 | -0.16 | -0.19 | 0.03 | -0.04 |

| Q9QXS1 | PLEC | 534.18 | 0.47 | 0.85 | 0.66 | 0.27 | 0.59 | 0.82 | 0.70 | 0.17 | -0.04 |
| --- | --- | --- | --- | --- | --- | --- | --- | --- | --- | --- | --- |
| P11688 | ITGA5 | 115.04 | 0.03 | 0.53 | 0.28 | 0.35 | 0.19 | 0.45 | 0.32 | 0.18 | -0.04 |
| Q6P5E4 | UGGT1 | 176.43 | -0.29 | -0.02 | -0.15 | 0.19 | -0.16 | -0.06 | -0.11 | 0.07 | -0.04 |
| O55022 | PGRMC1 | 21.694 | 0.25 | 0.15 | 0.20 | 0.07 | 0.22 | 0.26 | 0.24 | 0.03 | -0.04 |
| Q76LS9 | FAM63A | 51.225 | 0.14 | 0.23 | 0.19 | 0.06 | 0.17 | 0.29 | 0.23 | 0.08 | -0.04 |
| Q8CG76 | AKR7A2 | 40.612 | 0.90 | 0.19 | 0.55 | 0.50 | 0.77 | 0.41 | 0.59 | 0.25 | -0.04 |
| P47962 | RPL5 | 34.4 | 0.08 | -0.11 | -0.01 | 0.13 | 0.11 | -0.06 | 0.03 | 0.12 | -0.04 |
| P97823 | LYPLA1 | 24.687 | -0.12 | -0.14 | -0.13 | 0.01 | -0.12 | -0.06 | -0.09 | 0.04 | -0.04 |
| P04919 | SLC4A1 | 103.13 | 0.71 | 0.84 | 0.78 | 0.09 | 0.84 | 0.80 | 0.82 | 0.03 | -0.04 |
| Q9QZE5 | COPG1 | 97.512 | 0.13 | 0.12 | 0.12 | 0.00 | 0.17 | 0.17 | 0.17 | 0.00 | -0.04 |
| Q8VC52 | RBPMS2 | 22.462 | -0.30 | -0.06 | -0.18 | 0.17 | -0.37 | 0.10 | -0.13 | 0.34 | -0.05 |
| Q8K411 | PITRM1 | 117.37 | 0.21 | 0.10 | 0.15 | 0.08 | 0.32 | 0.08 | 0.20 | 0.17 | -0.05 |
| Q9JHS4 | CLPX | 69.228 | -0.03 | -0.04 | -0.04 | 0.00 | -0.08 | 0.10 | 0.01 | 0.13 | -0.05 |

| O88343 | SLC4A4 | 121.48 | 0.25 | 0.63 | 0.44 | 0.27 | 0.41 | 0.56 | 0.49 | 0.10 | -0.05 |
| --- | --- | --- | --- | --- | --- | --- | --- | --- | --- | --- | --- |
| Q9JMD3 | STARD10 | 32.951 | 0.48 | 0.26 | 0.37 | 0.16 | 0.43 | 0.40 | 0.42 | 0.02 | -0.05 |
| Q9CQ65 | MTAP | 31.062 | -0.08 | -0.03 | -0.06 | 0.04 | -0.05 | 0.03 | -0.01 | 0.06 | -0.05 |
| P17427 | AP2A2 | 104.02 | 0.02 | 0.31 | 0.16 | 0.20 | 0.10 | 0.32 | 0.21 | 0.15 | -0.05 |
| P62245 | RPS15A | 14.839 | -0.22 | -0.02 | -0.12 | 0.14 | -0.11 | -0.03 | -0.07 | 0.06 | -0.05 |
| Q9QXY1 | TJP3 | 99.323 | -0.54 | -0.16 | -0.35 | 0.27 | -0.52 | -0.07 | -0.30 | 0.32 | -0.05 |
| Q8VBT2 | SDS | 34.593 | 1.12 | 0.94 | 1.03 | 0.12 | 1.12 | 1.05 | 1.08 | 0.05 | -0.05 |
| Q9JIF7 | COPB1 | 107.06 | -0.06 | 0.15 | 0.05 | 0.14 | 0.10 | 0.10 | 0.10 | 0.00 | -0.05 |
| O08547 | SEC22B | 24.74 | 0.03 | 0.15 | 0.09 | 0.08 | 0.02 | 0.27 | 0.14 | 0.18 | -0.05 |
| A2ADY9 | DDI2 | 44.59 | -0.19 | -0.28 | -0.23 | 0.07 | -0.20 | -0.17 | -0.18 | 0.02 | -0.05 |
| Q8CGY8 | OGT | 116.95 | -0.37 | -0.25 | -0.31 | 0.08 | -0.19 | -0.33 | -0.26 | 0.10 | -0.05 |
| Q8VDC0 | LARS2 | 101.48 | -0.26 | -0.22 | -0.24 | 0.03 | -0.14 | -0.23 | -0.18 | 0.07 | -0.05 |
| Q9JHI5 | IVD | 46.325 | 0.08 | 0.08 | 0.08 | 0.01 | 0.09 | 0.18 | 0.13 | 0.06 | -0.05 |

| P15105 | GLUL | 42.119 | -0.39 | -0.52 | -0.46 | 0.10 | -0.39 | -0.42 | -0.40 | 0.02 | -0.05 |
| --- | --- | --- | --- | --- | --- | --- | --- | --- | --- | --- | --- |
| O88441 | MTX2 | 29.758 | -0.01 | -0.11 | -0.06 | 0.07 | -0.04 | 0.03 | -0.01 | 0.05 | -0.06 |
| Q9JK38 | GNPNAT1 | 20.791 | -0.02 | -0.06 | -0.04 | 0.03 | 0.12 | -0.10 | 0.01 | 0.16 | -0.06 |
| O88587 | COMT | 29.486 | -0.04 | -0.07 | -0.05 | 0.02 | -0.08 | 0.09 | 0.00 | 0.11 | -0.06 |
| O54734 | DDOST | 49.027 | 0.01 | 0.00 | 0.01 | 0.00 | 0.06 | 0.07 | 0.06 | 0.00 | -0.06 |
| Q8VCC1 | HPGD | 29.18 | -1.18 | -1.17 | -1.18 | 0.01 | -1.26 | -0.98 | -1.12 | 0.20 | -0.06 |
| Q9EQ06 | HSD17B11 | 32.88 | -0.20 | -0.51 | -0.35 | 0.22 | -0.29 | -0.29 | -0.29 | 0.00 | -0.06 |
| P62830 | RPL23 | 14.865 | -0.23 | 0.06 | -0.09 | 0.20 | -0.13 | 0.08 | -0.03 | 0.15 | -0.06 |
| P23249 | MOV10 | 113.58 | -0.40 | -0.37 | -0.39 | 0.03 | -0.30 | -0.36 | -0.33 | 0.04 | -0.06 |
| O08756 | HSD17B10 | 27.418 | 0.12 | 0.20 | 0.16 | 0.06 | 0.16 | 0.29 | 0.23 | 0.09 | -0.06 |
| Q78JT3 | HAAO | 32.804 | 0.02 | -0.18 | -0.08 | 0.14 | -0.04 | -0.01 | -0.02 | 0.02 | -0.06 |
| Q9D826 | PIPOX | 43.846 | 0.15 | 0.16 | 0.15 | 0.00 | 0.15 | 0.28 | 0.21 | 0.09 | -0.06 |
| Q9WTP6 | AK2 | 26.468 | -0.29 | -0.43 | -0.36 | 0.10 | -0.29 | -0.30 | -0.30 | 0.01 | -0.06 |

| P61961 | UFM1 | 9.1175 | -0.58 | -0.26 | -0.42 | 0.22 | -0.40 | -0.31 | -0.36 | 0.06 | -0.06 |
| --- | --- | --- | --- | --- | --- | --- | --- | --- | --- | --- | --- |
| P05201 | GOT1 | 46.247 | 0.48 | 0.50 | 0.49 | 0.02 | 0.44 | 0.67 | 0.55 | 0.17 | -0.06 |
| P16460 | ASS1 | 46.584 | 0.27 | 0.20 | 0.23 | 0.05 | 0.28 | 0.31 | 0.30 | 0.03 | -0.06 |
| Q8CIE6 | COPA | 138.43 | -0.20 | 0.11 | -0.05 | 0.22 | -0.09 | 0.12 | 0.02 | 0.15 | -0.06 |
| P21278 | GNA11 | 42.024 | 0.17 | -0.08 | 0.04 | 0.18 | 0.16 | 0.06 | 0.11 | 0.07 | -0.06 |
| Q7TT50 | CDC42BPB | 194.75 | 0.02 | 0.28 | 0.15 | 0.19 | 0.12 | 0.31 | 0.22 | 0.13 | -0.06 |
| Q8R2Y8 | PTRH2 | 19.526 | 0.20 | 0.08 | 0.14 | 0.09 | 0.24 | 0.17 | 0.20 | 0.05 | -0.06 |
| P63325 | RPS10 | 18.916 | -0.27 | -0.07 | -0.17 | 0.14 | -0.16 | -0.06 | -0.11 | 0.08 | -0.07 |
| Q64374 | RGN | 33.406 | -0.39 | -0.57 | -0.48 | 0.13 | -0.31 | -0.52 | -0.42 | 0.15 | -0.07 |
| Q8BMA6 | SRP68 | 70.573 | -0.23 | -0.12 | -0.17 | 0.07 | -0.08 | -0.14 | -0.11 | 0.04 | -0.07 |
| Q91X52 | DCXR | 25.746 | 0.13 | 0.01 | 0.07 | 0.08 | 0.16 | 0.10 | 0.13 | 0.04 | -0.07 |
| Q99KI0 | ACO2 | 85.462 | 0.05 | -0.09 | -0.02 | 0.09 | 0.09 | 0.00 | 0.05 | 0.07 | -0.07 |
| Q8BVA5 |  | 37.374 | 0.18 | 0.01 | 0.09 | 0.12 | 0.14 | 0.18 | 0.16 | 0.02 | -0.07 |

| O89079 | COPE | 34.567 | 0.23 | 0.03 | 0.13 | 0.14 | 0.26 | 0.13 | 0.20 | 0.09 | -0.07 |
| --- | --- | --- | --- | --- | --- | --- | --- | --- | --- | --- | --- |
| Q64213 | SF1 | 70.402 | -0.39 | 0.23 | -0.08 | 0.44 | -0.14 | 0.11 | -0.02 | 0.18 | -0.07 |
| Q99KU0 | VMP1 | 45.96 | -0.21 | -0.23 | -0.22 | 0.01 | -0.27 | -0.03 | -0.15 | 0.17 | -0.07 |
| Q9EP96 |  | 73.964 | 0.30 | 0.23 | 0.26 | 0.05 | 0.25 | 0.42 | 0.34 | 0.12 | -0.07 |
| Q8R0Y6 | ALDH1L1 | 98.708 | -0.16 | -0.24 | -0.20 | 0.06 | -0.07 | -0.18 | -0.13 | 0.08 | -0.07 |
| Q9CXI5 | MANF | 20.373 | -0.50 | -0.34 | -0.42 | 0.12 | -0.37 | -0.32 | -0.35 | 0.04 | -0.07 |
| Q8CI33 | CWF19L1 | 60.193 | 0.05 | 0.26 | 0.15 | 0.15 | 0.08 | 0.37 | 0.22 | 0.21 | -0.07 |
| Q9DC71 | MRPS15 | 29.463 | -0.06 | 0.04 | -0.01 | 0.07 | 0.12 | 0.00 | 0.06 | 0.08 | -0.07 |
| Q8R1G2 | CMBL | 27.902 | 0.14 | 0.04 | 0.09 | 0.07 | 0.20 | 0.13 | 0.16 | 0.04 | -0.07 |
| Q9DBT9 | DMGDH | 97.254 | 0.56 | 0.50 | 0.53 | 0.04 | 0.47 | 0.74 | 0.60 | 0.20 | -0.07 |
| Q8BGH2 | SAMM50 | 51.863 | -0.40 | -0.09 | -0.24 | 0.22 | -0.27 | -0.07 | -0.17 | 0.14 | -0.07 |
| Q8VCN5 | CTH | 43.567 | 1.27 | 1.23 | 1.25 | 0.03 | 1.28 | 1.37 | 1.32 | 0.06 | -0.07 |
| O55023 | IMPA1 | 30.436 | -0.62 | 0.46 | -0.08 | 0.76 | 0.02 | -0.03 | 0.00 | 0.03 | -0.08 |

| Q9DBB8 | DHDH | 36.3 | 0.13 | -0.07 | 0.03 | 0.14 | 0.13 | 0.08 | 0.10 | 0.03 | -0.08 |
| --- | --- | --- | --- | --- | --- | --- | --- | --- | --- | --- | --- |
| B2RX12 | ABCC3 | 169.12 | 1.61 | 1.94 | 1.78 | 0.23 | 1.78 | 1.93 | 1.85 | 0.11 | -0.08 |
| Q9DCV4 | RMDN1 | 35 | -0.04 | -0.18 | -0.11 | 0.10 | -0.07 | 0.01 | -0.03 | 0.06 | -0.08 |
| Q8R146 | APEH | 81.58 | -0.23 | -0.17 | -0.20 | 0.04 | -0.15 | -0.09 | -0.12 | 0.04 | -0.08 |
| O09173 | HGD | 49.959 | -0.09 | -0.22 | -0.16 | 0.09 | 0.01 | -0.17 | -0.08 | 0.12 | -0.08 |
| P26043 | RDX | 68.542 | -0.17 | -0.15 | -0.16 | 0.01 | -0.21 | 0.05 | -0.08 | 0.18 | -0.08 |
| Q8K1R3 | PNPT1 | 85.682 | 0.01 | 0.03 | 0.02 | 0.02 | -0.02 | 0.22 | 0.10 | 0.17 | -0.08 |
| Q99JB8 | PACSIN3 | 48.584 | 0.67 | 0.48 | 0.57 | 0.13 | 0.49 | 0.82 | 0.65 | 0.24 | -0.08 |
| Q6DFW4 | NOP58 | 60.342 | 0.37 | 0.44 | 0.40 | 0.05 | 0.55 | 0.41 | 0.48 | 0.10 | -0.08 |
| Q9Z2I0 | LETM1 | 82.988 | -0.10 | -0.13 | -0.11 | 0.02 | -0.15 | 0.09 | -0.03 | 0.17 | -0.08 |
| Q3UQ44 | IQGAP2 | 180.53 | 0.07 | 0.30 | 0.18 | 0.16 | 0.10 | 0.43 | 0.27 | 0.23 | -0.08 |
| P46978 | STT3A | 80.597 | 0.04 | -0.04 | 0.00 | 0.06 | 0.01 | 0.15 | 0.08 | 0.09 | -0.08 |
| Q9CRA5 | GOLPH3 | 33.752 | 0.22 | 0.23 | 0.22 | 0.00 | 0.23 | 0.39 | 0.31 | 0.11 | -0.08 |

| Q6ZWV7 | RPL35 | 14.552 | -0.25 | -0.10 | -0.18 | 0.10 | -0.08 | -0.10 | -0.09 | 0.02 | -0.08 |
| --- | --- | --- | --- | --- | --- | --- | --- | --- | --- | --- | --- |
| Q8BGC4 | ZADH2 | 40.528 | -0.12 | -0.26 | -0.19 | 0.10 | -0.04 | -0.18 | -0.11 | 0.10 | -0.08 |
| P30416 | FKBP4 | 51.572 | -0.93 | -1.14 | -1.03 | 0.14 | -1.03 | -0.87 | -0.95 | 0.11 | -0.08 |
| Q61247 | SERPINF2 | 54.971 | -0.49 | 0.08 | -0.21 | 0.40 | -0.25 | 0.01 | -0.12 | 0.18 | -0.08 |
| Q9CY27 | TECR | 36.09 | -0.30 | -0.20 | -0.25 | 0.07 | -0.21 | -0.11 | -0.16 | 0.07 | -0.09 |
| Q8R519 | ACMSD | 38.027 | 1.65 | 1.64 | 1.65 | 0.01 | 1.71 | 1.76 | 1.73 | 0.03 | -0.09 |
| O89023 | TPP1 | 61.341 | 1.69 | 1.39 | 1.54 | 0.22 | 1.69 | 1.56 | 1.62 | 0.10 | -0.09 |
| Q9D1M0 | SEC13 | 35.565 | 0.40 | 0.17 | 0.29 | 0.16 | 0.41 | 0.33 | 0.37 | 0.06 | -0.09 |
| P13412 | TNNI2 | 21.357 | -4.99 | -4.89 | -4.94 | 0.07 | -4.92 | -4.79 | -4.86 | 0.09 | -0.09 |
| Q8BW75 | MAOB | 58.557 | -0.08 | -0.17 | -0.12 | 0.06 | -0.05 | -0.02 | -0.04 | 0.02 | -0.09 |
| Q6ZWY3 | RPS27L | 9.4771 | -0.05 | 0.08 | 0.01 | 0.09 | -0.01 | 0.21 | 0.10 | 0.16 | -0.09 |
| Q91Y97 | ALDOB | 39.507 | -0.56 | -0.83 | -0.70 | 0.19 | -0.45 | -0.77 | -0.61 | 0.23 | -0.09 |
| Q99K67 | AASS | 102.97 | 0.68 | 1.20 | 0.94 | 0.37 | 0.84 | 1.20 | 1.02 | 0.25 | -0.09 |

| Q9CPR4 | RPL17 | 21.423 | -0.23 | 0.02 | -0.10 | 0.18 | -0.10 | 0.07 | -0.02 | 0.12 | -0.09 |
| --- | --- | --- | --- | --- | --- | --- | --- | --- | --- | --- | --- |
| Q9QXE0 | HACL1 | 63.66 | -0.52 | -0.65 | -0.59 | 0.09 | -0.46 | -0.54 | -0.50 | 0.06 | -0.09 |
| Q64435 |  |  | 0.46 | 0.10 | 0.28 | 0.26 | 0.43 | 0.32 | 0.38 | 0.08 | -0.09 |
| P70697 | UROD | 40.691 | -0.23 | -0.31 | -0.27 | 0.06 | -0.17 | -0.18 | -0.17 | 0.00 | -0.10 |
| P28660 | NCKAP1 | 128.78 | -0.02 | 0.36 | 0.17 | 0.27 | 0.29 | 0.24 | 0.27 | 0.03 | -0.10 |
| O08810 | EFTUD2 | 109.36 | -0.20 | 0.38 | 0.09 | 0.41 | 0.22 | 0.15 | 0.19 | 0.05 | -0.10 |
| Q3UHN9 | NDST1 | 100.72 | 0.02 | 0.40 | 0.21 | 0.27 | 0.28 | 0.33 | 0.30 | 0.04 | -0.10 |
| B7ZMP1 | XPNPEP3 | 56.677 | -0.37 | -0.07 | -0.22 | 0.21 | -0.07 | -0.17 | -0.12 | 0.07 | -0.10 |
| P97364 | SEPHS2 | 47.833 | 0.33 | 0.14 | 0.23 | 0.13 | 0.24 | 0.42 | 0.33 | 0.12 | -0.10 |
| P35293 | RAB18 | 23.035 | 0.29 | 0.25 | 0.27 | 0.03 | 0.52 | 0.22 | 0.37 | 0.21 | -0.10 |
| Q62186 | SSR4 | 18.936 | 0.03 | -0.12 | -0.05 | 0.10 | -0.07 | 0.17 | 0.05 | 0.17 | -0.10 |
| A2ALW5 | DNAJC25 | 41.937 | -0.13 | -0.42 | -0.28 | 0.20 | -0.13 | -0.23 | -0.18 | 0.07 | -0.10 |
| Q9D5J6 | SHPK | 51.302 | -0.38 | -0.52 | -0.45 | 0.10 | -0.28 | -0.43 | -0.35 | 0.11 | -0.10 |

| Q8C7X2 | EMC1 | 111.6 | -0.24 | 0.08 | -0.08 | 0.23 | 0.05 | -0.02 | 0.02 | 0.05 | -0.10 |
| --- | --- | --- | --- | --- | --- | --- | --- | --- | --- | --- | --- |
| P99027 | RPLP2 | 11.651 | -0.31 | -0.14 | -0.23 | 0.12 | -0.17 | -0.08 | -0.12 | 0.07 | -0.10 |
| Q6ZQI3 | MLEC | 32.342 | 0.42 | 0.34 | 0.38 | 0.06 | 0.43 | 0.54 | 0.48 | 0.08 | -0.10 |
| P26443 | GLUD1 | 61.336 | -0.08 | -0.25 | -0.16 | 0.13 | -0.08 | -0.04 | -0.06 | 0.03 | -0.10 |
| Q9JLZ3 | AUH | 33.395 | 0.23 | 0.34 | 0.29 | 0.08 | 0.36 | 0.42 | 0.39 | 0.04 | -0.10 |
| Q9CW42 | 42064 | 37.978 | 0.17 | 0.15 | 0.16 | 0.01 | 0.26 | 0.26 | 0.26 | 0.00 | -0.10 |
| Q9D2G2 | DLST | 48.994 | -0.27 | -0.23 | -0.25 | 0.03 | -0.31 | 0.02 | -0.15 | 0.24 | -0.11 |
| Q91VS7 | MGST1 | 17.551 | 0.51 | 0.64 | 0.58 | 0.09 | 0.63 | 0.74 | 0.68 | 0.08 | -0.11 |
| Q9D8N0 | EEF1G | 50.06 | 0.15 | 0.00 | 0.08 | 0.11 | 0.26 | 0.11 | 0.18 | 0.10 | -0.11 |
| P06909 | CFH | 139.14 | -0.04 | 0.27 | 0.11 | 0.22 | 0.14 | 0.31 | 0.22 | 0.12 | -0.11 |
| Q9JJJ3 | AQP9 | 31.764 | 0.97 | 0.67 | 0.82 | 0.21 | 1.17 | 0.70 | 0.93 | 0.33 | -0.11 |
| Q91WT9 | CBS | 61.543 | 0.35 | 0.36 | 0.35 | 0.00 | 0.42 | 0.51 | 0.46 | 0.06 | -0.11 |
| Q922R8 | PDIA6 | 48.1 | -0.13 | -0.30 | -0.22 | 0.12 | -0.03 | -0.19 | -0.11 | 0.11 | -0.11 |

| Q60676 | PPP5C | 56.876 | -0.07 | -0.12 | -0.09 | 0.04 | 0.00 | 0.03 | 0.02 | 0.02 | -0.11 |
| --- | --- | --- | --- | --- | --- | --- | --- | --- | --- | --- | --- |
| Q60759 | GCDH | 48.605 | -0.05 | -0.19 | -0.12 | 0.10 | -0.15 | 0.12 | -0.01 | 0.19 | -0.11 |
| P15208 | INSR | 155.61 | -0.33 | 0.31 | -0.01 | 0.45 | -0.13 | 0.33 | 0.10 | 0.33 | -0.11 |
| Q8VCW8 | ACSF2 | 67.95 | 0.38 | 0.34 | 0.36 | 0.03 | 0.40 | 0.54 | 0.47 | 0.10 | -0.11 |
| Q9DBG1 | CYP27A1 | 60.719 | 1.00 | 0.99 | 0.99 | 0.01 | 1.00 | 1.21 | 1.10 | 0.15 | -0.11 |
| Q9D898 | ARPC5L | 16.98 | 0.33 | 0.32 | 0.33 | 0.01 | 0.40 | 0.47 | 0.44 | 0.05 | -0.11 |
| Q8VC97 | UPB1 | 43.937 | 0.97 | 0.92 | 0.95 | 0.04 | 1.04 | 1.08 | 1.06 | 0.03 | -0.11 |
| P42925 | PXMP2 | 22.265 | -0.07 | -0.10 | -0.09 | 0.02 | 0.04 | 0.01 | 0.02 | 0.02 | -0.11 |
| P50247 | AHCY | 47.688 | 0.14 | 0.07 | 0.10 | 0.05 | 0.15 | 0.27 | 0.21 | 0.08 | -0.11 |
| P24270 | CAT | 59.795 | -0.05 | -0.22 | -0.13 | 0.12 | 0.01 | -0.05 | -0.02 | 0.05 | -0.11 |
| P42669 | PURA | 34.883 | 0.26 | 0.27 | 0.27 | 0.00 | 0.24 | 0.52 | 0.38 | 0.19 | -0.11 |
| Q8BH00 | ALDH8A1 | 53.663 | -0.35 | -0.40 | -0.37 | 0.04 | -0.29 | -0.23 | -0.26 | 0.05 | -0.11 |
| O88291 | ZNF326 | 65.225 | -0.02 | 0.03 | 0.01 | 0.04 | 0.09 | 0.16 | 0.12 | 0.05 | -0.12 |

| Q2TPA8 | HSDL2 | 54.208 | -0.40 | -0.12 | -0.26 | 0.20 | -0.12 | -0.17 | -0.14 | 0.04 | -0.12 |
| --- | --- | --- | --- | --- | --- | --- | --- | --- | --- | --- | --- |
| P16406 | ENPEP | 107.96 | -0.82 | -0.37 | -0.59 | 0.32 | -0.68 | -0.27 | -0.48 | 0.29 | -0.12 |
| Q9CQ06 | MRPL24 | 24.944 | -0.07 | 0.00 | -0.04 | 0.05 | 0.00 | 0.16 | 0.08 | 0.11 | -0.12 |
| Q91X91 | QPRT | 31.53 | -0.08 | -0.21 | -0.15 | 0.09 | 0.09 | -0.15 | -0.03 | 0.17 | -0.12 |
| P24456 |  | 57.233 | -0.12 | 0.13 | 0.01 | 0.18 | 0.19 | 0.06 | 0.12 | 0.09 | -0.12 |
| Q9JHW2 | NIT2 | 30.501 | -0.56 | -0.52 | -0.54 | 0.03 | -0.43 | -0.41 | -0.42 | 0.01 | -0.12 |
| Q9DC61 | PMPCA | 58.278 | -0.17 | 0.01 | -0.08 | 0.13 | -0.17 | 0.25 | 0.04 | 0.29 | -0.12 |
| P51174 | ACADL | 47.907 | -0.40 | -0.45 | -0.43 | 0.04 | -0.27 | -0.33 | -0.30 | 0.05 | -0.12 |
| Q61823 | PDCD4 | 51.702 | 0.35 | 0.28 | 0.32 | 0.05 | 0.49 | 0.39 | 0.44 | 0.07 | -0.12 |
| P97742 | CPT1A | 88.25 | -0.11 | -0.15 | -0.13 | 0.03 | -0.05 | 0.04 | -0.01 | 0.06 | -0.12 |
| P62751 | RPL23A | 17.695 | -0.39 | -0.05 | -0.22 | 0.24 | -0.13 | -0.06 | -0.10 | 0.05 | -0.12 |
| P59325 | EIF5 | 48.968 | 0.04 | 0.03 | 0.04 | 0.01 | 0.08 | 0.24 | 0.16 | 0.12 | -0.12 |
| Q60931 | VDAC3 | 30.752 | 0.17 | 0.24 | 0.21 | 0.05 | 0.33 | 0.34 | 0.33 | 0.01 | -0.13 |

| Q91ZJ5 | UGP2 | 56.979 | -1.30 | -1.17 | -1.24 | 0.09 | -1.24 | -0.99 | -1.11 | 0.17 | -0.13 |
| --- | --- | --- | --- | --- | --- | --- | --- | --- | --- | --- | --- |
| Q8QZT1 | ACAT1 | 44.816 | -0.56 | -0.82 | -0.69 | 0.19 | -0.56 | -0.56 | -0.56 | 0.00 | -0.13 |
| P51658 | HSD17B2 | 41.835 | -0.72 | -0.81 | -0.77 | 0.06 | -0.56 | -0.72 | -0.64 | 0.12 | -0.13 |
| Q9D110 | MTHFS | 23.201 | -0.05 | 0.08 | 0.02 | 0.09 | 0.22 | 0.07 | 0.14 | 0.11 | -0.13 |
| Q91WS0 | CISD1 | 12.097 | -0.23 | -0.04 | -0.13 | 0.14 | -0.06 | 0.04 | -0.01 | 0.07 | -0.13 |
| P45700 | MAN1A1 | 73.275 | -0.16 | -0.04 | -0.10 | 0.09 | 0.08 | -0.03 | 0.03 | 0.08 | -0.13 |
| Q8VI47 | ABCC2 | 173.67 | 0.29 | 0.42 | 0.35 | 0.09 | 0.38 | 0.58 | 0.48 | 0.14 | -0.13 |
| O08914 | FAAH | 63.221 | -0.07 | -0.14 | -0.10 | 0.04 | 0.02 | 0.03 | 0.02 | 0.01 | -0.13 |
| Q8BZ09 | SLC25A21 | 33.228 | -0.03 | -0.14 | -0.08 | 0.07 | 0.12 | -0.03 | 0.04 | 0.11 | -0.13 |
| Q9DCM2 | GSTK1 | 25.704 | 0.35 | 0.34 | 0.35 | 0.00 | 0.44 | 0.51 | 0.48 | 0.05 | -0.13 |
| P99029 | PRDX5 | 21.897 | -0.11 | 0.37 | 0.13 | 0.34 | 0.17 | 0.35 | 0.26 | 0.13 | -0.13 |
| Q8CFX1 | H6PD | 88.927 | 0.39 | 0.42 | 0.41 | 0.02 | 0.53 | 0.54 | 0.54 | 0.01 | -0.13 |
| O35658 | C1QBP | 31.013 | 0.51 | 0.34 | 0.42 | 0.12 | 0.53 | 0.58 | 0.56 | 0.04 | -0.13 |

| P32020 | SCP2 | 59.125 | 1.34 | 0.52 | 0.93 | 0.58 | 1.43 | 0.69 | 1.06 | 0.52 | -0.13 |
| --- | --- | --- | --- | --- | --- | --- | --- | --- | --- | --- | --- |
| P23492 | PNP | 32.277 | -0.09 | -0.14 | -0.11 | 0.04 | -0.01 | 0.05 | 0.02 | 0.04 | -0.13 |
| Q9R112 | SQRDL | 50.282 | -0.04 | -0.21 | -0.13 | 0.12 | -0.07 | 0.08 | 0.00 | 0.10 | -0.13 |
| Q99KR3 | LACTB2 | 32.754 | -0.10 | -0.18 | -0.14 | 0.06 | -0.03 | 0.01 | -0.01 | 0.02 | -0.13 |
| Q9CQE1 | NIPSNAP3B | 28.308 | 0.06 | -0.19 | -0.06 | 0.18 | 0.00 | 0.15 | 0.07 | 0.10 | -0.13 |
| Q8CAY6 | ACAT2 | 41.297 | 0.13 | -0.19 | -0.03 | 0.23 | 0.16 | 0.05 | 0.10 | 0.08 | -0.14 |
| Q91XE4 | ACY3 | 35.286 | -0.53 | -0.55 | -0.54 | 0.01 | -0.34 | -0.47 | -0.41 | 0.09 | -0.14 |
| P47758 | SRPRB | 29.579 | 0.06 | 0.18 | 0.12 | 0.08 | 0.17 | 0.34 | 0.26 | 0.12 | -0.14 |
| Q8K157 | GALM | 37.798 | -0.12 | -0.30 | -0.21 | 0.12 | -0.06 | -0.08 | -0.07 | 0.02 | -0.14 |
| P47802 | MTX1 | 35.623 | -0.19 | -0.24 | -0.22 | 0.04 | -0.11 | -0.05 | -0.08 | 0.05 | -0.14 |
| Q9CWX2 | NDUFAF1 | 37.809 | -0.09 | -0.22 | -0.15 | 0.09 | -0.08 | 0.05 | -0.01 | 0.09 | -0.14 |
| Q9Z2Z6 | SLC25A20 | 33.026 | 0.03 | -0.12 | -0.04 | 0.11 | 0.08 | 0.11 | 0.10 | 0.02 | -0.14 |
| Q61646 | HP | 38.752 | 2.15 | 2.49 | 2.32 | 0.24 | 2.29 | 2.63 | 2.46 | 0.24 | -0.14 |

| Q64310 | SURF4 | 30.381 | -0.06 | -0.12 | -0.09 | 0.04 | 0.14 | -0.03 | 0.06 | 0.11 | -0.14 |
| --- | --- | --- | --- | --- | --- | --- | --- | --- | --- | --- | --- |
| Q9D0G0 | MRPS30 | 49.939 | 0.01 | -0.07 | -0.03 | 0.05 | 0.26 | -0.03 | 0.11 | 0.20 | -0.14 |
| Q8BMJ2 | LARS | 134.19 | -0.35 | 0.12 | -0.11 | 0.33 | 0.01 | 0.04 | 0.03 | 0.02 | -0.14 |
| Q8BWM0 | PTGES2 | 43.323 | 0.14 | 0.05 | 0.09 | 0.06 | 0.18 | 0.29 | 0.24 | 0.08 | -0.14 |
| Q9JKX3 | TFR2 | 88.401 | 0.43 | 0.37 | 0.40 | 0.04 | 0.44 | 0.65 | 0.54 | 0.15 | -0.14 |
| Q71KU9 | FGL1 | 36.439 | 1.53 | 1.44 | 1.49 | 0.07 | 1.61 | 1.65 | 1.63 | 0.03 | -0.15 |
| Q920A5 | SCPEP1 | 50.964 | 0.65 | 0.67 | 0.66 | 0.01 | 0.73 | 0.88 | 0.80 | 0.10 | -0.15 |
| Q9DB29 | IAH1 | 27.974 | -0.36 | -0.32 | -0.34 | 0.02 | -0.22 | -0.17 | -0.19 | 0.03 | -0.15 |
| Q9D6Z1 | NOP56 | 64.464 | 0.38 | 0.44 | 0.41 | 0.04 | 0.59 | 0.53 | 0.56 | 0.04 | -0.15 |
| Q8BH95 | ECHS1 | 31.474 | -0.03 | -0.01 | -0.02 | 0.01 | 0.09 | 0.16 | 0.13 | 0.05 | -0.15 |
| Q8R4N0 | CLYBL | 37.548 | 0.32 | 0.18 | 0.25 | 0.10 | 0.40 | 0.39 | 0.40 | 0.00 | -0.15 |
| Q91XF0 | PNPO | 30.114 | 0.30 | 0.37 | 0.34 | 0.05 | 0.44 | 0.53 | 0.49 | 0.07 | -0.15 |
| O09111 | NDUFB11 | 17.444 | 0.28 | -0.09 | 0.09 | 0.26 | 0.11 | 0.37 | 0.24 | 0.18 | -0.15 |

| Q91VT4 | CBR4 | 25.414 | -0.07 | -0.05 | -0.06 | 0.02 | 0.05 | 0.13 | 0.09 | 0.05 | -0.15 |
| --- | --- | --- | --- | --- | --- | --- | --- | --- | --- | --- | --- |
| Q60575 |  | 204.08 | 0.14 | 0.19 | 0.17 | 0.03 | 0.40 | 0.24 | 0.32 | 0.11 | -0.15 |
| Q9D6Y7 | MSRA | 25.988 | -0.03 | 0.00 | -0.01 | 0.02 | 0.10 | 0.18 | 0.14 | 0.06 | -0.15 |
| Q8K2I4 | MANBA | 100.83 | 0.74 | 1.04 | 0.89 | 0.21 | 0.90 | 1.19 | 1.05 | 0.21 | -0.15 |
| Q9JHW4 | EEFSEC | 63.538 | 0.26 | 0.40 | 0.33 | 0.10 | 0.48 | 0.49 | 0.48 | 0.01 | -0.15 |
| Q9CPV4 | GLOD4 | 33.316 | -0.06 | -0.07 | -0.06 | 0.01 | 0.08 | 0.09 | 0.09 | 0.01 | -0.15 |
| Q9WUA2 | FARSB | 65.696 | 0.08 | 0.12 | 0.10 | 0.03 | 0.37 | 0.13 | 0.25 | 0.17 | -0.15 |
| P61211 | ARL1 | 20.411 | 0.12 | 0.22 | 0.17 | 0.07 | 0.28 | 0.38 | 0.33 | 0.07 | -0.15 |
| Q99KB8 | HAGH | 34.084 | -0.44 | -0.39 | -0.41 | 0.04 | -0.32 | -0.20 | -0.26 | 0.08 | -0.15 |
| Q9CQ62 | DECR1 | 36.213 | -0.27 | -0.38 | -0.32 | 0.08 | -0.19 | -0.15 | -0.17 | 0.03 | -0.16 |
| Q3ULD5 | MCCC2 | 61.378 | 0.07 | -0.09 | -0.01 | 0.11 | 0.10 | 0.19 | 0.14 | 0.06 | -0.16 |
| Q9JM62 | REEP6 | 22.204 | 0.11 | 0.32 | 0.22 | 0.15 | 0.37 | 0.38 | 0.37 | 0.01 | -0.16 |
| Q8BYL4 | YARS2 | 52.597 | 0.13 | -0.12 | 0.01 | 0.18 | 0.02 | 0.31 | 0.16 | 0.20 | -0.16 |

| Q8BSE0 | RMDN2 | 47.016 | -0.05 | -0.07 | -0.06 | 0.01 | 0.01 | 0.19 | 0.10 | 0.13 | -0.16 |
| --- | --- | --- | --- | --- | --- | --- | --- | --- | --- | --- | --- |
| Q8BFR5 | TUFM | 49.508 | -0.17 | -0.20 | -0.18 | 0.02 | -0.12 | 0.06 | -0.03 | 0.12 | -0.16 |
| O88451 | RDH7 | 35.66 | -0.16 | -0.20 | -0.18 | 0.03 | -0.04 | -0.01 | -0.02 | 0.02 | -0.16 |
| Q8CIF4 | BTD | 58.154 | 0.47 | 0.31 | 0.39 | 0.12 | 0.53 | 0.57 | 0.55 | 0.03 | -0.16 |
| P00920 | CA2 | 29.032 | 0.30 | 0.28 | 0.29 | 0.02 | 0.43 | 0.47 | 0.45 | 0.03 | -0.16 |
| Q9JLB0 | MPP6 | 62.63 | 0.01 | -0.18 | -0.08 | 0.13 | 0.06 | 0.09 | 0.08 | 0.02 | -0.16 |
| P27046 | MAN2A1 | 131.63 | 0.28 | 0.74 | 0.51 | 0.32 | 0.50 | 0.83 | 0.67 | 0.23 | -0.16 |
| Q6ZQ38 | CAND1 | 136.33 | -0.29 | 0.17 | -0.06 | 0.33 | 0.14 | 0.06 | 0.10 | 0.06 | -0.16 |
| O89020 | AFM | 69.378 | -0.38 | -0.57 | -0.47 | 0.14 | -0.18 | -0.44 | -0.31 | 0.18 | -0.16 |
| O35857 | TIMM44 | 51.091 | -0.25 | -0.29 | -0.27 | 0.02 | -0.21 | -0.01 | -0.11 | 0.14 | -0.16 |
| P24369 | PPIB | 23.713 | -0.32 | -0.25 | -0.29 | 0.04 | -0.16 | -0.09 | -0.12 | 0.05 | -0.16 |
| Q91VA6 | POLDIP2 | 41.87 | 0.09 | 0.06 | 0.07 | 0.02 | 0.03 | 0.44 | 0.24 | 0.29 | -0.16 |
| Q91WQ3 | YARS | 59.105 | -0.38 | -0.43 | -0.41 | 0.03 | -0.31 | -0.17 | -0.24 | 0.09 | -0.16 |

| O35129 | PHB2 | 33.296 | 0.12 | 0.04 | 0.08 | 0.06 | 0.27 | 0.22 | 0.24 | 0.04 | -0.16 |
| --- | --- | --- | --- | --- | --- | --- | --- | --- | --- | --- | --- |
| Q6PB66 | LRPPRC | 156.61 | -0.94 | -0.39 | -0.66 | 0.39 | -0.69 | -0.31 | -0.50 | 0.26 | -0.17 |
| Q9CR62 | SLC25A11 | 34.155 | -0.31 | -0.37 | -0.34 | 0.04 | -0.16 | -0.20 | -0.18 | 0.03 | -0.17 |
| P34914 | EPHX2 | 62.515 | 0.30 | 0.14 | 0.22 | 0.11 | 0.36 | 0.41 | 0.39 | 0.04 | -0.17 |
| Q9EQQ9 | MGEA5 | 103.16 | -0.18 | 0.35 | 0.08 | 0.38 | 0.44 | 0.06 | 0.25 | 0.27 | -0.17 |
| Q8BHN3 | GANAB | 106.91 | -0.16 | -0.09 | -0.13 | 0.05 | 0.08 | 0.00 | 0.04 | 0.06 | -0.17 |
| Q3UNZ8 |  | 37.808 | -0.04 | 0.08 | 0.02 | 0.08 | 0.15 | 0.23 | 0.19 | 0.06 | -0.17 |
| Q3UJU9 | RMDN3 | 52.028 | -0.46 | -0.16 | -0.31 | 0.21 | -0.48 | 0.20 | -0.14 | 0.49 | -0.17 |
| Q01279 | EGFR | 134.85 | 0.36 | 1.03 | 0.70 | 0.47 | 0.29 | 1.43 | 0.86 | 0.81 | -0.17 |
| Q8CHT0 | ALDH4A1 | 61.84 | -0.05 | -0.12 | -0.09 | 0.05 | 0.11 | 0.05 | 0.08 | 0.04 | -0.17 |
| Q8BWF0 | ALDH5A1 | 55.968 | -0.07 | -0.11 | -0.09 | 0.02 | 0.00 | 0.15 | 0.08 | 0.11 | -0.17 |
| Q91YT0 | NDUFV1 | 50.834 | 0.09 | 0.01 | 0.05 | 0.05 | 0.14 | 0.30 | 0.22 | 0.11 | -0.17 |
| Q8JZN7 | RHOT2 | 69.07 | 0.22 | -0.02 | 0.10 | 0.17 | 0.26 | 0.28 | 0.27 | 0.01 | -0.17 |

| Q8QZR1 | TAT | 50.565 | 2.65 | 2.68 | 2.66 | 0.02 | 2.74 | 2.93 | 2.83 | 0.13 | -0.17 |
| --- | --- | --- | --- | --- | --- | --- | --- | --- | --- | --- | --- |
| Q8K274 | FN3KRP | 34.468 | -0.39 | -0.09 | -0.24 | 0.21 | -0.15 | 0.03 | -0.06 | 0.13 | -0.17 |
| P15116 | CDH2 | 99.795 | 0.20 | 0.41 | 0.30 | 0.15 | 0.31 | 0.65 | 0.48 | 0.24 | -0.17 |
| Q9QYB1 | CLIC4 | 28.729 | 0.17 | 0.18 | 0.18 | 0.01 | 0.37 | 0.33 | 0.35 | 0.03 | -0.17 |
| P16015 | CA3 | 29.366 | -0.04 | -0.23 | -0.14 | 0.14 | 0.07 | 0.01 | 0.04 | 0.04 | -0.17 |
| Q9WVL0 | GSTZ1 | 24.275 | -0.32 | -0.31 | -0.32 | 0.01 | -0.22 | -0.06 | -0.14 | 0.12 | -0.17 |
| Q78PY7 | SND1 | 102.09 | 0.07 | 0.18 | 0.13 | 0.08 | 0.36 | 0.24 | 0.30 | 0.09 | -0.17 |
| A2AJL3 | FGGY | 60.336 | -0.70 | -0.69 | -0.69 | 0.01 | -0.60 | -0.43 | -0.52 | 0.12 | -0.18 |
| Q9ET01 | PYGL | 97.462 | -1.14 | -1.23 | -1.18 | 0.07 | -0.95 | -1.06 | -1.01 | 0.08 | -0.18 |
| E9Q735 | UBE4A | 118.2 | 0.06 | 0.37 | 0.22 | 0.22 | 0.37 | 0.42 | 0.39 | 0.04 | -0.18 |
| Q3U0B3 | DHRS11 | 28.274 | 0.32 | 0.37 | 0.34 | 0.04 | 0.56 | 0.48 | 0.52 | 0.05 | -0.18 |
| Q9D7B6 | ACAD8 | 45.019 | 0.21 | 0.00 | 0.10 | 0.15 | 0.27 | 0.29 | 0.28 | 0.01 | -0.18 |
| Q9D7J9 | ECHDC3 | 32.402 | 0.00 | 0.08 | 0.04 | 0.05 | 0.23 | 0.21 | 0.22 | 0.01 | -0.18 |

| Q8QZR3 | CES2A | 61.94 | 0.74 | 0.90 | 0.82 | 0.11 | 0.99 | 1.01 | 1.00 | 0.01 | -0.18 |
| --- | --- | --- | --- | --- | --- | --- | --- | --- | --- | --- | --- |
| Q05421 | CYP2E1 | 56.804 | 0.37 | 0.23 | 0.30 | 0.10 | 0.43 | 0.53 | 0.48 | 0.07 | -0.18 |
| Q9JKR6 | HYOU1 | 111.18 | -0.52 | -0.33 | -0.42 | 0.14 | -0.34 | -0.14 | -0.24 | 0.14 | -0.18 |
| Q80XN0 | BDH1 | 38.299 | -1.17 | -1.26 | -1.21 | 0.07 | -1.08 | -0.98 | -1.03 | 0.07 | -0.18 |
| P25688 | UOX | 35.039 | 0.40 | 0.26 | 0.33 | 0.10 | 0.59 | 0.44 | 0.51 | 0.11 | -0.18 |
| Q9CQS8 | SEC61B | 9.9583 | -0.84 | -0.56 | -0.70 | 0.19 | -0.30 | -0.73 | -0.52 | 0.30 | -0.18 |
| O70133 | DHX9 | 149.47 | -0.43 | 0.40 | -0.01 | 0.59 | 0.10 | 0.24 | 0.17 | 0.10 | -0.19 |
| Q91WD5 | NDUFS2 | 52.625 | 0.16 | -0.01 | 0.07 | 0.12 | 0.14 | 0.38 | 0.26 | 0.17 | -0.19 |
| Q91W52 | TMEM19 | 36.296 | 0.95 | 1.06 | 1.01 | 0.08 | 1.13 | 1.25 | 1.19 | 0.09 | -0.19 |
| P58281 | OPA1 | 111.34 | 0.16 | 0.01 | 0.09 | 0.11 | 0.26 | 0.28 | 0.27 | 0.01 | -0.19 |
| Q8K370 | ACAD10 | 118.98 | 0.00 | -0.01 | 0.00 | 0.00 | 0.16 | 0.20 | 0.18 | 0.03 | -0.19 |
| Q8VCR2 | HSD17B13 | 33.458 | 0.52 | 0.42 | 0.47 | 0.07 | 0.71 | 0.62 | 0.66 | 0.06 | -0.19 |
| Q91V64 | ISOC1 | 32.032 | -0.42 | -0.47 | -0.45 | 0.03 | -0.29 | -0.22 | -0.25 | 0.05 | -0.19 |

| P40630 | TFAM | 27.987 | -0.25 | -0.25 | -0.25 | 0.00 | -0.12 | 0.00 | -0.06 | 0.08 | -0.19 |
| --- | --- | --- | --- | --- | --- | --- | --- | --- | --- | --- | --- |
| Q99LP6 | GRPEL1 | 24.307 | -0.17 | -0.16 | -0.17 | 0.01 | -0.01 | 0.06 | 0.03 | 0.05 | -0.19 |
| Q62425 | NDUFA4 | 9.3267 | -0.18 | -0.15 | -0.17 | 0.02 | -0.04 | 0.10 | 0.03 | 0.10 | -0.20 |
| O08600 | ENDOG | 32.19 | -0.46 | -0.69 | -0.57 | 0.16 | -0.37 | -0.38 | -0.38 | 0.01 | -0.20 |
| Q8BJ64 | CHDH | 66.414 | 0.17 | -0.02 | 0.08 | 0.13 | 0.28 | 0.27 | 0.27 | 0.01 | -0.20 |
| P01872 | IGHM | 49.971 | 4.99 | 4.62 | 4.80 | 0.26 | 4.89 | 5.11 | 5.00 | 0.16 | -0.20 |
| P11352 | GPX1 | 22.329 | 0.75 | 0.91 | 0.83 | 0.11 | 0.93 | 1.13 | 1.03 | 0.14 | -0.20 |
| P01029 | C4B | 192.91 | -0.21 | 0.05 | -0.08 | 0.19 | -0.02 | 0.26 | 0.12 | 0.20 | -0.20 |
| Q63836 | SELENBP2 | 52.609 | 0.49 | 0.50 | 0.50 | 0.01 | 0.13 | 1.27 | 0.70 | 0.81 | -0.20 |
| P06802 | ENPP1 | 103.17 | 0.16 | 0.49 | 0.32 | 0.24 | 0.52 | 0.54 | 0.53 | 0.02 | -0.20 |
| P20918 | PLG | 90.807 | -0.31 | -0.29 | -0.30 | 0.01 | -0.13 | -0.06 | -0.10 | 0.05 | -0.20 |
| Q64726 | AZGP1 | 35.332 | 0.52 | 0.67 | 0.59 | 0.11 | 0.73 | 0.87 | 0.80 | 0.10 | -0.20 |
| P29391 | FTL1 | 20.802 | 1.52 | 1.93 | 1.72 | 0.29 | 1.62 | 2.24 | 1.93 | 0.44 | -0.20 |

| P97290 | SERPING1 | 55.584 | -0.16 | 0.14 | -0.01 | 0.21 | 0.10 | 0.29 | 0.19 | 0.14 | -0.20 |
| --- | --- | --- | --- | --- | --- | --- | --- | --- | --- | --- | --- |
| Q8C7H1 | MMAA | 45.932 | -0.19 | -0.31 | -0.25 | 0.08 | -0.11 | 0.02 | -0.04 | 0.09 | -0.21 |
| Q925I1 | ATAD3 | 66.741 | 0.08 | -0.01 | 0.03 | 0.06 | 0.24 | 0.23 | 0.24 | 0.01 | -0.21 |
| Q9D4H8 | CUL2 | 86.876 | -0.22 | -0.19 | -0.20 | 0.02 | -0.05 | 0.06 | 0.00 | 0.08 | -0.21 |
| P62843 | RPS15 | 17.04 | -0.36 | -0.06 | -0.21 | 0.21 | -0.12 | 0.11 | 0.00 | 0.17 | -0.21 |
| Q9CPY7 | LAP3 | 56.141 | 0.15 | 0.07 | 0.11 | 0.06 | 0.29 | 0.35 | 0.32 | 0.04 | -0.21 |
| Q9JLT4 | TXNRD2 | 56.602 | -0.07 | -0.18 | -0.13 | 0.08 | 0.08 | 0.08 | 0.08 | 0.00 | -0.21 |
| O88696 | CLPP | 29.8 | 0.45 | 0.26 | 0.36 | 0.13 | 0.54 | 0.59 | 0.57 | 0.04 | -0.21 |
| Q8VEM8 | SLC25A3 | 39.632 | -0.19 | -0.08 | -0.14 | 0.08 | 0.08 | 0.06 | 0.07 | 0.01 | -0.21 |
| Q9DAU1 | CNPY3 | 30.537 | -0.31 | -0.37 | -0.34 | 0.04 | -0.25 | -0.01 | -0.13 | 0.17 | -0.21 |
| Q61838 | A2M | 165.85 | 0.36 | 0.74 | 0.55 | 0.27 | 0.67 | 0.85 | 0.76 | 0.13 | -0.21 |
| Q922Q8 | LRRC59 | 34.877 | 0.19 | 0.12 | 0.16 | 0.05 | 0.39 | 0.35 | 0.37 | 0.03 | -0.21 |
| Q9EP89 | LACTB | 60.705 | 0.17 | 0.29 | 0.23 | 0.08 | 0.29 | 0.60 | 0.44 | 0.22 | -0.21 |

| P17717 | UGT2B17 | 60.855 | 0.50 | 0.39 | 0.44 | 0.08 | 0.58 | 0.74 | 0.66 | 0.11 | -0.21 |
| --- | --- | --- | --- | --- | --- | --- | --- | --- | --- | --- | --- |
| P61620 |  | 52.264 | -0.02 | -0.22 | -0.12 | 0.14 | 0.12 | 0.06 | 0.09 | 0.05 | -0.21 |
| P33267 | CYP2F2 | 55.948 | 0.42 | 0.13 | 0.27 | 0.20 | 0.55 | 0.42 | 0.49 | 0.09 | -0.21 |
| Q91VM9 | PPA2 | 38.114 | 0.42 | 0.21 | 0.32 | 0.15 | 0.61 | 0.46 | 0.53 | 0.11 | -0.21 |
| P58710 | GULO | 50.478 | 0.00 | -0.19 | -0.10 | 0.14 | 0.19 | 0.05 | 0.12 | 0.10 | -0.22 |
| P38647 | HSPA9 | 73.46 | -0.01 | -0.15 | -0.08 | 0.10 | 0.27 | 0.00 | 0.14 | 0.19 | -0.22 |
| P97493 | TXN2 | 18.255 | -0.29 | 0.07 | -0.11 | 0.26 | 0.04 | 0.18 | 0.11 | 0.10 | -0.22 |
| Q9DCG6 | PBLD1 | 32.047 | -0.12 | -0.22 | -0.17 | 0.07 | 0.06 | 0.04 | 0.05 | 0.02 | -0.22 |
| Q9D6M3 | SLC25A22 | 34.67 | 0.08 | -0.05 | 0.01 | 0.09 | 0.23 | 0.23 | 0.23 | 0.01 | -0.22 |
| Q99LC5 | ETFA | 35.009 | -0.14 | -0.27 | -0.21 | 0.09 | 0.02 | 0.00 | 0.01 | 0.02 | -0.22 |
| F6ZDS4 | TPR | 273.99 | 0.05 | 0.34 | 0.20 | 0.20 | 0.38 | 0.46 | 0.42 | 0.06 | -0.22 |
| Q9DB77 | UQCRC2 | 48.234 | -0.14 | -0.17 | -0.16 | 0.02 | -0.01 | 0.14 | 0.06 | 0.10 | -0.22 |
| Q91YJ5 | MTIF2 | 81.288 | 0.12 | 0.23 | 0.17 | 0.08 | 0.31 | 0.47 | 0.39 | 0.11 | -0.22 |

| Q8R4U0 | STAB2 | 277.53 | -0.34 | -0.32 | -0.33 | 0.01 | -0.07 | -0.14 | -0.11 | 0.05 | -0.22 |
| --- | --- | --- | --- | --- | --- | --- | --- | --- | --- | --- | --- |
| Q9DBC0 | SELO | 74.22 | 0.60 | 0.45 | 0.53 | 0.11 | 0.82 | 0.68 | 0.75 | 0.10 | -0.22 |
| Q99MR6 | SRRT | 100.45 | -0.25 | 0.28 | 0.01 | 0.37 | 0.31 | 0.16 | 0.24 | 0.11 | -0.22 |
| Q8CFA2 | AMT | 44.008 | -0.02 | -0.17 | -0.09 | 0.11 | 0.08 | 0.18 | 0.13 | 0.07 | -0.22 |
| Q4VA53 | PDS5B | 164.42 | -0.20 | 0.08 | -0.06 | 0.20 | 0.32 | 0.01 | 0.16 | 0.22 | -0.22 |
| P35505 | FAH | 46.175 | -0.32 | -0.20 | -0.26 | 0.09 | -0.10 | 0.04 | -0.03 | 0.10 | -0.23 |
| Q9DB26 | PHYHD1 | 32.517 | -0.17 | -0.18 | -0.17 | 0.01 | 0.13 | -0.02 | 0.05 | 0.11 | -0.23 |
| P29341 | PABPC1 | 70.67 | 0.21 | 0.13 | 0.17 | 0.05 | 0.84 | -0.04 | 0.40 | 0.62 | -0.23 |
| Q5SW19 | CLUH | 148.07 | -0.43 | -0.15 | -0.29 | 0.20 | -0.13 | 0.00 | -0.07 | 0.10 | -0.23 |
| Q9WUR2 | ECI2 | 43.267 | -0.15 | -0.28 | -0.21 | 0.09 | 0.03 | 0.00 | 0.01 | 0.02 | -0.23 |
| Q91XD4 | FTCD | 58.938 | -0.41 | -0.32 | -0.36 | 0.06 | -0.25 | -0.02 | -0.13 | 0.16 | -0.23 |
| Q8JZS0 | LIN7A | 25.992 | -0.21 | 0.04 | -0.09 | 0.18 | 0.00 | 0.30 | 0.15 | 0.21 | -0.23 |
| Q921X9 | PDIA5 | 59.266 | 0.21 | 0.18 | 0.19 | 0.02 | 0.35 | 0.50 | 0.42 | 0.11 | -0.23 |

| Q8VCB3 | GYS2 | 80.87 | -0.81 | -0.82 | -0.82 | 0.01 | -0.60 | -0.57 | -0.59 | 0.02 | -0.23 |
| --- | --- | --- | --- | --- | --- | --- | --- | --- | --- | --- | --- |
| Q60597 | OGDH | 116.45 | 0.19 | 0.13 | 0.16 | 0.04 | 0.49 | 0.30 | 0.40 | 0.13 | -0.24 |
| Q9CRB3 | URAH | 13.559 | -0.24 | -0.20 | -0.22 | 0.03 | -0.04 | 0.07 | 0.01 | 0.08 | -0.24 |
| Q8JZQ2 | AFG3L2 | 89.518 | -0.15 | -0.16 | -0.15 | 0.01 | -0.02 | 0.18 | 0.08 | 0.14 | -0.24 |
| Q9CZW5 | TOMM70A | 67.589 | -0.17 | -0.13 | -0.15 | 0.03 | 0.00 | 0.17 | 0.08 | 0.12 | -0.24 |
| P12710 | FABP1 | 14.245 | -1.45 | -1.17 | -1.31 | 0.19 | -1.07 | -1.07 | -1.07 | 0.00 | -0.24 |
| P67778 | PHB | 29.82 | 0.00 | -0.01 | 0.00 | 0.01 | 0.19 | 0.28 | 0.24 | 0.07 | -0.24 |
| Q8JZU2 | SLC25A1 | 33.931 | -0.11 | 0.10 | -0.01 | 0.15 | 0.19 | 0.27 | 0.23 | 0.06 | -0.24 |
| Q91WN4 | KMO | 54.531 | 0.34 | 0.29 | 0.31 | 0.03 | 0.62 | 0.48 | 0.55 | 0.10 | -0.24 |
| Q9ESB3 | HRG | 59.162 | 0.74 | 1.06 | 0.90 | 0.23 | 1.10 | 1.18 | 1.14 | 0.06 | -0.24 |
| O35386 | PHYH | 38.607 | 0.98 | 0.97 | 0.97 | 0.01 | 1.16 | 1.27 | 1.22 | 0.08 | -0.24 |
| P22907 | HMBS | 39.344 | -0.43 | -0.29 | -0.36 | 0.09 | -0.21 | -0.02 | -0.12 | 0.14 | -0.24 |
| P49429 | HPD | 45.054 | -0.14 | -0.25 | -0.20 | 0.08 | 0.02 | 0.07 | 0.05 | 0.04 | -0.24 |

| Q9DCS9 | NDUFB10 | 21.024 | 0.05 | -0.04 | 0.01 | 0.07 | 0.25 | 0.25 | 0.25 | 0.00 | -0.24 |
| --- | --- | --- | --- | --- | --- | --- | --- | --- | --- | --- | --- |
| Q9DCT2 | NDUFS3 | 30.149 | 0.22 | -0.01 | 0.10 | 0.17 | 0.33 | 0.37 | 0.35 | 0.02 | -0.25 |
| Q9D0M3 | CYC1 | 35.327 | -0.11 | -0.16 | -0.14 | 0.03 | 0.16 | 0.07 | 0.11 | 0.06 | -0.25 |
| Q9CWD8 | NUBPL | 34.139 | 0.04 | 0.00 | 0.02 | 0.03 | 0.08 | 0.46 | 0.27 | 0.27 | -0.25 |
| Q5SGK3 | AOX2 | 147.91 | 1.18 | 1.46 | 1.32 | 0.20 | 1.23 | 1.91 | 1.57 | 0.48 | -0.25 |
| P19783 | COX4I1 | 19.53 | -0.12 | -0.13 | -0.13 | 0.01 | 0.07 | 0.17 | 0.12 | 0.07 | -0.25 |
| Q9WTP7 | AK3 | 25.426 | 0.17 | 0.04 | 0.10 | 0.09 | 0.35 | 0.36 | 0.36 | 0.01 | -0.25 |
| Q5BL07 | PEX1 | 141.43 | -0.12 | 0.00 | -0.06 | 0.09 | 0.41 | -0.02 | 0.19 | 0.30 | -0.25 |
| Q9Z2I9 | SUCLA2 | 50.113 | -0.33 | -0.42 | -0.38 | 0.06 | -0.22 | -0.03 | -0.12 | 0.13 | -0.25 |
| P62075 | TIMM13 | 10.458 | -0.25 | -0.13 | -0.19 | 0.08 | 0.01 | 0.11 | 0.06 | 0.07 | -0.26 |
| Q9WV27 | ATP1A4 | 114.89 | 0.36 | 0.25 | 0.31 | 0.08 | 0.62 | 0.51 | 0.57 | 0.08 | -0.26 |
| Q99JB2 | STOML2 | 38.384 | 0.16 | -0.07 | 0.04 | 0.16 | 0.32 | 0.28 | 0.30 | 0.03 | -0.26 |
| Q64458 | CYP2C29 | 55.715 | 0.03 | -0.18 | -0.07 | 0.14 | 0.14 | 0.23 | 0.18 | 0.07 | -0.26 |

| Q9DBF1 | ALDH7A1 | 58.861 | 0.74 | 0.84 | 0.79 | 0.07 | 0.97 | 1.13 | 1.05 | 0.11 | -0.26 |
| --- | --- | --- | --- | --- | --- | --- | --- | --- | --- | --- | --- |
| P34927 | ASGR1 | 32.591 | -0.37 | -0.61 | -0.49 | 0.17 | -0.26 | -0.21 | -0.23 | 0.04 | -0.26 |
| P10630 | EIF4A2 | 46.402 | 0.03 | -0.08 | -0.03 | 0.08 | 0.25 | 0.22 | 0.23 | 0.02 | -0.26 |
| Q5XJY4 | PARL | 41.963 | 0.14 | -0.16 | -0.01 | 0.22 | 0.25 | 0.26 | 0.25 | 0.00 | -0.26 |
| Q9CPQ1 | COX6C | 8.4689 | -0.18 | -0.14 | -0.16 | 0.03 | 0.08 | 0.13 | 0.10 | 0.04 | -0.26 |
| P08228 | SOD1 | 15.942 | -0.76 | -0.16 | -0.46 | 0.43 | -0.27 | -0.12 | -0.19 | 0.11 | -0.26 |
| P53395 | DBT | 53.246 | 0.14 | -0.10 | 0.02 | 0.17 | 0.24 | 0.33 | 0.28 | 0.06 | -0.26 |
| Q8BUV3 | GPHN | 83.281 | -0.09 | -0.04 | -0.06 | 0.04 | 0.02 | 0.39 | 0.20 | 0.26 | -0.27 |
| P19536 | COX5B | 13.813 | -0.27 | -0.16 | -0.21 | 0.08 | 0.01 | 0.09 | 0.05 | 0.06 | -0.27 |
| Q8R3F5 | MCAT | 41.928 | 0.10 | 0.00 | 0.05 | 0.07 | 0.24 | 0.40 | 0.32 | 0.11 | -0.27 |
| Q4LDG0 | SLC27A5 | 76.202 | -0.08 | -0.06 | -0.07 | 0.02 | 0.23 | 0.17 | 0.20 | 0.04 | -0.27 |
| Q8VDN2 | ATP1A1 | 112.98 | 0.32 | 0.17 | 0.25 | 0.10 | 0.62 | 0.41 | 0.51 | 0.15 | -0.27 |
| Q03265 | ATP5A1 | 59.752 | -0.03 | -0.25 | -0.14 | 0.16 | 0.14 | 0.11 | 0.13 | 0.02 | -0.27 |

| Q80UM7 | MOGS | 91.83 | -0.33 | -0.33 | -0.33 | 0.00 | -0.11 | -0.02 | -0.06 | 0.06 | -0.27 |
| --- | --- | --- | --- | --- | --- | --- | --- | --- | --- | --- | --- |
| P39039 | MBL1 | 25.396 | -0.52 | -0.58 | -0.55 | 0.04 | -0.35 | -0.21 | -0.28 | 0.10 | -0.27 |
| Q61176 | ARG1 | 34.807 | 0.52 | 0.27 | 0.40 | 0.18 | 0.74 | 0.59 | 0.67 | 0.11 | -0.27 |
| P05202 | GOT2 | 47.411 | -0.03 | -0.21 | -0.12 | 0.12 | 0.23 | 0.07 | 0.15 | 0.11 | -0.27 |
| Q6PA06 | ATL2 | 66.223 | -0.49 | -0.02 | -0.26 | 0.33 | -0.14 | 0.17 | 0.02 | 0.22 | -0.27 |
| Q8CGZ0 | CHERP | 106.17 | -0.22 | 0.03 | -0.10 | 0.18 | 0.24 | 0.11 | 0.18 | 0.09 | -0.27 |
| Q8CHR6 | DPYD | 111.25 | 0.28 | 0.31 | 0.29 | 0.03 | 0.68 | 0.46 | 0.57 | 0.16 | -0.27 |
| Q9CQ54 | NDUFC2 | 14.164 | 0.08 | -0.10 | -0.01 | 0.13 | 0.23 | 0.30 | 0.27 | 0.05 | -0.27 |
| Q91VD9 | NDUFS1 | 79.776 | 0.08 | 0.01 | 0.05 | 0.05 | 0.27 | 0.38 | 0.32 | 0.08 | -0.27 |
| O08705 | SLC10A1 | 39.413 | -0.74 | -0.86 | -0.80 | 0.08 | -0.63 | -0.42 | -0.52 | 0.15 | -0.28 |
| P61922 | ABAT | 56.451 | 0.56 | 0.31 | 0.43 | 0.18 | 0.74 | 0.68 | 0.71 | 0.04 | -0.28 |
| Q99LM2 | CDK5RAP3 | 56.99 | -0.40 | -0.16 | -0.28 | 0.17 | 0.12 | -0.12 | 0.00 | 0.17 | -0.28 |
| O08749 | DLD | 54.272 | -0.13 | -0.19 | -0.16 | 0.05 | 0.08 | 0.16 | 0.12 | 0.05 | -0.28 |

| Q5U5V2 | HYKK | 42.356 | -0.44 | -0.42 | -0.43 | 0.02 | -0.13 | -0.16 | -0.15 | 0.02 | -0.28 |
| --- | --- | --- | --- | --- | --- | --- | --- | --- | --- | --- | --- |
| Q8QZS1 | HIBCH | 43.037 | -0.21 | -0.38 | -0.30 | 0.12 | -0.03 | -0.01 | -0.02 | 0.01 | -0.28 |
| Q791V5 | MTCH2 | 33.499 | 0.01 | -0.05 | -0.02 | 0.04 | 0.29 | 0.24 | 0.26 | 0.03 | -0.28 |
| Q9JHR7 | IDE | 117.77 | -0.30 | -0.26 | -0.28 | 0.03 | 0.14 | -0.14 | 0.00 | 0.20 | -0.28 |
| P48771 | COX7A2 | 9.2908 | -0.21 | -0.23 | -0.22 | 0.02 | 0.14 | -0.02 | 0.06 | 0.12 | -0.28 |
| Q9D1I5 | MCEE | 19.017 | -0.25 | -0.19 | -0.22 | 0.04 | -0.01 | 0.13 | 0.06 | 0.10 | -0.28 |
| P03921 | MTND5 | 68.474 | -0.10 | -0.01 | -0.05 | 0.06 | 0.20 | 0.27 | 0.23 | 0.05 | -0.28 |
| P38060 | HMGCL | 34.238 | -0.11 | -0.33 | -0.22 | 0.16 | 0.20 | -0.07 | 0.07 | 0.19 | -0.28 |
| Q9CQA3 | SDHB | 31.814 | 0.00 | -0.11 | -0.05 | 0.08 | 0.24 | 0.22 | 0.23 | 0.02 | -0.28 |
| Q8VCZ9 | PRODH2 | 50.723 | -0.65 | -0.88 | -0.77 | 0.16 | -0.44 | -0.52 | -0.48 | 0.05 | -0.28 |
| Q9Z2I8 | SUCLG2 | 46.839 | -0.15 | -0.22 | -0.18 | 0.05 | 0.07 | 0.13 | 0.10 | 0.04 | -0.28 |
| Q8K440 | ABCA8B | 183.04 | -0.22 | -0.22 | -0.22 | 0.00 | 0.12 | 0.00 | 0.06 | 0.09 | -0.29 |
| Q9D7A8 | ARMC1 | 31.246 | -0.22 | -0.23 | -0.23 | 0.01 | 0.01 | 0.11 | 0.06 | 0.08 | -0.29 |

| Q8BG51 | RHOT1 | 72.241 | -0.02 | 0.00 | -0.01 | 0.01 | 0.25 | 0.30 | 0.28 | 0.03 | -0.29 |
| --- | --- | --- | --- | --- | --- | --- | --- | --- | --- | --- | --- |
| Q99J39 | MLYCD | 54.735 | -0.04 | -0.15 | -0.10 | 0.08 | 0.19 | 0.20 | 0.19 | 0.01 | -0.29 |
| Q9Z1P6 | NDUFA7 | 12.575 | -0.06 | 0.02 | -0.02 | 0.06 | 0.24 | 0.30 | 0.27 | 0.04 | -0.29 |
| Q9Z0M5 | LIPA | 45.325 | 0.18 | 0.55 | 0.37 | 0.26 | 0.82 | 0.50 | 0.66 | 0.22 | -0.29 |
| Q8BMS4 | COQ3 | 40.956 | -0.20 | -0.22 | -0.21 | 0.02 | 0.10 | 0.06 | 0.08 | 0.03 | -0.29 |
| Q9CXZ1 | NDUFS4 | 19.784 | 0.03 | -0.01 | 0.01 | 0.03 | 0.26 | 0.35 | 0.30 | 0.07 | -0.29 |
| P68368 | TUBA4A | 49.924 | -0.58 | -0.23 | -0.41 | 0.25 | -0.29 | 0.06 | -0.12 | 0.25 | -0.29 |
| Q9DCW4 | ETFB | 27.623 | -0.16 | -0.27 | -0.22 | 0.07 | 0.08 | 0.07 | 0.08 | 0.01 | -0.29 |
| Q9CXN7 | PBLD2 | 31.983 | -0.40 | -0.35 | -0.38 | 0.04 | -0.10 | -0.07 | -0.09 | 0.02 | -0.29 |
| Q91W64 | CYP2C70 | 56.019 | -0.28 | -0.33 | -0.31 | 0.04 | -0.10 | 0.07 | -0.01 | 0.12 | -0.30 |
| Q91YQ5 | RPN1 | 68.527 | -0.14 | 0.01 | -0.07 | 0.10 | 0.38 | 0.08 | 0.23 | 0.21 | -0.30 |
| P56480 | ATP5B | 56.3 | -0.01 | -0.25 | -0.13 | 0.17 | 0.19 | 0.15 | 0.17 | 0.03 | -0.30 |
| Q9D023 | MPC2 | 14.286 | -0.90 | -0.71 | -0.81 | 0.13 | -0.61 | -0.42 | -0.51 | 0.14 | -0.30 |

| Q9CQJ8 | NDUFB9 | 21.984 | -0.04 | -0.02 | -0.03 | 0.01 | 0.22 | 0.32 | 0.27 | 0.07 | -0.30 |
| --- | --- | --- | --- | --- | --- | --- | --- | --- | --- | --- | --- |
| O35423 | AGXT | 45.912 | 0.00 | -0.05 | -0.03 | 0.03 | 0.34 | 0.21 | 0.27 | 0.09 | -0.30 |
| Q922D8 | MTHFD1 | 101.2 | -0.71 | -0.61 | -0.66 | 0.07 | -0.31 | -0.41 | -0.36 | 0.07 | -0.30 |
| Q8BWQ1 | UGT2A3 | 61.119 | 0.11 | 0.17 | 0.14 | 0.04 | 0.43 | 0.46 | 0.44 | 0.02 | -0.30 |
| Q9D6S7 | MRRF | 29.05 | -0.36 | -0.27 | -0.31 | 0.06 | -0.13 | 0.10 | -0.01 | 0.16 | -0.30 |
| Q8C5H8 | NADK2 | 50.858 | -0.10 | -0.18 | -0.14 | 0.05 | 0.15 | 0.17 | 0.16 | 0.02 | -0.30 |
| Q5SX40 | MYH1 | 223.34 | -6.04 | -5.09 | -5.56 | 0.67 | -5.46 | -5.06 | -5.26 | 0.28 | -0.30 |
| Q9WUM5 | SUCLG1 | 36.154 | -0.13 | -0.30 | -0.21 | 0.12 | 0.15 | 0.04 | 0.09 | 0.08 | -0.30 |
| P07759 | SERPINA3K | 46.879 | 0.74 | 0.83 | 0.79 | 0.06 | 0.98 | 1.20 | 1.09 | 0.16 | -0.31 |
| Q64433 | HSPE1 | 10.963 | -0.29 | -0.27 | -0.28 | 0.02 | -0.01 | 0.06 | 0.03 | 0.05 | -0.31 |
| Q9CQN1 | TRAP1 | 80.208 | -0.18 | -0.23 | -0.20 | 0.04 | 0.17 | 0.04 | 0.10 | 0.10 | -0.31 |
| Q99MR8 | MCCC1 | 79.343 | -0.01 | -0.13 | -0.07 | 0.09 | 0.32 | 0.17 | 0.24 | 0.10 | -0.31 |
| Q91YP0 | L2HGDH | 50.898 | 0.08 | 0.07 | 0.07 | 0.00 | 0.28 | 0.49 | 0.38 | 0.14 | -0.31 |

| Q8BIJ6 | IARS2 | 112.8 | -0.02 | 0.11 | 0.05 | 0.09 | 0.41 | 0.31 | 0.36 | 0.07 | -0.31 |
| --- | --- | --- | --- | --- | --- | --- | --- | --- | --- | --- | --- |
| P31651 | SLC6A12 | 69.613 | 0.17 | 0.10 | 0.13 | 0.05 | 0.42 | 0.47 | 0.45 | 0.04 | -0.31 |
| O88531 | PPT1 | 34.49 | 1.11 | 1.09 | 1.10 | 0.02 | 1.35 | 1.48 | 1.41 | 0.09 | -0.31 |
| Q99JY0 | HADHB | 51.386 | -0.21 | -0.34 | -0.27 | 0.09 | 0.04 | 0.04 | 0.04 | 0.00 | -0.31 |
| Q8K3J1 | NDUFS8 | 24.038 | 0.01 | -0.04 | -0.02 | 0.03 | 0.28 | 0.31 | 0.30 | 0.02 | -0.31 |
| Q8BP40 | ACP6 | 47.624 | 0.31 | 0.31 | 0.31 | 0.00 | 0.60 | 0.65 | 0.62 | 0.04 | -0.32 |
| Q8C5W0 | CLMN | 117.23 | -0.34 | 0.19 | -0.07 | 0.37 | 0.18 | 0.31 | 0.24 | 0.09 | -0.32 |
| Q8K023 | AKR1C18 | 37.176 | -0.37 | -0.73 | -0.55 | 0.25 | -0.10 | -0.37 | -0.23 | 0.19 | -0.32 |
| Q99JR1 | SFXN1 | 35.649 | 0.43 | 0.30 | 0.36 | 0.10 | 0.76 | 0.61 | 0.68 | 0.11 | -0.32 |
| Q06185 | ATP5I | 8.2355 | -0.33 | -0.32 | -0.32 | 0.01 | 0.05 | -0.05 | 0.00 | 0.07 | -0.32 |
| Q99LC3 | NDUFA10 | 40.603 | 0.00 | -0.06 | -0.03 | 0.04 | 0.32 | 0.26 | 0.29 | 0.05 | -0.32 |
| Q925N0 | SFXN5 | 37.328 | -0.06 | -0.06 | -0.06 | 0.00 | 0.26 | 0.27 | 0.26 | 0.00 | -0.32 |
| Q07417 | ACADS | 44.889 | 0.02 | -0.15 | -0.06 | 0.12 | 0.31 | 0.21 | 0.26 | 0.07 | -0.32 |

| P63038 | HSPD1 | 60.955 | -0.09 | -0.20 | -0.14 | 0.08 | 0.15 | 0.21 | 0.18 | 0.04 | -0.33 |
| --- | --- | --- | --- | --- | --- | --- | --- | --- | --- | --- | --- |
| Q5SX39 | MYH4 | 222.86 | -5.78 | -5.03 | -5.40 | 0.52 | -5.19 | -4.96 | -5.08 | 0.16 | -0.33 |
| P56391 | COX6B1 | 10.071 | -0.10 | -0.14 | -0.12 | 0.03 | 0.21 | 0.21 | 0.21 | 0.00 | -0.33 |
| O09172 | GCLM | 30.534 | -0.41 | -0.34 | -0.38 | 0.05 | -0.08 | -0.02 | -0.05 | 0.04 | -0.33 |
| Q8BTY1 | CCBL1 | 47.563 | -0.48 | -0.59 | -0.53 | 0.08 | -0.27 | -0.14 | -0.21 | 0.09 | -0.33 |
| Q9D6U8 | FAM162A | 17.725 | -0.31 | 0.07 | -0.12 | 0.26 | 0.13 | 0.29 | 0.21 | 0.12 | -0.33 |
| P08775 | POLR2A | 217.17 | -0.08 | 0.66 | 0.29 | 0.52 | 0.92 | 0.33 | 0.62 | 0.41 | -0.33 |
| Q8K441 | ABCA6 | 183.28 | -0.01 | 0.28 | 0.13 | 0.21 | 0.39 | 0.54 | 0.47 | 0.11 | -0.33 |
| Q9Z2V4 | PCK1 | 69.354 | 1.86 | 1.97 | 1.92 | 0.08 | 2.45 | 2.06 | 2.25 | 0.28 | -0.34 |
| P06797 | CTSL | 37.547 | 1.44 | 1.09 | 1.27 | 0.25 | 1.63 | 1.58 | 1.60 | 0.04 | -0.34 |
| P03888 | MTND1 | 36.059 | -0.03 | -0.01 | -0.02 | 0.01 | 0.36 | 0.28 | 0.32 | 0.05 | -0.34 |
| Q3UNX5 | ACSM3 | 65.622 | -0.21 | -0.26 | -0.24 | 0.03 | 0.02 | 0.18 | 0.10 | 0.11 | -0.34 |
| P50544 | ACADVL | 70.875 | -0.27 | -0.31 | -0.29 | 0.03 | 0.08 | 0.01 | 0.04 | 0.04 | -0.34 |

| Q99L13 | HIBADH | 35.44 | 0.18 | 0.02 | 0.10 | 0.11 | 0.43 | 0.45 | 0.44 | 0.02 | -0.34 |
| --- | --- | --- | --- | --- | --- | --- | --- | --- | --- | --- | --- |
| Q9DCJ5 | NDUFA8 | 19.992 | -0.14 | 0.06 | -0.04 | 0.14 | 0.22 | 0.38 | 0.30 | 0.11 | -0.34 |
| P16332 | MUT | 82.843 | 0.21 | 0.20 | 0.21 | 0.01 | 0.45 | 0.65 | 0.55 | 0.14 | -0.34 |
| Q8R0F8 | FAHD1 | 25.172 | -0.12 | -0.09 | -0.10 | 0.02 | 0.20 | 0.28 | 0.24 | 0.06 | -0.34 |
| Q91WK5 | GCSH | 18.637 | -0.20 | -0.39 | -0.30 | 0.13 | 0.04 | 0.06 | 0.05 | 0.01 | -0.34 |
| Q71LX4 | TLN2 | 253.62 | 0.74 | 1.06 | 0.90 | 0.22 | 1.23 | 1.27 | 1.25 | 0.03 | -0.35 |
| Q05920 | PC | 129.68 | -0.61 | -0.45 | -0.53 | 0.11 | -0.27 | -0.10 | -0.18 | 0.12 | -0.35 |
| P41216 | ACSL1 | 77.951 | 0.23 | 0.13 | 0.18 | 0.07 | 0.62 | 0.44 | 0.53 | 0.13 | -0.35 |
| Q8R1S0 | COQ6 | 51.392 | -0.08 | -0.19 | -0.13 | 0.08 | 0.29 | 0.14 | 0.21 | 0.10 | -0.35 |
| Q8BMS1 | HADHA | 82.669 | -0.20 | -0.34 | -0.27 | 0.10 | 0.19 | -0.01 | 0.09 | 0.14 | -0.35 |
| Q9CZ13 | UQCRC1 | 52.851 | 0.00 | -0.20 | -0.10 | 0.14 | 0.33 | 0.17 | 0.25 | 0.11 | -0.35 |
| Q61425 | HADH | 34.463 | -0.41 | -0.54 | -0.48 | 0.09 | -0.08 | -0.16 | -0.12 | 0.06 | -0.35 |
| P09671 | SOD2 | 24.603 | -0.07 | -0.11 | -0.09 | 0.03 | 0.21 | 0.32 | 0.26 | 0.07 | -0.36 |

| Q9DB20 | ATP5O | 23.363 | -0.21 | -0.29 | -0.25 | 0.06 | 0.08 | 0.14 | 0.11 | 0.04 | -0.36 |
| --- | --- | --- | --- | --- | --- | --- | --- | --- | --- | --- | --- |
| Q5U458 | DNAJC11 | 63.232 | -0.32 | -0.23 | -0.28 | 0.07 | 0.13 | 0.03 | 0.08 | 0.07 | -0.36 |
| Q8R0Y8 | SLC25A42 | 35.24 | 0.01 | 0.01 | 0.01 | 0.00 | 0.36 | 0.37 | 0.37 | 0.01 | -0.36 |
| Q9DCX2 | ATP5H | 18.749 | -0.19 | -0.24 | -0.22 | 0.03 | 0.11 | 0.17 | 0.14 | 0.04 | -0.36 |
| Q8VC12 | UROC1 | 74.589 | -0.08 | -0.22 | -0.15 | 0.10 | 0.37 | 0.05 | 0.21 | 0.22 | -0.36 |
| Q9JKY5 | HIP1R | 119.43 | 0.14 | 0.42 | 0.28 | 0.20 | 0.56 | 0.73 | 0.65 | 0.12 | -0.36 |
| Q921G7 | ETFDH | 68.09 | -0.15 | -0.15 | -0.15 | 0.00 | 0.29 | 0.13 | 0.21 | 0.11 | -0.36 |
| Q9Z1J3 | NFS1 | 50.569 | -0.24 | 0.02 | -0.11 | 0.18 | 0.26 | 0.26 | 0.26 | 0.00 | -0.36 |
| Q8CGK3 | LONP1 | 105.84 | -0.35 | -0.11 | -0.23 | 0.17 | 0.15 | 0.12 | 0.14 | 0.02 | -0.36 |
| Q8BH59 | SLC25A12 | 74.569 | -0.16 | -0.01 | -0.09 | 0.11 | 0.36 | 0.20 | 0.28 | 0.12 | -0.37 |
| Q9CQC7 | NDUFB4 | 15.081 | -0.25 | 0.08 | -0.08 | 0.23 | 0.27 | 0.29 | 0.28 | 0.02 | -0.37 |
| Q811D0 | DLG1 | 100.12 | -0.52 | 0.20 | -0.16 | 0.51 | -0.07 | 0.48 | 0.21 | 0.39 | -0.37 |
| Q8C196 | CPS1 | 164.62 | 0.15 | 0.31 | 0.23 | 0.11 | 0.41 | 0.78 | 0.60 | 0.26 | -0.37 |

| Q3UEG6 | AGXT2 | 57.114 | 0.39 | 0.38 | 0.38 | 0.01 | 0.69 | 0.82 | 0.75 | 0.09 | -0.37 |
| --- | --- | --- | --- | --- | --- | --- | --- | --- | --- | --- | --- |
| Q60634 | FLOT2 | 47.037 | 0.38 | 0.19 | 0.28 | 0.13 | 0.63 | 0.68 | 0.65 | 0.03 | -0.37 |
| P43883 | PLIN2 | 46.646 | -1.79 | 1.36 | -0.22 | 2.23 | 0.18 | 0.13 | 0.15 | 0.04 | -0.37 |
| Q63880 | CES3A | 63.317 | -0.02 | 0.15 | 0.07 | 0.11 | 0.53 | 0.34 | 0.44 | 0.13 | -0.37 |
| Q9WU79 | PRODH | 68.035 | -0.08 | -0.02 | -0.05 | 0.04 | 0.23 | 0.41 | 0.32 | 0.13 | -0.37 |
| P13542 | MYH8 | 222.7 | -6.43 | -5.03 | -5.73 | 0.99 | -5.56 | -5.15 | -5.35 | 0.29 | -0.38 |
| Q8BM55 | TMEM214 | 76.429 | -0.50 | -0.29 | -0.40 | 0.15 | -0.02 | -0.01 | -0.02 | 0.01 | -0.38 |
| Q8K010 | OPLAH | 137.61 | -0.25 | 0.29 | 0.02 | 0.38 | 0.31 | 0.48 | 0.40 | 0.12 | -0.38 |
| Q3UZZ6 | SULT1D1 | 35.083 | -0.60 | -0.64 | -0.62 | 0.02 | -0.23 | -0.25 | -0.24 | 0.02 | -0.38 |
| P10922 | H1F0 | 20.861 | 0.47 | 0.48 | 0.48 | 0.00 | 0.95 | 0.77 | 0.86 | 0.12 | -0.38 |
| P14246 | SLC2A2 | 57.106 | -0.57 | -0.70 | -0.64 | 0.10 | -0.28 | -0.21 | -0.25 | 0.05 | -0.39 |
| O55125 | NIPSNAP1 | 33.363 | 0.15 | -0.05 | 0.05 | 0.14 | 0.44 | 0.44 | 0.44 | 0.00 | -0.39 |
| Q61009 | SCARB1 | 56.753 | -0.28 | -0.32 | -0.30 | 0.03 | -0.06 | 0.24 | 0.09 | 0.21 | -0.39 |

| Q9QXF8 | GNMT | 32.675 | 0.73 | 0.55 | 0.64 | 0.13 | 1.14 | 0.93 | 1.03 | 0.15 | -0.39 |
| --- | --- | --- | --- | --- | --- | --- | --- | --- | --- | --- | --- |
| Q8BWT1 | ACAA2 | 41.829 | -0.14 | -0.26 | -0.20 | 0.08 | 0.21 | 0.18 | 0.19 | 0.02 | -0.40 |
| P62897 | CYCS | 11.605 | -0.73 | -0.11 | -0.42 | 0.44 | -0.05 | 0.00 | -0.02 | 0.04 | -0.40 |
| Q9WU78 | PDCD6IP | 96.023 | -0.11 | 0.22 | 0.06 | 0.23 | 0.96 | -0.05 | 0.45 | 0.71 | -0.40 |
| P17426 | AP2A1 | 107.66 | 0.15 | 0.46 | 0.31 | 0.21 | 1.11 | 0.30 | 0.70 | 0.58 | -0.40 |
| Q9CZS1 | ALDH1B1 | 57.552 | 0.30 | 0.33 | 0.31 | 0.02 | 0.65 | 0.77 | 0.71 | 0.09 | -0.40 |
| Q9R092 | HSD17B6 | 36.102 | -0.55 | -0.29 | -0.42 | 0.19 | 0.00 | -0.03 | -0.02 | 0.02 | -0.40 |
| Q91VR2 | ATP5C1 | 32.886 | -0.15 | -0.33 | -0.24 | 0.13 | 0.18 | 0.14 | 0.16 | 0.03 | -0.40 |
| Q9D855 | UQCRB | 13.527 | -0.26 | -0.29 | -0.27 | 0.02 | 0.16 | 0.09 | 0.13 | 0.05 | -0.40 |
| Q9CXF0 | KYNU | 52.325 | 0.08 | -0.14 | -0.03 | 0.16 | 0.30 | 0.44 | 0.37 | 0.09 | -0.40 |
| Q5EBG8 |  | 21.862 | -0.78 | -0.84 | -0.81 | 0.04 | -0.63 | -0.18 | -0.41 | 0.32 | -0.40 |
| Q9DCU9 | HOGA1 | 34.644 | 0.34 | 0.23 | 0.28 | 0.08 | 1.12 | 0.26 | 0.69 | 0.61 | -0.41 |
| P97807 | FH | 54.356 | 0.14 | 0.04 | 0.09 | 0.07 | 0.48 | 0.51 | 0.49 | 0.02 | -0.41 |

| Q9CQQ7 | ATP5F1 | 28.948 | -0.26 | -0.30 | -0.28 | 0.03 | 0.06 | 0.20 | 0.13 | 0.10 | -0.41 |
| --- | --- | --- | --- | --- | --- | --- | --- | --- | --- | --- | --- |
| Q9D172 | D10JHU81E | 28.09 | -0.06 | -0.09 | -0.08 | 0.02 | 0.31 | 0.34 | 0.33 | 0.02 | -0.41 |
| P14094 | ATP1B1 | 35.194 | 0.05 | -0.06 | -0.01 | 0.08 | 0.48 | 0.31 | 0.40 | 0.12 | -0.41 |
| G3X982 | AOX3 | 146.9 | 1.14 | 1.44 | 1.29 | 0.21 | 1.47 | 1.92 | 1.69 | 0.32 | -0.41 |
| Q9DC69 | NDUFA9 | 42.525 | 0.03 | -0.12 | -0.04 | 0.10 | 0.45 | 0.27 | 0.36 | 0.13 | -0.41 |
| Q8BXA1 | GOLIM4 | 76.784 | -0.43 | -0.02 | -0.23 | 0.30 | 0.03 | 0.34 | 0.19 | 0.21 | -0.41 |
| P22315 | FECH | 47.13 | 0.43 | 0.46 | 0.45 | 0.02 | 0.81 | 0.91 | 0.86 | 0.07 | -0.41 |
| O35488 | SLC27A2 | 70.422 | -0.54 | -0.54 | -0.54 | 0.00 | 0.02 | -0.28 | -0.13 | 0.21 | -0.41 |
| Q91W43 | GLDC | 113.27 | -0.73 | -0.63 | -0.68 | 0.08 | -0.28 | -0.26 | -0.27 | 0.01 | -0.41 |
| Q61578 | FDXR | 54.201 | 0.28 | 0.12 | 0.20 | 0.11 | 0.67 | 0.55 | 0.61 | 0.08 | -0.41 |
| Q8BX70 | VPS13C | 420.08 | -0.18 | 0.27 | 0.04 | 0.32 | 0.36 | 0.55 | 0.46 | 0.13 | -0.41 |
| P54869 | HMGCS2 | 56.822 | -0.45 | -0.66 | -0.55 | 0.15 | -0.01 | -0.26 | -0.14 | 0.18 | -0.41 |
| Q99MN9 | PCCB | 58.408 | -0.20 | -0.21 | -0.20 | 0.00 | 0.18 | 0.24 | 0.21 | 0.04 | -0.41 |

| P46735 | MYO1B | 128.56 | 0.21 | 0.58 | 0.40 | 0.27 | 0.79 | 0.83 | 0.81 | 0.03 | -0.42 |
| --- | --- | --- | --- | --- | --- | --- | --- | --- | --- | --- | --- |
| A2AS89 | AGMAT | 38.255 | 0.02 | -0.10 | -0.04 | 0.09 | 0.40 | 0.35 | 0.38 | 0.04 | -0.42 |
| Q9EQ20 | ALDH6A1 | 57.915 | -0.08 | -0.02 | -0.05 | 0.04 | 0.21 | 0.52 | 0.36 | 0.22 | -0.42 |
| Q9D6J5 | NDUFB8 | 21.876 | -0.05 | -0.04 | -0.05 | 0.01 | 0.37 | 0.38 | 0.37 | 0.01 | -0.42 |
| Q99JW2 | ACY1 | 45.78 | -0.44 | -0.46 | -0.45 | 0.02 | -0.03 | -0.03 | -0.03 | 0.00 | -0.42 |
| P52196 | TST | 33.466 | -0.03 | -0.25 | -0.14 | 0.15 | 0.57 | -0.01 | 0.28 | 0.41 | -0.42 |
| Q9CQ75 | NDUFA2 | 10.916 | -0.09 | 0.04 | -0.03 | 0.09 | 0.40 | 0.40 | 0.40 | 0.00 | -0.43 |
| P50172 | HSD11B1 | 32.364 | -0.35 | -0.53 | -0.44 | 0.13 | 0.10 | -0.12 | -0.01 | 0.15 | -0.43 |
| Q9QY30 | ABCB11 | 146.75 | -0.24 | -0.15 | -0.19 | 0.07 | 0.19 | 0.28 | 0.24 | 0.06 | -0.43 |
| O09158 | CYP3A25 | 58.121 | 0.64 | 0.57 | 0.61 | 0.05 | 1.01 | 1.06 | 1.04 | 0.04 | -0.43 |
| Q922Q1 | 42065 | 38.194 | 0.10 | -0.07 | 0.01 | 0.13 | 0.51 | 0.39 | 0.45 | 0.09 | -0.44 |
| Q9CR68 | UQCRFS1 | 29.367 | 0.05 | 0.07 | 0.06 | 0.02 | 0.47 | 0.51 | 0.49 | 0.03 | -0.44 |
| O88962 | CYP8B1 | 57.706 | 0.21 | 0.01 | 0.11 | 0.14 | 0.58 | 0.52 | 0.55 | 0.04 | -0.44 |

| Q9DCM0 | ETHE1 | 27.738 | -0.07 | 0.32 | 0.13 | 0.27 | 0.48 | 0.65 | 0.57 | 0.13 | -0.44 |
| --- | --- | --- | --- | --- | --- | --- | --- | --- | --- | --- | --- |
| P56379 | MP68 | 6.6979 | -0.30 | -0.35 | -0.33 | 0.04 | 0.09 | 0.13 | 0.11 | 0.03 | -0.44 |
| P56395 | CYB5A | 15.241 | -0.40 | 0.13 | -0.14 | 0.38 | 0.28 | 0.33 | 0.31 | 0.03 | -0.44 |
| E9Q557 | DSP | 332.91 | -0.33 | 0.03 | -0.15 | 0.26 | 0.25 | 0.34 | 0.29 | 0.06 | -0.44 |
| P24721 | ASGR2 | 34.906 | -0.47 | -0.81 | -0.64 | 0.24 | -0.17 | -0.22 | -0.19 | 0.03 | -0.45 |
| P12787 | COX5A | 16.101 | -0.41 | -0.15 | -0.28 | 0.18 | 0.10 | 0.24 | 0.17 | 0.10 | -0.45 |
| Q5YD48 | A1CF | 65.681 | -0.54 | -0.25 | -0.40 | 0.20 | -0.03 | 0.14 | 0.05 | 0.12 | -0.45 |
| Q8VBW8 | TTC36 | 20.132 | -0.72 | -0.52 | -0.62 | 0.14 | -0.40 | 0.07 | -0.17 | 0.33 | -0.45 |
| Q9DCS3 | MECR | 40.342 | 0.10 | -0.04 | 0.03 | 0.10 | 0.47 | 0.51 | 0.49 | 0.03 | -0.46 |
| Q8R164 | BPHL | 32.851 | -0.30 | -0.24 | -0.27 | 0.04 | 0.12 | 0.26 | 0.19 | 0.10 | -0.46 |
| Q8BX90 | FNDC3A | 131.96 | -0.49 | -0.18 | -0.33 | 0.22 | 0.31 | -0.05 | 0.13 | 0.26 | -0.46 |
| O54749 | CYP2J5 | 57.783 | 0.06 | 0.10 | 0.08 | 0.03 | 0.52 | 0.57 | 0.54 | 0.03 | -0.46 |
| Q8BHE8 |  | 32.985 | -0.45 | -0.19 | -0.32 | 0.18 | 0.12 | 0.17 | 0.14 | 0.03 | -0.46 |

| Q9WVD5 | SLC25A15 | 32.823 | 0.07 | 0.02 | 0.05 | 0.03 | 0.57 | 0.46 | 0.51 | 0.08 | -0.47 |
| --- | --- | --- | --- | --- | --- | --- | --- | --- | --- | --- | --- |
| P01868 | IGHG1 | 35.704 | 3.40 | 3.17 | 3.28 | 0.17 | 3.74 | 3.76 | 3.75 | 0.02 | -0.47 |
| Q91YN5 | UAP1 | 58.608 | -0.82 | -0.44 | -0.63 | 0.27 | -0.18 | -0.14 | -0.16 | 0.03 | -0.47 |
| Q9QXD6 | FBP1 | 36.912 | -0.61 | -0.87 | -0.74 | 0.18 | -0.19 | -0.33 | -0.26 | 0.10 | -0.47 |
| Q91ZA3 | PCCA | 79.921 | -0.08 | -0.22 | -0.15 | 0.10 | 0.44 | 0.21 | 0.32 | 0.16 | -0.48 |
| Q9Z0X1 | AIFM1 | 66.765 | -0.43 | -0.31 | -0.37 | 0.09 | 0.33 | -0.10 | 0.11 | 0.31 | -0.48 |
| P28650 | ADSSL1 | 50.254 | 0.64 | 0.52 | 0.58 | 0.09 | 1.13 | 0.99 | 1.06 | 0.10 | -0.48 |
| P45952 | ACADM | 46.481 | 0.14 | 0.18 | 0.16 | 0.03 | 0.58 | 0.70 | 0.64 | 0.09 | -0.48 |
| P42125 | ECI1 | 32.25 | -0.31 | -0.48 | -0.40 | 0.12 | 0.07 | 0.10 | 0.09 | 0.02 | -0.48 |
| P10518 | ALAD | 36.023 | -0.55 | -0.50 | -0.52 | 0.04 | 0.05 | -0.13 | -0.04 | 0.12 | -0.48 |
| Q8R3Q6 | CCDC58 | 16.665 | -0.26 | -0.12 | -0.19 | 0.10 | 0.31 | 0.27 | 0.29 | 0.03 | -0.48 |
| Q78IK2 | USMG5 | 6.3814 | -0.53 | -0.32 | -0.43 | 0.15 | 0.01 | 0.11 | 0.06 | 0.07 | -0.49 |
| O35988 | SDC4 | 21.482 | -0.04 | 0.49 | 0.23 | 0.38 | 0.45 | 0.98 | 0.71 | 0.38 | -0.49 |

| Q61102 | ABCB7 | 82.58 | -0.25 | -0.15 | -0.20 | 0.07 | 0.35 | 0.24 | 0.30 | 0.08 | -0.49 |
| --- | --- | --- | --- | --- | --- | --- | --- | --- | --- | --- | --- |
| Q07076 | ANXA7 | 49.925 | -0.13 | 0.51 | 0.19 | 0.45 | 1.37 | 0.02 | 0.69 | 0.95 | -0.50 |
| Q9DBL1 | ACADSB | 47.874 | 0.47 | 0.28 | 0.37 | 0.14 | 0.88 | 0.89 | 0.88 | 0.01 | -0.51 |
| P60334 | CDO1 | 23.026 | -1.08 | -0.90 | -0.99 | 0.12 | -0.46 | -0.49 | -0.48 | 0.02 | -0.52 |
| Q99LB7 | SARDH | 101.68 | -0.19 | -0.23 | -0.21 | 0.03 | 0.39 | 0.23 | 0.31 | 0.11 | -0.52 |
| Q5RKZ7 | MOCS1 | 69.858 | -0.08 | 0.06 | -0.01 | 0.10 | 0.37 | 0.64 | 0.51 | 0.19 | -0.52 |
| P36552 | CPOX | 49.714 | -0.28 | -0.28 | -0.28 | 0.01 | 0.20 | 0.28 | 0.24 | 0.05 | -0.52 |
| Q61595 | KTN1 | 152.59 | -0.23 | 0.16 | -0.03 | 0.28 | 1.11 | -0.13 | 0.49 | 0.88 | -0.52 |
| Q8R086 | SUOX | 60.755 | -0.07 | -0.23 | -0.15 | 0.11 | 0.39 | 0.36 | 0.38 | 0.02 | -0.52 |
| Q8C165 | PM20D1 | 55.662 | -0.28 | -0.22 | -0.25 | 0.04 | 0.20 | 0.35 | 0.28 | 0.11 | -0.53 |
| O70362 | GPLD1 | 93.254 | 0.20 | 0.67 | 0.44 | 0.33 | 0.86 | 1.08 | 0.97 | 0.16 | -0.53 |
| Q8VEK0 | TMEM30A | 41.06 | 0.15 | 0.09 | 0.12 | 0.04 | 0.67 | 0.64 | 0.66 | 0.02 | -0.54 |
| Q9DBW0 | CYP4V2 | 60.938 | 0.75 | 0.50 | 0.63 | 0.18 | 1.21 | 1.13 | 1.17 | 0.06 | -0.54 |

| O70570 | PIGR | 84.998 | -0.27 | 0.66 | 0.19 | 0.66 | 0.49 | 0.98 | 0.74 | 0.34 | -0.54 |
| --- | --- | --- | --- | --- | --- | --- | --- | --- | --- | --- | --- |
| Q9JIL4 | PDZK1 | 56.498 | -0.36 | -0.33 | -0.35 | 0.02 | 0.32 | 0.08 | 0.20 | 0.17 | -0.55 |
| Q8K2B3 | SDHA | 72.585 | -0.19 | -0.03 | -0.11 | 0.11 | 0.66 | 0.21 | 0.44 | 0.32 | -0.55 |
| Q5FW57 | GM4952 | 34.168 | -0.68 | -0.85 | -0.76 | 0.12 | -0.18 | -0.24 | -0.21 | 0.05 | -0.55 |
| Q9WVM8 | AADAT | 47.597 | 0.10 | 0.08 | 0.09 | 0.01 | 0.60 | 0.69 | 0.65 | 0.07 | -0.55 |
| O88986 | GCAT | 44.93 | -0.21 | -0.31 | -0.26 | 0.07 | 0.26 | 0.33 | 0.30 | 0.05 | -0.56 |
| P52825 | CPT2 | 73.98 | -0.03 | 0.03 | 0.00 | 0.04 | 0.81 | 0.31 | 0.56 | 0.35 | -0.56 |
| O08917 | FLOT1 | 47.513 | 0.12 | 0.20 | 0.16 | 0.06 | 0.79 | 0.66 | 0.72 | 0.10 | -0.56 |
| Q99MZ7 | PECR | 32.41 | -0.09 | -0.32 | -0.21 | 0.17 | 0.53 | 0.19 | 0.36 | 0.24 | -0.57 |
| Q9QXD1 | ACOX2 | 76.862 | 0.83 | 0.79 | 0.81 | 0.03 | 1.41 | 1.34 | 1.38 | 0.05 | -0.57 |
| Q8BGA8 | ACSM5 | 64.328 | 0.44 | 0.45 | 0.45 | 0.01 | 1.01 | 1.03 | 1.02 | 0.02 | -0.57 |
| Q9JJL3 | SLCO1B2 | 76.728 | 0.11 | -0.26 | -0.08 | 0.26 | 0.75 | 0.24 | 0.50 | 0.36 | -0.57 |
| Q9ER35 | FN3K | 35.032 | -0.38 | -0.46 | -0.42 | 0.06 | 0.05 | 0.27 | 0.16 | 0.15 | -0.58 |

| P52760 | HRSP12 | 14.255 | -0.35 | -0.15 | -0.25 | 0.14 | 0.28 | 0.38 | 0.33 | 0.07 | -0.58 |
| --- | --- | --- | --- | --- | --- | --- | --- | --- | --- | --- | --- |
| P40237 | CD82 | 29.628 | 0.11 | 0.21 | 0.16 | 0.07 | 0.78 | 0.69 | 0.74 | 0.07 | -0.58 |
| Q99P30 | NUDT7 | 26.856 | -0.02 | -0.05 | -0.04 | 0.02 | 0.56 | 0.54 | 0.55 | 0.01 | -0.59 |
| Q60936 | ADCK3 | 71.742 | -1.17 | -1.19 | -1.18 | 0.02 | -0.69 | -0.49 | -0.59 | 0.14 | -0.59 |
| Q91WG8 | GNE | 79.198 | 0.11 | 0.03 | 0.07 | 0.06 | 0.70 | 0.62 | 0.66 | 0.06 | -0.59 |
| Q8BWU8 | ETNPPL | 55.496 | -0.39 | -0.43 | -0.41 | 0.03 | 0.03 | 0.32 | 0.18 | 0.20 | -0.59 |
| Q3UUI3 | THEM4 | 26.031 | -0.44 | -0.41 | -0.43 | 0.02 | 0.03 | 0.30 | 0.17 | 0.19 | -0.59 |
| Q9QXX4 | SLC25A13 | 74.466 | -0.06 | 0.06 | 0.00 | 0.09 | 0.76 | 0.44 | 0.60 | 0.22 | -0.60 |
| P11725 | OTC | 39.764 | -0.06 | -0.19 | -0.12 | 0.09 | 0.50 | 0.46 | 0.48 | 0.03 | -0.60 |
| P28843 | DPP4 | 87.436 | 0.02 | 0.07 | 0.05 | 0.03 | 0.70 | 0.59 | 0.65 | 0.08 | -0.60 |
| Q99LI8 | HGS | 86.014 | -0.78 | 0.14 | -0.32 | 0.65 | 0.64 | -0.05 | 0.30 | 0.49 | -0.61 |
| O70589 | CASK | 105.11 | 0.67 | 0.81 | 0.74 | 0.10 | 1.52 | 1.19 | 1.35 | 0.24 | -0.62 |
| P55937 | GOLGA3 | 167.22 | -0.13 | 0.30 | 0.08 | 0.30 | 1.19 | 0.23 | 0.71 | 0.68 | -0.63 |

| Q9CPQ8 | ATP5L | 11.424 | -0.80 | -0.27 | -0.54 | 0.37 | 0.01 | 0.19 | 0.10 | 0.12 | -0.64 |
| --- | --- | --- | --- | --- | --- | --- | --- | --- | --- | --- | --- |
| Q8VE09 | TTC39C | 65.444 | 0.72 | 0.69 | 0.70 | 0.03 | 1.36 | 1.33 | 1.34 | 0.02 | -0.64 |
| Q9QY81 | NUP210 | 204.1 | -0.58 | -0.30 | -0.44 | 0.20 | 0.20 | 0.20 | 0.20 | 0.00 | -0.64 |
| Q8C0Z1 | ITFG3 | 60.575 | -0.31 | -0.08 | -0.20 | 0.16 | 0.48 | 0.41 | 0.45 | 0.05 | -0.64 |
| P70296 | PEBP1 | 20.83 | 0.18 | 0.12 | 0.15 | 0.04 | 0.79 | 0.81 | 0.80 | 0.02 | -0.65 |
| Q9WUR9 | AK4 | 25.061 | -0.69 | -0.67 | -0.68 | 0.01 | 0.44 | -0.50 | -0.03 | 0.66 | -0.65 |
| Q922B1 | MACROD1 | 35.294 | -0.29 | -0.44 | -0.37 | 0.10 | 0.25 | 0.33 | 0.29 | 0.05 | -0.66 |
| Q00898 | SERPINA1E | 45.891 | 3.76 | 0.80 | 2.28 | 2.09 | 5.03 | 0.96 | 3.00 | 2.88 | -0.72 |
| Q91XE0 | GLYAT | 34.098 | 0.36 | 0.19 | 0.28 | 0.12 | 1.08 | 0.92 | 1.00 | 0.11 | -0.72 |
| P23589 | CA5A | 34.072 | -0.49 | -0.65 | -0.57 | 0.11 | 0.14 | 0.19 | 0.17 | 0.04 | -0.74 |
| P50136 | BCKDHA | 50.37 | -0.09 | -0.07 | -0.08 | 0.01 | 0.55 | 0.83 | 0.69 | 0.20 | -0.77 |
| P28665 | MUG1 | 165.3 | 0.35 | 0.56 | 0.46 | 0.15 | 1.14 | 1.33 | 1.24 | 0.14 | -0.78 |
| Q9EQF5 | DPYS | 56.724 | -0.68 | -0.58 | -0.63 | 0.07 | 0.05 | 0.27 | 0.16 | 0.15 | -0.80 |

| Q7TNG8 | LDHD | 51.847 | 1.36 | 1.41 | 1.38 | 0.04 | 2.17 | 2.20 | 2.19 | 0.02 | -0.80 |
| --- | --- | --- | --- | --- | --- | --- | --- | --- | --- | --- | --- |
| Q91VA0 | ACSM1 | 64.76 | 0.15 | 0.09 | 0.12 | 0.04 | 1.02 | 0.85 | 0.93 | 0.12 | -0.81 |
| P52843 | SULT2A1 | 33.213 | -4.40 | -4.52 | -4.46 | 0.08 | -4.85 | -2.42 | -3.63 | 1.72 | -0.83 |
| P01867 | IGH-3 | 44.259 | 4.15 | 4.08 | 4.11 | 0.05 | 4.96 | 4.94 | 4.95 | 0.02 | -0.84 |
| Q9QZW0 | ATP11C | 129.24 | -0.48 | 0.00 | -0.24 | 0.34 | 0.50 | 0.71 | 0.60 | 0.15 | -0.85 |
| P58735 | SLC26A1 | 75.787 | -0.64 | -0.66 | -0.65 | 0.01 | 0.28 | 0.14 | 0.21 | 0.09 | -0.86 |
| Q6P3A8 | BCKDHB | 42.88 | -0.07 | -0.05 | -0.06 | 0.01 | 0.93 | 0.79 | 0.86 | 0.10 | -0.92 |
| P06684 | C5 | 188.88 | 0.18 | 0.63 | 0.40 | 0.32 | 1.29 | 1.43 | 1.36 | 0.10 | -0.96 |
| Q9WU19 | HAO1 | 41.001 | -0.76 | -0.67 | -0.71 | 0.06 | 0.23 | 0.28 | 0.26 | 0.03 | -0.97 |
| Q9R257 | HEBP1 | 21.067 | 0.39 | 0.42 | 0.41 | 0.02 | 1.21 | 1.56 | 1.38 | 0.25 | -0.98 |
| Q61419 | CMAH | 66.935 | -0.73 | -0.83 | -0.78 | 0.07 | 0.31 | 0.12 | 0.22 | 0.13 | -1.00 |
| P11589 | MUP2 | 20.663 | 1.74 | 3.21 | 2.47 | 1.04 | 2.89 | 4.09 | 3.49 | 0.85 | -1.02 |
| Q99J99 | MPST | 33.023 | 0.23 | 0.04 | 0.13 | 0.13 | 1.19 | 1.11 | 1.15 | 0.06 | -1.02 |

| P19157 | GSTP1 | 23.609 | 2.15 | 2.07 | 2.11 | 0.06 | 3.01 | 3.27 | 3.14 | 0.19 | -1.03 |
| --- | --- | --- | --- | --- | --- | --- | --- | --- | --- | --- | --- |
| Q8BVW0 | GANC | 102.01 | 0.46 | 0.75 | 0.60 | 0.21 | 1.78 | 1.50 | 1.64 | 0.20 | -1.03 |
| Q9DCY0 | KEG1 | 33.723 | 1.00 | 0.94 | 0.97 | 0.04 | 2.11 | 1.91 | 2.01 | 0.14 | -1.04 |
| Q91Z83 | MYH7 | 222.88 | -6.28 | -5.07 | -5.67 | 0.85 | -4.71 | -4.53 | -4.62 | 0.13 | -1.06 |
| Q9CPU0 | GLO1 | 20.809 | 0.79 | 0.85 | 0.82 | 0.04 | 1.90 | 1.98 | 1.94 | 0.05 | -1.12 |
| Q8VCU1 | CES3B | 63.352 | 0.37 | 0.24 | 0.31 | 0.09 | 1.50 | 1.38 | 1.44 | 0.09 | -1.13 |
| A2ATU0 | DHTKD1 | 102.79 | 1.02 | 0.88 | 0.95 | 0.10 | 2.18 | 2.02 | 2.10 | 0.11 | -1.15 |
| O55060 | TPMT | 27.585 | -0.05 | 0.03 | -0.01 | 0.06 | 1.24 | 1.30 | 1.27 | 0.04 | -1.28 |
| O35744 | CHIL3 | 44.458 | 4.06 | 4.37 | 4.21 | 0.22 | 5.33 | 5.89 | 5.61 | 0.39 | -1.40 |
| Q8K4H1 | AFMID | 34.228 | -1.00 | 0.09 | -0.46 | 0.78 | 1.03 | 1.02 | 1.02 | 0.01 | -1.48 |
| A2ASS6 | TTN | 3906.4 | 0.06 | -3.63 | -1.79 | 2.61 | 1.10 | -1.68 | -0.29 | 1.96 | -1.50 |
| Q3U4I7 | PYROXD2 | 62.939 | 1.38 | 1.36 | 1.37 | 0.01 | 2.71 | 3.07 | 2.89 | 0.25 | -1.52 |
| Q60991 | CYP7B1 | 58.47 | 3.84 | 3.44 | 3.64 | 0.29 | 5.37 | 5.32 | 5.34 | 0.04 | -1.70 |
