# Supplementary material for: Hepatitis B virus X induces inflammation and cancer in mice liver through dysregulation of cytoskeletal remodeling and lipid metabolism
Source: Oncotarget. 2016 Sep 30;7(43):70559–74. doi: 10.18632/oncotarget.12372 (PMC5342574; doi:10.18632/oncotarget.12372)
Supplement: Supplementary file 5 [file oncotarget-07-70559-s005.docx]

**Table 5S. The list for changed proteins identified from the comparison of 24M *p21* HBx/+ samples and WT littermates.**

| **Protein IDs** | **Gene**  **names** | **Mol.**  **weight**  **[kDa]** | （**24 M**  **Log2**  **HBX /SILAM**）  **GEL** | （**24 M**  **Log2**  **HBX /SILAM**）  **2D-LC** | **Mean**  **value_24M**  **HBx vs**  **SILAM** | **SD** | （**12 M**  **Log2**  **WT/SILAM**）  **GEL** | （**12 M**  **Log2**  **WT /SILAM**）  **2D-LC** | **Mean**  **value_24M**  **WT vs**  **SILAM** | **SD** | （**24M**  **Log2**  **HBx/24M WT**） |
| --- | --- | --- | --- | --- | --- | --- | --- | --- | --- | --- | --- |
| Q62523 | ZYX | 60.545 | 1.36 | 1.45 | 1.40 | 0.06 | -0.20 | 0.19 | -0.01 | 0.28 | 1.41 |
| P68510 | YWHAH | 28.211 | 0.77 | 0.70 | 0.74 | 0.05 | -0.10 | -0.15 | -0.13 | 0.03 | 0.86 |
| Q00519 | XDH | 146.56 | 0.13 | 0.43 | 0.28 | 0.21 | -0.70 | -0.18 | -0.44 | 0.36 | 0.72 |
| O88342 | WDR1 | 66.406 | 0.95 | 0.94 | 0.94 | 0.01 | 0.11 | 0.00 | 0.05 | 0.07 | 0.89 |
| P20152 | VIM | 53.687 | 2.12 | 1.99 | 2.06 | 0.09 | -0.21 | -0.02 | -0.11 | 0.13 | 2.17 |
| Q62465 | VAT1 | 43.096 | 1.77 | 1.64 | 1.70 | 0.09 | 0.56 | 0.73 | 0.65 | 0.12 | 1.06 |
| P63280 | UBE2I | 18.007 | 0.37 | 0.47 | 0.42 | 0.07 | -0.45 | -0.22 | -0.33 | 0.16 | 0.75 |
| Q91YR1 | TWF1 | 40.079 | 0.88 | 0.69 | 0.78 | 0.13 | 0.01 | 0.17 | 0.09 | 0.11 | 0.70 |
| P99024 | TUBB5 | 49.67 | 0.51 | 0.78 | 0.65 | 0.19 | -0.62 | -0.05 | -0.34 | 0.41 | 0.98 |
| A2ASS6 | TTN | 3906.4 | 0.06 | -3.63 | -1.79 | 2.61 | 1.10 | -1.68 | -0.29 | 1.96 | -1.50 |
| A3KMP2 | TTC38 | 52.223 | 0.15 | -0.08 | 0.03 | 0.16 | -0.81 | -0.95 | -0.88 | 0.10 | 0.92 |

| O55060 | TPMT | 27.585 | -0.05 | 0.03 | -0.01 | 0.06 | 1.24 | 1.30 | 1.27 | 0.04 | -1.28 |
| --- | --- | --- | --- | --- | --- | --- | --- | --- | --- | --- | --- |
| Q6IRU2 | TPM4 | 28.467 | 1.12 | 1.03 | 1.08 | 0.06 | 0.05 | -0.03 | 0.01 | 0.06 | 1.06 |
| Q62393 | TPD52 | 24.313 | 1.12 | 1.23 | 1.17 | 0.08 | -0.63 | 0.15 | -0.24 | 0.55 | 1.41 |
| Q9QYA2 | TOMM40 | 37.895 | 0.40 | 0.01 | 0.20 | 0.28 | -1.04 | 0.01 | -0.52 | 0.74 | 0.72 |
| Q8VBT0 | TMX1 | 31.395 | 0.32 | 0.30 | 0.31 | 0.01 | -0.54 | -0.41 | -0.47 | 0.09 | 0.78 |
| Q61029 | TMPO | 50.372 | 0.48 | 0.42 | 0.45 | 0.05 | -0.72 | -0.40 | -0.56 | 0.23 | 1.01 |
| P26039 | TLN1 | 269.82 | 0.83 | 1.33 | 1.08 | 0.35 | 0.11 | 0.40 | 0.26 | 0.20 | 0.83 |
| P35441 | THBS1 | 129.65 | 2.44 | 2.99 | 2.72 | 0.39 | 1.16 | 1.49 | 1.32 | 0.23 | 1.39 |
| P21981 | TGM2 | 77.06 | 0.89 | 0.79 | 0.84 | 0.07 | -0.10 | -0.16 | -0.13 | 0.04 | 0.97 |
| P82198 | TGFBI | 74.596 | 1.82 | 2.07 | 1.94 | 0.17 | 0.60 | 0.27 | 0.43 | 0.23 | 1.51 |
| Q62351 | TFRC | 85.73 | 1.20 | 0.98 | 1.09 | 0.16 | -0.31 | -0.59 | -0.45 | 0.20 | 1.54 |
| Q921I1 | TF | 76.723 | 0.80 | 0.85 | 0.82 | 0.03 | 0.06 | 0.11 | 0.08 | 0.03 | 0.74 |
| Q9R233 | TAPBP | 49.736 | 0.53 | 0.66 | 0.59 | 0.09 | -0.68 | -0.53 | -0.60 | 0.11 | 1.20 |

| Q9WVA4 | TAGLN2 | 22.395 | 1.73 | 1.74 | 1.73 | 0.00 | -0.13 | 0.01 | -0.06 | 0.10 | 1.79 |
| --- | --- | --- | --- | --- | --- | --- | --- | --- | --- | --- | --- |
| P37804 | TAGLN | 22.576 | 2.09 | 2.16 | 2.12 | 0.05 | 0.10 | 0.21 | 0.15 | 0.07 | 1.97 |
| Q9D5V6 | SYAP1 | 41.349 | 0.14 | 0.19 | 0.16 | 0.04 | -1.02 | -0.36 | -0.69 | 0.46 | 0.85 |
| Q6A028 | SWAP70 | 68.995 | 0.97 | 1.05 | 1.01 | 0.06 | 0.34 | 0.30 | 0.32 | 0.03 | 0.69 |
| P52843 | SULT2A1 | 33.213 | -4.40 | -4.52 | -4.46 | 0.08 | -4.85 | -2.42 | -3.63 | 1.72 | -0.83 |
| P52840 | SULT1A1 | 33.974 | -0.03 | -0.13 | -0.08 | 0.07 | -0.86 | -0.83 | -0.84 | 0.02 | 0.76 |
| Q7TNE1 | SUGCT | 47.69 | 1.25 | 1.15 | 1.20 | 0.07 | 0.52 | 0.34 | 0.43 | 0.13 | 0.77 |
| P11031 | SUB1 | 14.427 | 0.33 | 0.31 | 0.32 | 0.01 | -0.46 | -0.50 | -0.48 | 0.04 | 0.80 |
| O88983 | STX8 | 26.925 | 0.31 | 0.52 | 0.41 | 0.15 | -0.52 | -0.25 | -0.39 | 0.19 | 0.80 |
| O70439 | STX7 | 29.82 | 1.22 | 1.02 | 1.12 | 0.14 | 0.17 | -0.14 | 0.01 | 0.21 | 1.11 |
| Q9JI11 | STK4;STK3 | 55.541 | 0.72 | 0.92 | 0.82 | 0.14 | -0.53 | -0.25 | -0.39 | 0.20 | 1.21 |
| P42225 | STAT1 | 87.196 | 0.46 | 0.42 | 0.44 | 0.03 | -1.11 | -1.17 | -1.14 | 0.04 | 1.58 |
| Q3UTJ2 | SORBS2 | 132.35 | 0.07 | 0.70 | 0.38 | 0.45 | -0.84 | 0.19 | -0.33 | 0.73 | 0.71 |

| Q6P8X1 | SNX6 | 46.648 | 0.66 | 0.45 | 0.56 | 0.15 | -0.19 | -0.09 | -0.14 | 0.07 | 0.70 |
| --- | --- | --- | --- | --- | --- | --- | --- | --- | --- | --- | --- |
| Q9D8U8 | SNX5 | 46.797 | 0.93 | 0.89 | 0.91 | 0.02 | -0.15 | 0.10 | -0.03 | 0.17 | 0.94 |
| Q9CWK8 | SNX2 | 58.47 | 0.79 | 0.73 | 0.76 | 0.05 | -0.13 | 0.07 | -0.03 | 0.14 | 0.79 |
| Q9WV80 | SNX1 | 58.951 | 0.40 | 0.58 | 0.49 | 0.13 | -0.63 | 0.01 | -0.31 | 0.45 | 0.80 |
| Q6P5D8 | SMCHD1 | 225.65 | 0.38 | 0.61 | 0.49 | 0.16 | -0.55 | -0.73 | -0.64 | 0.13 | 1.13 |
| Q7TN29 | SMAP2 | 46.577 | -0.23 | 0.21 | -0.01 | 0.31 | -1.35 | -0.12 | -0.74 | 0.87 | 0.73 |
| Q9R0P4 | SMAP | 20.046 | 0.25 | 0.35 | 0.30 | 0.07 | -0.83 | 0.00 | -0.41 | 0.58 | 0.71 |
| P10852 | SLC3A2 | 58.336 | 1.48 | 1.80 | 1.64 | 0.22 | 0.09 | 0.09 | 0.09 | 0.00 | 1.55 |
| P58735 | SLC26A1 | 75.787 | -0.64 | -0.66 | -0.65 | 0.01 | 0.28 | 0.14 | 0.21 | 0.09 | -0.86 |
| P48962 | SLC25A4 | 32.904 | -1.16 | -1.19 | -1.18 | 0.02 | -2.24 | -2.39 | -2.31 | 0.11 | 1.14 |
| Q9JK48 | SH3GLB1 | 40.855 | 0.65 | 0.80 | 0.72 | 0.11 | -0.40 | -0.11 | -0.25 | 0.20 | 0.98 |
| Q62419 | SH3GL1 | 41.518 | -0.03 | 0.04 | 0.00 | 0.05 | -1.29 | -0.46 | -0.88 | 0.59 | 0.88 |
| Q9JJU8 | SH3BGRL | 12.811 | 1.08 | 1.02 | 1.05 | 0.04 | -0.34 | -0.20 | -0.27 | 0.10 | 1.32 |

| Q8R0X7 | SGPL1 | 63.676 | 0.33 | 0.37 | 0.35 | 0.02 | -0.48 | -0.19 | -0.34 | 0.21 | 0.69 |
| --- | --- | --- | --- | --- | --- | --- | --- | --- | --- | --- | --- |
| P19324 | SERPINH1 | 46.533 | 0.06 | -0.45 | -0.19 | 0.36 | -1.92 | -1.97 | -1.94 | 0.03 | 1.75 |
| Q60854 | SERPINB6 | 42.598 | 1.59 | 1.77 | 1.68 | 0.13 | -0.27 | 0.39 | 0.06 | 0.47 | 1.62 |
| Q9D154 | SERPINB1A | 42.574 | 2.14 | 2.25 | 2.19 | 0.08 | 0.55 | 0.82 | 0.69 | 0.19 | 1.51 |
| Q00898 | SERPINA1E | 45.891 | 3.76 | 0.80 | 2.28 | 2.09 | 5.03 | 0.96 | 3.00 | 2.88 | -0.72 |
| P13516 | SCD1 | 41.046 | -0.16 | -0.21 | -0.19 | 0.04 | -1.38 | -1.23 | -1.31 | 0.11 | 1.12 |
| Q60710 | SAMHD1 | 72.65 | 0.83 | 0.96 | 0.90 | 0.09 | 0.05 | 0.03 | 0.04 | 0.02 | 0.85 |
| P31725 | S100A9 | 13.049 | 3.64 | 3.69 | 3.66 | 0.04 | 2.14 | 2.25 | 2.19 | 0.08 | 1.47 |
| Q99P72 | RTN4 | 126.61 | 0.71 | 0.71 | 0.71 | 0.00 | -1.19 | -0.77 | -0.98 | 0.29 | 1.69 |
| Q8BVY0 | RSL1D1 | 50.421 | -0.28 | -0.28 | -0.28 | 0.00 | -1.27 | -1.02 | -1.15 | 0.18 | 0.87 |
| P07742 | RRM1 | 90.209 | -0.21 | -0.21 | -0.21 | 0.00 | -0.85 | -1.09 | -0.97 | 0.17 | 0.76 |
| P10833 | RRAS | 23.764 | 1.17 | 0.99 | 1.08 | 0.12 | -0.50 | 0.97 | 0.23 | 1.03 | 0.85 |
|  |  | 44.12 | 0.81 | 0.49 | 0.65 | 0.23 | -0.31 | 0.08 | -0.12 | 0.28 | 0.76 |

| Q00915 | RBP1 | 15.846 | 0.38 | 0.58 | 0.48 | 0.14 | -0.66 | -0.65 | -0.65 | 0.01 | 1.13 |
| --- | --- | --- | --- | --- | --- | --- | --- | --- | --- | --- | --- |
| Q64012 | RALY | 33.188 | 0.53 | 0.40 | 0.46 | 0.09 | -0.42 | -0.08 | -0.25 | 0.24 | 0.71 |
| Q9CZE3 | RAB32 | 25.068 | 1.14 | 1.03 | 1.09 | 0.08 | -0.14 | -0.13 | -0.14 | 0.00 | 1.22 |
| Q3U4I7 | PYROXD2 | 62.939 | 1.38 | 1.36 | 1.37 | 0.01 | 2.71 | 3.07 | 2.89 | 0.25 | -1.52 |
| Q8CI94 | PYGB | 96.729 | -0.40 | -0.45 | -0.43 | 0.04 | -1.67 | -1.74 | -1.70 | 0.05 | 1.28 |
| O54724 | PTRF | 43.953 | 0.37 | 0.39 | 0.38 | 0.02 | -0.54 | -0.21 | -0.37 | 0.23 | 0.75 |
| Q64455 | PTPRJ | 136.77 | 0.68 | 0.76 | 0.72 | 0.06 | 0.01 | -0.10 | -0.05 | 0.08 | 0.77 |
| P06800 | PTPRC | 144.6 | 1.88 | 2.36 | 2.12 | 0.34 | 0.06 | 0.38 | 0.22 | 0.22 | 1.90 |
| P29351 | PTPN6 | 67.558 | 1.50 | 1.82 | 1.66 | 0.23 | -0.33 | -0.05 | -0.19 | 0.20 | 1.84 |
| Q9JK53 | PRELP | 43.292 | 1.69 | 1.82 | 1.76 | 0.09 | 0.45 | 0.75 | 0.60 | 0.21 | 1.16 |
| Q8BVQ5 | PPME1 | 42.256 | 0.12 | 0.12 | 0.12 | 0.00 | -0.98 | -0.25 | -0.61 | 0.51 | 0.73 |
| Q9R0E2 | PLOD1 | 83.594 | 1.02 | 1.13 | 1.08 | 0.08 | -0.27 | -0.35 | -0.31 | 0.06 | 1.39 |
| P52480 | PKM | 57.844 | 2.05 | 1.80 | 1.93 | 0.17 | -0.05 | -0.18 | -0.12 | 0.09 | 2.04 |

| P53810 | PITPNA | 31.893 | 0.82 | 0.79 | 0.80 | 0.02 | 0.14 | 0.04 | 0.09 | 0.07 | 0.71 |
| --- | --- | --- | --- | --- | --- | --- | --- | --- | --- | --- | --- |
| Q9EQ32 | PIK3AP1 | 90.927 | 0.32 | 0.67 | 0.49 | 0.25 | -0.63 | 0.19 | -0.22 | 0.58 | 0.71 |
| Q9DCD0 | PGD | 53.247 | -0.05 | -0.21 | -0.13 | 0.11 | -1.27 | -1.19 | -1.23 | 0.05 | 1.10 |
| P62962 | PFN1 | 14.957 | 0.64 | 0.73 | 0.69 | 0.07 | -0.20 | -0.16 | -0.18 | 0.03 | 0.87 |
| Q9EQ61 | PES1 | 67.795 | 0.34 | 0.17 | 0.25 | 0.12 | -0.61 | -0.51 | -0.56 | 0.07 | 0.81 |
| Q8CI51 | PDLIM5 | 63.299 | -0.37 | -0.10 | -0.24 | 0.19 | -1.03 | -0.84 | -0.94 | 0.13 | 0.70 |
| O70400 | PDLIM1 | 35.774 | 0.93 | 0.81 | 0.87 | 0.09 | -0.32 | -0.09 | -0.21 | 0.16 | 1.08 |
| Q9JHU2 | PALMD | 62.699 | -0.12 | 0.00 | -0.06 | 0.09 | -1.34 | -0.44 | -0.89 | 0.64 | 0.83 |
| Q8CIN4 | PAK2 | 57.93 | 0.56 | 0.68 | 0.62 | 0.08 | -0.81 | -0.26 | -0.53 | 0.39 | 1.16 |
| Q60715 | P4HA1 | 60.909 | 0.76 | 0.70 | 0.73 | 0.04 | -0.97 | -1.04 | -1.01 | 0.05 | 1.74 |
| Q62422 | OSTF1 | 23.782 | 0.86 | 0.81 | 0.83 | 0.03 | -0.34 | -0.21 | -0.27 | 0.10 | 1.11 |
| Q61503 | NT5E | 63.864 | 0.17 | 0.17 | 0.17 | 0.00 | -1.29 | -0.33 | -0.81 | 0.68 | 0.98 |
| Q9R1J0 | NSDHL | 40.685 | 0.53 | 0.31 | 0.42 | 0.16 | -0.37 | -0.24 | -0.30 | 0.09 | 0.73 |

| Q64669 | NQO1 | 30.959 | 0.70 | 0.65 | 0.67 | 0.03 | -0.58 | -0.48 | -0.53 | 0.08 | 1.20 |
| --- | --- | --- | --- | --- | --- | --- | --- | --- | --- | --- | --- |
| Q9DCJ9 | NPL | 35.13 | 1.46 | 1.44 | 1.45 | 0.01 | 0.07 | 0.02 | 0.04 | 0.04 | 1.41 |
| P28656 | NAP1L1 | 45.345 | 0.60 | 0.52 | 0.56 | 0.05 | -0.25 | -0.18 | -0.22 | 0.05 | 0.78 |
| Q99KQ4 | NAMPT | 55.446 | 0.32 | 0.39 | 0.36 | 0.05 | -0.51 | -0.23 | -0.37 | 0.20 | 0.73 |
| Q9QZ08 | NAGK | 37.268 | 0.78 | 0.67 | 0.72 | 0.07 | -0.30 | -0.05 | -0.18 | 0.18 | 0.90 |
| Q6PDN3 | MYLK | 212.92 | 0.44 | 0.84 | 0.64 | 0.28 | -0.30 | -0.05 | -0.18 | 0.18 | 0.81 |
| Q60605 | MYL6 | 16.93 | 0.78 | 1.02 | 0.90 | 0.17 | 0.02 | 0.11 | 0.07 | 0.06 | 0.83 |
|  |  | 19.779 | 0.86 | 0.85 | 0.85 | 0.01 | -0.05 | 0.26 | 0.10 | 0.22 | 0.75 |
| Q8VDD5 | MYH9 | 226.37 | 0.74 | 1.04 | 0.89 | 0.21 | -0.04 | 0.17 | 0.06 | 0.15 | 0.82 |
| Q91Z83 | MYH7 | 222.88 | -6.28 | -5.07 | -5.67 | 0.85 | -4.71 | -4.53 | -4.62 | 0.13 | -1.06 |
| O08638 | MYH11 | 227.03 | 1.16 | 1.68 | 1.42 | 0.37 | 0.49 | 0.67 | 0.58 | 0.12 | 0.84 |
| P11589 | MUP2 | 20.663 | 1.74 | 3.21 | 2.47 | 1.04 | 2.89 | 4.09 | 3.49 | 0.85 | -1.02 |
| P28666 | MUG2 | 162.38 | -0.31 | 0.02 | -0.14 | 0.23 | -1.57 | -1.20 | -1.38 | 0.26 | 1.24 |

| P28665 | MUG1 | 165.3 | 0.35 | 0.56 | 0.46 | 0.15 | 1.14 | 1.33 | 1.24 | 0.14 | -0.78 |
| --- | --- | --- | --- | --- | --- | --- | --- | --- | --- | --- | --- |
| Q9Z2C5 | MTM1 | 69.558 | 0.86 | 0.64 | 0.75 | 0.15 | -0.01 | -0.02 | -0.01 | 0.01 | 0.76 |
| Q80WJ7 | MTDH | 63.845 | -0.22 | 0.12 | -0.05 | 0.24 | -1.49 | -0.40 | -0.94 | 0.77 | 0.89 |
| P26041 | MSN | 67.766 | 1.88 | 1.60 | 1.74 | 0.20 | 0.19 | 0.18 | 0.18 | 0.01 | 1.56 |
| Q99J99 | MPST | 33.023 | 0.23 | 0.04 | 0.13 | 0.13 | 1.19 | 1.11 | 1.15 | 0.06 | -1.02 |
| Q9CWP6 | MOSPD2 | 59.854 | 0.35 | 0.49 | 0.42 | 0.10 | -1.22 | -0.37 | -0.79 | 0.60 | 1.22 |
| Q9Z2D6 | MECP2 | 52.307 | 0.73 | 1.05 | 0.89 | 0.23 | -0.81 | 0.73 | -0.04 | 1.09 | 0.93 |
| P06801 | ME1 | 63.953 | -1.79 | -1.83 | -1.81 | 0.03 | -2.86 | -2.19 | -2.52 | 0.47 | 0.71 |
| P97287 | MCL1 | 35.217 | 0.59 | 0.62 | 0.60 | 0.02 | -1.16 | -0.34 | -0.75 | 0.58 | 1.35 |
| Q3THS6 | MAT2A | 43.688 | 1.05 | 0.52 | 0.79 | 0.37 | 0.00 | 0.04 | 0.02 | 0.03 | 0.77 |
| P26645 | MARCKS | 29.661 | 1.27 | 1.21 | 1.24 | 0.04 | -0.59 | -0.38 | -0.49 | 0.15 | 1.73 |
| Q61166 | MAPRE1 | 30.016 | 0.57 | 0.42 | 0.50 | 0.10 | -0.63 | -0.36 | -0.50 | 0.19 | 0.99 |
| O09159 | MAN2B1 | 114.65 | 1.15 | 1.06 | 1.11 | 0.06 | 0.32 | 0.20 | 0.26 | 0.09 | 0.85 |

| P25911 | LYN | 58.812 | 0.91 | 0.85 | 0.88 | 0.04 | 0.08 | 0.26 | 0.17 | 0.13 | 0.71 |
| --- | --- | --- | --- | --- | --- | --- | --- | --- | --- | --- | --- |
| P51885 | LUM | 38.265 | 0.99 | 1.37 | 1.18 | 0.27 | 0.07 | 0.32 | 0.19 | 0.18 | 0.99 |
| Q8BFW7 | LPP | 65.89 | 0.26 | 0.45 | 0.35 | 0.14 | -0.88 | -0.07 | -0.47 | 0.58 | 0.83 |
| Q8C129 | LNPEP | 117.3 | 0.81 | 1.13 | 0.97 | 0.22 | -0.10 | 0.01 | -0.04 | 0.07 | 1.01 |
| O89017 | LGMN | 49.372 | 1.80 | 1.69 | 1.74 | 0.07 | 0.96 | 1.01 | 0.99 | 0.03 | 0.76 |
| Q07797 | LGALS3BP | 64.49 | 0.65 | 0.85 | 0.75 | 0.14 | 0.02 | -0.48 | -0.23 | 0.35 | 0.98 |
| Q7TNG8 | LDHD | 51.847 | 1.36 | 1.41 | 1.38 | 0.04 | 2.17 | 2.20 | 2.19 | 0.02 | -0.80 |
| Q61233 | LCP1 | 70.148 | 1.55 | 1.58 | 1.57 | 0.02 | 0.46 | 0.37 | 0.41 | 0.07 | 1.15 |
| Q3U9G9 | LBR | 71.439 | 0.39 | 0.36 | 0.38 | 0.02 | -0.97 | -0.64 | -0.80 | 0.23 | 1.18 |
| Q61792 | LASP1 | 29.994 | 0.66 | 0.70 | 0.68 | 0.03 | -0.38 | 0.11 | -0.14 | 0.35 | 0.82 |
| P11438 | LAMP1 | 43.865 | 1.74 | 1.09 | 1.42 | 0.46 | 0.69 | 0.38 | 0.54 | 0.22 | 0.88 |
| P02469 | LAMB1 | 197.09 | -0.46 | 0.04 | -0.21 | 0.35 | -1.65 | -1.48 | -1.56 | 0.12 | 1.36 |
| Q8K2Q9 | KIAA1598 | 71.342 | 0.24 | 0.68 | 0.46 | 0.31 | -0.88 | -0.08 | -0.48 | 0.57 | 0.94 |

| Q9DCY0 | KEG1 | 33.723 | 1.00 | 0.94 | 0.97 | 0.04 | 2.11 | 1.91 | 2.01 | 0.14 | -1.04 |
| --- | --- | --- | --- | --- | --- | --- | --- | --- | --- | --- | --- |
| Q9JKF1 | IQGAP1 | 188.74 | 1.71 | 1.75 | 1.73 | 0.03 | -0.03 | 0.07 | 0.02 | 0.07 | 1.71 |
| P01867 | IGH-3 | 44.259 | 4.15 | 4.08 | 4.11 | 0.05 | 4.96 | 4.94 | 4.95 | 0.02 | -0.84 |
| Q07113 | IGF2R | 273.81 | 0.42 | 0.33 | 0.37 | 0.06 | -0.11 | -0.55 | -0.33 | 0.31 | 0.70 |
| Q61249 | IGBP1 | 38.97 | 0.01 | -0.03 | -0.01 | 0.02 | -1.03 | -0.36 | -0.70 | 0.47 | 0.69 |
| P58044 | IDI1 | 26.289 | 0.34 | 0.40 | 0.37 | 0.04 | -0.66 | -0.59 | -0.62 | 0.05 | 0.99 |
| Q05793 | HSPG2 | 398.29 | 0.60 | 1.00 | 0.80 | 0.28 | -0.10 | 0.27 | 0.08 | 0.26 | 0.72 |
| Q61696 |  |  | -0.05 | -0.01 | -0.03 | 0.03 | -0.65 | -0.85 | -0.75 | 0.14 | 0.72 |
|  |  |  | -0.58 | -0.63 | -0.61 | 0.04 | -1.38 | -1.25 | -1.32 | 0.09 | 0.71 |
| P00493 | HPRT1 | 24.57 | 1.19 | 1.28 | 1.24 | 0.06 | 0.24 | 0.34 | 0.29 | 0.07 | 0.95 |
| P49312 | HNRNPA1 | 34.196 | 0.82 | 0.70 | 0.76 | 0.08 | 0.08 | 0.02 | 0.05 | 0.04 | 0.72 |
| P14901 | HMOX1 | 32.928 | 2.52 | 2.51 | 2.52 | 0.00 | 0.06 | 0.36 | 0.21 | 0.21 | 2.30 |
| Q8JZK9 | HMGCS1 | 57.568 | -0.15 | -0.10 | -0.12 | 0.03 | -1.99 | -1.74 | -1.86 | 0.18 | 1.74 |

| P30681 | HMGB2 | 24.162 | 1.03 | 1.04 | 1.04 | 0.00 | -0.57 | -0.56 | -0.57 | 0.00 | 1.60 |
| --- | --- | --- | --- | --- | --- | --- | --- | --- | --- | --- | --- |
| P43277 | HIST1H1D | 22.099 | 0.81 | 0.71 | 0.76 | 0.07 | -0.09 | 0.15 | 0.03 | 0.17 | 0.73 |
| P15864 | HIST1H1C | 21.266 | 1.05 | 1.03 | 1.04 | 0.01 | -0.20 | -0.17 | -0.18 | 0.02 | 1.22 |
| P43276 | HIST1H1B | 22.576 | -0.03 | -0.18 | -0.11 | 0.11 | -2.80 | -2.90 | -2.85 | 0.07 | 2.74 |
| P43275 | HIST1H1A | 21.785 | 0.81 | 0.63 | 0.72 | 0.13 | -1.69 | -1.51 | -1.60 | 0.12 | 2.31 |
| Q8VD75 | HIP1 | 115.2 | 1.02 | 1.40 | 1.21 | 0.27 | 0.49 | 0.18 | 0.33 | 0.22 | 0.87 |
| Q9R257 | HEBP1 | 21.067 | 0.39 | 0.42 | 0.41 | 0.02 | 1.21 | 1.56 | 1.38 | 0.25 | -0.98 |
| Q9WU19 | HAO1 | 41.001 | -0.76 | -0.67 | -0.71 | 0.06 | 0.23 | 0.28 | 0.26 | 0.03 | -0.97 |
| P19157 | GSTP1 | 23.609 | 2.15 | 2.07 | 2.11 | 0.06 | 3.01 | 3.27 | 3.14 | 0.19 | -1.03 |
| O35660 | GSTM6 | 25.621 | 0.80 | 0.86 | 0.83 | 0.04 | -0.49 | -0.43 | -0.46 | 0.05 | 1.29 |
| P19639 | GSTM3 | 25.701 | 2.76 | 2.91 | 2.83 | 0.10 | 0.16 | 0.24 | 0.20 | 0.05 | 2.64 |
| P15626 | GSTM2 | 25.716 | 0.89 | 1.04 | 0.97 | 0.10 | 0.18 | 0.21 | 0.20 | 0.02 | 0.77 |
| P10648 | GSTA2 | 25.542 | 3.54 | 3.71 | 3.62 | 0.12 | 2.56 | 2.80 | 2.68 | 0.17 | 0.95 |

| O88958 | GNPDA1 | 32.549 | 0.92 | 0.83 | 0.87 | 0.07 | 0.12 | 0.00 | 0.06 | 0.08 | 0.81 |
| --- | --- | --- | --- | --- | --- | --- | --- | --- | --- | --- | --- |
| P21279 | GNAQ | 42.158 | 1.18 | 0.47 | 0.83 | 0.50 | 0.13 | 0.15 | 0.14 | 0.01 | 0.69 |
| Q91XE0 | GLYAT | 34.098 | 0.36 | 0.19 | 0.28 | 0.12 | 1.08 | 0.92 | 1.00 | 0.11 | -0.72 |
| Q9CPU0 | GLO1 | 20.809 | 0.79 | 0.85 | 0.82 | 0.04 | 1.90 | 1.98 | 1.94 | 0.05 | -1.12 |
| P23780 | GLB1 | 73.12 | 0.99 | 1.20 | 1.09 | 0.15 | -0.58 | -0.29 | -0.43 | 0.21 | 1.53 |
| Q8R0H9 | GGA1 | 69.971 | -0.07 | 0.53 | 0.23 | 0.42 | -1.26 | -0.27 | -0.76 | 0.70 | 1.00 |
| Q9Z0E6 | GBP2 | 66.739 | 0.78 | 0.81 | 0.80 | 0.02 | -0.96 | -1.18 | -1.07 | 0.16 | 1.87 |
| Q8BVW0 | GANC | 102.01 | 0.46 | 0.75 | 0.60 | 0.21 | 1.78 | 1.50 | 1.64 | 0.20 | -1.03 |
| Q00612 | G6PDX | 59.262 | 1.69 | 1.75 | 1.72 | 0.04 | -0.49 | -0.44 | -0.47 | 0.03 | 2.19 |
| Q91WJ8 | FUBP1 | 68.539 | 0.20 | 0.25 | 0.22 | 0.04 | -0.57 | -0.49 | -0.53 | 0.06 | 0.75 |
| P11276 | FN1 | 272.53 | 0.86 | 1.05 | 0.95 | 0.13 | 0.03 | 0.18 | 0.11 | 0.11 | 0.85 |
| P97501 | FMO3 | 60.515 | -1.50 | -1.30 | -1.40 | 0.14 | -4.81 | -4.58 | -4.70 | 0.17 | 3.29 |
| Q8BTM8 | FLNA | 281.22 | 1.68 | 1.96 | 1.82 | 0.19 | 0.21 | 0.47 | 0.34 | 0.18 | 1.48 |

| Q9JJ28 | FLII | 144.8 | 0.27 | 0.47 | 0.37 | 0.14 | -0.73 | 0.05 | -0.34 | 0.56 | 0.71 |
| --- | --- | --- | --- | --- | --- | --- | --- | --- | --- | --- | --- |
| P39749 | FEN1 | 42.314 | 1.27 | 1.23 | 1.25 | 0.03 | -1.06 | -0.51 | -0.78 | 0.38 | 2.04 |
| Q920E5 | FDPS | 40.581 | 0.39 | 0.14 | 0.27 | 0.18 | -0.59 | -0.52 | -0.55 | 0.05 | 0.82 |
| Q05816 | FABP5 | 15.137 | -0.96 | -1.11 | -1.04 | 0.11 | -1.69 | -1.83 | -1.76 | 0.10 | 0.72 |
| P26040 | EZR | 69.406 | 1.50 | 1.55 | 1.53 | 0.04 | -0.36 | 0.35 | 0.00 | 0.50 | 1.53 |
| O70318 | EPB41L2 | 109.94 | -0.07 | 0.50 | 0.22 | 0.40 | -1.20 | -0.10 | -0.65 | 0.78 | 0.86 |
| Q9EQP2 | EHD4 | 61.48 | 1.14 | 1.23 | 1.18 | 0.06 | -0.25 | -0.16 | -0.21 | 0.07 | 1.39 |
| Q9WVK4 | EHD1 | 60.602 | 0.92 | 0.97 | 0.94 | 0.03 | -0.12 | 0.14 | 0.01 | 0.19 | 0.93 |
| Q9D8Y0 | EFHD2 | 26.791 | 1.41 | 1.36 | 1.39 | 0.03 | 0.25 | 0.65 | 0.45 | 0.28 | 0.94 |
| Q8R1Q8 | DYNC1LI1 | 56.614 | 0.33 | 0.20 | 0.27 | 0.09 | -1.13 | -0.26 | -0.70 | 0.62 | 0.96 |
| O08553 | DPYSL2 | 62.277 | 0.97 | 1.18 | 1.08 | 0.15 | 0.01 | 0.24 | 0.13 | 0.16 | 0.95 |
| Q9EQF5 | DPYS | 56.724 | -0.68 | -0.58 | -0.63 | 0.07 | 0.05 | 0.27 | 0.16 | 0.15 | -0.80 |
| A2ATU0 | DHTKD1 | 102.79 | 1.02 | 0.88 | 0.95 | 0.10 | 2.18 | 2.02 | 2.10 | 0.11 | -1.15 |

| P31001 | DES | 53.497 | 0.55 | 0.67 | 0.61 | 0.08 | -0.75 | -0.44 | -0.59 | 0.22 | 1.20 |
| --- | --- | --- | --- | --- | --- | --- | --- | --- | --- | --- | --- |
| Q62418 | DBNL | 48.699 | 0.67 | 0.75 | 0.71 | 0.05 | -0.85 | -0.12 | -0.49 | 0.51 | 1.19 |
| Q60991 | CYP7B1 | 58.47 | 3.84 | 3.44 | 3.64 | 0.29 | 5.37 | 5.32 | 5.34 | 0.04 | -1.70 |
| Q8K0C4 | CYP51A1 | 56.775 | 0.09 | 0.25 | 0.17 | 0.11 | -0.65 | -0.42 | -0.53 | 0.17 | 0.71 |
| O88833 | CYP4A10 | 58.33 | 1.50 | 1.55 | 1.52 | 0.04 | 0.63 | 0.82 | 0.72 | 0.13 | 0.80 |
| P12790 | CYP2B9 | 55.74 | -1.59 | -1.65 | -1.62 | 0.04 | -3.99 | -3.97 | -3.98 | 0.01 | 2.36 |
| Q9CX80 | CYGB | 21.465 | 1.96 | 1.91 | 1.93 | 0.04 | 0.93 | 1.17 | 1.05 | 0.18 | 0.88 |
| P97792 | CXADR | 39.947 | -0.13 | -0.06 | -0.09 | 0.05 | -1.60 | -0.68 | -1.14 | 0.65 | 1.04 |
| O88712 | CTBP1 | 47.744 | 0.44 | 0.44 | 0.44 | 0.00 | -0.42 | -0.09 | -0.25 | 0.23 | 0.70 |
| Q9WVJ3 | CPQ | 51.813 | 1.04 | 0.96 | 1.00 | 0.05 | 0.08 | 0.25 | 0.17 | 0.12 | 0.83 |
| Q8C166 | CPNE1 | 58.886 | 0.41 | 0.26 | 0.34 | 0.10 | -0.44 | -0.35 | -0.40 | 0.06 | 0.73 |
| Q9CQI6 | COTL1 | 15.944 | 1.89 | 1.80 | 1.85 | 0.07 | -0.09 | -0.12 | -0.10 | 0.03 | 1.95 |
| Q9WUM4 | CORO1C | 53.12 | 0.96 | 0.87 | 0.91 | 0.07 | 0.07 | 0.00 | 0.03 | 0.05 | 0.88 |

| O89053 | CORO1A | 50.989 | 2.61 | 2.65 | 2.63 | 0.03 | 0.26 | 0.46 | 0.36 | 0.14 | 2.27 |
| --- | --- | --- | --- | --- | --- | --- | --- | --- | --- | --- | --- |
| Q8K297 | COLGALT1 | 71.06 | 1.07 | 0.97 | 1.02 | 0.07 | -0.20 | 0.04 | -0.08 | 0.17 | 1.10 |
| Q04857 | COL6A1 | 108.49 | 1.76 | 1.84 | 1.80 | 0.06 | 1.22 | 0.84 | 1.03 | 0.26 | 0.77 |
| Q80X19 | COL14A1 | 193.01 | 0.86 | 1.48 | 1.17 | 0.44 | -0.68 | -0.51 | -0.59 | 0.12 | 1.76 |
| Q9D1A2 | CNDP2 | 52.767 | 0.65 | 0.57 | 0.61 | 0.06 | -0.78 | -0.66 | -0.72 | 0.09 | 1.32 |
| Q3U5Q7 | CMPK2 | 50.036 | 0.90 | 0.74 | 0.82 | 0.11 | -1.12 | -1.25 | -1.19 | 0.09 | 2.00 |
| Q61419 | CMAH | 66.935 | -0.73 | -0.83 | -0.78 | 0.07 | 0.31 | 0.12 | 0.22 | 0.13 | -1.00 |
| Q9Z1Q5 | CLIC1 | 27.013 | 1.34 | 1.31 | 1.32 | 0.02 | 0.25 | 0.19 | 0.22 | 0.04 | 1.10 |
| P70194 | CLEC4F | 61.268 | 1.31 | 1.49 | 1.40 | 0.13 | 0.22 | 0.17 | 0.20 | 0.04 | 1.20 |
| Q04447 | CKB | 42.713 | 1.55 | 1.37 | 1.46 | 0.13 | -0.25 | -0.36 | -0.31 | 0.08 | 1.77 |
| Q8BMK4 | CKAP4 | 63.691 | 1.02 | 1.04 | 1.03 | 0.01 | -0.64 | -0.61 | -0.62 | 0.02 | 1.66 |
| Q9D8B3 |  | 24.936 | 0.72 | 0.81 | 0.77 | 0.07 | -0.75 | 0.25 | -0.25 | 0.71 | 1.02 |
| O35744 | CHIL3 | 44.458 | 4.06 | 4.37 | 4.21 | 0.22 | 5.33 | 5.89 | 5.61 | 0.39 | -1.40 |

| P18760 | CFL1 | 18.559 | 0.76 | 0.91 | 0.83 | 0.11 | -0.03 | 0.01 | -0.01 | 0.03 | 0.85 |
| --- | --- | --- | --- | --- | --- | --- | --- | --- | --- | --- | --- |
| Q8VCU1 | CES3B | 63.352 | 0.37 | 0.24 | 0.31 | 0.09 | 1.50 | 1.38 | 1.44 | 0.09 | -1.13 |
| Q8BK48 | CES2E | 62.317 | -0.20 | -0.24 | -0.22 | 0.03 | -1.17 | -1.11 | -1.14 | 0.04 | 0.92 |
| Q8VCC2 | CES1 | 62.679 | 0.11 | 0.00 | 0.06 | 0.08 | -1.16 | -1.07 | -1.12 | 0.06 | 1.17 |
| P60766 | CDC42 | 21.258 | 0.82 | 0.75 | 0.79 | 0.05 | -0.01 | 0.10 | 0.04 | 0.08 | 0.74 |
| Q61735 | CD47 | 33.097 | 1.09 | 0.84 | 0.97 | 0.18 | 0.27 | 0.16 | 0.21 | 0.07 | 0.75 |
| Q08857 | CD36 | 52.697 | 0.80 | 0.95 | 0.88 | 0.10 | -0.92 | -0.88 | -0.90 | 0.03 | 1.77 |
| Q9JLQ0 | CD2AP | 70.449 | 0.45 | 0.54 | 0.50 | 0.07 | -1.48 | -0.39 | -0.93 | 0.77 | 1.43 |
| P51125 | CAST | 84.921 | -0.14 | 0.40 | 0.13 | 0.38 | -1.68 | -0.71 | -1.20 | 0.69 | 1.33 |
| O89110 | CASP8 | 55.356 | 0.15 | 0.20 | 0.18 | 0.04 | -0.66 | -0.51 | -0.58 | 0.11 | 0.76 |
| P47753 | CAPZA1 | 32.939 | 0.60 | 0.48 | 0.54 | 0.08 | -0.28 | -0.05 | -0.16 | 0.16 | 0.70 |
| O88456 | CAPNS1 | 28.463 | 0.72 | 0.65 | 0.68 | 0.05 | -0.59 | 0.01 | -0.29 | 0.43 | 0.97 |
| O08529 | CAPN2 | 79.871 | 0.61 | 0.47 | 0.54 | 0.10 | -0.16 | -0.33 | -0.24 | 0.13 | 0.78 |

| P24452 | CAPG | 39.24 | 4.15 | 4.01 | 4.08 | 0.10 | 0.98 | 1.11 | 1.04 | 0.09 | 3.03 |
| --- | --- | --- | --- | --- | --- | --- | --- | --- | --- | --- | --- |
| P40124 | CAP1 | 51.564 | 0.81 | 0.91 | 0.86 | 0.07 | -0.28 | -0.06 | -0.17 | 0.16 | 1.03 |
| P23589 | CA5A | 34.072 | -0.49 | -0.65 | -0.57 | 0.11 | 0.14 | 0.19 | 0.17 | 0.04 | -0.74 |
| P06684 | C5 | 188.88 | 0.18 | 0.63 | 0.40 | 0.32 | 1.29 | 1.43 | 1.36 | 0.10 | -0.96 |
| Q8R2Q8 | BST2 | 19.152 | 0.65 | 0.06 | 0.36 | 0.41 | -1.42 | -1.26 | -1.34 | 0.11 | 1.70 |
| Q9CY64 | BLVRA | 33.524 | 0.77 | 0.70 | 0.73 | 0.05 | 0.10 | -0.16 | -0.03 | 0.19 | 0.76 |
| O08539 | BIN1 | 64.469 | 0.62 | 0.64 | 0.63 | 0.02 | -0.56 | 0.08 | -0.24 | 0.45 | 0.87 |
| P28653 | BGN | 41.639 | 0.76 | 1.83 | 1.29 | 0.75 | -0.08 | 0.74 | 0.33 | 0.58 | 0.97 |
| Q6P3A8 | BCKDHB | 42.88 | -0.07 | -0.05 | -0.06 | 0.01 | 0.93 | 0.79 | 0.86 | 0.10 | -0.92 |
| P50136 | BCKDHA | 50.37 | -0.09 | -0.07 | -0.08 | 0.01 | 0.55 | 0.83 | 0.69 | 0.20 | -0.77 |
| Q9Z1R2 | BAG6 | 121.04 | -0.15 | 0.31 | 0.08 | 0.32 | -1.10 | -0.30 | -0.70 | 0.57 | 0.78 |
| Q9JLV1 | BAG3 | 61.859 | -0.19 | 0.05 | -0.07 | 0.17 | -1.43 | -0.27 | -0.85 | 0.82 | 0.78 |
| Q8BVE3 | ATP6V1H | 55.854 | 0.53 | 0.52 | 0.52 | 0.01 | -0.30 | -0.04 | -0.17 | 0.18 | 0.70 |

| P50518 | ATP6V1E1 | 26.157 | 0.98 | 0.84 | 0.91 | 0.10 | 0.02 | 0.03 | 0.02 | 0.01 | 0.89 |
| --- | --- | --- | --- | --- | --- | --- | --- | --- | --- | --- | --- |
| Q9Z1G3 | ATP6V1C1 | 43.887 | 1.35 | 1.18 | 1.26 | 0.12 | 0.08 | 0.04 | 0.06 | 0.03 | 1.20 |
| P62814 | ATP6V1B2 | 56.55 | 0.76 | 0.84 | 0.80 | 0.06 | -0.37 | -0.03 | -0.20 | 0.24 | 1.00 |
| P50516 | ATP6V1A | 68.325 | 0.77 | 0.76 | 0.76 | 0.01 | 0.04 | -0.05 | -0.01 | 0.06 | 0.77 |
| P63082 | ATP6V0C | 15.808 | 1.65 | 1.72 | 1.69 | 0.05 | 0.72 | 0.98 | 0.85 | 0.19 | 0.84 |
| Q9QZW0 | ATP11C | 129.24 | -0.48 | 0.00 | -0.24 | 0.34 | 0.50 | 0.71 | 0.60 | 0.15 | -0.85 |
| Q9CPW4 | ARPC5 | 16.288 | 0.82 | 0.82 | 0.82 | 0.01 | -0.19 | 0.12 | -0.03 | 0.22 | 0.85 |
| Q9WV32 | ARPC1B | 41.063 | 1.18 | 0.86 | 1.02 | 0.22 | 0.19 | 0.04 | 0.11 | 0.10 | 0.91 |
| Q61599 | ARHGDIB | 22.851 | 2.03 | 2.13 | 2.08 | 0.07 | -0.13 | 0.07 | -0.03 | 0.14 | 2.11 |
| Q99PT1 | ARHGDIA | 23.407 | 0.87 | 0.91 | 0.89 | 0.03 | -0.07 | 0.03 | -0.02 | 0.07 | 0.91 |
| Q5FWK3 | ARHGAP1 | 50.41 | 0.76 | 0.67 | 0.71 | 0.06 | -0.32 | -0.06 | -0.19 | 0.18 | 0.90 |
| Q9EPJ9 | ARFGAP1 | 45.288 | -0.16 | -0.02 | -0.09 | 0.10 | -1.14 | -0.52 | -0.83 | 0.44 | 0.74 |
| P48036 | ANXA5 | 35.752 | 1.35 | 1.12 | 1.24 | 0.16 | -0.29 | -0.34 | -0.32 | 0.04 | 1.55 |

| O35639 | ANXA3 | 36.384 | 1.12 | 0.98 | 1.05 | 0.10 | -0.04 | -0.05 | -0.05 | 0.00 | 1.10 |
| --- | --- | --- | --- | --- | --- | --- | --- | --- | --- | --- | --- |
| P07356 | ANXA2 | 38.676 | 1.44 | 1.19 | 1.31 | 0.18 | 0.07 | -0.02 | 0.02 | 0.06 | 1.29 |
| P10107 | ANXA1 | 38.734 | 2.33 | 2.33 | 2.33 | 0.00 | 0.24 | 0.74 | 0.49 | 0.35 | 1.84 |
| P05063 | ALDOC | 39.394 | 1.10 | 1.08 | 1.09 | 0.01 | 0.19 | 0.07 | 0.13 | 0.08 | 0.96 |
| P05064 | ALDOA | 39.355 | 0.93 | 0.69 | 0.81 | 0.17 | -0.26 | -0.35 | -0.31 | 0.06 | 1.12 |
| P47740 | ALDH3A2 | 53.97 | 0.13 | 0.25 | 0.19 | 0.08 | -0.89 | -0.67 | -0.78 | 0.16 | 0.97 |
| P21300 | AKR1B7 | 35.988 | 0.67 | 0.24 | 0.46 | 0.31 | -1.29 | -2.39 | -1.84 | 0.78 | 2.30 |
| P45376 | AKR1B1 | 35.732 | 1.29 | 1.13 | 1.21 | 0.11 | 0.02 | -0.10 | -0.04 | 0.09 | 1.25 |
| Q9WTQ5 | AKAP12 | 180.69 | -0.64 | -0.11 | -0.37 | 0.37 | -2.09 | -0.82 | -1.45 | 0.89 | 1.08 |
| Q9R0Y5 | AK1 | 21.539 | -2.00 | -1.95 | -1.98 | 0.03 | -3.36 | -3.39 | -3.37 | 0.03 | 1.40 |
| O08915 | AIP | 37.605 | 0.71 | 0.48 | 0.59 | 0.16 | -0.39 | -0.21 | -0.30 | 0.13 | 0.89 |
| Q8K2K6 | AGFG1 | 58.042 | -0.47 | 0.44 | -0.02 | 0.64 | -1.41 | -0.20 | -0.80 | 0.86 | 0.78 |
| Q8K4H1 | AFMID | 34.228 | -1.00 | 0.09 | -0.46 | 0.78 | 1.03 | 1.02 | 1.02 | 0.01 | -1.48 |

| P63260 |  | 41.792 | 1.16 | 1.02 | 1.09 | 0.10 | 0.29 | 0.21 | 0.25 | 0.06 | 0.84 |
| --- | --- | --- | --- | --- | --- | --- | --- | --- | --- | --- | --- |
|  |  | 42.009 | 1.94 | 1.82 | 1.88 | 0.09 | 0.51 | 0.56 | 0.54 | 0.04 | 1.35 |
| P68134 | ACTA1 | 42.051 | 1.11 | 1.01 | 1.06 | 0.07 | 0.13 | 0.14 | 0.13 | 0.00 | 0.92 |
| Q14DH7 | ACSS3 | 74.517 | -1.17 | -1.18 | -1.18 | 0.01 | -2.18 | -2.28 | -2.23 | 0.07 | 1.05 |
| Q91VA0 | ACSM1 | 64.76 | 0.15 | 0.09 | 0.12 | 0.04 | 1.02 | 0.85 | 0.93 | 0.12 | -0.81 |
| Q9QUJ7 | ACSL4 | 79.076 | 0.82 | 0.76 | 0.79 | 0.04 | -0.24 | -0.12 | -0.18 | 0.08 | 0.97 |
| Q9R0X4 | ACOT9 | 50.56 | 1.83 | 1.82 | 1.83 | 0.01 | 0.02 | 0.11 | 0.07 | 0.06 | 1.76 |
| Q9QYR7 | ACOT3 | 47.489 | -0.75 | -0.65 | -0.70 | 0.07 | -2.16 | -1.85 | -2.01 | 0.22 | 1.31 |
| Q9QYR9 | ACOT2 | 49.656 | 0.57 | 0.53 | 0.55 | 0.03 | -0.44 | -0.48 | -0.46 | 0.03 | 1.00 |
| Q5SWU9 | ACACA | 265.25 | -1.54 | -1.12 | -1.33 | 0.30 | -2.06 | -1.99 | -2.02 | 0.05 | 0.70 |
| Q99LR1 | ABHD12 | 45.269 | 1.39 | 1.36 | 1.37 | 0.02 | -0.25 | 0.04 | -0.11 | 0.21 | 1.48 |
| Q61285 | ABCD2 | 83.482 | -1.06 | -1.16 | -1.11 | 0.07 | -1.14 | -3.62 | -2.38 | 1.76 | 1.27 |
| Q8C650 | SETP-4 | 52.422 | 0.55 | 0.34 | 0.45 | 0.15 | -0.44 | -0.26 | -0.35 | 0.13 | 0.80 |

| Q80UG5 | SETP-8 | 65.574 | 0.09 | 0.31 | 0.20 | 0.16 | -0.99 | -0.56 | -0.78 | 0.31 | 0.98 |
| --- | --- | --- | --- | --- | --- | --- | --- | --- | --- | --- | --- |
| Q8CHH9 | 9-Sep | 49.812 | 1.31 | 1.20 | 1.26 | 0.07 | -0.07 | 0.14 | 0.04 | 0.15 | 1.22 |
| O55131 | 42254 | 50.549 | 0.86 | 0.66 | 0.76 | 0.15 | 0.07 | 0.03 | 0.05 | 0.03 | 0.71 |
| P42208 | 42249 | 41.525 | 0.72 | 0.48 | 0.60 | 0.17 | -0.25 | -0.17 | -0.21 | 0.05 | 0.81 |
| Q9CXL3 |  | 22.168 | 0.74 | 0.96 | 0.85 | 0.15 | -0.20 | 0.16 | -0.02 | 0.25 | 0.87 |
