# Supplementary material for: Hepatitis B virus X induces inflammation and cancer in mice liver through dysregulation of cytoskeletal remodeling and lipid metabolism
Source: Oncotarget. 2016 Sep 30;7(43):70559–74. doi: 10.18632/oncotarget.12372 (PMC5342574; doi:10.18632/oncotarget.12372)
Supplement: Supplementary file 6 [file oncotarget-07-70559-s006.docx]

**Table 6S. Bioinformatics analysis for changing protein in 12M *p21* HBx/+ samples compared with WT littermates.**

| **Category** | **Term** | **Count** | **PValue** | **Genes** |
| --- | --- | --- | --- | --- |
| GOTERM_BP_FAT | GO:0032989~cellular component  morphogenesis | 6 | 5.35E-05 | CDC42, LIPA, ACTA1, CFL1, RHOA, TTN |
| GOTERM_BP_FAT | GO:0030036~actin cytoskeleton organization | 4 | 1.05E-03 | ACTA1, CFL1, RHOA, TTN |
| GOTERM_BP_FAT | GO:0055114~oxidation reduction | 6 | 1.10E-03 | AKR1B7, SQRDL, CYP17A1, AKR1C18, DHTKD1,  RETSAT |
| GOTERM_BP_FAT | GO:0030029~actin filament-based process | 4 | 1.27E-03 | ACTA1, CFL1, RHOA, TTN |
| GOTERM_BP_FAT | GO:0034754~cellular hormone metabolic  process | 3 | 1.88E-03 | CYP17A1, AKR1C18, RETSAT |
| GOTERM_BP_FAT | GO:0007015~actin filament organization | 3 | 2.18E-03 | ACTA1, CFL1, RHOA |
| GOTERM_BP_FAT | GO:0042445~hormone metabolic process | 3 | 5.53E-03 | CYP17A1, AKR1C18, RETSAT |
| GOTERM_BP_FAT | GO:0000902~cell morphogenesis | 4 | 6.25E-03 | CDC42, LIPA, CFL1, RHOA |
| GOTERM_BP_FAT | GO:0007010~cytoskeleton organization | 4 | 7.25E-03 | ACTA1, CFL1, RHOA, TTN |
| GOTERM_BP_FAT | GO:0051130~positive regulation of cellular  component organization | 3 | 9.96E-03 | IGHG1, CFL1, RHOA |
| GOTERM_BP_FAT | GO:0014706~striated muscle tissue  development | 3 | 1.08E-02 | ACTA1, RHOA, TTN |

| GOTERM_BP_FAT | GO:0010817~regulation of hormone levels | 3 | 1.09E-02 | CYP17A1, AKR1C18, RETSAT |
| --- | --- | --- | --- | --- |
| GOTERM_BP_FAT | GO:0060537~muscle tissue development | 3 | 1.23E-02 | ACTA1, RHOA, TTN |
| GOTERM_BP_FAT | GO:0033043~regulation of organelle  organization | 3 | 1.55E-02 | CDC42, CFL1, RHOA |
| GOTERM_BP_FAT | GO:0007517~muscle organ development | 3 | 2.00E-02 | ACTA1, RHOA, TTN |
| GOTERM_BP_FAT | GO:0030239~myofibril assembly | 2 | 2.35E-02 | ACTA1, TTN |
| GOTERM_BP_FAT | GO:0031032~actomyosin structure  organization | 2 | 3.08E-02 | ACTA1, TTN |
| GOTERM_BP_FAT | GO:0051495~positive regulation of  cytoskeleton organization | 2 | 3.21E-02 | CFL1, RHOA |
| GOTERM_BP_FAT | GO:0007266~Rho protein signal transduction | 2 | 3.33E-02 | CDC42, RHOA |
| GOTERM_BP_FAT | GO:0007163~establishment or maintenance of  cell polarity | 2 | 3.81E-02 | CDC42, CFL1 |
| GOTERM_BP_FAT | GO:0007264~small GTPase mediated signal  transduction | 3 | 4.05E-02 | CDC42, RHOA, ITSN1 |
| GOTERM_BP_FAT | GO:0010927~cellular component assembly  involved in morphogenesis | 2 | 4.41E-02 | ACTA1, TTN |
| GOTERM_CC_FAT | GO:0005739~mitochondrion | 6 | 0.03 | AKR1B7, SQRDL, CYP17A1, HEBP1, RHOA,  DHTKD1 |
| GOTERM_CC_FAT | GO:0015629~actin cytoskeleton | 3 | 0.03 | ACTA1, CFL1, TTN |

| GOTERM_CC_FAT | GO:0005829~cytosol | 4 | 0.04 | AKR1C18, HEBP1, CFL1, RHOA |
| --- | --- | --- | --- | --- |
| GOTERM_MF_FAT | GO:0005089~Rho guanyl-nucleotide exchange  factor activity | 2 | 0.05 | TTN, ITSN1 |
| GOTERM_MF_FAT | GO:0005088~Ras guanyl-nucleotide exchange  factor activity | 2 | 0.05 | TTN, ITSN1 |
| KEGG_PATHWAY | mmu04360:Axon guidance | 3 | 0.02 | CDC42, CFL1, RHOA |
| KEGG_PATHWAY | mmu04510:Focal adhesion | 3 | 0.04 | CDC42, RHOA, COL6A1 |
| KEGG_PATHWAY | mmu04810:Regulation of actin cytoskeleton | 3 | 0.04 | CDC42, CFL1, RHOA |
| KEGG_PATHWAY | mmu00140:Steroid hormone biosynthesis | 2 | 0.07 | CYP17A1, AKR1C18 |
