# Supplementary material for: Hepatitis B virus X induces inflammation and cancer in mice liver through dysregulation of cytoskeletal remodeling and lipid metabolism
Source: Oncotarget. 2016 Sep 30;7(43):70559–74. doi: 10.18632/oncotarget.12372 (PMC5342574; doi:10.18632/oncotarget.12372)
Supplement: Supplementary file 7 [file oncotarget-07-70559-s007.docx]

**Table 7S. Bioinformatics analysis for changing protein in 24M *p21* HBx/+ samples compared with WT littermates.**

| **Category** | **Term** | **Count** | **PValue** | **Genes** |
| --- | --- | --- | --- | --- |
| GOTERM_BP_FAT | GO:0030029~actin filament-based process | 19 | 2.57E-10 | MYL6, DBNL, TLN1, ACTA1, CNN3, S100A9, CAPZA1, MYH9,  TTN, FLNA, CORO1C, PFN1, CORO1A, EZR, CFL1, MYH11,  FLII, CAP1, LCP1 |
| GOTERM_BP_FAT | GO:0030036~actin cytoskeleton  organization | 18 | 7.35E-10 | DBNL, TLN1, ACTA1, CNN3, S100A9, CAPZA1, MYH9, TTN,  FLNA, CORO1C, PFN1, CORO1A, EZR, CFL1, MYH11, FLII,  CAP1, LCP1 |
| GOTERM_BP_FAT | GO:0007010~cytoskeleton organization | 23 | 7.25E-09 | DBNL, TLN1, AGFG1, ACTA1, CNN3, S100A9, CAPZA1, MYH9,  TTN, FLNA, CORO1C, PFN1, CORO1A, YWHAH, EZR, TAGLN,  LASP1, CFL1, MYH11, TUBB5, FLII, CAP1, LCP1 |
| GOTERM_BP_FAT | GO:0055114~oxidation reduction | 33 | 1.37E-08 | XDH, ME1, TMX1, EHHADH, CYP2B9, G6PDX, PGD, LDHD,  ALDH3A2, PLOD1, P4HA1, HMOX1, FMO3, DHTKD1, NQO1,  NSDHL, SCD1, BCKDHA, CTBP1, PYROXD2, CYP3A11,  BCKDHB, MECP2, CMAH, DECR2, VAT1, CYP4A10, BLVRA,  HAO1, AKR1B7, CYP7B1, RRM1, HAO2 |
| GOTERM_BP_FAT | GO:0008064~regulation of actin  polymerization or depolymerization | 9 | 9.08E-07 | DBNL, ARPC1B, PFN1, CORO1A, ARPC2, CFL1, CAPZA1,  CAPG, ARPC5 |
| GOTERM_BP_FAT | GO:0030832~regulation of actin filament  length | 9 | 1.06E-06 | DBNL, ARPC1B, PFN1, CORO1A, ARPC2, CFL1, CAPZA1,  CAPG, ARPC5 |
| GOTERM_BP_FAT | GO:0032271~regulation of protein  polymerization | 9 | 2.55E-06 | DBNL, ARPC1B, PFN1, CORO1A, ARPC2, CAPZA1, CAPG,  MAPRE1, ARPC5 |
| GOTERM_BP_FAT | GO:0032956~regulation of actin  cytoskeleton organization | 9 | 3.79E-06 | DBNL, ARPC1B, PFN1, CORO1A, ARPC2, CFL1, CAPZA1,  CAPG, ARPC5 |
| GOTERM_BP_FAT | GO:0032970~regulation of actin filament-  based process | 9 | 4.30E-06 | DBNL, ARPC1B, PFN1, CORO1A, ARPC2, CFL1, CAPZA1,  CAPG, ARPC5 |
| GOTERM_BP_FAT | GO:0033043~regulation of organelle  organization | 13 | 4.98E-06 | CDC42, ARPC1B, DBNL, PFN1, CORO1A, YWHAH, ARPC2,  CFL1, CAPZA1, CAPG, MECP2, MAPRE1, ARPC5 |

| GOTERM_BP_FAT | GO:0030833~regulation of actin filament  polymerization | 8 | 5.42E-06 | DBNL, ARPC1B, PFN1, CORO1A, ARPC2, CAPZA1, CAPG,  ARPC5 |
| --- | --- | --- | --- | --- |
| GOTERM_BP_FAT | GO:0043254~regulation of protein complex  assembly | 9 | 6.99E-06 | DBNL, ARPC1B, PFN1, CORO1A, ARPC2, CAPZA1, CAPG,  MAPRE1, ARPC5 |
| GOTERM_BP_FAT | GO:0044087~regulation of cellular  component biogenesis | 10 | 9.61E-06 | DBNL, ARPC1B, PFN1, CORO1A, ARPC2, CAPZA1, CAPG,  MECP2, MAPRE1, ARPC5 |
| GOTERM_BP_FAT | GO:0046034~ATP metabolic process | 10 | 1.05E-05 | ATP6V0C, ATP6V1C1, ATP6V1A, AK1, ATP6V1E1, LDHD,  ATP6V1H, MYH7, ATP11C, ATP6V1B2 |
| GOTERM_BP_FAT | GO:0009150~purine ribonucleotide  metabolic process | 11 | 1.61E-05 | ATP6V0C, ATP6V1C1, ATP6V1A, AK1, ATP6V1E1, LDHD,  ATP6V1H, MYH7, ATP11C, ATP6V1B2, NT5E |
| GOTERM_BP_FAT | GO:0009165~nucleotide biosynthetic  process | 13 | 1.73E-05 | NAMPT, G6PDX, LDHD, ATP6V1H, ATP11C, ATP6V1B2,  CMPK2, ATP6V0C, ATP6V1C1, ATP6V1A, ATP6V1E1, RRM1,  NT5E |
| GOTERM_BP_FAT | GO:0034404~nucleobase, nucleoside and  nucleotide biosynthetic process | 13 | 2.28E-05 | NAMPT, G6PDX, LDHD, ATP6V1H, ATP11C, ATP6V1B2,  CMPK2, ATP6V0C, ATP6V1C1, ATP6V1A, ATP6V1E1, RRM1,  NT5E |
| GOTERM_BP_FAT | GO:0034654~nucleobase, nucleoside,  nucleotide and nucleic acid biosynthetic  process | 13 | 2.28E-05 | NAMPT, G6PDX, LDHD, ATP6V1H, ATP11C, ATP6V1B2,  CMPK2, ATP6V0C, ATP6V1C1, ATP6V1A, ATP6V1E1, RRM1,  NT5E |
| GOTERM_BP_FAT | GO:0051493~regulation of cytoskeleton  organization | 10 | 2.29E-05 | DBNL, ARPC1B, PFN1, CORO1A, ARPC2, CFL1, CAPZA1,  CAPG, MAPRE1, ARPC5 |
| GOTERM_BP_FAT | GO:0009259~ribonucleotide metabolic  process | 11 | 2.47E-05 | ATP6V0C, ATP6V1C1, ATP6V1A, AK1, ATP6V1E1, LDHD,  ATP6V1H, MYH7, ATP11C, ATP6V1B2, NT5E |
| GOTERM_BP_FAT | GO:0009205~purine ribonucleoside  triphosphate metabolic process | 10 | 2.69E-05 | ATP6V0C, ATP6V1C1, ATP6V1A, AK1, ATP6V1E1, LDHD,  ATP6V1H, MYH7, ATP11C, ATP6V1B2 |
| GOTERM_BP_FAT | GO:0009199~ribonucleoside triphosphate  metabolic process | 10 | 2.91E-05 | ATP6V0C, ATP6V1C1, ATP6V1A, AK1, ATP6V1E1, LDHD,  ATP6V1H, MYH7, ATP11C, ATP6V1B2 |
| GOTERM_BP_FAT | GO:0009144~purine nucleoside triphosphate  metabolic process | 10 | 3.95E-05 | ATP6V0C, ATP6V1C1, ATP6V1A, AK1, ATP6V1E1, LDHD,  ATP6V1H, MYH7, ATP11C, ATP6V1B2 |
| GOTERM_BP_FAT | GO:0009141~nucleoside triphosphate  metabolic process | 10 | 7.51E-05 | ATP6V0C, ATP6V1C1, ATP6V1A, AK1, ATP6V1E1, LDHD,  ATP6V1H, MYH7, ATP11C, ATP6V1B2 |

| GOTERM_BP_FAT | GO:0016192~vesicle-mediated transport | 20 | 1.21E-04 | ARFGAP1, DBNL, STX7, GNPDA1, IGH-3, AP3S1, FLNA,  CORO1C, CDC42, CD47, STX12, CD36, TFRC, CLEC4F, CAP1,  GGA1, EHD1, BIN1, SH3GL1, HIP1 |
| --- | --- | --- | --- | --- |
| GOTERM_BP_FAT | GO:0010324~membrane invagination | 12 | 1.71E-04 | CORO1C, DBNL, CD47, CD36, TFRC, CLEC4F, IGH-3, CAP1,  BIN1, EHD1, HIP1, SH3GL1 |
| GOTERM_BP_FAT | GO:0006897~endocytosis | 12 | 1.71E-04 | CORO1C, DBNL, CD47, CD36, TFRC, CLEC4F, IGH-3, CAP1,  BIN1, EHD1, HIP1, SH3GL1 |
| GOTERM_BP_FAT | GO:0005996~monosaccharide metabolic  process | 12 | 1.96E-04 | ALDOA, GNPDA1, GANC, ALDOC, PGD, G6PDX, CMAH,  NAGK, MAN2B1, DHTKD1, FABP5, PYGB |
| GOTERM_BP_FAT | GO:0006163~purine nucleotide metabolic  process | 11 | 1.97E-04 | ATP6V0C, ATP6V1C1, ATP6V1A, AK1, ATP6V1E1, LDHD,  ATP6V1H, MYH7, ATP11C, ATP6V1B2, NT5E |
| GOTERM_BP_FAT | GO:0006119~oxidative phosphorylation | 7 | 2.22E-04 | ATP6V0C, ATP6V1C1, ATP6V1A, ATP6V1E1, MECP2,  ATP6V1H, ATP6V1B2 |
| GOTERM_BP_FAT | GO:0007015~actin filament organization | 7 | 2.22E-04 | DBNL, CORO1A, EZR, ACTA1, CFL1, LCP1, FLNA |
| GOTERM_BP_FAT | GO:0006091~generation of precursor  metabolites and energy | 14 | 2.23E-04 | ALDOA, TMX1, GNPDA1, ALDOC, CMAH, MECP2, ATP6V1H,  ATP6V1B2, ATP6V0C, ATP6V1C1, ATP6V1A, ATP6V1E1,  DHTKD1, PYGB |
| GOTERM_BP_FAT | GO:0015986~ATP synthesis coupled proton  transport | 6 | 2.50E-04 | ATP6V0C, ATP6V1C1, ATP6V1A, ATP6V1E1, ATP6V1H,  ATP6V1B2 |
| GOTERM_BP_FAT | GO:0015985~energy coupled proton  transport, down electrochemical gradient | 6 | 2.50E-04 | ATP6V0C, ATP6V1C1, ATP6V1A, ATP6V1E1, ATP6V1H,  ATP6V1B2 |
| GOTERM_BP_FAT | GO:0006754~ATP biosynthetic process | 8 | 2.80E-04 | ATP6V0C, ATP6V1C1, ATP6V1A, ATP6V1E1, LDHD,  ATP6V1H, ATP11C, ATP6V1B2 |
| GOTERM_BP_FAT | GO:0034220~ion transmembrane transport | 6 | 4.59E-04 | ATP6V0C, ATP6V1C1, ATP6V1A, ATP6V1E1, ATP6V1H,  ATP6V1B2 |
| GOTERM_BP_FAT | GO:0051129~negative regulation of cellular  component organization | 8 | 6.04E-04 | RTN4, DBNL, PFN1, YWHAH, CAPZA1, CAPG, MECP2,  MAPRE1 |
| GOTERM_BP_FAT | GO:0009201~ribonucleoside triphosphate  biosynthetic process | 8 | 6.04E-04 | ATP6V0C, ATP6V1C1, ATP6V1A, ATP6V1E1, LDHD,  ATP6V1H, ATP11C, ATP6V1B2 |
| GOTERM_BP_FAT | GO:0009206~purine ribonucleoside  triphosphate biosynthetic process | 8 | 6.04E-04 | ATP6V0C, ATP6V1C1, ATP6V1A, ATP6V1E1, LDHD,  ATP6V1H, ATP11C, ATP6V1B2 |

| GOTERM_BP_FAT | GO:0009145~purine nucleoside triphosphate  biosynthetic process | 8 | 6.44E-04 | ATP6V0C, ATP6V1C1, ATP6V1A, ATP6V1E1, LDHD,  ATP6V1H, ATP11C, ATP6V1B2 |
| --- | --- | --- | --- | --- |
| GOTERM_BP_FAT | GO:0009142~nucleoside triphosphate  biosynthetic process | 8 | 6.86E-04 | ATP6V0C, ATP6V1C1, ATP6V1A, ATP6V1E1, LDHD,  ATP6V1H, ATP11C, ATP6V1B2 |
| GOTERM_BP_FAT | GO:0031333~negative regulation of protein  complex assembly | 5 | 7.49E-04 | DBNL, PFN1, CAPZA1, CAPG, MAPRE1 |
| GOTERM_BP_FAT | GO:0032272~negative regulation of protein  polymerization | 5 | 7.49E-04 | DBNL, PFN1, CAPZA1, CAPG, MAPRE1 |
| GOTERM_BP_FAT | GO:0034613~cellular protein localization | 14 | 8.11E-04 | STX8, STX7, AKAP12, SNX2, SNX1, TOMM40, AP3S1, FLNA,  AIP, CDC42, YWHAH, STX12, SH3GLB1, GGA1 |
| GOTERM_BP_FAT | GO:0070727~cellular macromolecule  localization | 14 | 8.61E-04 | STX8, STX7, AKAP12, SNX2, SNX1, TOMM40, AP3S1, FLNA,  AIP, CDC42, YWHAH, STX12, SH3GLB1, GGA1 |
| GOTERM_BP_FAT | GO:0044271~nitrogen compound  biosynthetic process | 14 | 8.88E-04 | NAMPT, MAT2A, G6PDX, LDHD, ATP6V1H, ATP11C,  ATP6V1B2, CMPK2, ATP6V0C, ATP6V1C1, ATP6V1A,  ATP6V1E1, RRM1, NT5E |
| GOTERM_BP_FAT | GO:0032535~regulation of cellular  component size | 10 | 9.21E-04 | RTN4, DBNL, ARPC1B, PFN1, CORO1A, ARPC2, CFL1,  CAPZA1, CAPG, ARPC5 |
| GOTERM_BP_FAT | GO:0016044~membrane organization | 13 | 1.13E-03 | CORO1C, CD47, DBNL, CD36, TFRC, SH3GLB1, CLEC4F, IGH-  3, CAP1, EHD1, BIN1, SH3GL1, HIP1 |
| GOTERM_BP_FAT | GO:0046907~intracellular transport | 17 | 1.15E-03 | MYL6, STX8, STX7, AKAP12, SNX2, SNX1, TOMM40, AP3S1,  MYH9, HNRNPA1, FLNA, AIP, CDC42, YWHAH, STX12, GGA1,  EHD1 |
| GOTERM_BP_FAT | GO:0015992~proton transport | 6 | 1.24E-03 | ATP6V0C, ATP6V1C1, ATP6V1A, ATP6V1E1, ATP6V1H,  ATP6V1B2 |
| GOTERM_BP_FAT | GO:0019318~hexose metabolic process | 10 | 1.30E-03 | ALDOA, GNPDA1, GANC, ALDOC, PGD, G6PDX, MAN2B1,  DHTKD1, FABP5, PYGB |
| GOTERM_BP_FAT | GO:0006164~purine nucleotide biosynthetic  process | 9 | 1.34E-03 | ATP6V0C, ATP6V1C1, ATP6V1A, ATP6V1E1, LDHD,  ATP6V1H, ATP11C, ATP6V1B2, NT5E |
| GOTERM_BP_FAT | GO:0006818~hydrogen transport | 6 | 1.35E-03 | ATP6V0C, ATP6V1C1, ATP6V1A, ATP6V1E1, ATP6V1H,  ATP6V1B2 |
| GOTERM_BP_FAT | GO:0009152~purine ribonucleotide  biosynthetic process | 8 | 1.38E-03 | ATP6V0C, ATP6V1C1, ATP6V1A, ATP6V1E1, LDHD,  ATP6V1H, ATP11C, ATP6V1B2 |

| GOTERM_BP_FAT | GO:0008104~protein localization | 24 | 1.48E-03 | ARFGAP1, TLN1, STX8, STX7, SNX6, SNX5, AKAP12, HSPG2,  MECP2, SNX2, SNX1, TOMM40, AP3S1, MYH9, FLNA, AIP,  CDC42, RAB32, PFN1, YWHAH, STX12, CD36, SH3GLB1, |
| --- | --- | --- | --- | --- |
| GOTERM_BP_FAT | GO:0009260~ribonucleotide biosynthetic  process | 8 | 1.71E-03 | GGA1  ATP6V0C, ATP6V1C1, ATP6V1A, ATP6V1E1, LDHD,  ATP6V1H, ATP11C, ATP6V1B2 |
| GOTERM_BP_FAT | GO:0007517~muscle organ development | 10 | 1.72E-03 | MYL6, DES, TAGLN, ACTA1, HIST1H1B, MYH11, HSPG2,  CXADR, TTN, TAGLN2 |
| GOTERM_BP_FAT | GO:0051186~cofactor metabolic process | 10 | 2.16E-03 | BLVRA, NAMPT, HMOX1, EHHADH, PGD, HEBP1, G6PDX,  ACOT2, GSTP1, ACOT3 |
| GOTERM_BP_FAT | GO:0010639~negative regulation of  organelle organization | 6 | 2.72E-03 | DBNL, PFN1, CAPZA1, CAPG, MECP2, MAPRE1 |
| GOTERM_BP_FAT | GO:0008202~steroid metabolic process | 9 | 3.65E-03 | CYP7B1, YWHAH, SULT2A1, SULT1A1, FDPS, HMGCS1,  MECP2, IDI1, NSDHL |
| GOTERM_BP_FAT | GO:0022604~regulation of cell | 7 | 4.00E-03 | RTN4, CORO1A, YWHAH, EZR, PALMD, MYH9, FN1 |
| GOTERM_BP_FAT | GO:0006695~cholesterol biosynthetic | 4 | 5.20E-03 | FDPS, HMGCS1, IDI1, NSDHL |
| GOTERM_BP_FAT | GO:0046700~heterocycle catabolic process | 5 | 5.54E-03 | BLVRA, HMOX1, MYH7, NT5E, AFMID |
| GOTERM_BP_FAT | GO:0030834~regulation of actin filament  depolymerization | 4 | 5.87E-03 | DBNL, CFL1, CAPZA1, CAPG |
| GOTERM_BP_FAT | GO:0008360~regulation of cell shape | 5 | 5.98E-03 | CORO1A, EZR, PALMD, MYH9, FN1 |
| GOTERM_BP_FAT | GO:0006006~glucose metabolic process | 8 | 6.22E-03 | ALDOA, GANC, ALDOC, PGD, G6PDX, DHTKD1, FABP5, |
| GOTERM_BP_FAT | GO:0051494~negative regulation of  cytoskeleton organization | 5 | 6.45E-03 | DBNL, PFN1, CAPZA1, CAPG, MAPRE1 |
| GOTERM_BP_FAT | GO:0006575~cellular amino acid derivative  metabolic process | 8 | 6.46E-03 | MAT2A, P4HA1, G6PDX, MECP2, TPMT, FABP5, GSTP1,  AFMID |
| GOTERM_BP_FAT | GO:0030837~negative regulation of actin  filament polymerization | 4 | 6.60E-03 | DBNL, PFN1, CAPZA1, CAPG |
| GOTERM_BP_FAT | GO:0031032~actomyosin structure | 4 | 6.60E-03 | CNN3, ACTA1, MYH11, TTN |
| GOTERM_BP_FAT | GO:0065003~macromolecular complex  assembly | 13 | 6.63E-03 | HIST1H1D, HIST1H1C, HIST1H1B, HIST1H1A, NAP1L1,  ANXA5, FLNA, PTRF, RRM1, MYH11, TGM2, TUBB5, HIP1 |
| GOTERM_BP_FAT | GO:0009719~response to endogenous  stimulus | 9 | 8.05E-03 | BCKDHA, ME1, LNPEP, HMGB2, MAT2A, LYN, HMOX1,  CFL1, SERPINA1E |
| GOTERM_BP_FAT | GO:0030100~regulation of endocytosis | 5 | 8.55E-03 | CD47, IGH-3, BIN1, CD2AP, HIP1 |

| GOTERM_BP_FAT | GO:0060627~regulation of vesicle-mediated  transport | 6 | 9.39E-03 | CD47, HMOX1, IGH-3, BIN1, CD2AP, HIP1 |
| --- | --- | --- | --- | --- |
| GOTERM_BP_FAT | GO:0046365~monosaccharide catabolic | 5 | 9.75E-03 | ALDOA, GNPDA1, ALDOC, PGD, DHTKD1 |
| GOTERM_BP_FAT | GO:0042692~muscle cell differentiation | 7 | 9.81E-03 | ACTA1, MYH11, MYH9, CXADR, TTN, CAPN2, BIN1 |
| GOTERM_BP_FAT | GO:0016126~sterol biosynthetic process | 4 | 1.10E-02 | FDPS, HMGCS1, IDI1, NSDHL |
| GOTERM_BP_FAT | GO:0006886~intracellular protein transport | 11 | 1.11E-02 | STX8, YWHAH, STX12, STX7, AKAP12, SNX2, TOMM40,  SNX1, AP3S1, GGA1, AIP |
| GOTERM_BP_FAT | GO:0007163~establishment or maintenance  of cell polarity | 4 | 1.20E-02 | CDC42, EZR, CFL1, MYH9 |
| GOTERM_BP_FAT | GO:0043933~macromolecular complex  subunit organization | 13 | 1.23E-02 | HIST1H1D, HIST1H1C, HIST1H1B, HIST1H1A, NAP1L1,  ANXA5, FLNA, PTRF, RRM1, MYH11, TGM2, TUBB5, HIP1 |
| GOTERM_BP_FAT | GO:0051146~striated muscle cell | 6 | 1.25E-02 | ACTA1, MYH11, MYH9, CXADR, TTN, CAPN2 |
| GOTERM_BP_FAT | GO:0034330~cell junction organization | 4 | 1.31E-02 | CDC42, TLN1, CXADR, FN1 |
| GOTERM_BP_FAT | GO:0044275~cellular carbohydrate catabolic  process | 5 | 1.40E-02 | ALDOA, GNPDA1, ALDOC, PGD, DHTKD1 |
| GOTERM_BP_FAT | GO:0007155~cell adhesion | 17 | 1.42E-02 | PTPRC, TLN1, LPP, HSPG2, MYH9, CXADR, CD2AP, CDC42,  CD47, LGALS3BP, CD36, COL14A1, TGFBI, COL6A1, ZYX,  THBS1, FN1 |
| GOTERM_BP_FAT | GO:0031532~actin cytoskeleton | 3 | 1.43E-02 | S100A9, MYH9, FLNA |
| GOTERM_BP_FAT | GO:0022610~biological adhesion | 17 | 1.44E-02 | PTPRC, TLN1, LPP, HSPG2, MYH9, CXADR, CD2AP, CDC42,  CD47, LGALS3BP, CD36, COL14A1, TGFBI, COL6A1, ZYX,  THBS1, FN1 |
| GOTERM_BP_FAT | GO:0034621~cellular macromolecular  complex subunit organization | 10 | 1.45E-02 | HIST1H1D, HIST1H1C, PTRF, HIST1H1B, HIST1H1A, MYH11,  NAP1L1, TUBB5, FLNA, HIP1 |
| GOTERM_BP_FAT | GO:0009725~response to hormone stimulus | 8 | 1.46E-02 | BCKDHA, ME1, LNPEP, HMGB2, MAT2A, LYN, HMOX1,  SERPINA1E |
| GOTERM_BP_FAT | GO:0030865~cortical cytoskeleton | 3 | 1.67E-02 | CORO1C, TLN1, LASP1 |
| GOTERM_BP_FAT | GO:0010927~cellular component assembly  involved in morphogenesis | 4 | 1.81E-02 | AGFG1, ACTA1, MYH11, TTN |
| GOTERM_BP_FAT | GO:0046164~alcohol catabolic process | 5 | 1.83E-02 | ALDOA, GNPDA1, ALDOC, PGD, DHTKD1 |
| GOTERM_BP_FAT | GO:0050853~B cell receptor signaling | 3 | 1.93E-02 | PTPN6, PTPRC, LYN |
| GOTERM_BP_FAT | GO:0042440~pigment metabolic process | 4 | 1.95E-02 | BLVRA, HMOX1, HEBP1, IDI1 |

| GOTERM_BP_FAT | GO:0034622~cellular macromolecular  complex assembly | 9 | 2.02E-02 | HIST1H1D, HIST1H1C, HIST1H1B, HIST1H1A, MYH11,  NAP1L1, TUBB5, FLNA, HIP1 |
| --- | --- | --- | --- | --- |
| GOTERM_BP_FAT | GO:0030198~extracellular matrix | 6 | 2.05E-02 | P4HA1, TGFBI, MYH11, HSPG2, SERPINH1, ANXA2 |
| GOTERM_BP_FAT | GO:0051017~actin filament bundle | 3 | 2.21E-02 | DBNL, EZR, LCP1 |
| GOTERM_BP_FAT | GO:0032101~regulation of response to  external stimulus | 6 | 2.21E-02 | CD47, TGM2, IGH-3, THBS1, NT5E, ANXA2 |
| GOTERM_BP_FAT | GO:0019748~secondary metabolic process | 5 | 2.23E-02 | NAMPT, RBP1, PGD, G6PDX, IDI1 |
| GOTERM_BP_FAT | GO:0032103~positive regulation of response  to external stimulus | 4 | 2.24E-02 | CD47, TGM2, IGH-3, THBS1 |
| GOTERM_BP_FAT | GO:0008203~cholesterol metabolic process | 5 | 2.34E-02 | CYP7B1, FDPS, HMGCS1, IDI1, NSDHL |
| GOTERM_BP_FAT | GO:0006732~coenzyme metabolic process | 7 | 2.41E-02 | NAMPT, EHHADH, PGD, G6PDX, ACOT2, GSTP1, ACOT3 |
| GOTERM_BP_FAT | GO:0006694~steroid biosynthetic process | 5 | 2.45E-02 | CYP7B1, FDPS, HMGCS1, IDI1, NSDHL |
| GOTERM_BP_FAT | GO:0006631~fatty acid metabolic process | 8 | 2.48E-02 | CYP4A10, SCD1, ACSM1, EHHADH, HAO2, ACACA, ACSL4,  ACOT3 |
| GOTERM_BP_FAT | GO:0006334~nucleosome assembly | 5 | 2.68E-02 | HIST1H1D, HIST1H1C, HIST1H1B, HIST1H1A, NAP1L1 |
| GOTERM_BP_FAT | GO:0045184~establishment of protein  localization | 18 | 2.69E-02 | ARFGAP1, STX8, STX7, SNX6, SNX5, AKAP12, SNX2, SNX1,  TOMM40, AP3S1, MYH9, FLNA, AIP, RAB32, YWHAH, STX12,  CD36, GGA1 |
| GOTERM_BP_FAT | GO:0044270~nitrogen compound catabolic  process | 4 | 2.72E-02 | BLVRA, HMOX1, MYH7, NT5E |
| GOTERM_BP_FAT | GO:0043062~extracellular structure | 7 | 2.88E-02 | P4HA1, TGFBI, MYH11, MECP2, HSPG2, SERPINH1, ANXA2 |
| GOTERM_BP_FAT | GO:0043244~regulation of protein complex  disassembly | 4 | 2.89E-02 | DBNL, CFL1, CAPZA1, CAPG |
| GOTERM_BP_FAT | GO:0031497~chromatin assembly | 5 | 2.92E-02 | HIST1H1D, HIST1H1C, HIST1H1B, HIST1H1A, NAP1L1 |
| GOTERM_BP_FAT | GO:0034728~nucleosome organization | 5 | 3.05E-02 | HIST1H1D, HIST1H1C, HIST1H1B, HIST1H1A, NAP1L1 |
| GOTERM_BP_FAT | GO:0065004~protein-DNA complex | 5 | 3.05E-02 | HIST1H1D, HIST1H1C, HIST1H1B, HIST1H1A, NAP1L1 |
| GOTERM_BP_FAT | GO:0042167~heme catabolic process | 2 | 3.08E-02 | BLVRA, HMOX1 |
| GOTERM_BP_FAT | GO:0046149~pigment catabolic process | 2 | 3.08E-02 | BLVRA, HMOX1 |
| GOTERM_BP_FAT | GO:0019322~pentose biosynthetic process | 2 | 3.08E-02 | PGD, G6PDX |
| GOTERM_BP_FAT | GO:0032796~uropod organization | 2 | 3.08E-02 | CORO1A, MYH9 |
| GOTERM_BP_FAT | GO:0016125~sterol metabolic process | 5 | 3.17E-02 | CYP7B1, FDPS, HMGCS1, IDI1, NSDHL |

| GOTERM_BP_FAT | GO:0008610~lipid biosynthetic process | 10 | 3.41E-02 | SCD1, CYP7B1, ACSM1, SH3GLB1, FDPS, HMGCS1, ACACA,  IDI1, FABP5, NSDHL |
| --- | --- | --- | --- | --- |
| GOTERM_BP_FAT | GO:0051693~actin filament capping | 3 | 3.45E-02 | DBNL, CAPZA1, CAPG |
| GOTERM_BP_FAT | GO:0042168~heme metabolic process | 3 | 3.45E-02 | BLVRA, HMOX1, HEBP1 |
| GOTERM_BP_FAT | GO:0006040~amino sugar metabolic process | 3 | 3.45E-02 | GNPDA1, CMAH, NAGK |
| GOTERM_BP_FAT | GO:0030239~myofibril assembly | 3 | 3.45E-02 | ACTA1, MYH11, TTN |
| GOTERM_BP_FAT | GO:0048514~blood vessel morphogenesis | 8 | 3.49E-02 | RTN4, PTPRJ, SGPL1, HMOX1, CASP8, TGM2, MYH9, ANXA2 |
| GOTERM_BP_FAT | GO:0001568~blood vessel development | 9 | 3.69E-02 | RTN4, PTPRJ, SGPL1, HMOX1, CASP8, TGM2, MYH9, NSDHL,  ANXA2 |
| GOTERM_BP_FAT | GO:0016052~carbohydrate catabolic process | 5 | 3.72E-02 | ALDOA, GNPDA1, ALDOC, PGD, DHTKD1 |
| GOTERM_BP_FAT | GO:0006637~acyl-CoA metabolic process | 3 | 3.80E-02 | EHHADH, ACOT2, ACOT3 |
| GOTERM_BP_FAT | GO:0002698~negative regulation of immune  effector process | 3 | 3.80E-02 | PTPN6, PTPRC, HMOX1 |
| GOTERM_BP_FAT | GO:0006720~isoprenoid metabolic process | 4 | 4.03E-02 | RBP1, FDPS, HMGCS1, IDI1 |
| GOTERM_BP_FAT | GO:0006909~phagocytosis | 4 | 4.03E-02 | CORO1C, CD47, CD36, IGH-3 |
| GOTERM_BP_FAT | GO:0030835~negative regulation of actin  filament depolymerization | 3 | 4.15E-02 | DBNL, CAPZA1, CAPG |
| GOTERM_BP_FAT | GO:0030199~collagen fibril organization | 3 | 4.15E-02 | P4HA1, SERPINH1, ANXA2 |
| GOTERM_BP_FAT | GO:0051130~positive regulation of cellular  component organization | 6 | 4.15E-02 | CD47, ARPC2, CFL1, MECP2, IGH-3, HIP1 |
| GOTERM_BP_FAT | GO:0001944~vasculature development | 9 | 4.17E-02 | RTN4, PTPRJ, SGPL1, HMOX1, CASP8, TGM2, MYH9, NSDHL,  ANXA2 |
| GOTERM_BP_FAT | GO:0008299~isoprenoid biosynthetic | 3 | 4.52E-02 | FDPS, HMGCS1, IDI1 |
| GOTERM_BP_FAT | GO:0051014~actin filament severing | 2 | 4.59E-02 | DBNL, FLII |
| GOTERM_BP_FAT | GO:0033015~tetrapyrrole catabolic process | 2 | 4.59E-02 | BLVRA, HMOX1 |
| GOTERM_BP_FAT | GO:0016584~nucleosome positioning | 2 | 4.59E-02 | HIST1H1D, HIST1H1C |
| GOTERM_BP_FAT | GO:0006787~porphyrin catabolic process | 2 | 4.59E-02 | BLVRA, HMOX1 |
| GOTERM_BP_FAT | GO:0006979~response to oxidative stress | 5 | 4.64E-02 | HMOX1, G6PDX, CYGB, MYH7, NQO1 |
| GOTERM_BP_FAT | GO:0002252~immune effector process | 6 | 4.66E-02 | PTPN6, PTPRC, CD47, SWAP70, IGH-3, SAMHD1 |
| GOTERM_BP_FAT | GO:0006007~glucose catabolic process | 4 | 4.68E-02 | ALDOA, ALDOC, PGD, DHTKD1 |
| GOTERM_BP_FAT | GO:0019320~hexose catabolic process | 4 | 4.68E-02 | ALDOA, ALDOC, PGD, DHTKD1 |

| GOTERM_BP_FAT | GO:0015031~protein transport | 17 | 4.69E-02 | ARFGAP1, STX8, STX7, SNX6, SNX5, AKAP12, SNX2, SNX1,  TOMM40, AP3S1, MYH9, AIP, RAB32, YWHAH, CD36, STX12,  GGA1 |
| --- | --- | --- | --- | --- |
| GOTERM_BP_FAT | GO:0014706~striated muscle tissue | 6 | 4.80E-02 | MYL6, ACTA1, MYH11, HSPG2, CXADR, TTN |
| GOTERM_BP_FAT | GO:0032312~regulation of ARF GTPase | 3 | 4.90E-02 | ARFGAP1, SMAP2, AGFG1 |
| GOTERM_CC_FAT | GO:0015629~actin cytoskeleton | 22 | 1.12E-11 | MYL6, TLN1, TWF1, ACTA1, CAPZA1, MYH7, ARPC5, MYH9,  TTN, TPM4, ANXA2, ARPC1B, PFN1, CORO1A, EZR, ARPC2,  LASP1, CFL1, MYH11, CAP1, WDR1, LCP1 |
| GOTERM_CC_FAT | GO:0005938~cell cortex | 13 | 7.55E-07 | DBNL, CORO1A, EZR, LASP1, CFL1, CAPZA1, MARCKS,  MAPRE1, CAP1, MYH9, CD2AP, TPM4, FLNA |
| GOTERM_CC_FAT | GO:0031252~cell leading edge | 12 | 1.63E-06 | DBNL, CORO1A, TLN1, ARPC2, SWAP70, VIM, ARHGAP1,  ARPC5, MYH9, CD2AP, IQGAP1, LCP1 |
| GOTERM_CC_FAT | GO:0005856~cytoskeleton | 40 | 2.30E-06 | MYL6, DYNC1LI1, TLN1, VIM, CAPZA1, AKAP12, PDLIM1,  ARPC5, TTN, CD2AP, TPM4, KEG1, PFN1, DES, EZR, ARPC2,  ACTR1A, TUBB5, MSN, ZYX, CAP1, DBNL, TWF1, ACTA1,  MYH7, MYH9, COTL1, FLNA, ANXA2, CORO1C, ARPC1B,  CORO1A, LASP1, CFL1, MYH11, FLII, MARCKS, WDR1,  MAPRE1, LCP1 |
| GOTERM_CC_FAT | GO:0005829~cytosol | 25 | 6.75E-06 | ME1, XDH, CNDP2, ATP6V1B2, ACOT3, AFMID, PFN1,  SH3GLB1, BAG3, HMOX1, TUBB5, TGM2, RRAS, CAP1,  CTBP1, MECP2, CLIC1, TPMT, MYH9, ATP6V1A, PTRF,  ATP6V1E1, HEBP1, CFL1, LCP1 |
| GOTERM_CC_FAT | GO:0043232~intracellular non-membrane-  bounded organelle | 53 | 5.24E-05 | DYNC1LI1, TLN1, CAPZA1, PDLIM1, TTN, CD2AP, DES,  HMOX1, ACTR1A, CASP8, TUBB5, ZYX, CAP1, MSN, DBNL,  TWF1, HIST1H1D, HIST1H1C, ACTA1, HIST1H1B, HIST1H1A,  MECP2, MYH7, MYH9, FLNA, RSL1D1, ARPC1B, CFL1, FLII,  MAPRE1, LCP1, MYL6, HMGB2, MTDH, VIM, AKAP12,  ARPC5, TPM4, KEG1, PFN1, EZR, ARPC2, SMCHD1, COTL1,  ANXA2, CORO1C, CORO1A, LASP1, MYH11, MARCKS,  WDR1, TMPO, PES1 |

| GOTERM_CC_FAT | GO:0043228~non-membrane-bounded  organelle | 53 | 5.24E-05 | DYNC1LI1, TLN1, CAPZA1, PDLIM1, TTN, CD2AP, DES,  HMOX1, ACTR1A, CASP8, TUBB5, ZYX, CAP1, MSN, DBNL,  TWF1, HIST1H1D, HIST1H1C, ACTA1, HIST1H1B, HIST1H1A,  MECP2, MYH7, MYH9, FLNA, RSL1D1, ARPC1B, CFL1, FLII,  MAPRE1, LCP1, MYL6, HMGB2, MTDH, VIM, AKAP12,  ARPC5, TPM4, KEG1, PFN1, EZR, ARPC2, SMCHD1, COTL1,  ANXA2, CORO1C, CORO1A, LASP1, MYH11, MARCKS,  WDR1, TMPO, PES1 |
| --- | --- | --- | --- | --- |
| GOTERM_CC_FAT | GO:0030863~cortical cytoskeleton | 7 | 5.62E-05 | LASP1, CFL1, CAPZA1, MAPRE1, CAP1, MYH9, TPM4 |
| GOTERM_CC_FAT | GO:0033178~proton-transporting two-sector  ATPase complex, catalytic domain | 5 | 1.26E-04 | ATP6V1C1, ATP6V1A, ATP6V1E1, ATP6V1H, ATP6V1B2 |
| GOTERM_CC_FAT | GO:0033176~proton-transporting V-type  ATPase complex | 5 | 1.60E-04 | ATP6V0C, ATP6V1C1, ATP6V1A, ATP6V1H, ATP6V1B2 |
| GOTERM_CC_FAT | GO:0033180~proton-transporting V-type  ATPase, V1 domain | 4 | 2.10E-04 | ATP6V1C1, ATP6V1A, ATP6V1H, ATP6V1B2 |
| GOTERM_CC_FAT | GO:0005777~peroxisome | 9 | 2.50E-04 | XDH, HAO1, EHHADH, HAO2, ABCD2, DECR2, ACSL4, IDI1,  ACOT3 |
| GOTERM_CC_FAT | GO:0042579~microbody | 9 | 2.50E-04 | XDH, HAO1, EHHADH, HAO2, ABCD2, DECR2, ACSL4, IDI1,  ACOT3 |
| GOTERM_CC_FAT | GO:0048770~pigment granule | 8 | 4.02E-04 | LAMP1, TFRC, CAPG, MYH11, NAP1L1, SLC3A2, ATP6V1B2,  ANXA2 |
| GOTERM_CC_FAT | GO:0042470~melanosome | 8 | 4.02E-04 | LAMP1, TFRC, CAPG, MYH11, NAP1L1, SLC3A2, ATP6V1B2,  ANXA2 |
| GOTERM_CC_FAT | GO:0005792~microsome | 11 | 5.11E-04 | CYP4A10, LNPEP, CYP7B1, PTRF, SH3GLB1, CYP2B9,  HMOX1, CYP3A11, FMO3, ACSL4, TAPBP |
| GOTERM_CC_FAT | GO:0016469~proton-transporting two-sector  ATPase complex | 6 | 5.12E-04 | ATP6V0C, ATP6V1C1, ATP6V1A, ATP6V1E1, ATP6V1H,  ATP6V1B2 |
| GOTERM_CC_FAT | GO:0044430~cytoskeletal part | 26 | 5.70E-04 | MYL6, DYNC1LI1, VIM, CAPZA1, ARPC5, TTN, KEG1, TPM4,  DES, EZR, ARPC2, ACTR1A, TUBB5, CAP1, ACTA1, MYH7,  MYH9, ANXA2, ARPC1B, LASP1, CFL1, MYH11, FLII,  MARCKS, MAPRE1, LCP1 |

| GOTERM_CC_FAT | GO:0042598~vesicular fraction | 11 | 6.64E-04 | CYP4A10, LNPEP, CYP7B1, PTRF, SH3GLB1, CYP2B9,  HMOX1, CYP3A11, FMO3, ACSL4, TAPBP |
| --- | --- | --- | --- | --- |
| GOTERM_CC_FAT | GO:0042995~cell projection | 21 | 8.50E-04 | RTN4, ALDOA, DBNL, TLN1, AGFG1, SWAP70, ANXA1,  ARPC5, DPYSL2, MYH9, CD2AP, IQGAP1, CDC42, EZR,  ARPC2, ARHGAP1, CYGB, MAPRE1, MSN, LCP1, PYGB |
| GOTERM_CC_FAT | GO:0001726~ruffle | 6 | 8.66E-04 | DBNL, TLN1, ARHGAP1, MYH9, CD2AP, LCP1 |
| GOTERM_CC_FAT | GO:0042383~sarcolemma | 6 | 8.66E-04 | LAMP1, DES, BGN, ANXA1, COL6A1, ANXA2 |
| GOTERM_CC_FAT | GO:0005739~mitochondrion | 37 | 8.86E-04 | ME1, MCL1, EHHADH, ALDOC, LDHD, ACOT2, ACSS3,  ALDH3A2, KEG1, CMPK2, CKB, ACOT9, SH3GLB1, CASP8,  TGM2, ABCD2, DHTKD1, ACSL4, BCKDHA, SLC25A4, AK1,  BCKDHB, FDPS, ACACA, TOMM40, DPYSL2, ATP6V1A,  AKR1B7, RAB32, ACSM1, GLYAT, PTRF, ATP6V1E1, HEBP1,  HAO2, IDI1, MPST |
| GOTERM_CC_FAT | GO:0044448~cell cortex part | 7 | 1.06E-03 | LASP1, CFL1, CAPZA1, MAPRE1, CAP1, MYH9, TPM4 |
| GOTERM_CC_FAT | GO:0000267~cell fraction | 21 | 1.31E-03 | ME1, CYP2B9, CYP3A11, CLIC1, NAGK, ANXA2, TAPBP, AIP,  LNPEP, BLVRA, CYP4A10, CYP7B1, DES, GNAQ, PTRF,  SH3GLB1, HMOX1, IGF2R, FMO3, ACSL4, NT5E |
| GOTERM_CC_FAT | GO:0005768~endosome | 12 | 2.35E-03 | LAMP1, STX7, TFRC, IGF2R, LGMN, ATP6V1E1, SNX1, GGA1,  EHD1, SH3GL1, ANXA2, EHD4 |
| GOTERM_CC_FAT | GO:0031254~trailing edge | 3 | 2.44E-03 | EZR, MSN, MYH9 |
| GOTERM_CC_FAT | GO:0001931~uropod | 3 | 2.44E-03 | EZR, MSN, MYH9 |
| GOTERM_CC_FAT | GO:0005885~Arp2/3 protein complex | 3 | 3.62E-03 | ARPC1B, ARPC2, ARPC5 |
| GOTERM_CC_FAT | GO:0005626~insoluble fraction | 18 | 4.58E-03 | CYP2B9, CYP3A11, CLIC1, ANXA2, AIP, TAPBP, LNPEP,  CYP4A10, CYP7B1, DES, GNAQ, PTRF, SH3GLB1, HMOX1,  IGF2R, FMO3, ACSL4, NT5E |
| GOTERM_CC_FAT | GO:0001725~stress fiber | 4 | 5.56E-03 | ACTA1, MYH11, MYH9, ANXA2 |
| GOTERM_CC_FAT | GO:0016023~cytoplasmic membrane-  bounded vesicle | 15 | 6.48E-03 | ALDOA, AGFG1, NAP1L1, SLC3A2, ATP6V1B2, CD2AP,  ANXA2, LAMP1, CORO1A, TFRC, IGF2R, CAPG, MYH11, |
| GOTERM_CC_FAT | GO:0005912~adherens junction | 7 | 6.89E-03 | EHD1, HIP1PTPRC, TLN1, LASP1, ARPC2, ZYX, MYH9, CXADR |
| GOTERM_CC_FAT | GO:0031988~membrane-bounded vesicle | 15 | 7.31E-03 | ALDOA, AGFG1, NAP1L1, SLC3A2, ATP6V1B2, CD2AP, ANXA2, LAMP1, CORO1A, TFRC, IGF2R, CAPG, MYH11, |

EHD1, HIP1

| GOTERM_CC_FAT | GO:0005624~membrane fraction | 17 | 7.49E-03 | CYP2B9, CYP3A11, CLIC1, AIP, TAPBP, ANXA2, LNPEP,  CYP4A10, CYP7B1, GNAQ, PTRF, SH3GLB1, HMOX1, IGF2R,  FMO3, ACSL4, NT5E |
| --- | --- | --- | --- | --- |
| GOTERM_CC_FAT | GO:0042641~actomyosin | 4 | 8.77E-03 | ACTA1, MYH11, MYH9, ANXA2 |
| GOTERM_CC_FAT | GO:0031982~vesicle | 17 | 8.79E-03 | ALDOA, AGFG1, NAP1L1, SLC3A2, AP3S1, ATP6V1B2,  CD2AP, ANXA2, LNPEP, LAMP1, CORO1A, TFRC, IGF2R,  CAPG, MYH11, EHD1, HIP1 |
| GOTERM_CC_FAT | GO:0005859~muscle myosin complex | 3 | 1.04E-02 | MYH11, MYH7, TTN |
| GOTERM_CC_FAT | GO:0048471~perinuclear region of  cytoplasm | 9 | 1.08E-02 | LNPEP, HMGB2, MTDH, TFRC, PAK2, SORBS2, TPD52,  CD2AP, ANXA2 |
| GOTERM_CC_FAT | GO:0005884~actin filament | 4 | 1.18E-02 | EZR, ACTA1, LCP1, TPM4 |
| GOTERM_CC_FAT | GO:0044449~contractile fiber part | 6 | 1.20E-02 | DES, ACTA1, PDLIM5, MYH11, MYH7, TTN |
| GOTERM_CC_FAT | GO:0005925~focal adhesion | 5 | 1.27E-02 | PTPRC, TLN1, LASP1, ARPC2, ZYX |
| GOTERM_CC_FAT | GO:0070161~anchoring junction | 7 | 1.38E-02 | PTPRC, TLN1, LASP1, ARPC2, ZYX, MYH9, CXADR |
| GOTERM_CC_FAT | GO:0005783~endoplasmic reticulum | 23 | 1.41E-02 | SCD1, RTN4, SGPL1, TMX1, MTDH, CES1, CYP2B9, CYP3A11,  CKAP4, CMAH, TPD52, SERPINH1, ALDH3A2, TAPBP,  CYP4A10, CYP7B1, PTRF, PLOD1, SH3GLB1, P4HA1, HMOX1,  FMO3, ACSL4 |
| GOTERM_CC_FAT | GO:0016460~myosin II complex | 3 | 1.50E-02 | MYH11, MYH7, TTN |
| GOTERM_CC_FAT | GO:0030016~myofibril | 6 | 1.50E-02 | DES, ACTA1, PDLIM5, MYH11, MYH7, TTN |
| GOTERM_CC_FAT | GO:0031410~cytoplasmic vesicle | 16 | 1.58E-02 | ALDOA, AGFG1, NAP1L1, SLC3A2, AP3S1, ATP6V1B2,  CD2AP, ANXA2, LAMP1, CORO1A, TFRC, IGF2R, CAPG,  MYH11, EHD1, HIP1 |
| GOTERM_CC_FAT | GO:0016459~myosin complex | 5 | 1.60E-02 | MYL6, MYH11, MYH7, MYH9, TTN |
| GOTERM_CC_FAT | GO:0005924~cell-substrate adherens | 5 | 1.60E-02 | PTPRC, TLN1, LASP1, ARPC2, ZYX |
| GOTERM_CC_FAT | GO:0009898~internal side of plasma  membrane | 11 | 1.66E-02 | CDC42, TLN1, STX12, EZR, SNX1, AP3S1, RRAS, MSN, EHD1,  GBP2, ALDH3A2 |
| GOTERM_CC_FAT | GO:0001772~immunological synapse | 3 | 1.75E-02 | CORO1A, MYH9, ARHGDIA |
| GOTERM_CC_FAT | GO:0043292~contractile fiber | 6 | 1.78E-02 | DES, ACTA1, PDLIM5, MYH11, MYH7, TTN |
| GOTERM_CC_FAT | GO:0019898~extrinsic to membrane | 15 | 1.88E-02 | CYP2B9, CYP3A11, ATP6V1B2, CYP4A10, CYP7B1, EZR,  GNAQ, PLIN2, SH3GLB1, CAP1, MSN, GGA1, EHD1, EHD4, |

SH3GL1

| GOTERM_CC_FAT | GO:0005578~proteinaceous extracellular  matrix | 11 | 2.06E-02 | LGALS3BP, BGN, COL14A1, LUM, TGFBI, HSPG2, TGM2,  COL6A1, PRELP, FN1, ANXA2 |
| --- | --- | --- | --- | --- |
| GOTERM_CC_FAT | GO:0030055~cell-substrate junction | 5 | 2.08E-02 | PTPRC, TLN1, LASP1, ARPC2, ZYX |
| GOTERM_CC_FAT | GO:0005886~plasma membrane | 60 | 2.11E-02 | TLN1, AP3S1, CXADR, IQGAP1, TAPBP, LNPEP, CD47, CDC42,  DES, ARHGAP1, TUBB5, RRAS, PIK3AP1, MSN, ZYX, CAP1,  NT5E, CAPNS1, LYN, BST2, SLC3A2, CLIC1, MYH9, FLNA,  ATP6V1C1, ATP6V1A, BGN, CD36, GNAQ, PTRF, MAPRE1,  GBP2, LCP1, MTDH, SNX1, ATP6V1B2, ALDH3A2, STX12,  EZR, PLIN2, ARPC2, TGM2, COL6A1, EHD1, ARHGDIA, FN1,  PTPRC, PTPN6, LPP, SWAP70, AK1, ANXA1, IGH-3, CAPN2,  ANXA2, LAMP1, CORO1A, TFRC, LASP1, ATP6V1E1 |
| GOTERM_CC_FAT | GO:0016323~basolateral plasma membrane | 7 | 2.52E-02 | PTPRC, TLN1, LASP1, ARPC2, ANXA1, ZYX, MSN |
| GOTERM_CC_FAT | GO:0031012~extracellular matrix | 11 | 2.61E-02 | LGALS3BP, BGN, COL14A1, LUM, TGFBI, HSPG2, TGM2,  COL6A1, PRELP, FN1, ANXA2 |
| GOTERM_CC_FAT | GO:0030017~sarcomere | 5 | 3.86E-02 | DES, ACTA1, PDLIM5, MYH7, TTN |
| GOTERM_CC_FAT | GO:0045098~type III intermediate filament | 2 | 4.70E-02 | DES, VIM |
| GOTERM_CC_FAT | GO:0030062~mitochondrial tricarboxylic  acid cycle enzyme complex | 2 | 4.70E-02 | BCKDHA, BCKDHB |
| GOTERM_CC_FAT | GO:0030687~preribosome, large subunit  precursor | 2 | 4.70E-02 | CASP8, PES1 |
| GOTERM_CC_FAT | GO:0005947~mitochondrial alpha-  ketoglutarate dehydrogenase complex | 2 | 4.70E-02 | BCKDHA, BCKDHB |
| GOTERM_CC_FAT | GO:0045240~dihydrolipoyl dehydrogenase  complex | 2 | 4.70E-02 | BCKDHA, BCKDHB |
| GOTERM_CC_FAT | GO:0030864~cortical actin cytoskeleton | 3 | 4.73E-02 | LASP1, CFL1, CAP1 |
| GOTERM_CC_FAT | GO:0000785~chromatin | 7 | 4.83E-02 | SMCHD1, HIST1H1D, HIST1H1C, HIST1H1B, HIST1H1A,  MECP2, TMPO |
| GOTERM_MF_FAT | GO:0003779~actin binding | 29 | 4.51E-14 | TLN1, CNN3, CAPZA1, ARPC5, TPM4, PFN1, EZR, ARPC2,  CAP1, HIP1, DBNL, TWF1, MYH7, MYH9, COTL1, FLNA,  CORO1C, ARPC1B, CORO1A, YWHAH, LASP1, CFL1, CAPG,  MYH11, FLII, MARCKS, WDR1, LCP1, MYLK |

| GOTERM_MF_FAT | GO:0008092~cytoskeletal protein binding | 32 | 2.08E-12 | TLN1, CNN3, CAPZA1, ARPC5, TPM4, PFN1, EZR, ARPC2,  MSN, CAP1, HIP1, DBNL, TWF1, MYH7, MYH9, COTL1, FLNA,  ANXA2, CORO1C, ARPC1B, CORO1A, YWHAH, LASP1, CFL1,  CAPG, MYH11, FLII, MARCKS, MAPRE1, WDR1, LCP1, MYLK |
| --- | --- | --- | --- | --- |
| GOTERM_MF_FAT | GO:0051015~actin filament binding | 8 | 1.56E-05 | DBNL, CORO1A, EZR, LASP1, MYH7, MYH9, LCP1, FLNA |
| GOTERM_MF_FAT | GO:0005543~phospholipid binding | 11 | 3.01E-05 | SNX6, SNX5, PITPNA, CPNE1, SNX2, ANXA1, SNX1, ANXA5,  ANXA3, HIP1, ANXA2 |
| GOTERM_MF_FAT | GO:0016765~transferase activity,  transferring alkyl or aryl (other than methyl) | 7 | 2.42E-04 | GSTA2, GSTM2, GSTM3, MAT2A, FDPS, GSTM6, GSTP1 |
| GOTERM_MF_FAT | groups  GO:0050662~coenzyme binding | 11 | 3.55E-04 | XDH, HAO1, ME1, CTBP1, EHHADH, PGD, LDHD, FMO3,  HAO2, NQO1, DHTKD1 |
| GOTERM_MF_FAT | GO:0048037~cofactor binding | 13 | 4.19E-04 | ME1, XDH, SGPL1, CTBP1, EHHADH, PGD, LDHD, HAO1,  HAO2, FMO3, NQO1, DHTKD1, PYGB |
| GOTERM_MF_FAT | GO:0008289~lipid binding | 16 | 6.94E-04 | SNX6, RBP1, SNX5, PITPNA, ANXA1, SNX2, SNX1, ANXA5,  ANXA3, ANXA2, CD36, SH3GLB1, CPNE1, FABP5, SH3GL1,  HIP1 |
| GOTERM_MF_FAT | GO:0004364~glutathione transferase activity | 5 | 8.49E-04 | GSTA2, GSTM2, GSTM3, GSTM6, GSTP1 |
| GOTERM_MF_FAT | GO:0042625~ATPase activity, coupled to  transmembrane movement of ions | 7 | 1.01E-03 | ATP6V0C, ATP6V1C1, ATP6V1A, ATP6V1E1, ATP6V1H,  ATP11C, ATP6V1B2 |
| GOTERM_MF_FAT | GO:0046961~proton-transporting ATPase  activity, rotational mechanism | 4 | 1.80E-03 | ATP6V1A, ATP6V1E1, ATP6V1H, ATP6V1B2 |
| GOTERM_MF_FAT | GO:0004859~phospholipase inhibitor | 3 | 2.69E-03 | ANXA1, ANXA3, ANXA2 |
| GOTERM_MF_FAT | GO:0055102~lipase inhibitor activity | 3 | 2.69E-03 | ANXA1, ANXA3, ANXA2 |
| GOTERM_MF_FAT | GO:0005516~calmodulin binding | 8 | 3.00E-03 | CNN3, MYH11, MARCKS, MYH7, MYH9, TTN, IQGAP1, MYLK |
| GOTERM_MF_FAT | GO:0019904~protein domain specific  binding | 10 | 4.99E-03 | KHDRBS1, PTPN6, SH3BGRL, HMGB2, YWHAH, LYN,  ARHGAP1, TGM2, UBE2I, CD2AP |
| GOTERM_MF_FAT | GO:0005506~iron ion binding | 14 | 5.14E-03 | XDH, SCD1, STX7, CYP2B9, CYP3A11, CMAH, CYP4A10,  CYP7B1, PLOD1, P4HA1, HMOX1, HEBP1, CYGB, NT5E |
| GOTERM_MF_FAT | GO:0016832~aldehyde-lyase activity | 3 | 5.52E-03 | ALDOA, SGPL1, ALDOC |
| GOTERM_MF_FAT | GO:0042626~ATPase activity, coupled to  transmembrane movement of substances | 7 | 5.69E-03 | ATP6V0C, ATP6V1C1, ATP6V1A, ATP6V1E1, ATP6V1H,  ATP11C, ATP6V1B2 |

| GOTERM_MF_FAT | GO:0043492~ATPase activity, coupled to  movement of substances | 7 | 5.69E-03 | ATP6V0C, ATP6V1C1, ATP6V1A, ATP6V1E1, ATP6V1H,  ATP11C, ATP6V1B2 |
| --- | --- | --- | --- | --- |
| GOTERM_MF_FAT | GO:0016820~hydrolase activity, acting on  acid anhydrides, catalyzing transmembrane  movement of substances | 7 | 5.69E-03 | ATP6V0C, ATP6V1C1, ATP6V1A, ATP6V1E1, ATP6V1H,  ATP11C, ATP6V1B2 |
| GOTERM_MF_FAT | GO:0005544~calcium-dependent  phospholipid binding | 4 | 7.19E-03 | ANXA1, ANXA5, ANXA3, ANXA2 |
| GOTERM_MF_FAT | GO:0008307~structural constituent of | 3 | 7.28E-03 | MYL6, MYH11, TTN |
| GOTERM_MF_FAT | GO:0019829~cation-transporting ATPase | 4 | 8.07E-03 | ATP6V1A, ATP6V1E1, ATP6V1H, ATP6V1B2 |
| GOTERM_MF_FAT | GO:0035091~phosphoinositide binding | 6 | 8.33E-03 | SNX6, SNX5, SNX2, SNX1, HIP1, ANXA2 |
| GOTERM_MF_FAT | GO:0015405~P-P-bond-hydrolysis-driven  transmembrane transporter activity | 7 | 9.07E-03 | ATP6V0C, ATP6V1C1, ATP6V1A, ATP6V1E1, ATP6V1H,  ATP11C, ATP6V1B2 |
| GOTERM_MF_FAT | GO:0016624~oxidoreductase activity, acting  on the aldehyde or oxo group of donors,  disulfide as acceptor | 3 | 9.26E-03 | BCKDHA, BCKDHB, DHTKD1 |
| GOTERM_MF_FAT | GO:0015399~primary active transmembrane  transporter activity | 7 | 9.47E-03 | ATP6V0C, ATP6V1C1, ATP6V1A, ATP6V1E1, ATP6V1H,  ATP11C, ATP6V1B2 |
| GOTERM_MF_FAT | GO:0017124~SH3 domain binding | 6 | 1.03E-02 | KHDRBS1, PTPN6, SH3BGRL, LYN, ARHGAP1, CD2AP |
| GOTERM_MF_FAT | GO:0015078~hydrogen ion transmembrane  transporter activity | 6 | 1.20E-02 | ATP6V0C, ATP6V1C1, ATP6V1A, ATP6V1E1, ATP6V1H,  ATP6V1B2 |
| GOTERM_MF_FAT | GO:0005200~structural constituent of  cytoskeleton | 4 | 1.22E-02 | TLN1, DES, TUBB5, TTN |
| GOTERM_MF_FAT | GO:0015077~monovalent inorganic cation  transmembrane transporter activity | 6 | 1.52E-02 | ATP6V0C, ATP6V1C1, ATP6V1A, ATP6V1E1, ATP6V1H,  ATP6V1B2 |
| GOTERM_MF_FAT | GO:0019842~vitamin binding | 7 | 1.59E-02 | SGPL1, PLOD1, RBP1, P4HA1, ACACA, DHTKD1, PYGB |
| GOTERM_MF_FAT | GO:0000166~nucleotide binding | 50 | 1.65E-02 | XDH, ME1, RALY, DYNC1LI1, PGD, LDHD, NAGK, TTN,  ACSS3, CMPK2, CKB, CDC42, PAK2, ACTR1A, SULT1A1,  FMO3, TUBB5, ABCD2, TGM2, RRAS, ACSL4, EHD1, NT5E,  EHD4, CTBP1, SMCHD1, MAT2A, ACTA1, LYN, SWAP70, AK1,  MECP2, ACACA, UBE2I, MYH7, ATP11C, MYH9, HNRNPA1,  BLVRA, HAO1, RAB32, ATP6V1A, ACSM1, GNAQ, HAO2,  RRM1, CPNE1, MYH11, GBP2, MYLK |

| GOTERM_MF_FAT | GO:0022890~inorganic cation  transmembrane transporter activity | 7 | 1.98E-02 | ATP6V0C, ATP6V1C1, ATP6V1A, TFRC, ATP6V1E1, ATP6V1H,  ATP6V1B2 |
| --- | --- | --- | --- | --- |
| GOTERM_MF_FAT | GO:0009055~electron carrier activity | 9 | 2.03E-02 | XDH, CYP4A10, HAO1, CYP7B1, CYP2B9, CYP3A11, HAO2,  CMAH, NQO1 |
| GOTERM_MF_FAT | GO:0004197~cysteine-type endopeptidase | 5 | 2.10E-02 | CAPNS1, LGMN, CASP8, TTN, CAPN2 |
| GOTERM_MF_FAT | GO:0008553~hydrogen-exporting ATPase  activity, phosphorylative mechanism | 3 | 2.22E-02 | ATP6V0C, ATP6V1C1, ATP6V1E1 |
| GOTERM_MF_FAT | GO:0016290~palmitoyl-CoA hydrolase | 3 | 2.53E-02 | ACOT9, ACOT2, ACOT3 |
| GOTERM_MF_FAT | GO:0004198~calcium-dependent cysteine-  type endopeptidase activity | 3 | 2.53E-02 | CAPNS1, TTN, CAPN2 |
| GOTERM_MF_FAT | GO:0005484~SNAP receptor activity | 3 | 2.86E-02 | STX8, STX12, STX7 |
| GOTERM_MF_FAT | GO:0003826~alpha-ketoacid dehydrogenase  activity | 2 | 3.31E-02 | BCKDHA, BCKDHB |
| GOTERM_MF_FAT | GO:0003973~(S)-2-hydroxy-acid oxidase | 2 | 3.31E-02 | HAO1, HAO2 |
| GOTERM_MF_FAT | GO:0042623~ATPase activity, coupled | 9 | 3.39E-02 | MYL6, ATP6V0C, ATP6V1C1, ATP6V1A, ATP6V1E1,  ATP6V1H, ATP11C, ATP6V1B2, MYH9 |
| GOTERM_MF_FAT | GO:0020037~heme binding | 7 | 3.39E-02 | CYP4A10, CYP7B1, CYP2B9, HMOX1, CYP3A11, HEBP1, |
| GOTERM_MF_FAT | GO:0004091~carboxylesterase activity | 6 | 3.47E-02 | ACOT9, CES1, PPME1, ACOT2, ABHD12, ACOT3 |
| GOTERM_MF_FAT | GO:0046906~tetrapyrrole binding | 7 | 4.12E-02 | CYP4A10, CYP7B1, CYP2B9, HMOX1, CYP3A11, HEBP1, |
| GOTERM_MF_FAT | GO:0001948~glycoprotein binding | 3 | 4.33E-02 | PTPRC, IGF2R, FLNA |
| GOTERM_MF_FAT | GO:0016291~acyl-CoA thioesterase activity | 3 | 4.33E-02 | ACOT9, ACOT2, ACOT3 |
| GOTERM_MF_FAT | GO:0016887~ATPase activity | 10 | 4.66E-02 | MYL6, ATP6V0C, ATP6V1C1, ATP6V1A, ATP6V1E1, ABCD2,  ATP6V1H, ATP11C, ATP6V1B2, MYH9 |
| GOTERM_MF_FAT | GO:0016899~oxidoreductase activity, acting  on the CH-OH group of donors, oxygen as | 2 | 4.93E-02 | HAO1, HAO2 |
| GOTERM_MF_FAT | acceptor  GO:0003863~3-methyl-2-oxobutanoate  dehydrogenase (2-methylpropanoyl-  transferring) activity | 2 | 4.93E-02 | BCKDHA, BCKDHB |
| GOTERM_MF_FAT | GO:0047961~glycine N-acyltransferase | 2 | 4.93E-02 | GLYAT, KEG1 |
| GOTERM_MF_FAT | GO:0005094~Rho GDP-dissociation  inhibitor activity | 2 | 4.93E-02 | ARHGDIA, ARHGDIB |
| GOTERM_MF_FAT | GO:0019834~phospholipase A2 inhibitor | 2 | 4.93E-02 | ANXA1, ANXA3 |

| KEGG_PATHWAY | mmu00480:Glutathione metabolism | 8 | 2.07E-04 | GSTA2, GSTM2, GSTM3, PGD, G6PDX, RRM1, GSTM6, GSTP1 |
| --- | --- | --- | --- | --- |
| KEGG_PATHWAY | mmu04810:Regulation of actin cytoskeleton | 14 | 1.90E-03 | CDC42, ARPC1B, PFN1, EZR, PAK2, ARPC2, CFL1, RRAS,  MSN, ARPC5, MYH9, MYLK, IQGAP1, FN1 |
| KEGG_PATHWAY | mmu00982:Drug metabolism | 8 | 1.95E-03 | GSTA2, GSTM2, GSTM3, CYP2B9, CYP3A11, FMO3, GSTM6,  GSTP1 |
| KEGG_PATHWAY | mmu04666:Fc gamma R-mediated  phagocytosis | 9 | 2.22E-03 | CDC42, ARPC1B, PTPRC, LYN, ARPC2, CFL1, IGH-3,  MARCKS, ARPC5 |
| KEGG_PATHWAY | mmu00620:Pyruvate metabolism | 6 | 2.71E-03 | ME1, AKR1B7, LDHD, ACACA, GLO1, ALDH3A2 |
| KEGG_PATHWAY | mmu00980:Metabolism of xenobiotics by  cytochrome P450 | 7 | 4.66E-03 | GSTA2, GSTM2, GSTM3, CYP2B9, CYP3A11, GSTM6, GSTP1 |
| KEGG_PATHWAY | mmu04142:Lysosome | 9 | 7.29E-03 | ATP6V0C, LAMP1, IGF2R, LGMN, ATP6V1H, AP3S1, GGA1,  MAN2B1, GLB1 |
| KEGG_PATHWAY | mmu03320:PPAR signaling pathway | 7 | 1.11E-02 | CYP4A10, SCD1, ME1, CD36, EHHADH, ACSL4, FABP5 |
| KEGG_PATHWAY | mmu00030:Pentose phosphate pathway | 4 | 2.32E-02 | ALDOA, ALDOC, PGD, G6PDX |
| KEGG_PATHWAY | mmu00280:Valine, leucine and isoleucine  degradation | 5 | 2.34E-02 | BCKDHA, EHHADH, BCKDHB, HMGCS1, ALDH3A2 |
| KEGG_PATHWAY | mmu01040:Biosynthesis of unsaturated fatty  acids | 4 | 2.56E-02 | ACOT9, SCD1, ACOT2, ACOT3 |
| KEGG_PATHWAY | mmu00900:Terpenoid backbone biosynthesis | 3 | 4.27E-02 | FDPS, HMGCS1, IDI1 |
| KEGG_PATHWAY | mmu04510:Focal adhesion | 10 | 4.65E-02 | CDC42, TLN1, PAK2, COL6A1, ZYX, CAPN2, THBS1, MYLK,  FLNA, FN1 |
| KEGG_PATHWAY | mmu00903:Limonene and pinene  degradation | 3 | 4.85E-02 | ACOT9, EHHADH, ALDH3A2 |
