# Supplementary material for: Hepatitis B virus X induces inflammation and cancer in mice liver through dysregulation of cytoskeletal remodeling and lipid metabolism
Source: Oncotarget. 2016 Sep 30;7(43):70559–74. doi: 10.18632/oncotarget.12372 (PMC5342574; doi:10.18632/oncotarget.12372)
Supplement: Supplementary file 8 [file oncotarget-07-70559-s008.docx]

**Table 8S. GO analysis for HBx interacting proteins**

| **Category** | **Term** | **P-Value** | **Genes** |
| --- | --- | --- | --- |
| GOTERM_BP_FAT | cytoskeleton organization | 1.57E-06 | INA, LIMA1, KIF11, MAP1B, ARF6, DOCK7, ARPC4, ARPC5, TPM1, CTNNB1,  ARPC1A, PPP1R9B, ANK3, SVIL, AMOT, NEFL, NEFM, TMOD1 |
| GOTERM_BP_FAT | nuclear mRNA splicing, via  spliceosome | 3.00E-06 | SFRS7, DDX39, POLR2E, SF3B14, CD2BP2, SNRPD3, SNRPD1, HNRNPD,  SFRS13A, SNRPA, DDX20 |
| GOTERM_BP_FAT | RNA splicing | 2.70E-05 | POLR2E, SF3B14, SNRPD3, SFRS13A, SNRPD1, NONO, DDX39, PPP1R9B,  SFRS7, CD2BP2, HNRNPD, SNRPA, DDX20 |
| GOTERM_BP_FAT | regulation of actin cytoskeleton | 2.94E-05 | ARPC1A, LIMA1, ACTR3B, AMOT, SPTBN1, ARF6, ARPC5, TPM1 |
| GOTERM_BP_FAT | regulation of organelle organization | 3.25E-04 | ARPC1A, LIMA1, ACTR3B, MAP1B, AMOT, SPTBN1, ARF6, ARPC5, TPM1,  CTNNB1 |
| GOTERM_BP_FAT | mRNA ing | 3.70E-04 | NONO, SFRS7, DDX39, POLR2E, SF3B14, CD2BP2, SNRPD3, SNRPD1,  HNRNPD, SFRS13A, SNRPA, DDX20 |
| GOTERM_BP_FAT | cell cycle | 6.98E-04 | ARL2, LOC642954, SPIN1, KIF11, NASP, MRE11A, UBE2I, SKP1, CTNNB1,  PPP1R9B, PSMA1, PPP1CA, CHMP1A, PSMB6, HSPA2, PSMB1, PSMB2, |
| GOTERM_BP_FAT | cell projection organization | 1.15E-03 | CAMK2D, MCTS1  EFHD1, PPP1R9B, LIMA1, ATXN10, ANK3, KIF5C, MAP1B, DOCK7, ARF6,  ARPC4, NEFL, TPM1 |
| GOTERM_BP_FAT | mRNA metabolic | 1.20E-03 | NONO, SFRS7, DDX39, POLR2E, SF3B14, CD2BP2, SNRPD3, SNRPD1,  HNRNPD, SFRS13A, SNRPA, DDX20 |
| GOTERM_BP_FAT | actin cytoskeleton organization | 1.93E-03 | ARPC1A, PPP1R9B, LIMA1, AMOT, ARF6, ARPC4, ARPC5, TPM1, TMOD1 |
| GOTERM_BP_FAT | regulation of protein complex assembly | 2.05E-03 | ARPC1A, ACTR3B, MAP1B, SPTBN1, ARF6, ARPC5 |
| GOTERM_BP_FAT | spliceosomal snRNP biogenesis | 2.65E-03 | SNRPD3, SNRPD1, SFRS13A, DDX20 |
| GOTERM_BP_FAT | neurofilament cytoskeleton | 2.66E-03 | INA, NEFL, NEFM |
| GOTERM_BP_FAT | ubiquitin-dependent protein catabolic | 2.95E-03 | PSMA1, PSMB6, PSMB1, PSMB2, FBXO22OS, LOC100132973, UBR3, UBE2I, |
| GOTERM_BP_FAT | ruffle organization | 3.40E-03 | LIMA1, ARF6, TPM1 |
| GOTERM_BP_FAT | microtubule cytoskeleton organization | 3.54E-03 | KIF11, MAP1B, DOCK7, ARPC4, NEFL, NEFM, CTNNB1 |
| GOTERM_BP_FAT | cellular component morphogenesis | 6.44E-03 | SSBP1, ANK3, KIF5C, MAP1B, DOCK7, ARPC4, NEFL, TPM1, CTNNB1,  HNRNPAB, TMOD1 |

| GOTERM_BP_FAT | positive regulation of protein | 9.89E-03 | PSMA1, PSMB6, PSMB1, PSMB2, SKP1 |
| --- | --- | --- | --- |
| GOTERM_BP_FAT | positive regulation of cytoskeleton  organization | 1.02E-02 | MAP1B, AMOT, ARF6, TPM1 |
| GOTERM_BP_FAT | mitochondrion organization | 1.24E-02 | GGCT, SSBP1, MTX2, TOMM22, TIMM23, NDUFAF3 |
| GOTERM_BP_FAT | microtubule-based | 1.35E-02 | KIF11, KIF5C, MAP1B, DOCK7, ARPC4, NEFL, NEFM, CTNNB1 |
| GOTERM_BP_FAT | intracellular transport | 1.42E-02 | SRP14, MTX2, SFRS13A, TIMM23, TPM1, DDX39, ARF1, MYL6B, RAB11A,  SPTBN1, TOM1, TOMM22, NEFL, NEFM |
| GOTERM_BP_FAT | neuron projection development | 1.43E-02 | EFHD1, PPP1R9B, ATXN10, ANK3, KIF5C, MAP1B, DOCK7, NEFL |
| GOTERM_BP_FAT | cellular macromolecular complex | 1.44E-02 | ARL2, SNRPD3, SNRPD1, SFRS13A, TOMM22, ARPC4, DDX20, NEFL, |
| GOTERM_BP_FAT | cytoskeleton-dependent intracellular  transport | 1.50E-02 | MYL6B, NEFL, TPM1, NEFM |
| GOTERM_BP_FAT | macromolecular complex assembly | 1.55E-02 | ARL2, POLR2E, SNRPD3, SFRS13A, SNRPD1, ARPC4, NDUFAF3, CTNNB1,  MAZ, THRAP3, TCEB2, TOMM22, DDX20, NEFL |
| GOTERM_BP_FAT | intermediate filament cytoskeleton  organization | 1.67E-02 | INA, NEFL, NEFM |
| GOTERM_BP_FAT | chromatin remodeling | 1.83E-02 | LOC642954, HDAC2, NASP, ACTL6A |
| GOTERM_BP_FAT | muscle organ development | 1.91E-02 | MYL6B, SVIL, FHL1, LMNA, TAGLN2, CSRP2, TPM1 |
| GOTERM_BP_FAT | intermediate filament-based | 2.00E-02 | INA, NEFL, NEFM |
| GOTERM_BP_FAT | negative regulation of RNA metabolic | 2.87E-02 | CHMP1A, HDAC2, CSDAP1, SFRS13A, UBE2I, PAWR, DDX20, CTNNB1, |
| GOTERM_BP_FAT | axon regeneration in the peripheral  nervous system | 2.96E-02 | MAP1B, NEFL |
| GOTERM_BP_FAT | regulation of cell projection size | 2.96E-02 | NEFL, NEFM |
| GOTERM_BP_FAT | in utero embryonic development | 3.16E-02 | PRMT1, TJP1, CSDAP1, NASP, AMOT, TPM1 |
| GOTERM_BP_FAT | mitotic cell cycle | 3.20E-02 | PSMA1, CHMP1A, KIF11, PSMB6, PSMB1, PSMB2, CAMK2D, UBE2I, SKP1 |
| GOTERM_BP_FAT | positive regulation of protein metabolic | 3.50E-02 | PSMA1, PSMB6, HDAC2, PSMB1, PSMB2, DOCK7, SKP1 |
| GOTERM_BP_FAT | cell morphogenesis involved in  differentiation | 3.56E-02 | ANK3, KIF5C, MAP1B, DOCK7, NEFL, CTNNB1, HNRNPAB |
| GOTERM_BP_FAT | assembly of spliceosomal tri-snRNP | 3.93E-02 | SFRS13A, DDX20 |
| GOTERM_BP_FAT | positive regulation of protein | 3.94E-02 | PSMA1, PSMB6, PSMB1, PSMB2, DOCK7, SKP1 |
| GOTERM_BP_FAT | negative regulation of transcription | 4.04E-02 | CHMP1A, BCLAF1, HDAC2, CSDAP1, ZNF24, UBE2I, PAWR, DDX20,  CTNNB1, HNRNPAB |
| GOTERM_BP_FAT | protein targeting to mitochondrion | 4.50E-02 | MTX2, TOMM22, TIMM23 |

| GOTERM_BP_FAT | protein localization in mitochondrion | 4.50E-02 | MTX2, TOMM22, TIMM23 |
| --- | --- | --- | --- |
| GOTERM_BP_FAT | positive regulation of glycoprotein  biosynthetic | 4.89E-02 | PAWR, CTNNB1 |
| GOTERM_BP_FAT | androgen receptor signaling pathway | 4.99E-02 | THRAP3, FHL2, CTNNB1 |
| GOTERM_BP_FAT | neuron development | 5.34E-02 | EFHD1, PPP1R9B, ATXN10, ANK3, KIF5C, MAP1B, DOCK7, NEFL |
| GOTERM_BP_FAT | regulation of cell size | 5.55E-02 | PPP1R9B, FHL1, MAP1B, AMOT, NDUFS3, CSRP2 |
| GOTERM_BP_FAT | modification-dependent macromolecule  catabolic | 6.13E-02 | PSMA1, PSMB6, PSMB1, PSMB2, TCEB2, FBXO22OS, LOC100132973, UBR3,  UBE2I, SKP1, UBE2T |
| GOTERM_BP_FAT | regulation of myeloid leukocyte  differentiation | 6.30E-02 | NME2, CA2, CTNNB1 |
| GOTERM_BP_FAT | negative regulation of protein complex  disassembly | 6.30E-02 | LIMA1, MAP1B, SPTBN1 |
| GOTERM_BP_FAT | cell morphogenesis | 6.58E-02 | ANK3, KIF5C, MAP1B, DOCK7, ARPC4, NEFL, CTNNB1, HNRNPAB |
| GOTERM_BP_FAT | negative regulation of transcription,  DNA-dependent | 6.58E-02 | CHMP1A, HDAC2, CSDAP1, UBE2I, PAWR, DDX20, CTNNB1, HNRNPAB |
| GOTERM_BP_FAT | protein complex biogenesis | 6.60E-02 | ARL2, MAZ, POLR2E, THRAP3, TCEB2, TOMM22, ARPC4, NEFL, NDUFAF3,  CTNNB1 |
| GOTERM_BP_FAT | oxidative phosphorylation | 7.47E-02 | NDUFA5, NDUFA6, ATP5L, NDUFS3 |
| GOTERM_BP_FAT | regulation of protein modification | 7.52E-02 | PPP1R9B, PSMA1, PSMB6, PSMB1, PSMB2, DOCK7, SKP1 |
| GOTERM_BP_FAT | cellular protein complex assembly | 7.90E-02 | ARL2, TOMM22, ARPC4, NEFL, NDUFAF3 |
| GOTERM_BP_FAT | proteasomal ubiquitin-dependent  protein catabolic | 8.20E-02 | PSMA1, PSMB6, PSMB1, PSMB2 |
| GOTERM_BP_FAT | phosphagen metabolic | 8.63E-02 | CKMT1A, CKB |
| GOTERM_BP_FAT | creatine metabolic | 8.63E-02 | CKMT1A, CKB |
| GOTERM_BP_FAT | actin-mediated cell contraction | 8.63E-02 | MYL6B, TPM1 |
| GOTERM_BP_FAT | macromolecule catabolic | 9.02E-02 | UBR3, UBE2I, SKP1, PSMA1, TXNDC12, PSMB6, PSMB1, PSMB2,  LOC100132973, TCEB2, HNRNPD, FBXO22OS, UBE2T |
| GOTERM_BP_FAT | regulation of protein complex | 9.19E-02 | LIMA1, MAP1B, SPTBN1 |
| GOTERM_BP_FAT | generation of precursor metabolites and  energy | 9.34E-02 | NDUFA5, LDHB, PPP1CA, NDUFA6, SLC25A3, ATP5L, NDUFS3 |
| GOTERM_BP_FAT | positive regulation of epithelial cell  differentiation | 9.55E-02 | NME2, CTNNB1 |

| GOTERM_BP_FAT | cell projection morphogenesis | 9.87E-02 | ANK3, KIF5C, MAP1B, DOCK7, ARPC4, NEFL |
| --- | --- | --- | --- |
| GOTERM_CC_FAT | cytoskeleton | 3.41E-08 | SPIN1, LIMA1, ACTR3B, FHL2, ARPC4, C14ORF166, ARPC5, TPM1, CTNNB1,  LOC652798, CTTN, MYL6B, ANK3, ACTR1B, DDX20, NEFL, NEFM, ARL2,  INA, KIF11, EPPK1, FLOT1, MAP1B, KIF5C, C21ORF33, LMNA, RAI14,  ARPC1A, PPP1R9B, NME2, CHMP1A, SVIL, AMOT, TOM1, SPTBN1, FLII, |
| GOTERM_CC_FAT | mitochondrion | 4.42E-06 | TMOD1  GGCT, FHL1, SNRPD3, SNRPD1, CKB, CTNNB1, PSMB6, PSMB1, EIF1AX,  PSMB2, HNRNPD, CAMK2D, LOC100132973, DDX20, NEFL, ARL2, MAP1B,  LOC645870, HBA2, SKP1, APRT, PSMA1, PFDN2, NME2, PPP1CA, ARF1,  CSNK1E, EEF1E1, TCEB2, TOM1, SPTBN1, DUT |
| GOTERM_CC_FAT | contractile fiber | 1.05E-04 | NDUFA5, NDUFA6, LMNA, TIMM23, NDUFAF3, SLC25A11, EFHD1,  CKMT1A, SLC25A3, ATP5L, TOMM22, HADH, NDUFS3 |
| GOTERM_CC_FAT | organelle envelope | 2.00E-04 | ARF1, MYL6B, SVIL, FHL2, SPTBN1, TPM1, CTNNB1, TMOD1 |
| GOTERM_CC_FAT | contractile fiber part | 8.26E-04 | ARF1, SVIL, FHL2, SPTBN1, TPM1, CTNNB1, TMOD1 |
| GOTERM_CC_FAT | membrane-enclosed lumen | 9.65E-04 | PSMA1, PSMB6, PSMB1, PSMB2 |
| GOTERM_CC_FAT | organelle lumen | 1.43E-03 | INA, NEFL, NEFM |
| GOTERM_CC_FAT | neuron projection | 1.76E-03 | ARL2, NME2, FLOT1, ACTR1B, C21ORF33, TOM1, FLII, C14ORF166, CTNNB1 |
| GOTERM_CC_FAT | nucleoplasm | 2.16E-03 | PPP1R9B, PPP1CA, ATXN10, ANK3, KIF5C, MAP1B, DOCK7, CA2, NEFL,  NEFM, CTNNB1 |
| GOTERM_CC_FAT | chromatin remodeling complex | 3.34E-03 | PPP1R9B, CTTN, RAI14, SPTBN1, ARF6, CTNNB1, TMOD1 |
| GOTERM_CC_FAT | nuclear lumen | 5.41E-03 | LOC642954, KIF11, HDAC2, SAP18, ACTL6A |
| GOTERM_CC_FAT | apicolateral plasma membrane | 1.70E-02 | LOC652798, TJP1, MPP5, AMOT, CTNNB1 |
| GOTERM_CC_FAT | nucleoplasm part | 2.11E-02 | ANK3, MAP1B, DOCK7, CA2, NEFL, NEFM |
| GOTERM_CC_FAT | proteasome complex | 2.23E-02 | LOC642954, PPP1CA, POLR2E, HDAC2, STK38, THRAP3, SFRS13A, SAP18,  UBE2I, ACTL6A, DDX20, CTNNB1 |
| GOTERM_CC_FAT | ruffle | 2.34E-02 | LIMA1, AMOT, TPM1 |
| GOTERM_CC_FAT | actomyosin | 2.89E-02 | CTTN, NME2, ARF6, TPM1 |
| GOTERM_CC_FAT | basolateral plasma membrane | 3.24E-02 | CTTN, NME2, AMOT, CTNNB1 |
| GOTERM_CC_FAT | protein serine/threonine phosphatase  complex | 5.18E-02 | TJP1, LIMA1, ANK3, FHL2, CA2, CTNNB1 |
| GOTERM_CC_FAT | histone deacetylase complex | 5.98E-02 | PPP1R9B, PPM1B, PPP2R2A |
| GOTERM_CC_FAT | transcriptional repressor complex | 6.25E-02 | LOC642954, HDAC2, SAP18 |
| GOTERM_CC_FAT | NADH dehydrogenase complex | 6.25E-02 | LOC642954, HDAC2, DDX20 |

| GOTERM_CC_FAT | mitochondrial respiratory chain | 6.52E-02 | NDUFA5, NDUFA6, NDUFS3 |
| --- | --- | --- | --- |
| GOTERM_CC_FAT | organelle membrane | 6.75E-02 | ARPC4, ARPC5 |
| GOTERM_CC_FAT | MLL5-L complex | 7.64E-02 | NDUFA5, NDUFA6, MTX2, LMNA, TIMM23, TAGLN2, NDUFAF3, EFHD1,  SLC25A11, CKMT1A, ARF1, CAMK2D, SLC25A3, ATP5L, TOMM22, HADH, |
| GOTERM_CC_FAT | Sin3 complex | 7.68E-02 | NDUFS3  PPP1CA, STK38 |
| GOTERM_CC_FAT | anchoring junction | 7.68E-02 | LOC642954, HDAC2 |
| GOTERM_MF_FAT | cytoskeletal protein binding | 8.77E-04 | LIMA1, ACTR3B, SSFA2, MAP1B, ARPC4, ARPC5, TPM1, ARPC1A, PPP1R9B,  NME2, SVIL, SPTBN1, FLII, POF1B, TMOD1 |
| GOTERM_MF_FAT | structural constituent of cytoskeleton | 1.09E-03 | INA, SPTBN1, ARPC5, NEFL, TPM1, NEFM |
| GOTERM_MF_FAT | threonine-type endopeptidase activity | 1.15E-03 | PSMA1, PSMB6, PSMB1, PSMB2 |
| GOTERM_MF_FAT | creatine kinase activity | 4.16E-02 | CKMT1A, CKB |
| GOTERM_MF_FAT | transcription repressor activity | 5.01E-02 | BCLAF1, HDAC2, CSDAP1, ZNF24, SAP18, UBE2I, PAWR, DDX20 |
| GOTERM_MF_FAT | protein domain specific binding | 6.12E-02 | CHMP1A, TJP1, SFRS13A, UBE2I, LRRFIP2, PAWR, DDX20, NEFL |
| GOTERM_MF_FAT | acid-amino acid ligase activity | 6.17E-02 | UBL4A, FBXO22OS, UBR3, UBE2I, ARPC4, UBE2T |
| GOTERM_MF_FAT | NADH dehydrogenase activity | 7.54E-02 | NDUFA5, NDUFA6, NDUFS3 |
| GOTERM_MF_FAT | NADH dehydrogenase (ubiquinone) | 7.54E-02 | NDUFA5, NDUFA6, NDUFS3 |
| GOTERM_MF_FAT | NADH dehydrogenase (quinone) | 7.54E-02 | NDUFA5, NDUFA6, NDUFS3 |
| GOTERM_MF_FAT | LRR domain binding | 8.14E-02 | LRRFIP2, PAWR |
| GOTERM_MF_FAT | protein serine/threonine phosphatase  activity | 8.16E-02 | PPP1CA, PPM1B, PPP2R2A |
| GOTERM_MF_FAT | oxidoreductase activity, | 9.43E-02 | NDUFA5, NDUFA6, NDUFS3 |
| GOTERM_MF_FAT | ligase activity, forming carbon-nitrogen  bonds | 9.78E-02 | UBL4A, FBXO22OS, UBR3, UBE2I, ARPC4, UBE2T |
| GOTERM_MF_FAT | small conjugating protein ligase activity | 9.88E-02 | UBL4A, FBXO22OS, UBR3, UBE2I, UBE2T |
