# Supplementary material for: Hepatitis B virus X induces inflammation and cancer in mice liver through dysregulation of cytoskeletal remodeling and lipid metabolism
Source: Oncotarget. 2016 Sep 30;7(43):70559–74. doi: 10.18632/oncotarget.12372 (PMC5342574; doi:10.18632/oncotarget.12372)
Supplement: Supplementary file 9 [file oncotarget-07-70559-s009.docx]

**Table 9S. The biochemical characteristics of clinical tissue samples on HBV-HCC, non-HBV–HCC patients**

| **Number** | **Sex** | **Age**  （**year**） | **History**  **of** | **Family**  **history of** | **Distant**  **metastase** | **Primary**  **organ** | **Patholog**  **ical type** | **TNM**  **stages** | **History of liver**  **disease** | **Alcoholis**  **m(Yes/No** | **Preoperative laboratory tests** | | | | | | | | |
| --- | --- | --- | --- | --- | --- | --- | --- | --- | --- | --- | --- | --- | --- | --- | --- | --- | --- | --- | --- |
|  |  |  |  |  |  |  |  |  |  |  | **AFP (ng/ml)** | **HBcAb** | **HBeAb** | **HBeAg** | **HBsAb** | **HBsAg** | **HBVDNA** | **HCVRNA** | **CEA(ng/ml)** |
| 1 | M | 55 | No | No | No | Yes | HCC | T3N0M0 | HBV | No | 27.32 | ＋ | － | － | － | ＋ | ＋ | － | 1.21 |
| 2 | M | 44 | No | No | No | Yes | HCC | T2N0M0 | HBV | No | 12.37 | ＋ | ＋ | － | － | ＋ | ＋ | － | 3.12 |
| 3 | M | 42 | No | No | No | Yes | HCC | T3N0M0 | HBV | No | 22.48 | ＋ | ＋ | － | － | ＋ | ＋ | － | 0.89 |
| 4 | M | 54 | No | No | No | Yes | HCC | T2NxM0 | HBV | No | 6.06 | ＋ | ＋ | － | － | ＋ | ＋ | － | 2.35 |
| 5 | F | 37 | No | No | No | Yes | HCC | T3N0M0 | HBV | No | 3000.00 | ＋ | － | － | － | ＋ | ＋ | － | 3.19 |
| 6 | M | 53 | No | No | No | Yes | HCC | T2N0M0 | HBV | No | 5.62 | ＋ | － | ＋ | － | ＋ | ＋ | － | 4.23 |
| 7 | M | 37 | No | No | No | Yes | HCC | T2N0M0 | HBV | No | 74.38 | ＋ | － | ＋ | － | ＋ | ＋ | － | 2.79 |
| 8 | F | 63 | No | No | No | Yes | HCC | T2N0M0 | HBV | No | 300.00 | ＋ | － | － | － | ＋ | ＋ | － | 5.99 |
| 9 | M | 59 | No | No | No | Yes | HCC | T2N0M0 | HBV | No | 110.11 | ＋ | － | － | － | ＋ | ＋ | － | 1.68 |
| 10 | F | 52 | No | No | No | Yes | HCC | T4NxM0 | HBV | No | 121.36 | ＋ | － | － | － | ＋ | ＋ | － | 1.14 |
| 11 | M | 53 | No | No | No | Yes | HCC | T3NxM0 | HBV | No | 56.74 | ＋ | － | － | － | ＋ | ＋ | － | 2.92 |
| 12 | F | 60 | No | No | No | Yes | HCC | T2N0M0 | HBV | No | 182.91 | ＋ | － | ＋ | － | ＋ | ＋ | － | 3.54 |
| 13 | F | 47 | No | No | No | Yes | HCC | T4NxM0 | HBV | No | 5.68 | ＋ | － | － | － | ＋ | ＋ | － | 2.94 |
| 14 | M | 65 | No | No | No | Yes | HCC | T2N0M0 | HBV | No | 16.79 | ＋ | － | ＋ | － | ＋ | ＋ | － | 2.32 |
| 15 | M | 55 | No | No | No | Yes | HCC | T3N0M0 | HBV | No | 27.32 | ＋ | － | － | － | ＋ | ＋ | － | 1.21 |
| 16 | M | 44 | No | No | No | Yes | HCC | T2N0M0 | HBV | No | 12.37 | ＋ | ＋ | － | － | ＋ | ＋ | － | 3.12 |
| 17 | M | 42 | No | No | No | Yes | HCC | T3N0M0 | HBV | No | 22.48 | ＋ | ＋ | － | － | ＋ | ＋ | － | 0.89 |
| 18 | M | 54 | No | No | No | Yes | HCC | T2NxM0 | HBV | No | 6.06 | ＋ | ＋ | － | － | ＋ | ＋ | － | 2.35 |
| 19 | F | 37 | No | No | No | Yes | HCC | T3N0M0 | HBV | No | 29.21 | ＋ | － | － | － | ＋ | ＋ | － | 3.19 |
| 20 | M | 53 | No | No | No | Yes | HCC | T2N0M0 | HBV | No | 5.62 | ＋ | － | ＋ | － | ＋ | ＋ | － | 4.23 |
| 21 | M | 37 | No | No | No | Yes | HCC | T2N0M0 | HBV | No | 74.38 | ＋ | － | ＋ | － | ＋ | ＋ | － | 2.79 |
| 22 | F | 63 | No | No | No | Yes | HCC | T2N0M0 | HBV | No | 288.00 | ＋ | － | － | － | ＋ | ＋ | － | 5.99 |
| 23 | M | 59 | No | No | No | Yes | HCC | T2N0M0 | HBV | No | 110.11 | ＋ | － | － | － | ＋ | ＋ | － | 1.68 |
| 24 | F | 52 | No | No | No | Yes | HCC | T4NxM0 | HBV | No | 121.36 | ＋ | － | － | － | ＋ | ＋ | － | 1.14 |
| 25 | M | 53 | No | No | No | Yes | HCC | T3NxM0 | HBV | No | 56.74 | ＋ | － | － | － | ＋ | ＋ | － | 2.92 |
| 26 | F | 60 | No | No | No | Yes | HCC | T2N0M0 | HBV | No | 182.91 | ＋ | － | ＋ | － | ＋ | ＋ | － | 3.54 |
| 27 | F | 47 | No | No | No | Yes | HCC | T4NxM0 | HBV | No | 5.68 | ＋ | － | － | － | ＋ | ＋ | － | 2.94 |
| 28 | M | 65 | No | No | No | Yes | HCC | T2N0M0 | HBV | No | 16.79 | ＋ | － | ＋ | － | ＋ | ＋ | － | 2.32 |
| 29 | M | 49 | No | No | No | Yes | HCC | T2N0M0 | HBV | No | 87.12 | ＋ | ＋ | － | － | ＋ | ＋ | － | 3.23 |
| 30 | M | 57 | No | No | No | Yes | HCC | T3N0M0 | HBV | No | 57.32 | ＋ | － | － | － | ＋ | ＋ | － | 2.21 |
| 31 | M | 47 | No | No | No | Yes | HCC | T3N0M0 | HBV | No | 29.48 | ＋ | - | － | － | ＋ | ＋ | － | 1.23 |
| 32 | M | 57 | No | No | No | Yes | HCC | T3N0M0 | HBV | No | 33.86 | ＋ | ＋ | － | － | ＋ | ＋ | － | 2.84 |
| 33 | M | 48 | No | No | No | Yes | HCC | T3N0M0 | HBV | No | 285.00 | ＋ | ＋ | － | － | ＋ | + | － | 5.39 |
| 34 | M | 49 | No | No | No | Yes | HCC | T3N0M0 | HBV | No | 4.98 | － | － | － | － | ＋ | + | － | 4.70 |
| 35 | M | 61 | No | No | No | Yes | HCC | T3N0M0 | HBV | No | 117.48 | － | － | － | － | ＋ | + | － | 4.15 |
| 36 | M | 62 | No | No | No | Yes | HCC | T2N0M0 | HBV | No | 56.44 | ＋ | ＋ | － | － | ＋ | + | － | 1.77 |
| 37 | M | 43 | No | No | No | Yes | HCC | T1N0M0 | HBV | No | 23.45 | ＋ | ＋ | － | － | ＋ | + | － | 1.78 |
| 38 | M | 45 | No | No | No | Yes | HCC | T2N0M0 | HBV | No | 40.46 | － | ＋ | － | － | ＋ | + | － | 1.29 |
| 39 | M | 68 | No | No | No | Yes | HCC | T2NxM0 | HBV | No | 84.76 | － | ＋ | － | － | ＋ | + | － | 2.58 |
| 40 | M | 39 | No | No | No | Yes | HCC | T2NxM0 | HBV | No | 7.66 | ＋ | ＋ | － | － | ＋ | + | － | 1.90 |
| 41 | M | 47 | No | No | No | Yes | HCC | T1N0M0 | HBV | No | 961.40 | ＋ | ＋ | － | － | ＋ | + | － | 2.03 |
| 42 | M | 47 | No | No | No | Yes | HCC | T2N0M0 | HBV | No | 22.36 | － | ＋ | － | － | ＋ | + | － | 3.08 |
| 43 | M | 48 | No | No | No | Yes | HCC | T3N0M0 | HBV | No | 961.40 | ＋ | ＋ | － | － | ＋ | + | － | 2.39 |
| 44 | M | 42 | No | No | No | Yes | HCC | T4NxMx | HBV | No | 84.24 | ＋ | ＋ | － | － | ＋ | + | － | 4.69 |
| 45 | M | 34 | No | No | No | Yes | HCC | T2N0M0 | HBV | No | 7.25 | － | ＋ | － | － | ＋ | + | － | 2.03 |

| 46 | M | 42 | No | No | No | Yes | HCC | T3NxM0 | HBV | No | 118.95 | ＋ | ＋ | － | － | ＋ | + | － | 2.15 |
| --- | --- | --- | --- | --- | --- | --- | --- | --- | --- | --- | --- | --- | --- | --- | --- | --- | --- | --- | --- |
| 47 | M | 54 | No | No | No | Yes | HCC | T1N0M0 | HBV | No | 271.61 | － | － | － | － | ＋ | + | － | 3.68 |
| 48 | M | 35 | No | No | No | Yes | HCC | T2N0M0 | HBV | No | 8.96 | ＋ | ＋ | － | － | ＋ | + | － | 3.70 |
| 49 | M | 45 | No | No | No | Yes | HCC | T3N0M0 | HBV | No | 5.02 | ＋ | － | ＋ | － | ＋ | + | － | 1.11 |
| 50 | F | 44 | No | No | No | Yes | HCC | T2N0M0 | HBV | No | 59.64 | ＋ | － | － | － | ＋ | + | － | 2.24 |
| 51 | M | 54 | No | No | No | Yes | HCC | T2N0Mx | HBV | No | 19.11 | ＋ | ＋ | － | － | ＋ | + | － | 0.99 |
| 52 | M | 44 | No | No | No | Yes | HCC | T3NxM0 | HBV | No | 2850.00 | ＋ | － | － | － | ＋ | + | － | 1.50 |
| 53 | M | 61 | No | No | No | Yes | HCC | T1NxM0 | HBV | No | 22.33 | ＋ | － | － | － | ＋ | + | － | 2.98 |
| 54 | M | 48 | No | No | No | Yes | HCC | T3N0M0 | HBV | No | 285.00 | ＋ | ＋ | － | － | ＋ | + | － | 5.39 |
| 55 | M | 49 | No | No | No | Yes | HCC | T3N0M0 | HBV | No | 4.98 | － | － | － | － | ＋ | + | － | 4.70 |
| 56 | M | 61 | No | No | No | Yes | HCC | T3N0M0 | HBV | No | 117.48 | － | － | － | － | ＋ | + | － | 4.15 |
| 57 | M | 62 | No | No | No | Yes | HCC | T2N0M0 | HBV | No | 56.44 | ＋ | ＋ | － | － | ＋ | + | － | 1.77 |
| 58 | M | 43 | No | No | No | Yes | HCC | T1N0M0 | HBV | No | 23.45 | ＋ | ＋ | － | － | ＋ | + | － | 1.78 |
| 59 | M | 45 | No | No | No | Yes | HCC | T2N0M0 | HBV | No | 40.46 | － | ＋ | － | － | ＋ | + | － | 1.29 |
| 60 | M | 68 | No | No | No | Yes | HCC | T2NxM0 | HBV | No | 84.76 | － | ＋ | － | － | ＋ | + | － | 2.58 |
| 61 | M | 39 | No | No | No | Yes | HCC | T2NxM0 | HBV | No | 7.66 | ＋ | ＋ | － | － | ＋ | + | － | 1.90 |
| 62 | M | 47 | No | No | No | Yes | HCC | T1N0M0 | HBV | No | 961.40 | ＋ | ＋ | － | － | ＋ | + | － | 2.03 |
| 63 | M | 47 | No | No | No | Yes | HCC | T2N0M0 | HBV | No | 22.36 | － | ＋ | － | － | ＋ | + | － | 3.08 |
| 64 | M | 48 | No | No | No | Yes | HCC | T3N0M0 | HBV | No | 961.40 | ＋ | ＋ | － | － | ＋ | + | － | 2.39 |
| 65 | M | 42 | No | No | No | Yes | HCC | T4NxMx | HBV | No | 84.24 | ＋ | ＋ | － | － | ＋ | + | － | 4.69 |
| 66 | M | 34 | No | No | No | Yes | HCC | T2N0M0 | HBV | No | 7.25 | － | ＋ | － | － | ＋ | + | － | 2.03 |
| 67 | M | 42 | No | No | No | Yes | HCC | T3NxM0 | HBV | No | 118.95 | ＋ | ＋ | － | － | ＋ | + | － | 2.15 |
| 68 | M | 54 | No | No | No | Yes | HCC | T1N0M0 | HBV | No | 271.61 | － | － | － | － | ＋ | + | － | 3.68 |
| 69 | M | 35 | No | No | No | Yes | HCC | T2N0M0 | HBV | No | 8.96 | ＋ | ＋ | － | － | ＋ | + | － | 3.70 |
| 70 | M | 45 | No | No | No | Yes | HCC | T3N0M0 | HBV | No | 5.02 | ＋ | － | ＋ | － | ＋ | + | － | 1.11 |
| 71 | F | 44 | No | No | No | Yes | HCC | T2N0M0 | HBV | No | 59.64 | ＋ | － | － | － | ＋ | + | － | 2.24 |
| 72 | M | 54 | No | No | No | Yes | HCC | T2N0Mx | HBV | No | 19.11 | ＋ | ＋ | － | － | ＋ | + | － | 0.99 |
| 73 | M | 44 | No | No | No | Yes | HCC | T3NxM0 | HBV | No | 2850.00 | ＋ | － | － | － | ＋ | + | － | 1.50 |
| 74 | M | 61 | No | No | No | Yes | HCC | T1NxM0 | HBV | No | 22.33 | ＋ | － | － | － | ＋ | + | － | 2.98 |
| 75 | M | 57 | No | No | No | Yes | HCC | T3N0M0 | HBV | No | 60.36 | － | ＋ | － | － | ＋ | + | － | 4.88 |
| 76 | M | 40 | No | No | No | Yes | HCC | T2N0Mx | HBV | No | 3.13 | ＋ | ＋ | － | － | ＋ | + | － | 4.36 |
| 77 | M | 40 | No | No | No | Yes | HCC | T2N0Mx | HBV | No | 3.13 | ＋ | ＋ | － | － | ＋ | + | － | 4.36 |
| 78 | M | 33 | No | No | No | Yes | HCC | T2N0M0 | HBV | No | 8.34 | － | ＋ | － | － | ＋ | + | － | 2.33 |
| 79 | M | 41 | No | No | No | Yes | HCC | T3NxM0 | HBV | No | 136.79 | ＋ | ＋ | － | － | ＋ | + | － | 2.47 |
| 80 | M | 53 | No | No | No | Yes | HCC | T1N0M0 | HBV | No | 312.35 | － | － | － | － | ＋ | + | － | 4.23 |
| 81 | M | 34 | No | No | No | Yes | HCC | T2N0M0 | HBV | No | 10.30 | ＋ | ＋ | － | － | ＋ | + | － | 4.25 |
| 82 | M | 44 | No | No | No | Yes | HCC | T3N0M0 | HBV | No | 5.77 | ＋ | － | ＋ | － | ＋ | + | － | 1.27 |
| 83 | F | 43 | No | No | No | Yes | HCC | T2N0M0 | HBV | No | 68.59 | ＋ | － | － | － | ＋ | + | － | 2.58 |
| 84 | M | 53 | No | No | No | Yes | HCC | T2N0Mx | HBV | No | 21.98 | ＋ | ＋ | － | － | ＋ | + | － | 1.14 |
| 85 | M | 43 | No | No | No | Yes | HCC | T3NxM0 | HBV | No | 3277.50 | ＋ | － | － | － | ＋ | + | － | 1.73 |
| 86 | M | 60 | No | No | No | Yes | HCC | T1NxM0 | HBV | No | 25.67 | ＋ | － | － | － | ＋ | + | － | 3.43 |
| 87 | M | 56 | No | No | No | Yes | HCC | T3N0M0 | HBV | No | 69.42 | － | ＋ | － | － | ＋ | + | － | 5.61 |
| 88 | M | 39 | No | No | No | Yes | HCC | T2N0Mx | HBV | No | 3.59 | ＋ | ＋ | － | － | ＋ | + | － | 5.01 |
| 89 | M | 39 | No | No | No | Yes | HCC | T2N0Mx | HBV | No | 3.59 | ＋ | ＋ | － | － | ＋ | + | － | 5.01 |
| 90 | M | 48 | No | No | No | Yes | HCC | T2N0M0 | HBV | No | 11.12 | ＋ | ＋ | － | － | ＋ | ＋ | － | 3.23 |
| 91 | M | 68 | No | No | No | Yes | HCC | T3N0M0 | HCV | No | 30.23 | － | － | － | ＋ | － | － | ＋ | 3.80 |
| 92 | M | 54 | No | No | No | Yes | HCC | T2N0M0 | HCV | No | 48.21 | － | － | － | - | － | － | ＋ | 5.99 |
| 93 | M | 42 | No | No | No | Yes | HCC | T3N0M0 | HCV | No | 46.80 | － | － | － | － | － | － | ＋ | 5.99 |
| 94 | F | 59 | No | No | No | Yes | HCC | T2N0M0 | HCV | No | 18.26 | － | － | － | － | － | － | ＋ | 3.26 |
| 95 | F | 43 | No | No | No | Yes | HCC | T2N0M0 | HBV | No | 56.42 | ＋ | ＋ | － | ＋ | ＋ | － | ＋ | 2.13 |

| 96 | F | 43 | No | No | No | Yes | HCC | T2N0M0 | HBV | No | 56.42 | ＋ | ＋ | － | ＋ | ＋ | － | ＋ | 2.13 |
| --- | --- | --- | --- | --- | --- | --- | --- | --- | --- | --- | --- | --- | --- | --- | --- | --- | --- | --- | --- |
| 97 | M | 68 | No | No | No | Yes | HCC | T3N0M0 | HCV | No | 37.79 | － | － | － | ＋ | － | － | ＋ | 3.80 |
| 98 | M | 54 | No | No | No | Yes | HCC | T2N0M0 | HCV | No | 60.26 | － | － | － | - | － | － | ＋ | 6.55 |
| 99 | M | 42 | No | No | No | Yes | HCC | T3N0M0 | HCV | No | 58.50 | － | － | － | － | － | － | ＋ | 5.99 |
| 100 | F | 59 | No | No | No | Yes | HCC | T2N0M0 | HCV | No | 22.83 | － | － | － | － | － | － | ＋ | 3.26 |
| 101 | F | 43 | No | No | No | Yes | HCC | T2N0M0 | HCV | No | 70.53 | ＋ | ＋ | － | ＋ | ＋ | － | ＋ | 2.13 |
| 102 | F | 43 | No | No | No | Yes | HCC | T2N0M0 | HCV | No | 74.23 | ＋ | ＋ | － | ＋ | ＋ | － | ＋ | 2.13 |
| 103 | M | 67 | No | No | No | Yes | HCC | T2N0M0 | HCV | No | 35.52 | － | － | － | ＋ | － | － | ＋ | 3.99 |
| 104 | M | 55 | No | No | No | Yes | HCC | T2N0M0 | HCV | No | 45.32 | － | － | － | - | － | － | ＋ | 6.29 |
| 105 | M | 41 | No | No | No | Yes | HCC | T2N0M0 | HCV | No | 43.99 | － | － | － | － | － | － | ＋ | 5.28 |
| 106 | F | 62 | No | No | No | Yes | HCC | T2N0M0 | HCV | No | 17.16 | － | － | － | － | － | － | ＋ | 3.42 |
| 107 | F | 48 | No | No | No | Yes | HCC | T2N0M0 | HCV | No | 53.03 | ＋ | ＋ | － | ＋ | ＋ | － | ＋ | 2.24 |
| 108 | F | 64 | No | No | No | Yes | HCC | T2N0M0 | HCV | No | 53.03 | ＋ | ＋ | － | ＋ | ＋ | － | ＋ | 2.81 |
| 109 | M | 55 | No | No | No | Yes | HCC | T2N0M0 | HCV | No | 28.42 | － | － | － | ＋ | － | － | ＋ | 2.51 |
| 110 | M | 66 | No | No | No | Yes | HCC | T2N0M0 | HCV | No | 45.32 | － | － | － | - | － | － | ＋ | 4.84 |
| 111 | M | 43 | No | No | No | Yes | HCC | T2N0M0 | HCV | No | 43.99 | － | － | － | － | － | － | ＋ | 6.23 |
| 112 | F | 61 | No | No | No | Yes | HCC | T2N0M0 | HCV | No | 17.16 | － | － | － | － | － | － | ＋ | 4.77 |
| 113 | F | 53 | No | No | No | Yes | HCC | T2N0M0 | HCV | No | 53.03 | ＋ | ＋ | － | ＋ | ＋ | － | ＋ | 5.21 |
| 114 | M | 56 | No | No | No | Yes | HCC | T2N0M0 | HCV | No | 58.42 | － | － | － | - | － | － | ＋ | 4.32 |
| 115 | M | 53 | No | No | No | Yes | HCC | T2N0M0 | Non-HBV ＆ HCV | No | 85.97 | － | － | － | － | － | － | － | 1.55 |
| 116 | M | 48 | No | No | No | Yes | HCC | T2N0M0 | Non-HBV ＆ HCV | No | 187.12 | － | － | － | - | － | － | － | 4.23 |
| 117 | M | 52 | No | No | No | Yes | HCC | T3N0M0 | Non-HBV ＆ HCV | No | 57.12 | － | － | － | - | － | － | － | 0.98 |
| 118 | F | 65 | No | No | No | Yes | HCC | T2N0M0 | Non-HBV ＆ HCV | No | 2050.00 | － | － | － | － | － | － | － | 0.73 |
| 119 | M | 53 | No | No | No | Yes | HCC | TxNxMx | Non-HBV ＆ HCV | No | 58.96 | － | － | － | ＋ | － | － | － | 1.97 |
| 120 | M | 53 | No | No | No | Yes | HCC | T2N0M0 | Non-HBV ＆ HCV | No | 3.04 | － | － | － | － | － | － | － | 1.01 |
| 121 | M | 36 | No | No | No | Yes | HCC | T2N0M0 | Non-HBV ＆ HCV | No | 15.82 | － | － | － | － | － | － | － | 2.22 |
| 122 | M | 55 | No | No | No | Yes | HCC | T2N0M0 | Non-HBV ＆ HCV | No | 120.36 | － | － | － | － | － | － | － | 1.43 |
| 123 | M | 63 | No | No | No | Yes | HCC | T2N0M0 | Non-HBV ＆ HCV | No | 261.97 | － | － | － | - | － | － | － | 4.51 |
| 124 | M | 48 | No | No | No | Yes | HCC | T3N0M0 | Non-HBV ＆ HCV | No | 79.97 | － | － | － | - | － | － | － | 1.89 |
| 125 | F | 47 | No | No | No | Yes | HCC | T2N0M0 | Non-HBV ＆ HCV | No | 2870.00 | － | － | － | － | － | － | － | 2.73 |
| 126 | M | 51 | No | No | No | Yes | HCC | TxNxMx | Non-HBV ＆ HCV | No | 82.54 | － | － | － | ＋ | － | － | － | 2.22 |
| 127 | M | 56 | No | No | No | Yes | HCC | T2N0M0 | Non-HBV ＆ HCV | No | 4.26 | － | － | － | － | － | － | － | 3.15 |
| 128 | M | 60 | No | No | No | Yes | HCC | T2N0M0 | Non-HBV ＆ HCV | No | 77.37 | － | － | － | － | － | － | － | 6.28 |
| 129 | M | 59 | No | No | No | Yes | HCC | T2N0M0 | Non-HBV ＆ HCV | No | 168.41 | － | － | － | － | － | － | － | 4.44 |
| 130 | M | 54 | No | No | No | Yes | HCC | T2N0M0 | Non-HBV ＆ HCV | No | 51.41 | － | － | － | - | － | － | － | 3.15 |
| 131 | M | 52 | No | No | No | Yes | HCC | T3N0M0 | Non-HBV ＆ HCV | No | 1845.00 | － | － | － | - | － | － | － | 1.98 |
| 132 | F | 61 | No | No | No | Yes | HCC | T2N0M0 | Non-HBV ＆ HCV | No | 53.06 | － | － | － | － | － | － | － | 1.76 |
| 133 | M | 51 | No | No | No | Yes | HCC | T2N0M0 | Non-HBV ＆ HCV | No | 2.74 | － | － | － | ＋ | － | － | － | 1.87 |
| 134 | M | 52 | No | No | No | Yes | HCC | T2N0M0 | Non-HBV ＆ HCV | No | 14.24 | － | － | － | － | － | － | － | 2.59 |
| 135 | F | 48 | No | No | No | Yes | HCC | T2N0M0 | Non-HBV ＆ HCV | No | 22.12 | － | － | － | － | － | － | － | 3.73 |
