# Supplementary material for: Hepatitis B virus X induces inflammation and cancer in mice liver through dysregulation of cytoskeletal remodeling and lipid metabolism
Source: Oncotarget. 2016 Sep 30;7(43):70559–74. doi: 10.18632/oncotarget.12372 (PMC5342574; doi:10.18632/oncotarget.12372)
Supplement: Supplementary file 10 [file oncotarget-07-70559-s010.docx]

**Table 10S. The biochemical characteristics of serum samples on HBV-HCC, non-HBV–HCC patients**

| **Number** | **Sex** | **Age (year)** | **Feature** | **ALT(U/L)** | **AST(U/L)** | **TBIL(μ**  **mol/L)** | **DBIL(μ**  **mol/L)** | **IBIL** | **ALP(U/L)** | **LDH(U/L)** | **GGT(U/L)** |
| --- | --- | --- | --- | --- | --- | --- | --- | --- | --- | --- | --- |
| 1 | M | 61 | Health | 22 | 32 | 10.00 | 2.10 | 3.10 | 71.10 | 110.10 | 25.30 |
| 2 | M | 35 | Health | 23 | 33 | 8.90 | 2.30 | 4.10 | 68.20 | 113.40 | 22.40 |
| 3 | M | 33 | Health | 15 | 21 | 8.80 | 1.90 | 2.70 | 55.90 | 121.50 | 19.80 |
| 4 | F | 39 | Health | 18 | 25 | 7.20 | 3.60 | 3.30 | 47.10 | 143.20 | 16.20 |
| 5 | F | 38 | Health | 16 | 24 | 6.30 | 2.20 | 3.90 | 52.30 | 127.80 | 15.20 |
| 6 | F | 36 | Health | 15 | 23 | 21.00 | 4.10 | 4.70 | 57.80 | 135.40 | 17.20 |
| 7 | F | 56 | HCV | 20 | 18 | 10.00 | 2.00 | 7.83 | 73.28 | 165.47 | 14.70 |
| 8 | M | 20 | HCV | 65 | 52 | 22.00 | 9.00 | 13.44 | 134.14 | 155.94 | 6.79 |
| 9 | F | 41 | HCV | 74 | 54 | 23.00 | 6.00 | 16.59 | 91.04 | 205.65 | 115.75 |
| 10 | F | 42 | HCV | 24 | 27 | 6.00 | 2.00 | 4.32 | 65.89 | 145.17 | 22.20 |
| 11 | F | 60 | HCV | 32 | 34 | 14.00 | 4.00 | 10.07 | 86.76 | 131.23 | 81.81 |

| 12 | M | 40 | HBV-HCC | 196 | 65 | 26.00 | 9.00 | 17.27 | 90.21 | 229.41 | 88.43 |
| --- | --- | --- | --- | --- | --- | --- | --- | --- | --- | --- | --- |
| 13 | M | 60 | HCC | 22 | 45 | 65.00 | 38.00 | 26.95 | 137.88 | 187.83 | 69.56 |
| 14 | M | 53 | HBV-HCC | 47 | 29 | 11.00 | 4.00 | 6.81 | 84.20 | 171.96 | 27.71 |
| 15 | F | 58 | HBV-HCC | 25 | 42 | 28.00 | 12.00 | 16.43 | 92.85 | 175.95 | 28.46 |
| 16 | M | 70 | HBV-HCC | 54 | 66 | 32.00 | 16.00 | 16.35 | 108.00 | 179.82 | 81.44 |
| 17 | F | 71 | HBV-HCC | 31 | 26 | 12.00 | 4.00 | 8.05 | 88.37 | 137.58 | 45.46 |
| 18 | M | 46 | HBV | 59 | 66 | 27.00 | 11.00 | 15.70 | 94.35 | 209.71 | 58.02 |
| 19 | M | 69 | HBV | 60 | 37 | 17.00 | 6.00 | 11.41 | 96.66 | 151.18 | 89.13 |
| 20 | M | 29 | HBV | 63 | 29 | 17.00 | 5.00 | 11.50 | 73.57 | 155.80 | 37.42 |
| 21 | M | 62 | HBV | 30 | 53 | 19.00 | 6.00 | 13.03 | 53.81 | 211.54 | 19.32 |
| 22 | M | 42 | HBV | 306 | 230 | 20.00 | 7.00 | 12.87 | 116.80 | 175.89 | 100.88 |
| 23 | M | 60 | HBV | 63 | 37 | 7.00 | 3.00 | 4.41 | 60.80 | 117.50 | 28.08 |
| 24 | M | 40 | HBV | 284 | 102 | 23.00 | 10.00 | 12.92 | 125.52 | 128.41 | 382.47 |

| 25 | M | 42 | HBV | 196 | 295 | 33.00 | 14.00 | 19.10 | 133.50 | 237.80 | 399.26 |
| --- | --- | --- | --- | --- | --- | --- | --- | --- | --- | --- | --- |
| 26 | M | 26 | HBV | 42 | 21 | 8.00 | 3.00 | 5.14 | 81.76 | 142.90 | 99.37 |
| 27 | M | 16 | HBV | 119 | 56 | 6.00 | 2.00 | 3.92 | 169.19 | 147.87 | 60.56 |
| 28 | M | 31 | HBV | 470 | 117 | 15.00 | 6.00 | 19.50 | 79.52 | 161.81 | 240.73 |
| 29 | M | 20 | HBV | 42 | 28 | 9.00 | 4.00 | 5.00 | 52.00 | 171.70 | 17.00 |
| 30 | M | 22 | HBV | 22 | 16 | 19.00 | 7.00 | 12.38 | 66.93 | 143.52 | 46.90 |
| 31 | M | 47 | HBV | 46 | 20 | 11.00 | 4.00 | 7.59 | 80.03 | 92.80 | 33.31 |
| 32 | F | 61 | HBV | 22 | 21 | 27.00 | 11.00 | 15.87 | 71.16 | 142.12 | 17.77 |
| 33 | F | 24 | HBV | 28 | 25 | 11.00 | 3.00 | 8.09 | 80.75 | 138.62 | 18.60 |
| 34 | F | 19 | HBV | 46 | 22 | 6.00 | 3.00 | 3.67 | 47.18 | 118.60 | 12.81 |
| 35 | F | 29 | HBV | 110 | 48 | 7.00 | 2.00 | 5.02 | 55.32 | 144.56 | 26.30 |
| 36 | F | 21 | HBV | 18 | 14 | 13.00 | 3.00 | 9.51 | 45.59 | 145.52 | 9.30 |
| 37 | F | 65 | HBV | 25 | 24 | 18.00 | 6.00 | 12.74 | 102.75 | 206.81 | 30.89 |

| 38 | M | 24 | Drug-  Induced  Liver Injury | 115 | 37 | 15.00 | 4.00 | 10.91 | 86.43 | 170.23 | 79.88 |
| --- | --- | --- | --- | --- | --- | --- | --- | --- | --- | --- | --- |
| 39 | M | 57 | Drug-  Induced  Liver Injury | 78 | 29 | 25.00 | 10.00 | 14.85 | 35.48 | 161.14 | 18.68 |
| 40 | F | 58 | Drug-  Induced  Liver Injury | 89 | 47 | 9.00 | 2.00 | 6.26 | 81.64 | 157.19 | 77.28 |
